# Telomere-Associated Changes in Nuclear Architecture of Cancer-Associated Macrophage-like Cells in Liquid Biopsies from Melanoma Patients

**DOI:** 10.3390/biomedicines10102391

**Published:** 2022-09-25

**Authors:** Aline Rangel-Pozzo, Janine Wechsler, Jessica Groult, Laetitia Da Meda, Celeste Lebbe, Sabine Mai

**Affiliations:** 1 CancerCare Manitoba Research Institute, University of Manitoba, Winnipeg, MB R3C 2B1, Canada; 2 Screencell Company, 62 rue de Wattignies, F-75012 Paris, France; 3 INSERM U976, Team 1, HIPI, Université de Paris, F-75010 Paris, France; 4 Service de Dermatologie, AP-HP Hôpital Saint Louis, F-75010 Paris, France

**Keywords:** tumor-associated macrophages, circulating tumor cells, cancer-associated macrophage-like cells, telomere-related genomic instability, metastatic cutaneous melanoma stage IV

## Abstract

During phagocytosis, tumor-associated macrophages (TAMs) can incorporate genetic material from tumor cells. The incorporation of extra genetic material may be responsible for advanced malignant behavior observed in some TAMs, making TAMs potentially important players in cancer progression. More recently, similar cells were described in the blood as cancer-associated macrophage-like cells (CAMLs). CAMLs may be equivalent to TAMs cells in the blood, and they express macrophage markers. However, their origin is still unclear. In a previous study, we showed for the first time the distinct telomere 3D structure of circulating tumor cells (CTCs) in melanoma and other cancers. In the present pilot study, we investigated, comparatively, the 3D telomere structure of CAMLs, CTCs and leucocytes from nine melanoma patients with metastatic cutaneous melanoma stage IV. CTC capture was performed by size-based filtration followed by cytological and immunocytological evaluation. Three-dimensional Quantitative Fluorescent in situ Hybridization was performed to measure differences in five 3D telomere parameters. Telomere parameters, such as number, length, telomere aggregates, nuclear volume, and *a*/*c* ratio, were compared among different cellular types (CTCs, CAMLs, and normal leucocytes). Three telomere parameters were significantly different between CAMLs and leucocytes. The combination of two telomere parameters (telomere length against the number of telomeres) resulted in the identification of two CAMLs subpopulations with different levels of genomic instability. Those populations were classified as profile 1 and 2. Profile 2, characterized by a high number of short telomeres, was observed in four of the nine melanoma patients. To our knowledge, this is the first pilot study to investigate 3D telomere parameters as hallmarks of nuclear architecture in CAMLs’ population in comparison to leucocytes from the same patient. Further studies involving a larger patient sample size are necessary to validate these findings and explore their potential prognostic value.

## 1. Introduction

Macrophages play a central role in antigen presentation and inflammation [1]. Together with fibroblasts, vascular endothelial cells and the extracellular matrix, they form the tumor microenvironment [1]. When macrophages infiltrate into malignant tumor tissues or are recruited into the tumor microenvironment, they are called tumor-associated macrophages (TAMs) [1]. TAMs are very heterogeneous and reportedly support tumor establishment, progression, angiogenesis, invasion, and immunoregulation [2]. TAMs also express PD-1/PD-L1-signaling programmed cell death protein (PD-1), which is an important molecule in immunosuppression [3]. The PD-1/L1 signaling pathway promotes tumor immune escape by limiting the function of T effector, natural killer (NK), and dendritic cells [4]. Many studies have demonstrated pro-tumor activities of TAMs, although the correlation between TAMs and prognosis has not been investigated for all types of tumors. A high density of TAMs has been significantly associated with negative effects in overall survival in gastric, breast, bladder, ovarian, oral, and thyroid cancers [5]. Interestingly, it is known that the depletion of macrophages by clodronate liposomes revoked tumor progression in an animal model [6,7,8].

Circulating tumor cells (CTCs) are derived from primary and/or metastatic tumors and are used as a real-time minimally invasive liquid biopsy in solid tumors for the clinical assessment of patients [9,10]. CTC studies can guide clinical decisions regarding patient treatment, disease prognosis, response to therapy, early detection of treatment resistance or disease recurrence, tumor progression, and design of new therapeutic approaches [9]. For the clinical use of CTCs as a prognostic and/or predictive biomarker in cancers, a combination of enrichment (isolation), detection (identification), and characterization strategies (such as molecular profiling), is necessary to improve our ability to identify high-risk patients [10].

Melanoma has a high cure potential when diagnosed in early stages, otherwise it can become a metastatic disease, with drastically reduced survival rates [11]. CTCs are detectable in most patients with advanced melanoma [12]. Recent advances in target and immune therapies for metastatic cutaneous melanoma have improved patient survival [13]. However, immunotherapies are highly toxic and effective only in a small proportion of melanoma patients [14,15,16]. In addition, mitogen-activated protein kinases (MAPK) inhibitors, used for most melanoma patients, are also associated with drug resistance [17,18,19].

The effects of the association of CTCs with TAMs are not well understood. It seems that CTC–leucocyte interactions can induce monocyte–macrophage differentiation with recruitment of inflammatory cells, stroma breakdown, and invasion [20]. Interestingly, studies have shown that TAMs, through phagocytosis, can acquire genetic material that may transform them into tumor cells with advanced malignant behavior [21,22,23,24,25]. The incorporation of this extra genetic material could create genomic instability of TAMs that may then play major roles in cancer progression. Zang et al. (2017) demonstrated that the integrated tumor-derived DNA not only increased migratory and proliferative macrophages’ abilities, but also induced the expression of new proteins, such as epithelial markers, and transformed the previous macrophage into stem-like cell [26].

Telomere repeats at chromosomal ends are of critical importance to maintain genomic integrity and have been shown to be effective in assessing genomic instability of cancer cells in several studies [10,27,28]. Particularly, peripheral blood leucocytes’ samples from cutaneous melanoma patients were previously associated with short telomeres and poor survival [29].

In the present pilot study, we isolated CTCs and cancer-associated macrophage-like cells (CAMLs) from melanoma patients’ blood samples. CAMLs are defined as highly differentiated giant phagocytic cells of myeloid lineage (CD14+/CD11c+) presenting large-atypical nuclei or multiple individual nuclei, CD45+, and expressing both cytokeratin and epithelial cell adhesion molecule (EpCAM) [30]. CAMLs are disseminated TAMs which express phagocytic markers, capable of interacting with CTCs in peripheral blood. CAMLs are not found in healthy individuals [31]. Since the purpose of the present study was to compare the 3D telomere parameters of CAMLs, CTCs, and normal leucocytes, only cells with a characteristic distinct morphology (typical CTC morphology—i.e., large and hyperchromatic nucleus and high nucleus–cytoplasmic ratio—and CAML—i.e., large cytoplasm and low nucleus–cytoplasmic ratio) were considered. Clusters (a cluster of two or more CTCs) were excluded from the telomere analysis. To complete the CTCs’ and CAMLs’ characterization, we proceeded to a double immunocytochemistry (ICC) comparing results on the patient’s microporous filter with controls using SK-Mel 28 cultured melanocytes spiked in normal blood.

We used three-dimensional (3D) fluorescent microscopy and quantitative imaging to analyze potential telomeric changes. We evaluated five different telomeric parameters including number of telomere signals, number of telomere aggregates, telomeric signal intensity (telomere length), *a*/*c* ratio, and nuclear volume to identify dysfunctional telomeres. Size-based filtration was used to isolate CTCs, CAMLs, and normal leucocytes. Leucocytes are easy to analyze since they are often found in spaces between the randomly distributed 10^5^ pores on the 8 mm diameter microporous filter; they served as internal patient-specific controls. Our results identified the presence of CAMLs with 3D telomere parameters associated with changes in nuclear architecture, which were distinct from those found in normal l leucocytes of melanoma patients. A high level of telomere dysfunction in melanoma CTCs has been previously described in another study by our group [32].

## 2. Materials and Methods

### 2.1. Patient Samples

Ten peripheral blood samples from nine patients with melanoma stage IV from the Department of Onco-dermatology of Saint-Louis Hospital, Paris, France, were used in this study. They were collected under patients’ written informed consent. The study was carried out in accordance with the Declaration of Helsinki, having the protocol approved by the Institutional Review Board Agreement from US Department of Health and Human Services (n°IRB 00003835, protocol 2015/66NICB). Histopathological and clinical data are shown in the Supplementary Materials, Table S1. The eligibility criteria for patient recruitment were: age ≥ 18 years and diagnosis of metastatic cutaneous melanoma stage IV.

### 2.2. May-Grunwald Giemsa Staining, CTCs and CAMLs Isolation

CTCs and CAMLs enrichment were performed by size-based capture (ScreenCell^®^) using 3 mL of patient’s peripheral blood collected in an EDTA tube [33]. CTC and CAMLs isolation were performed within 3 h of the blood draw. The blood samples were processed through ScreenCell^®^ capture devices (Paris, France), according to the manufacturer’s instructions [33]. Briefly, patient blood was incubated with the ScreenCell Cyto^®^ buffer for 8 min. The blood was then transferred into the top of the filtration unit and vacuum filtered through a microporous filter. After completing the filtration, the filter was stained with standard May Grünwald–Giemsa. The ScreenCell Cyto^®^ buffer lyses the red blood cells and prefixes all nucleated cells present in the blood sample while preserving their nuclear architecture and allows their fixation on the filter of the device. This technique results in an average of 91% CTC recovery rate [33].

### 2.3. Cytologic Evaluation of CTCs and CAMLs

The material was selected according to 2 criteria: first, patient blood had to be sampled less than 6 months earlier in order to be available for the 3D telomere analysis; second, only filters containing both CTCs and CAMLs on the same membrane were selected. Microscopic analysis of the 10 filters was performed by an experienced pathologist (JW). All filters contained CTCs within criteria previously described in the literature [34], i.e., characterized by high nucleus–cytoplasmic ratio (N/C = 0.95–0.75) and hyperchromatic nucleus with a cell diameter larger than 20 µM. Those features allowed the distinction of CTCs from normal leucocytes. CAMLs were much larger than CTCs (up to 300 µM in length), with multiple or lobulated nuclei, as well as larger and elongated cytoplasm. Such morphologic features were consistent with previously described CAMLs [30]. CTC-clusters were defined by groups of 2 or more CTCs [35].

### 2.4. Double Immunocytochemistry (ICC)

Before the immunocytochemistry assay, fixed cells isolated on the filters of the ScreenCell^®^ Cyto device were air-dried overnight at room temperature and then hydrated with tris-buffered saline (TBS; Dakocytomation, Glostrup, Denmark) containing 0.05% Tween 20. The antigens were retrieved with target retrieval solution pH 9 (ER1; Leica Biosystems, Wetzlar, Germany) at 95–99 °C for 20 min and rinsed with Bond Wash (Leica Biosystems). Isolated cells were treated for 5 min at room temperature with a peroxidase block solution (Leica Biosystems). Then, the samples were incubated for 30 min at room temperature with mouse anti-human Melan-A (Dako). A post-primary rabbit anti-mouse was then applied for 8 min followed by a polymer HRP anti-rabbit for an additional 8 min. A sequential incubation step with mouse anti-human CD45 antibody (Leica Biosystems) was applied for 30 min. Finally, a chromogenic staining using DAB-RED detection according to Leica Biosystems protocol and a counter-staining with Hematoxylin (DS9665, Leica Biosystems) for 10 min allowed the revelation of the antigen detection. After a final wash with distilled water, the ScreenCell^®^ Cyto filter was mounted on a glass slide with the Faramount mounting medium (Agilent Technologies, Santa Clara, CA, USA), and covered with an 8 mm diameter coverslip.

### 2.5. Telomere Three-Dimensional Quantitative Fluorescent in Situ Hybridization (3D-QFISH)

For 3D-QFISH [10,26,27], cells on the filters were incubated in 1× PBS for 5 min followed by a 10 min fixation in 3.7% formaldehyde/1× PBS and 3× washes in 1× PBS for 5 min each. Filters were dehydrated in an ethanol series (70%, 90%, and 100%) and air-dried. Fluorochrome-coupled (Cy3) telomere-specific peptide nucleic acid (PNA) probe (DAKO; Agilent Technologies, Santa Clara, CA, USA) was applied (5 μL probe/filter) and, following denaturation at 80 °C for 3 min, hybridization was completed for 2 h at 30 °C. The PNA probe is specific for the telomere repeat sequence ([CCCTAA]_3_–PNA) (DAKO; Agilent Technologies, Santa Clara, CA, USA). The filters were washed in 70% deionized formamide (Sigma-Aldrich, St. Louis, MO, USA) in 10 mM Tris pH 7.4 for 15 min, rinsed in 1× PBS and once each in 2× SSC (5 min at 55 °C), 0.1× SSC and 2× SSC/0.05% Tween-20 at RT. After that, the filters were 4′,6-diamidino-2-phenylindole (DAPI)- stained, mounted with Vectashield (Vector Laboratories, Burlingame, CA, USA) with a coverslip (Fisherbrand; Thermo Fisher Scientific, Waltham, MA, USA).

### 2.6. Imaging & Analysis

All CAMLs, CTCs, and normal leucocytes interphase nuclei found on the filtration device were analyzed using an AxioImager Z2 microscope (Carl Zeiss, Toronto, ON, Canada). An oil objective lens 63×/1.4 (Carl Zeiss Canada Ltd.) was used for image acquisition. A Cy3 filter was used to detect Cy3 probe nuclear hybridization to telomeric repeats at an exposure time of 400 ms for all cells and samples examined. Exposure times for DAPI differed between slides. Forty z-stacks were acquired at a sampling distance of *x*, *y*: 102 nm and *z*: 200 nm for each slice of the stack. Images were deconvolved using the constrained iterative algorithm [36] creating three dimensional nuclear images of the CTCs, CAMLs, and normal leucocytes on the filters. ZEN 2.3 software (Carl Zeiss Canada Ltd.) was used for 3D image acquisition and processing [36]. Deconvolved images were analyzed using the TeloView^®^ v1.03 software program (Telo Genomics Corp., Toronto, ON, Canada) [37]. TeloView^®^ determines 6 telomeric parameters, including telomeric signal intensity (telomere length), number of telomeric signals, number of telomere aggregates, nuclear volume, *a/c* ratio, and nuclear position (relative distance of telomeres to nuclear center/edge) [37]. The *a*/*c* ratio is defined as the nuclear space occupied by telomeres, represented by three axes of length *a*, *b*, and *c*. The ratio between *a* and *c* axes, *a*/*c* ratio, reflects the distribution of telomeres, which changes at different stages of the cell cycle. Lower *a*/*c* ratio numbers are associated with initial stage of the cell cycle, such as G1. On the other hand, high *a*/*c* ratio numbers are found when all telomeres align in the center of the nucleus as cells progress into the late G2 phase. When cells are captured on the ScreenCell filtration device, they are flattened due to the mild vacuum applied during isolation [32]. Therefore, the nuclear volumes and *a*/*c* ratios discussed here can only be seen in a comparative manner (CAMLs vs. leucocytes vs. CTCs) and do not represent absolute measurements.

### 2.7. Statistical Analysis

Telomeric parameters (number, length, telomere aggregates, nuclear volume, *a*/*c* ratio) were compared between different cell types using a randomized block analysis of variance followed by a least-square means multiple comparison. Each patient constitutes a block, and the types of cells are compared within the patients. Graphical presentations indicated the *p*-value for the overall test of differences across the cell types described above taking into consideration both the effect of the patient and the cell type. Chi-square analysis compared the percentage of interphase telomeric signals intensities at defined quartile cut-offs. We considered *p* values ≤ 0.05 to be significant.

## 3. Results

### 3.1. Identification of Circulating Tumor Cells and Circulating Tumor Associated Cells

**Figure 1:**
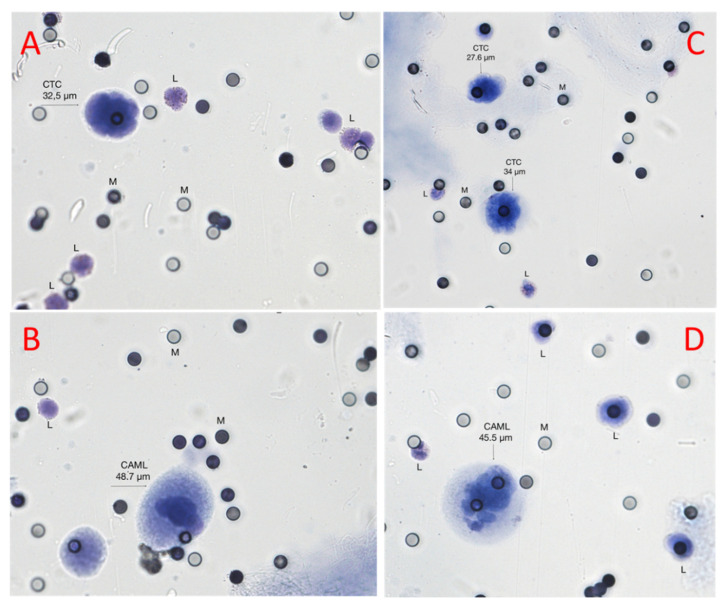
Morphology of the cells identified as CTCs, CAMLs, and normal leucocytes (L) visualized on Screencell^©^ filter colored by May Grunwald–Giemsa, with micropores (M) measuring 6.5 µm in diameter. (**A**) and (**B**) are from patient 9: (**A**) CTC (size = 32.5 microns) characterized by a hyperchromatic nucleus and a high nucleus–cytoplasmic ratio; and (**B**) a typical CAML (size = 48.7 microns) characterized by a low nucleus–cytoplasmic ratio and a normal leucocyte (L) (size = 11.7 microns). C and D are from patient 1: (**C**) 2 CTCs (size = 27.6 microns and 34 microns, respectively) with a large and hyperchromatic nucleus and a high nucleus–cytoplasmic ratio; and (**D**) representative CAMLs characterized by a large and elongated cytoplasm (size = 45.5 microns) and a low nucleus–cytoplasmic ratio.

**Figure 2:**
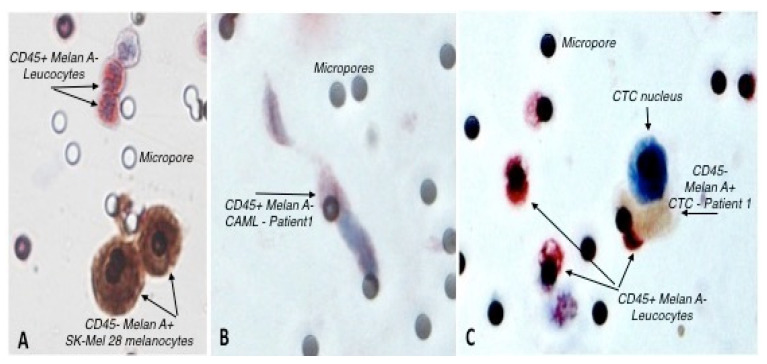
Double ICC. (**A**) Control sample showing CD45+/Melan A− red-stained leucocytes and CD45−/Melan A+ brown-stained SK-Mel 28 cultured melanocytes; (**B**) Microporous filter from patient 1 showing a CD45+/Melan A− CAML with red-stained cytoplasm and blue-stained nucleus; (**C**) Microporous filter from patient 1 showing a CD45−/Melan A+ brown-stained CTC located close to CD45+/Melan A− red-stained leucocytes. G × obj. 40.

A total of 53 blood samples were collected from the 9 metastatic melanoma patients along the 6-month duration of the study. The patients’ venous blood samples were explored for the presence of CTCs three to five times during disease progression. They had three blood samples of 3 mL each time. All blood samples were found to contain CTCs (single CTCs +/− CTC-clusters). Out of the 53 samples, only 10 samples contained both CTCs and CAMLs on the same filter. Thus, 10 blood samples from 9 melanoma patients were processed for CTCs, CTC clusters, and CAMLs evaluation. Table 1 shows the number of CAMLs, CTCs, and CTC-clusters captured on the filter from 3 mL of blood. Blood samples from patient number 1 were analyzed at two different time points (entries 1 and 1.1), T0 and 1 month after. A minimum of 25 normal leucocytes were analyzed per sample. CTCs, CAMLs, and normal leucocytes present in the melanoma patient samples were stained using May Grünwald–Giemsa (Figure 1) and double ICC (Figure 2)

The telomere 3D structure analysis of all the cells in this study showed characteristic morphology of CTCs, CAMLs, and normal leucocytes (Figure 1 and Figure 2), which enabled us to proceed to the genetic profiling in order to explore the telomere cell heterogeneity.

### 3.2. 3D Telomere Profiles Identify CAML Cells with Short Telomeres

**Figure 3:**
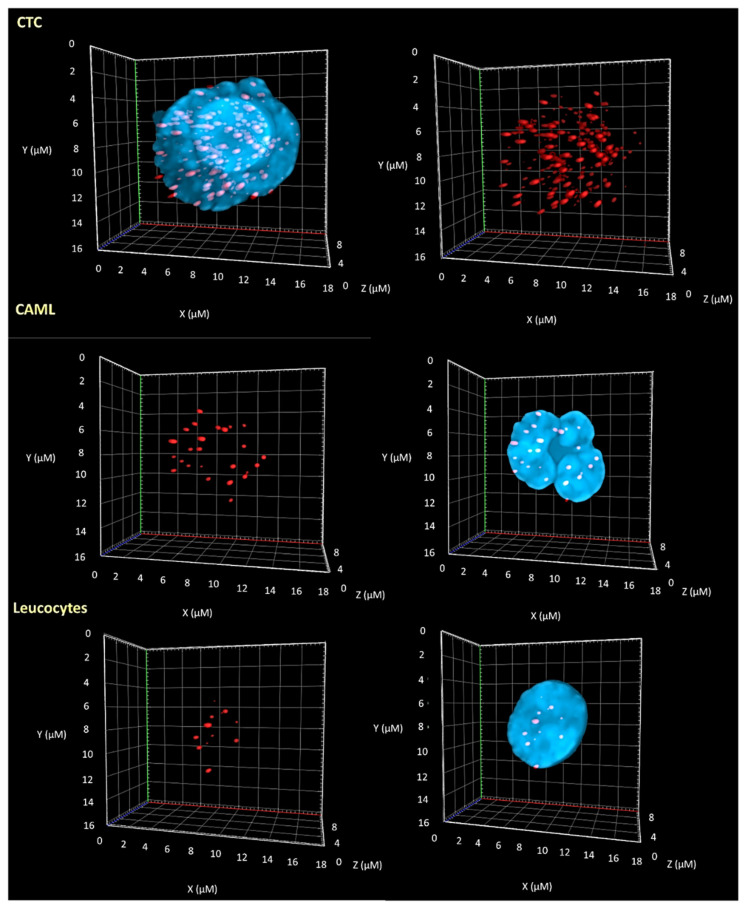
Differences in the 3D nuclear telomeric architecture between CTCs, CAMLs, and leucocytes. Representative nuclei, counterstained with DAPI (blue) from melanoma patient samples (patient 2). The Cy-3 labeled telomeres appear as red signals.

CAMLs can bind and migrate through the blood circulation attached to CTCs in 10% of late-stage cancer patients, pointing to the possible importance of this cell-to-cell interaction [38]. The molecular profiles of CTCs and CAMLs are essential in the comprehensive biological characterization of solid tumors since the interactions between macrophages and tumor cells lead to the development of a pro-tumor phenotype of macrophages (integration of tumor DNA into their genomes) [26]. Genomic instability is a dynamic process where a complex set of genetic alterations creates and propagates clonal diversity [39]. To investigate the levels of genomic instability in CTCs, CAMLs, and normal leucocytes, we analyzed ten melanoma patient samples with 3D-QFISH telomere technology (patient 1 was analyzed at two time points). Representative nuclei were counterstained with DAPI, shown in blue, and the telomeres are visualized as red signals (Figure 3).

**Figure 4:**
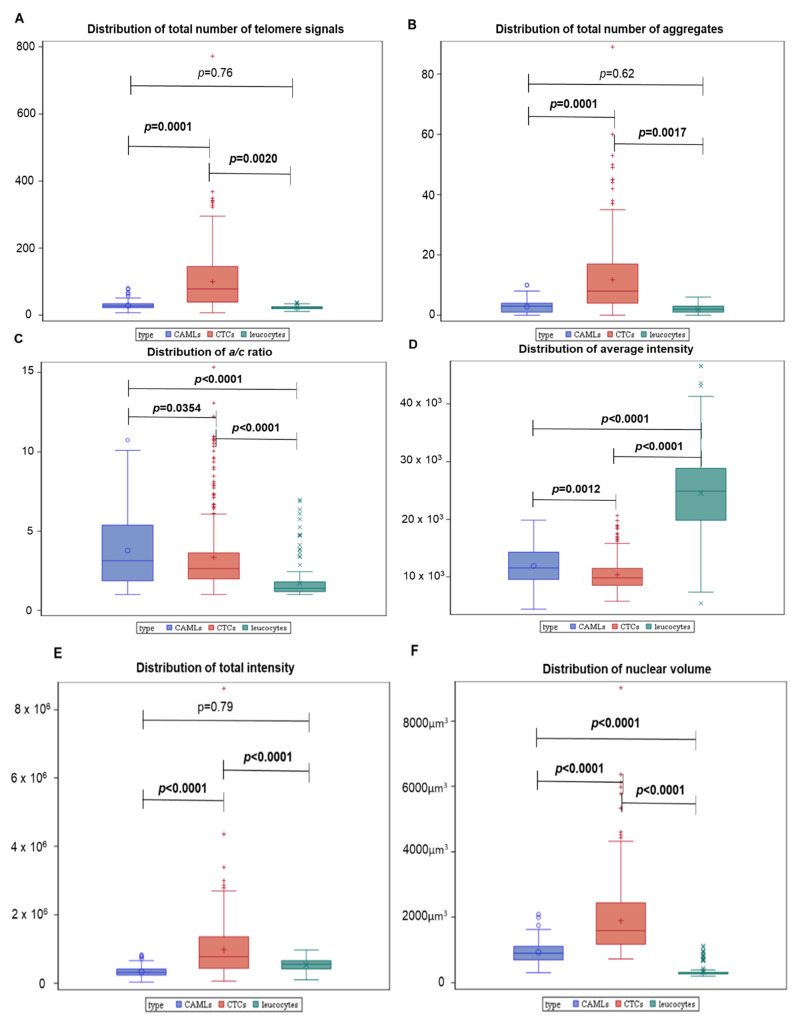
Differences in telomere parameters between CTCs, normal leucocytes, and CAMLs. (**A**) Total number of telomere signals—a sum value representing the number of telomeres found in each cell population. (**B**) Total number of telomere aggregates—telomeres in close proximity forming clusters that cannot be further resolved at an optical resolution limit of 200 nm. Functionally, telomeric aggregates are fused telomeric signals or telomeres in close illegitimate proximity able to engage in recombination events. (**C**) The *a*/*c* ratio (nuclear spatial distribution of telomeres). The a/c ratio is defined as the nuclear space occupied by telomeres, represented by three axes of length *a, b*, and *c*. The ratio between *a* and *c* axes, *a*/*c* ratio, reflects the distribution of telomeres, which changes at different stages of the cell cycle. A higher *a*/*c* ratio indicates cells in G2 and/or G2/M phase, while lower *a*/*c* ratio represents cells in G0/G1 and/or S phase. (**D**) Total telomere signal intensity. (**E**) Average intensity (proportional to telomere length. For total intensity and average intensity, the position of each telomere is identified by using a threshold. Then, the center of gravity and the integrated intensity of each telomere is calculated as arbitrary units based on the number of sequences CCCTAA/probe intensity. The integrated intensity of each telomere is the appropriate parameter for determining the length of the telomere. (**F**) Nuclear Volume. The x-axis assigns one box for each cell population analyzed (results of all analyzed cells). The y-axis refers to the lower value, first quartile, average (mean), median, and third quartile.

The first step was to compare all five TeloView^®^ parameters (total number of telomere signals, total number of telomere aggregates, *a*/*c* ratio, telomeric signal intensity (telomere length—total and average intensity), and nuclear volume) among the three cell types. We found significant differences between CTCs vs. normal leucocytes and CTC vs. macrophages among all five TeloView^®^ parameters (Figure 4). However, just three parameters were significantly different between CAMLs and normal leucocytes (*a*/*c* ratio, average intensity (proportional to telomere length) and nuclear volume). File S1 (Supplementary Materials) shows the individual comparisons for all patients. The *p*-values, included in each graph, represent the significance of the difference in telomere architecture (TeloView^®^ parameters) among cell types.

**Figure 5:**
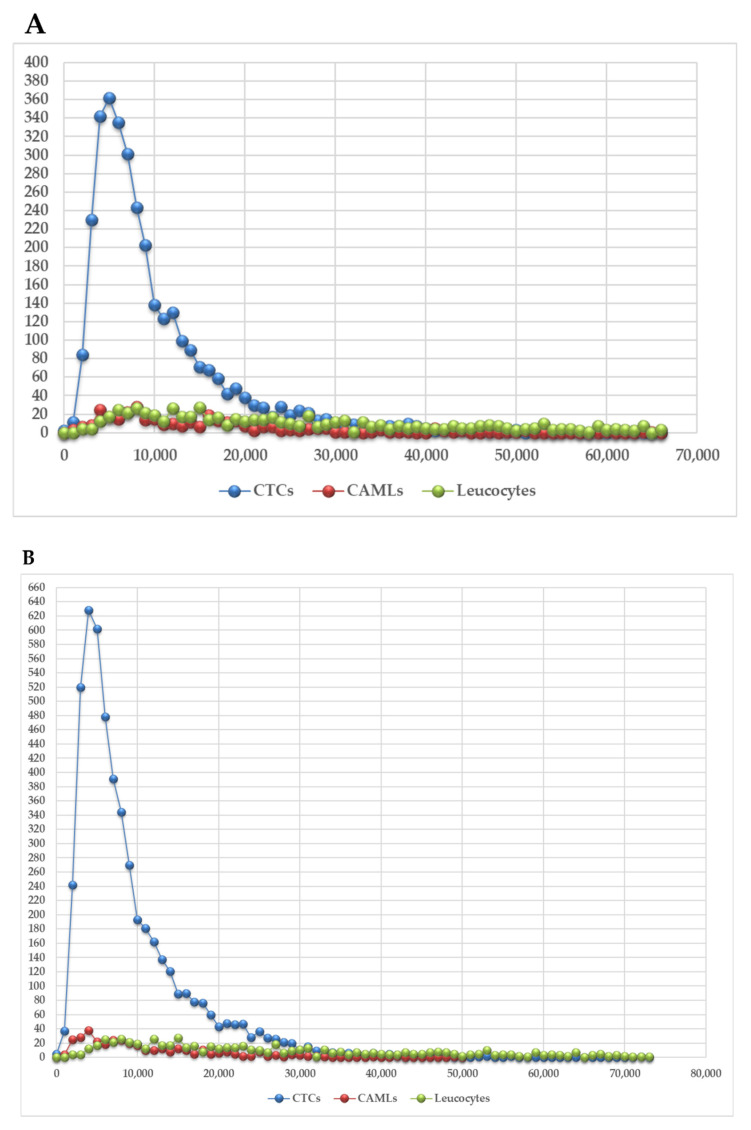

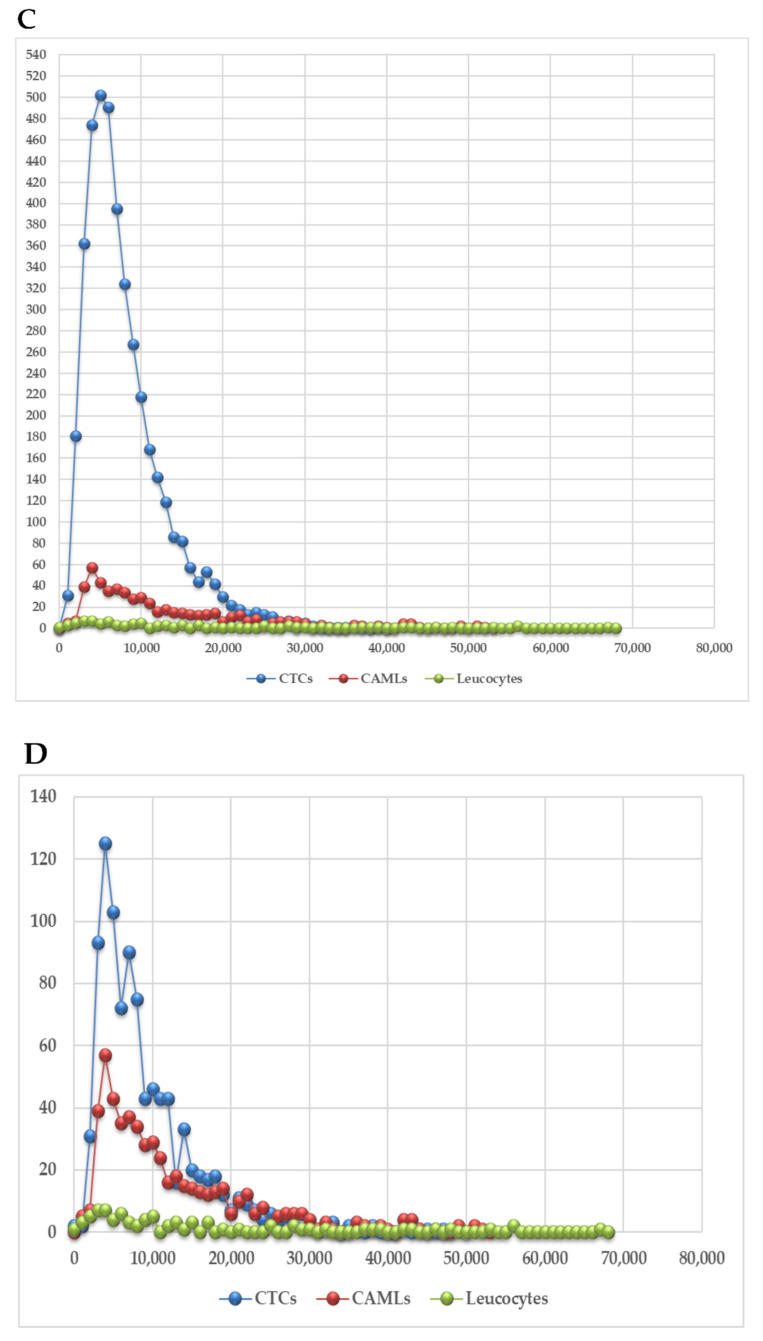
Representative examples of the CTCs, CAMLs, and normal leucocytes 3D telomere profile for patients assigned to profile 1 (**A**,**B**) and profile 2 (**C**,**D**). The X-axis shows the number of telomeres and the y-axis represents the telomere length in arbitrary units of fluorescence (a.u). CTCs, CAMLs, and leucocytes are demarked with colors. The graphs correspond to patient 7 (**A**), patient 3 (**B**), patient 1 (**C**), and patient 2 (**D**).

The second step was to investigate further the significant differences observed in average intensity between CAMLs and leucocytes. Therefore, we graphically demonstrated the relationship between telomere length and the number of telomeres. The total number of telomere signals corresponds to the number of telomeres detected in the 3D preserved nucleus of the cell. This combination differentiated two distinct profiles within the CAMLs population (profile 1 and profile 2—Table 2). In the first one, the CAML telomere and leucocytes’ profiles were similar (Figure 5A,B—profile 1) with a very low number of short telomeres (telomeres with low intensity signals). In the second one, the levels of genomic instability found in CAMLs were higher in comparison with leucocytes (Figure 5C,D—profile 2). Profile 2 was characterized by an accumulation of telomeres with low signal intensities (short telomeres).

In Figure 5, the telomere length (signal intensity, x-axis) is plotted against the number of telomeres (y-axis) for all analyzed cells. Signals are grouped by their intensity level, and this gives a picture of the telomere distribution in each sample. For normal leucocytes, for example, this plot usually has small peaks between 0 and 20,000 a.u (arbitrary units of relative fluorescence intensity), in which the number of telomeres per nucleus on the y-axis range between 5 and 25 [10]. For leucocytes, most of the telomere signals have high relative intensities, with signals detected up to 120,000 a.u [10]. CTCs are known to have a high peak of shorter telomeres, and this finding was similarly described in other CTCs studies [10,32,39]. Additionally, we found that four/five melanoma patients had CAMLs with high levels of genomic instability (signal intensity vs. number of telomere signals) in comparison with parental leucocytes (Figure S1, Supplementary Materials). Samples from patient 1 were analyzed at two time points (T0 and T + 1 month). Interestingly, patient 1 presented the same profile at the two time points analyzed (Profile 2), which is characterized by a higher number of short telomeres in the CAMLs population in comparison with leucocytes. Telomere length vs. number of telomeres for all patients are shown in Figure S1 (Supplementary Materials).

## 4. Discussion

Although studies have focused on enumeration and molecular characterization of CTCs, only CAMLs’ enumeration platforms are being used in clinical studies [31]. Adams et al. (2016) showed that CAMLs could be differentiated in malignant disease and benign breast lesions emphasizing that TAMs are also an important cell type to be characterized at the molecular level [38]. TAMs can migrate with CTCs within the blood circulation, and the CTC–CAML interaction is correlated with worse prognosis [31,35]. CAMLs could be considered as disseminated TAMs which express phagocytic markers, capable of interacting with CTCs in peripheral blood. TAMs were reported to be found in the tissue microenvironment. More recently, similar cells were described in the blood as CAMLs. Briefly, TAMs are macrophages present in tissue close to the tumor, CAMLs could be regarded as the equivalent cells in the blood, but their origin is still unclear. CAMLs are not found in healthy individuals [30]. CAMLs may be indistinguishable from CTCs or normal leucocytes by immunophenotyping, since CAMLs may be also CK and CD45 positive cells. In our study, CAMLs expressed the CD45 antigen but not the Melan-A antigen. In addition, TAMs can incorporate DNA from apoptotic bodies. The incorporation of DNA from cancer cells could lead to genomic instability in the CAMLs’ population. Passerini et al. (2016) demonstrated that the addition of even a single chromosome to human cells promotes genomic instability by increasing DNA damage and sensitivity to replication stress [40]. However, colony stimulating factor 1 (CSF-1), C-C motif ligand 2 (CCL2), vascular endothelial growth factor A (VEGF-A), and the estimated glomerular filtration rate (EGFR) signaling pathway are the most well-documented macrophage stimulating protumor phenotype [41]. CSF-1 depletion reduces macrophage density, delays tumor progression, and inhibits metastasis. CCL2 was reported to shape macrophage polarization toward the protumor phenotype via the C-C chemokine receptor 2 (CCR2) expressed on the surface of macrophages [41]. In addition, VEGF-A promotes growth of tumors by inducing TAM infiltration. EGFR indirectly adjusts the tumor microenvironment by regulating macrophage recruitment. Disrupted EGFR signaling is correlated with better prognosis in colon cancer models [41].

In our current study, we used a size-based filtration device to isolate CAMLs from blood samples of melanoma patients (Figure 1 and Figure 2). Together, our results demonstrate that CTCs and CAMLs are efficiently captured and enriched by this method to be used in cytological analysis. A CAML cell population was detected in all patients. Other studies had used FDA-approved CellSearch™ system to isolate CAMLs [30]. The CellSearch^®^ platform is an EpCAM-based capture assay based on the expression of epithelial cell adhesion molecule (EpCAM). However, low or lack of EpCAM expression in CAMLs can results in an underestimation of the number of CAMLs [35]. Our study showed that three telomere parameters were able to differentiate normal leucocytes and CAMLs (Figure 3). As expected, CTCs have a higher nuclear volume in comparison with CAMLs and leucocytes. Interestingly, CAMLs have the highest *a*/*c* ratio among the other cell types. A higher *a*/*c* ratio indicates cells in G2 and/or G2/M phase. Telomere length was also significantly different between cell types. CAMLs have shorter telomeres in comparison with leucocytes, but they are not as shorten as the telomeres found in CTCs (Figure 4). In addition, we combined number of telomeres vs. telomere length and identified the presence of two CAMLs subpopulations with different telomere profiles (Figure 5). Profile 2 showed a high peak of short telomeres (in CAMLs) in comparison with control leucocytes (from the same patient) and profile 1 showed a telomere length similar to control leucocytes.

The profile 2 was observed in almost half of the melanoma patients. These results demonstrate the presence of two CAMLs subpopulations with low and high numbers of short telomeres. There were no differences in CAMLs’ morphology between the two profiles.

It is important to note that the cells retained by the microporous filter have a flatter morphology than the cells placed on a two-dimensional surface such as in a regular microscope slide. This is due to the mild vacuum that is applied when the cells are captured onto the device. This factor influences size measurements and, consequently, the TeloView^®^ parameters of nuclear volume and *a*/*c* ratio are also influenced. However, since all cell types were subjected to the same enrichment technology, we assumed an even impact of the mild vacuum applied during the cell isolation across all cell types, which warranted both nuclear volume and *a*/*c* ratio appropriate as comparative elements between cell types. Vacuum cell aspiration through the micropores can modify the cell-to-cell interaction. For this reason, cell-to-cell interaction between CTCs and CAMLs is absent.

Interestingly, in the single patient where we compared the 3D telomere profile at T0 and one month after (patient 1 in Figure S1, Supplementary Materials), numbers of shorter telomeres in the CAML population were unchanged at the different time points. However, only patient 1 was analyzed at two time points. Even though the peak of short telomeres decreased slightly in CTCs between the two time points, the CAMLs did not change. Further validation studies, including a larger patient sample size, are necessary to confirm these findings in order to explore the possible prognostic value of the profile number 2. Our data provide evidence that increased levels of telomere dysfunction can be found in CAMLs for some of the melanoma patients. Interactions between CTCs and CAMLs could be responsible for the highly heterogeneous phenotypes found in circulating CAMLs. Other observations from the literature showed evidence for the spontaneous fusion of tumor cells with macrophages producing circulating hybrid cells [42]. Thus, it is possible to suggest that those hybrid cells might be a part of the Profile 2 CAMLs subpopulation with a high peak of short telomeres. However, the nature of the CTCs–CAMLs interaction remains unclear, as well as whether this association has a prognostic value.

We acknowledge that the main limitation of the present study is the small sample size, which could interfere with a more substantiated conclusion. Our intention in putting forward these relevant preliminary observations concerning the differences in the 3D telomere parameters between CAMLs, leucocytes, and CTCs (from the same patient) was to bring light to this individualized approach. Another point of consideration is that all patient samples used in this study were after treatment (no pretreatment samples available), which prevented a broader discussion. Considering that it is unknown what is creating genomic instability in the CAMLs population, if the treatment were to be accounted responsible for that, we would have seen an increased genomic instability in all patients. Although the impact of treatment cannot be excluded, we did not observe that. For example, patients 4 and 9 received the same treatment and they fell into different profiles (profiles 2 and 1, respectively), suggesting no association with the treatment received.

In summary, we demonstrated, for the first time, the different levels of telomere related genomic instability on CAMLs compared to control leucocytes and CTCs from melanoma patients. This study provided insights into the importance of molecular characterization of both CTCs and CAMLs as a promising, minimally invasive approach to evaluate the nature of the CTCs–CAMLs interactions. This may, in the future, allow for an assessment of their possible combined prognostic impact.

## Figures and Tables

**Table 1:** Results of enumeration of CAMLs, CTCs, and clusters in 10 melanoma blood samples.

Patient Number	Reference of Sample	Blood Sample Volume	CAML Nb	CTC Nb	CTC-Cluster Nb
1	VS-1-018 (ET3) 16AB0304	3 ml	2	56	0
1.1	VS-1-018_M1-3-17AA1313	3 ml	7	62	4
2	TM-1-019_M1- 16AB0258	3 ml	2	11	0
3	LM-1-039_CL2-17AA0828	3 ml	1	33	0
4	BJ-1-071_ET4-16AA8528	3 ml	1	22	0
5	DA-1-075_ET2-16AA7280	3 ml	3	26	0
6	GF-1-080_ET1-16AA7266	3 ml	2	32	30
7	LM-1-081_ET1-16AA7320	3 ml	2	20	2
8	AR-1-092_ET1-16AA2042	3 ml	2	19	12
9	KJ-1-084_ET1-17AA0845	3 ml	2	12	0

CTCs, circulating tumor cells; CTC-clusters (clusters of 2 or more CTCs), circulating tumor cell clusters; CAMLs, cancer-associated macrophage-like cells; Nb, number.

**Table 2:** List of all patients with their corresponding telomere profile groups.

Patient Number	Reference of Sample	Telomere Profile
1	VS-1-018 (ET3) 16AB0304	Profile 2
1.1	VS-1-018_M1-3-17AA1313	Profile 2
2	TM-1-019_M1- 16AB0258	Profile 2
3	LM-1-039_CL2-17AA0828	Profile 1
4	BJ-1-071_ET4-16AA8528	Profile 2
5	DA-1-075_ET2-16AA7280	Profile 2
6	GF-1-080_ET1-16AA7266	Profile 1
7	LM-1-081_ET1-16AA7320	Profile 1
8	AR-1-092_ET1-16AA2042	Profile 1
9	KJ-1-084_ET1-17AA0845	Profile 1

## Data Availability

Not applicable.

## Supplementary Materials

The following supporting information can be downloaded at: https://www.mdpi.com/article/10.3390/biomedicines10102391/s1, Table S1: Clinical information of all patients included in the study; Figure S1: Representative figures of the CTCs, CAMLs, and leucocytes profiles for all patients assigned to profile 1 (Patients 3, 6, 7, 8, and 9) and profile 2 (Patients 1 and 1.1, 2, 4, and 5). In each graph, the telomere length is shown in arbitrary units of fluorescence (AU). The CTCs, CAMLs (macrophages) and leucocytes are demarked with colors. The telomere results for each patient are shown in File S1.
