# Supplementary material for: Telomere-Associated Changes in Nuclear Architecture of Cancer-Associated Macrophage-like Cells in Liquid Biopsies from Melanoma Patients
Source: Biomedicines. 2022 Sep 25;10(10):2391. doi: 10.3390/biomedicines10102391 (PMC9598704; doi:10.3390/biomedicines10102391)
Supplement: Supplementary file 1 [file biomedicines-10-02391-s001.zip › File S1 Telomere data for all patients.pdf]

The GLM Procedure

pt=16AA2042

Class Level Information		
**Class**	**Levels**	**Values**
**type**	3	CTCs Lymp Macr

**Number of Observations Read**	63
**Number of Observations Used**	63

The GLM Procedure

Dependent Variable: Totalnofsignals
Totalnofsignals

pt=16AA2042

Source	DF	Sum of Squares	Mean Square	F Value	Pr > F
**Model**	2	21976.04339	10988.02169	10.60	0.0001
**Error**	60	62171.60741	1036.19346		
**Corrected Total**	62	84147.65079			

R-Square	Coeff Var	Root MSE	Totalnofsignals Mean
0.261161	79.84124	32.18996	40.31746

Source	DF	Type III SS	Mean Square	F Value	Pr > F
**type**	2	21976.04339	10988.02169	10.60	0.0001

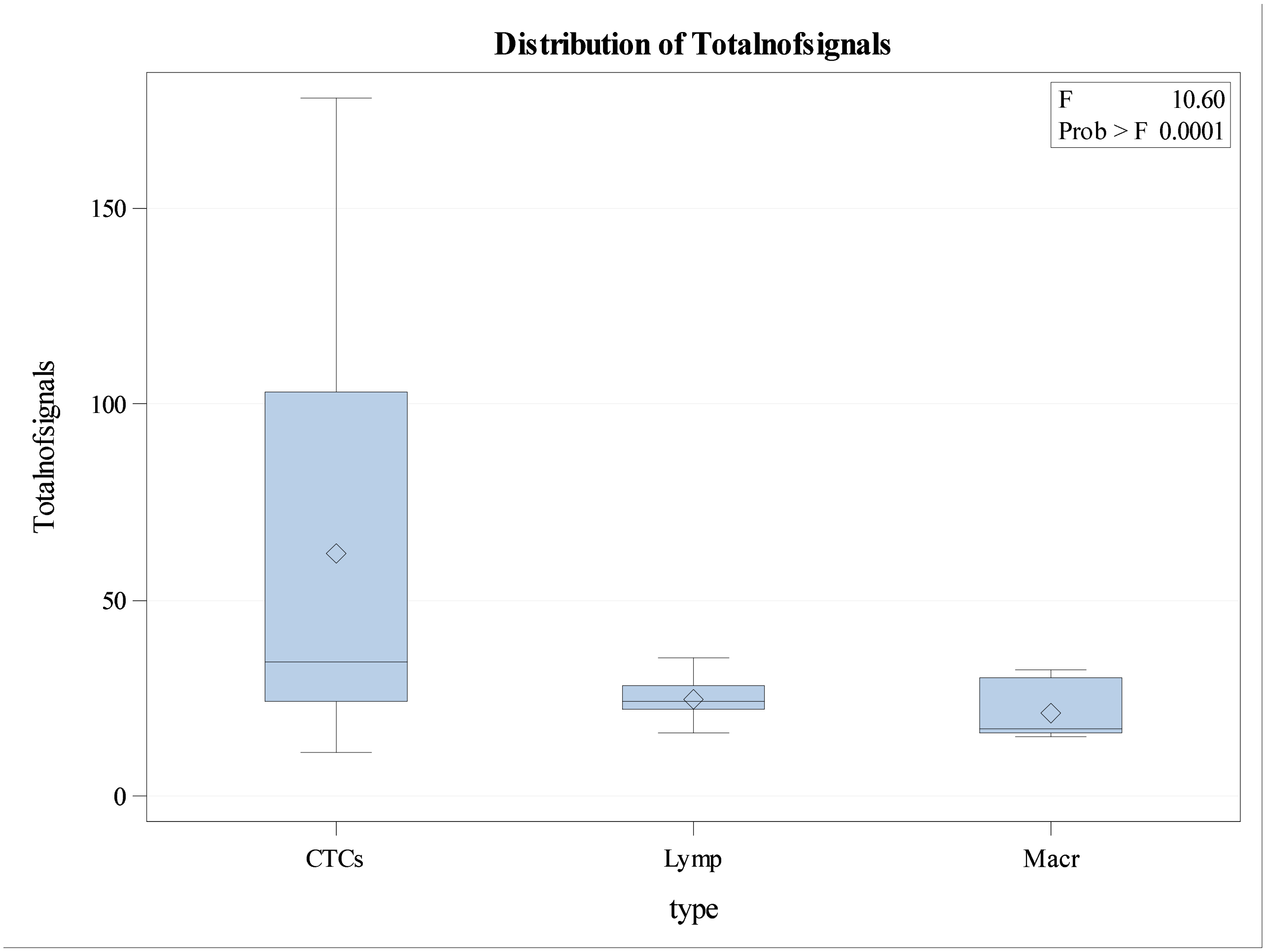

The GLM Procedure

Dependent Variable: Totalnofaggregates Totalnofaggregates

pt=16AA2042

Source	DF	Sum of Squares	Mean Square	F Value	Pr > F
Model	2	321.765608	160.882804	6.98	0.0019
Error	60	1383.218519	23.053642		
Corrected Total	62	1704.984127			

R-Square	Coeff Var	Root MSE	Totalnofaggregates Mean
0.188721	120.5137	4.801421	3.984127

Source	DF	Type III SS	Mean Square	F Value	Pr > F
type	2	321.7656085	160.8828042	6.98	0.0019

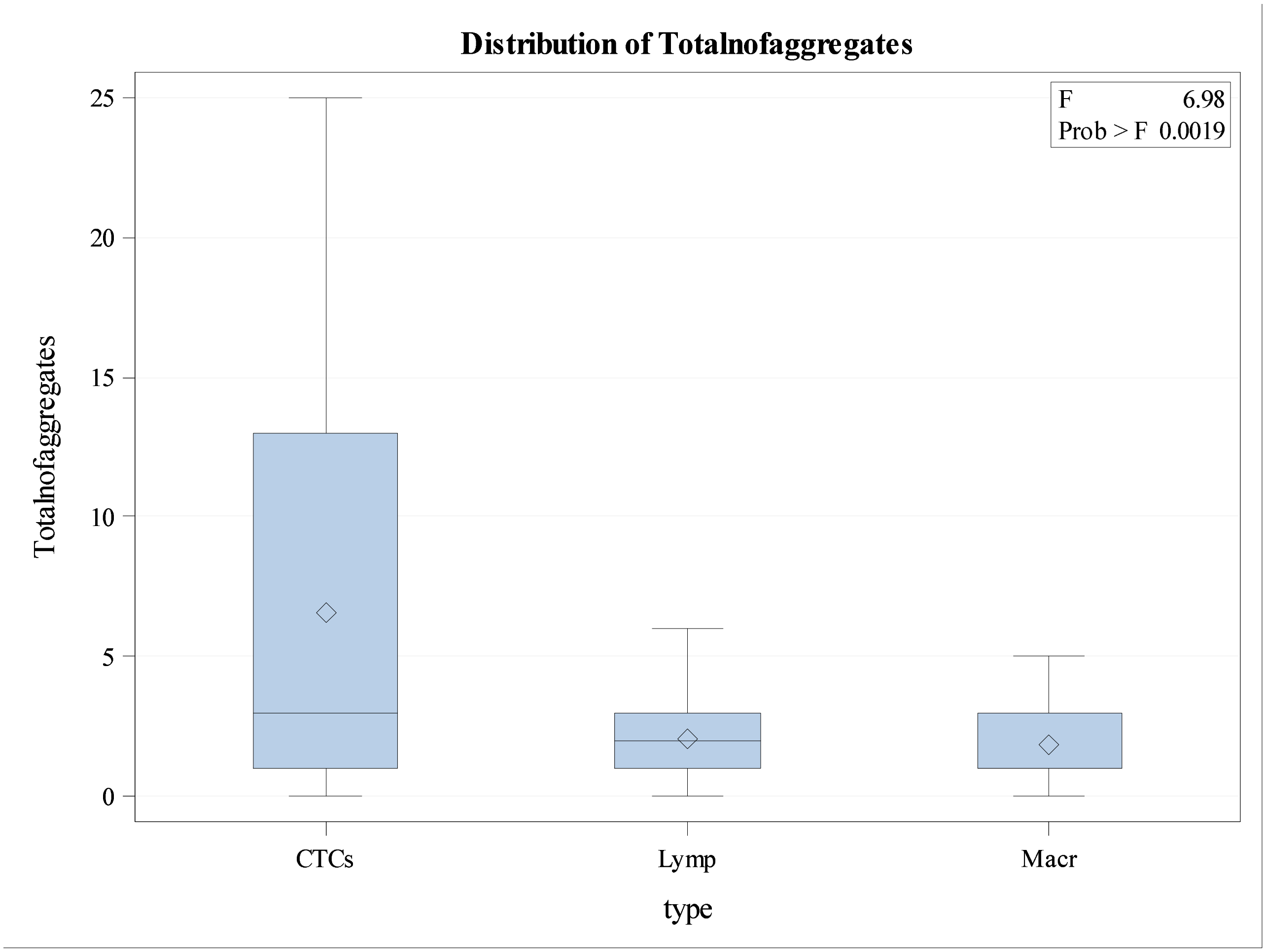

The GLM Procedure

Dependent Variable: acratio
acratio

pt=16AA2042

Source	DF	Sum of Squares	Mean Square	F Value	Pr > F
**Model**	2	26.3361139	13.1680569	3.67	0.0314
**Error**	60	215.3564603	3.5892743		
**Corrected Total**	62	241.6925742			

R-Square	Coeff Var	Root MSE	acratio Mean
0.108965	95.61216	1.894538	1.981482

Source	DF	Type III SS	Mean Square	F Value	Pr > F
**type**	2	26.33611385	13.16805693	3.67	0.0314

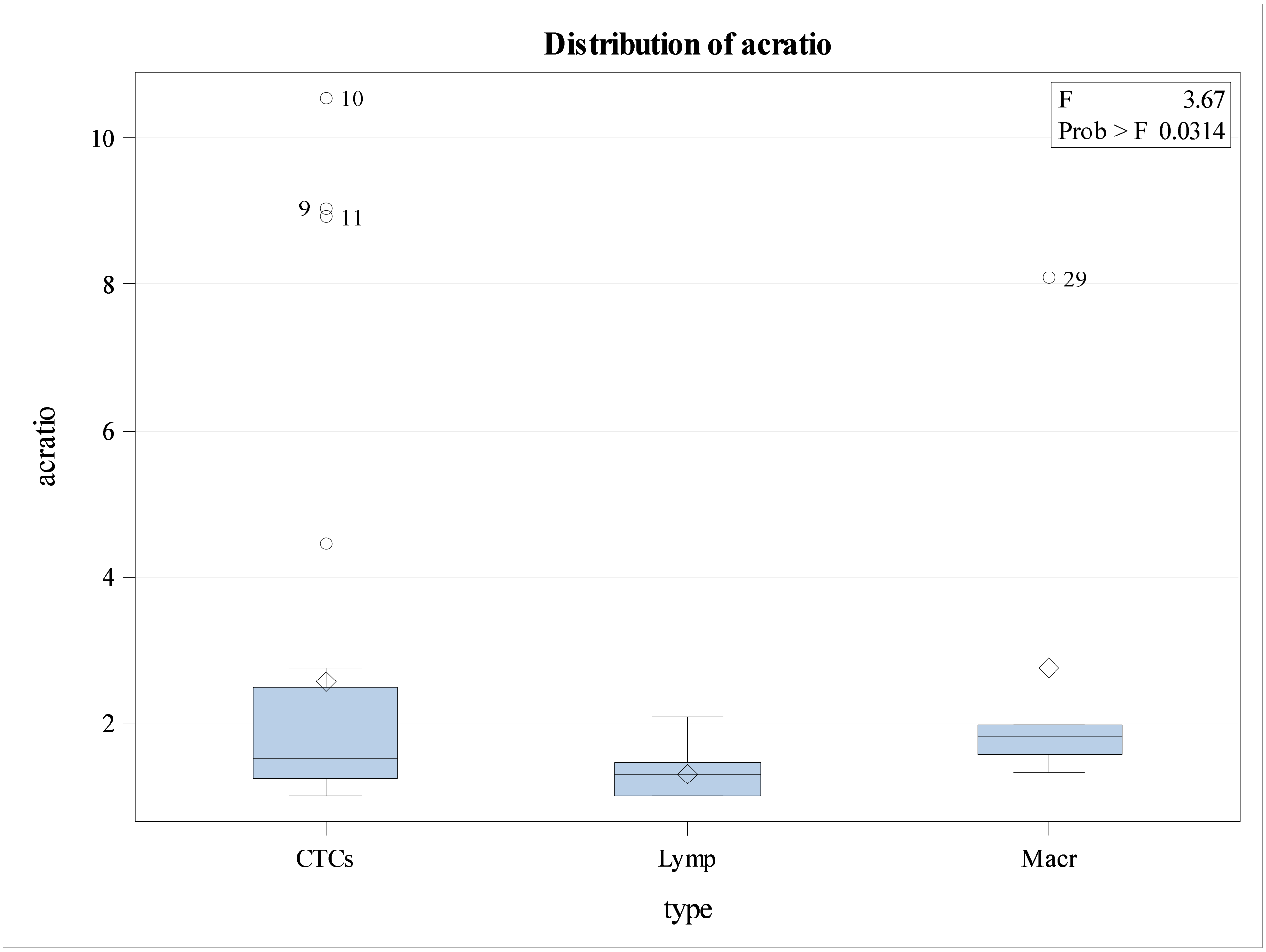

The GLM Procedure

Dependent Variable: AvIntallsignals AvIntallsignals

pt=16AA2042

Source	DF	Sum of Squares	Mean Square	F Value	Pr > F
**Model**	2	3008134963	1504067482	75.85	<.0001
**Error**	60	1189717370	19828623		
**Corrected Total**	62	4197852333			

R-Square	Coeff Var	Root MSE	AvIntallsignals Mean
0.716589	24.05866	4452.934	18508.65

Source	DF	Type III SS	Mean Square	F Value	Pr > F
**type**	2	3008134963	1504067482	75.85	<.0001

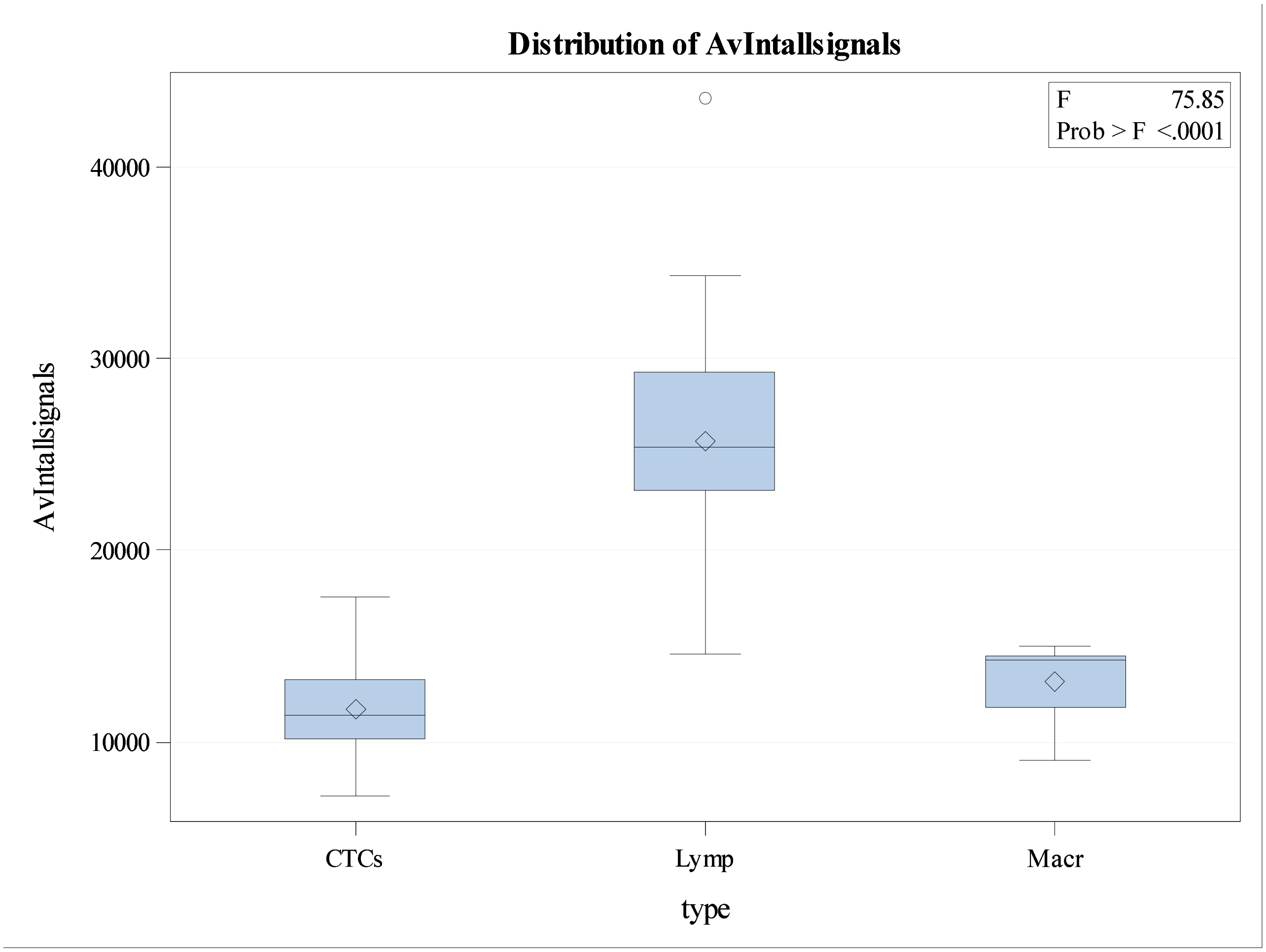

The GLM Procedure

Dependent Variable: Totalintensity Totalintensity

pt=16AA2042

Source	DF	Sum of Squares	Mean Square	F Value	Pr > F
Model	2	798667878172	399333939086	4.15	0.0206
Error	60	5.7771163E12	96285271503		
Corrected Total	62	6.5757842E12			

R-Square	Coeff Var	Root MSE	Totalintensity Mean
0.121456	50.98279	310298.7	608634.1

Source	DF	Type III SS	Mean Square	F Value	Pr > F
type	2	798667878172	399333939086	4.15	0.0206

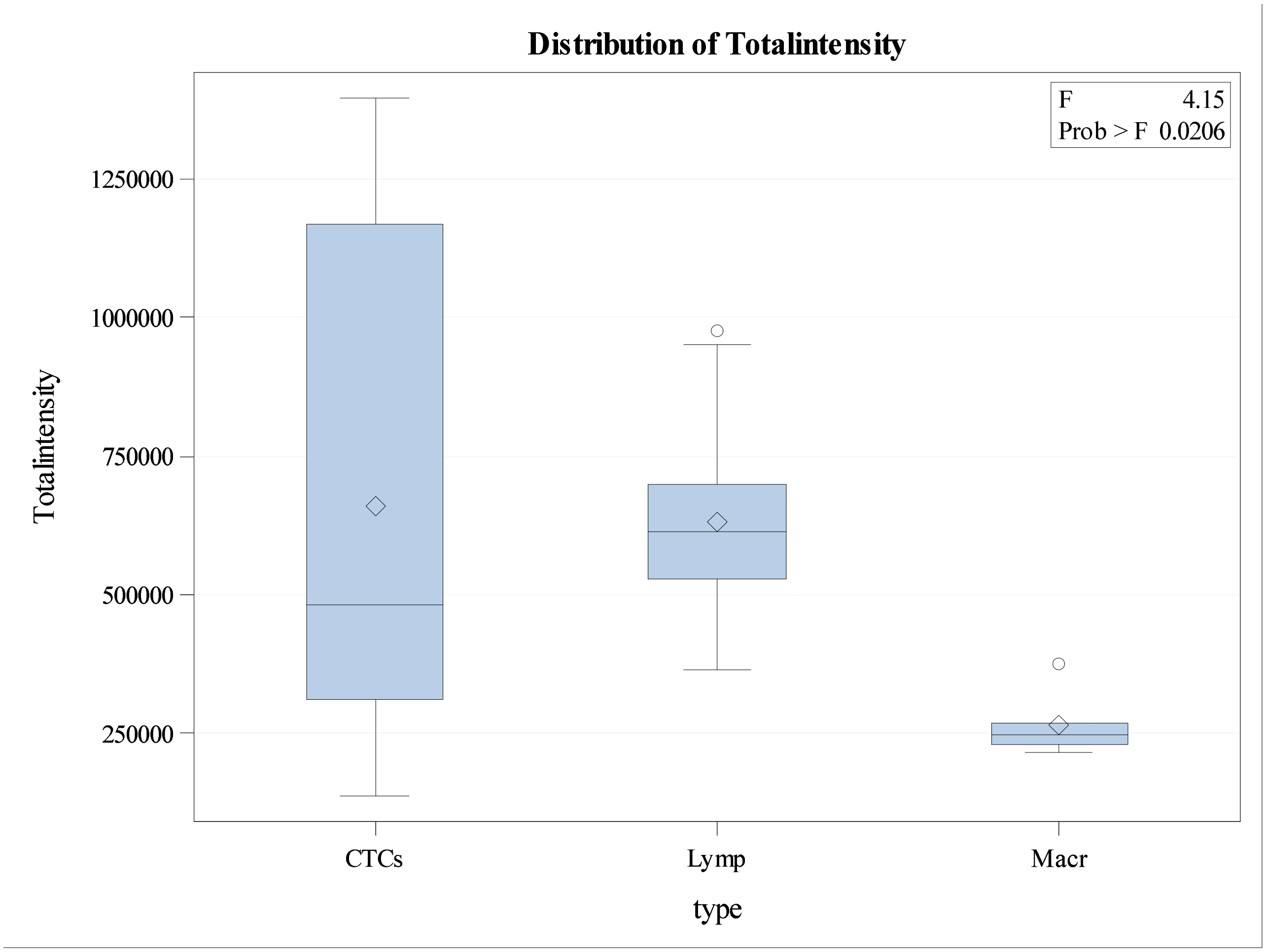

The GLM Procedure

Dependent Variable: Nuclearvolume Nuclearvolume

pt=16AA2042

Source	DF	Sum of Squares	Mean Square	F Value	Pr > F
**Model**	2	6.7641002E12	3.3820501E12	28.45	<.0001
**Error**	60	7.1321231E12	118868718736		
**Corrected Total**	62	1.3896223E13			

R-Square	Coeff Var	Root MSE	Nuclearvolume Mean
0.486758	69.98856	344773.4	492614.0

Source	DF	Type III SS	Mean Square	F Value	Pr > F
**type**	2	6.7641002E12	3.3820501E12	28.45	<.0001

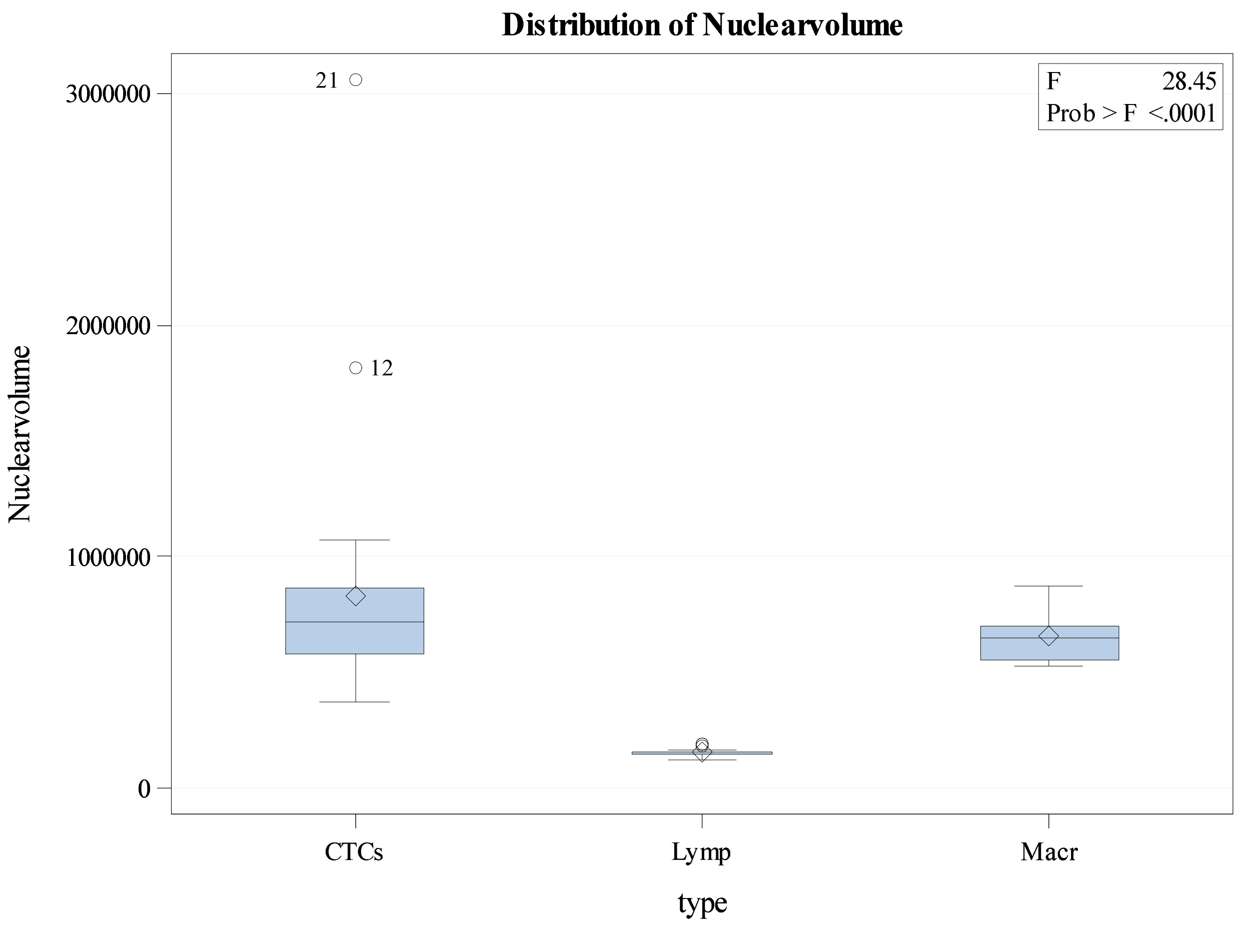

The GLM Procedure

Dependent Variable: telomereperkvol

pt=16AA2042

Source	DF	Sum of Squares	Mean Square	F Value	Pr > F
**Model**	2	0.14677950	0.07338975	46.15	<.0001
**Error**	60	0.09541443	0.00159024		
**Corrected Total**	62	0.24219393			

R-Square	Coeff Var	Root MSE	telomereperkvol Mean
0.606041	35.16967	0.039878	0.113387

Source	DF	Type III SS	Mean Square	F Value	Pr > F
**type**	2	0.14677950	0.07338975	46.15	<.0001

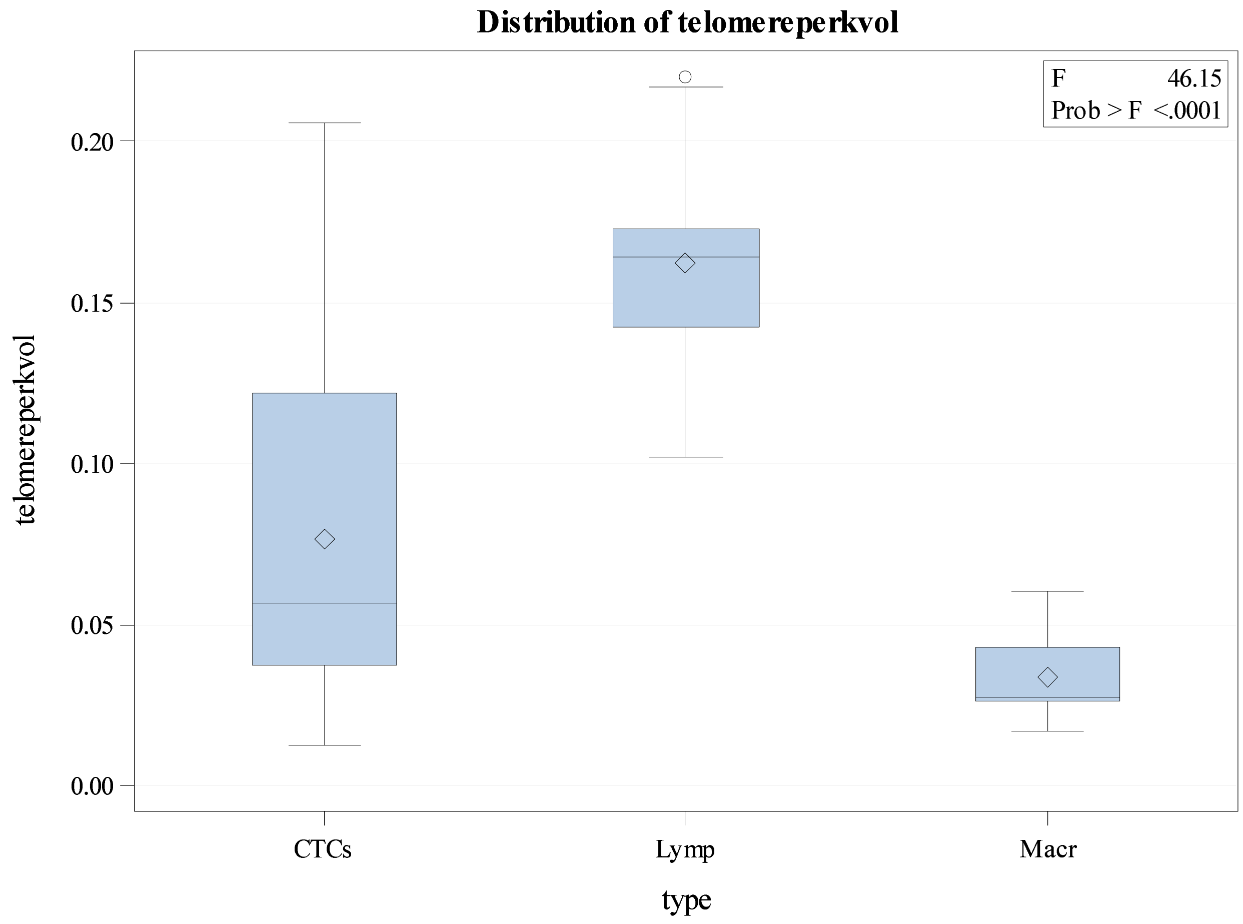

The GLM Procedure
Least Squares Means

pt=16AA2042

type	Totalnofsignals LSMEAN	Standard Error	Pr > |t|	LSMEAN Number
**CTCs**	61.8518519	6.1949605	<.0001	1
**Lymp**	24.7666667	5.8770555	<.0001	2
**Macr**	21.1666667	13.1414957	0.1125	3

Least Squares Means for effect type
Pr > |t| for H0: LSMean(i)=LSMean(j)

Dependent Variable: Totalnofsignals

i/j	1	2	3
**1**		<.0001	0.0069
**2**	<.0001		0.8034
**3**	0.0069	0.8034	

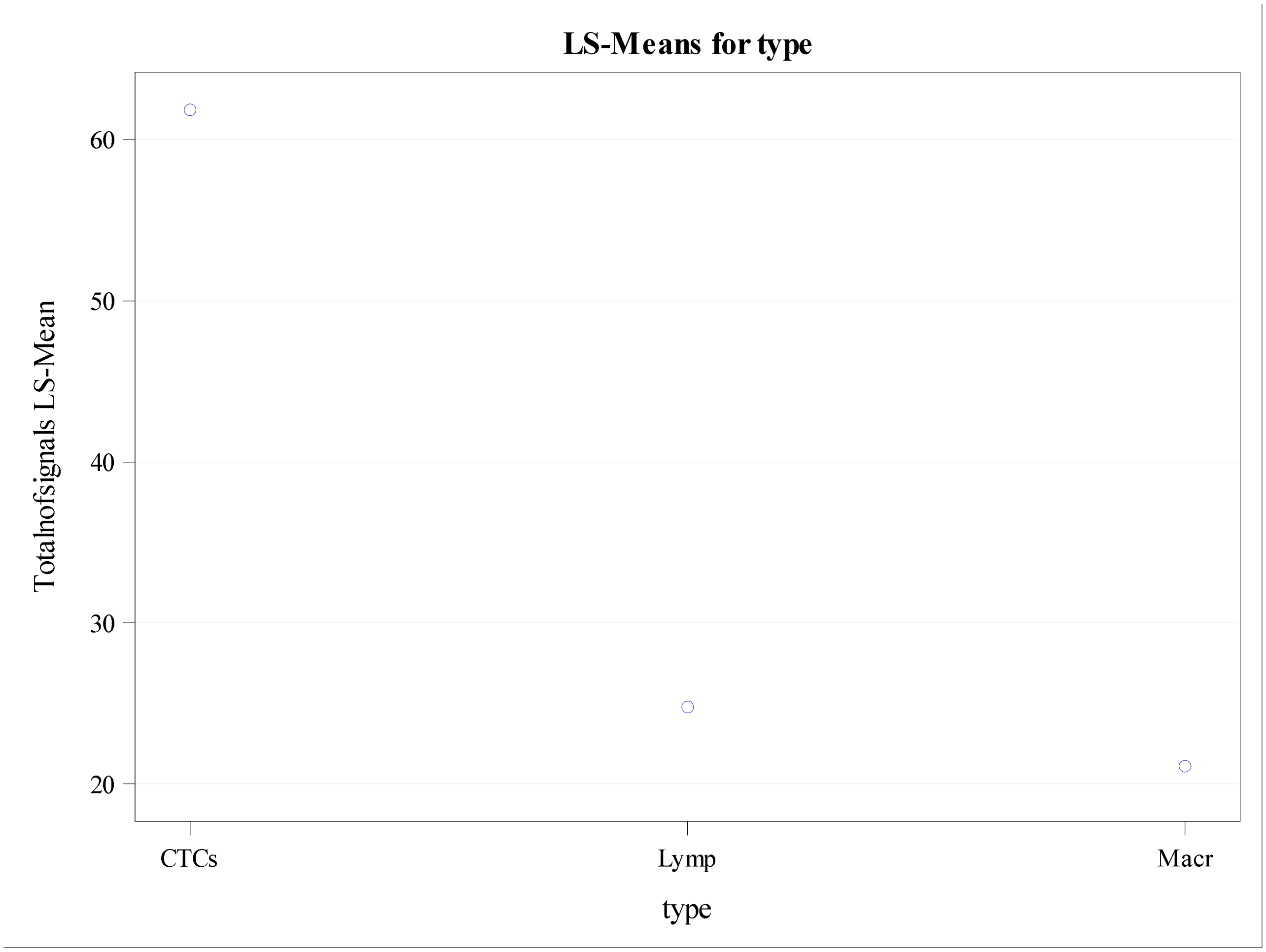

The GLM Procedure
Least Squares Means

pt=16AA2042

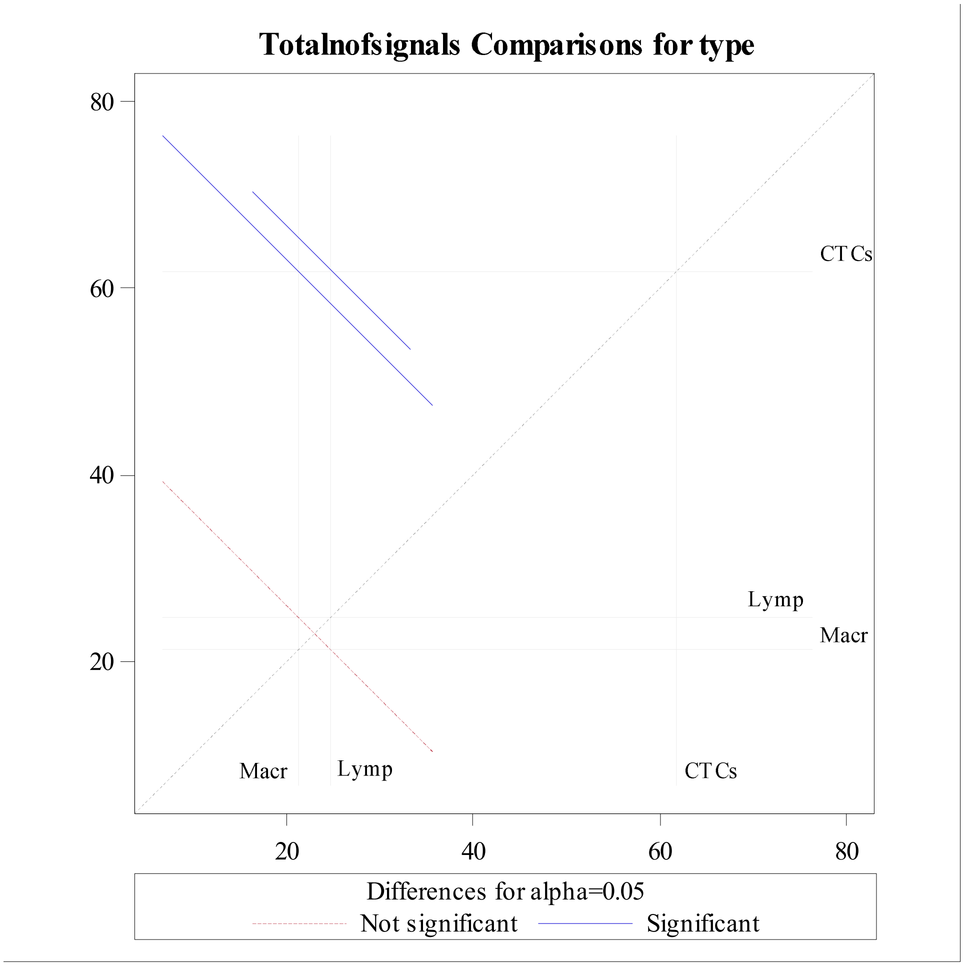

type	Totalnofaggregates LSMEAN	Standard Error	Pr > |t|	LSMEAN Number
**CTCs**	6.59259259	0.92403387	<.0001	1
**Lymp**	2.06666667	0.87661550	0.0217	2
**Macr**	1.83333333	1.96017185	0.3534	3

The GLM Procedure
Least Squares Means

pt=16AA2042

Least Squares Means for effect type<br>Pr > |t| for H0: LSMean(i)=LSMean(j)<br><br>Dependent Variable: Totalnofaggregates			
**i/j**	**1**	**2**	**3**
**1**		0.0007	0.0320
**2**	0.0007		0.9138
**3**	0.0320	0.9138	

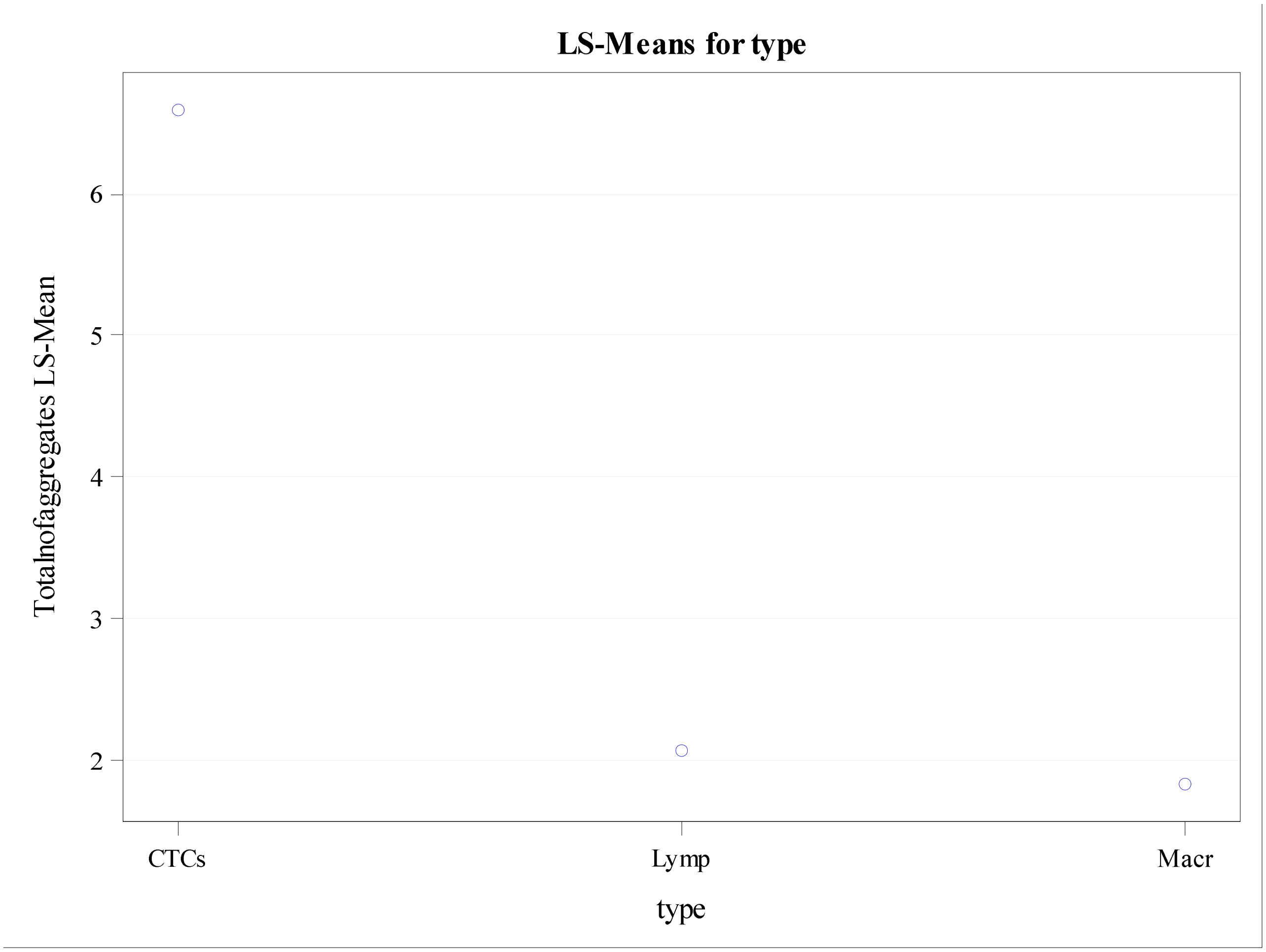

The GLM Procedure
Least Squares Means

pt=16AA2042

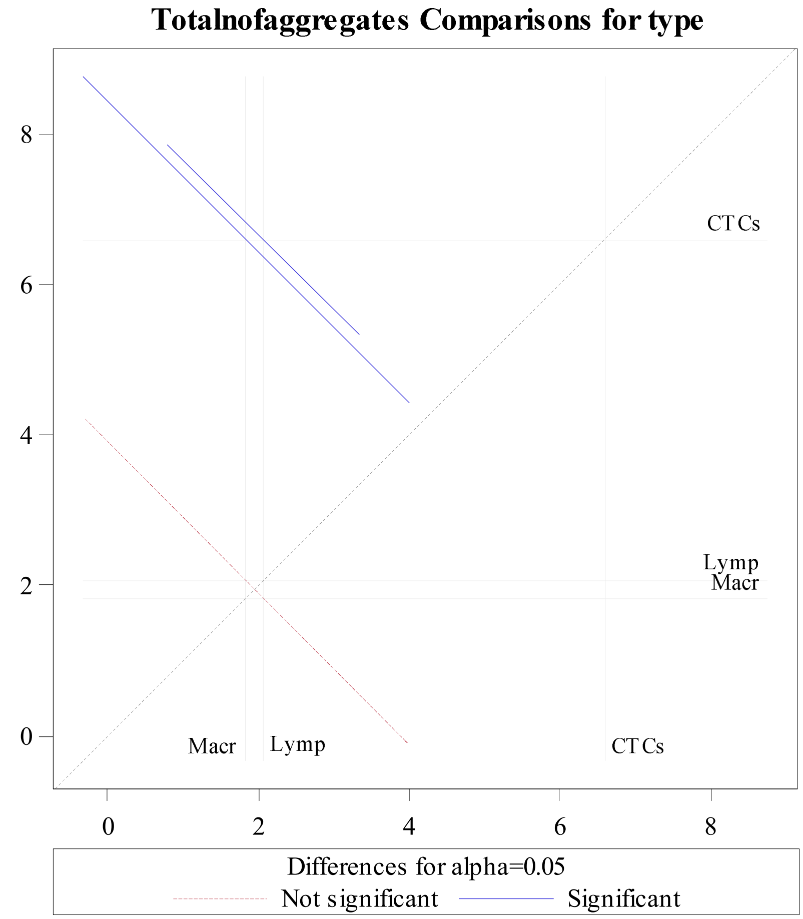

type	acratio LSMEAN	Standard Error	Pr > |t|	LSMEAN Number
**CTCs**	2.55897828	0.36460401	<.0001	1
**Lymp**	1.30594016	0.34589374	0.0004	2
**Macr**	2.76046183	0.77344191	0.0007	3

Least Squares Means for effect type
Pr > |t| for H0: LSMean(i)=LSMean(j)

Dependent Variable: acratio

i/j	1	2	3
**1**		0.0154	0.8145
**2**	0.0154		0.0912
**3**	0.8145	0.0912	

The GLM Procedure
Least Squares Means

pt=16AA2042

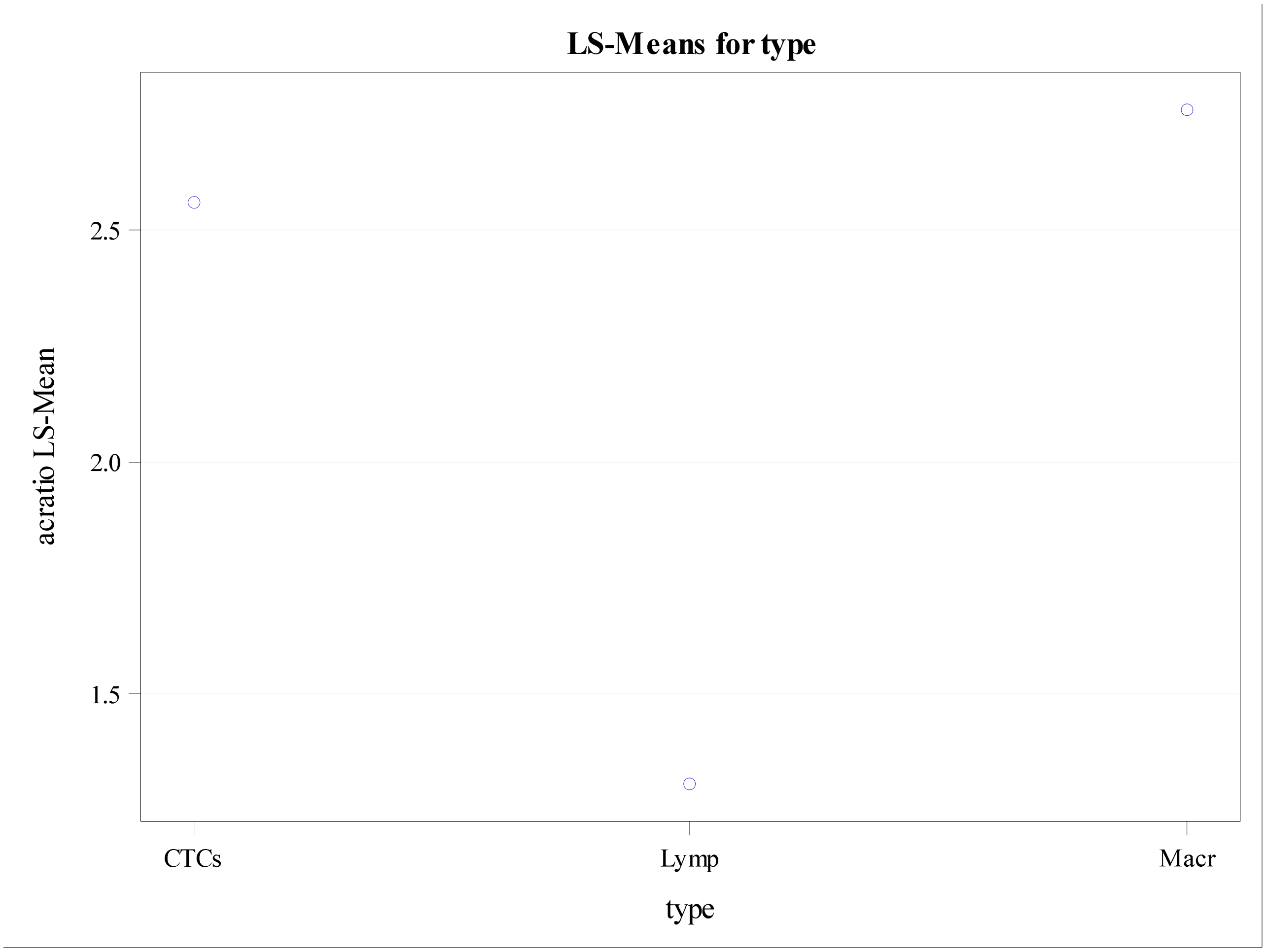

The GLM Procedure
Least Squares Means

pt=16AA2042

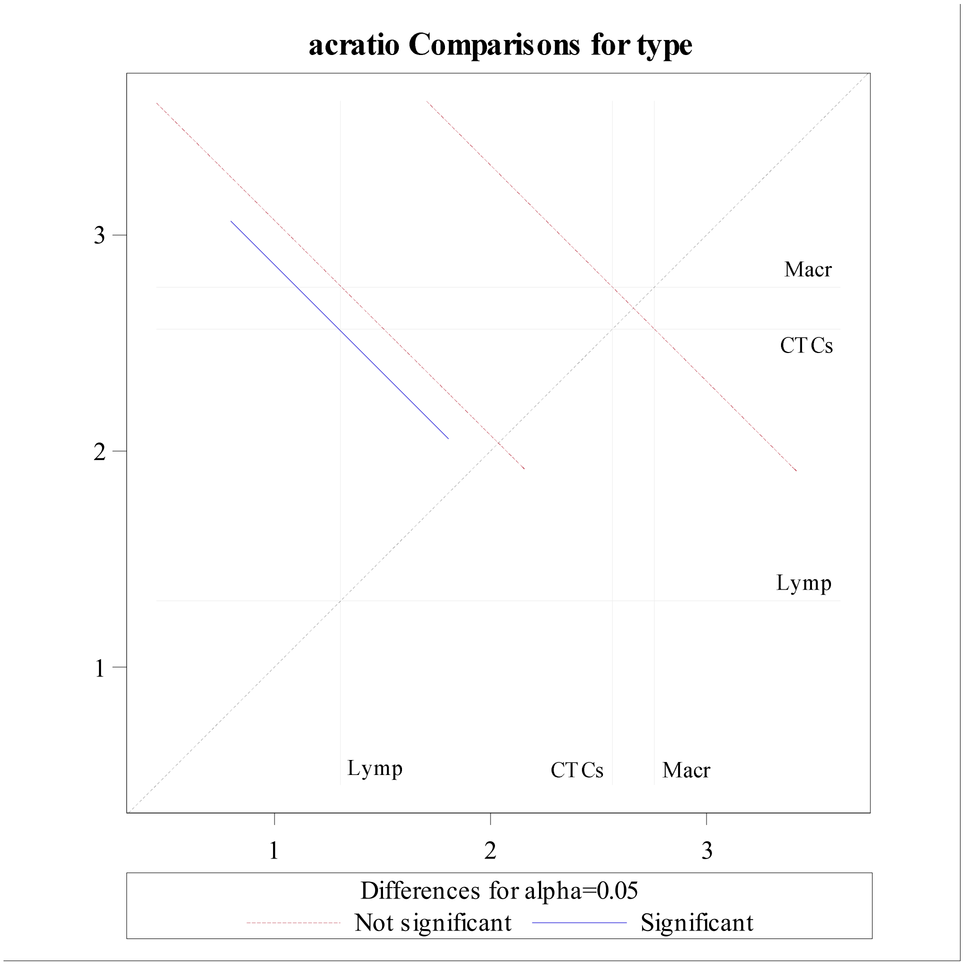

type	AvIntallsignals LSMEAN	Standard Error	Pr > |t|	LSMEAN Number
**CTCs**	11667.1625	856.9676	<.0001	1
**Lymp**	25743.4091	812.9908	<.0001	2
**Macr**	13121.5963	1817.9028	<.0001	3

Least Squares Means for effect type
Pr > |t| for H0: LSMean(i)=LSMean(j)

Dependent Variable: AvIntallsignals

i/j	1	2	3
**1**		<.0001	0.4721
**2**	<.0001		<.0001
**3**	0.4721	<.0001	

The GLM Procedure
Least Squares Means

pt=16AA2042

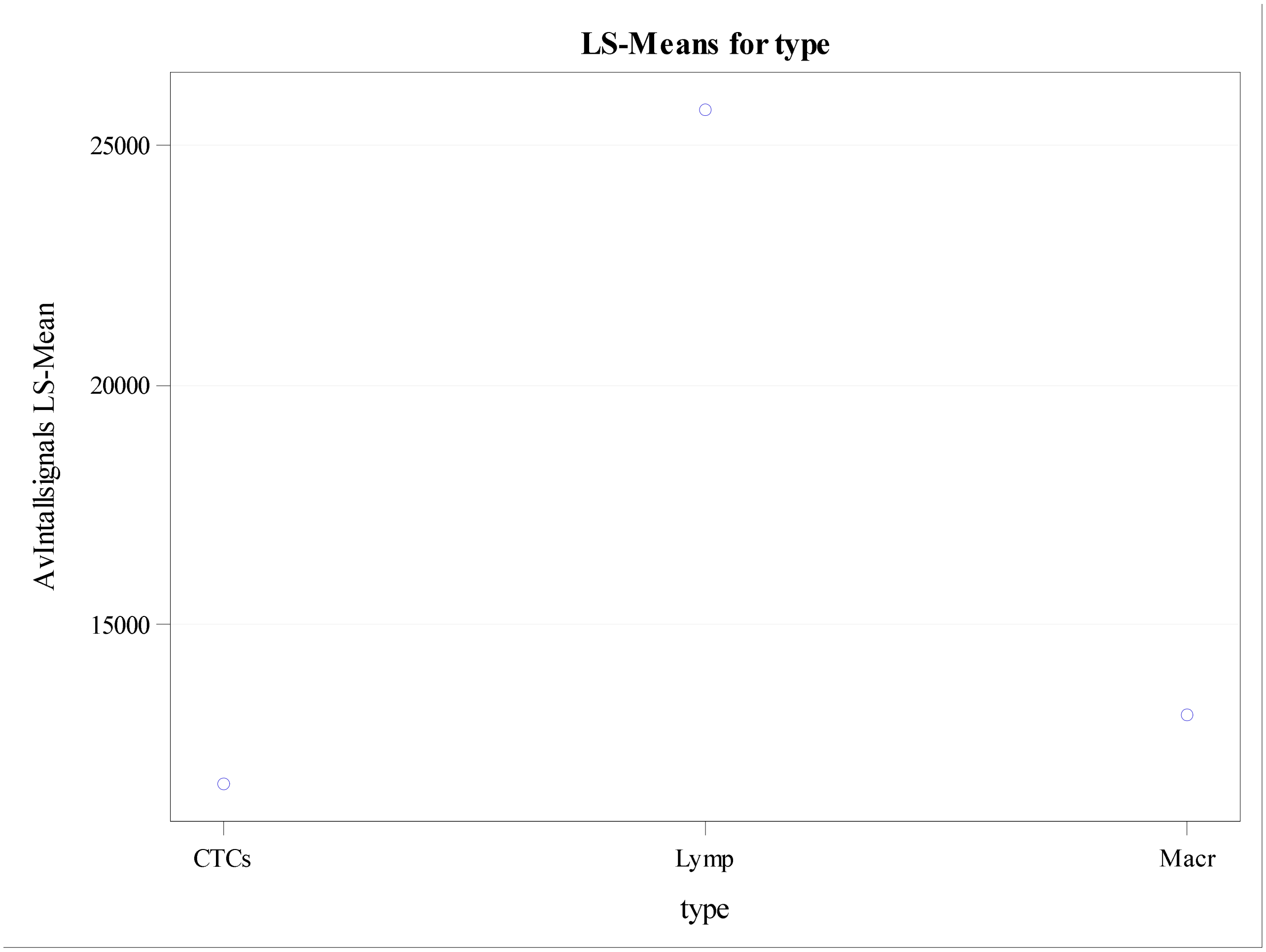

The GLM Procedure
Least Squares Means

pt=16AA2042

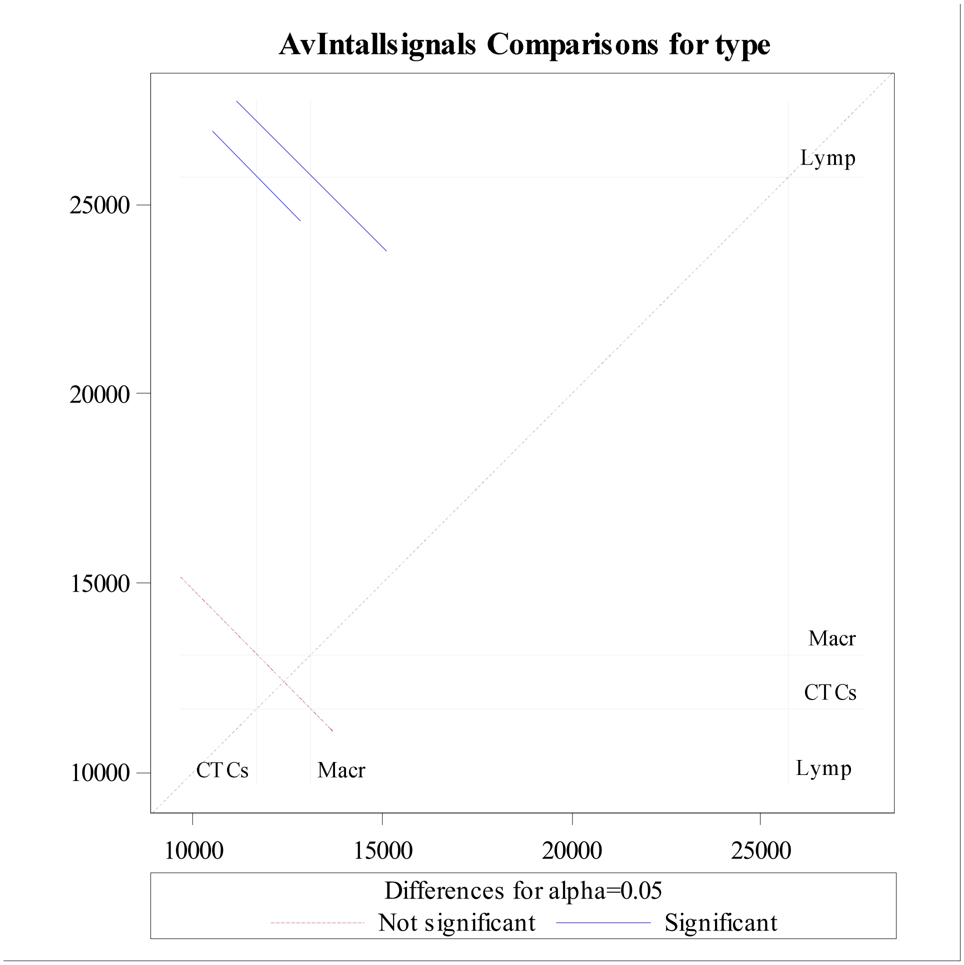

type	Totalintensity LSMEAN	Standard Error	Pr > |t|	LSMEAN Number
**CTCs**	661122.704	59717.009	<.0001	1
**Lymp**	630209.033	56652.529	<.0001	2
**Macr**	264561.167	126678.906	0.0410	3

Least Squares Means for effect type
Pr > |t| for H0: LSMean(i)=LSMean(j)

Dependent Variable: Totalintensity

i/j	1	2	3
**1**		0.7086	0.0063
**2**	0.7086		0.0107
**3**	0.0063	0.0107	

The GLM Procedure
Least Squares Means

pt=16AA2042

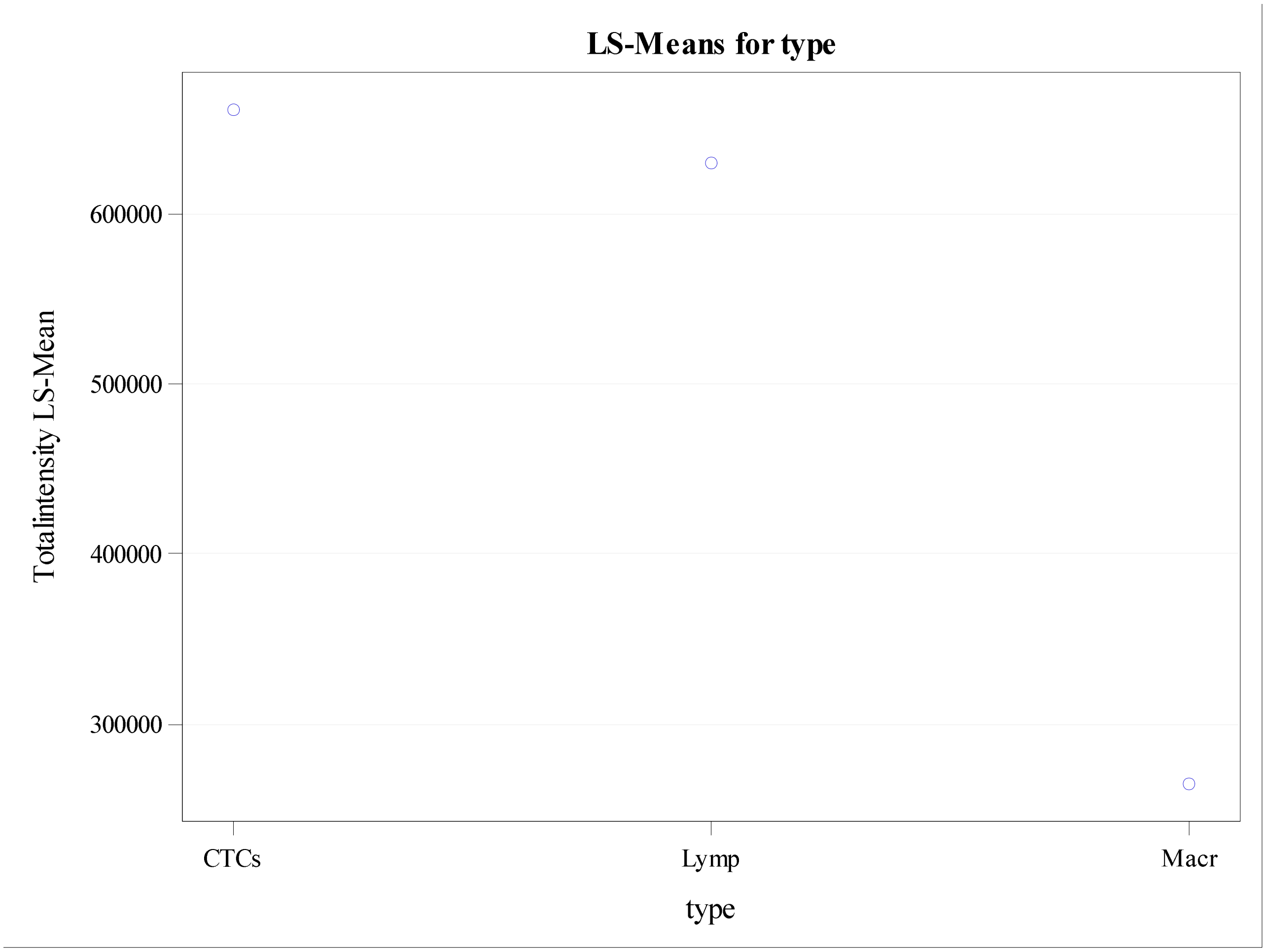

The GLM Procedure
Least Squares Means

pt=16AA2042

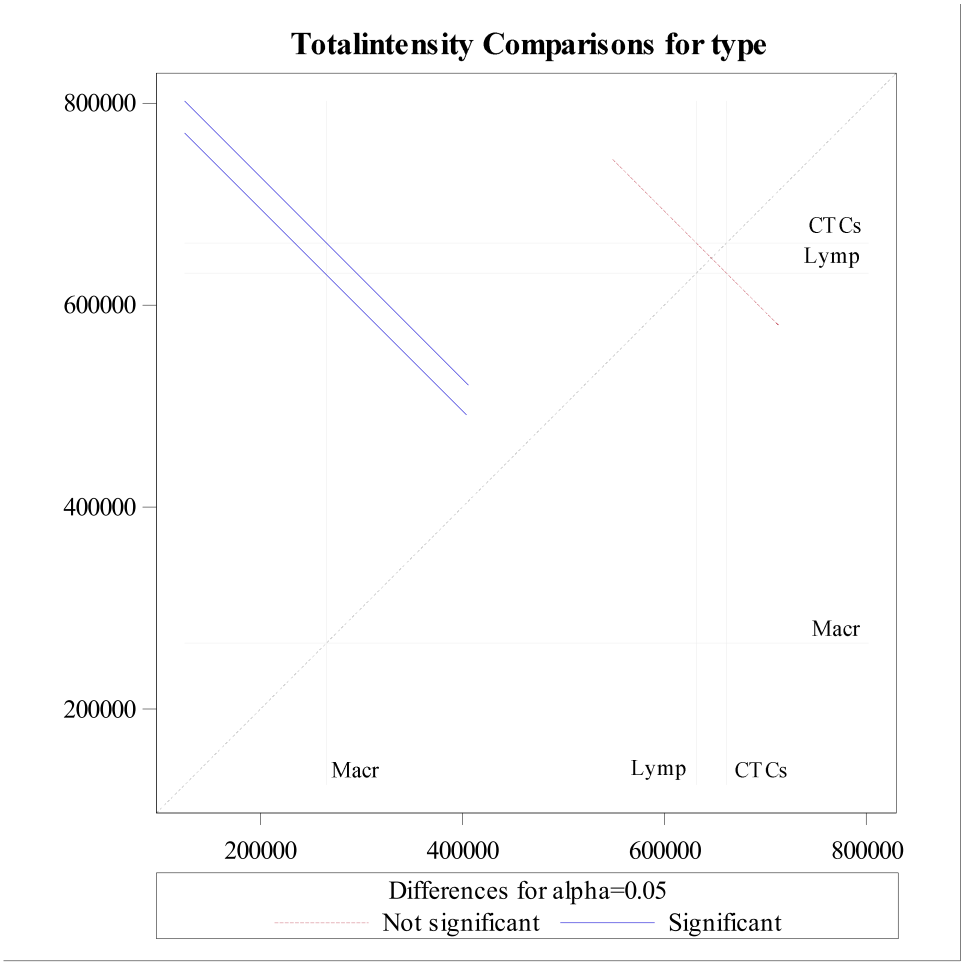

type	Nuclearvolume LSMEAN	Standard Error	Pr > |t|	LSMEAN Number
**CTCs**	832872.222	66351.678	<.0001	1
**Lymp**	152642.800	62946.728	0.0183	2
**Macr**	661307.500	140753.164	<.0001	3

Least Squares Means for effect type<br>Pr > |t| for H0: LSMean(i)=LSMean(j)<br><br>Dependent Variable: Nuclearvolume			
**i/j**	**1**	**2**	**3**
**1**		<.0001	0.2746
**2**	<.0001		0.0016
**3**	0.2746	0.0016	

The GLM Procedure
Least Squares Means

pt=16AA2042

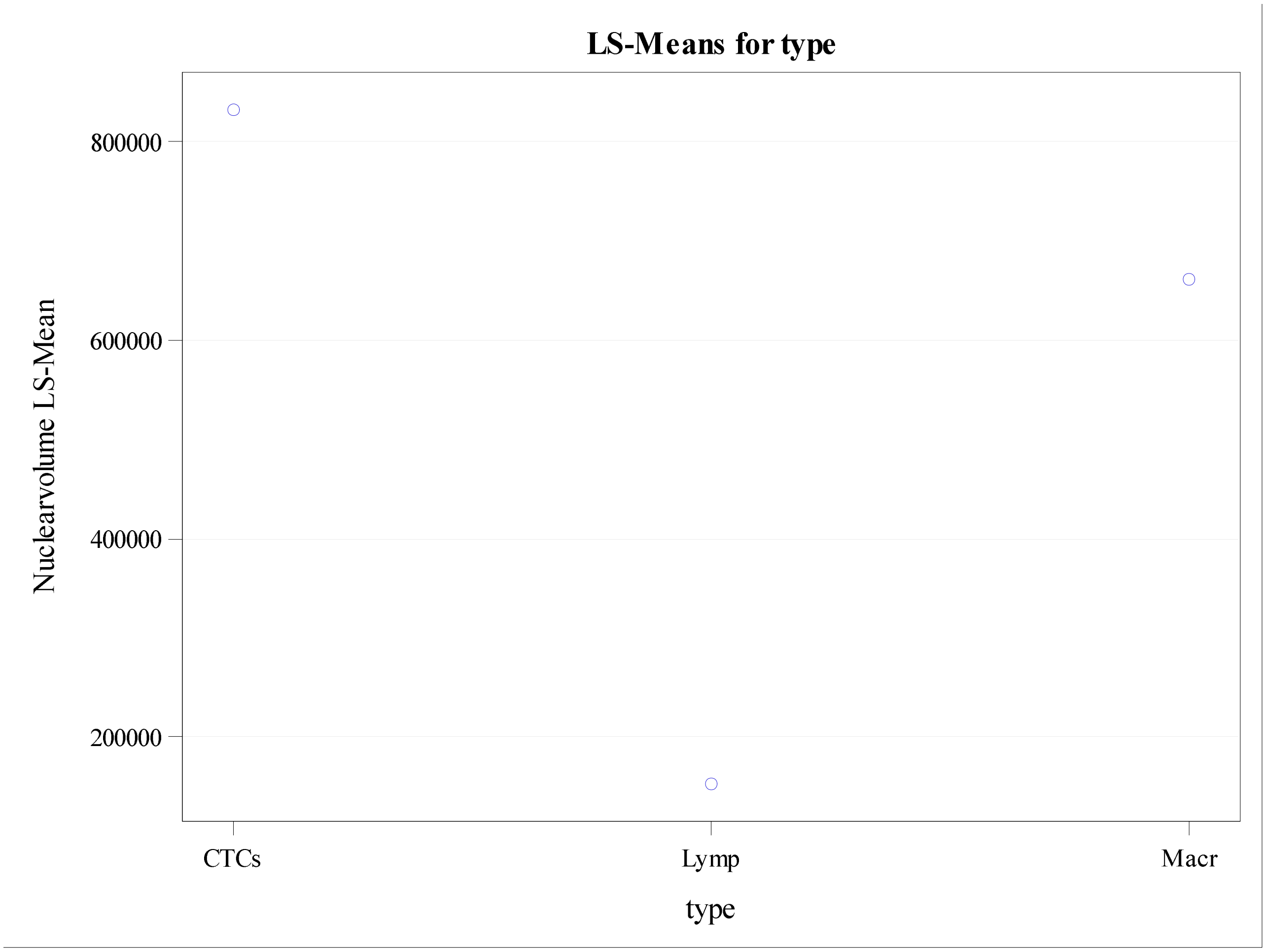

The GLM Procedure
Least Squares Means

pt=16AA2042

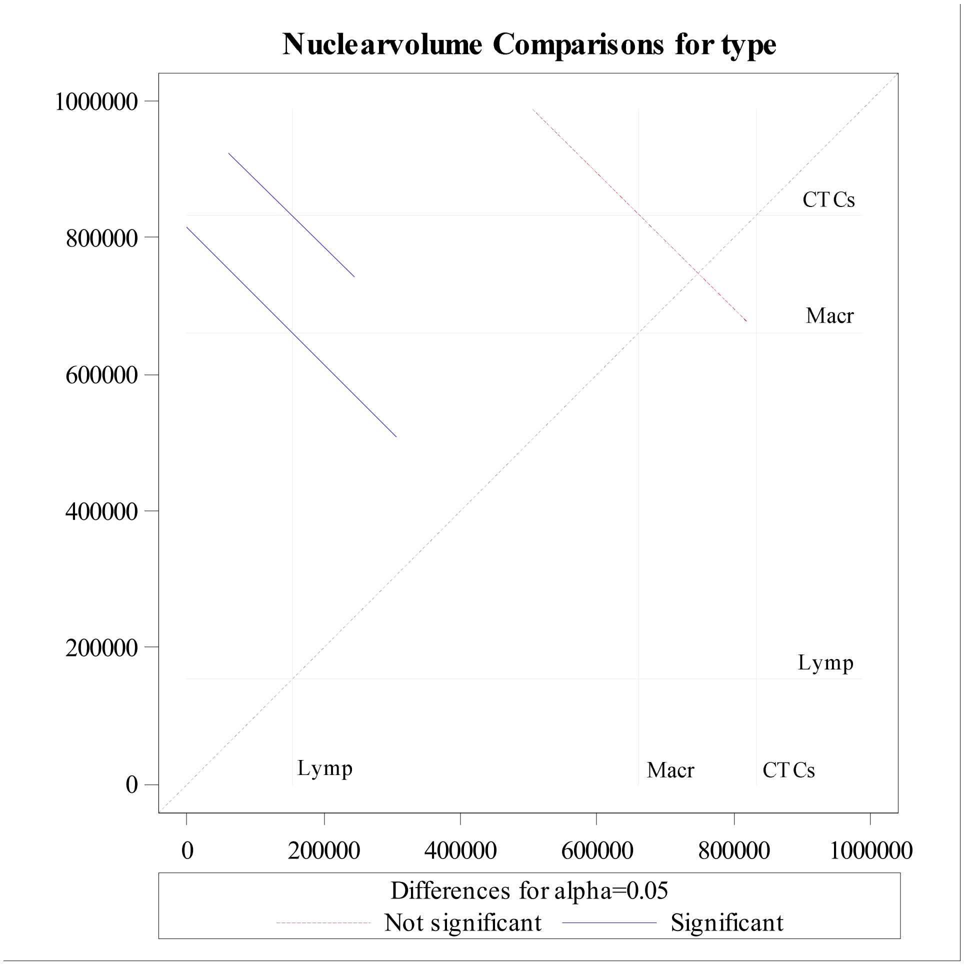

type	telomereperkvol LSMEAN	Standard Error	Pr > |t|	LSMEAN Number
**CTCs**	0.07666026	0.00767449	<.0001	1
**Lymp**	0.16241148	0.00728066	<.0001	2
**Macr**	0.03353455	0.01628005	0.0438	3

Least Squares Means for effect type
Pr > |t| for H0: LSMean(i)=LSMean(j)

Dependent Variable: telomereperkvol

i/j	1	2	3
**1**		<.0001	0.0197
**2**	<.0001		<.0001
**3**	0.0197	<.0001	

The GLM Procedure
Least Squares Means

pt=16AA2042

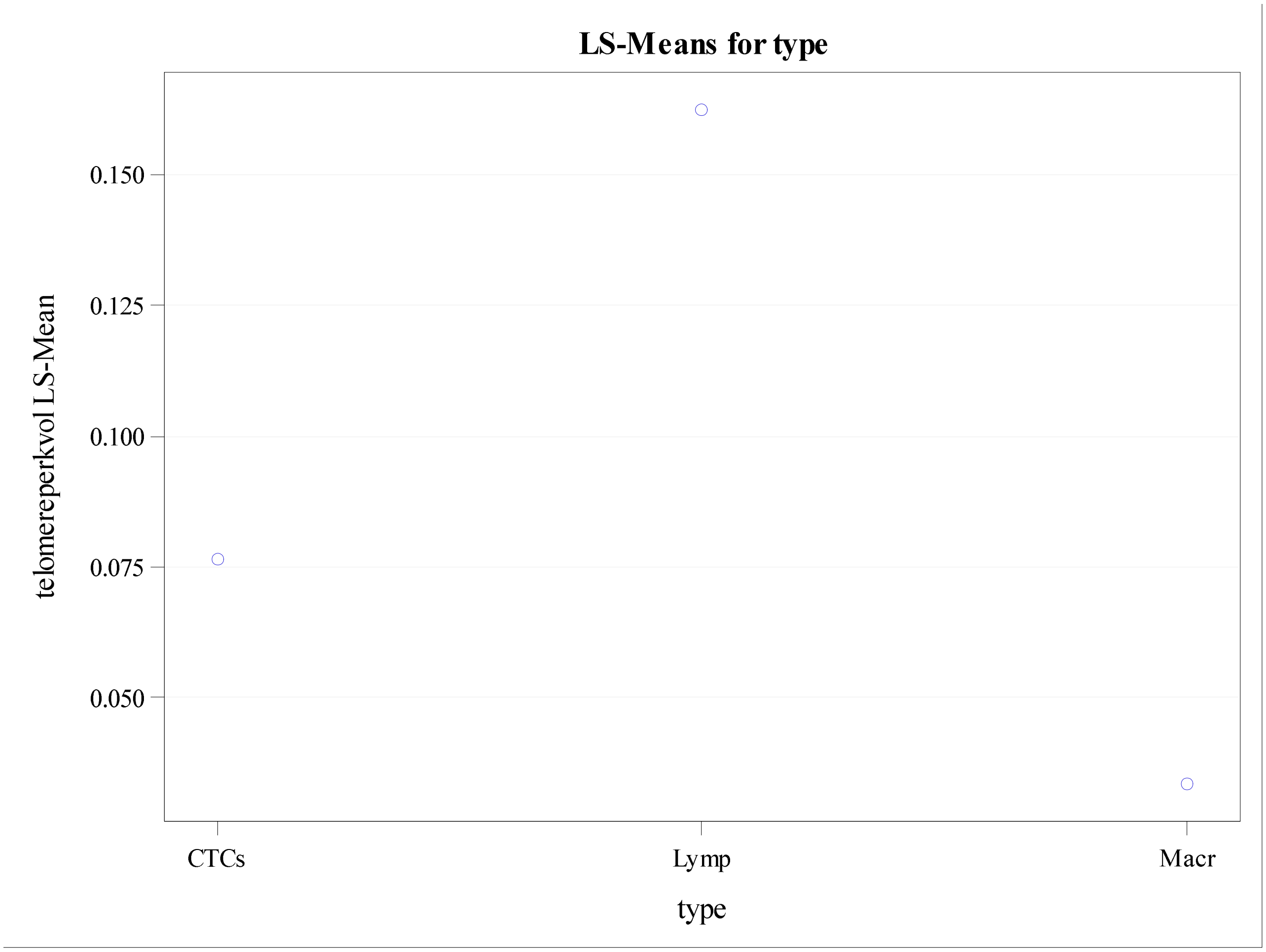

The GLM Procedure
Least Squares Means

pt=16AA2042

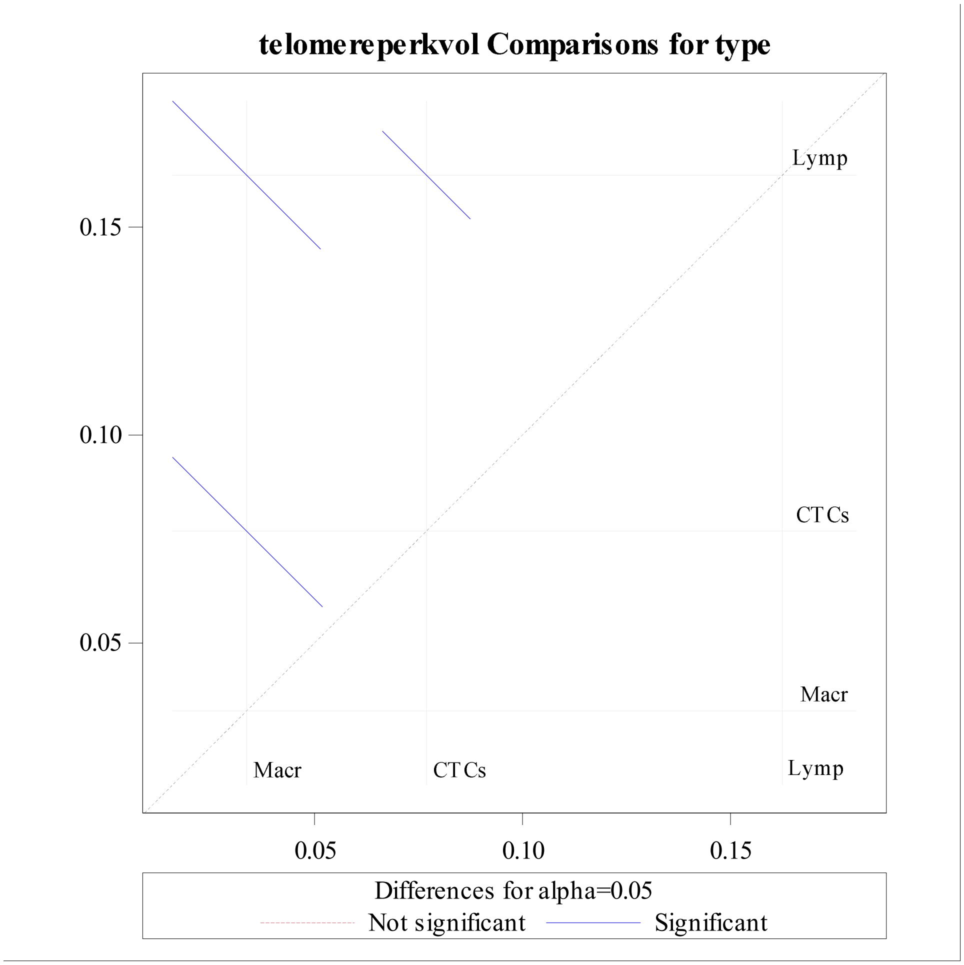

Note: To ensure overall protection level, only probabilities associated with pre-planned comparisons should be used.

The GLM Procedure

pt=16AA7266

Class Level Information		
**Class**	**Levels**	**Values**
**type**	3	CTCs Lymp Macr

**Number of Observations Read**	83
**Number of Observations Used**	83

The GLM Procedure

Dependent Variable: Totalnofsignals Totalnofsignals

pt=16AA7266

Source	DF	Sum of Squares	Mean Square	F Value	Pr > F
**Model**	2	6658.21552	3329.10776	9.78	0.0002
**Error**	80	27238.67605	340.48345		
**Corrected Total**	82	33896.89157			

R-Square	Coeff Var	Root MSE	Totalnofsignals Mean
0.196426	65.81573	18.45219	28.03614

Source	DF	Type III SS	Mean Square	F Value	Pr > F
**type**	2	6658.215515	3329.107758	9.78	0.0002

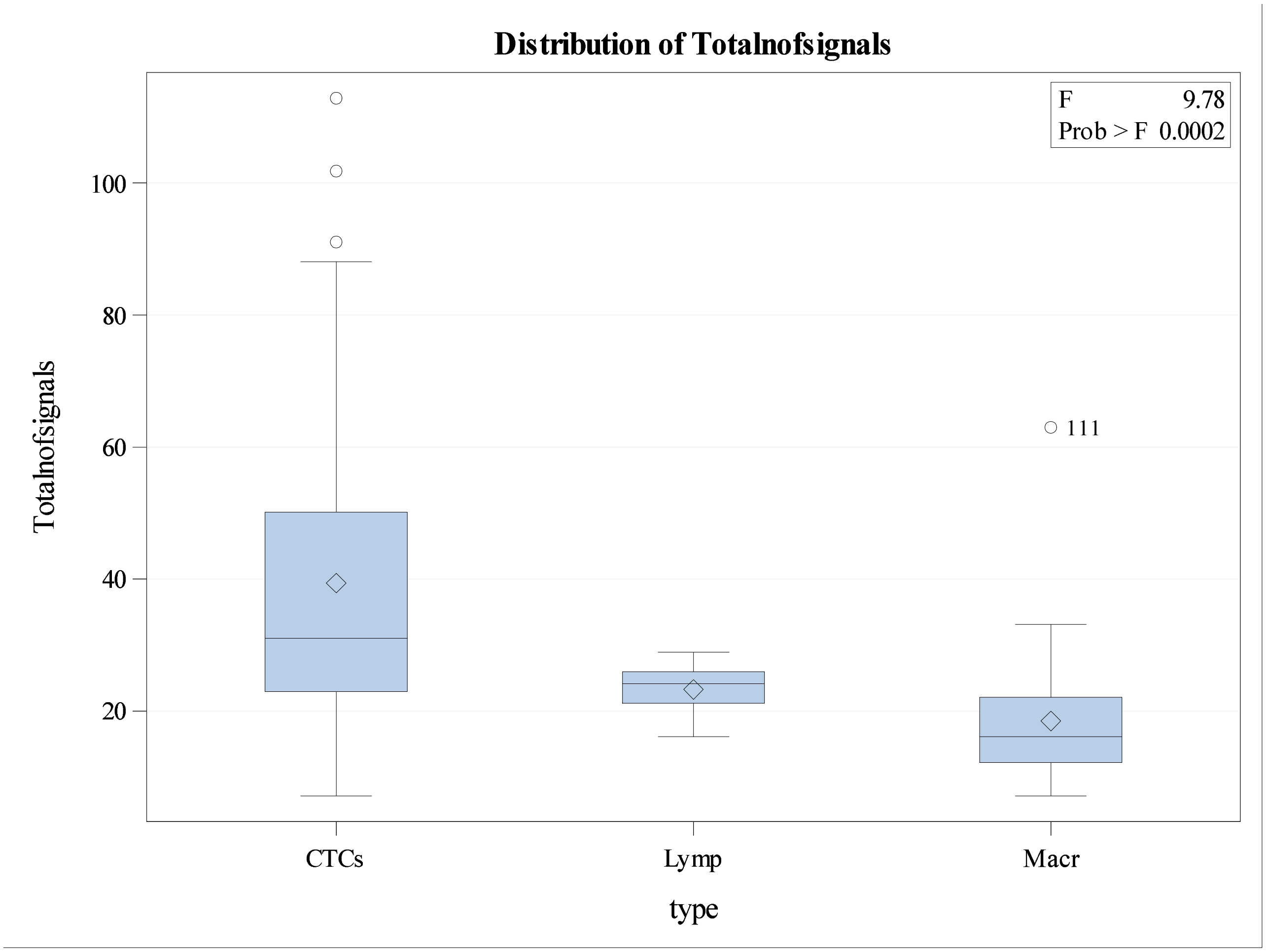

The GLM Procedure

Dependent Variable: Totalnofaggregates Totalnofaggregates

pt=16AA7266

Source	DF	Sum of Squares	Mean Square	F Value	Pr > F
**Model**	2	121.7746434	60.8873217	7.58	0.0010
**Error**	80	642.6349951	8.0329374		
**Corrected Total**	82	764.4096386			

R-Square	Coeff Var	Root MSE	Totalnofaggregates Mean
0.159305	97.20753	2.834244	2.915663

Source	DF	Type III SS	Mean Square	F Value	Pr > F
**type**	2	121.7746434	60.8873217	7.58	0.0010

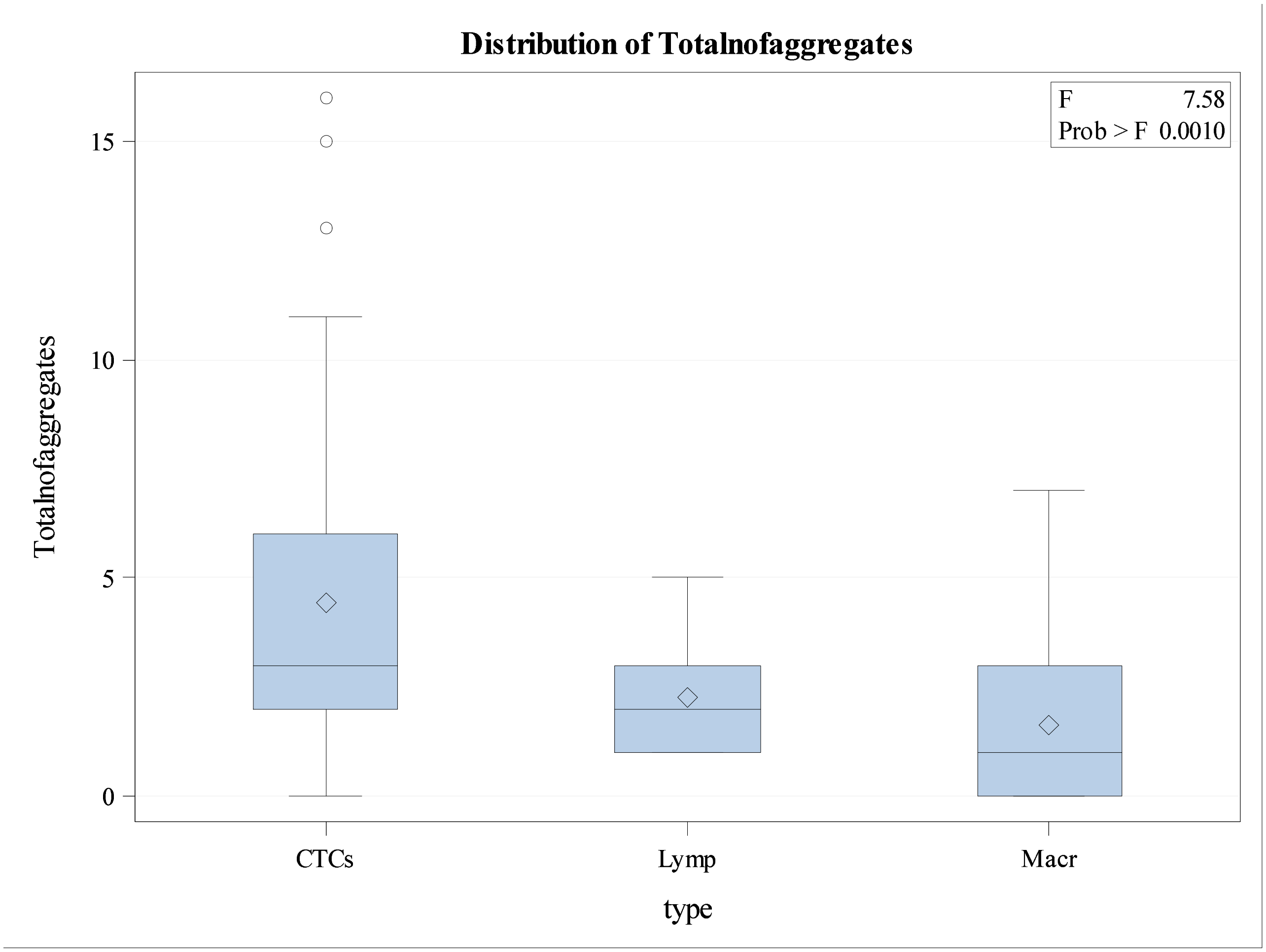

The GLM Procedure

Dependent Variable: acratio
acratio

pt=16AA7266

Source	DF	Sum of Squares	Mean Square	F Value	Pr > F
**Model**	2	15.0083748	7.5041874	2.53	0.0860
**Error**	80	237.2223645	2.9652796		
**Corrected Total**	82	252.2307393			

R-Square	Coeff Var	Root MSE	acratio Mean
0.059503	72.03649	1.721999	2.390454

Source	DF	Type III SS	Mean Square	F Value	Pr > F
**type**	2	15.00837480	7.50418740	2.53	0.0860

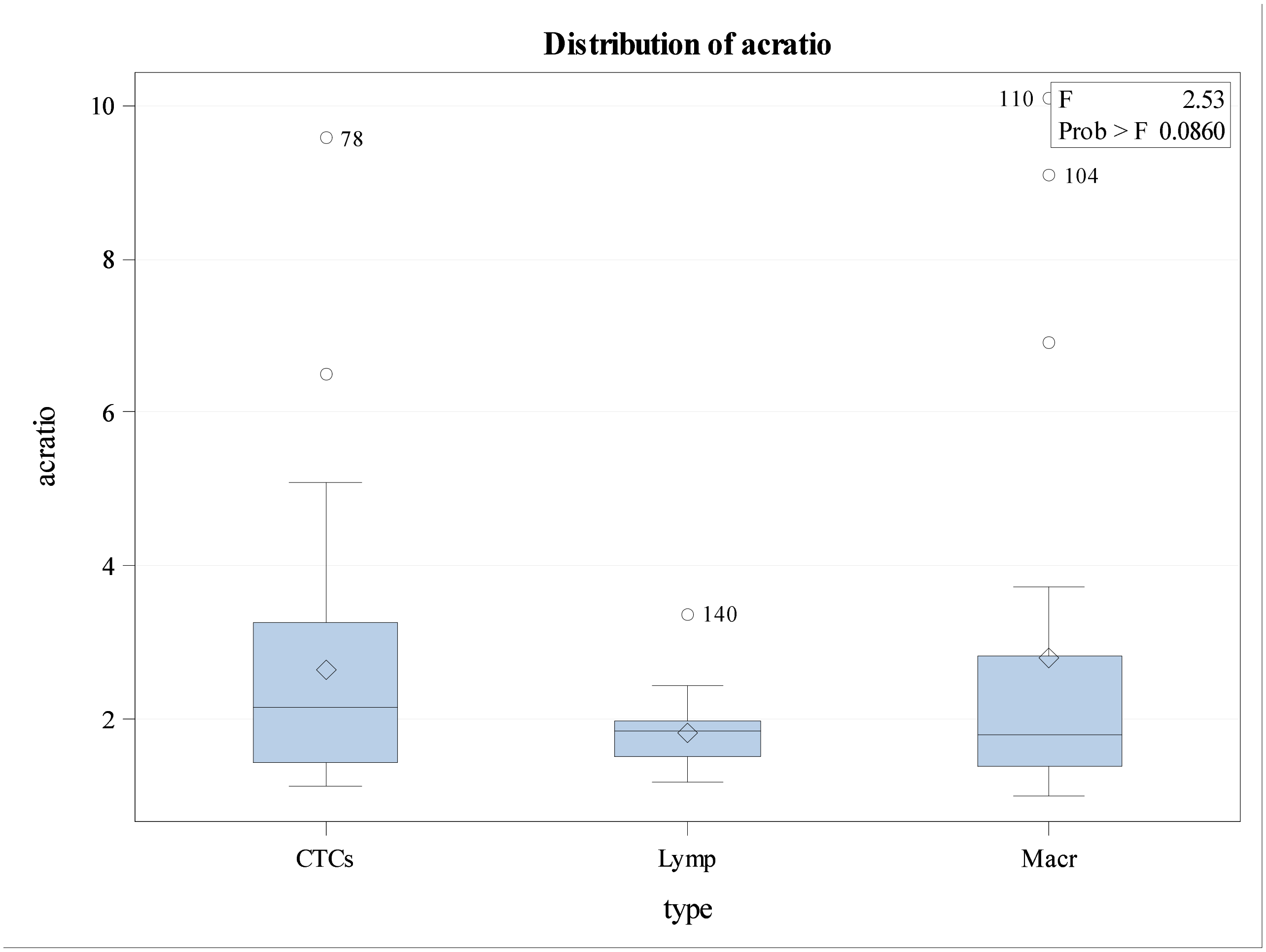

The GLM Procedure

Dependent Variable: AvIntallsignals
AvIntallsignals

pt=16AA7266

Source	DF	Sum of Squares	Mean Square	F Value	Pr > F
**Model**	2	2397076160	1198538080	52.52	<.0001
**Error**	80	1825778070	22822226		
**Corrected Total**	82	4222854231			

R-Square	Coeff Var	Root MSE	AvIntallsignals Mean
0.567644	31.36752	4777.261	15229.96

Source	DF	Type III SS	Mean Square	F Value	Pr > F
**type**	2	2397076160	1198538080	52.52	<.0001

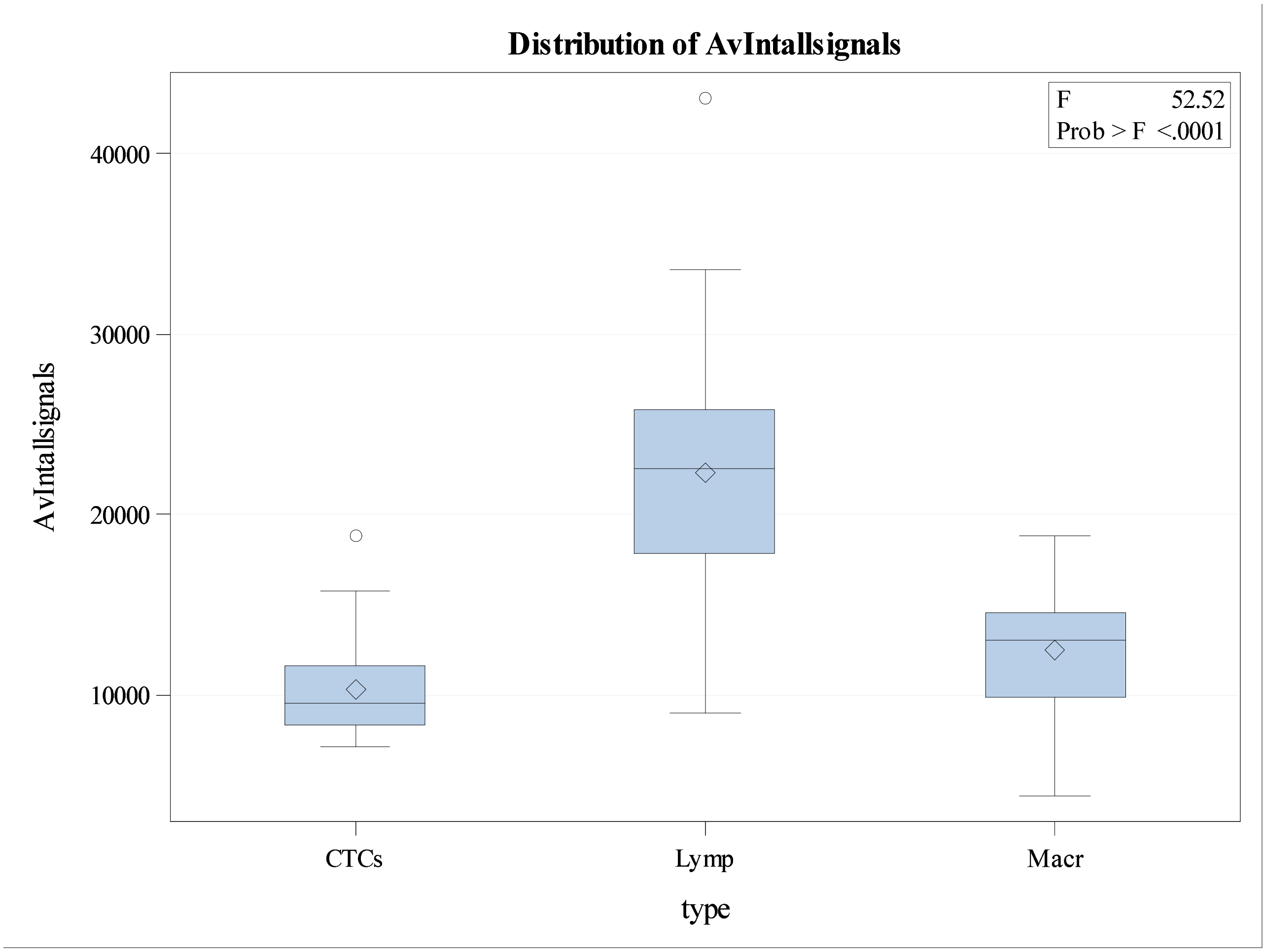

The GLM Procedure

Dependent Variable: Totalintensity Totalintensity

pt=16AA7266

Source	DF	Sum of Squares	Mean Square	F Value	Pr > F
**Model**	2	1.0046272E12	502313590088	14.99	<.0001
**Error**	80	2.681234E12	33515424484		
**Corrected Total**	82	3.6858611E12			

R-Square	Coeff Var	Root MSE	Totalintensity Mean
0.272562	47.20276	183072.2	387842.1

Source	DF	Type III SS	Mean Square	F Value	Pr > F
**type**	2	1.0046272E12	502313590088	14.99	<.0001

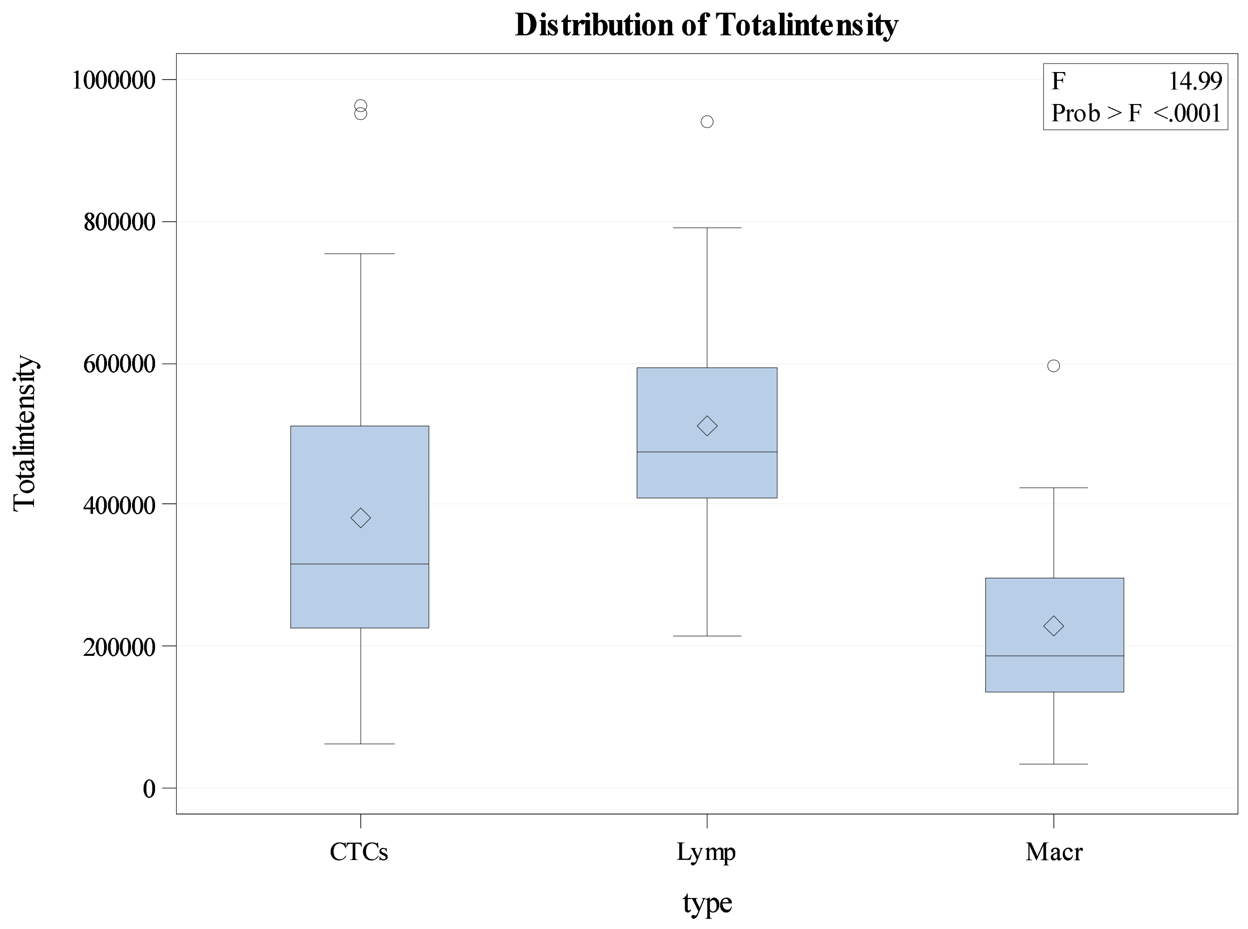

The GLM Procedure

Dependent Variable: Nuclearvolume Nuclearvolume

pt=16AA7266

Source	DF	Sum of Squares	Mean Square	F Value	Pr > F
**Model**	2	8.8354185E12	4.4177093E12	74.23	<.0001
**Error**	80	4.7610272E12	59512840558		
**Corrected Total**	82	1.3596446E13			

R-Square	Coeff Var	Root MSE	Nuclearvolume Mean
0.649833	44.54691	243952.5	547630.7

Source	DF	Type III SS	Mean Square	F Value	Pr > F
**type**	2	8.8354185E12	4.4177093E12	74.23	<.0001

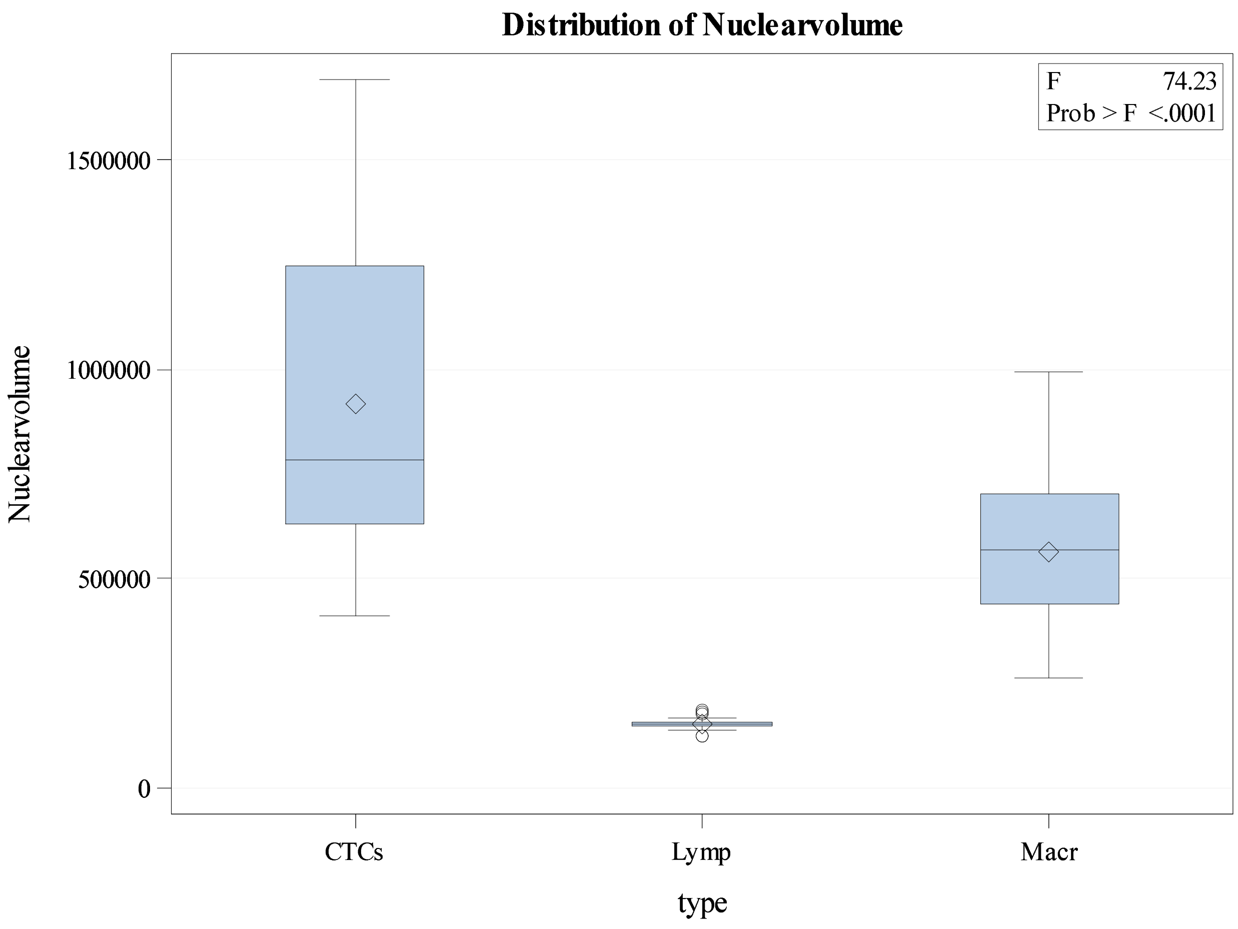

The GLM Procedure

Dependent Variable: telomereperkvol

pt=16AA7266

Source	DF	Sum of Squares	Mean Square	F Value	Pr > F
**Model**	2	0.24360278	0.12180139	239.53	<.0001
**Error**	80	0.04068063	0.00050851		
**Corrected Total**	82	0.28428341			

R-Square	Coeff Var	Root MSE	telomereperkvol Mean
0.856901	28.44140	0.022550	0.079286

Source	DF	Type III SS	Mean Square	F Value	Pr > F
**type**	2	0.24360278	0.12180139	239.53	<.0001

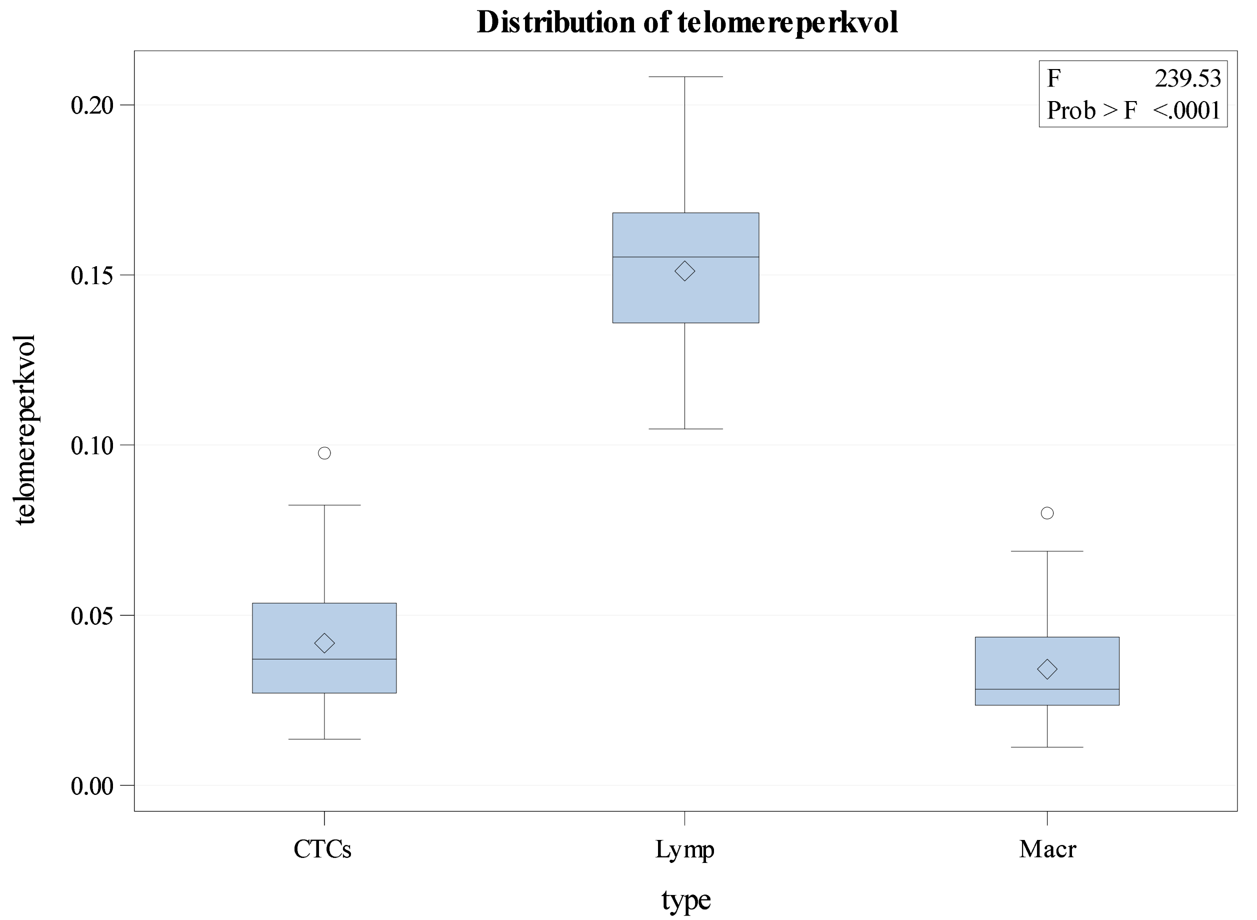

The GLM Procedure
Least Squares Means

pt=16AA7266

type	Totalnofsignals LSMEAN	Standard Error	Pr > |t|	LSMEAN Number
**CTCs**	39.3870968	3.3141118	<.0001	1
**Lymp**	23.2666667	3.3688942	<.0001	2
**Macr**	18.5454545	3.9340209	<.0001	3

Least Squares Means for effect type
Pr > |t| for H0: LSMean(i)=LSMean(j)

Dependent Variable: Totalnofsignals

i/j	1	2	3
**1**		0.0010	0.0001
**2**	0.0010		0.3647
**3**	0.0001	0.3647	

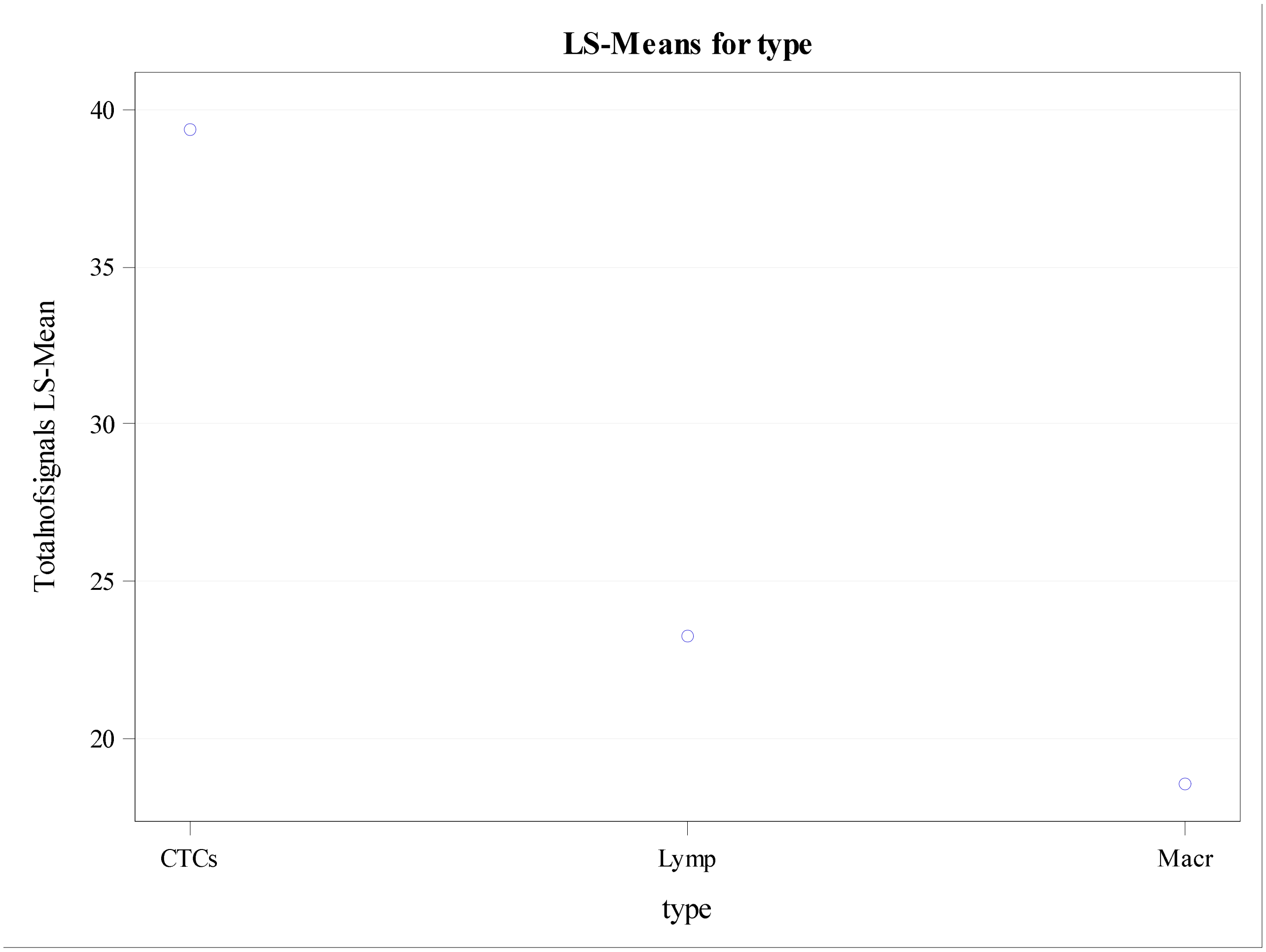

The GLM Procedure
Least Squares Means

pt=16AA7266

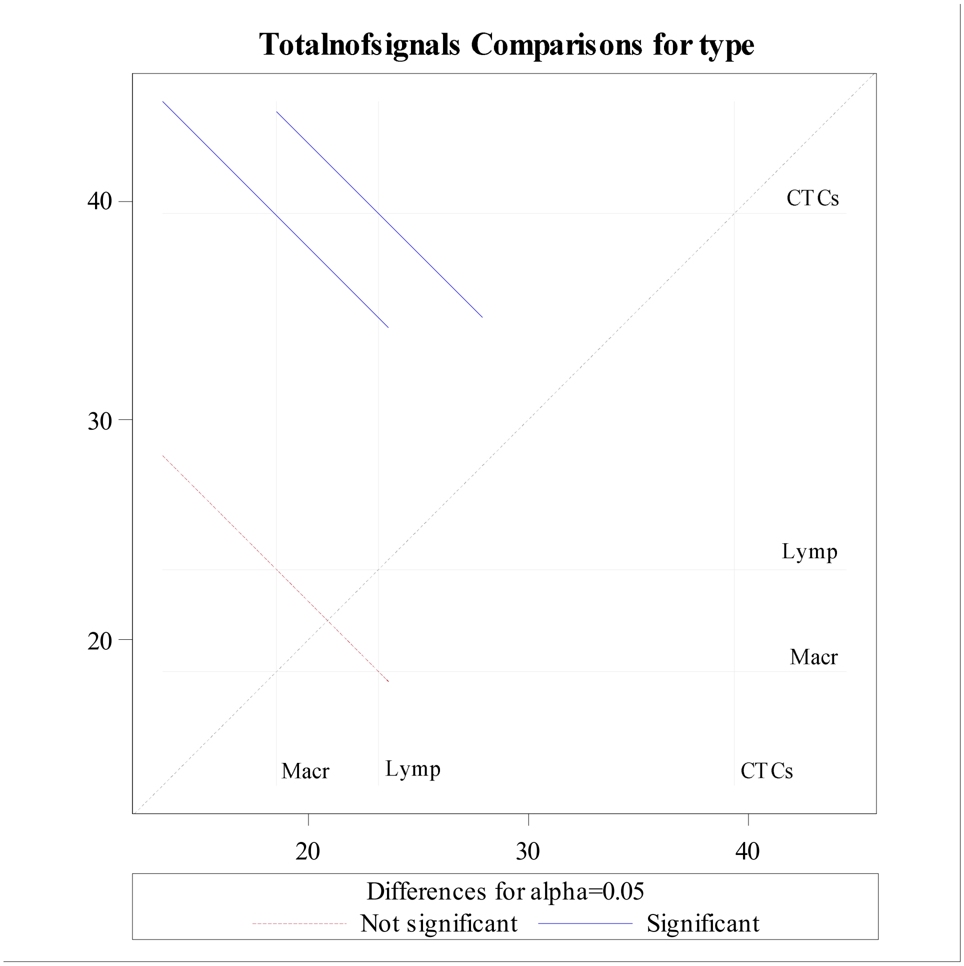

type	Totalnofaggregates LSMEAN	Standard Error	Pr > |t|	LSMEAN Number
**CTCs**	4.45161290	0.50904520	<.0001	1
**Lymp**	2.26666667	0.51745974	<.0001	2
**Macr**	1.63636364	0.60426279	0.0083	3

The GLM Procedure
Least Squares Means

pt=16AA7266

Least Squares Means for effect type<br>Pr > |t| for H0: LSMean(i)=LSMean(j)<br><br>Dependent Variable: Totalnofaggregates			
**i/j**	**1**	**2**	**3**
**1**		0.0035	0.0006
**2**	0.0035		0.4305
**3**	0.0006	0.4305	

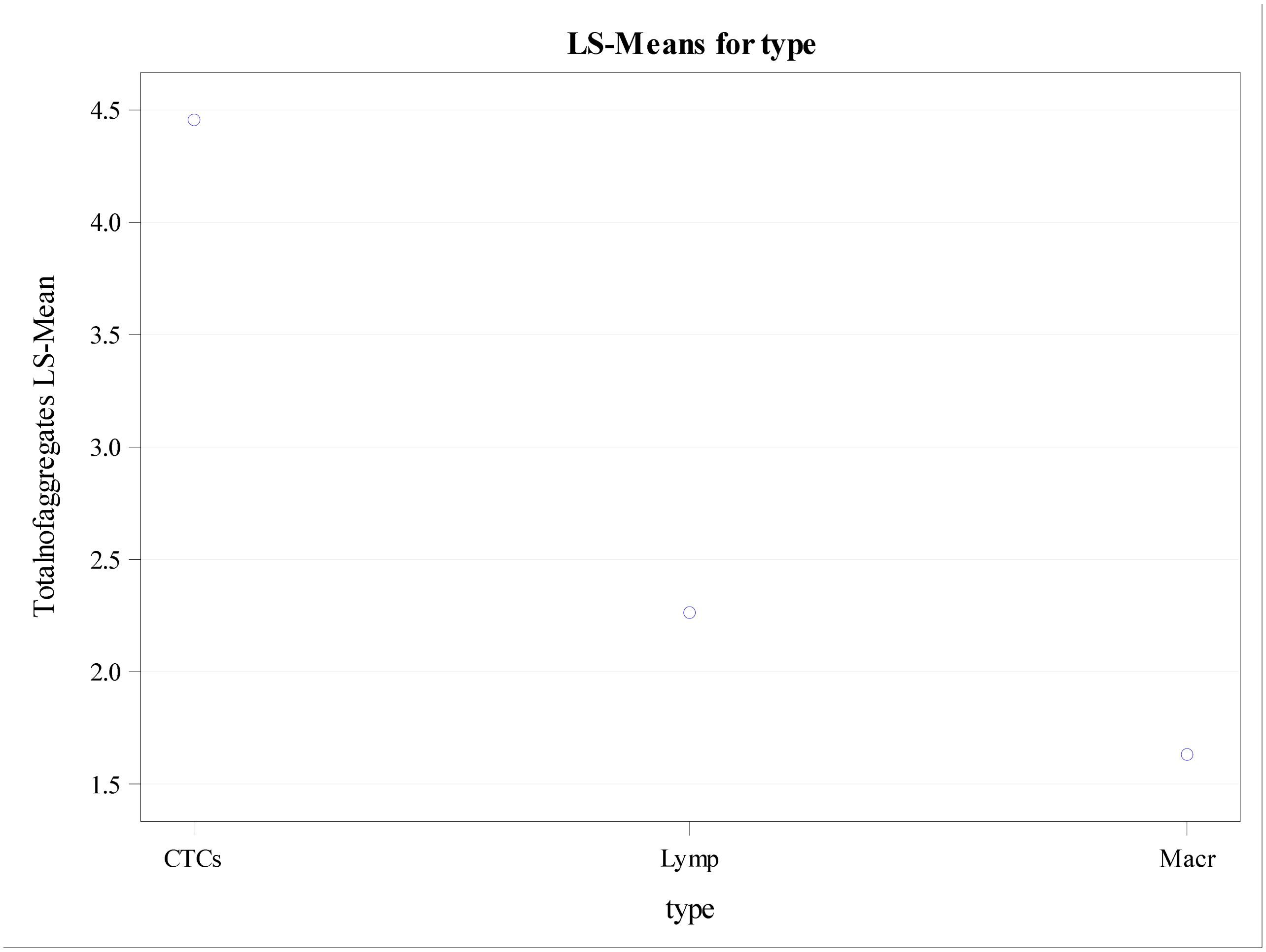

The GLM Procedure
Least Squares Means

pt=16AA7266

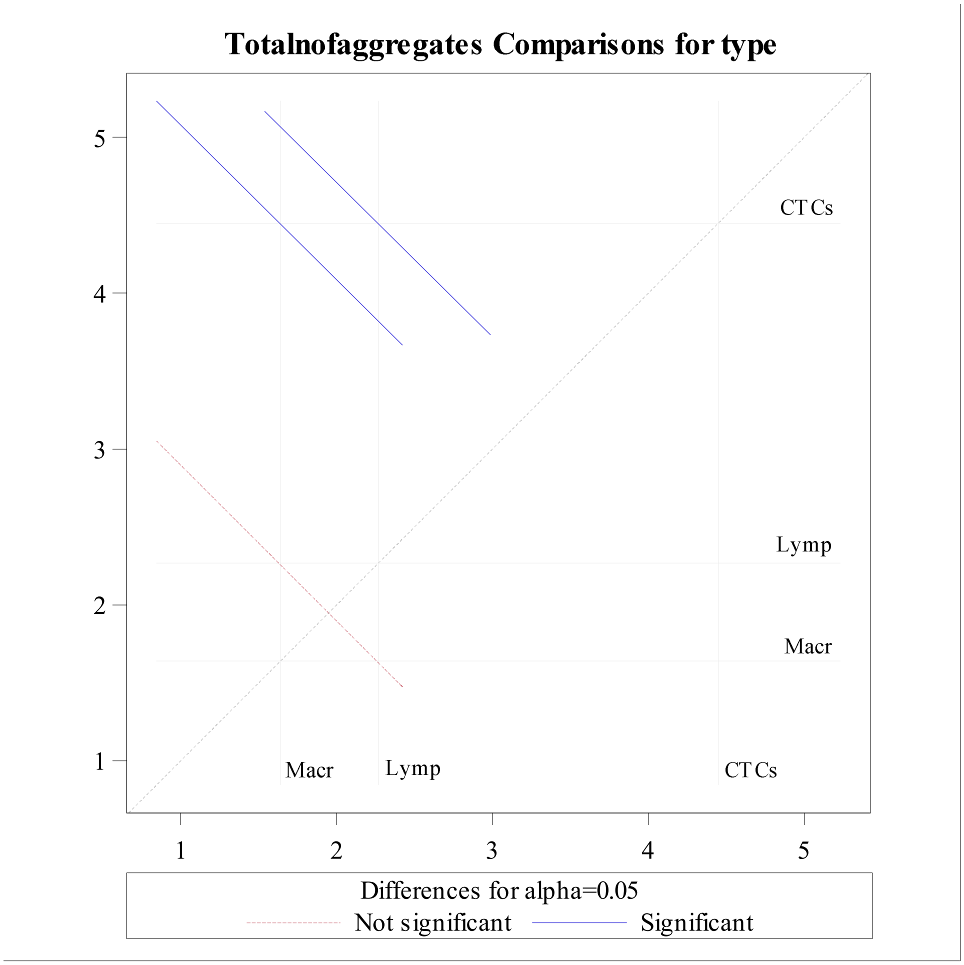

type	acratio LSMEAN	Standard Error	Pr > |t|	LSMEAN Number
**CTCs**	2.63963675	0.30928010	<.0001	1
**Lymp**	1.83162377	0.31439251	<.0001	2
**Macr**	2.80137230	0.36713136	<.0001	3

Least Squares Means for effect type
Pr > |t| for H0: LSMean(i)=LSMean(j)

Dependent Variable: acratio

i/j	1	2	3
**1**		0.0706	0.7371
**2**	0.0706		0.0482
**3**	0.7371	0.0482	

The GLM Procedure
Least Squares Means

pt=16AA7266

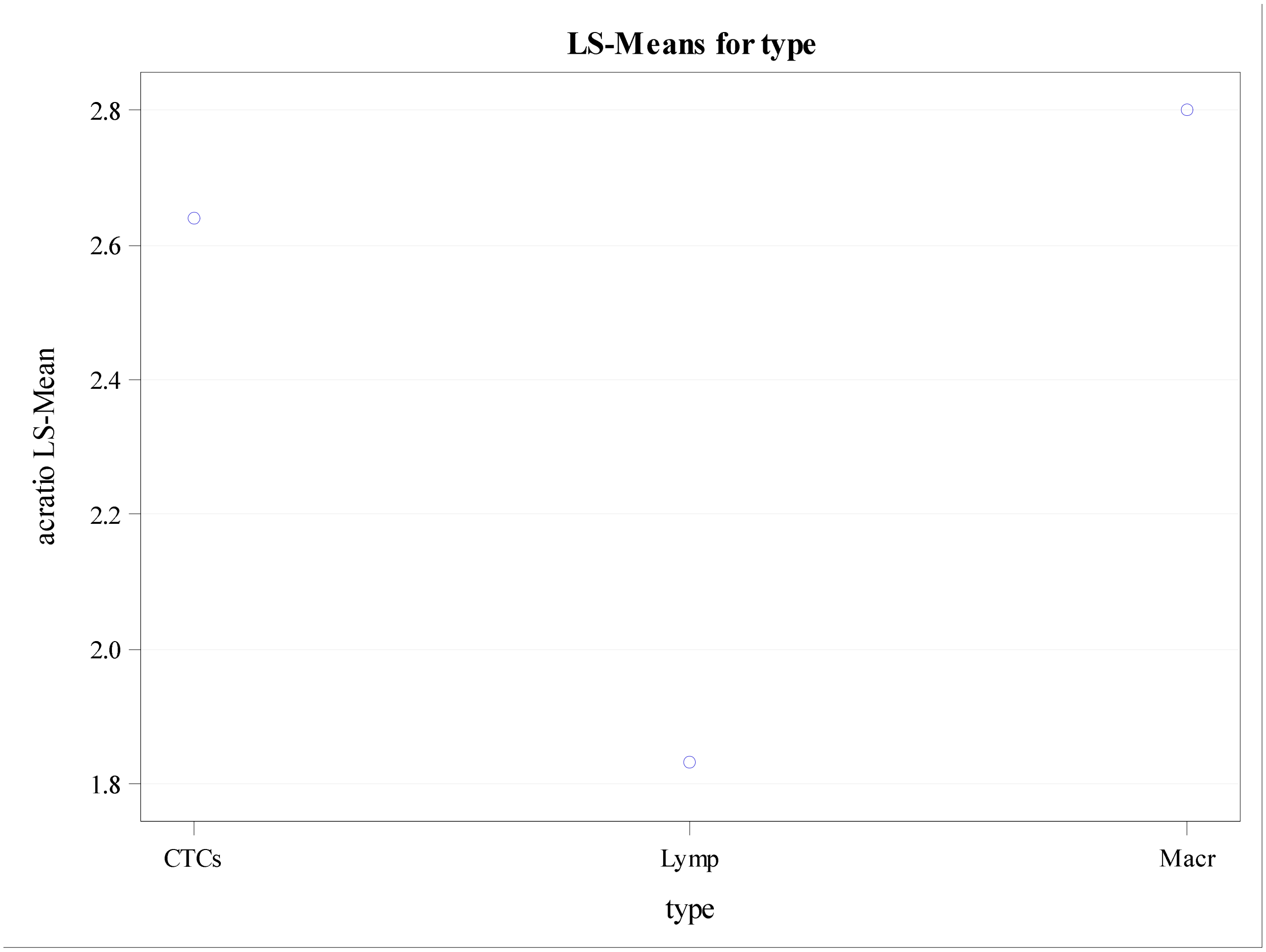

The GLM Procedure
Least Squares Means

pt=16AA7266

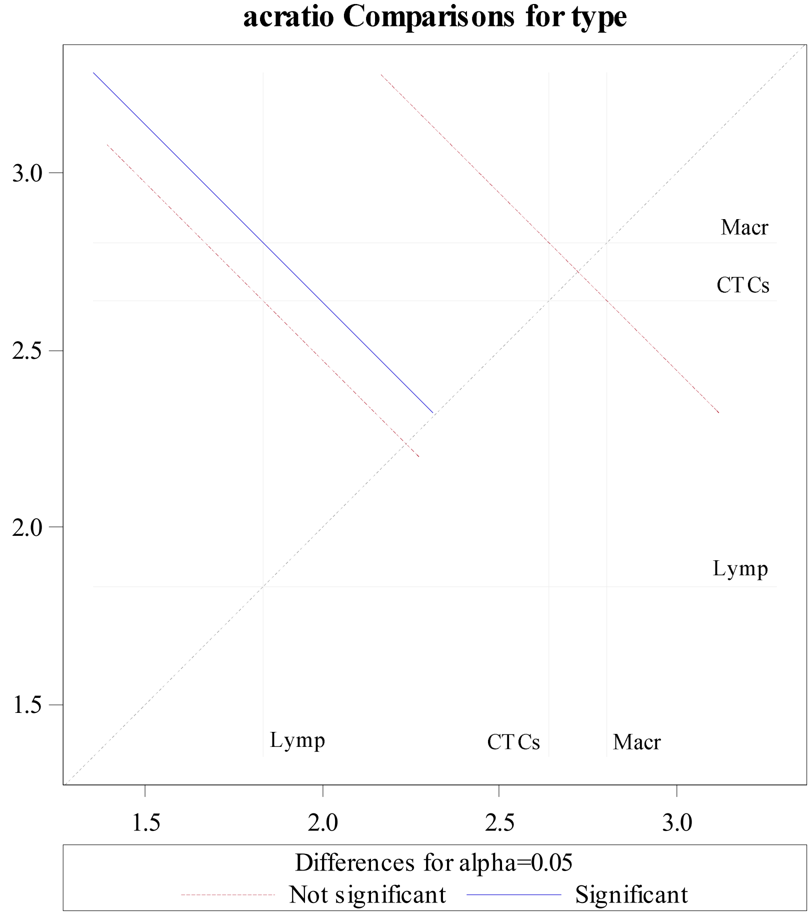

type	AvIntallsignals LSMEAN	Standard Error	Pr > |t|	LSMEAN Number
**CTCs**	10358.9711	858.0215	<.0001	1
**Lymp**	22286.9418	872.2046	<.0001	2
**Macr**	12470.4814	1018.5155	<.0001	3

Least Squares Means for effect type
Pr > |t| for H0: LSMean(i)=LSMean(j)

Dependent Variable: AvIntallsignals

i/j	1	2	3
**1**		<.0001	0.1168
**2**	<.0001		<.0001
**3**	0.1168	<.0001	

The GLM Procedure
Least Squares Means

pt=16AA7266

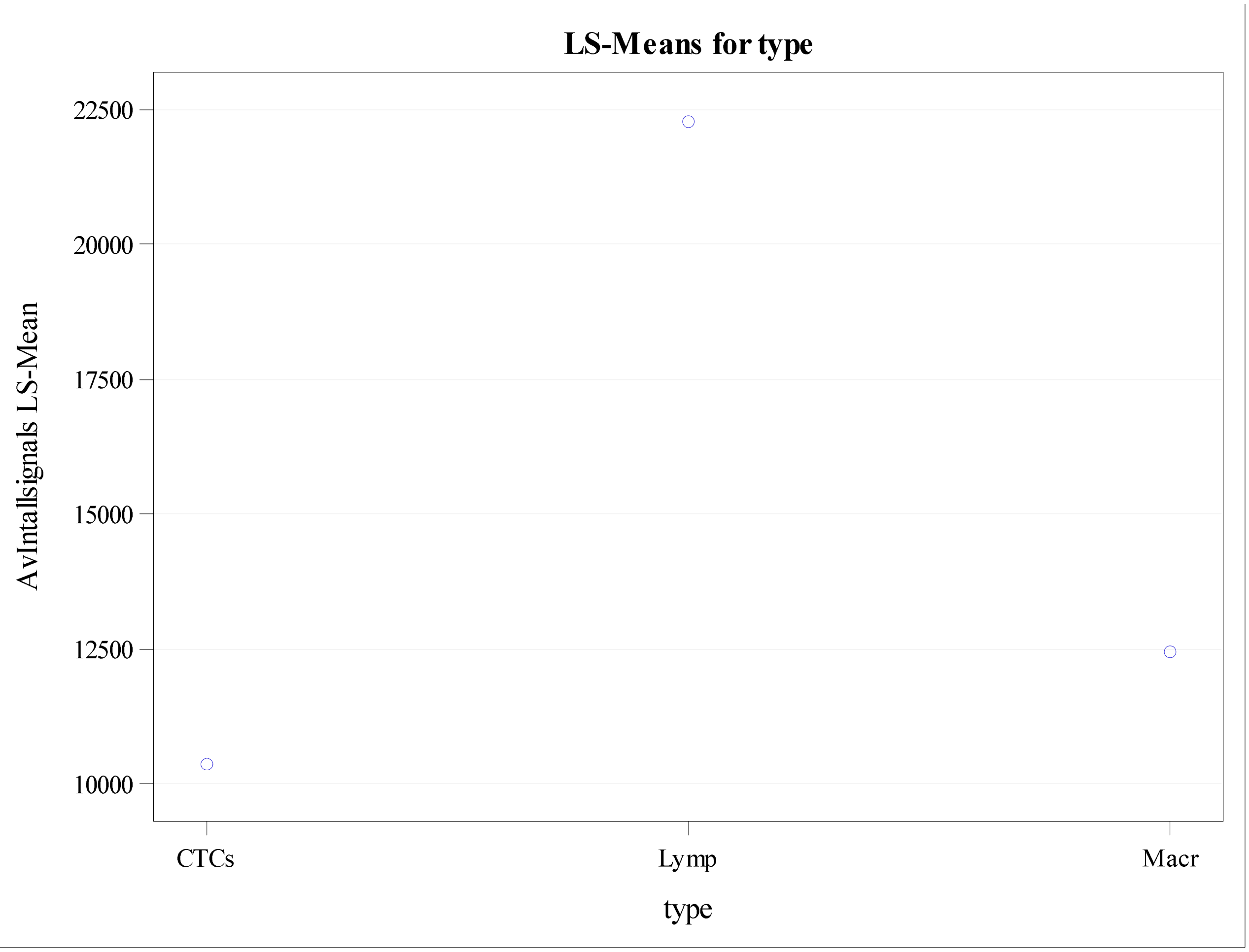

The GLM Procedure
Least Squares Means

pt=16AA7266

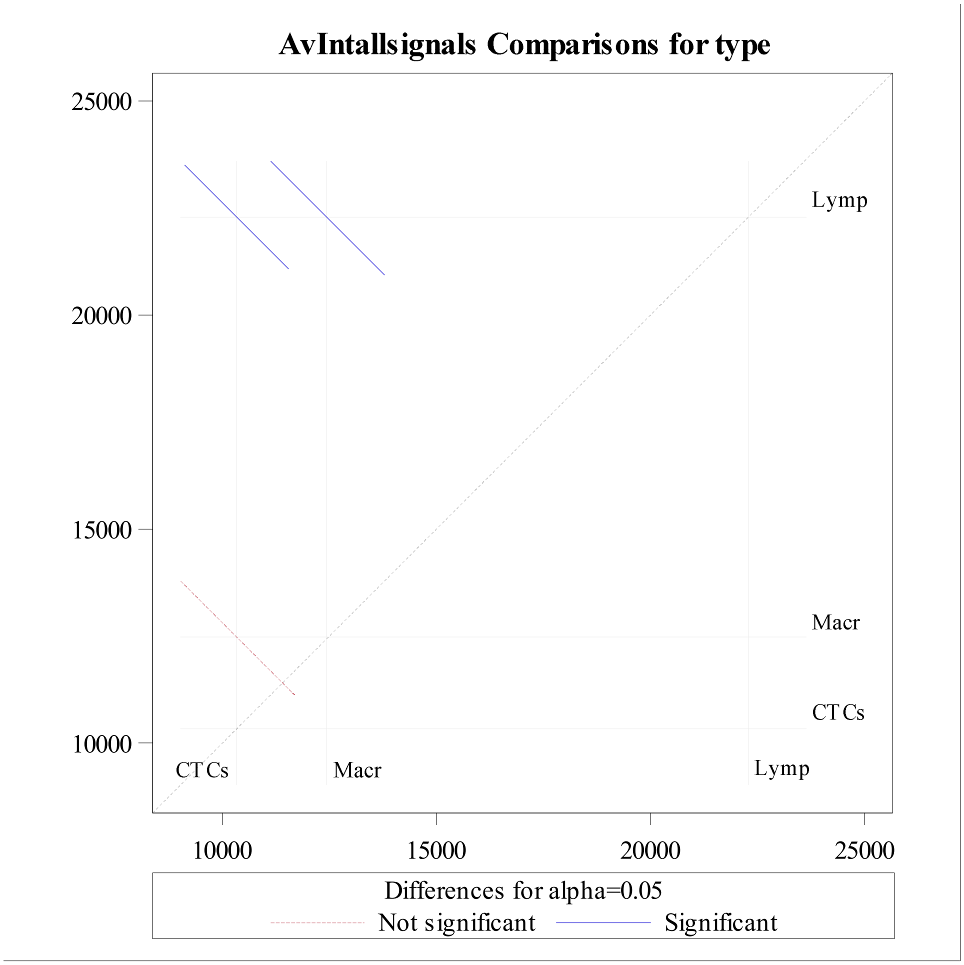

type	Totalintensity LSMEAN	Standard Error	Pr > |t|	LSMEAN Number
**CTCs**	380375.419	32880.735	<.0001	1
**Lymp**	511158.500	33424.255	<.0001	2
**Macr**	230204.591	39031.121	<.0001	3

Least Squares Means for effect type<br>Pr > |t| for H0: LSMean(i)=LSMean(j)<br><br>Dependent Variable: Totalintensity			
**i/j**	**1**	**2**	**3**
**1**		0.0066	0.0043
**2**	0.0066		<.0001
**3**	0.0043	<.0001	

The GLM Procedure
Least Squares Means

pt=16AA7266

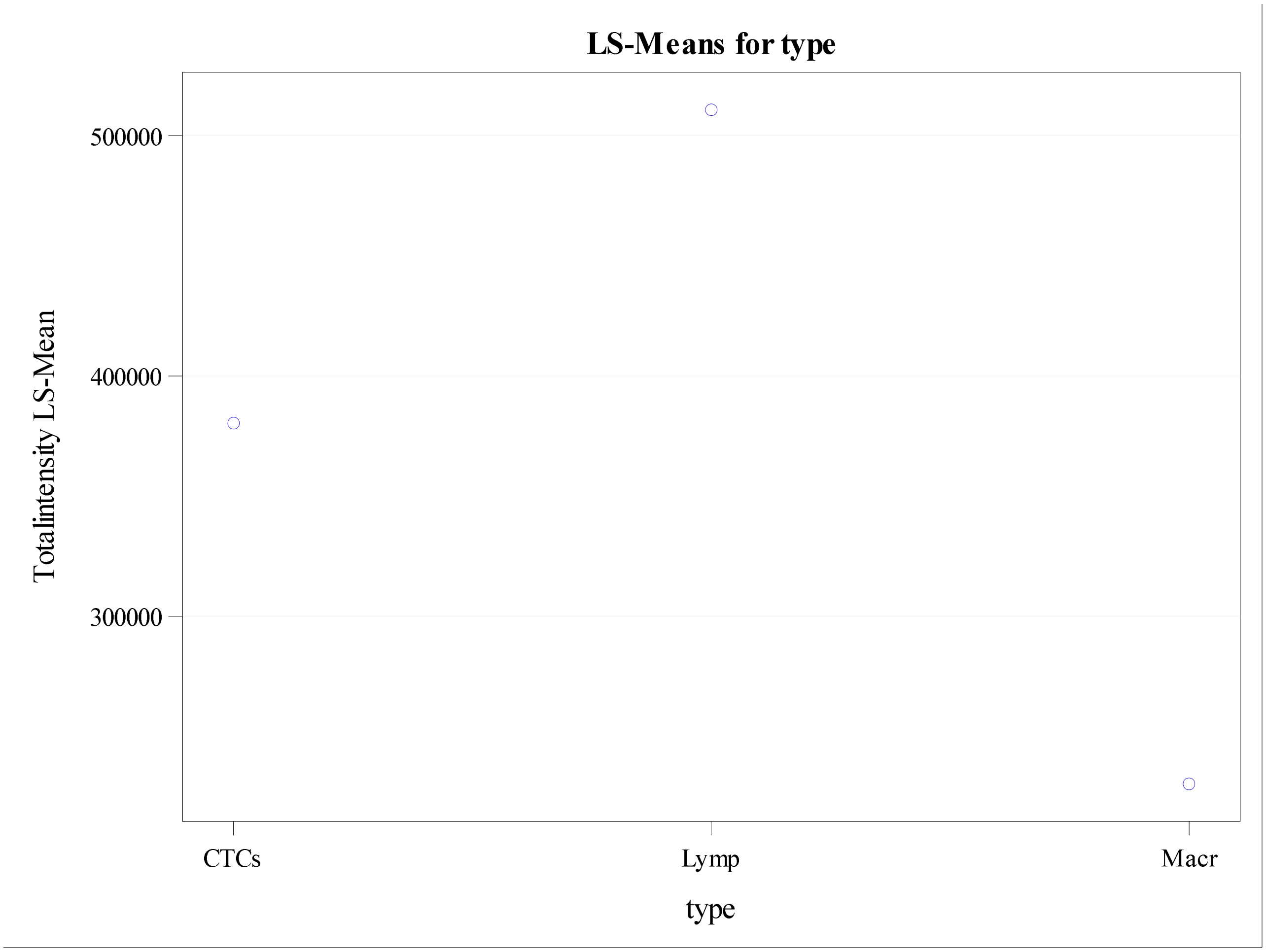

The GLM Procedure
Least Squares Means

pt=16AA7266

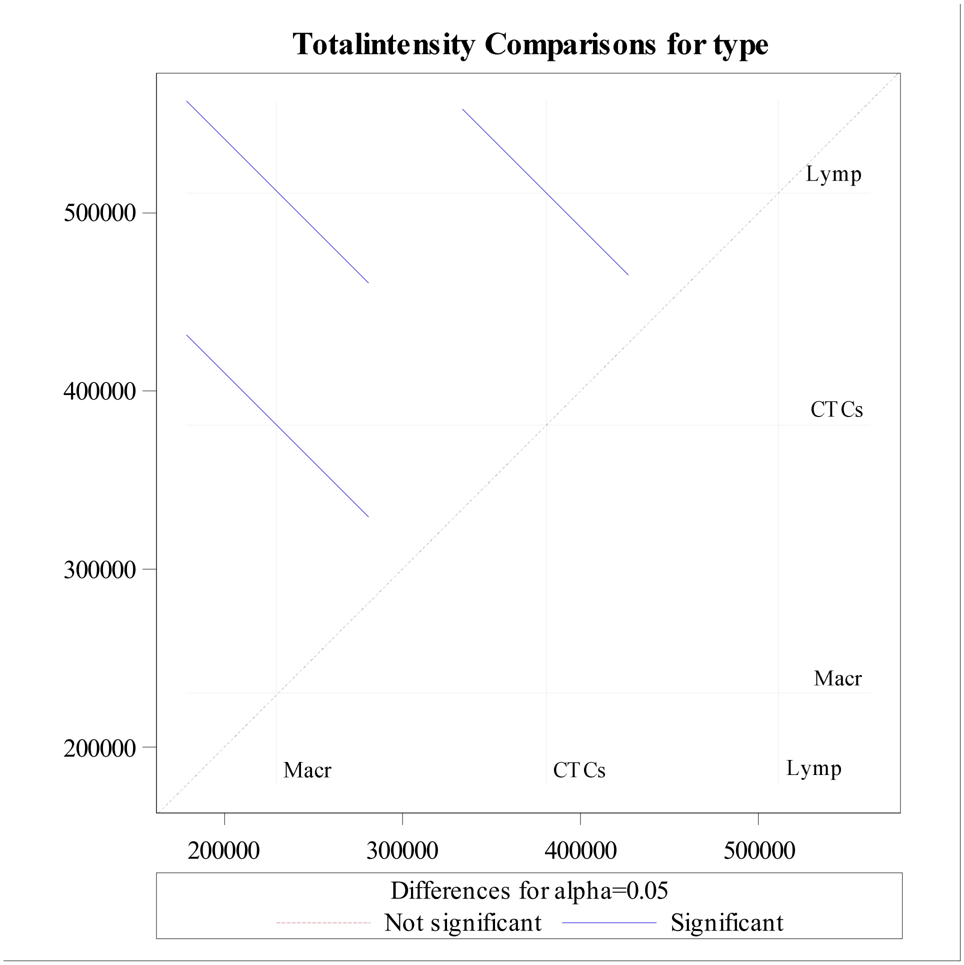

type	Nuclearvolume LSMEAN	Standard Error	Pr > |t|	LSMEAN Number
**CTCs**	915436.161	43815.169	<.0001	1
**Lymp**	154573.967	44539.436	0.0008	2
**Macr**	565345.727	52010.856	<.0001	3

Least Squares Means for effect type<br>Pr > |t| for H0: LSMean(i)=LSMean(j)<br><br>Dependent Variable: Nuclearvolume			
**i/j**	**1**	**2**	**3**
**1**		<.0001	<.0001
**2**	<.0001		<.0001
**3**	<.0001	[illegible]	

The GLM Procedure
Least Squares Means

pt=16AA7266

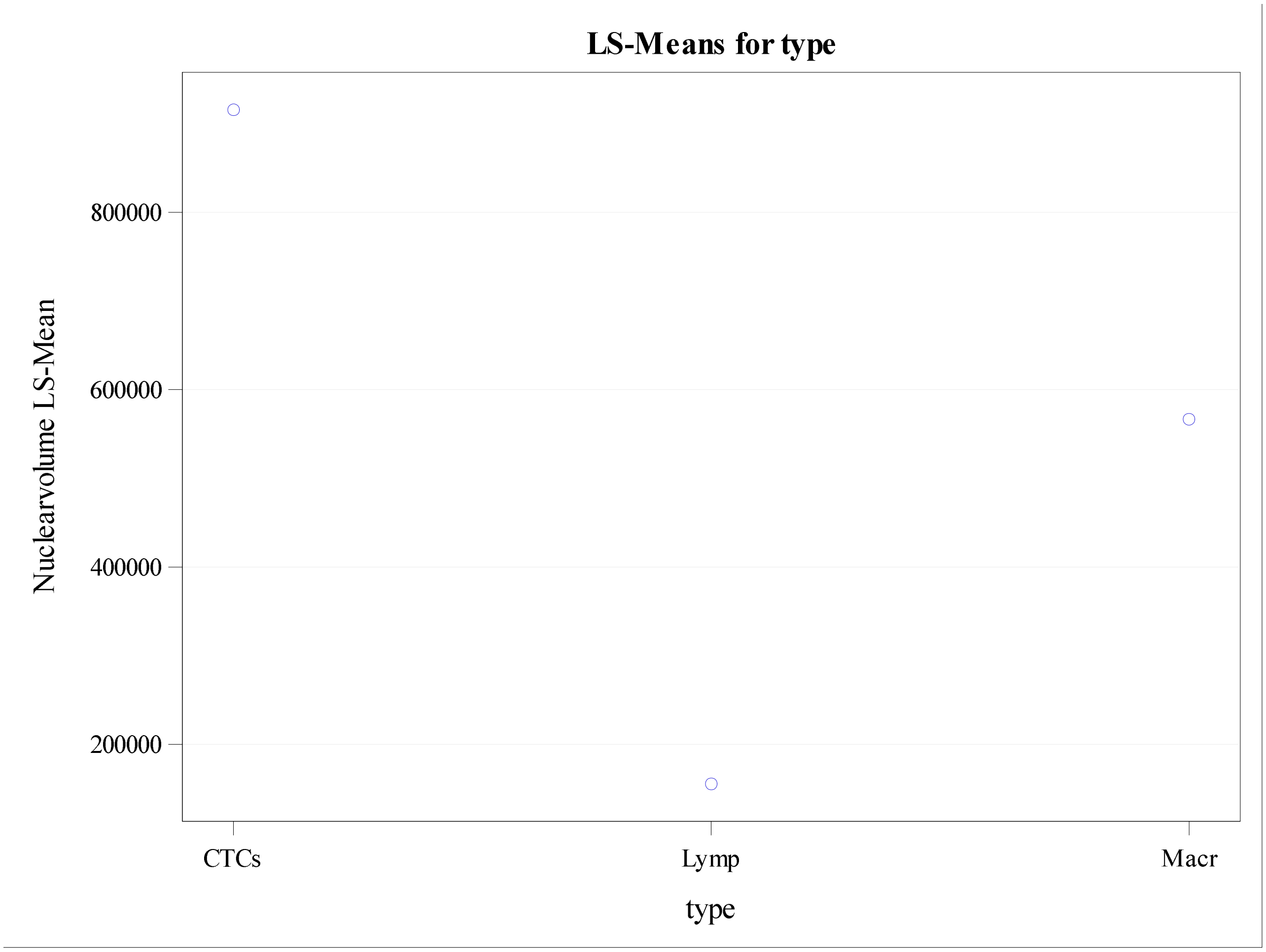

The GLM Procedure
Least Squares Means

pt=16AA7266

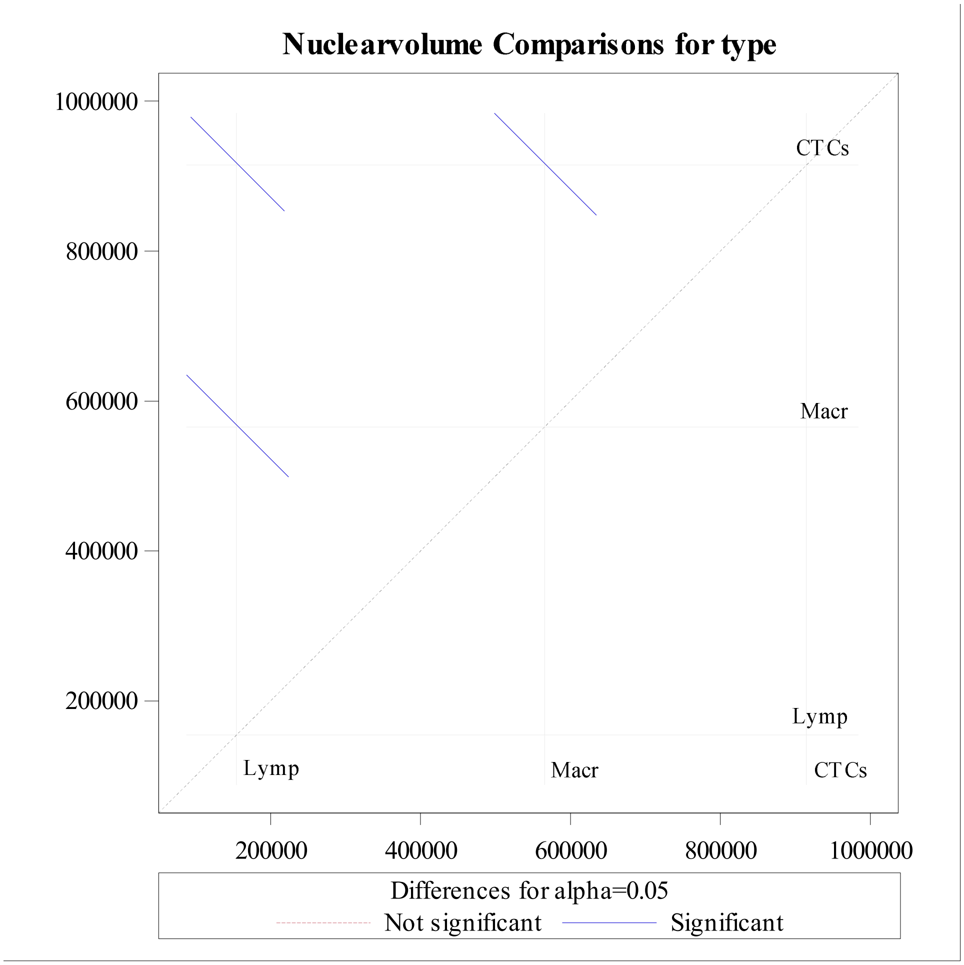

type	telomereperkvol LSMEAN	Standard Error	Pr > |t|	LSMEAN Number
**CTCs**	0.04168861	0.00405012	<.0001	1
**Lymp**	0.15118778	0.00411707	<.0001	2
**Macr**	0.03421721	0.00480770	<.0001	3

Least Squares Means for effect type
Pr > |t| for H0: LSMean(i)=LSMean(j)

Dependent Variable: telomereperkvol

i/j	1	2	3
**1**		<.0001	0.2381
**2**	<.0001		<.0001
**3**	0.2381	<.0001	

The GLM Procedure
Least Squares Means

pt=16AA7266

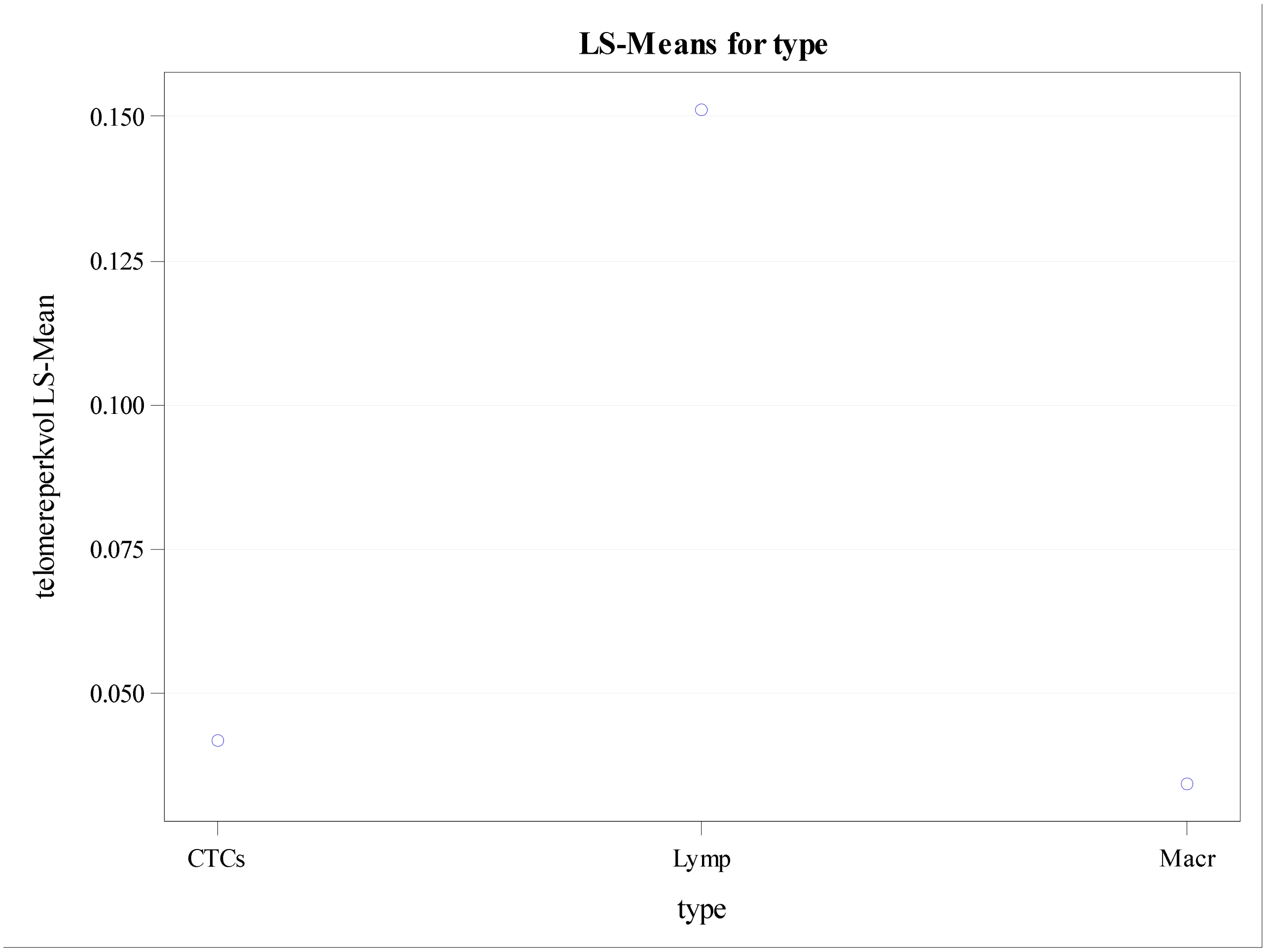

The GLM Procedure
Least Squares Means

pt=16AA7266

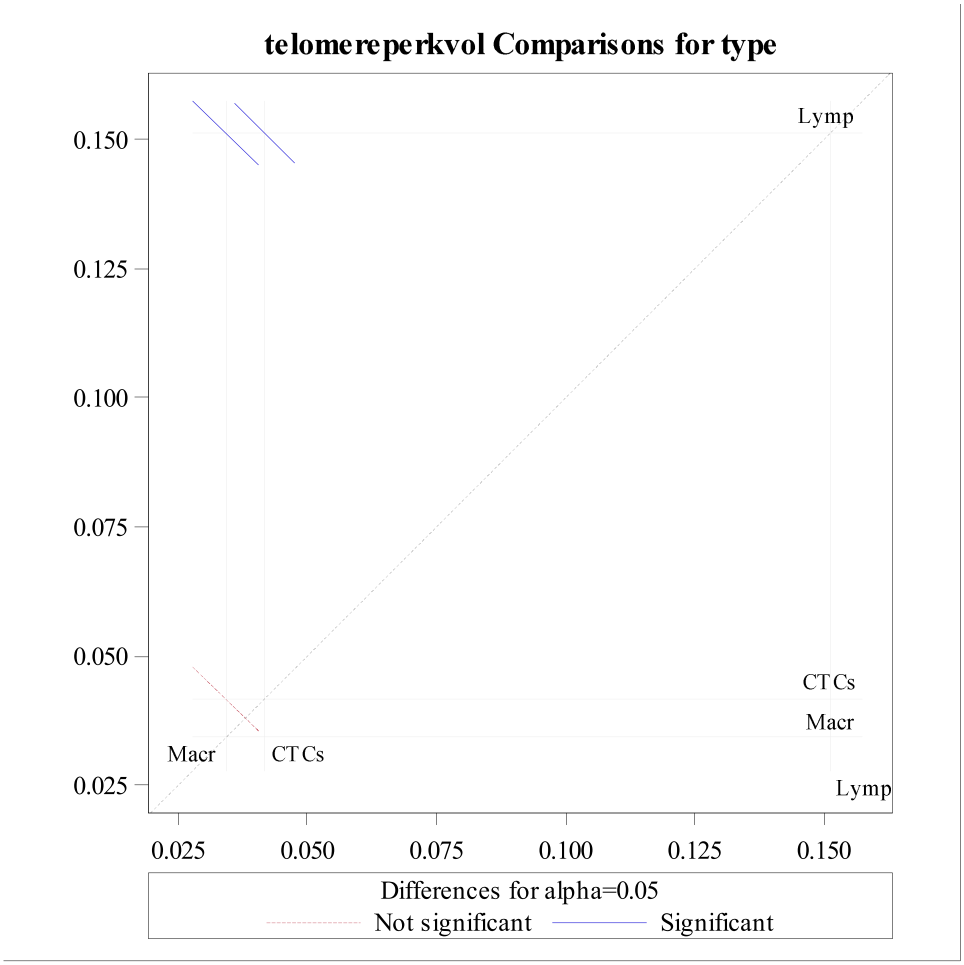

Note: To ensure overall protection level, only probabilities associated with pre-planned comparisons should be used.

The GLM Procedure

pt=16AA7280

Class Level Information		
**Class**	**Levels**	**Values**
**type**	3	CTCs Lymp Macr

**Number of Observations Read**	53
**Number of Observations Used**	53

The GLM Procedure

Dependent Variable: Totalnofsignals
Totalnofsignals

pt=16AA7280

Source	DF	Sum of Squares	Mean Square	F Value	Pr > F
**Model**	2	223150.8243	111575.4121	27.31	<.0001
**Error**	50	204277.8927	4085.5579		
**Corrected Total**	52	427428.7170			

R-Square	Coeff Var	Root MSE	Totalnofsignals Mean
0.522077	65.08499	63.91837	98.20755

Source	DF	Type III SS	Mean Square	F Value	Pr > F
**type**	2	223150.8243	111575.4121	27.31	<.0001

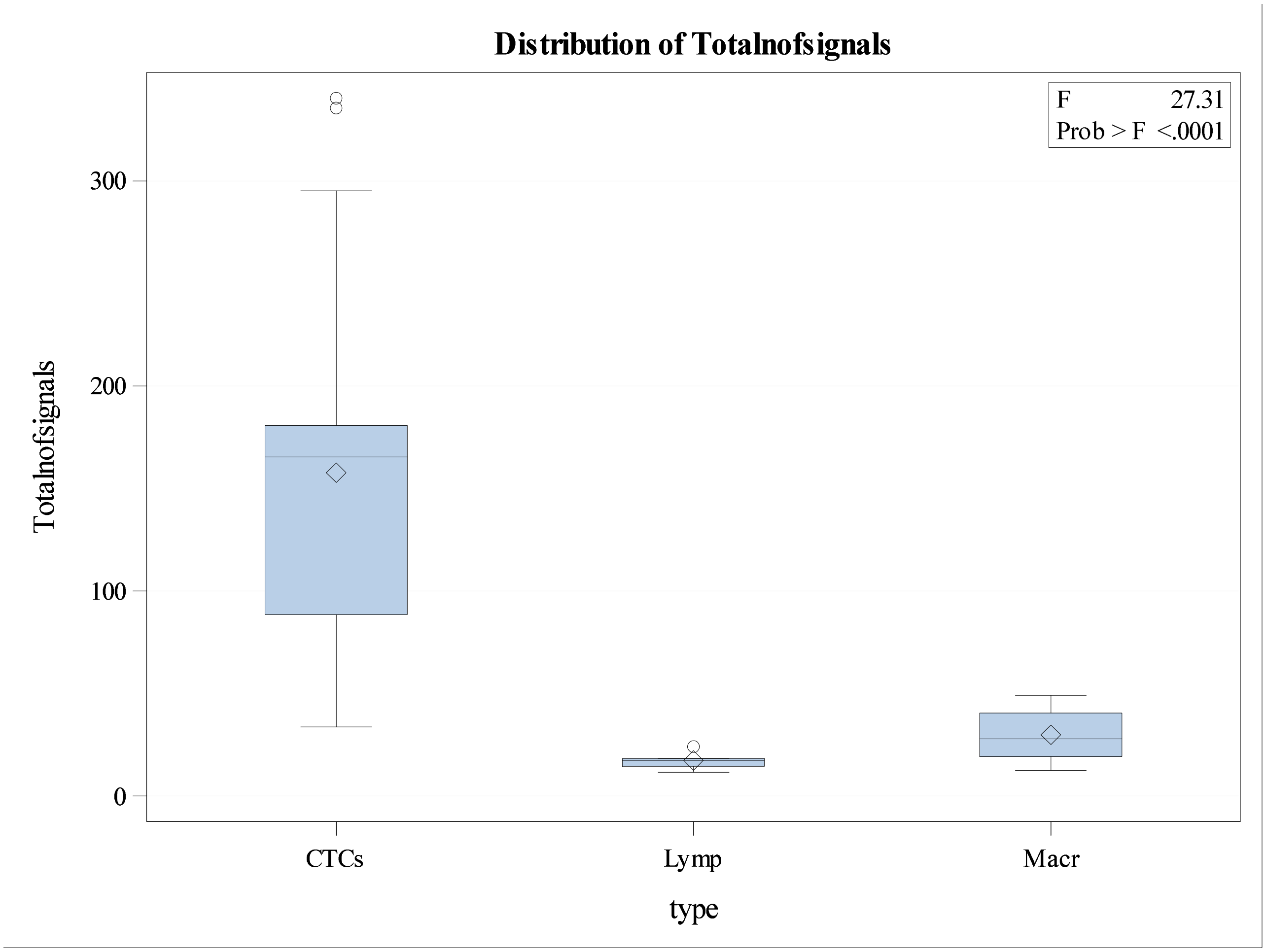

The GLM Procedure

Dependent Variable: Totalnofaggregates Totalnofaggregates

pt=16AA7280

Source	DF	Sum of Squares	Mean Square	F Value	Pr > F
**Model**	2	3894.543628	1947.271814	22.63	<.0001
**Error**	50	4301.984674	86.039693		
**Corrected Total**	52	8196.528302			

R-Square	Coeff Var	Root MSE	Totalnofaggregates Mean
0.475146	77.91049	9.275758	11.90566

Source	DF	Type III SS	Mean Square	F Value	Pr > F
**type**	2	3894.543628	1947.271814	22.63	<.0001

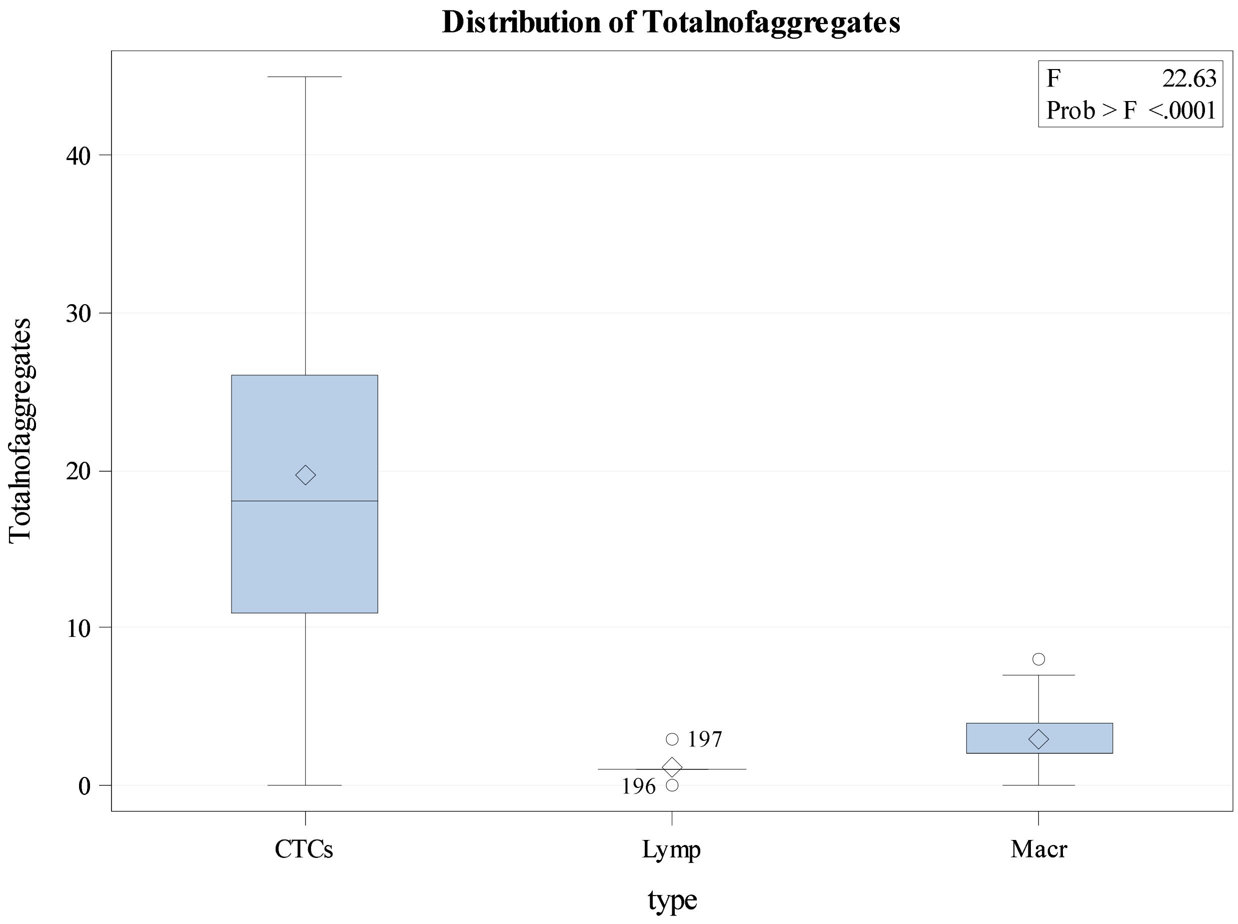

The GLM Procedure

Dependent Variable: acratio
acratio

pt=16AA7280

Source	DF	Sum of Squares	Mean Square	F Value	Pr > F
**Model**	2	36.0506358	18.0253179	5.30	0.0082
**Error**	50	170.1420984	3.4028420		
**Corrected Total**	52	206.1927342			

R-Square	Coeff Var	Root MSE	acratio Mean
0.174840	59.68891	1.844679	3.090489

Source	DF	Type III SS	Mean Square	F Value	Pr > F
**type**	2	36.05063580	18.02531790	5.30	0.0082

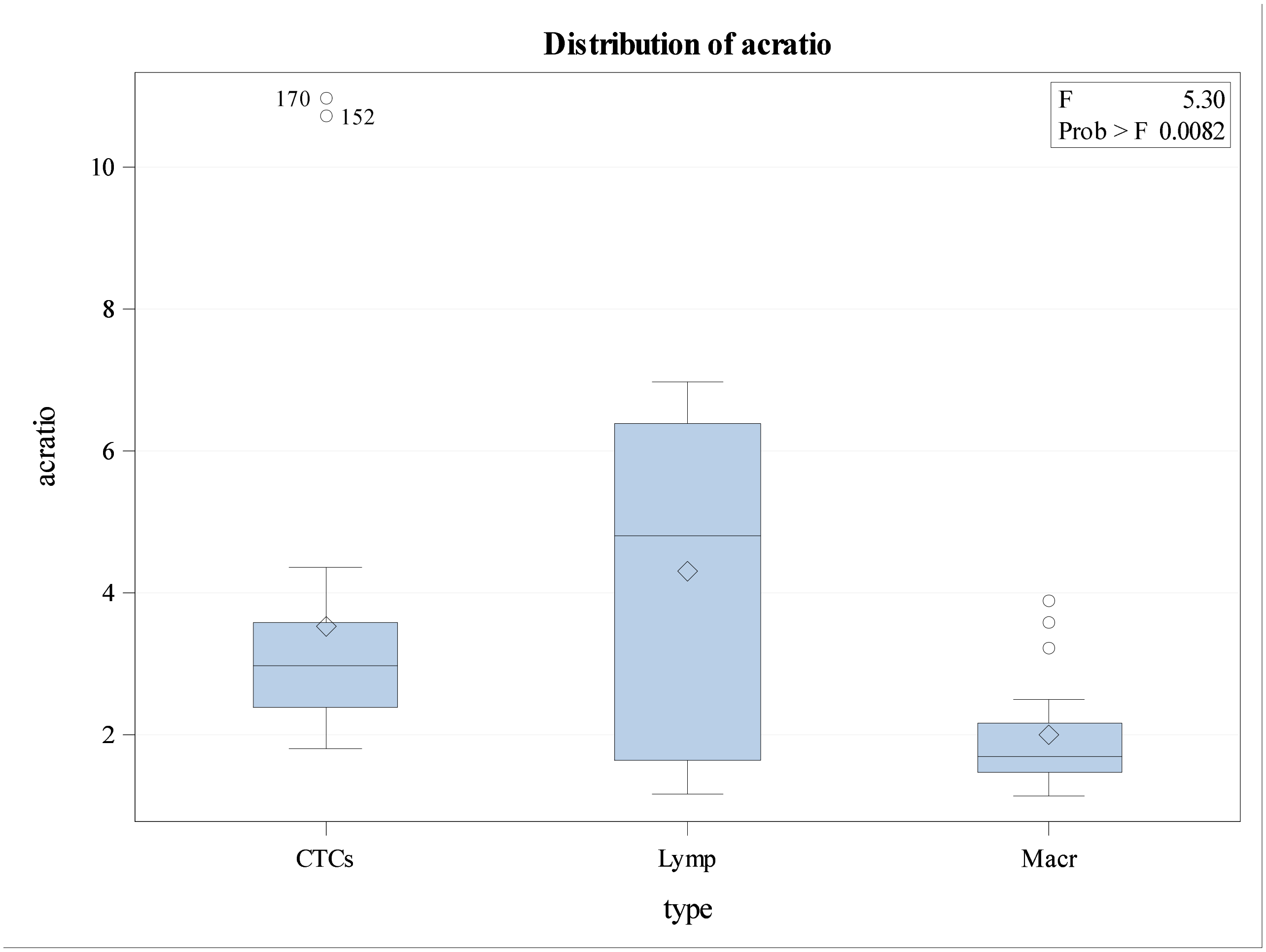

The GLM Procedure

Dependent Variable: AvIntallsignals
AvIntallsignals

pt=16AA7280

Source	DF	Sum of Squares	Mean Square	F Value	Pr > F
Model	2	140068458.0	70034229.0	7.09	0.0019
Error	50	493989122.5	9879782.4		
Corrected Total	52	634057580.5			

R-Square	Coeff Var	Root MSE	AvIntallsignals Mean
0.220908	28.48851	3143.212	11033.26

Source	DF	Type III SS	Mean Square	F Value	Pr > F
type	2	140068458.0	70034229.0	7.09	0.0019

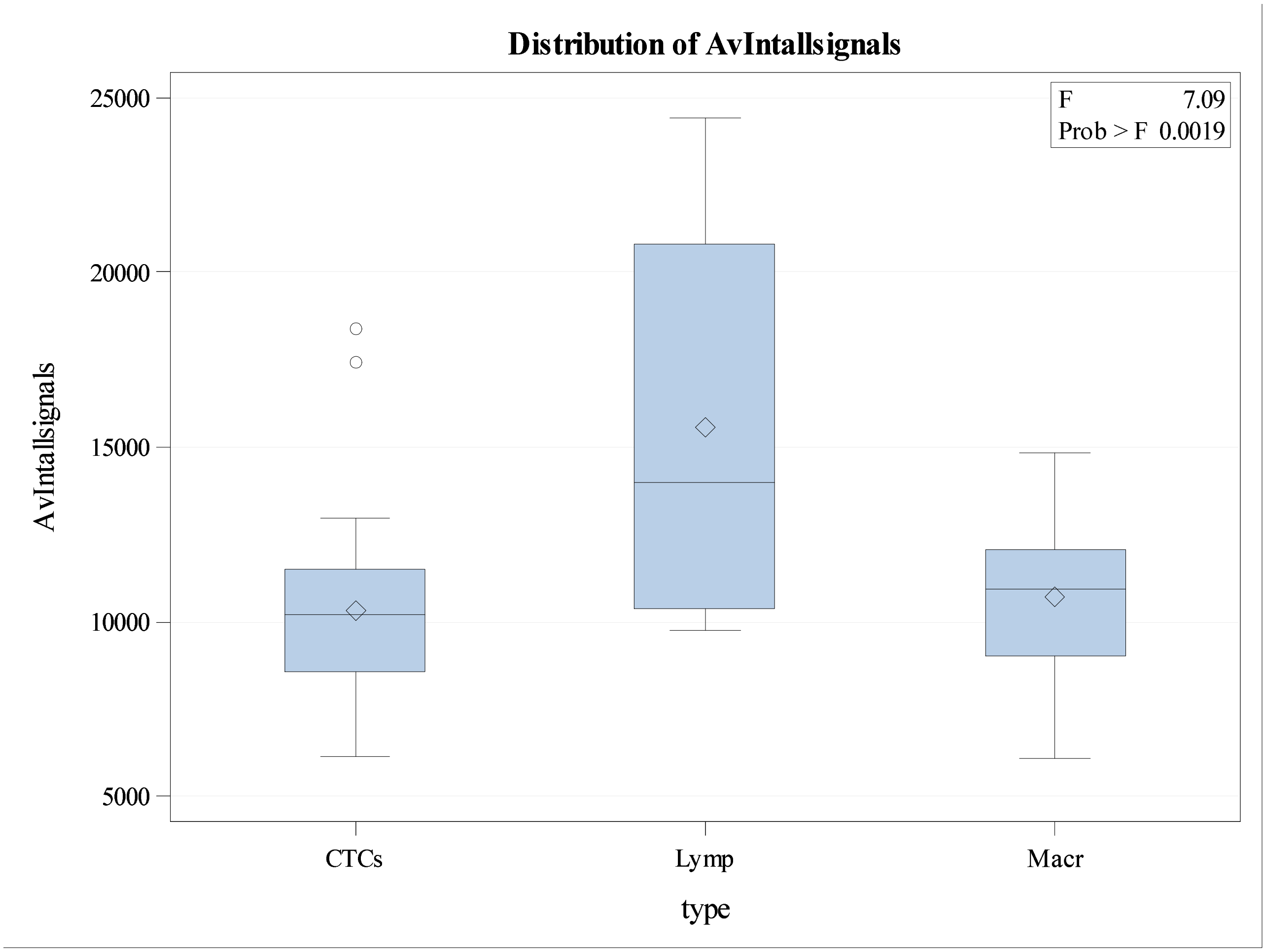

The GLM Procedure

Dependent Variable: Totalintensity
Totalintensity

pt=16AA7280

Source	DF	Sum of Squares	Mean Square	F Value	Pr > F
**Model**	2	2.0086564E13	1.0043282E13	24.20	<.0001
**Error**	50	2.075341E13	415068207694		
**Corrected Total**	52	4.0839974E13			

R-Square	Coeff Var	Root MSE	Totalintensity Mean
0.491836	65.26882	644257.9	987083.7

Source	DF	Type III SS	Mean Square	F Value	Pr > F
**type**	2	2.0086564E13	1.0043282E13	24.20	<.0001

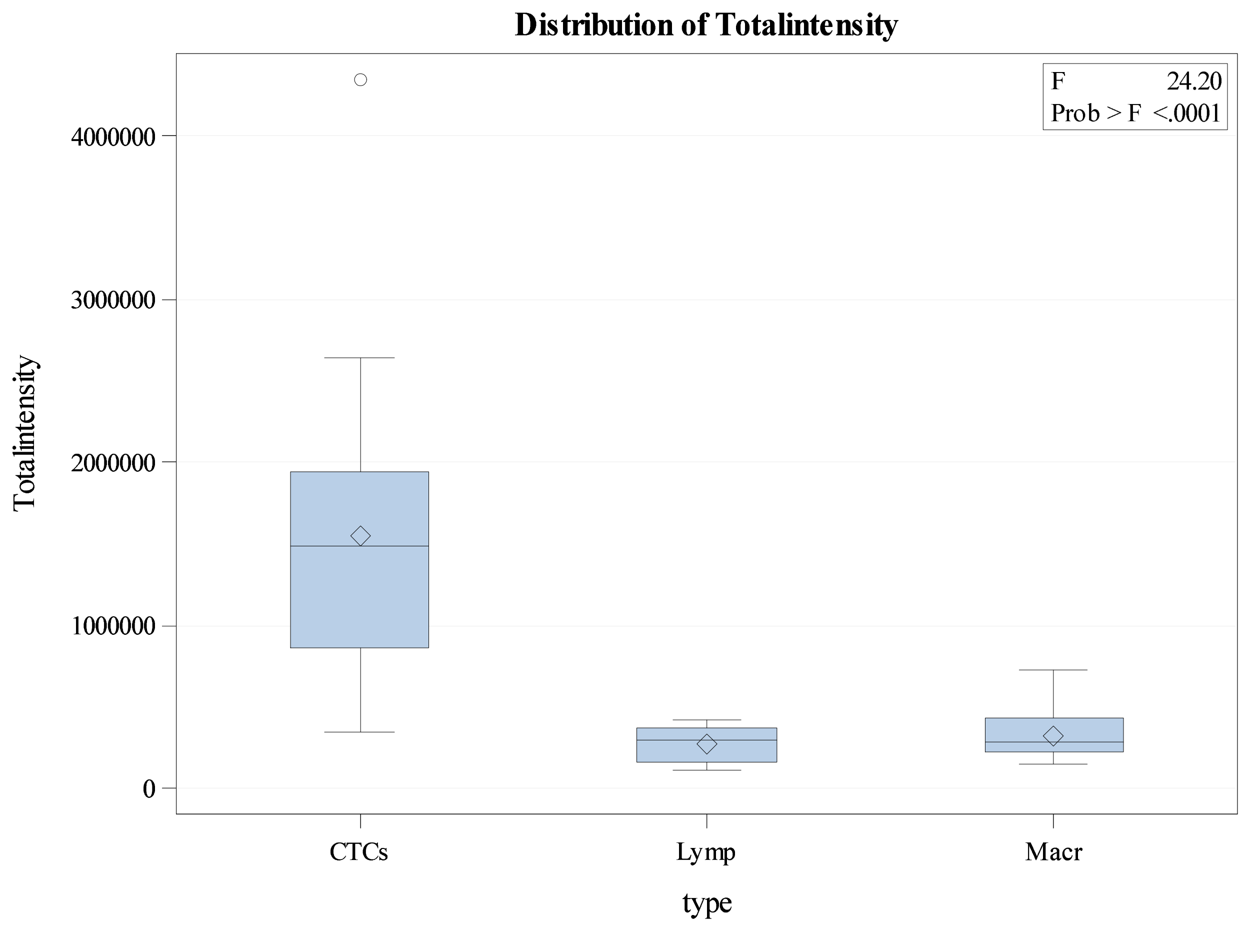

The GLM Procedure

Dependent Variable: Nuclearvolume Nuclearvolume

pt=16AA7280

Source	DF	Sum of Squares	Mean Square	F Value	Pr > F
**Model**	2	1.2415263E13	6.2076315E12	25.23	<.0001
**Error**	50	1.2302323E13	246046468818		
**Corrected Total**	52	2.4717586E13			

R-Square	Coeff Var	Root MSE	Nuclearvolume Mean
0.502285	58.88673	496030.7	842347.1

Source	DF	Type III SS	Mean Square	F Value	Pr > F
**type**	2	1.2415263E13	6.2076315E12	25.23	<.0001

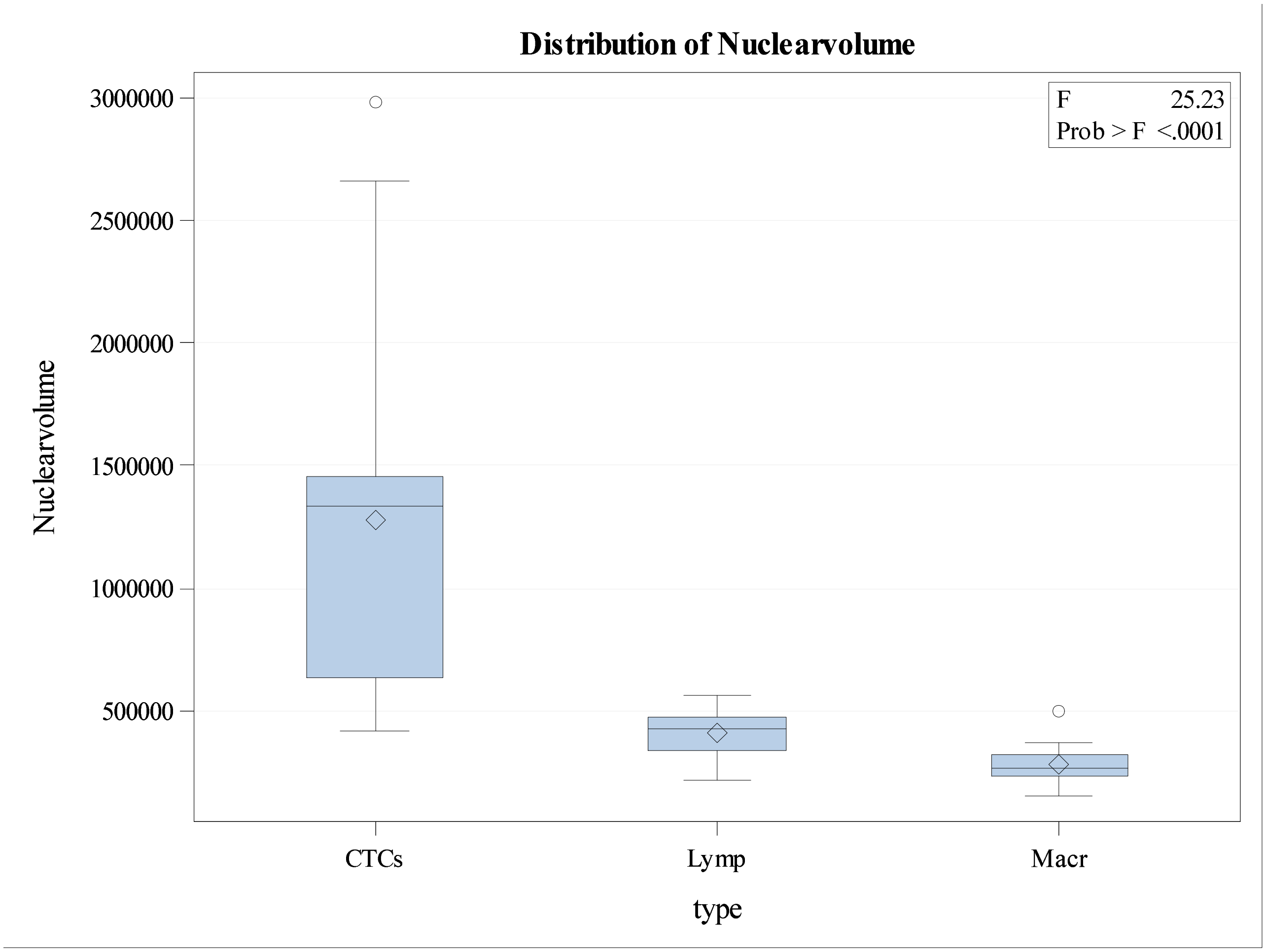

The GLM Procedure

Dependent Variable: telomereperkvol

pt=16AA7280

Source	DF	Sum of Squares	Mean Square	F Value	Pr > F
**Model**	2	0.02760431	0.01380216	14.46	<.0001
**Error**	50	0.04772167	0.00095443		
**Corrected Total**	52	0.07532598			

R-Square	Coeff Var	Root MSE	telomereperkvol Mean
0.366465	28.06963	0.030894	0.110062

Source	DF	Type III SS	Mean Square	F Value	Pr > F
**type**	2	0.02760431	0.01380216	14.46	<.0001

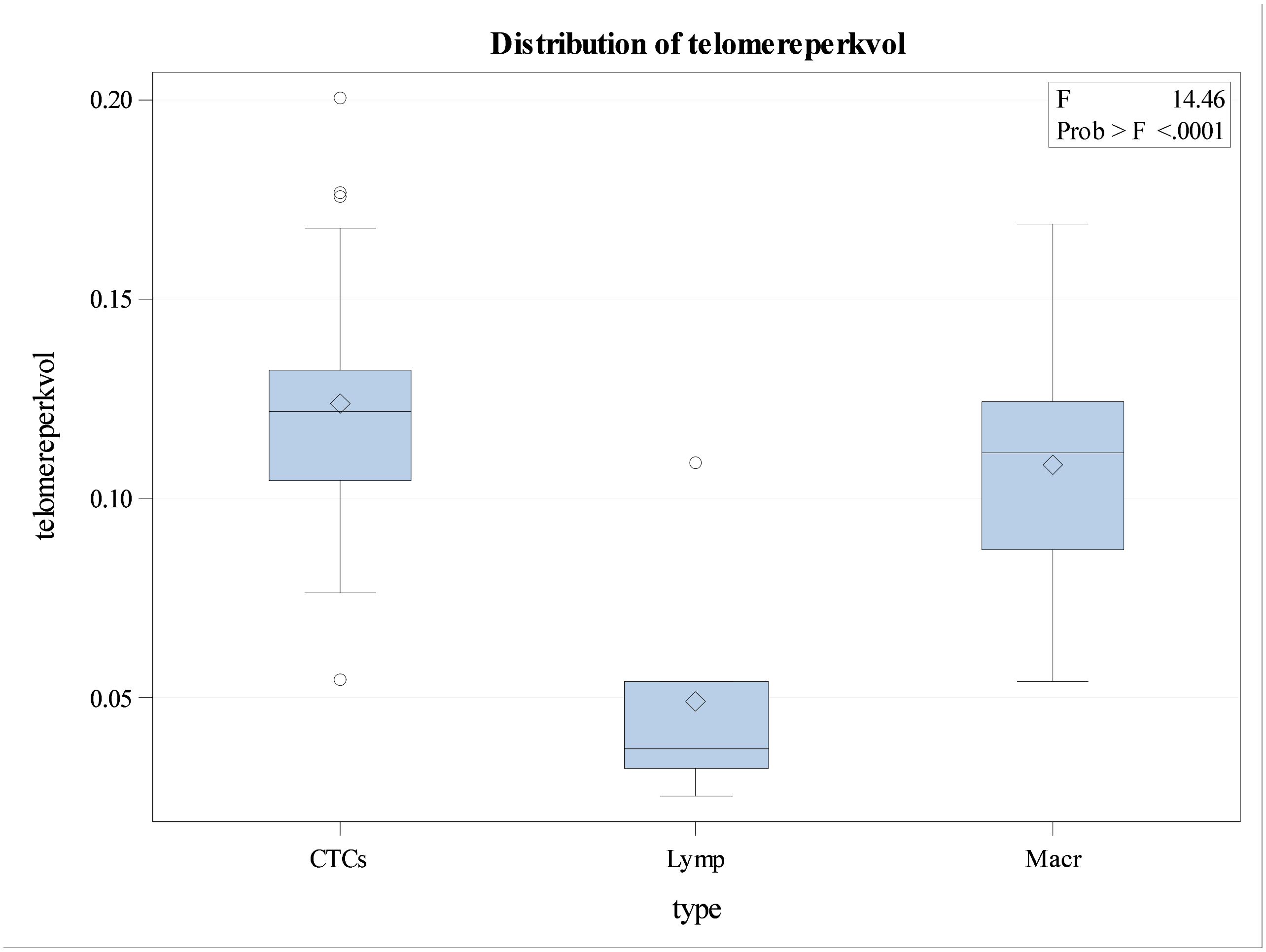

The GLM Procedure
Least Squares Means

pt=16AA7280

type	Totalnofsignals LSMEAN	Standard Error	Pr > |t|	LSMEAN Number
**CTCs**	157.137931	11.869343	<.0001	1
**Lymp**	17.333333	26.094565	0.5096	2
**Macr**	30.222222	15.065704	0.0503	3

Least Squares Means for effect type
Pr > |t| for H0: LSMean(i)=LSMean(j)

Dependent Variable: Totalnofsignals

i/j	1	2	3
**1**		<.0001	<.0001
**2**	<.0001		0.6707
**3**	<.0001	0.6707	

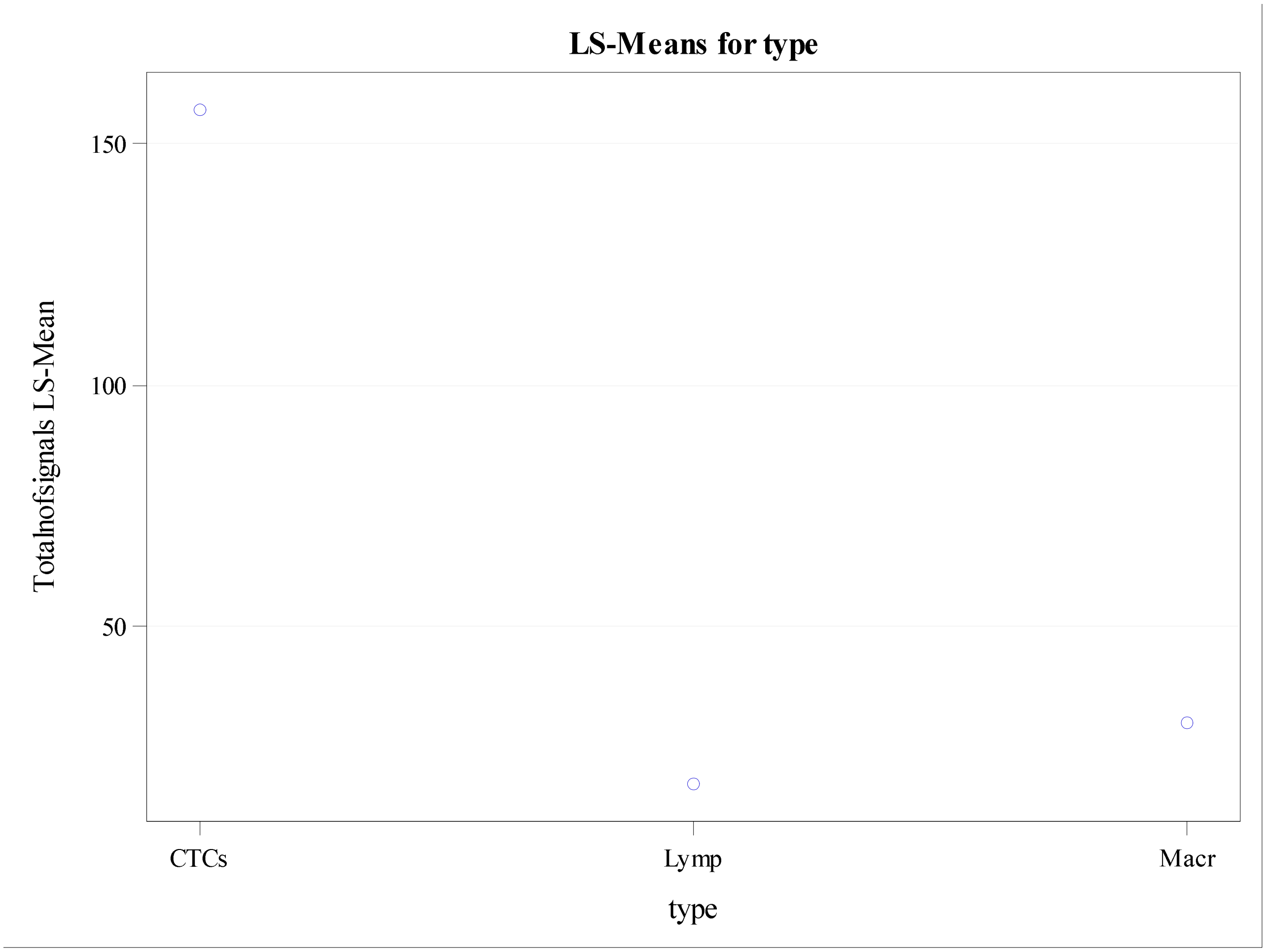

The GLM Procedure
Least Squares Means

pt=16AA7280

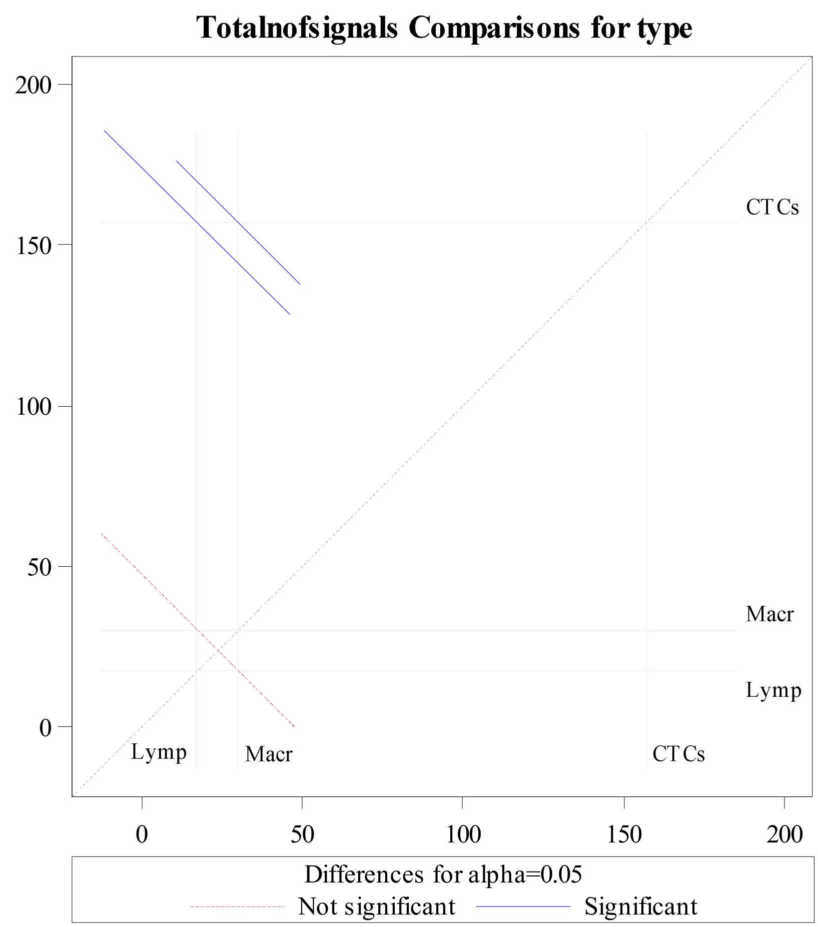

type	Totalnofaggregates LSMEAN	Standard Error	Pr > |t|	LSMEAN Number
**CTCs**	19.6896552	1.7224651	<.0001	1
**Lymp**	1.1666667	3.7868125	0.7593	2
**Macr**	2.9444444	2.1863172	0.1841	3

The GLM Procedure
Least Squares Means

pt=16AA7280

Least Squares Means for effect type<br>Pr > |t| for H0: LSMean(i)=LSMean(j)<br><br>Dependent Variable: Totalnofaggregates			
**i/j**	**1**	**2**	**3**
**1**		<.0001	<.0001
**2**	<.0001		0.6861
**3**	<.0001	0.6861	

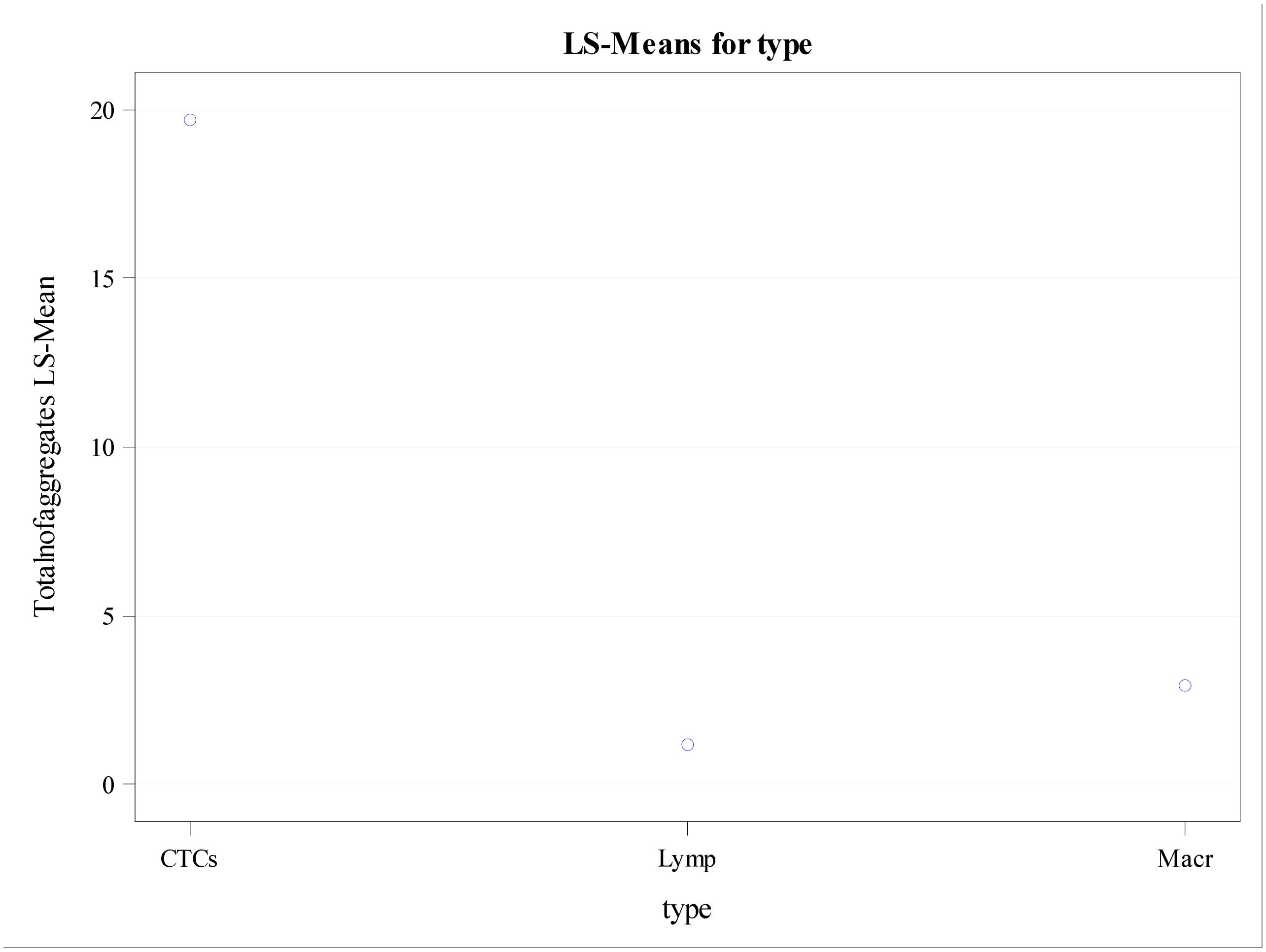

The GLM Procedure
Least Squares Means

pt=16AA7280

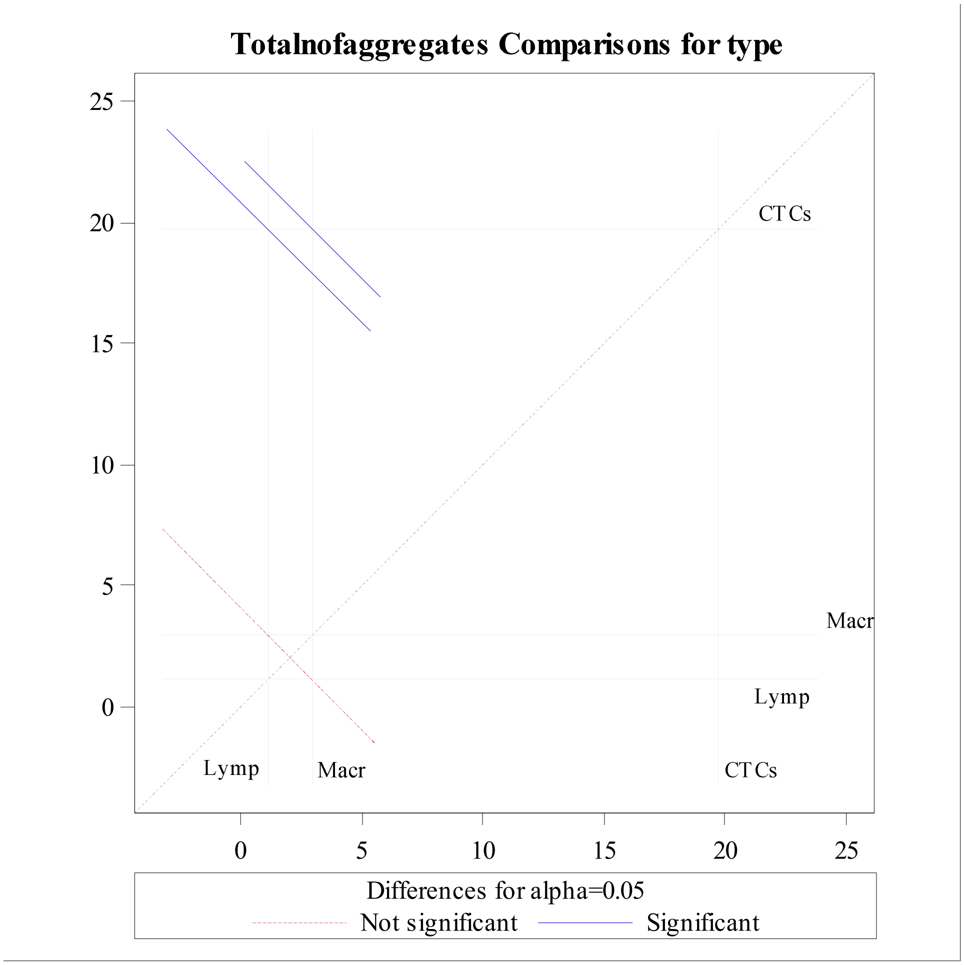

type	acratio LSMEAN	Standard Error	Pr > |t|	LSMEAN Number
**CTCs**	3.52720517	0.34254836	<.0001	1
**Lymp**	4.28893344	0.75308720	<.0001	2
**Macr**	1.98740967	0.43479510	<.0001	3

Least Squares Means for effect type
Pr > |t| for H0: LSMean(i)=LSMean(j)

Dependent Variable: acratio

i/j	1	2	3
**1**		0.3616	0.0076
**2**	0.3616		0.0108
**3**	0.0076	0.0108	

The GLM Procedure
Least Squares Means

pt=16AA7280

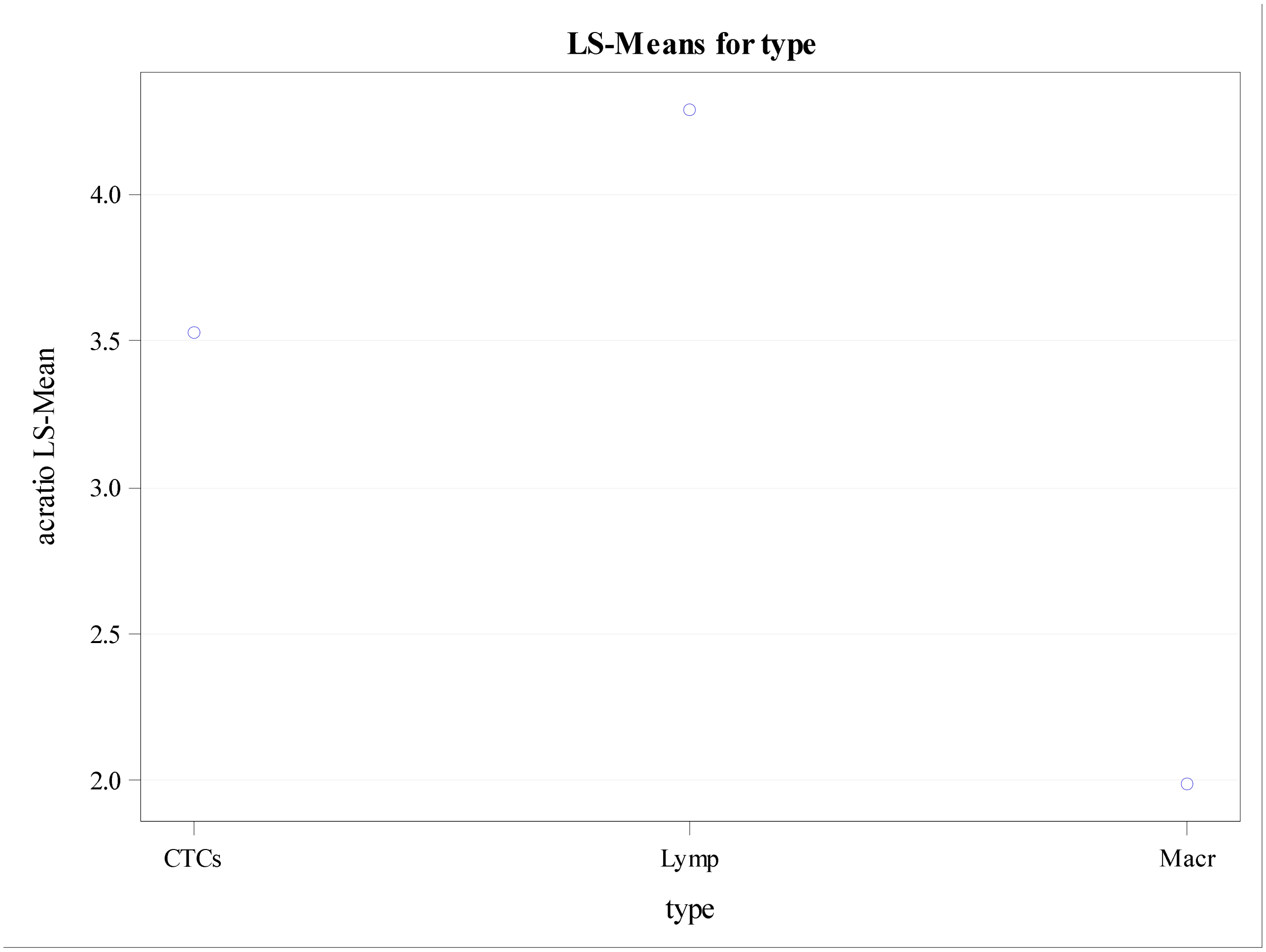

The GLM Procedure
Least Squares Means

pt=16AA7280

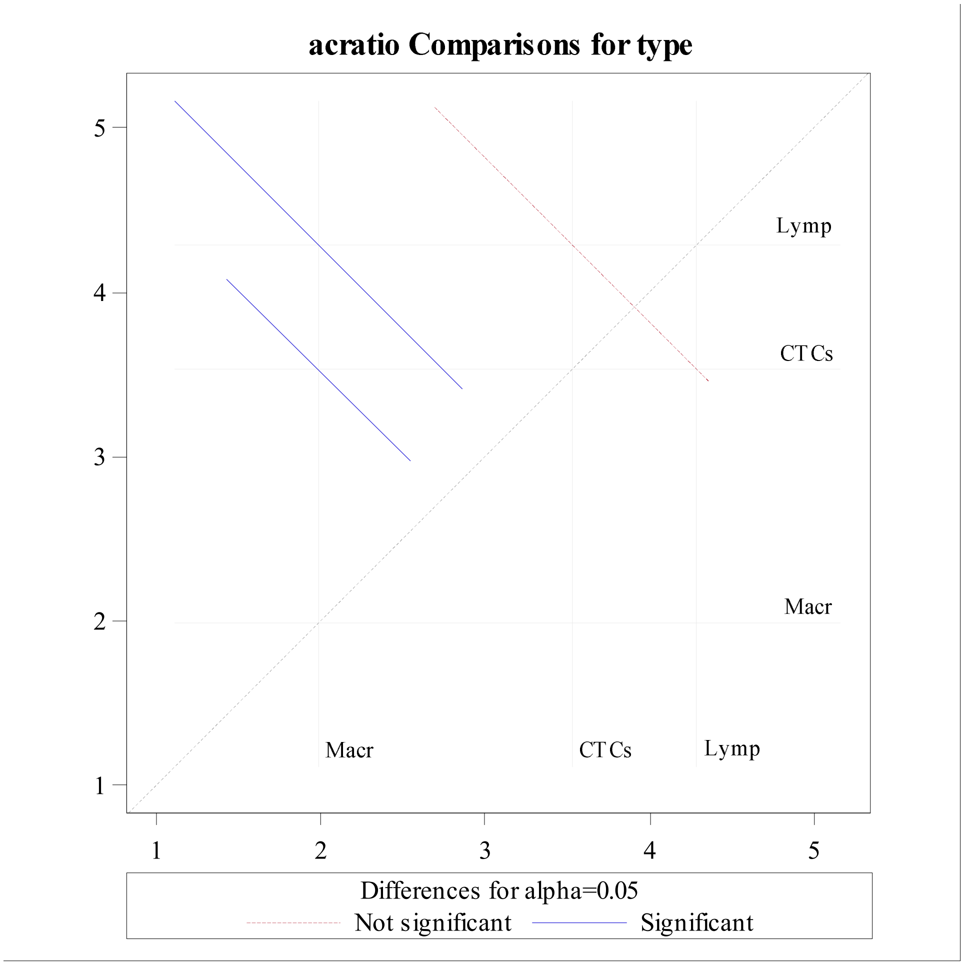

type	AvIntallsignals LSMEAN	Standard Error	Pr > |t|	LSMEAN Number
**CTCs**	10310.4806	583.6798	<.0001	1
**Lymp**	15557.1750	1283.2110	<.0001	2
**Macr**	10689.7710	740.8622	<.0001	3

Least Squares Means for effect type
Pr > |t| for H0: LSMean(i)=LSMean(j)

Dependent Variable: AvIntallsignals

i/j	1	2	3
**1**		0.0005	0.6893
**2**	0.0005		0.0019
**3**	0.6893	0.0019	

The GLM Procedure
Least Squares Means

pt=16AA7280

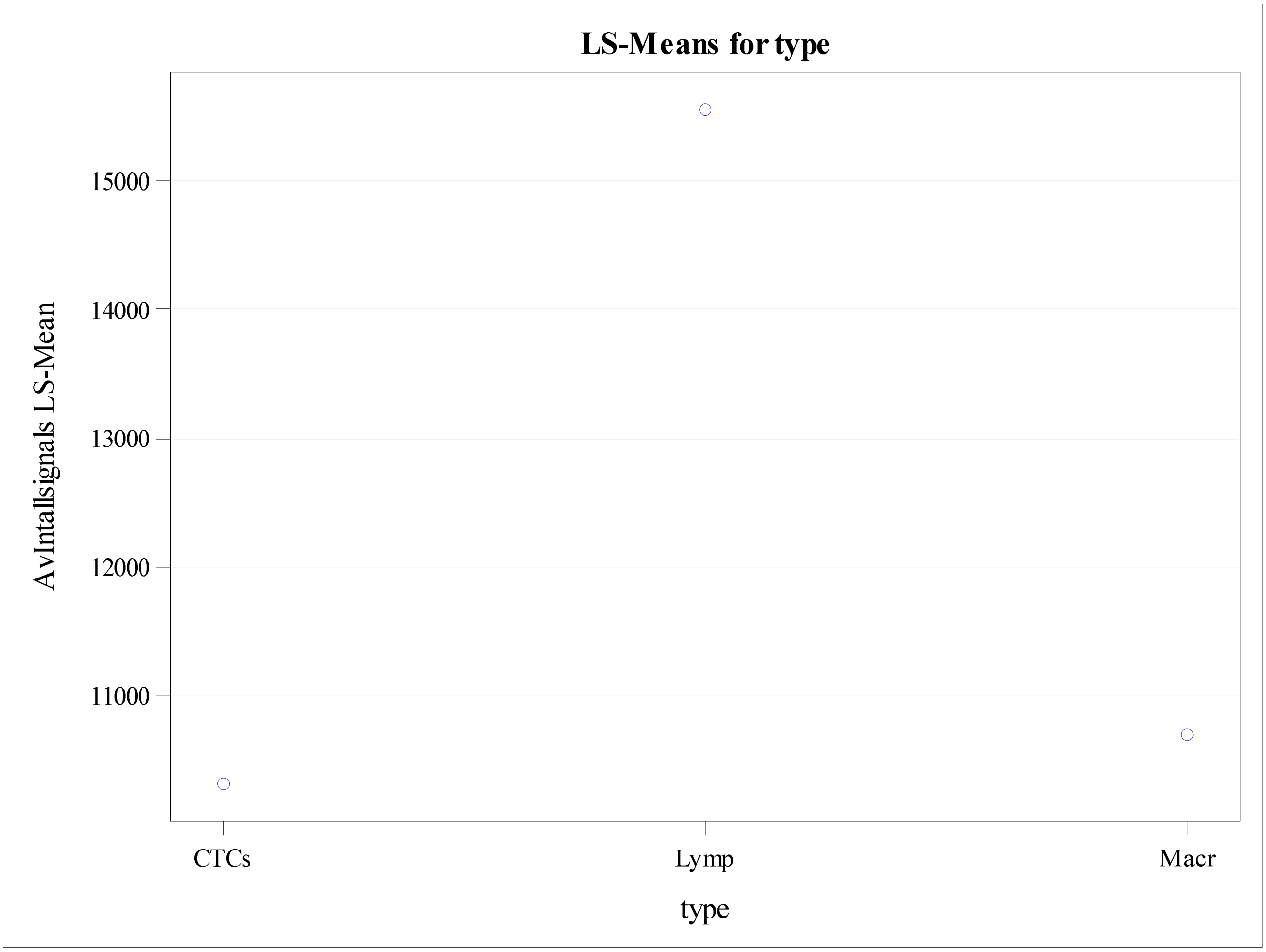

The GLM Procedure
Least Squares Means

pt=16AA7280

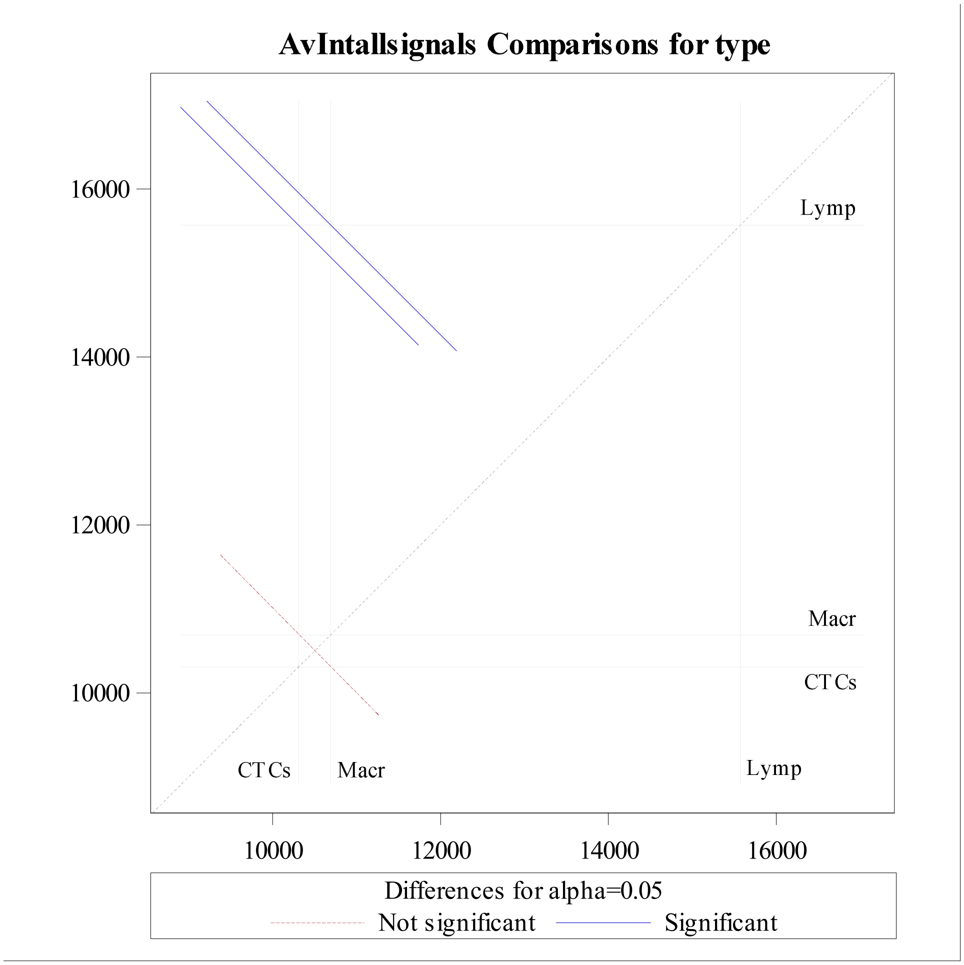

type	Totalintensity LSMEAN	Standard Error	Pr > |t|	LSMEAN Number
**CTCs**	1546991.45	119635.68	<.0001	1
**Lymp**	275688.67	263017.18	0.2996	2
**Macr**	322141.67	151853.04	0.0389	3

Least Squares Means for effect type<br>Pr > |t| for H0: LSMean(i)=LSMean(j)<br><br>Dependent Variable: Totalintensity			
**i/j**	**1**	**2**	**3**
**1**		<.0001	<.0001
**2**	<.0001		0.8791
**3**	<.0001	0.8791	

The GLM Procedure
Least Squares Means

pt=16AA7280

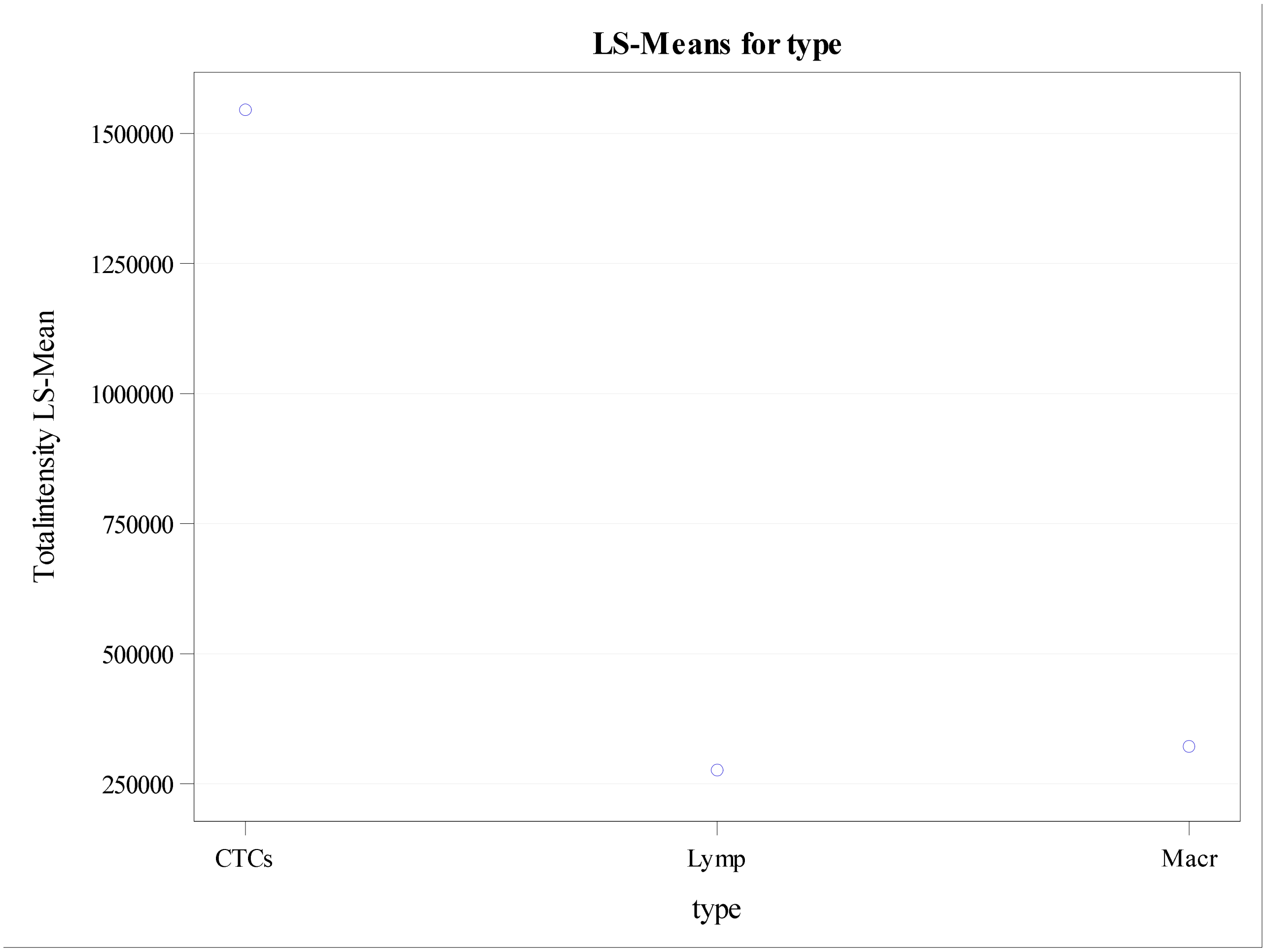

The GLM Procedure
Least Squares Means

pt=16AA7280

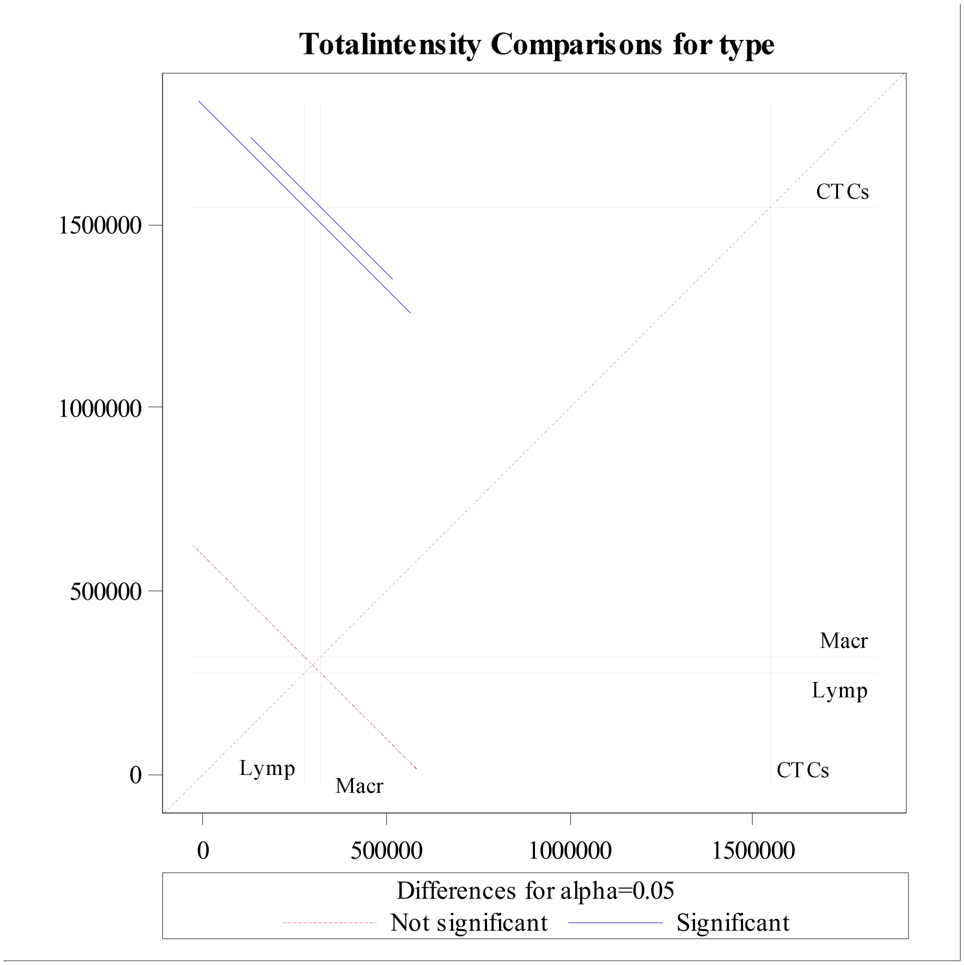

type	Nuclearvolume LSMEAN	Standard Error	Pr > |t|	LSMEAN Number
**CTCs**	1281357.52	92110.59	<.0001	1
**Lymp**	407076.67	202503.69	0.0498	2
**Macr**	280142.78	116915.56	0.0204	3

Least Squares Means for effect type<br>Pr > |t| for H0: LSMean(i)=LSMean(j)<br><br>Dependent Variable: Nuclearvolume			
**i/j**	**1**	**2**	**3**
**1**		0.0003	<.0001
**2**	0.0003		0.5896
**3**	<.0001	0.5896	

The GLM Procedure
Least Squares Means

pt=16AA7280

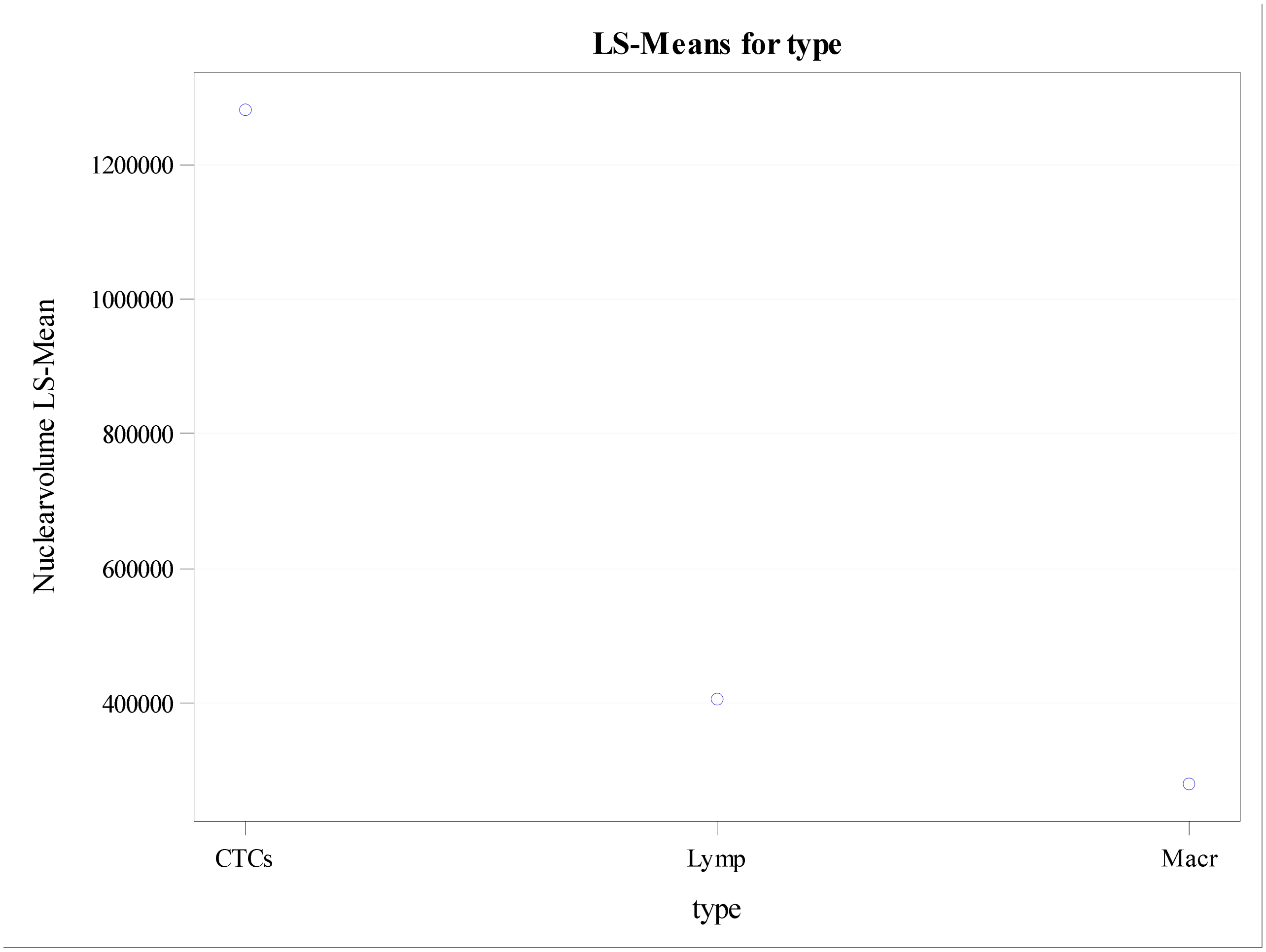

The GLM Procedure
Least Squares Means

pt=16AA7280

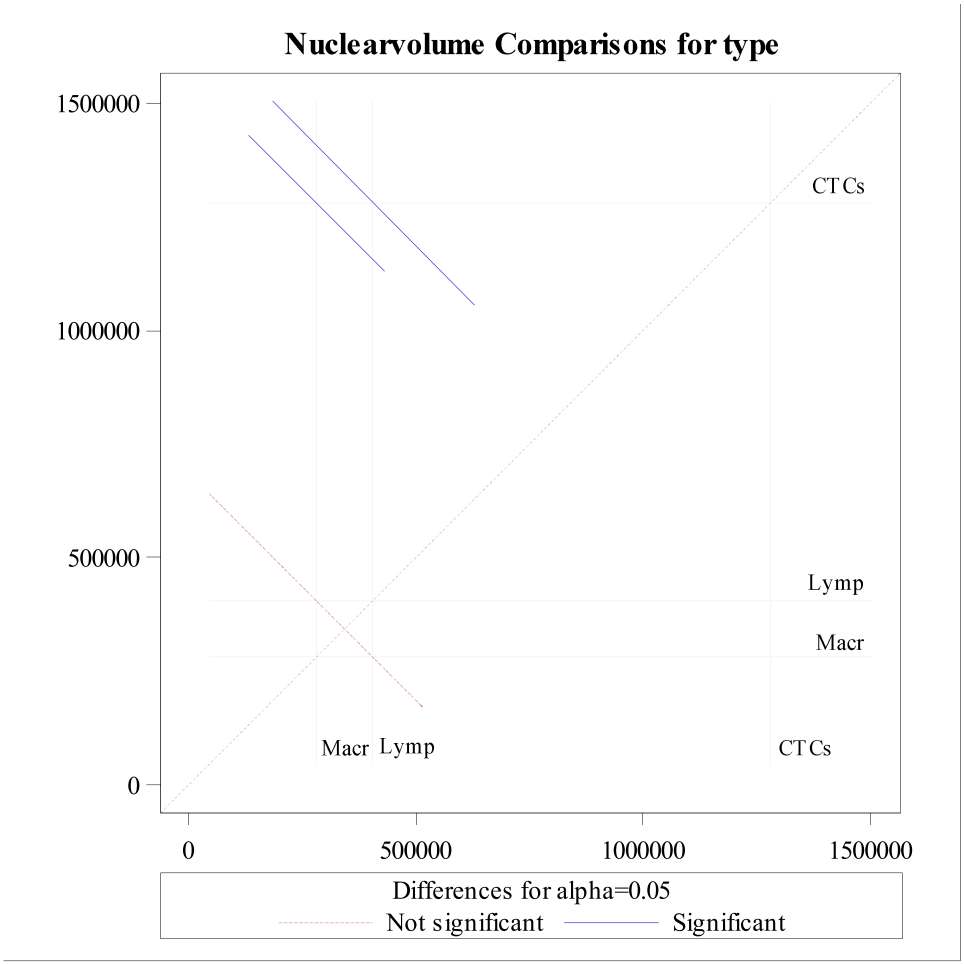

type	telomereperkvol LSMEAN	Standard Error	Pr > |t|	LSMEAN Number
**CTCs**	0.12365596	0.00573685	<.0001	1
**Lymp**	0.04923774	0.01261238	0.0003	2
**Macr**	0.10843439	0.00728176	<.0001	3

Least Squares Means for effect type<br>Pr > |t| for H0: LSMean(i)=LSMean(j)<br><br>Dependent Variable: telomereperkvol			
**i/j**	**1**	**2**	**3**
**1**		<.0001	0.1069
**2**	<.0001		0.0002
**3**	0.1069	0.0002	

The GLM Procedure
Least Squares Means

pt=16AA7280

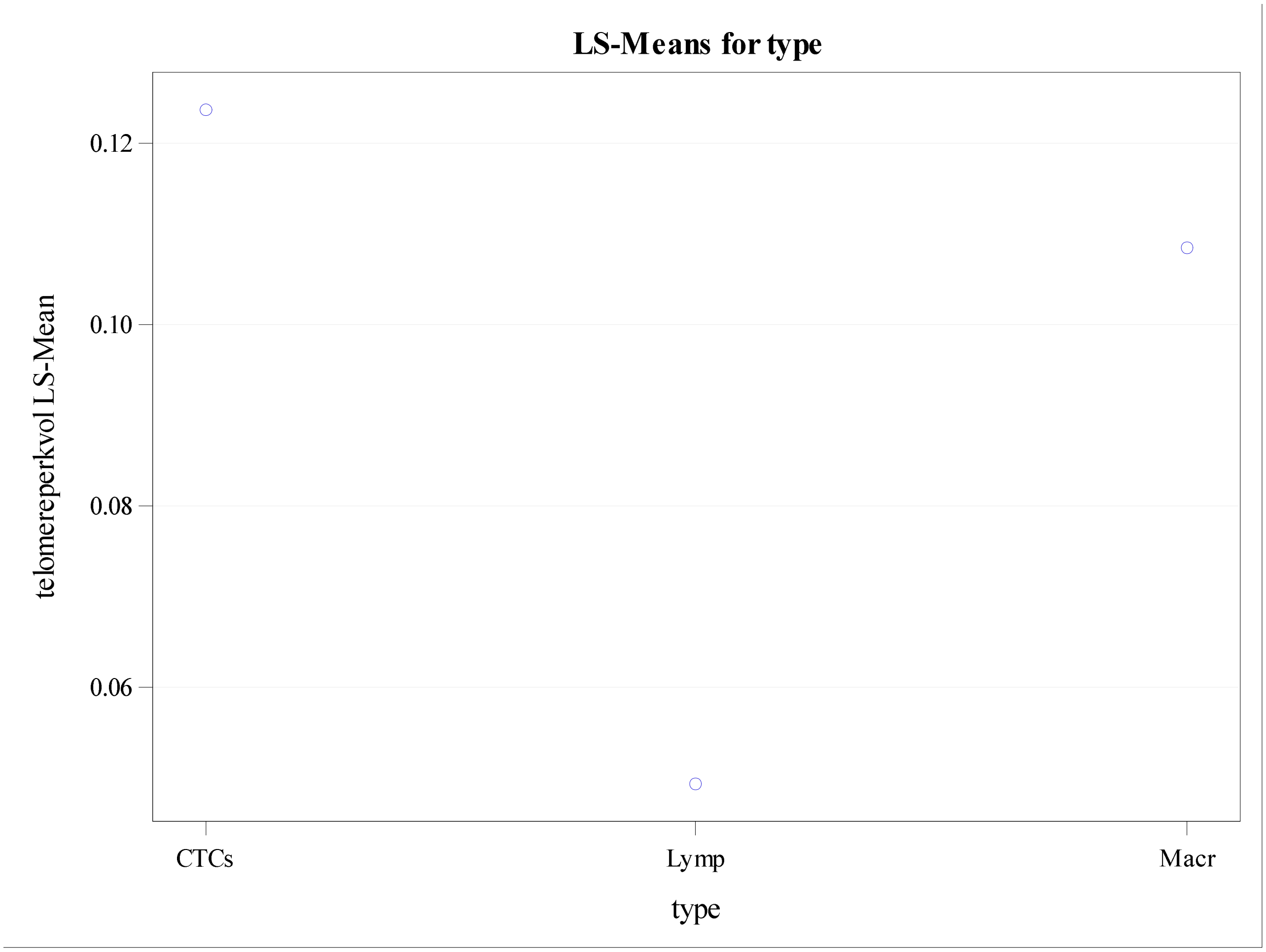

The GLM Procedure
Least Squares Means

pt=16AA7280

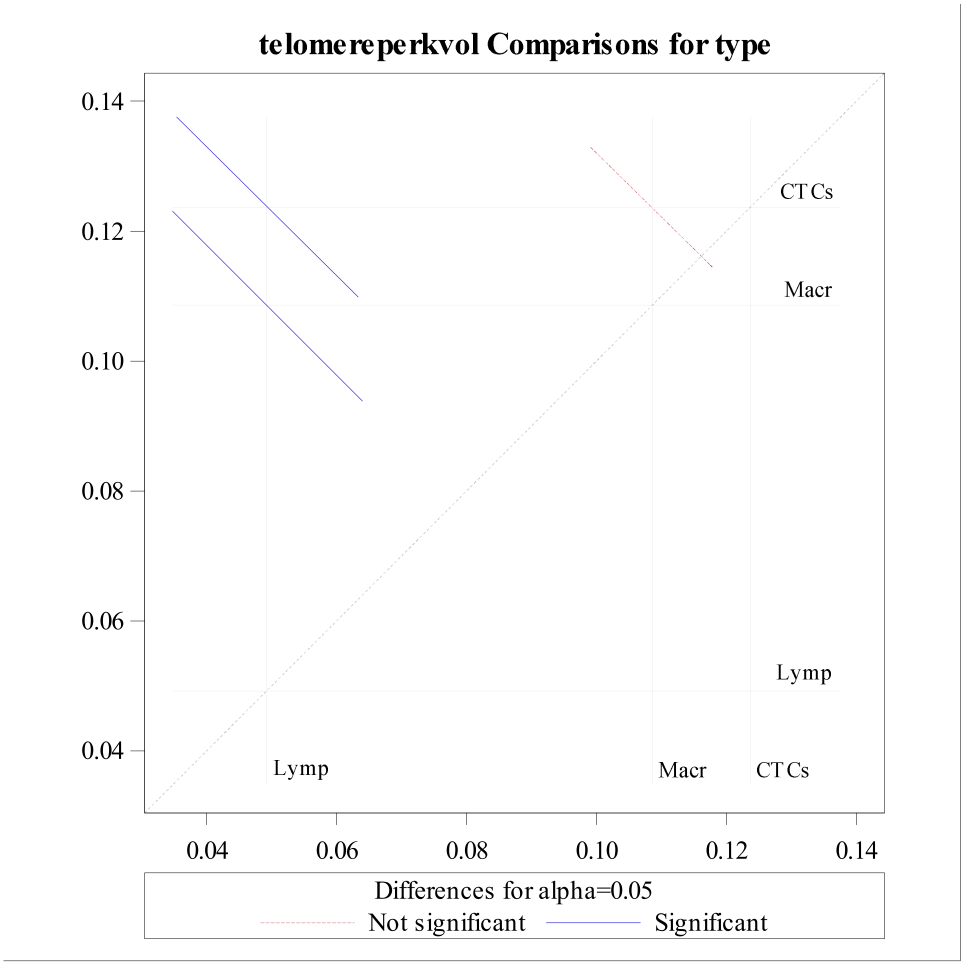

Note: To ensure overall protection level, only probabilities associated with pre-planned comparisons should be used.

The GLM Procedure

pt=16AA7320

Class Level Information		
**Class**	**Levels**	**Values**
**type**	3	CTCs Lymp Macr

**Number of Observations Read**	83
**Number of Observations Used**	83

The GLM Procedure

Dependent Variable: Totalnofsignals
Totalnofsignals

pt=16AA7320

Source	DF	Sum of Squares	Mean Square	F Value	Pr > F
**Model**	2	69736.8597	34868.4299	21.30	<.0001
**Error**	80	130963.3089	1637.0414		
**Corrected Total**	82	200700.1687			

R-Square	Coeff Var	Root MSE	Totalnofsignals Mean
0.347468	78.20705	40.46037	51.73494

Source	DF	Type III SS	Mean Square	F Value	Pr > F
**type**	2	69736.85973	34868.42987	21.30	<.0001

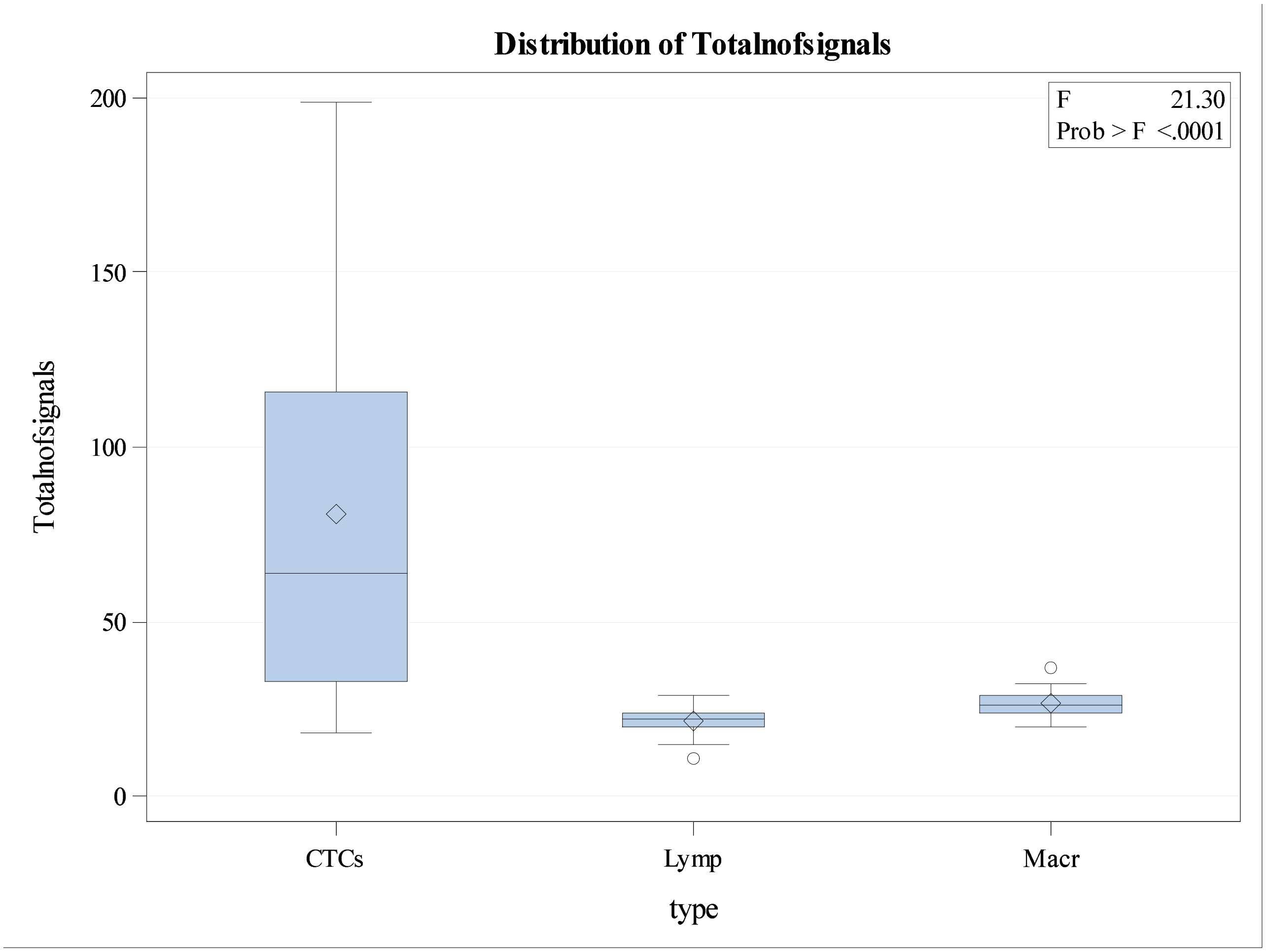

The GLM Procedure

Dependent Variable: Totalnofaggregates Totalnofaggregates

pt=16AA7320

Source	DF	Sum of Squares	Mean Square	F Value	Pr > F
Model	2	1070.268606	535.134303	14.27	<.0001
Error	80	2999.972358	37.499654		
Corrected Total	82	4070.240964			

R-Square	Coeff Var	Root MSE	Totalnofaggregates Mean
0.262950	109.7768	6.123696	5.578313

Source	DF	Type III SS	Mean Square	F Value	Pr > F
type	2	1070.268606	535.134303	14.27	<.0001

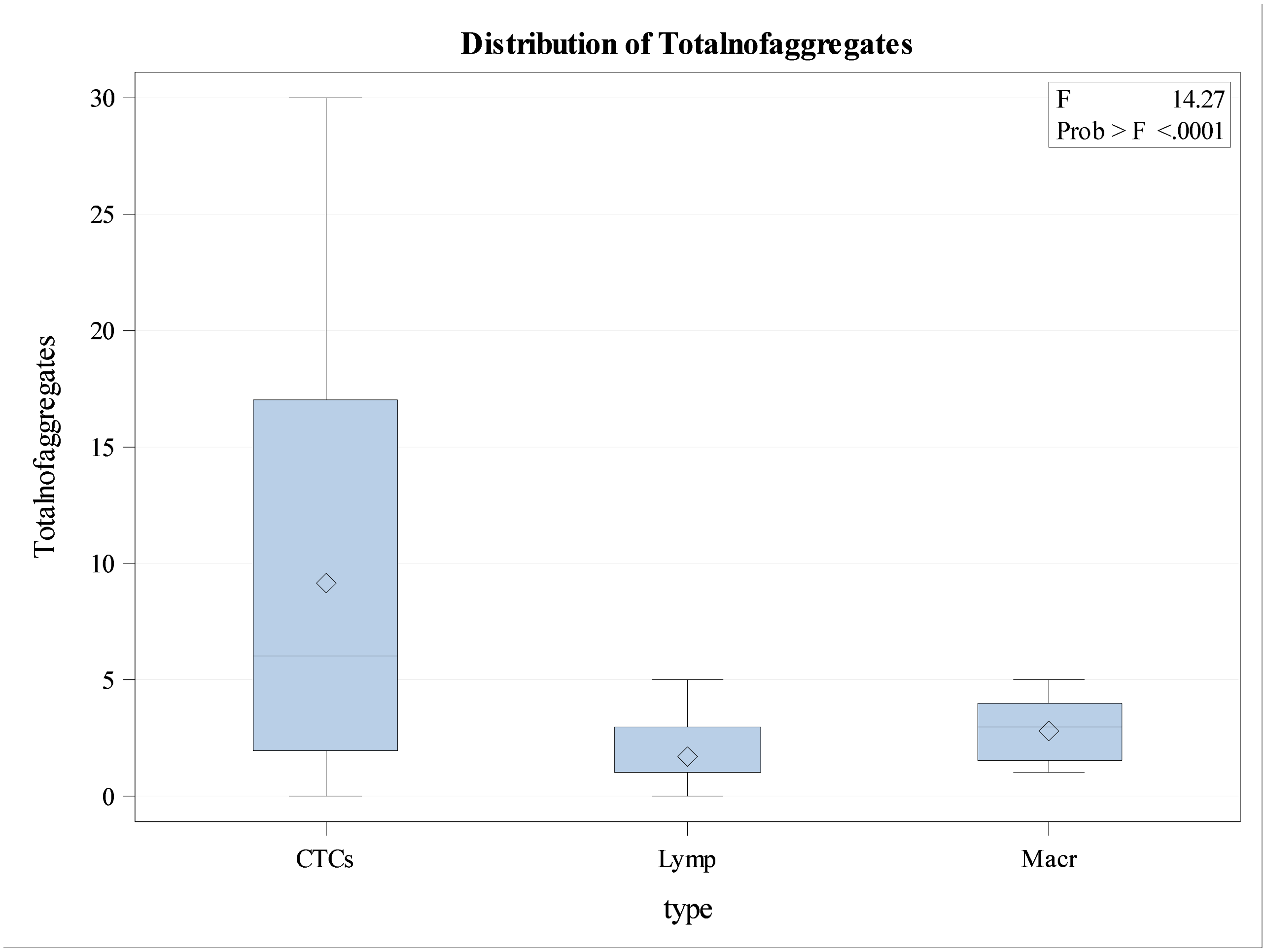

The GLM Procedure

Dependent Variable: acratio
acratio

pt=16AA7320

Source	DF	Sum of Squares	Mean Square	F Value	Pr > F
**Model**	2	241.8629818	120.9314909	20.58	<.0001
**Error**	80	470.1980437	5.8774755		
**Corrected Total**	82	712.0610255			

R-Square	Coeff Var	Root MSE	acratio Mean
0.339666	68.30916	2.424351	3.549085

Source	DF	Type III SS	Mean Square	F Value	Pr > F
**type**	2	241.8629818	120.9314909	20.58	<.0001

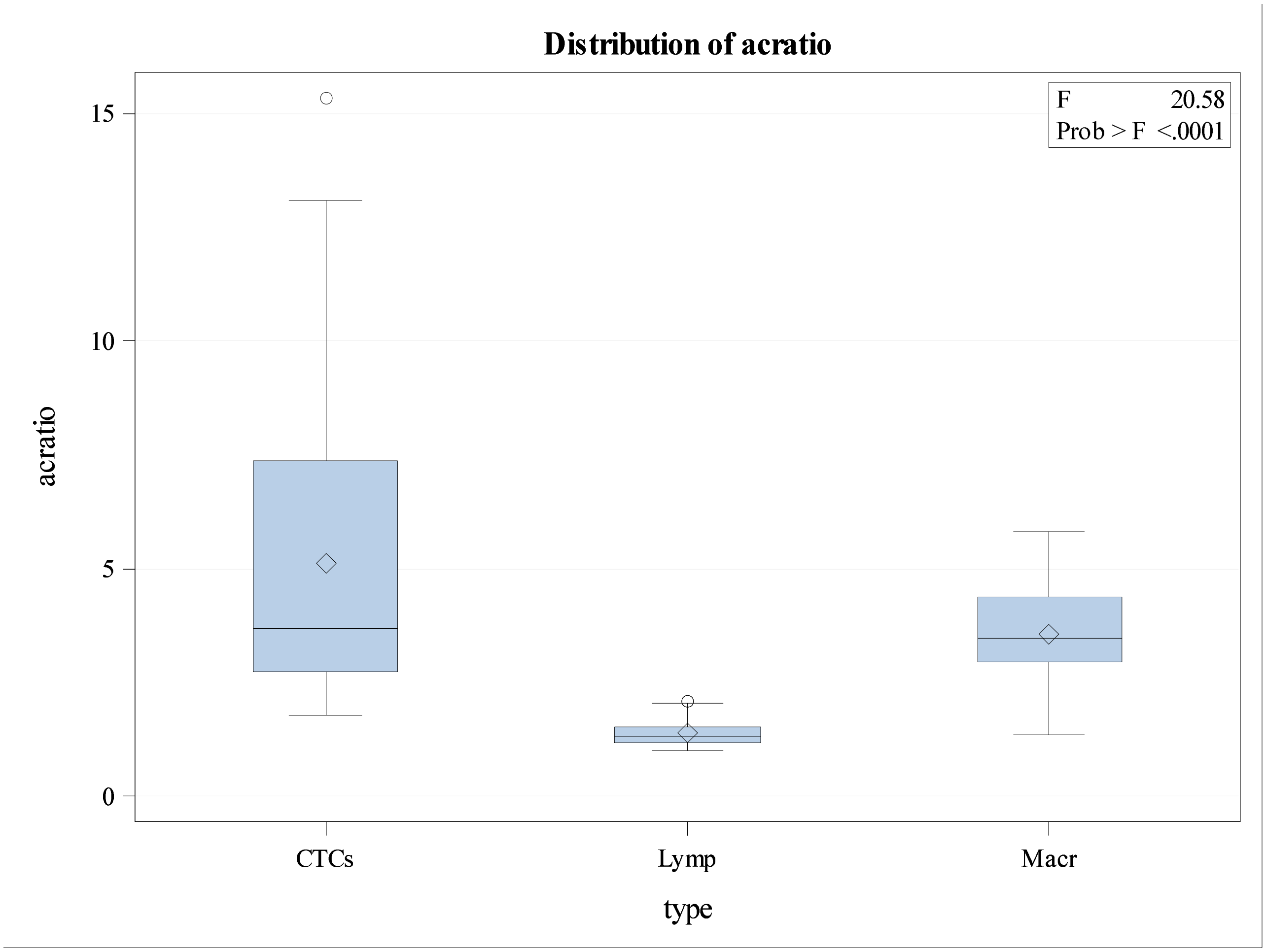

The GLM Procedure

Dependent Variable: AvIntallsignals
AvIntallsignals

pt=16AA7320

Source	DF	Sum of Squares	Mean Square	F Value	Pr > F
**Model**	2	5201981327	2600990664	103.56	<.0001
**Error**	80	2009342891	25116786		
**Corrected Total**	82	7211324218			

R-Square	Coeff Var	Root MSE	AvIntallsignals Mean
0.721363	27.42473	5011.665	18274.25

Source	DF	Type III SS	Mean Square	F Value	Pr > F
**type**	2	5201981327	2600990664	103.56	<.0001

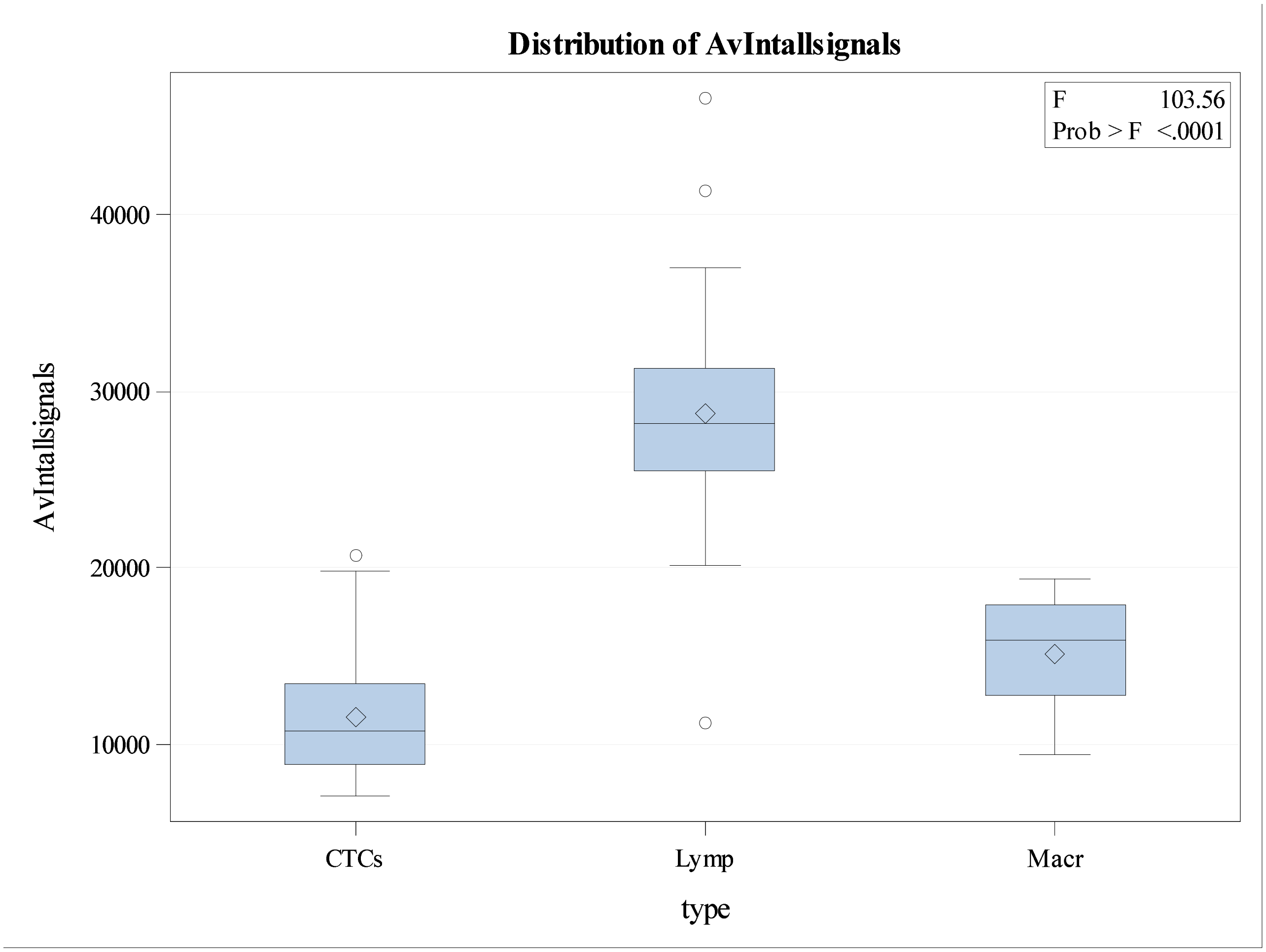

The GLM Procedure

Dependent Variable: Totalintensity Totalintensity

pt=16AA7320

Source	DF	Sum of Squares	Mean Square	F Value	Pr > F
Model	2	2.2402935E12	1.1201468E12	6.74	0.0020
Error	80	1.3289486E13	166118577497		
Corrected Total	82	1.552978E13			

R-Square	Coeff Var	Root MSE	Totalintensity Mean
0.144258	58.17479	407576.5	700606.7

Source	DF	Type III SS	Mean Square	F Value	Pr > F
type	2	2.2402935E12	1.1201468E12	6.74	0.0020

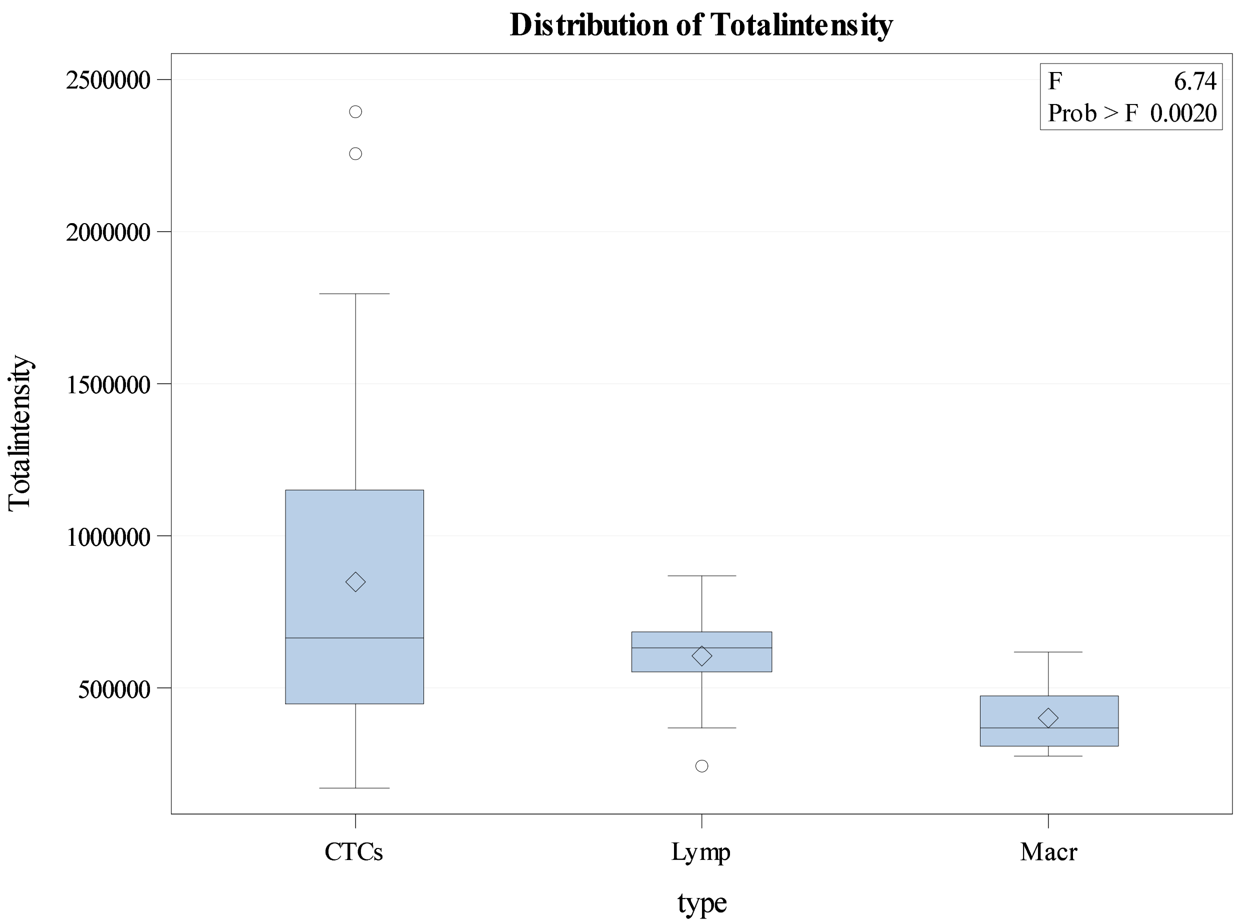

The GLM Procedure

Dependent Variable: Nuclearvolume Nuclearvolume

pt=16AA7320

Source	DF	Sum of Squares	Mean Square	F Value	Pr > F
**Model**	2	7.6233147E12	3.8116574E12	68.50	<.0001
**Error**	80	4.4518117E12	55647645804		
**Corrected Total**	82	1.2075126E13			

R-Square	Coeff Var	Root MSE	Nuclearvolume Mean
0.631324	47.23352	235897.5	499428.2

Source	DF	Type III SS	Mean Square	F Value	Pr > F
**type**	2	7.6233147E12	3.8116574E12	68.50	<.0001

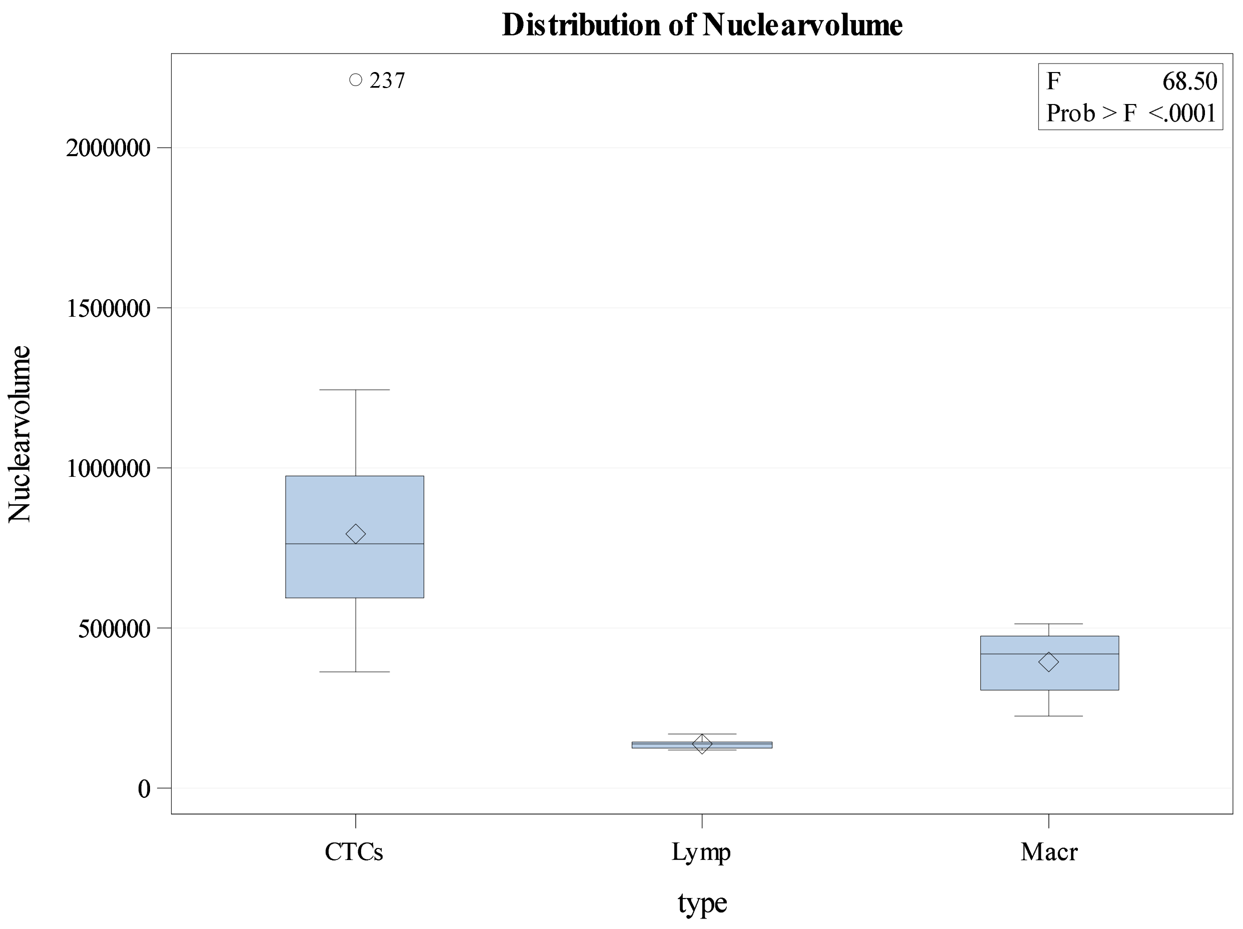

The GLM Procedure

Dependent Variable: telomereperkvol

pt=16AA7320

Source	DF	Sum of Squares	Mean Square	F Value	Pr > F
Model	2	0.08079289	0.04039645	21.68	<.0001
Error	80	0.14903417	0.00186293		
Corrected Total	82	0.22982706			

R-Square	Coeff Var	Root MSE	telomereperkvol Mean
0.351538	36.85276	0.043162	0.117119

Source	DF	Type III SS	Mean Square	F Value	Pr > F
type	2	0.08079289	0.04039645	21.68	<.0001

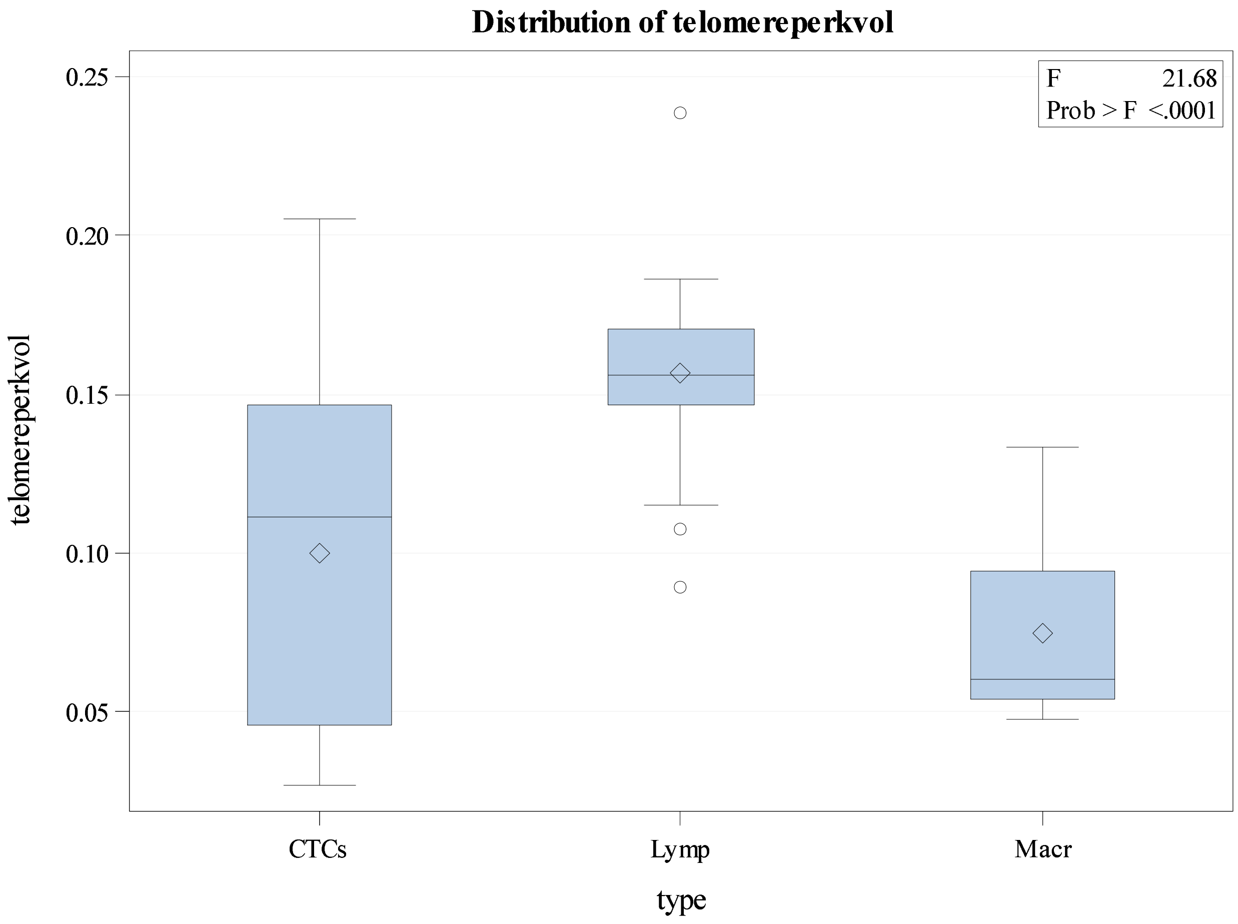

The GLM Procedure
Least Squares Means

pt=16AA7320

type	Totalnofsignals LSMEAN	Standard Error	Pr > |t|	LSMEAN Number
**CTCs**	81.0243902	6.3188478	<.0001	1
**Lymp**	21.6666667	7.3870187	0.0044	2
**Macr**	26.8333333	11.6799021	0.0242	3

Least Squares Means for effect type<br>Pr > |t| for H0: LSMean(i)=LSMean(j)<br><br>Dependent Variable: Totalnofsignals			
**i/j**	**1**	**2**	**3**
**1**		<.0001	0.0001
**2**	<.0001		0.7095
**3**	0.0001	0.7095	

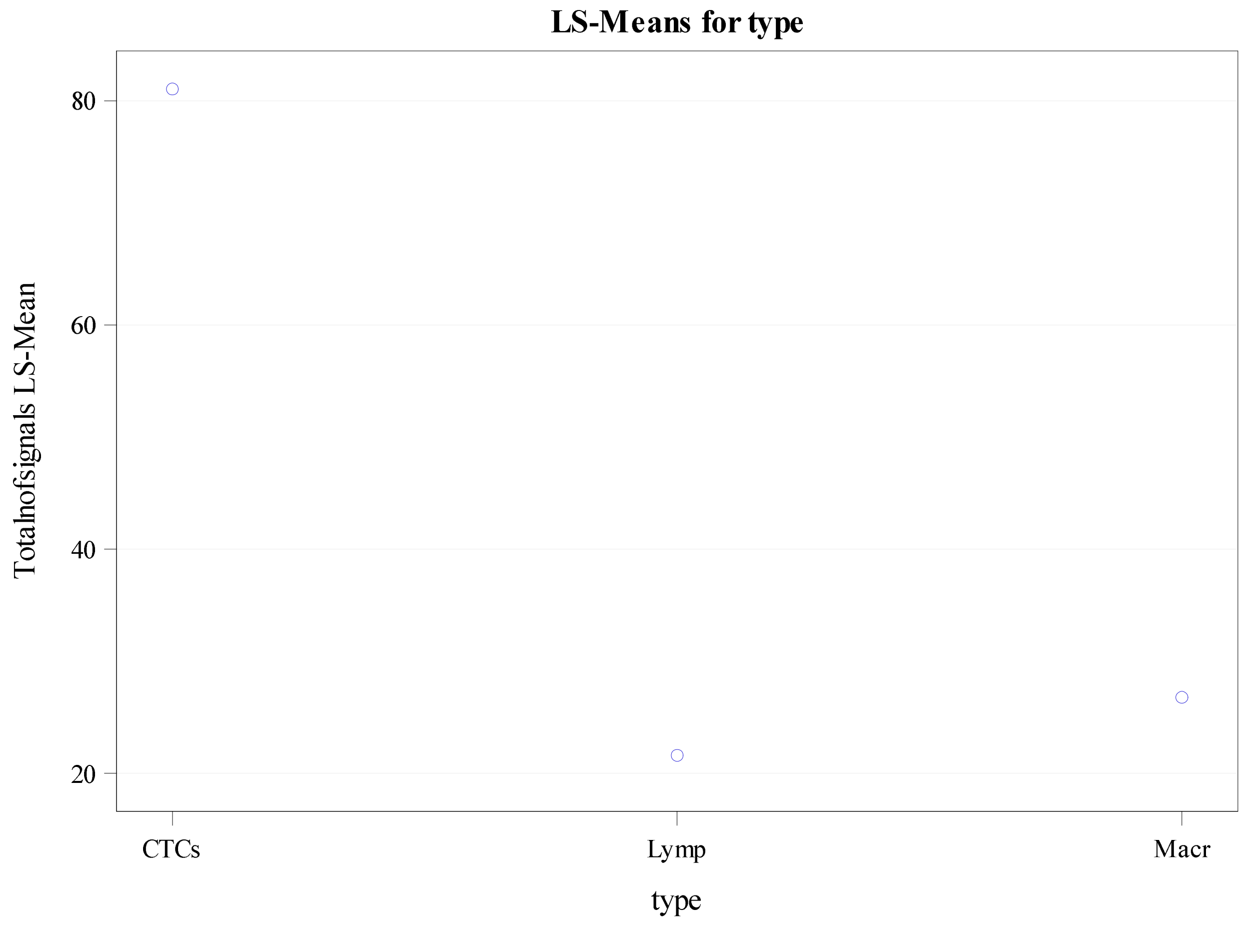

The GLM Procedure
Least Squares Means

pt=16AA7320

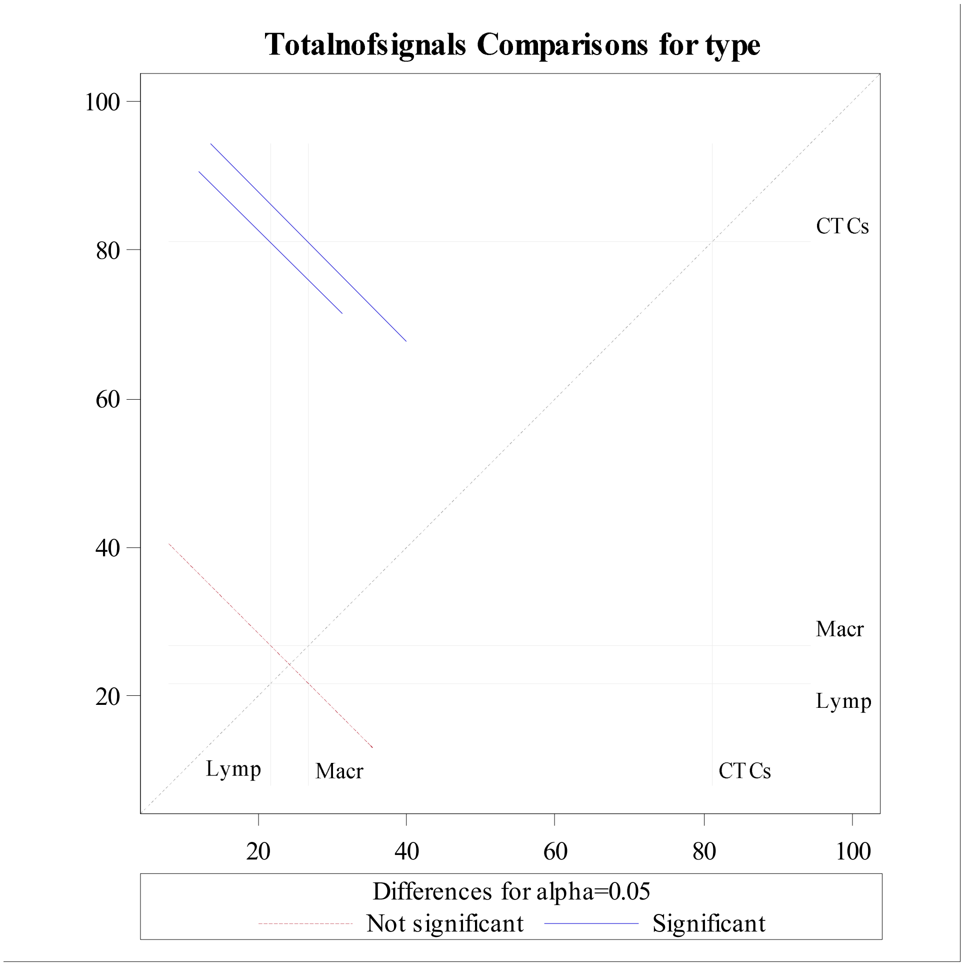

type	Totalnofaggregates LSMEAN	Standard Error	Pr > |t|	LSMEAN Number
**CTCs**	9.19512195	0.95636066	<.0001	1
**Lymp**	1.73333333	1.11802884	0.1250	2
**Macr**	2.83333333	1.76775881	0.1129	3

The GLM Procedure
Least Squares Means

pt=16AA7320

Least Squares Means for effect type<br>Pr > |t| for H0: LSMean(i)=LSMean(j)<br><br>Dependent Variable: Totalnofaggregates			
**i/j**	**1**	**2**	**3**
**1**		<.0001	0.0022
**2**	<.0001		0.6004
**3**	0.0022	0.6004	

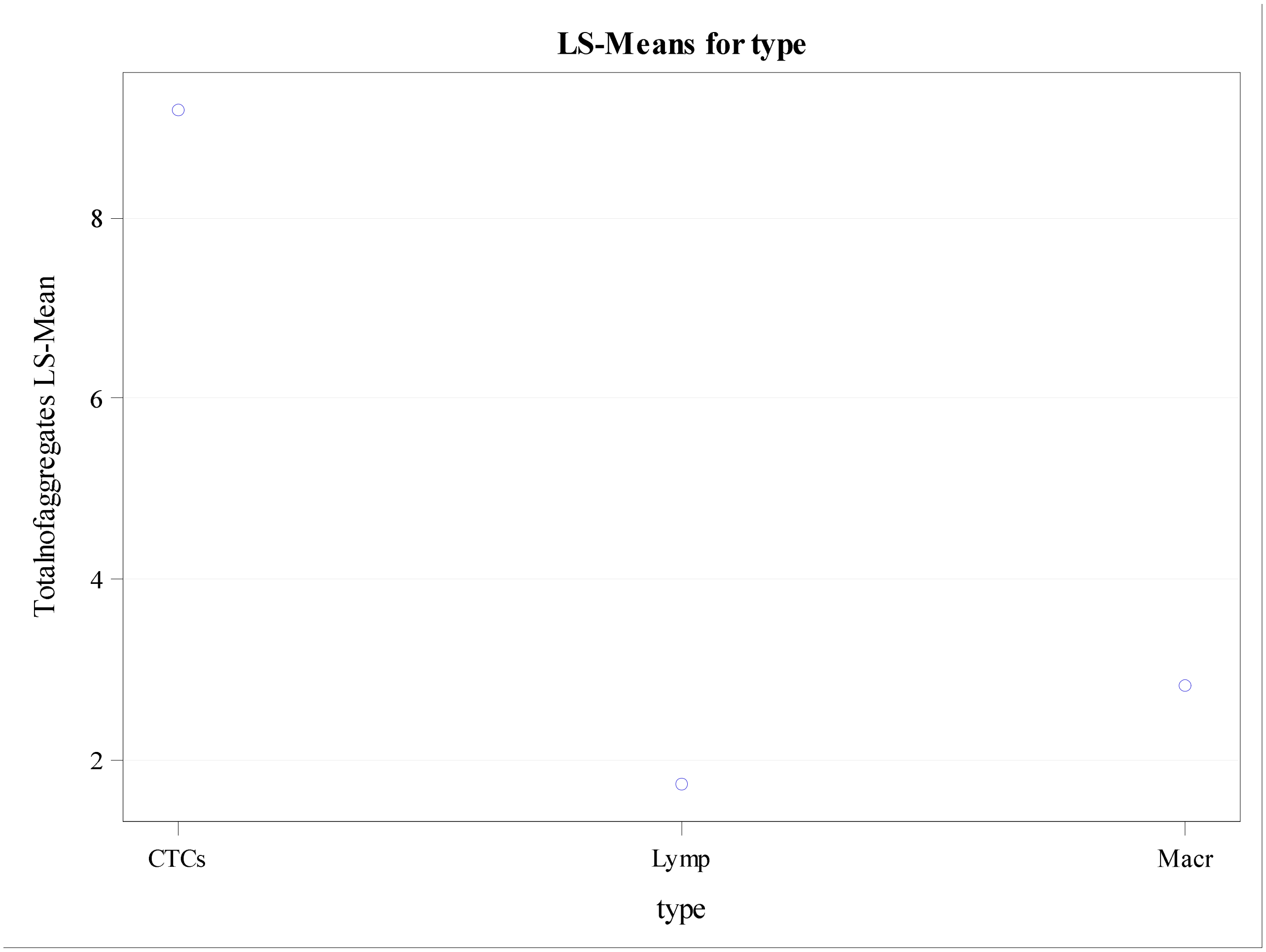

The GLM Procedure
Least Squares Means

pt=16AA7320

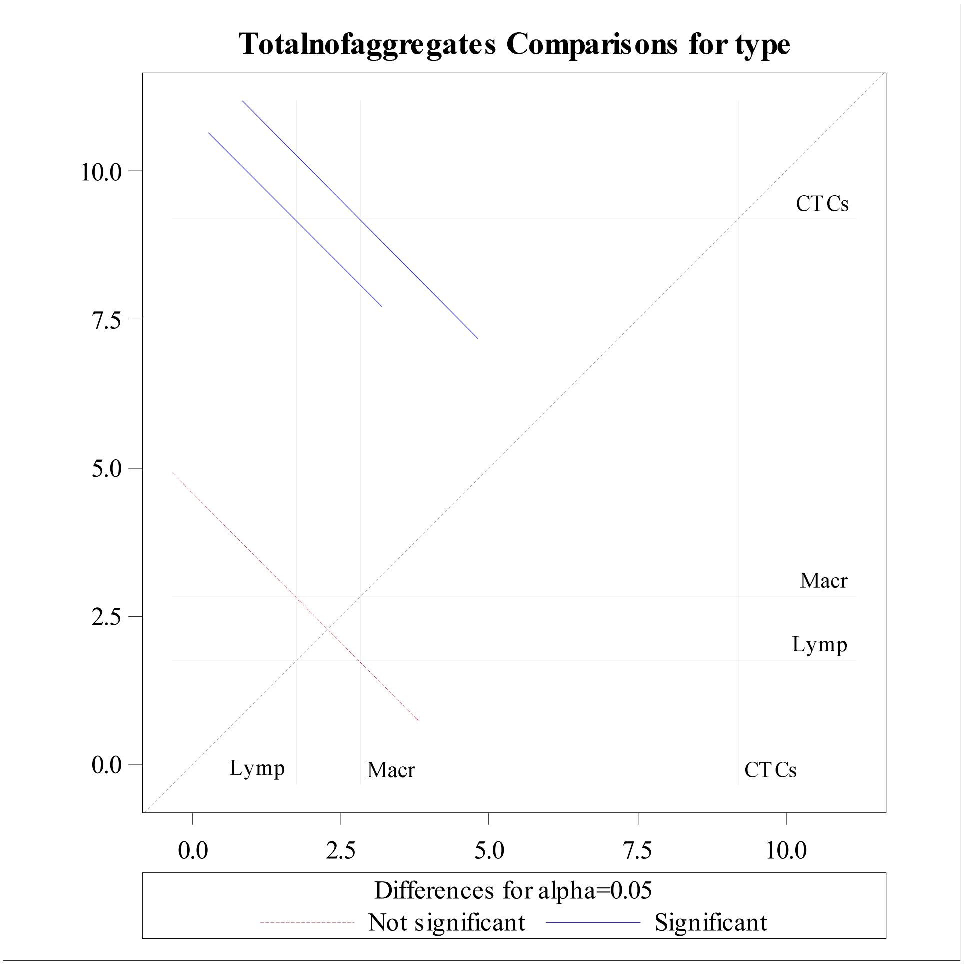

type	acratio LSMEAN	Standard Error	Pr > |t|	LSMEAN Number
**CTCs**	5.12348738	0.37861994	<.0001	1
**Lymp**	1.38709094	0.44262383	0.0024	2
**Macr**	3.57486547	0.69984972	<.0001	3

Least Squares Means for effect type<br>Pr > |t| for H0: LSMean(i)=LSMean(j)<br><br>Dependent Variable: acratio			
**i/j**	**1**	**2**	**3**
**1**		<.0001	0.0551
**2**	<.0001		0.0099
**3**	0.0551	0.0099	

The GLM Procedure
Least Squares Means

pt=16AA7320

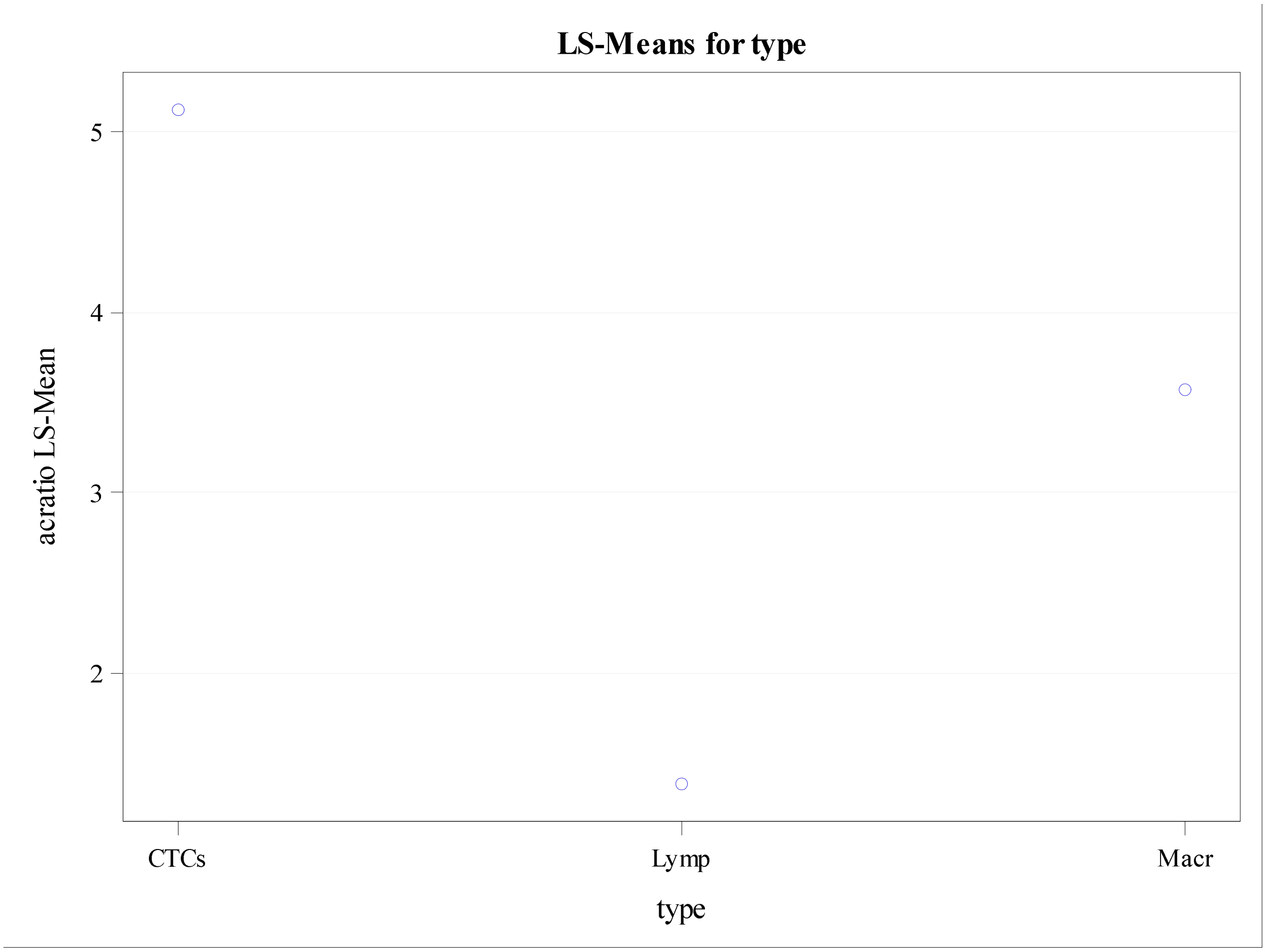

The GLM Procedure
Least Squares Means

pt=16AA7320

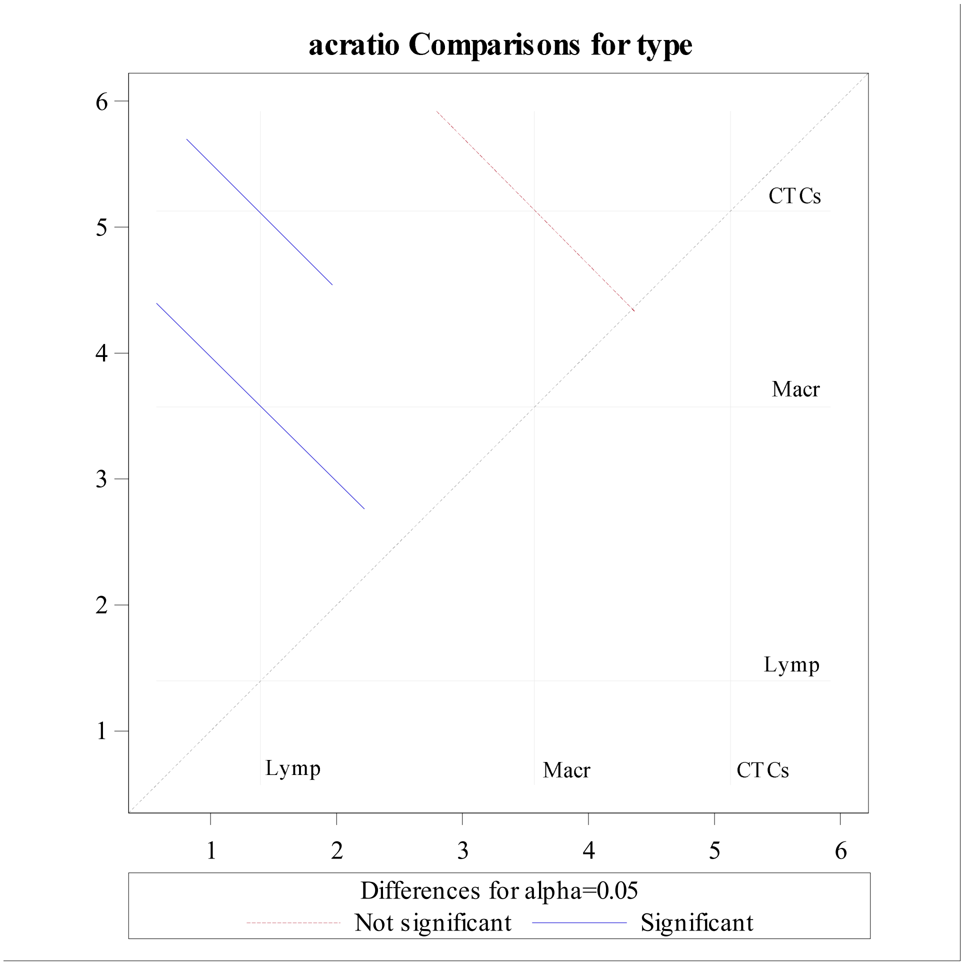

type	AvIntallsignals LSMEAN	Standard Error	Pr > |t|	LSMEAN Number
**CTCs**	11585.0971	782.6906	<.0001	1
**Lymp**	28679.0768	915.0007	<.0001	2
**Macr**	15116.8080	1446.7431	<.0001	3

Least Squares Means for effect type<br>Pr > |t| for H0: LSMean(i)=LSMean(j)<br><br>Dependent Variable: AvIntallsignals			
**i/j**	**1**	**2**	**3**
**1**		<.0001	0.0348
**2**	<.0001		<.0001
**3**	0.0348	<.0001	

The GLM Procedure
Least Squares Means

pt=16AA7320

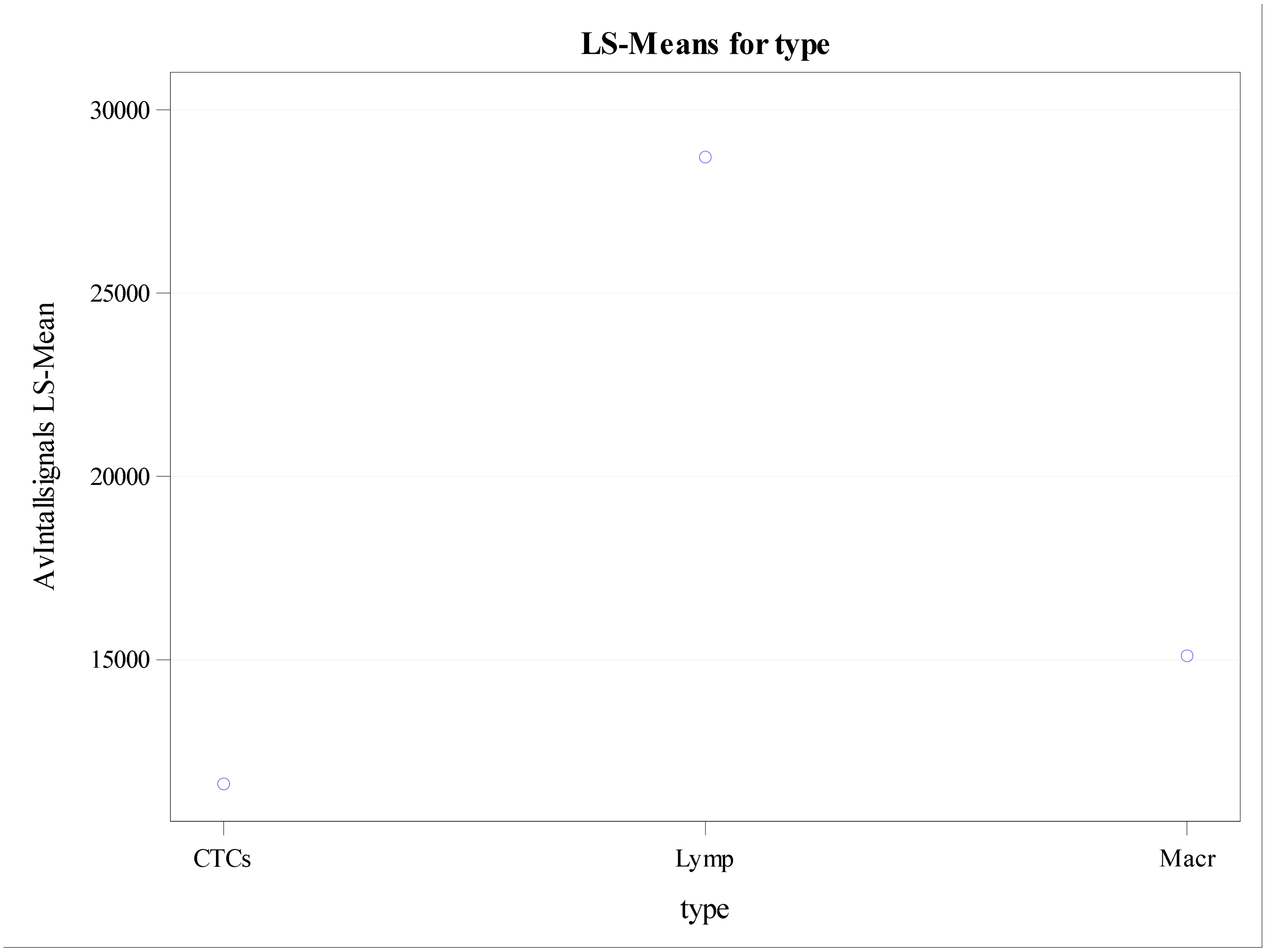

The GLM Procedure
Least Squares Means

pt=16AA7320

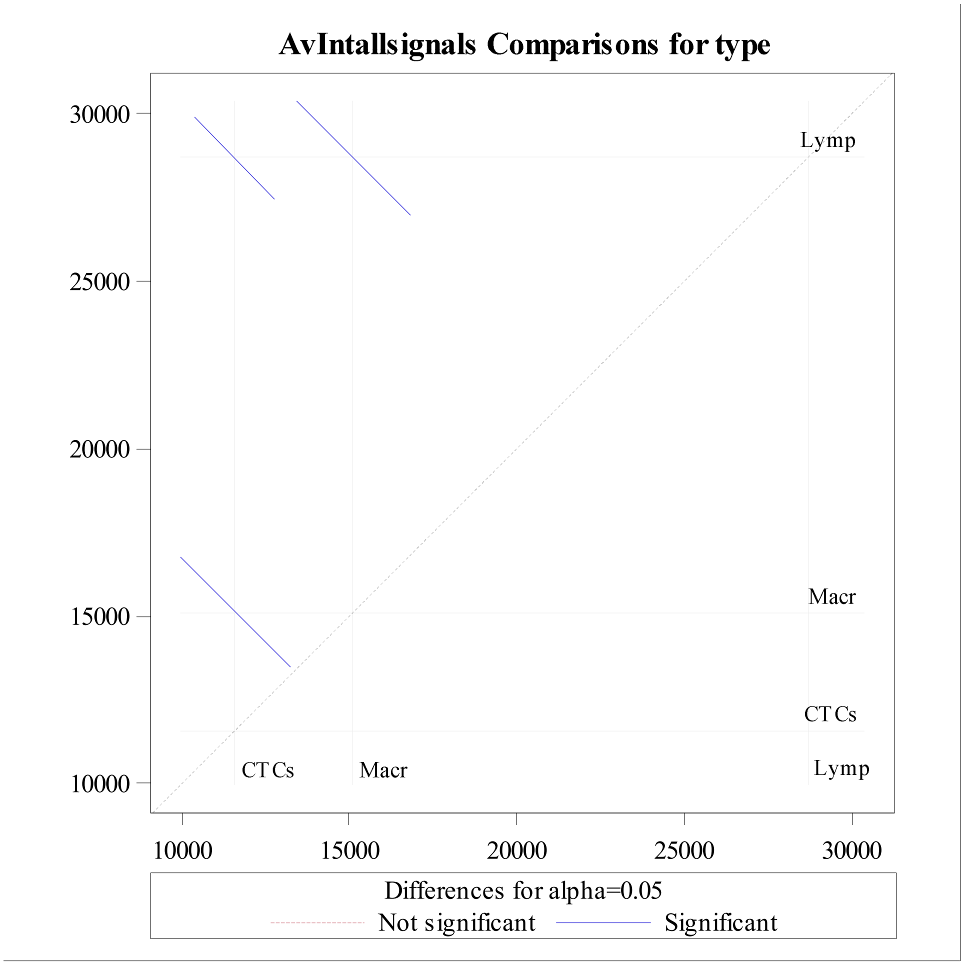

type	Totalintensity LSMEAN	Standard Error	Pr > |t|	LSMEAN Number
**CTCs**	853134.317	63652.750	<.0001	1
**Lymp**	609881.100	74412.942	<.0001	2
**Macr**	406284.333	117657.192	0.0009	3

**Least Squares Means for effect type Pr > |t| for H0: LSMean(i)=LSMean(j)<br><br>Dependent Variable: Totalintensity**			
**i/j**	**1**	**2**	**3**
**1**		0.0151	0.0013
**2**	0.0151		0.1475
**3**	0.0013	0.1475	

The GLM Procedure
Least Squares Means

pt=16AA7320

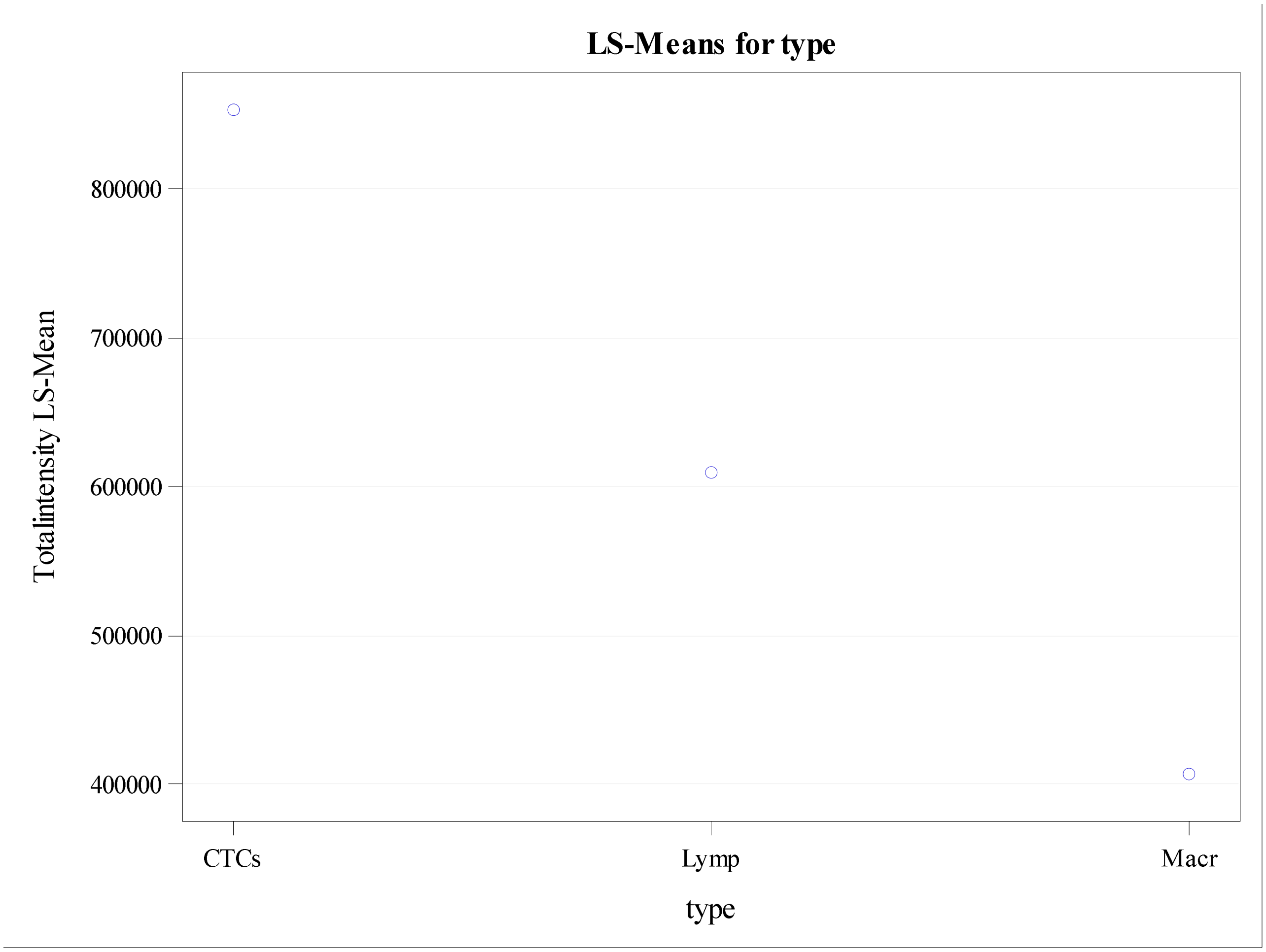

The GLM Procedure
Least Squares Means

pt=16AA7320

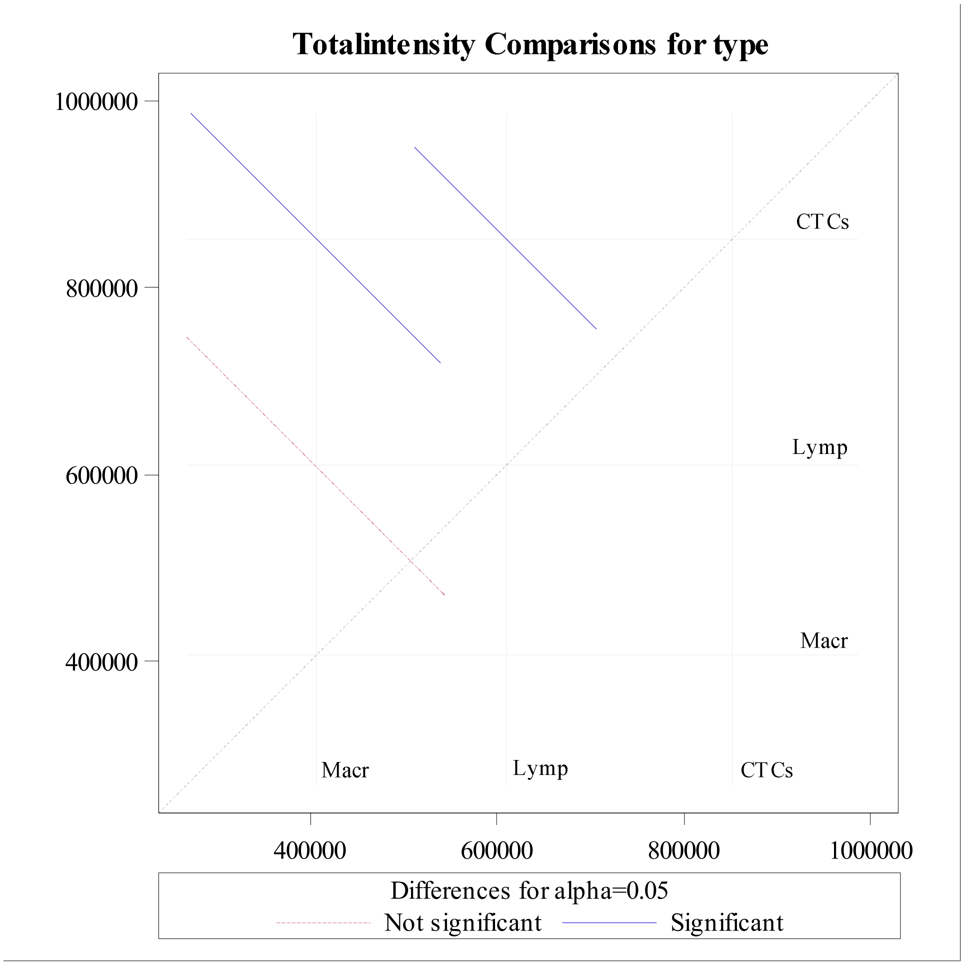

type	Nuclearvolume LSMEAN	Standard Error	Pr > |t|	LSMEAN Number
**CTCs**	794816.829	36841.005	<.0001	1
**Lymp**	138459.667	43068.800	0.0019	2
**Macr**	392605.083	68097.752	<.0001	3

Least Squares Means for effect type<br>Pr > |t| for H0: LSMean(i)=LSMean(j)<br><br>Dependent Variable: Nuclearvolume			
**i/j**	**1**	**2**	**3**
**1**		<.0001	<.0001
**2**	<.0001		0.0023
**3**	<.0001	0.0023	

The GLM Procedure
Least Squares Means

pt=16AA7320

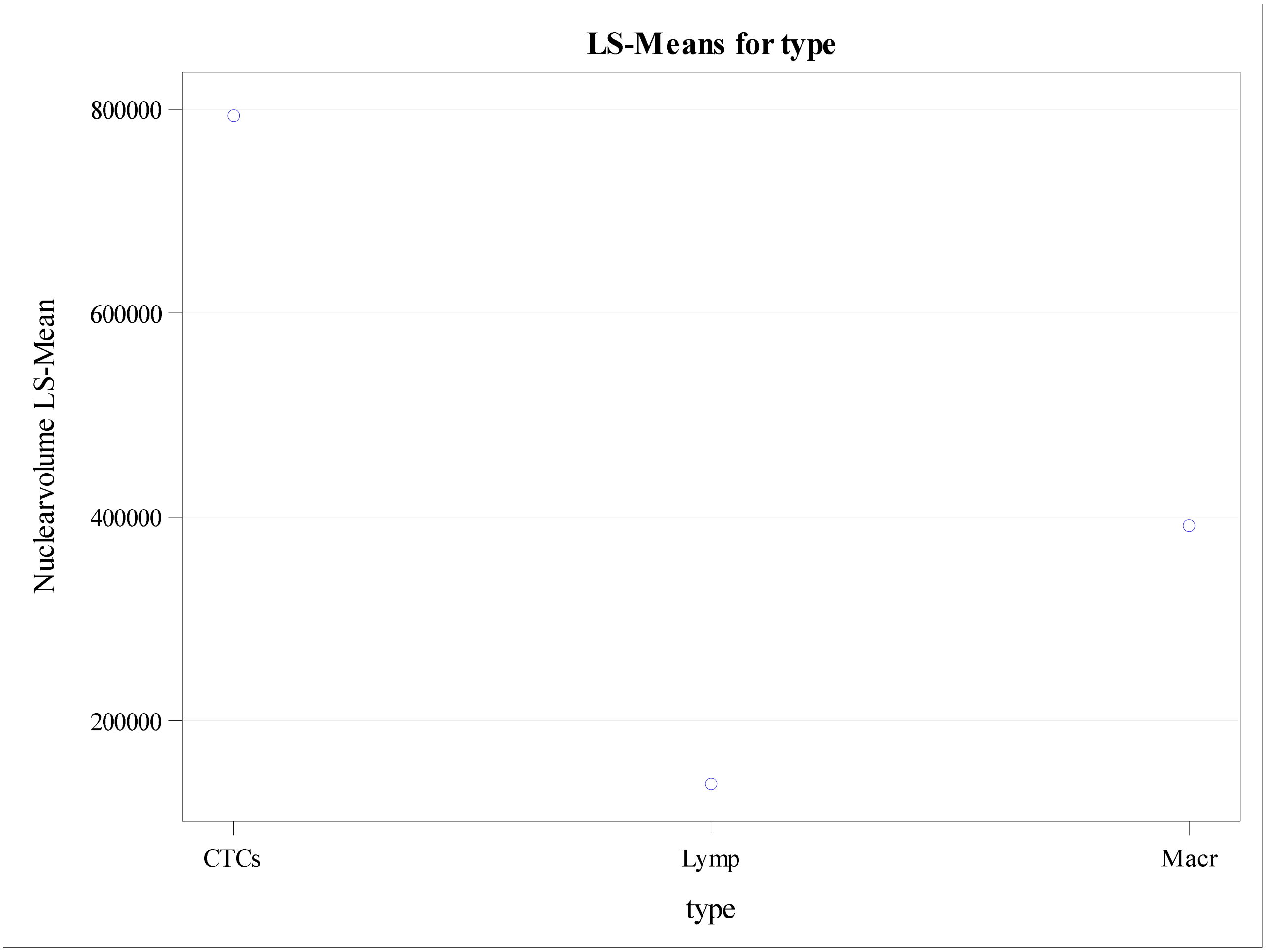

The GLM Procedure
Least Squares Means

pt=16AA7320

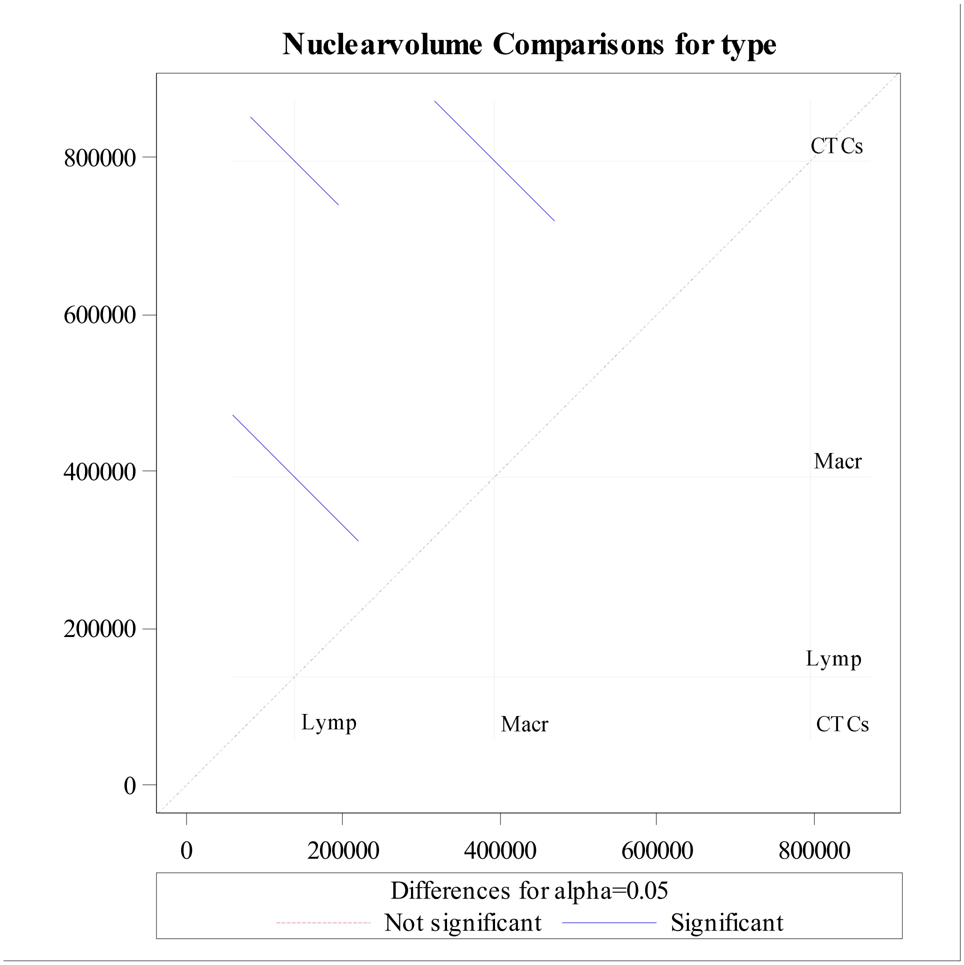

type	telomereperkvol LSMEAN	Standard Error	Pr > |t|	LSMEAN Number
**CTCs**	0.10027269	0.00674072	<.0001	1
**Lymp**	0.15702634	0.00788020	<.0001	2
**Macr**	0.07490989	0.01245969	<.0001	3

Least Squares Means for effect type
Pr > |t| for H0: LSMean(i)=LSMean(j)

Dependent Variable: telomereperkvol

i/j	1	2	3
**1**		<.0001	0.0772
**2**	<.0001		<.0001
**3**	0.0772	<.0001	

The GLM Procedure
Least Squares Means

pt=16AA7320

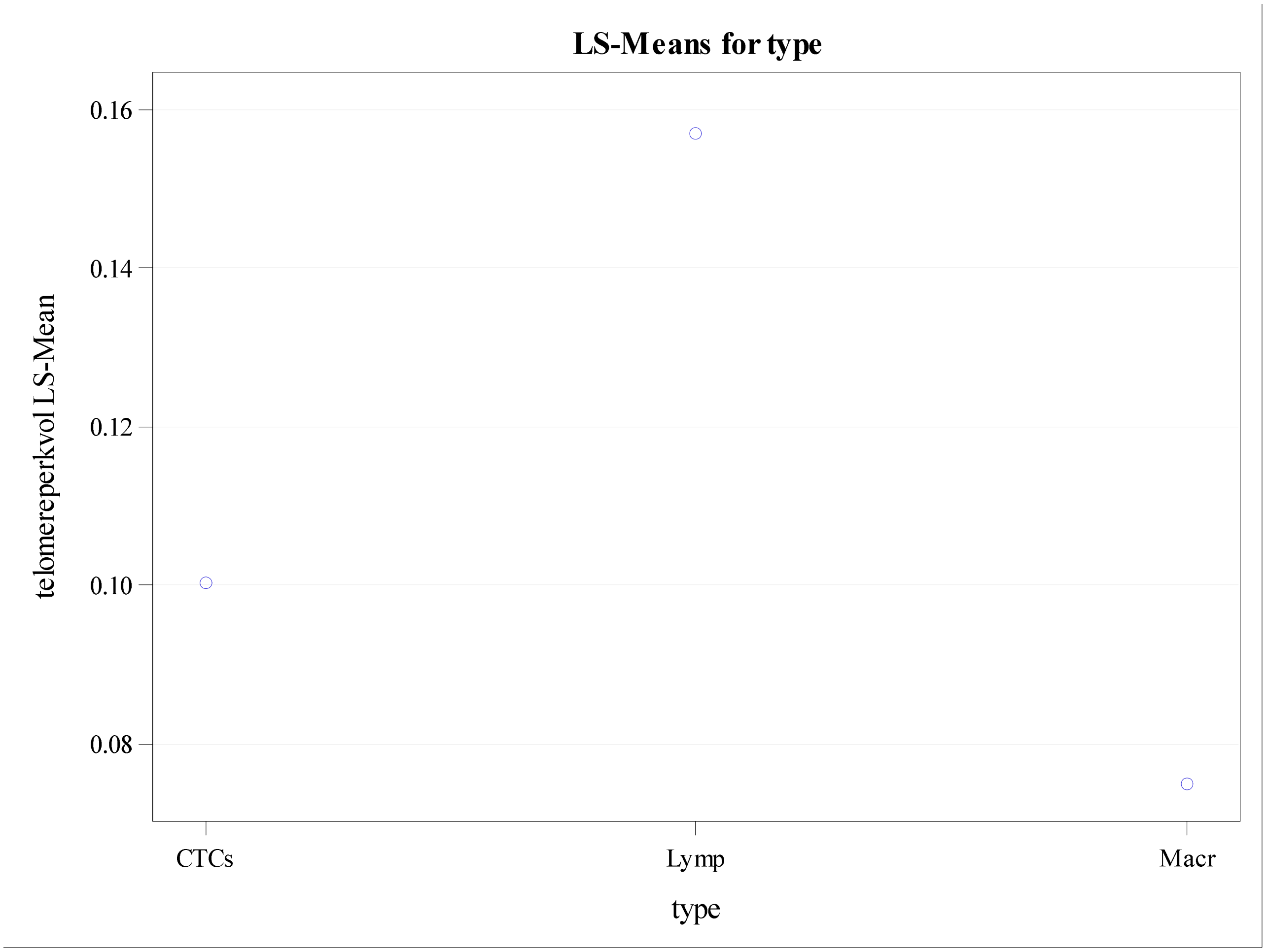

The GLM Procedure
Least Squares Means

pt=16AA7320

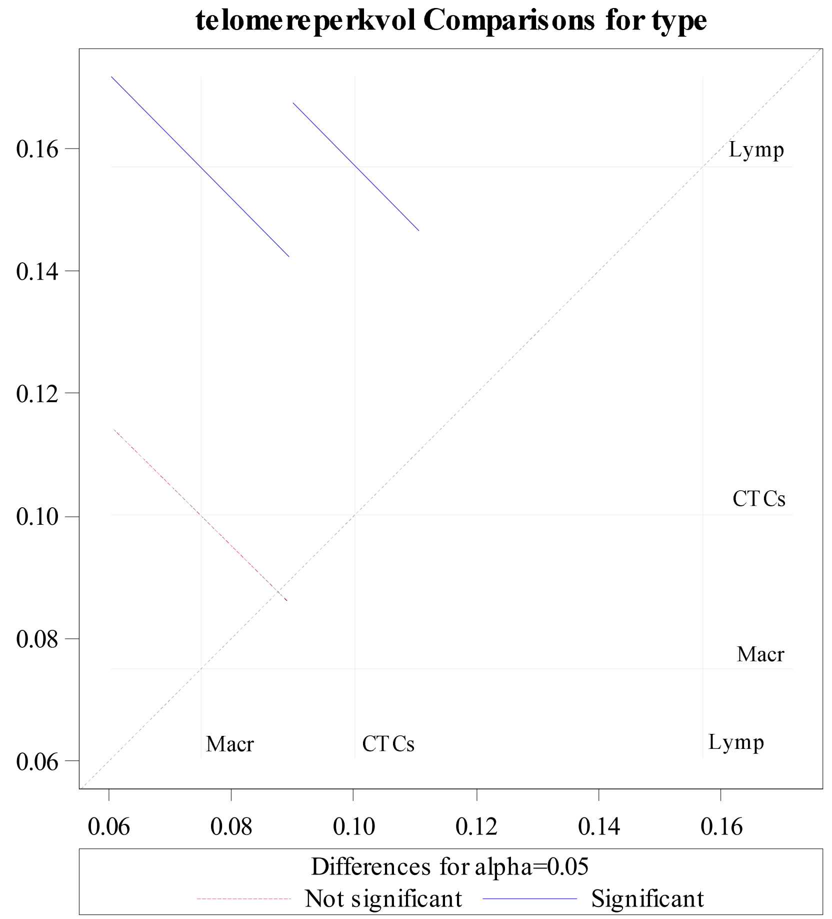

Note: To ensure overall protection level, only probabilities associated with pre-planned comparisons should be used.

The GLM Procedure

pt=16AA8528

Class Level Information		
**Class**	**Levels**	**Values**
**type**	3	CTCs Lymp Macr

**Number of Observations Read**	44
**Number of Observations Used**	44

The GLM Procedure

Dependent Variable: Totalnofsignals
Totalnofsignals

pt=16AA8528

Source	DF	Sum of Squares	Mean Square	F Value	Pr > F
**Model**	2	78821.6594	39410.8297	10.78	0.0002
**Error**	41	149868.2270	3655.3226		
**Corrected Total**	43	228689.8864			

R-Square	Coeff Var	Root MSE	Totalnofsignals Mean
0.344666	69.22216	60.45926	87.34091

Source	DF	Type III SS	Mean Square	F Value	Pr > F
**type**	2	78821.65935	39410.82968	10.78	0.0002

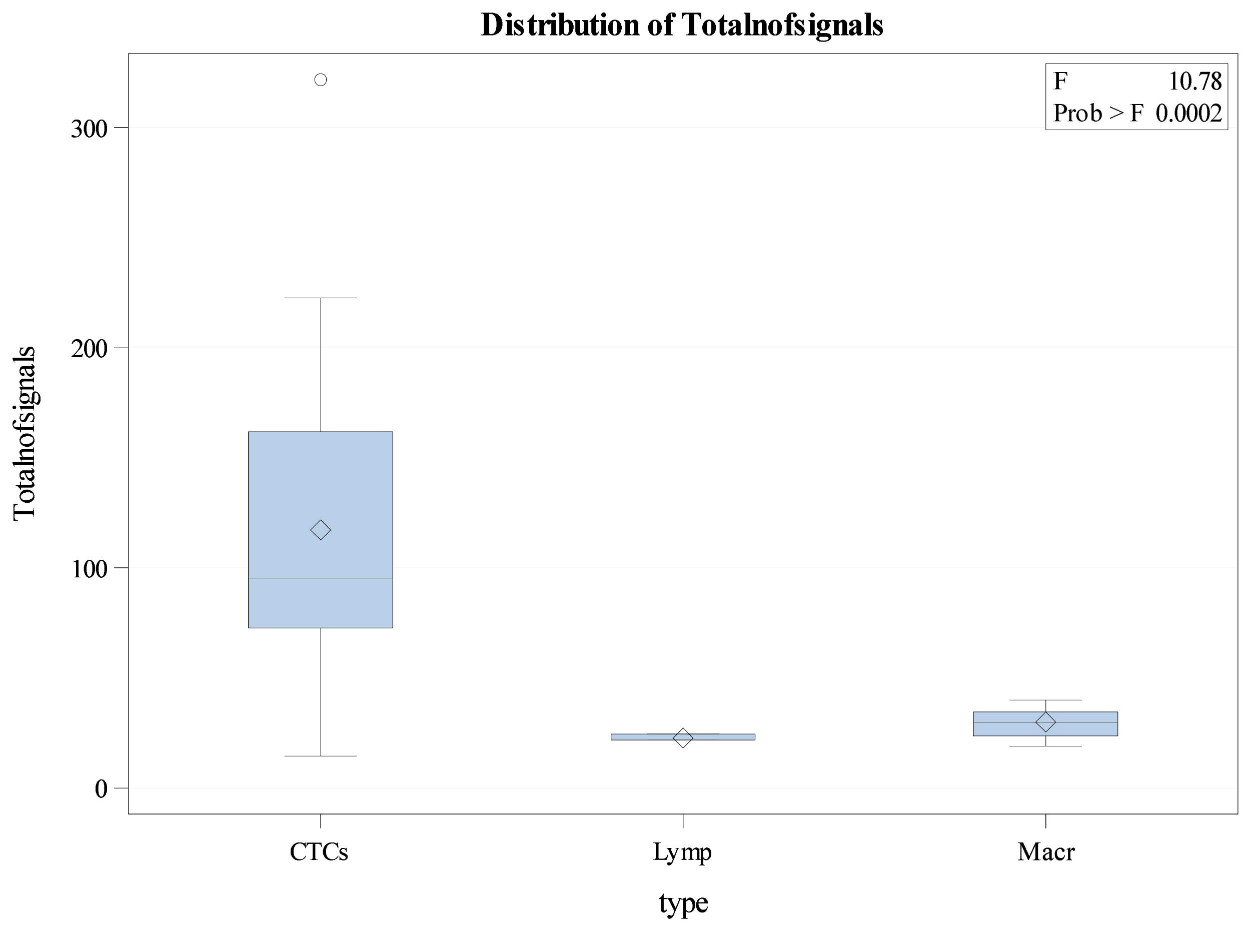

The GLM Procedure

Dependent Variable: Totalnofaggregates Totalnofaggregates

pt=16AA8528

Source	DF	Sum of Squares	Mean Square	F Value	Pr > F
**Model**	2	1243.575758	621.787879	9.32	0.0005
**Error**	41	2735.583333	66.721545		
**Corrected Total**	43	3979.159091			

R-Square	Coeff Var	Root MSE	Totalnofaggregates Mean
0.312522	80.04595	8.168326	10.20455

Source	DF	Type III SS	Mean Square	F Value	Pr > F
**type**	2	1243.575758	621.787879	9.32	0.0005

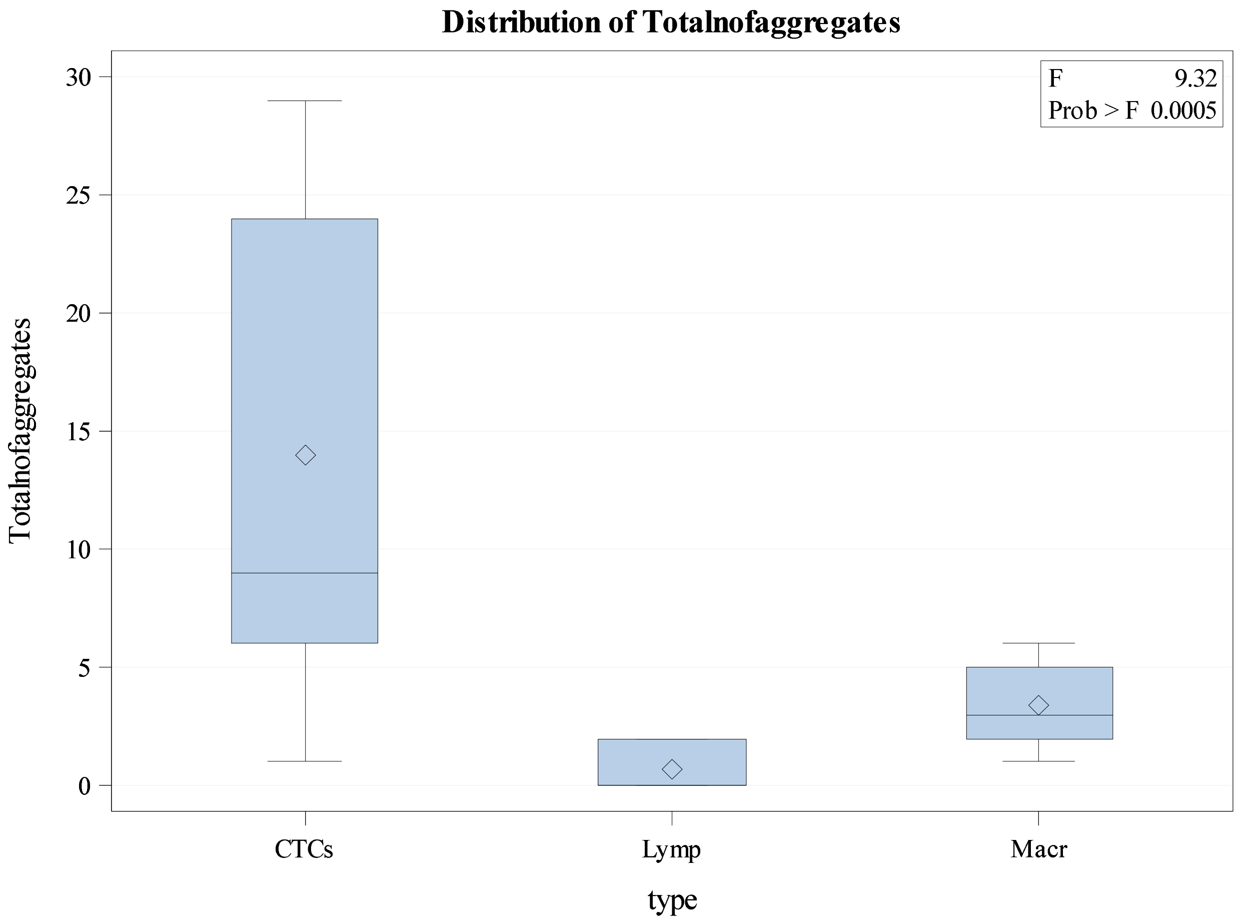

The GLM Procedure

Dependent Variable: acratio
acratio

pt=16AA8528

Source	DF	Sum of Squares	Mean Square	F Value	Pr > F
**Model**	2	4.2694954	2.1347477	0.29	0.7496
**Error**	41	301.4895202	7.3534029		
**Corrected Total**	43	305.7590156			

R-Square	Coeff Var	Root MSE	acratio Mean
0.013964	69.57107	2.711716	3.897763

Source	DF	Type III SS	Mean Square	F Value	Pr > F
**type**	2	4.26949540	2.13474770	0.29	0.7496

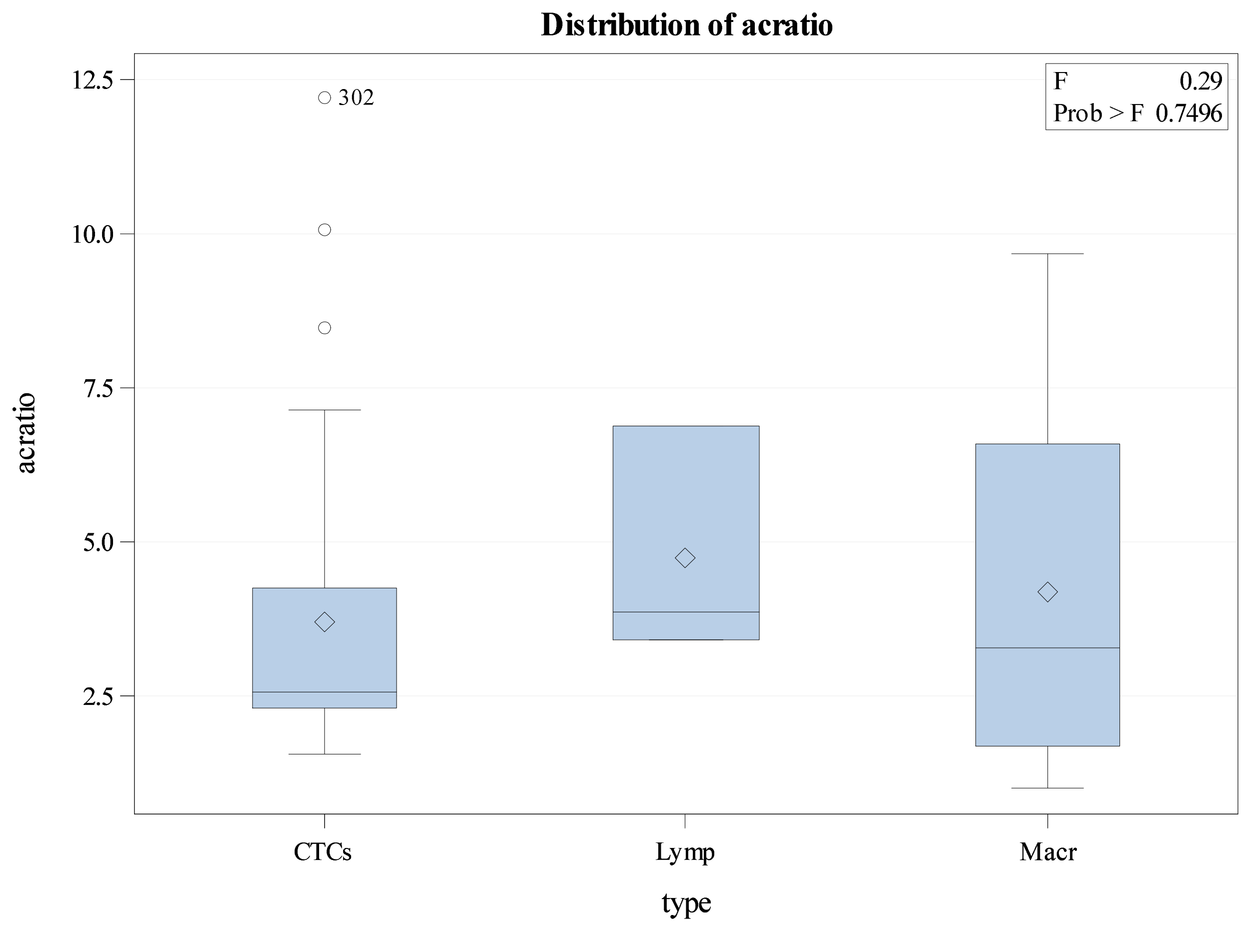

The GLM Procedure

Dependent Variable: AvIntallsignals
AvIntallsignals

pt=16AA8528

Source	DF	Sum of Squares	Mean Square	F Value	Pr > F
**Model**	2	81611616.7	40805808.4	6.11	0.0048
**Error**	41	273771248.3	6677347.5		
**Corrected Total**	43	355382865.0			

R-Square	Coeff Var	Root MSE	AvIntallsignals Mean
0.229644	22.06798	2584.056	11709.53

Source	DF	Type III SS	Mean Square	F Value	Pr > F
**type**	2	81611616.75	40805808.37	6.11	0.0048

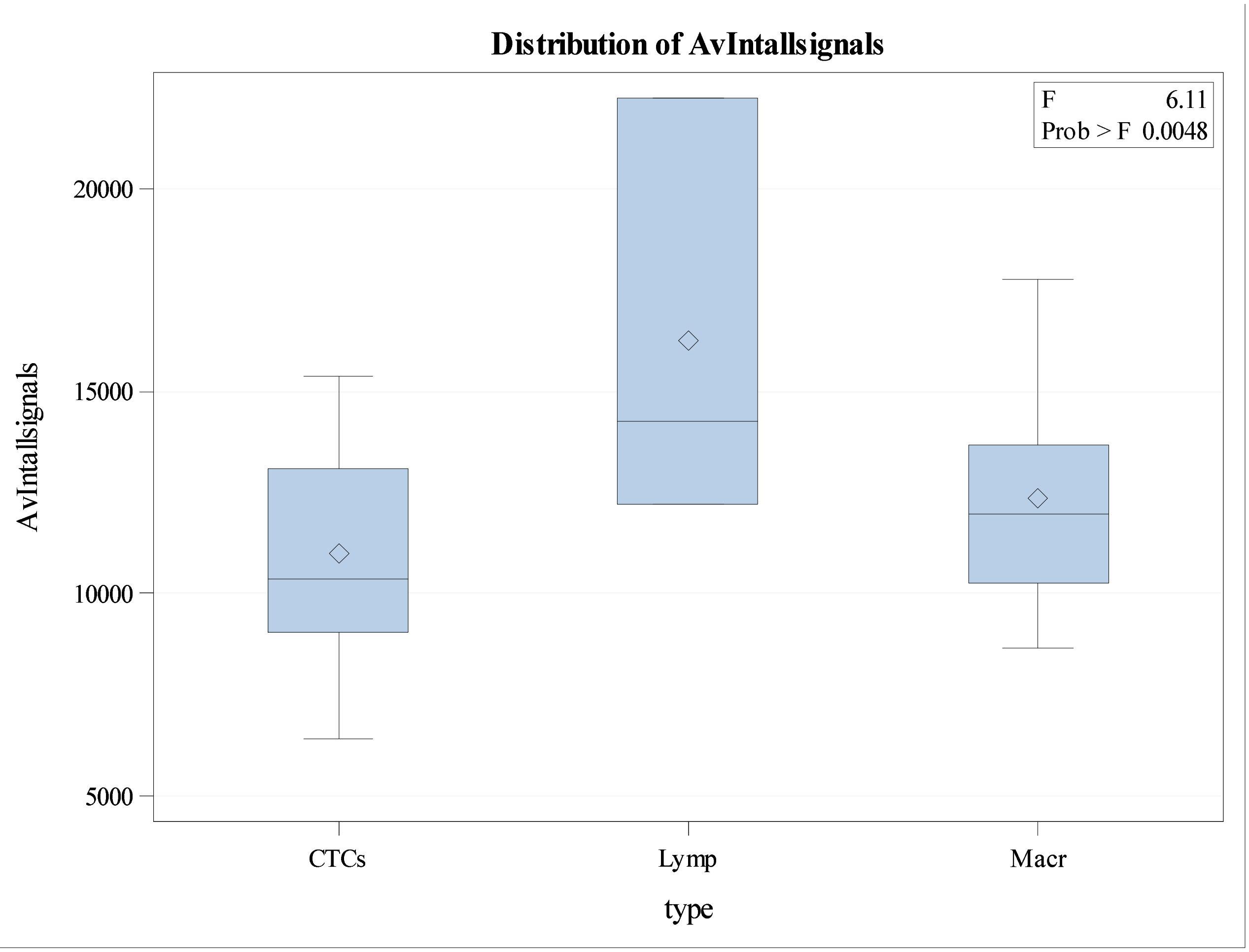

The GLM Procedure

Dependent Variable: Totalintensity
Totalintensity

pt=16AA8528

Source	DF	Sum of Squares	Mean Square	F Value	Pr > F
**Model**	2	7.5375708E12	3.7687854E12	10.45	0.0002
**Error**	41	1.4790674E13	360748136812		
**Corrected Total**	43	2.2328244E13			

R-Square	Coeff Var	Root MSE	Totalintensity Mean
0.337580	63.64346	600623.1	943731.0

Source	DF	Type III SS	Mean Square	F Value	Pr > F
**type**	2	7.5375708E12	3.7687854E12	10.45	0.0002

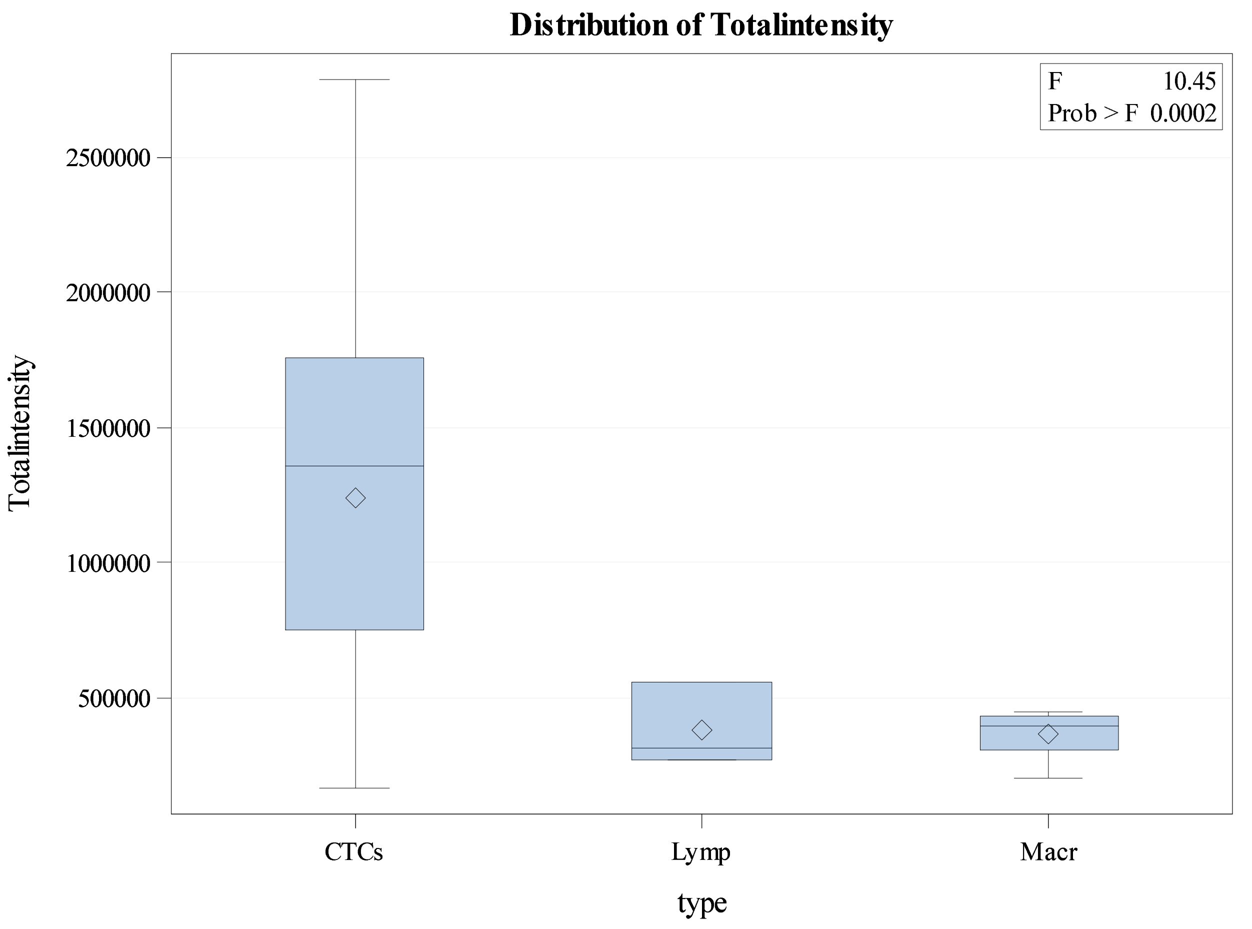

The GLM Procedure

Dependent Variable: Nuclearvolume Nuclearvolume

pt=16AA8528

Source	DF	Sum of Squares	Mean Square	F Value	Pr > F
**Model**	2	2.5587807E12	1.2793904E12	11.00	0.0001
**Error**	41	4.7673154E12	116275986384		
**Corrected Total**	43	7.3260961E12			

R-Square	Coeff Var	Root MSE	Nuclearvolume Mean
0.349269	43.16379	340992.6	789997.0

Source	DF	Type III SS	Mean Square	F Value	Pr > F
**type**	2	2.5587807E12	1.2793904E12	11.00	0.0001

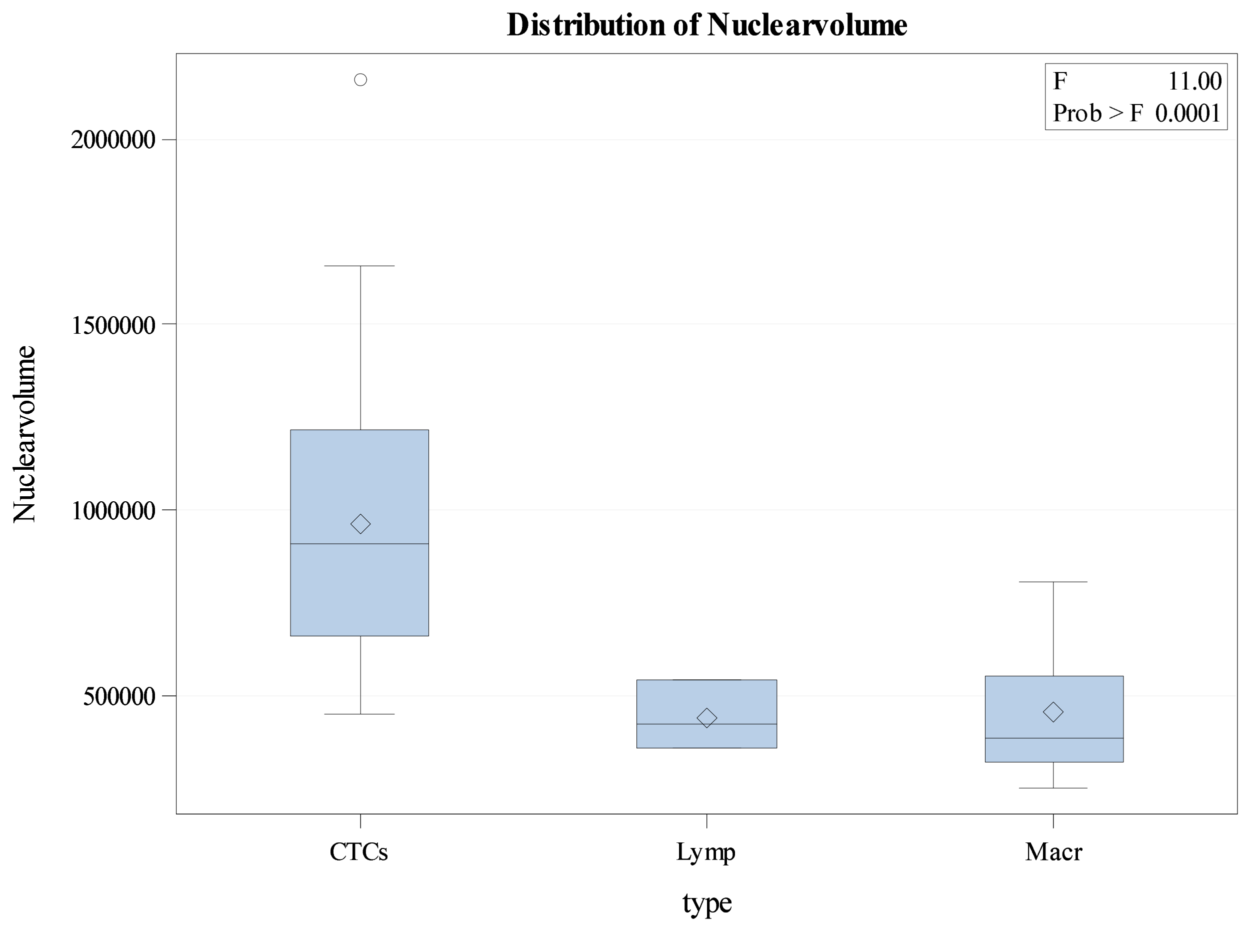

The GLM Procedure

Dependent Variable: telomereperkvol

pt=16AA8528

Source	DF	Sum of Squares	Mean Square	F Value	Pr > F
Model	2	0.03043991	0.01521995	4.42	0.0183
Error	41	0.14121591	0.00344429		
Corrected Total	43	0.17165582			

R-Square	Coeff Var	Root MSE	telomereperkvol Mean
0.177331	56.00607	0.058688	0.104789

Source	DF	Type III SS	Mean Square	F Value	Pr > F
type	2	0.03043991	0.01521995	4.42	0.0183

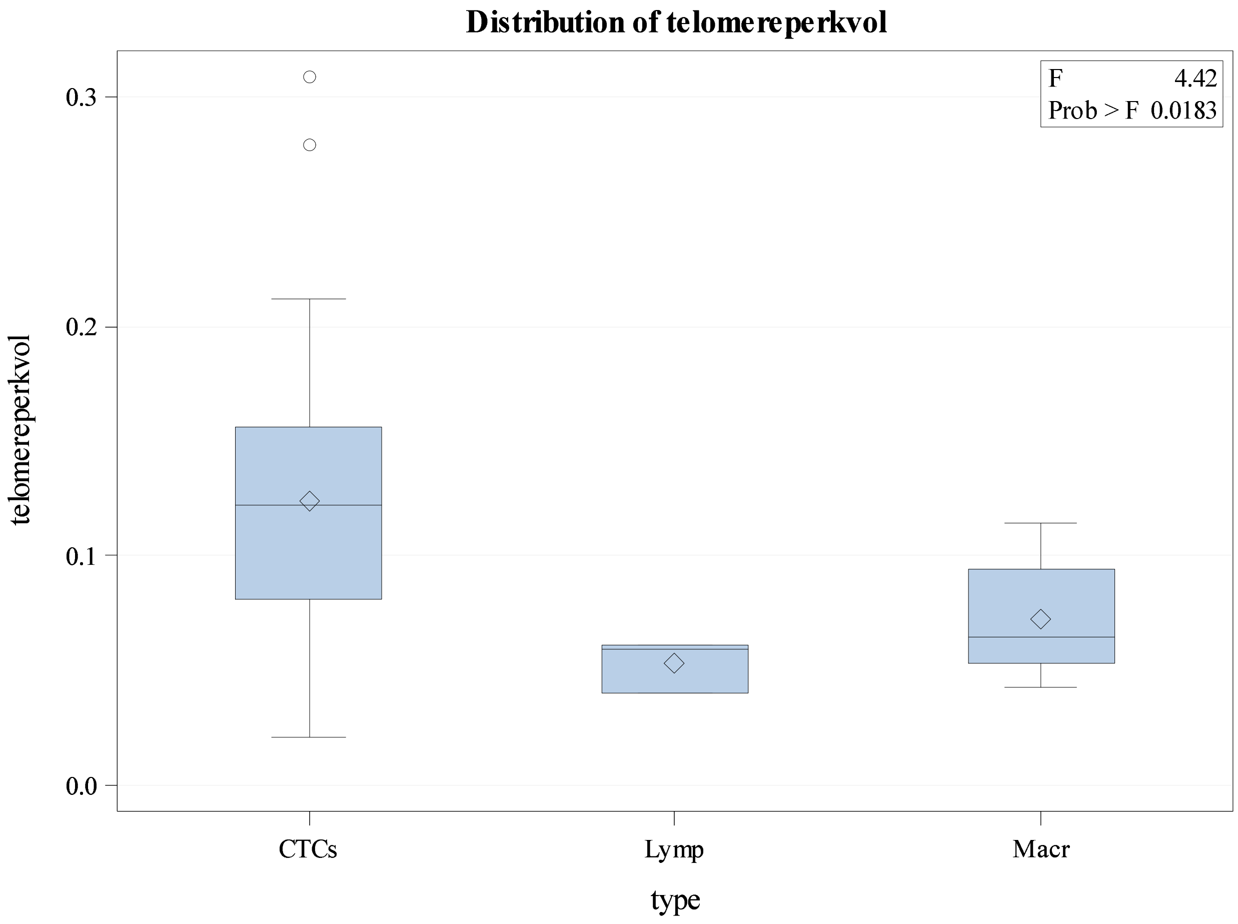

The GLM Procedure
Least Squares Means

pt=16AA8528

type	Totalnofsignals LSMEAN	Standard Error	Pr > |t|	LSMEAN Number
**CTCs**	117.758621	11.227003	<.0001	1
**Lymp**	23.000000	34.906172	0.5136	2
**Macr**	29.916667	17.453086	0.0941	3

Least Squares Means for effect type<br>Pr > |t| for H0: LSMean(i)=LSMean(j)<br><br>Dependent Variable: Totalnofsignals			
**i/j**	**1**	**2**	**3**
**1**		0.0134	0.0001
**2**	0.0134		0.8602
**3**	0.0001	0.8602	

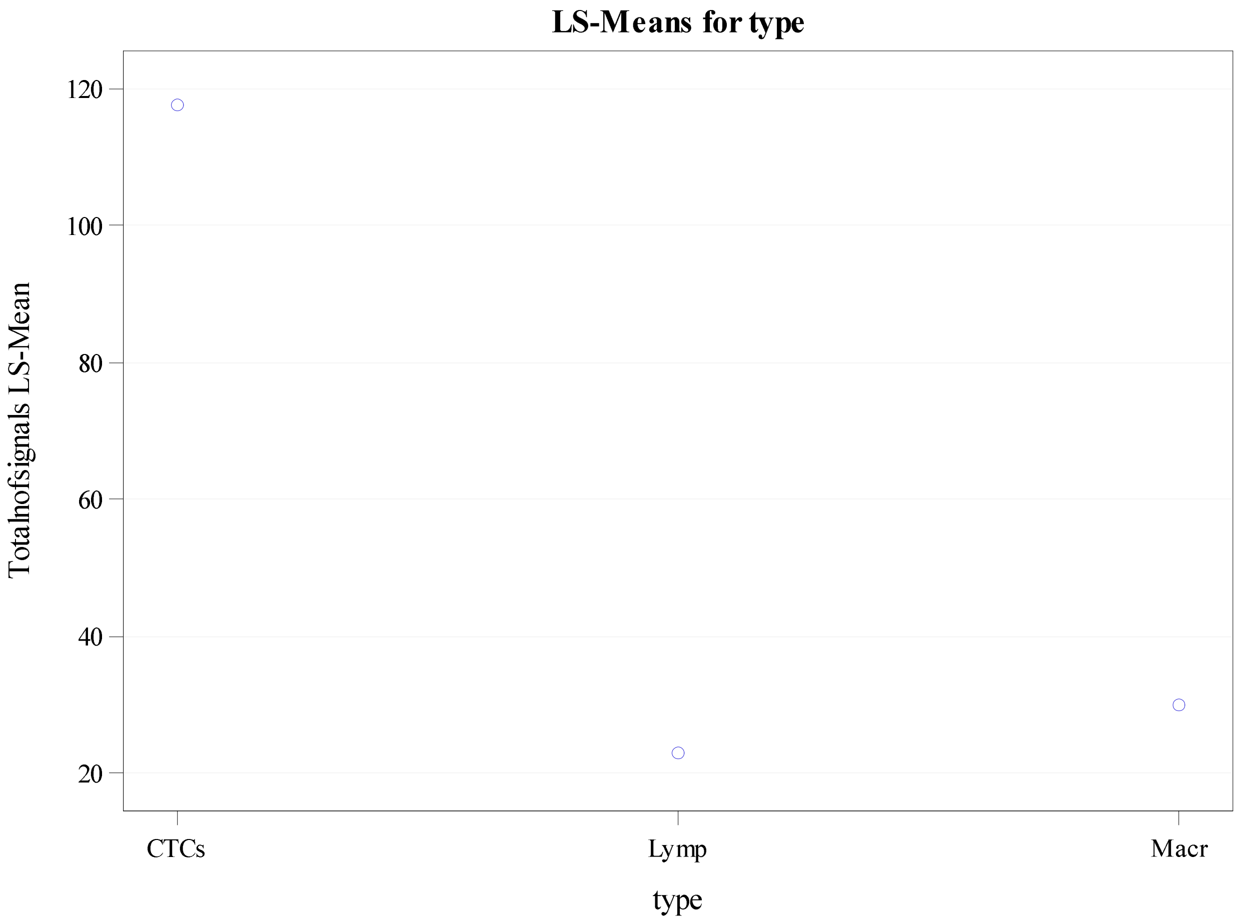

The GLM Procedure
Least Squares Means

pt=16AA8528

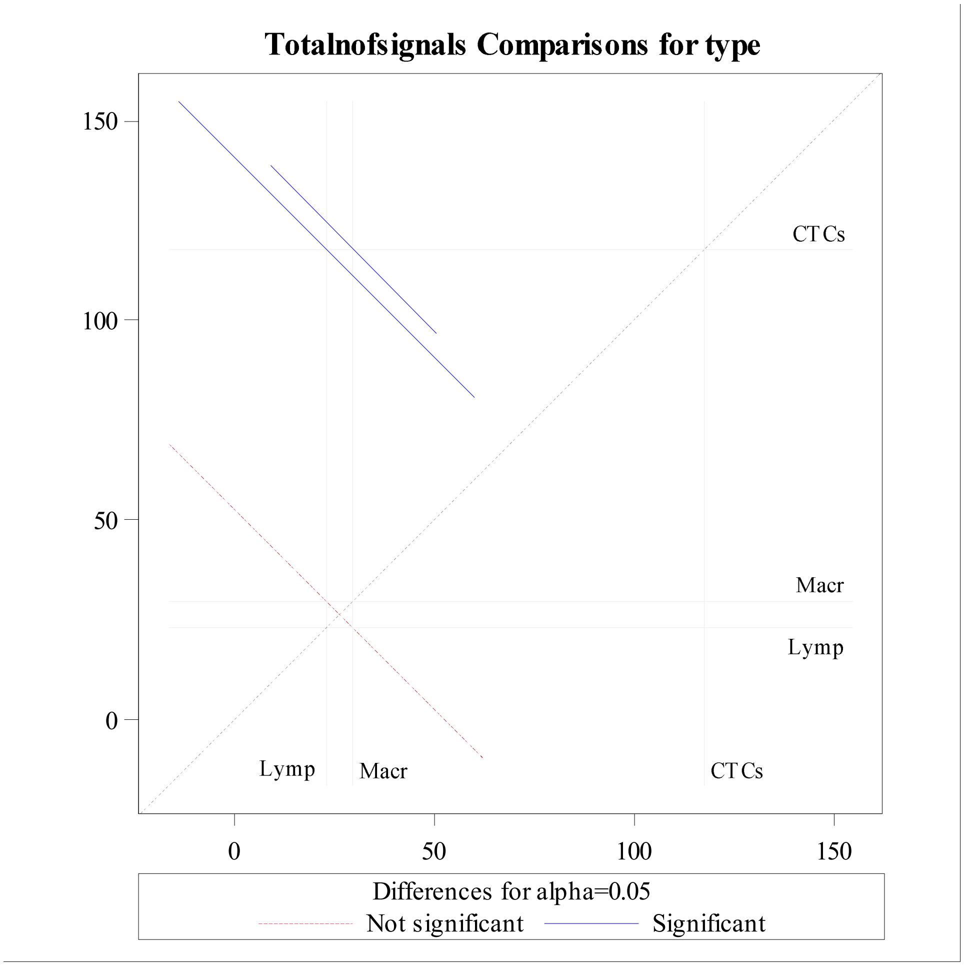

type	Totalnofaggregates LSMEAN	Standard Error	Pr > |t|	LSMEAN Number
**CTCs**	14.0000000	1.5168200	<.0001	1
**Lymp**	0.6666667	4.7159850	0.8883	2
**Macr**	3.4166667	2.3579925	0.1550	3

The GLM Procedure
Least Squares Means

pt=16AA8528

**Least Squares Means for effect type<br>Pr > |t| for H0: LSMean(i)=LSMean(j)<br><br>Dependent Variable: Totalnofaggregates**			
**i/j**	**1**	**2**	**3**
**1**		0.0102	0.0005
**2**	0.0102		0.6048
**3**	0.0005	0.6048	

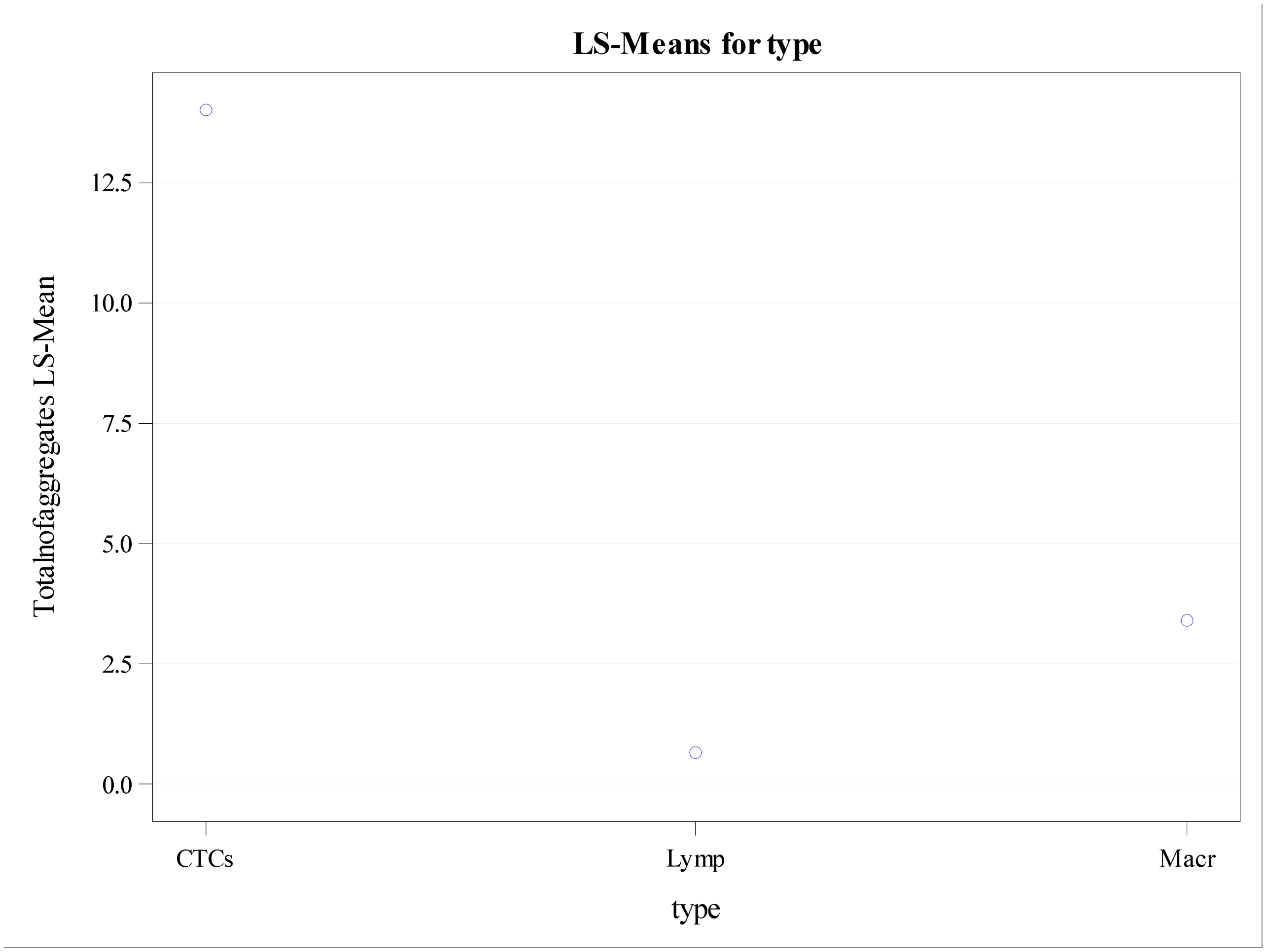

The GLM Procedure
Least Squares Means

pt=16AA8528

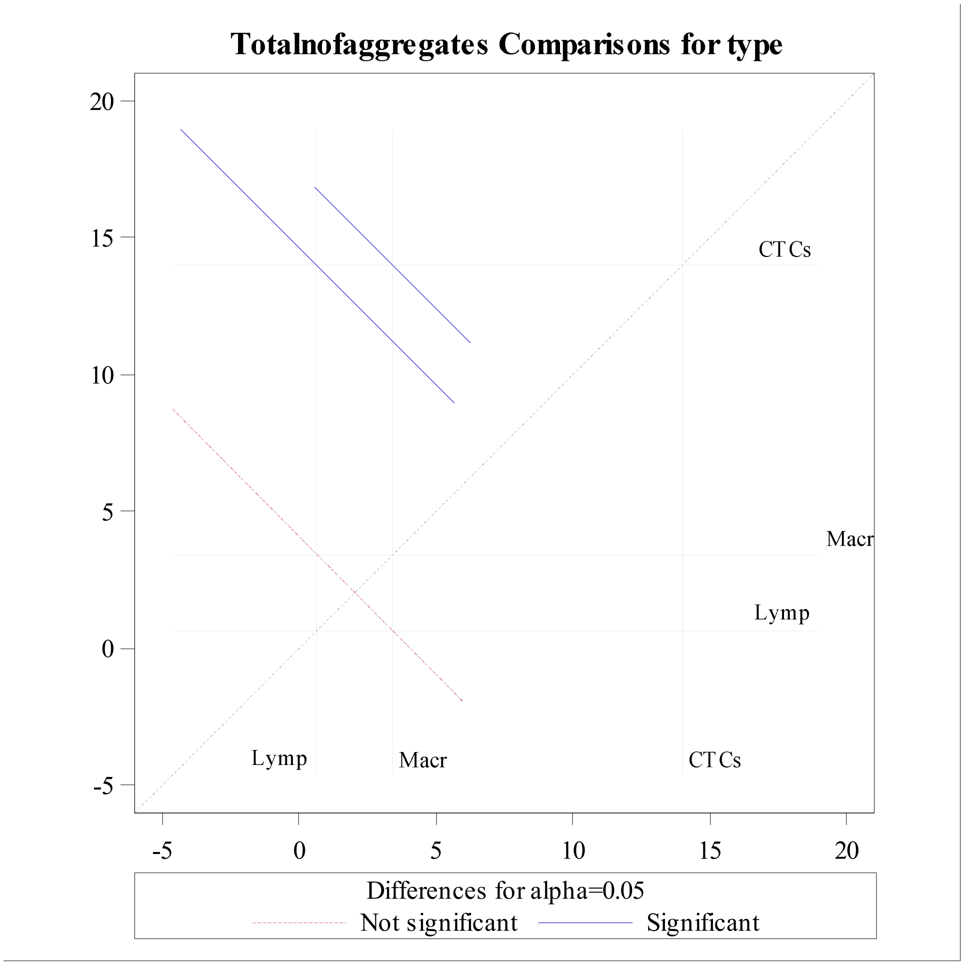

type	acratio LSMEAN	Standard Error	Pr > |t|	LSMEAN Number
**CTCs**	3.69249093	0.50355299	<.0001	1
**Lymp**	4.72203744	1.56560988	0.0044	2
**Macr**	4.18776994	0.78280494	<.0001	3

Least Squares Means for effect type
Pr > |t| for H0: LSMean(i)=LSMean(j)

Dependent Variable: acratio

i/j	1	2	3
**1**		0.5348	0.5975
**2**	0.5348		0.7617
**3**	0.5975	0.7617	

The GLM Procedure
Least Squares Means

pt=16AA8528

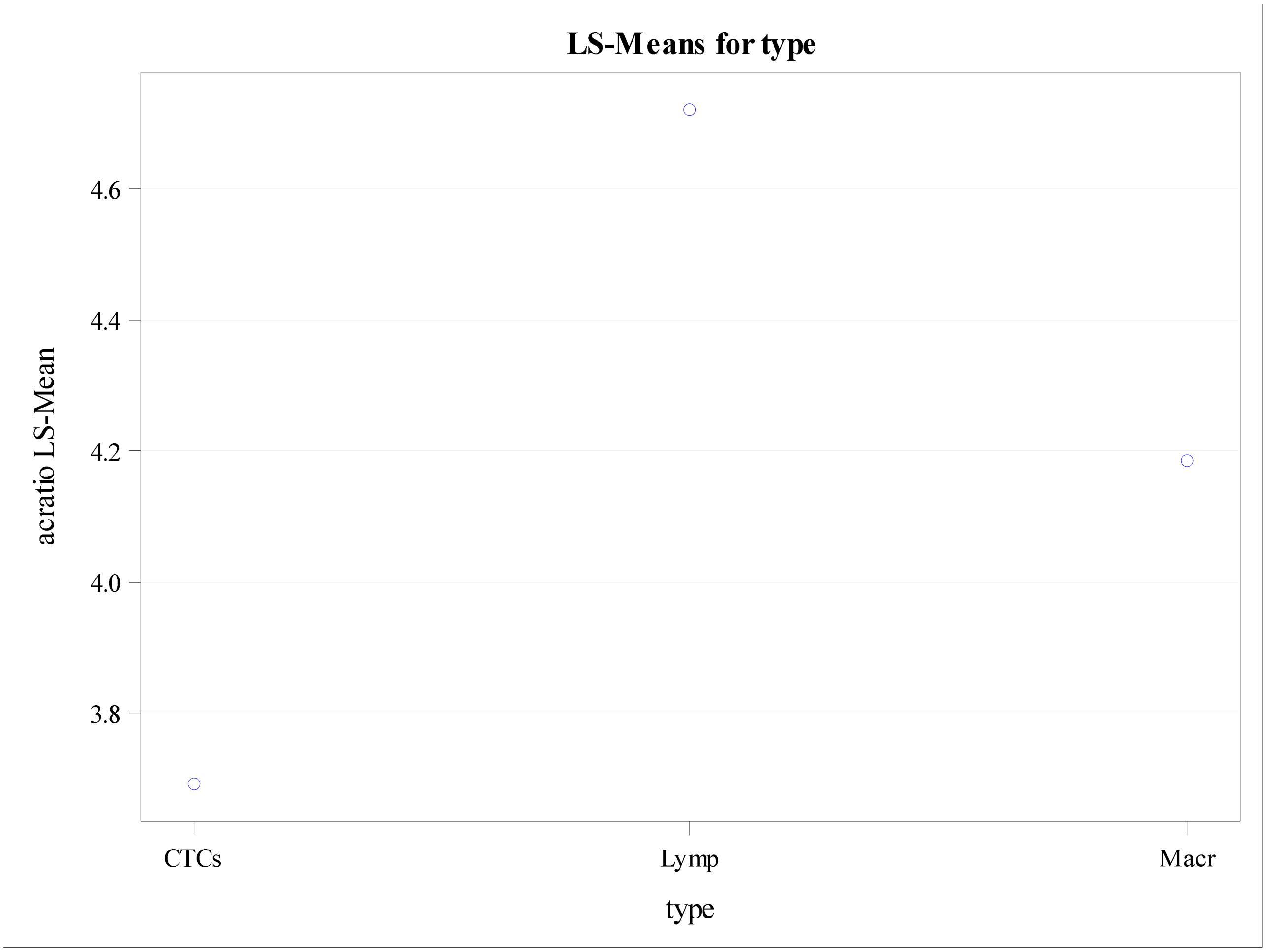

The GLM Procedure
Least Squares Means

pt=16AA8528

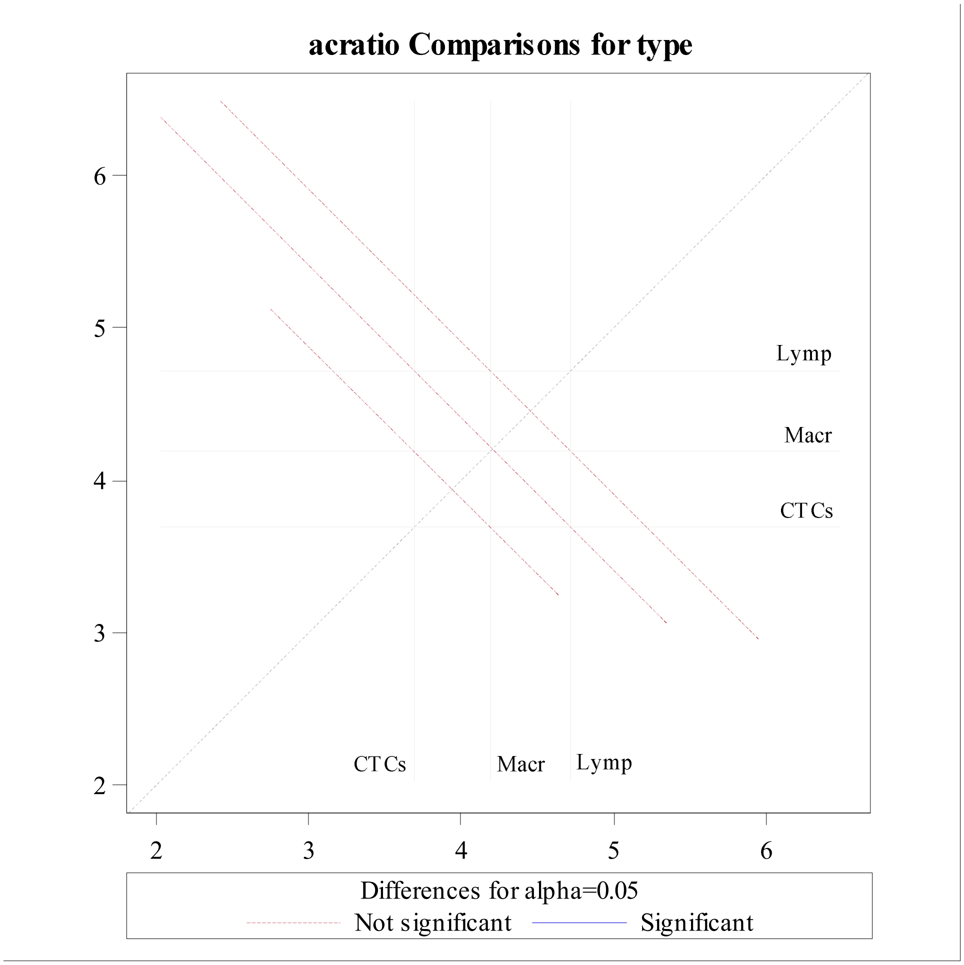

type	AvIntallsignals LSMEAN	Standard Error	Pr > |t|	LSMEAN Number
**CTCs**	10980.7677	479.8472	<.0001	1
**Lymp**	16235.4497	1491.9057	<.0001	2
**Macr**	12339.2143	745.9528	<.0001	3

Least Squares Means for effect type<br>Pr > |t| for H0: LSMean(i)=LSMean(j)<br><br>Dependent Variable: AvIntallsignals			
**i/j**	**1**	**2**	**3**
**1**		0.0017	0.1333
**2**	0.0017		0.0245
**3**	0.1333	0.0245	

The GLM Procedure
Least Squares Means

pt=16AA8528

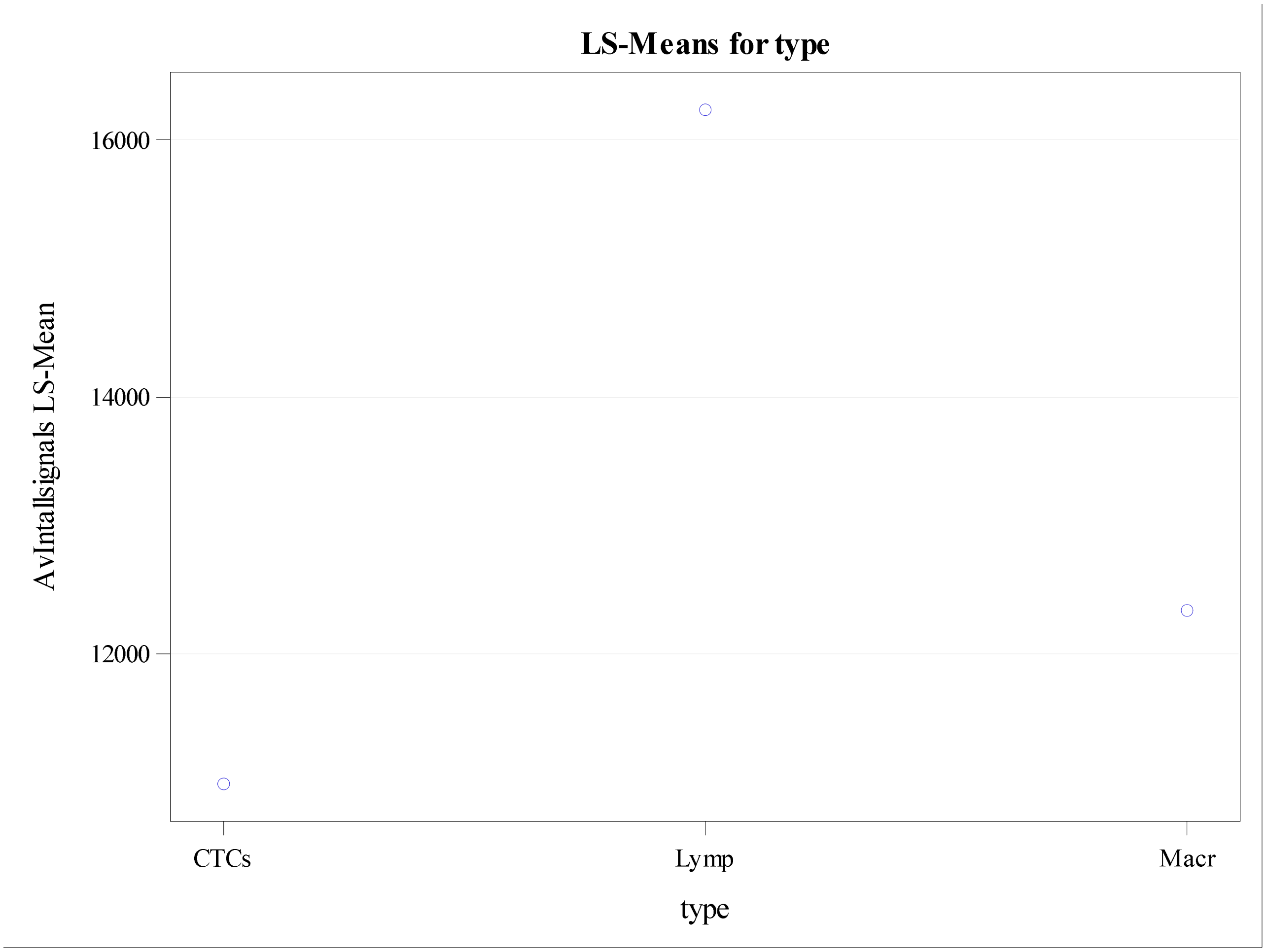

The GLM Procedure
Least Squares Means

pt=16AA8528

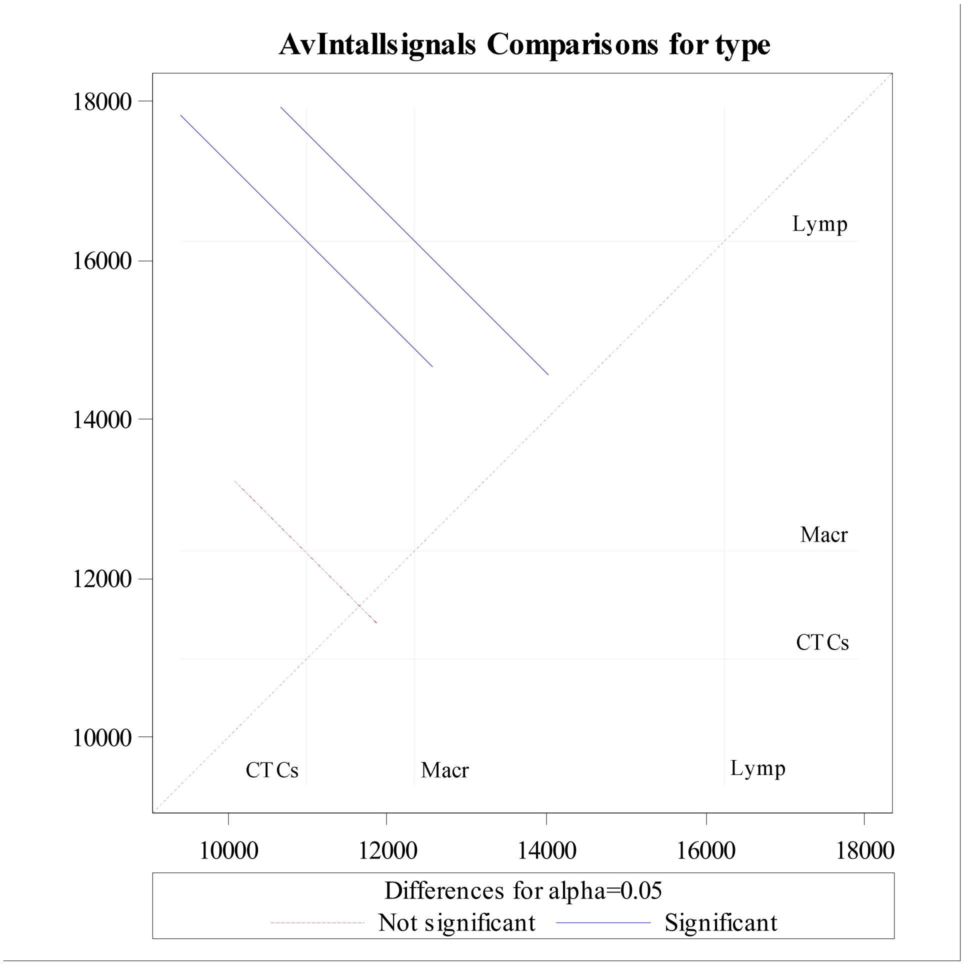

type	Totalintensity LSMEAN	Standard Error	Pr > |t|	LSMEAN Number
**CTCs**	1241392.45	111532.91	<.0001	1
**Lymp**	379418.33	346769.92	0.2803	2
**Macr**	365460.83	173384.96	0.0412	3

Least Squares Means for effect type<br>Pr > |t| for H0: LSMean(i)=LSMean(j)<br><br>Dependent Variable: Totalintensity			
**i/j**	**1**	**2**	**3**
**1**		0.0228	0.0001
**2**	0.0228		0.9715
**3**	0.0001	0.9715	

The GLM Procedure
Least Squares Means

pt=16AA8528

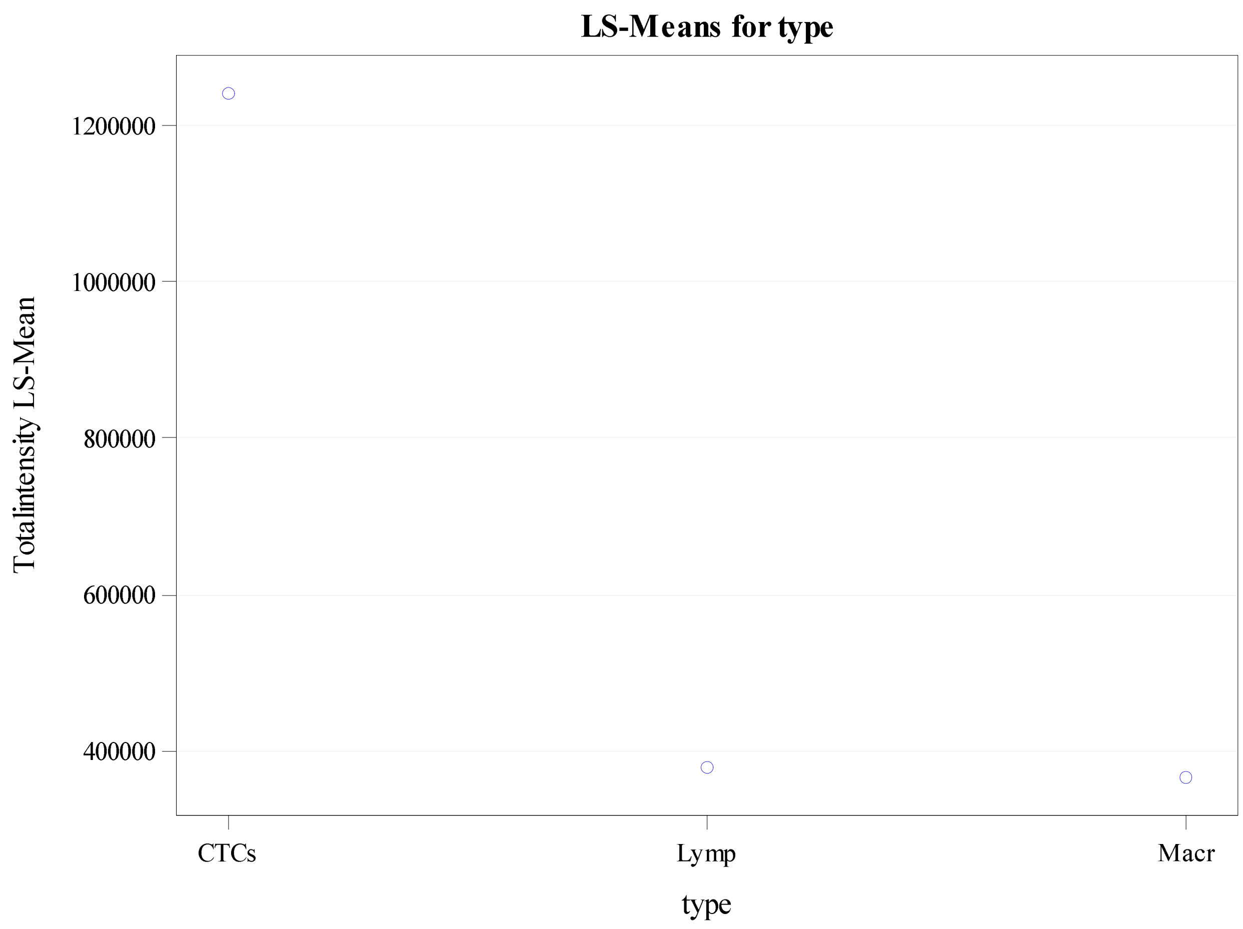

The GLM Procedure
Least Squares Means

pt=16AA8528

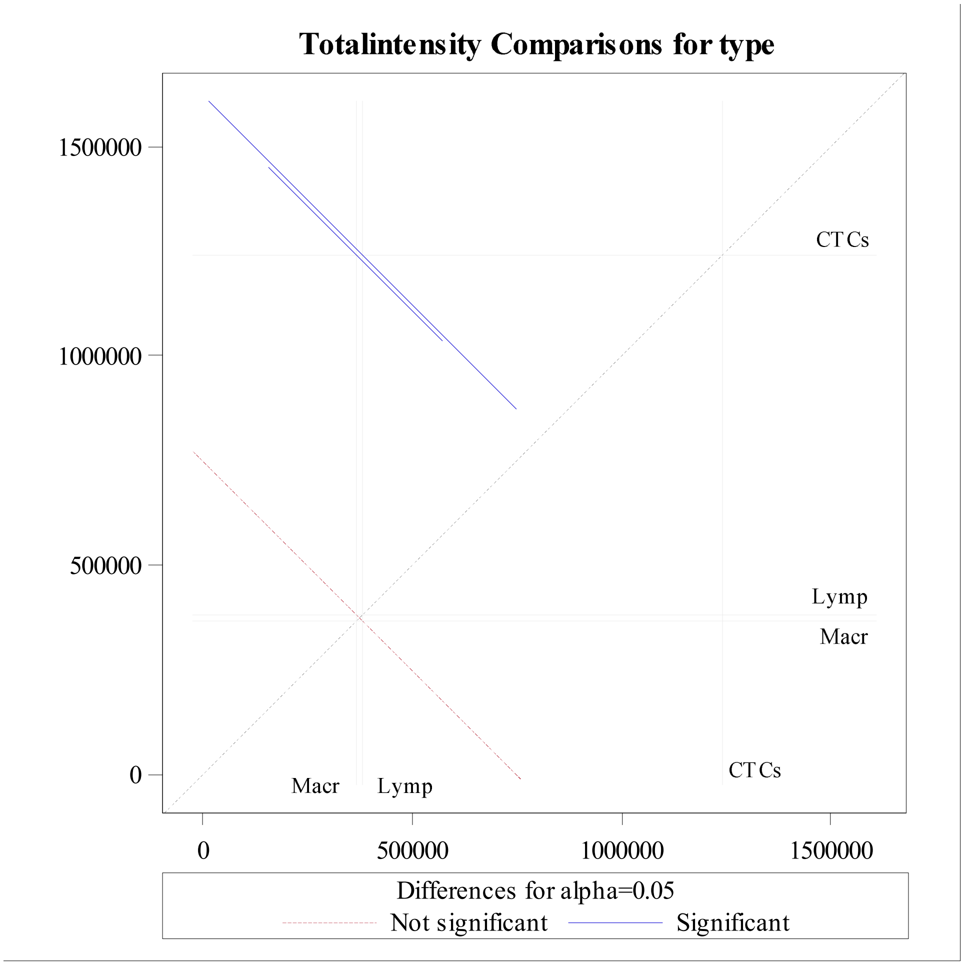

type	Nuclearvolume LSMEAN	Standard Error	Pr > |t|	LSMEAN Number
**CTCs**	963410.207	63320.745	<.0001	1
**Lymp**	441620.333	196872.197	0.0304	2
**Macr**	458009.333	98436.099	<.0001	3

Least Squares Means for effect type<br>Pr > |t| for H0: LSMean(i)=LSMean(j)<br><br>Dependent Variable: Nuclearvolume			
**i/j**	**1**	**2**	**3**
**1**		0.0156	<.0001
**2**	0.0156		0.9410
**3**	<.0001	0.9410	

The GLM Procedure
Least Squares Means

pt=16AA8528

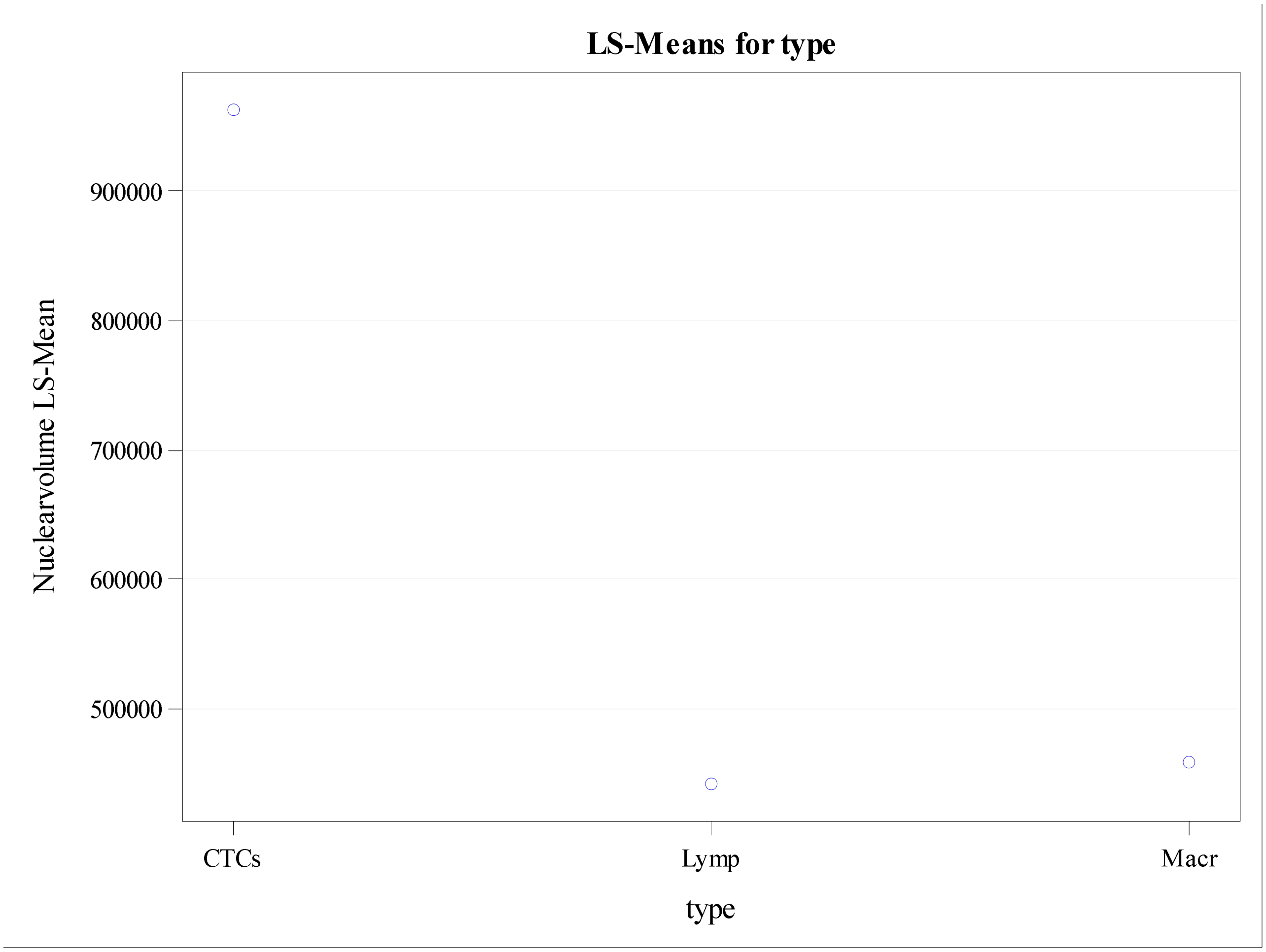

The GLM Procedure
Least Squares Means

pt=16AA8528

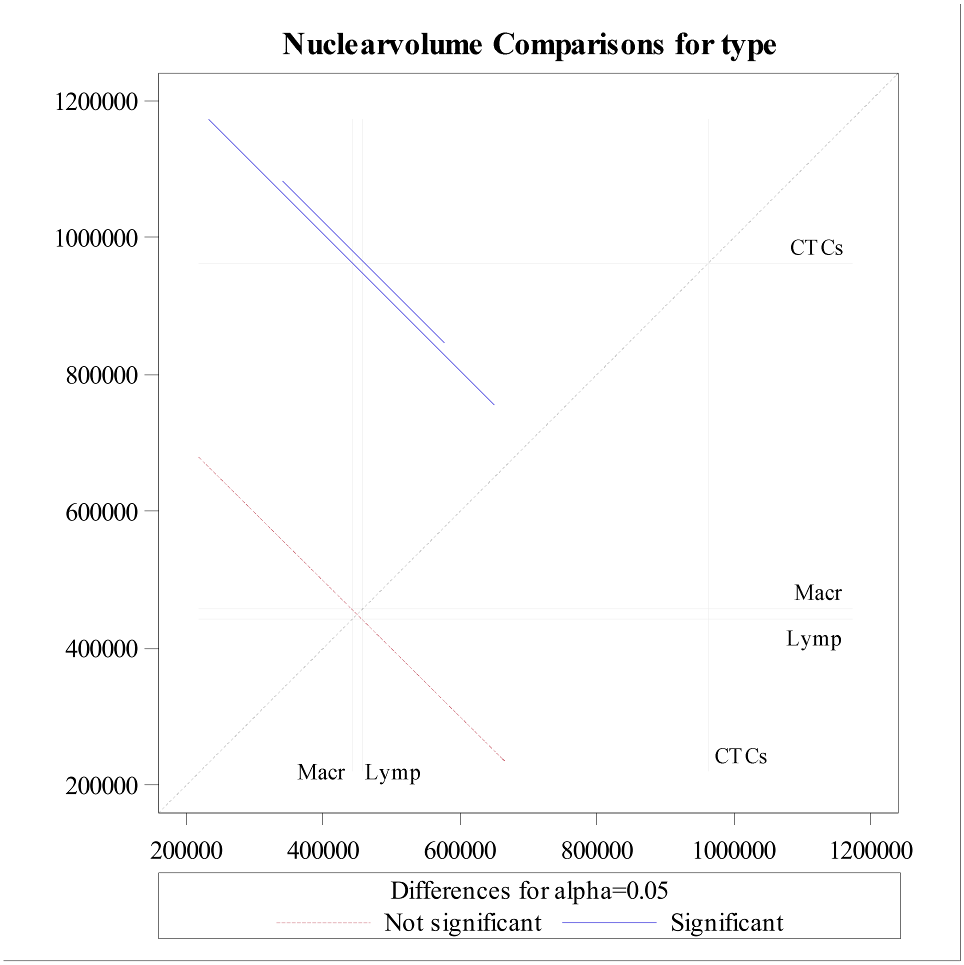

type	telomereperkvol LSMEAN	Standard Error	Pr > |t|	LSMEAN Number
**CTCs**	0.12343677	0.01089810	<.0001	1
**Lymp**	0.05360820	0.03388358	0.1213	2
**Macr**	0.07251796	0.01694179	0.0001	3

Least Squares Means for effect type<br>Pr > |t| for H0: LSMean(i)=LSMean(j)<br><br>Dependent Variable: telomereperkvol			
**i/j**	**1**	**2**	**3**
**1**		0.0566	0.0154
**2**	0.0566		0.6203
**3**	0.0154	0.6203	

The GLM Procedure
Least Squares Means

pt=16AA8528

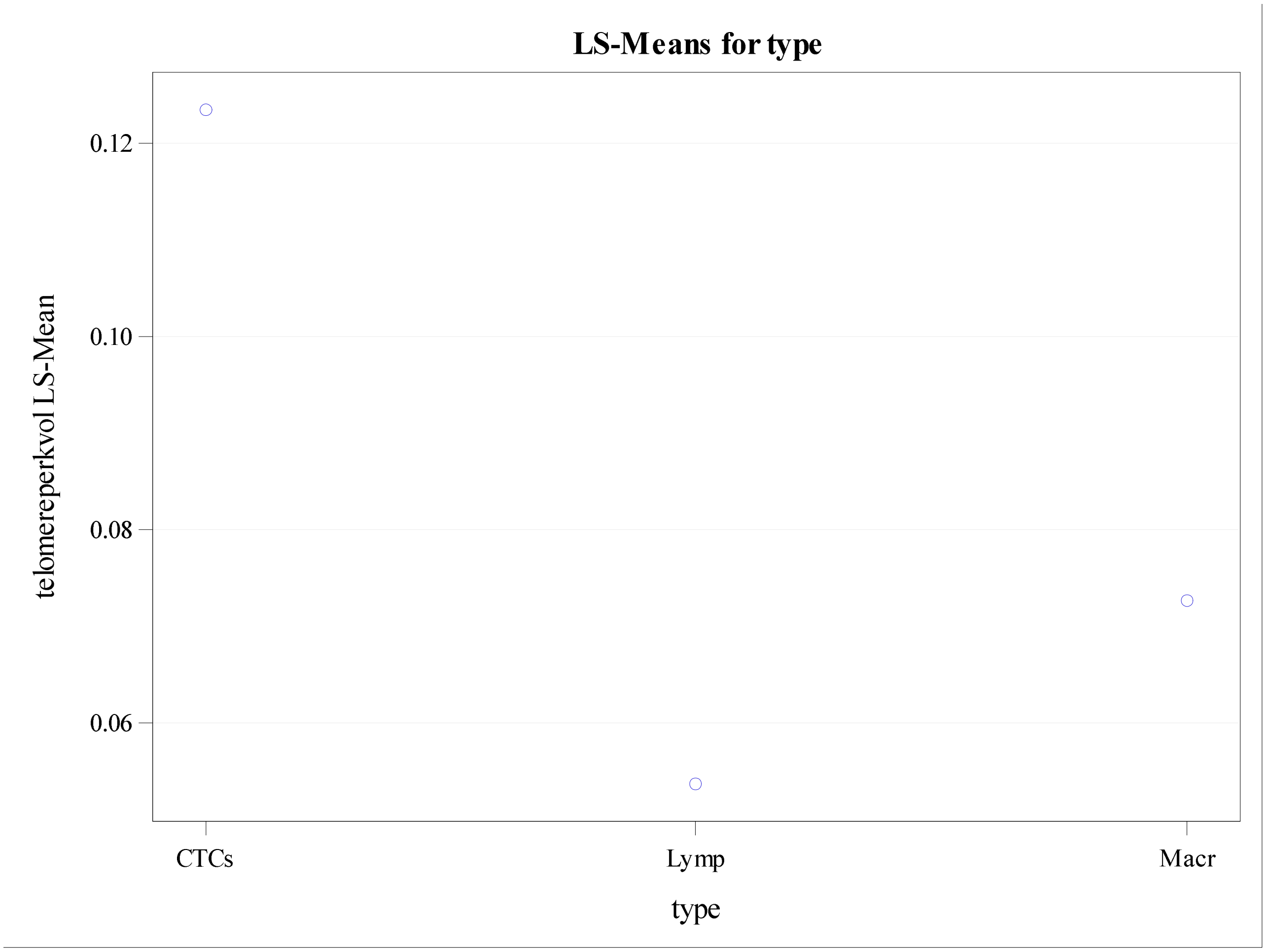

The GLM Procedure
Least Squares Means

pt=16AA8528

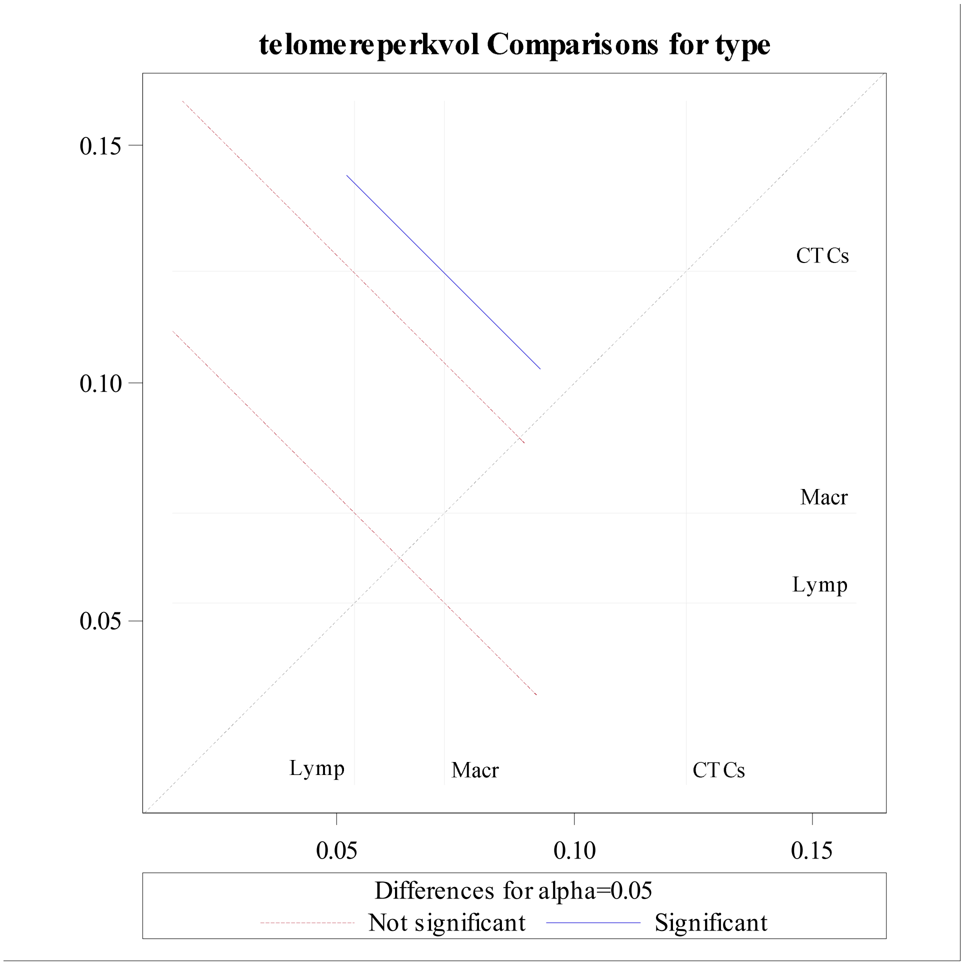

Note: To ensure overall protection level, only probabilities associated with pre-planned comparisons should be used.

The GLM Procedure

pt=16AB0258

Class Level Information		
**Class**	**Levels**	**Values**
**type**	3	CTCs Lymp Macr

**Number of Observations Read**	24
**Number of Observations Used**	24

The GLM Procedure

Dependent Variable: Totalnofsignals Totalnofsignals

pt=16AB0258

Source	DF	Sum of Squares	Mean Square	F Value	Pr > F
**Model**	2	73685.7250	36842.8625	12.36	0.0003
**Error**	21	62609.2333	2981.3921		
**Corrected Total**	23	136294.9583			

R-Square	Coeff Var	Root MSE	Totalnofsignals Mean
0.540634	81.44506	54.60213	67.04167

Source	DF	Type III SS	Mean Square	F Value	Pr > F
**type**	2	73685.72500	36842.86250	12.36	0.0003

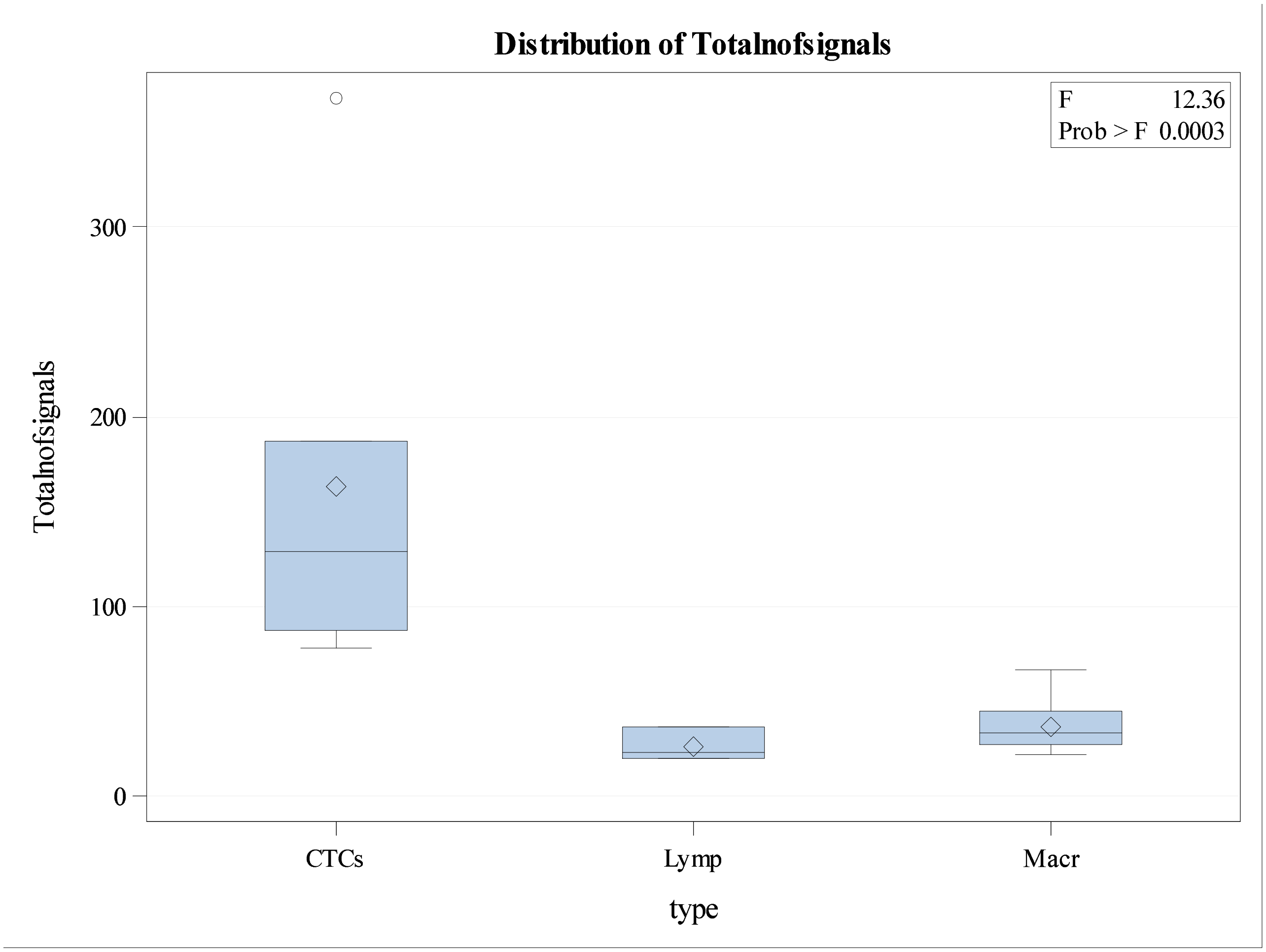

The GLM Procedure

Dependent Variable: Totalnofaggregates Totalnofaggregates

pt=16AB0258

Source	DF	Sum of Squares	Mean Square	F Value	Pr > F
**Model**	2	1748.458333	874.229167	11.84	0.0004
**Error**	21	1550.166667	73.817460		
**Corrected Total**	23	3298.625000			

R-Square	Coeff Var	Root MSE	Totalnofaggregates Mean
0.530057	105.7441	8.591709	8.125000

Source	DF	Type III SS	Mean Square	F Value	Pr > F
**type**	2	1748.458333	874.229167	11.84	0.0004

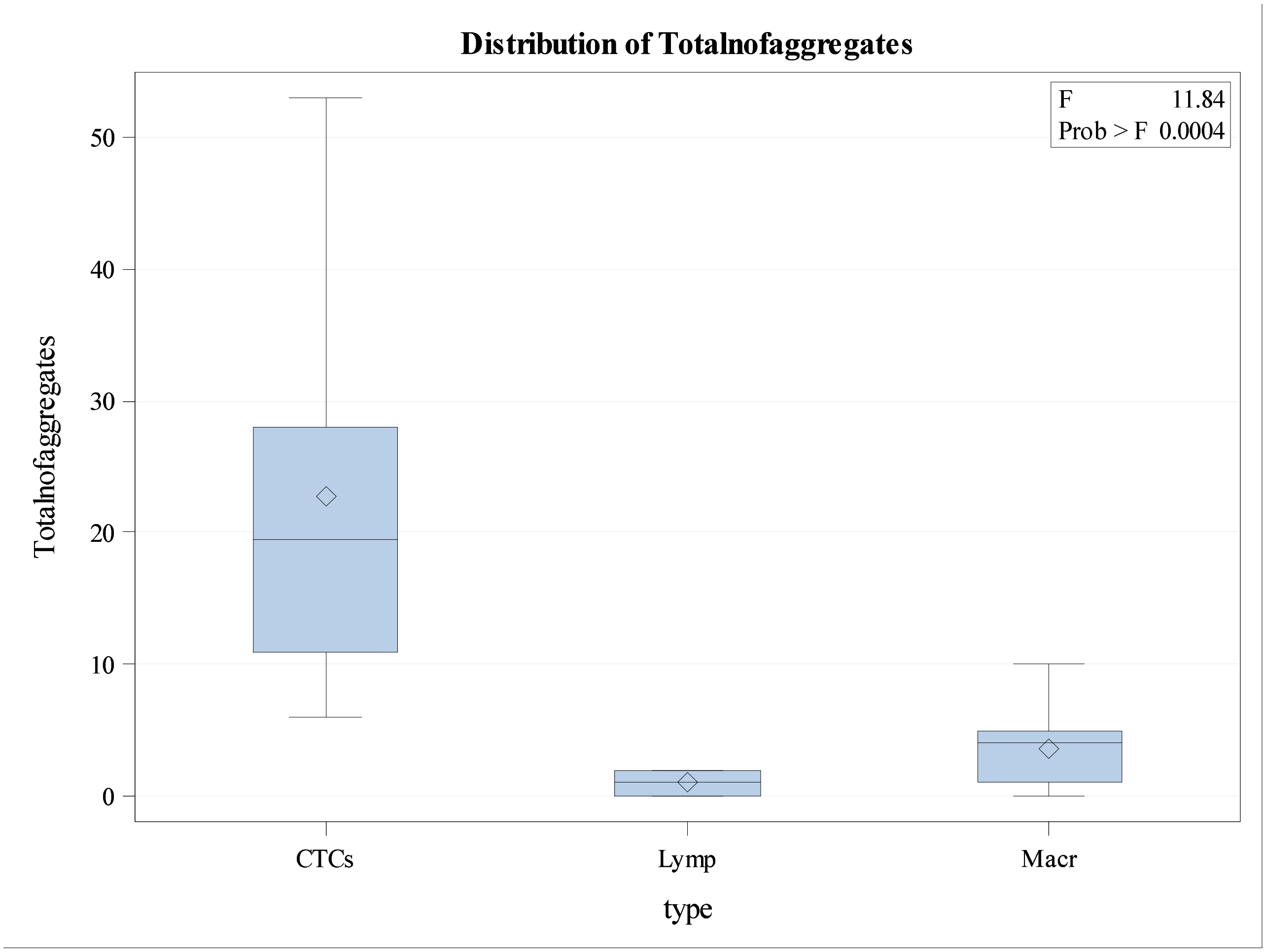

The GLM Procedure

Dependent Variable: acratio
acratio

pt=16AB0258

Source	DF	Sum of Squares	Mean Square	F Value	Pr > F
**Model**	2	13.31176276	6.65588138	4.87	0.0182
**Error**	21	28.67450926	1.36545282		
**Corrected Total**	23	41.98627202			

R-Square	Coeff Var	Root MSE	acratio Mean
0.317050	33.30396	1.168526	3.508669

Source	DF	Type III SS	Mean Square	F Value	Pr > F
**type**	2	13.31176276	6.65588138	4.87	0.0182

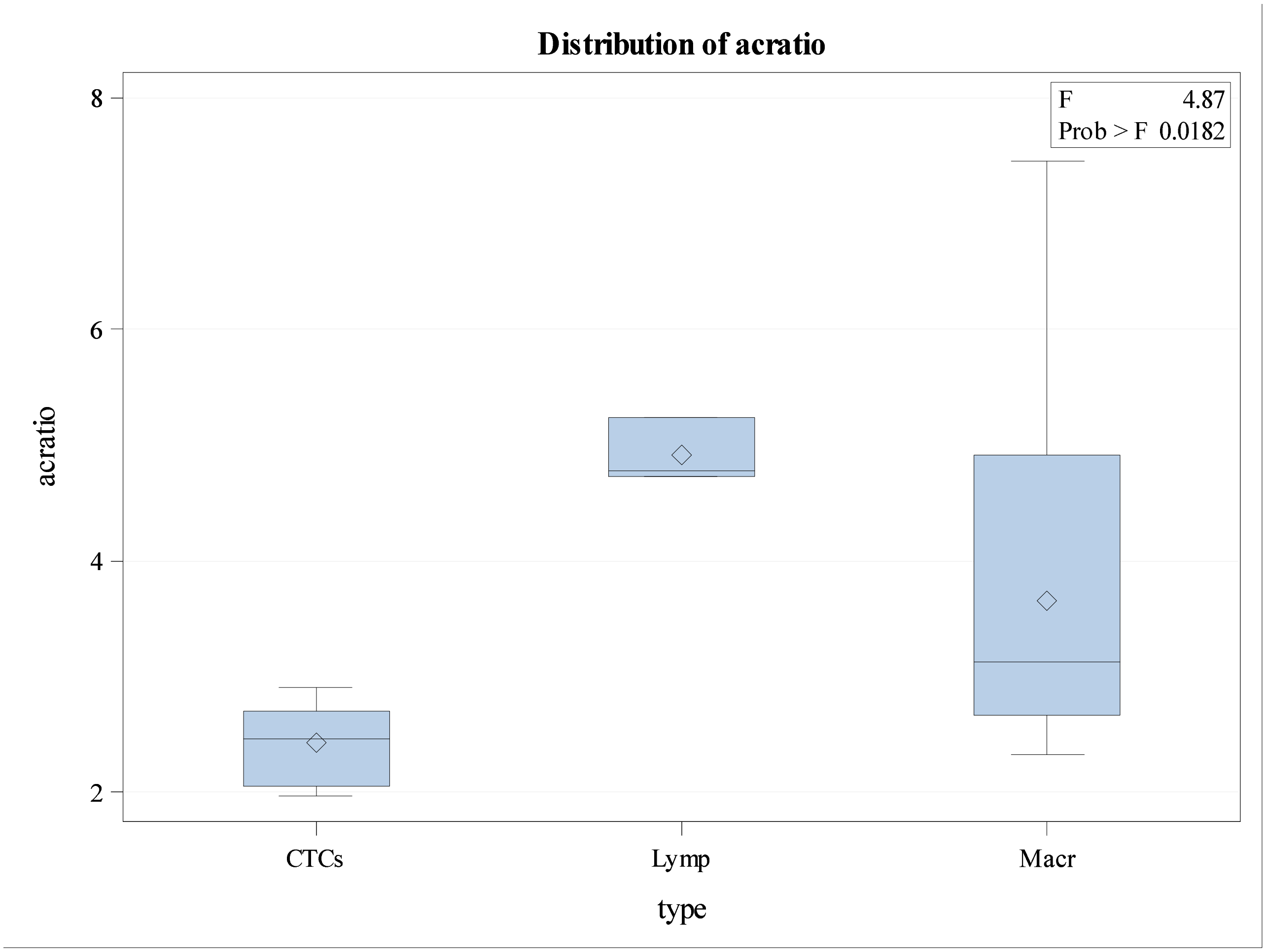

The GLM Procedure

Dependent Variable: AvIntallsignals
AvIntallsignals

pt=16AB0258

Source	DF	Sum of Squares	Mean Square	F Value	Pr > F
**Model**	2	92405509.9	46202754.9	2.65	0.0939
**Error**	21	365667191.1	17412723.4		
**Corrected Total**	23	458072700.9			

R-Square	Coeff Var	Root MSE	AvIntallsignals Mean
0.201727	32.23082	4172.856	12946.78

Source	DF	Type III SS	Mean Square	F Value	Pr > F
**type**	2	92405509.86	46202754.93	2.65	0.0939

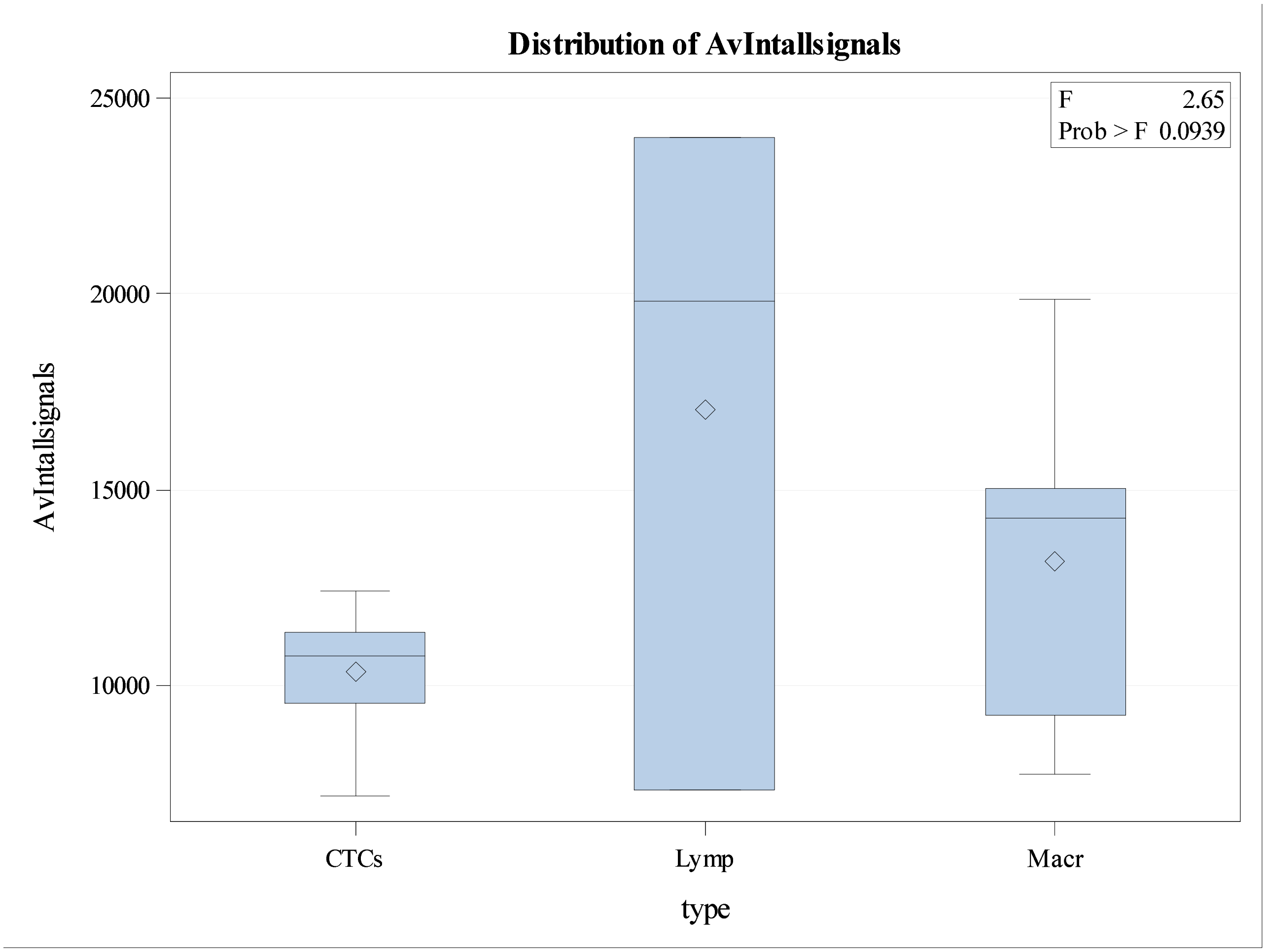

The GLM Procedure

Dependent Variable: Totalintensity
Totalintensity

pt=16AB0258

Source	DF	Sum of Squares	Mean Square	F Value	Pr > F
**Model**	2	5.58625E12	2.793125E12	16.79	<.0001
**Error**	21	3.4928964E12	166328401468		
**Corrected Total**	23	9.0791464E12			

R-Square	Coeff Var	Root MSE	Totalintensity Mean
0.615284	55.25344	407833.8	738114.8

Source	DF	Type III SS	Mean Square	F Value	Pr > F
**type**	2	5.58625E12	2.793125E12	16.79	<.0001

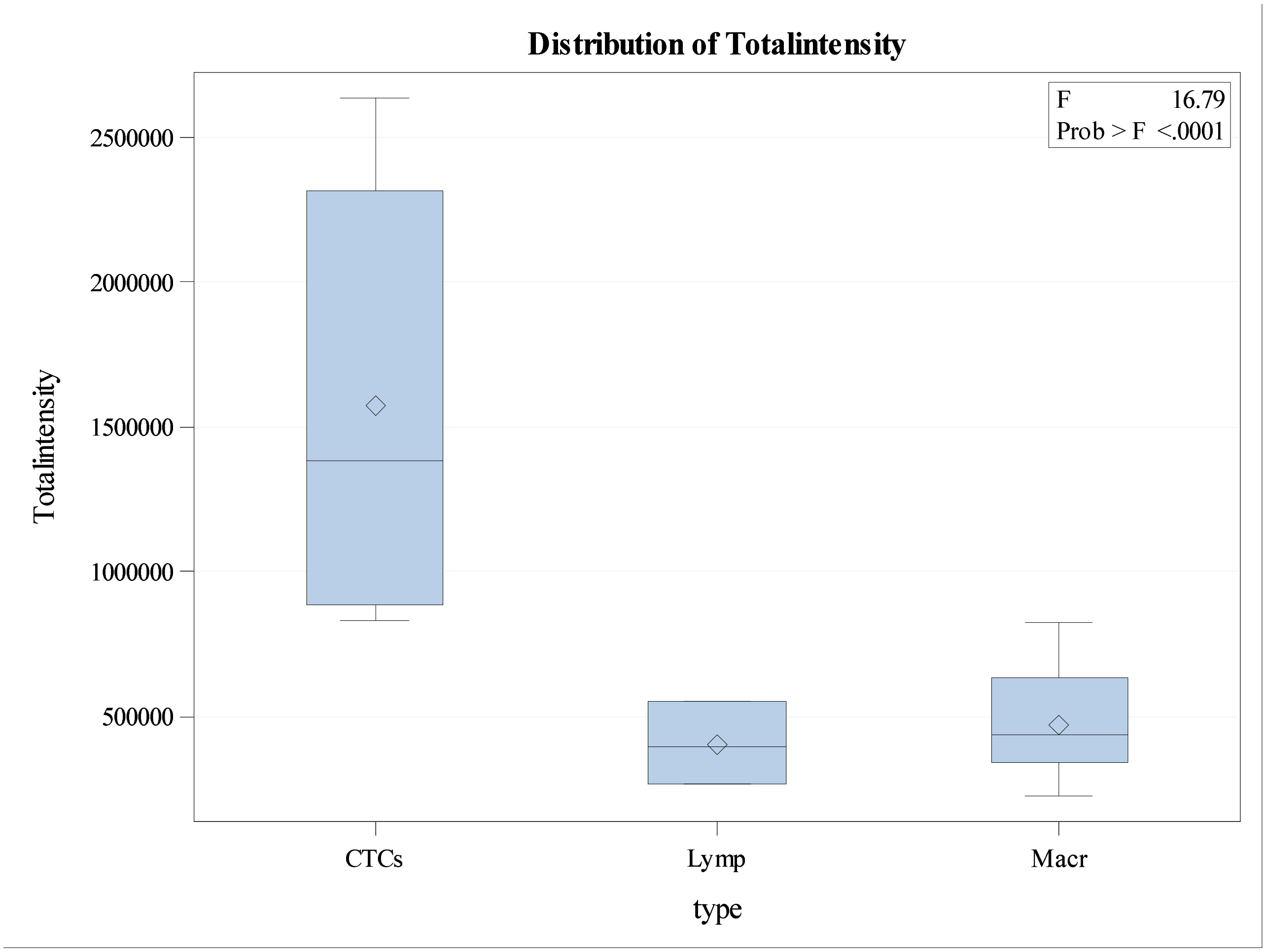

The GLM Procedure

Dependent Variable: Nuclearvolume Nuclearvolume

pt=16AB0258

Source	DF	Sum of Squares	Mean Square	F Value	Pr > F
**Model**	2	1.4984471E12	749223558400	12.48	0.0003
**Error**	21	1.2610145E12	60048311390		
**Corrected Total**	23	2.7594617E12			

R-Square	Coeff Var	Root MSE	Nuclearvolume Mean
0.543022	39.74086	245047.6	616613.7

Source	DF	Type III SS	Mean Square	F Value	Pr > F
**type**	2	1.4984471E12	749223558400	12.48	0.0003

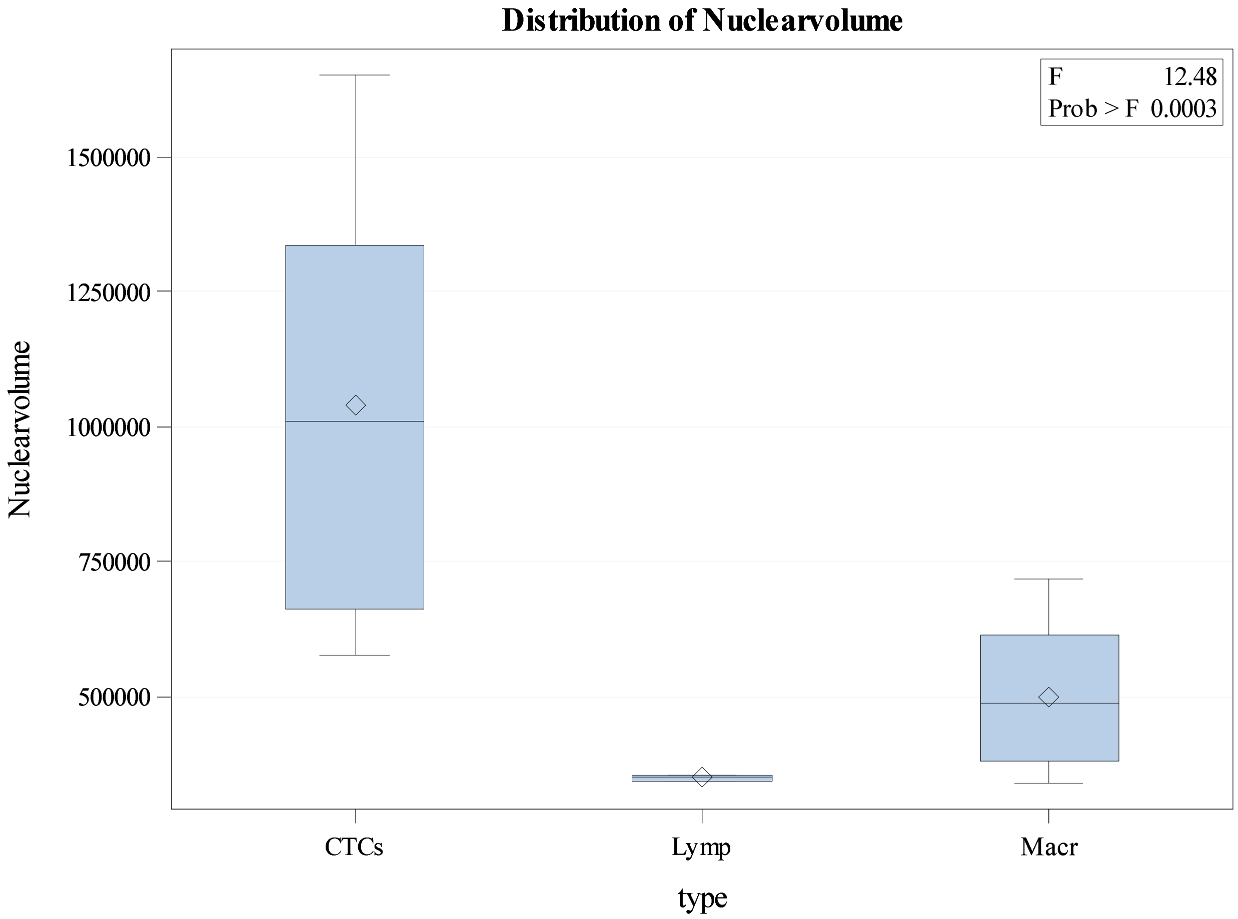

The GLM Procedure

Dependent Variable: telomereperkvol

pt=16AB0258

Source	DF	Sum of Squares	Mean Square	F Value	Pr > F
**Model**	2	0.02501614	0.01250807	21.02	<.0001
**Error**	21	0.01249744	0.00059512		
**Corrected Total**	23	0.03751359			

R-Square	Coeff Var	Root MSE	telomereperkvol Mean
0.666856	26.35463	0.024395	0.092564

Source	DF	Type III SS	Mean Square	F Value	Pr > F
**type**	2	0.02501614	0.01250807	21.02	<.0001

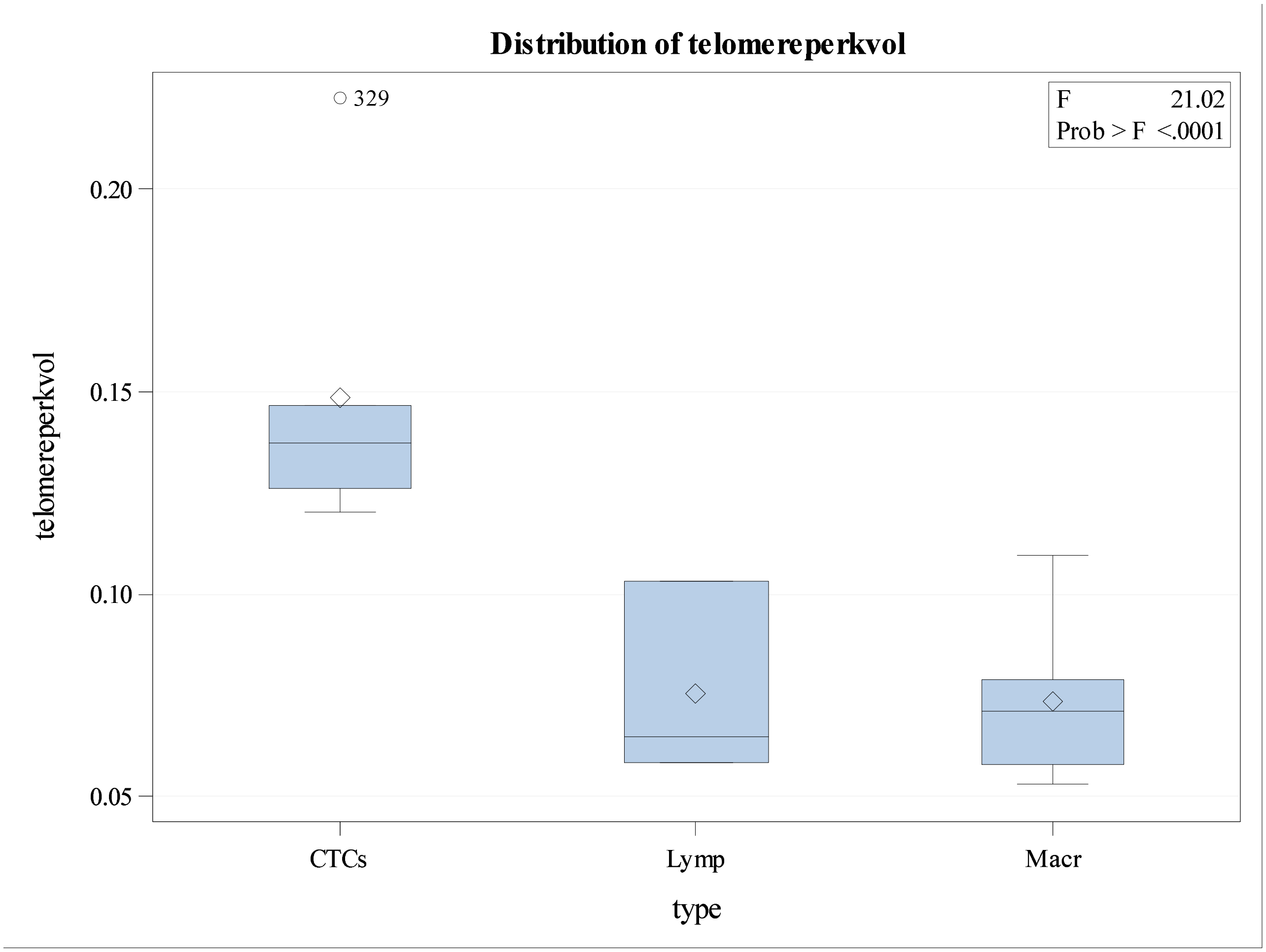

The GLM Procedure
Least Squares Means

pt=16AB0258

type	Totalnofsignals LSMEAN	Standard Error	Pr > |t|	LSMEAN Number
**CTCs**	162.833333	22.291224	<.0001	1
**Lymp**	26.333333	31.524552	0.4129	2
**Macr**	36.866667	14.098208	0.0162	3

Least Squares Means for effect type
Pr > |t| for H0: LSMean(i)=LSMean(j)

Dependent Variable: Totalnofsignals

i/j	1	2	3
**1**		0.0020	0.0001
**2**	0.0020		0.7634
**3**	0.0001	0.7634	

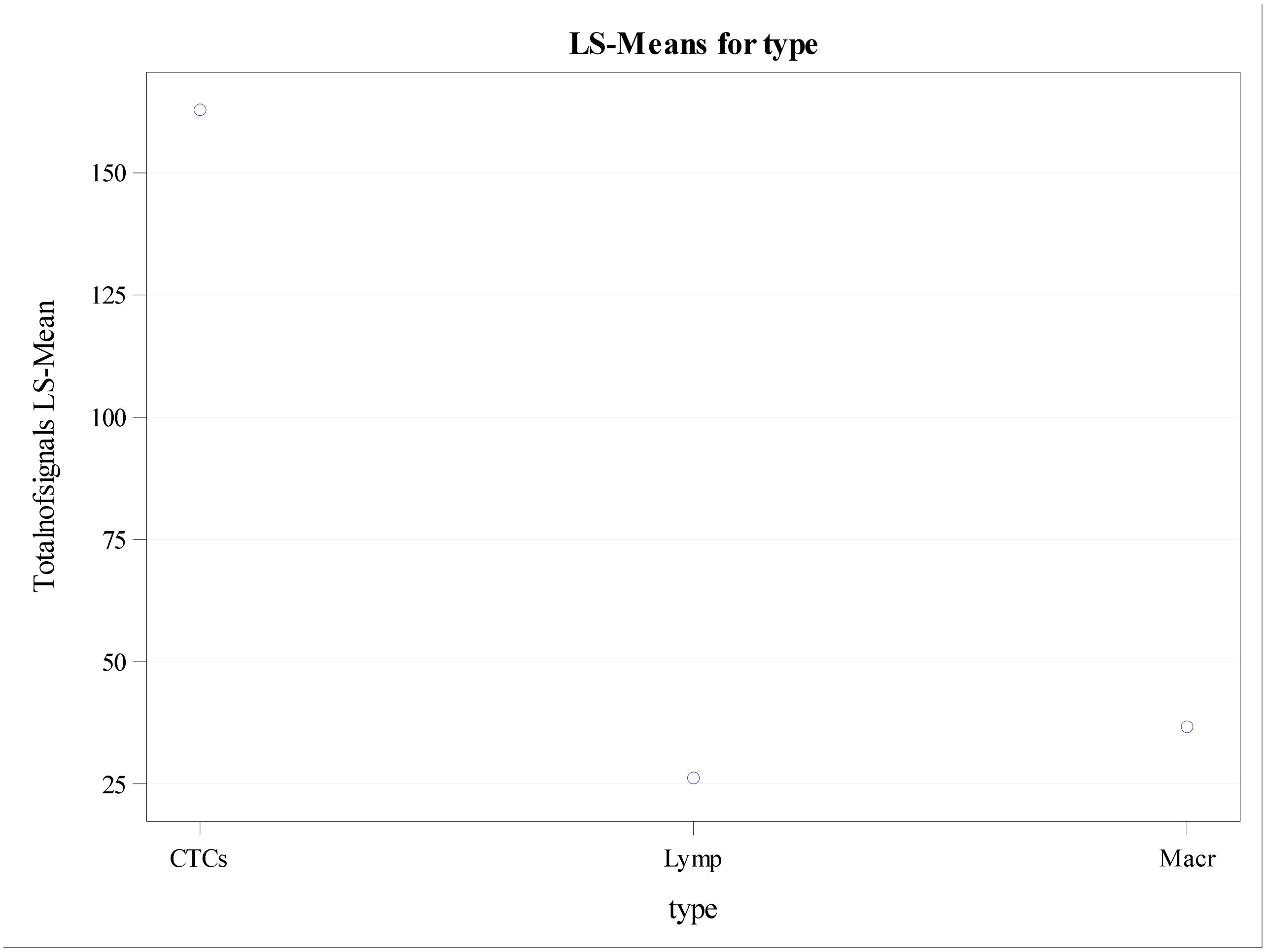

The GLM Procedure
Least Squares Means

pt=16AB0258

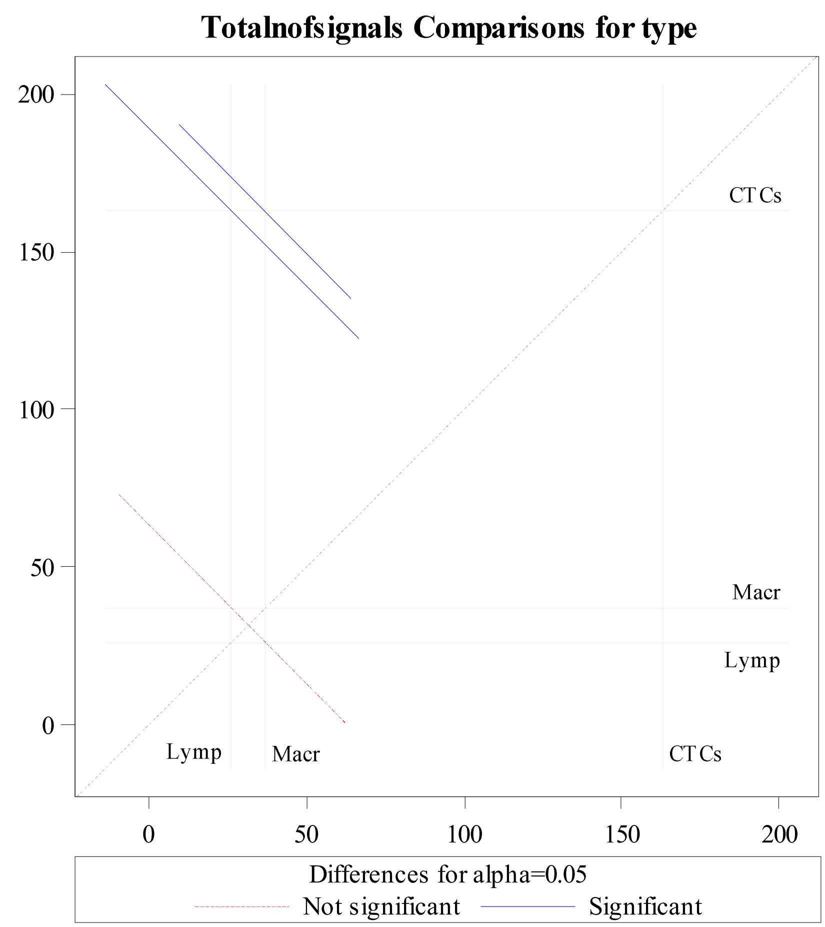

type	Totalnofaggregates LSMEAN	Standard Error	Pr > |t|	LSMEAN Number
**CTCs**	22.8333333	3.5075504	<.0001	1
**Lymp**	1.0000000	4.9604254	0.8422	2
**Macr**	3.6666667	2.2183697	0.1132	3

The GLM Procedure
Least Squares Means

pt=16AB0258

Least Squares Means for effect type<br>Pr > |t| for H0: LSMean(i)=LSMean(j)<br><br>Dependent Variable: Totalnofaggregates			
**i/j**	**1**	**2**	**3**
**1**		0.0017	0.0001
**2**	0.0017		0.6287
**3**	0.0001	0.6287	

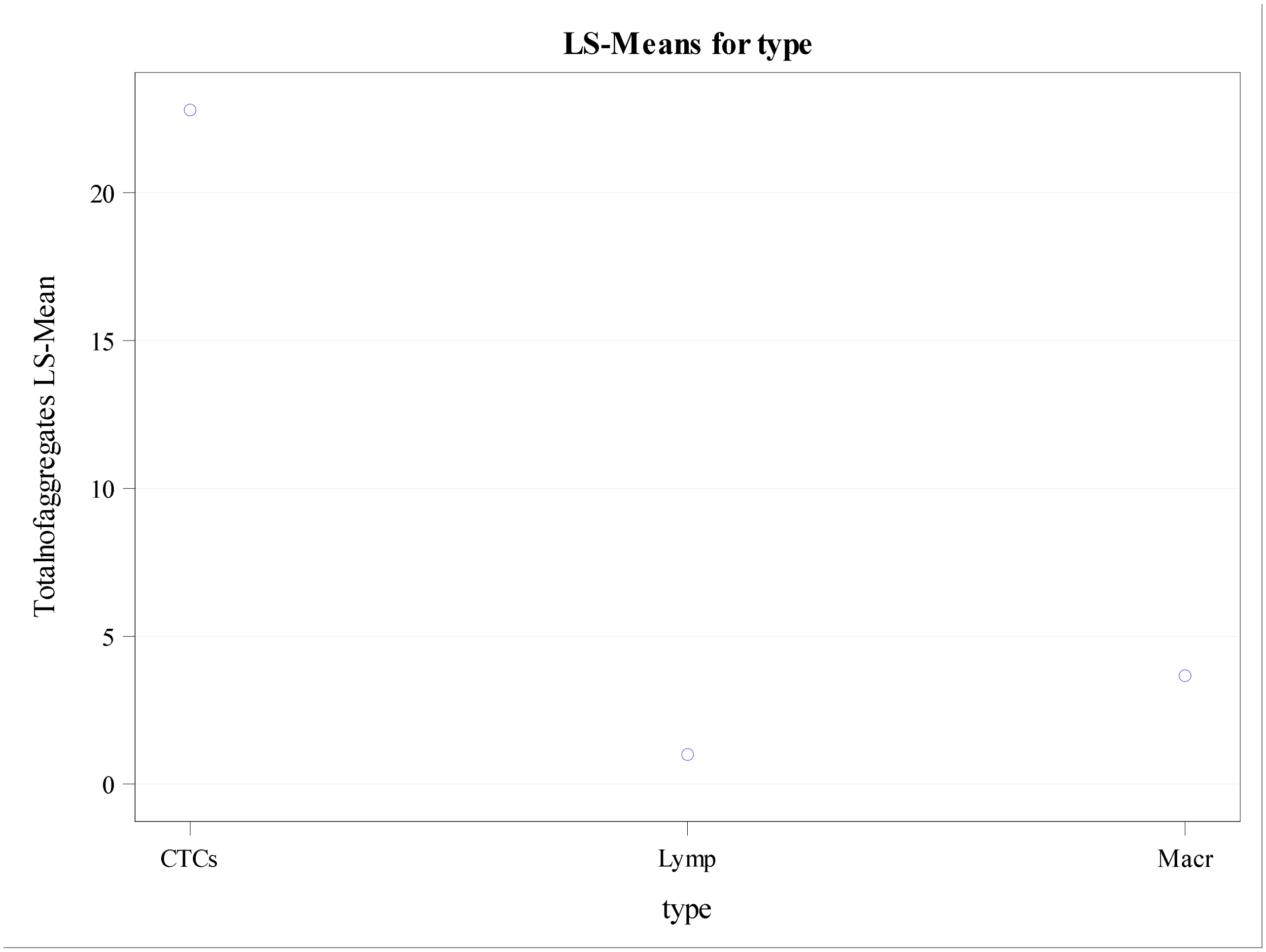

The GLM Procedure
Least Squares Means

pt=16AB0258

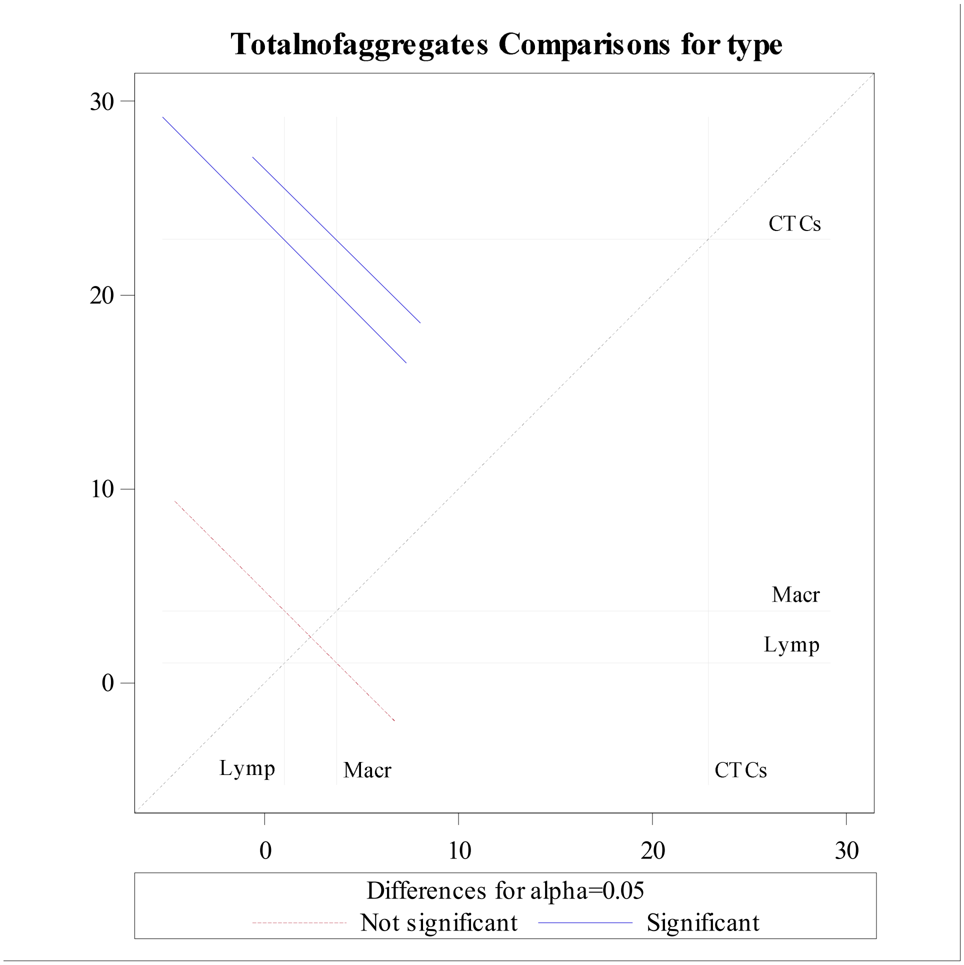

type	acratio LSMEAN	Standard Error	Pr > |t|	LSMEAN Number
**CTCs**	2.42781634	0.47704871	<.0001	1
**Lymp**	4.91853264	0.67464875	<.0001	2
**Macr**	3.65903776	0.30171209	<.0001	3

Least Squares Means for effect type<br>Pr > |t| for H0: LSMean(i)=LSMean(j)<br><br>Dependent Variable: acratio			
**i/j**	**1**	**2**	**3**
**1**		0.0066	0.0407
**2**	0.0066		0.1031
**3**	0.0407	0.1031	

The GLM Procedure
Least Squares Means

pt=16AB0258

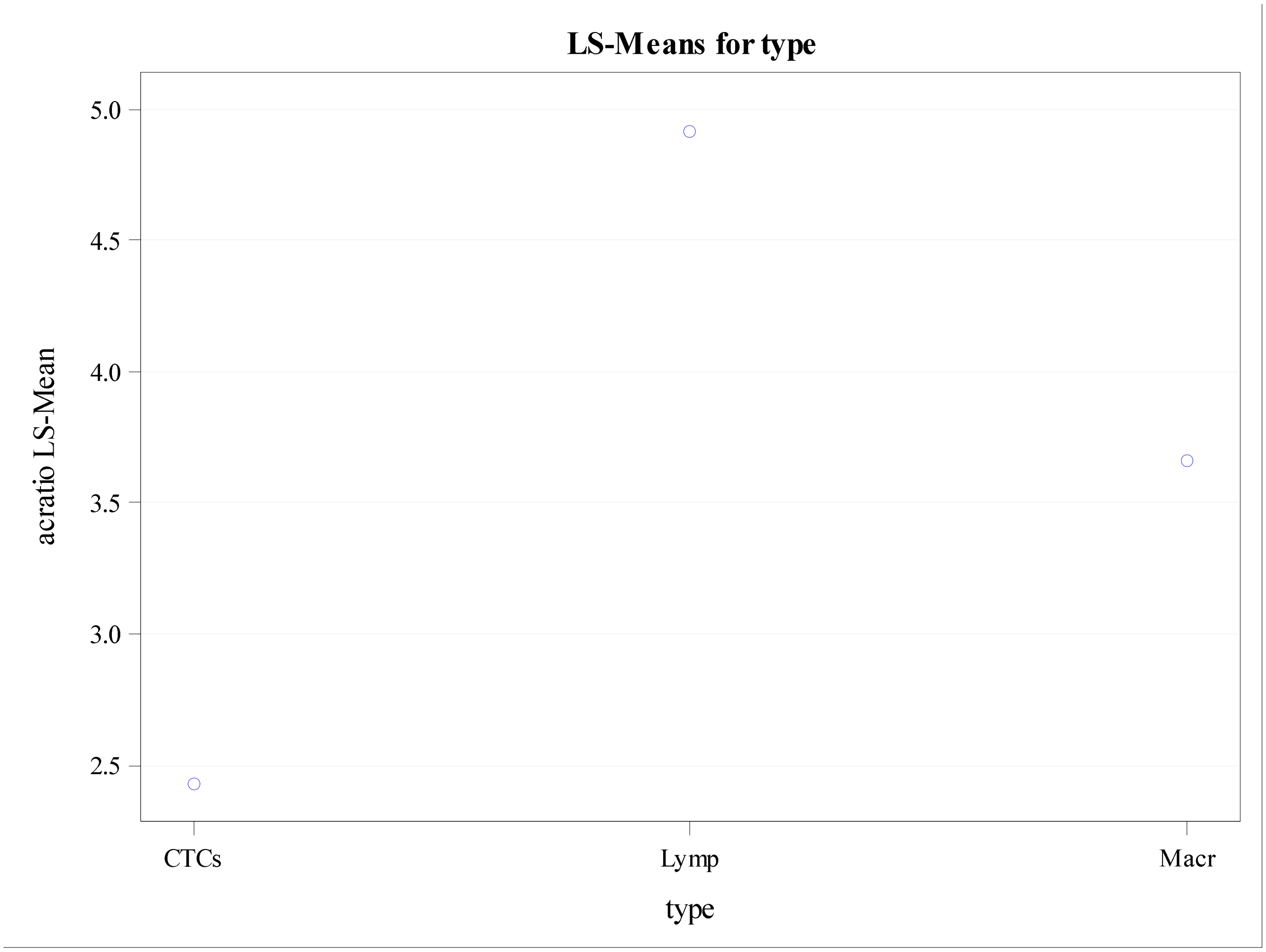

The GLM Procedure
Least Squares Means

pt=16AB0258

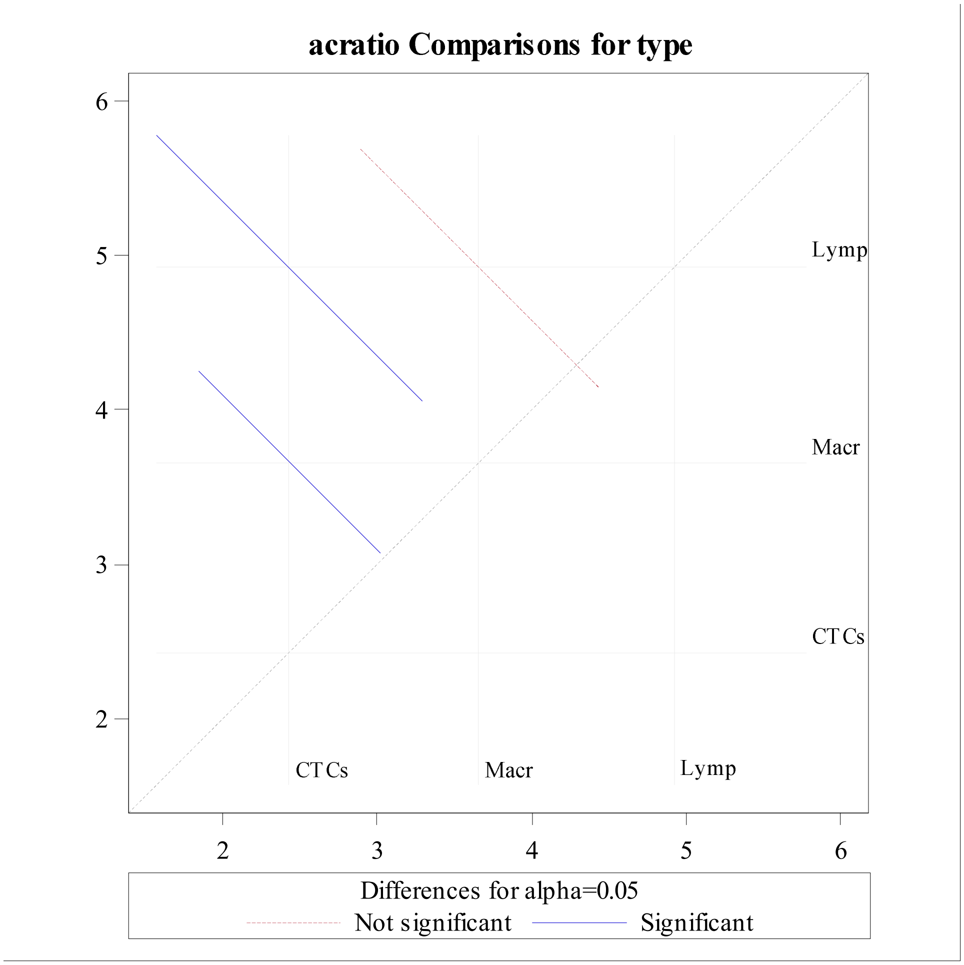

type	AvIntallsignals LSMEAN	Standard Error	Pr > |t|	LSMEAN Number
**CTCs**	10320.8782	1703.5611	<.0001	1
**Lymp**	17038.4698	2409.1993	<.0001	2
**Macr**	13178.8105	1077.4267	<.0001	3

Least Squares Means for effect type<br>Pr > |t| for H0: LSMean(i)=LSMean(j)<br><br>Dependent Variable: AvIntallsignals			
**i/j**	**1**	**2**	**3**
**1**		0.0334	0.1709
**2**	0.0334		0.1584
**3**	0.1709	0.1584	

The GLM Procedure
Least Squares Means

pt=16AB0258

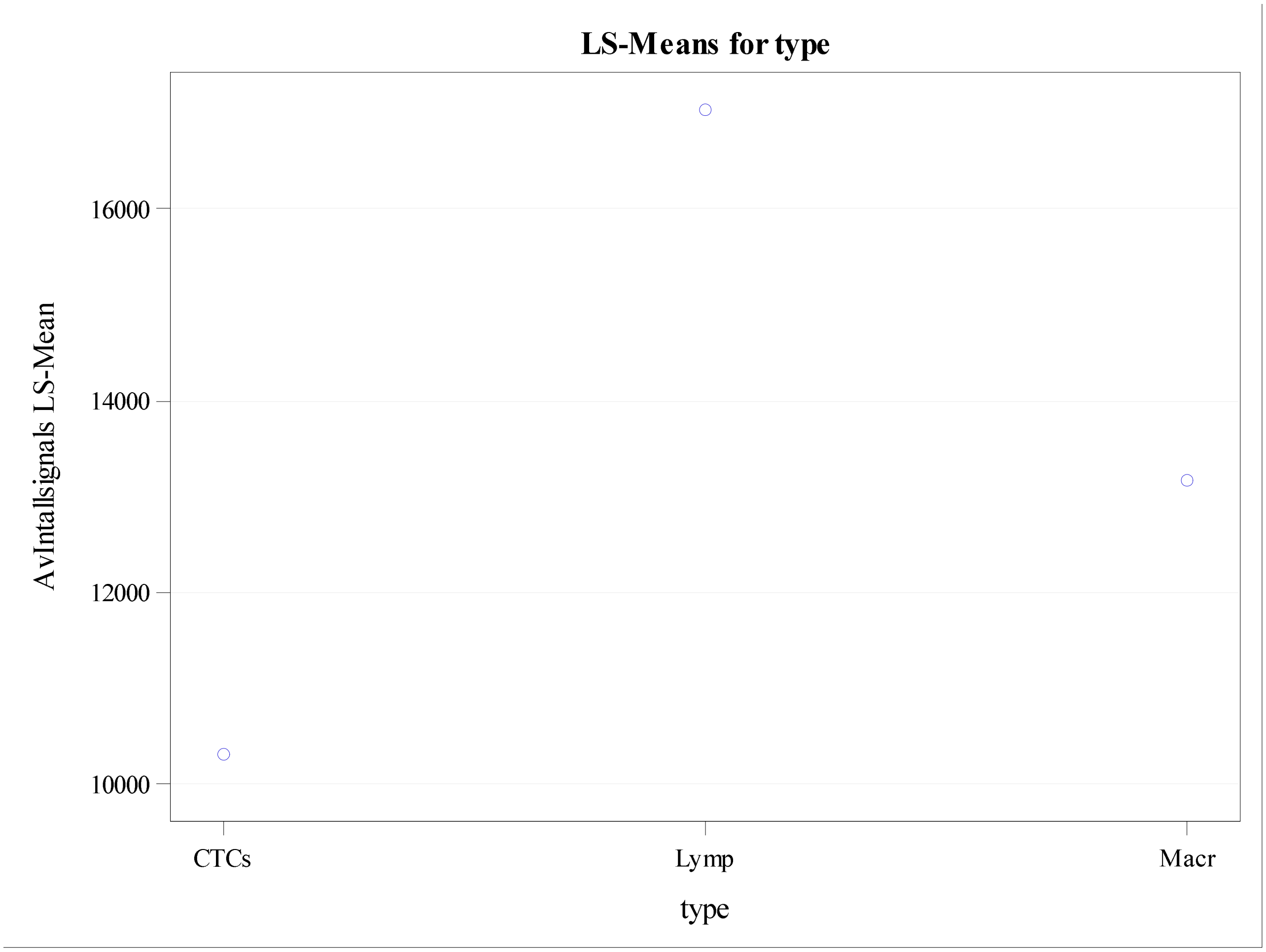

The GLM Procedure
Least Squares Means

pt=16AB0258

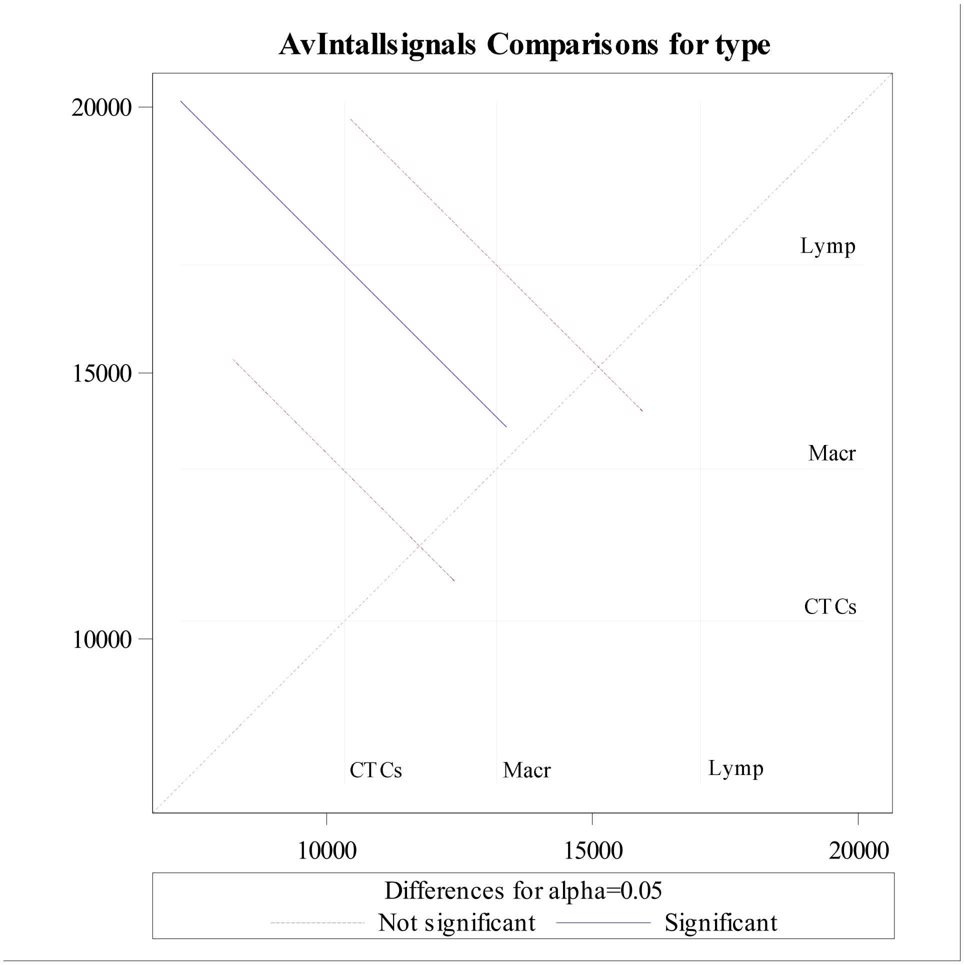

type	Totalintensity LSMEAN	Standard Error	Pr > |t|	LSMEAN Number
**CTCs**	1572904.33	166497.45	<.0001	1
**Lymp**	403921.67	235462.95	0.1010	2
**Macr**	471037.60	105302.23	0.0002	3

Least Squares Means for effect type<br>Pr > |t| for H0: LSMean(i)=LSMean(j)<br><br>Dependent Variable: Totalintensity			
**i/j**	**1**	**2**	**3**
**1**		0.0006	<.0001
**2**	0.0006		0.7972
**3**	<.0001	0.7972	

The GLM Procedure
Least Squares Means

pt=16AB0258

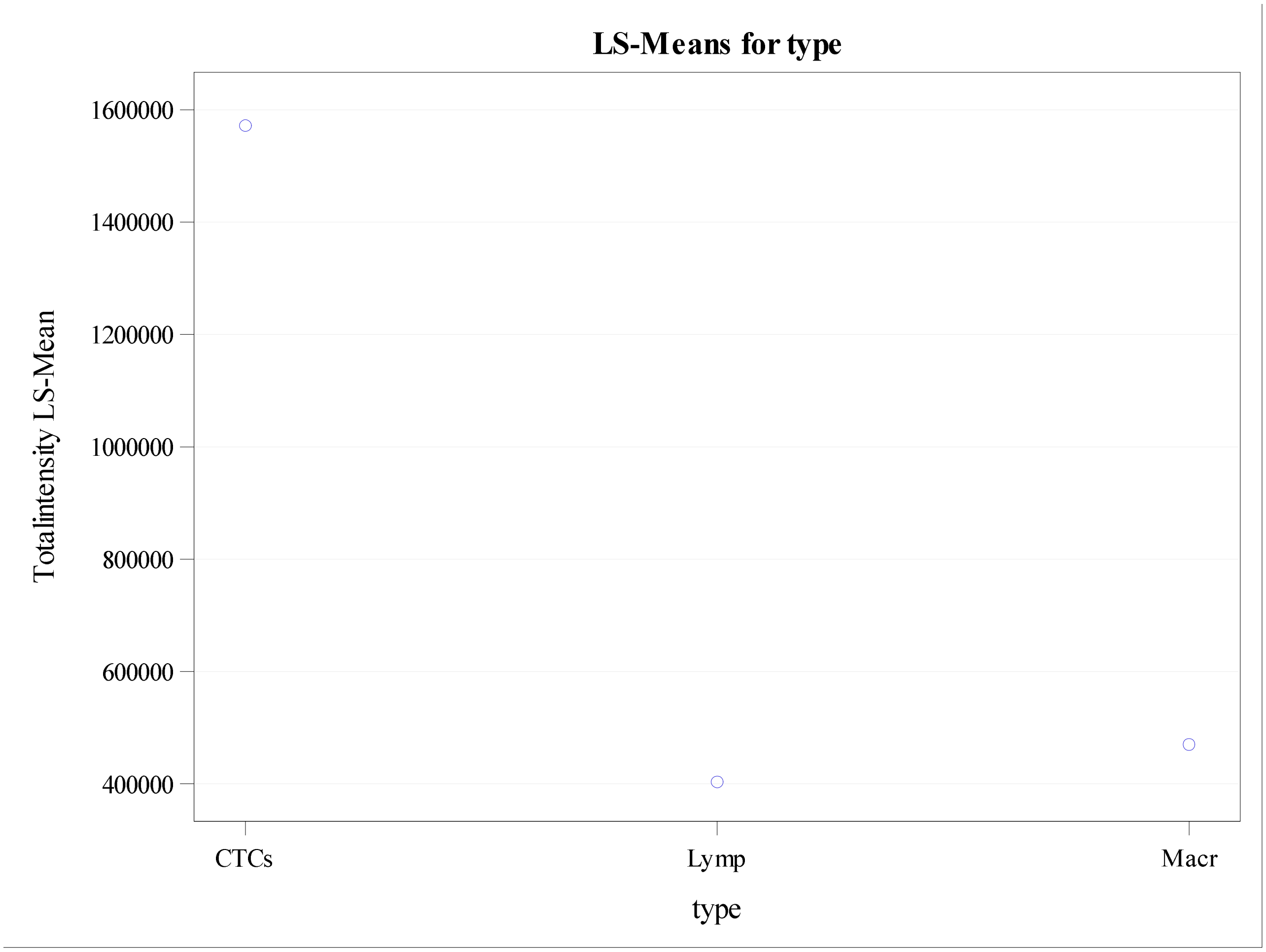

The GLM Procedure
Least Squares Means

pt=16AB0258

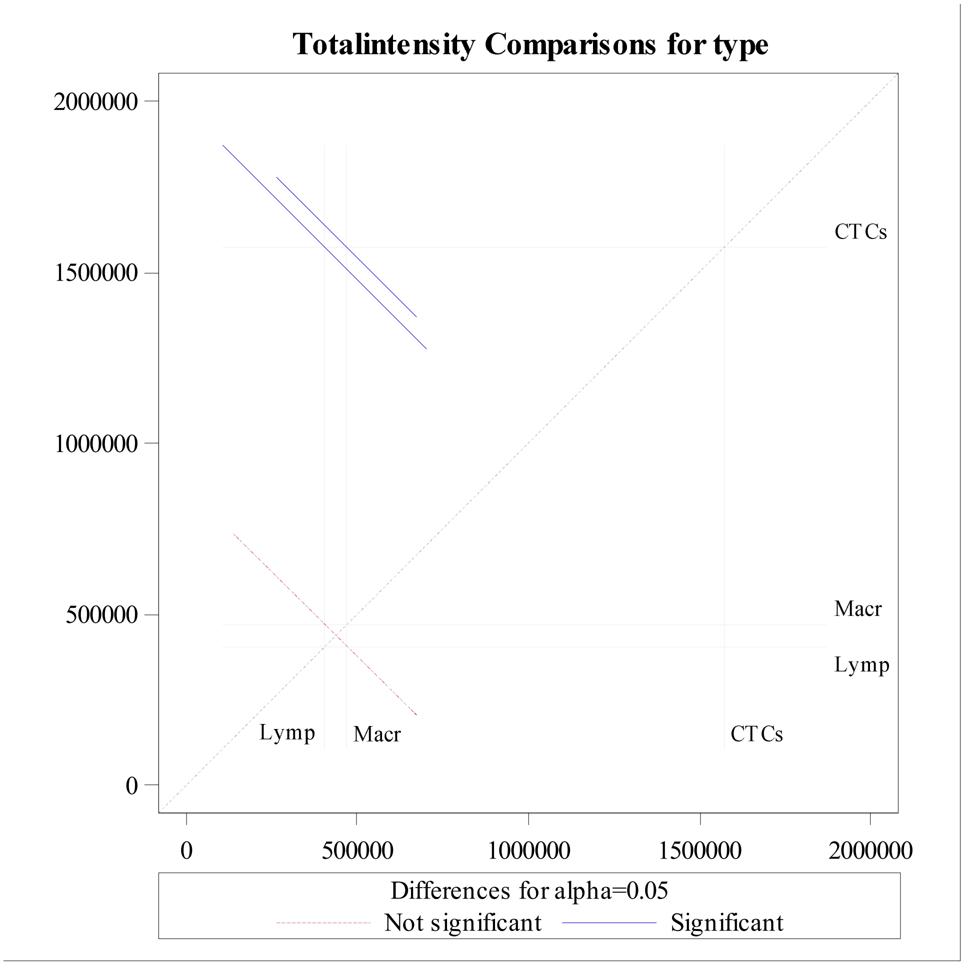

type	Nuclearvolume LSMEAN	Standard Error	Pr > |t|	LSMEAN Number
**CTCs**	1041021.83	100040.25	<.0001	1
**Lymp**	348797.33	141478.28	0.0224	2
**Macr**	500413.67	63271.01	<.0001	3

Least Squares Means for effect type<br>Pr > |t| for H0: LSMean(i)=LSMean(j)<br><br>Dependent Variable: Nuclearvolume			
**i/j**	**1**	**2**	**3**
**1**		0.0007	0.0002
**2**	0.0007		0.3391
**3**	0.0002	0.3391	

The GLM Procedure
Least Squares Means

pt=16AB0258

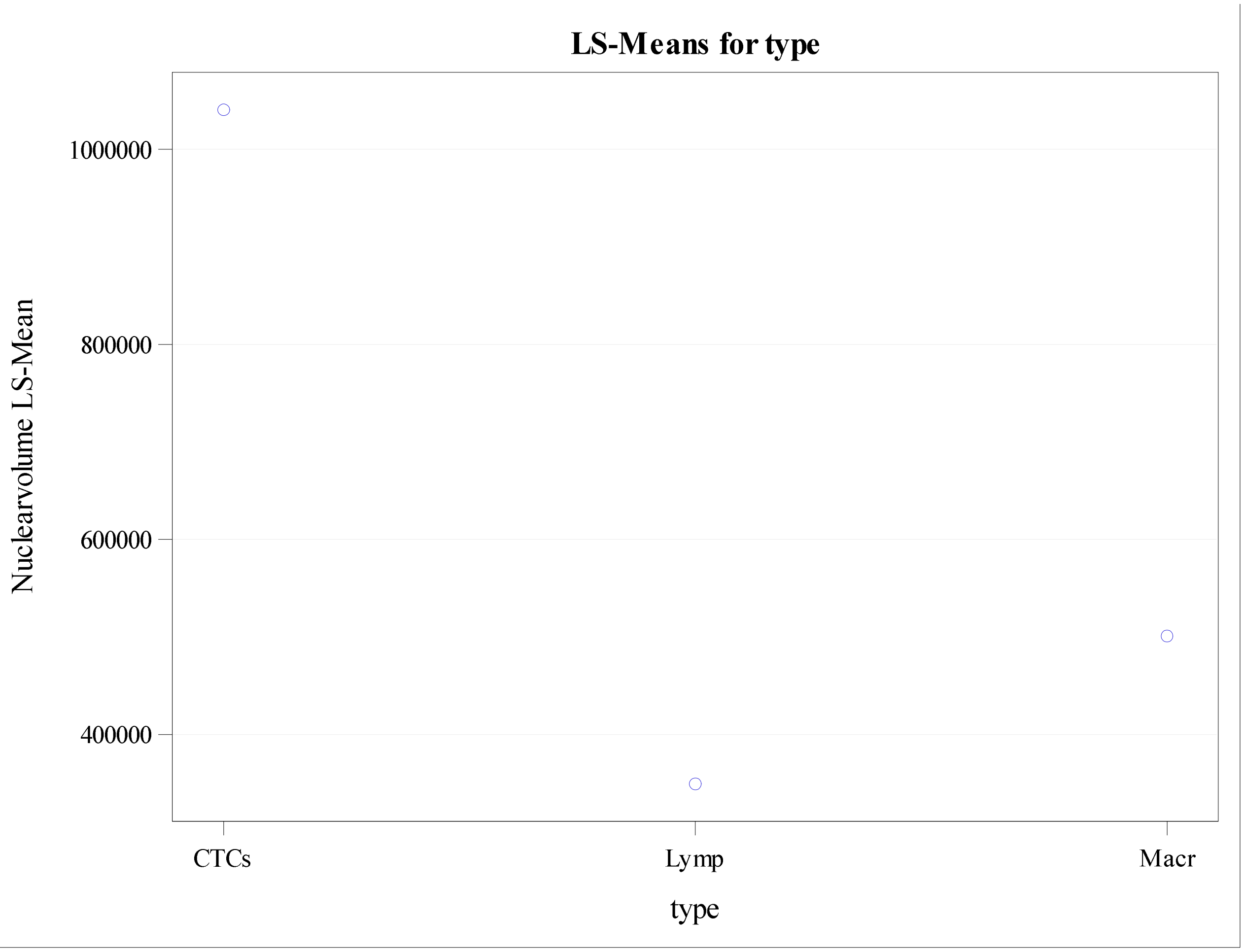

The GLM Procedure
Least Squares Means

pt=16AB0258

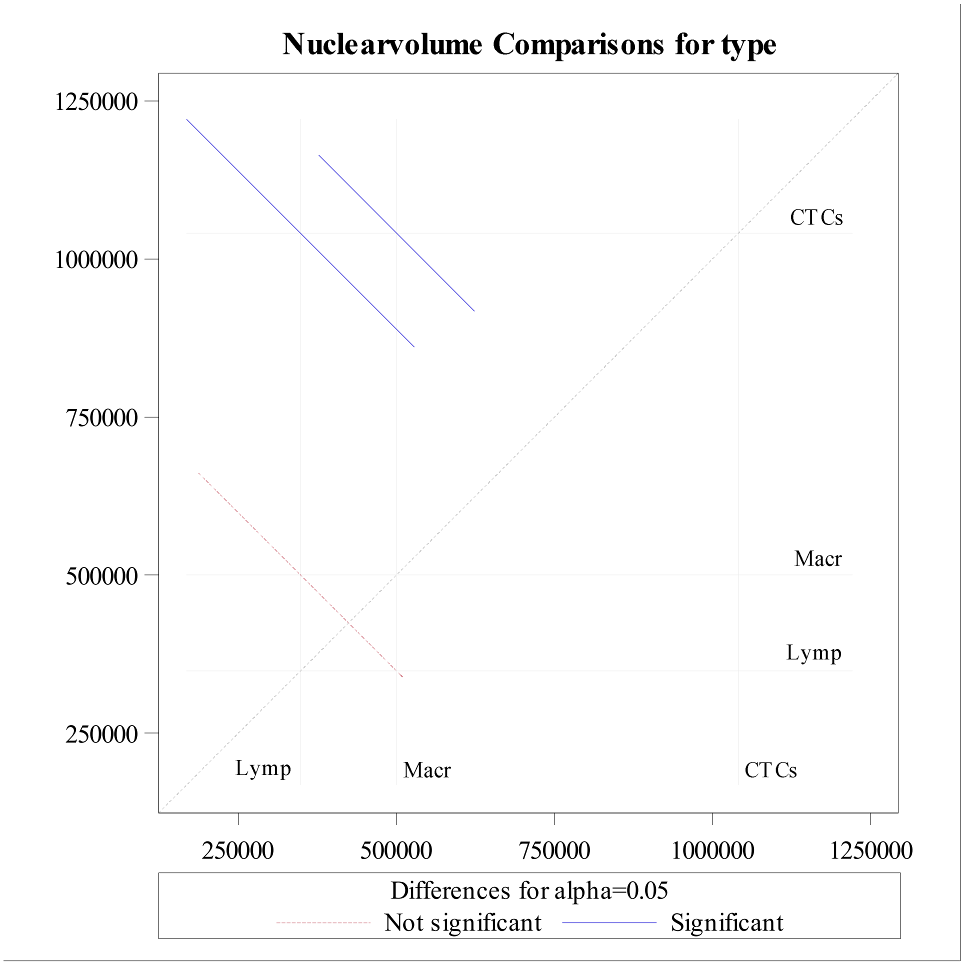

type	telomereperkvol LSMEAN	Standard Error	Pr > |t|	LSMEAN Number
**CTCs**	0.14847493	0.00995922	<.0001	1
**Lymp**	0.07544235	0.01408446	<.0001	2
**Macr**	0.07362463	0.00629876	<.0001	3

**Least Squares Means for effect type Pr > |t| for H0: LSMean(i)=LSMean(j) Dependent Variable: telomereperkvol**			
**i/j**	**1**	**2**	**3**
**1**		0.0004	<.0001
**2**	0.0004		0.9073
**3**	<.0001	0.9073	

The GLM Procedure
Least Squares Means

pt=16AB0258

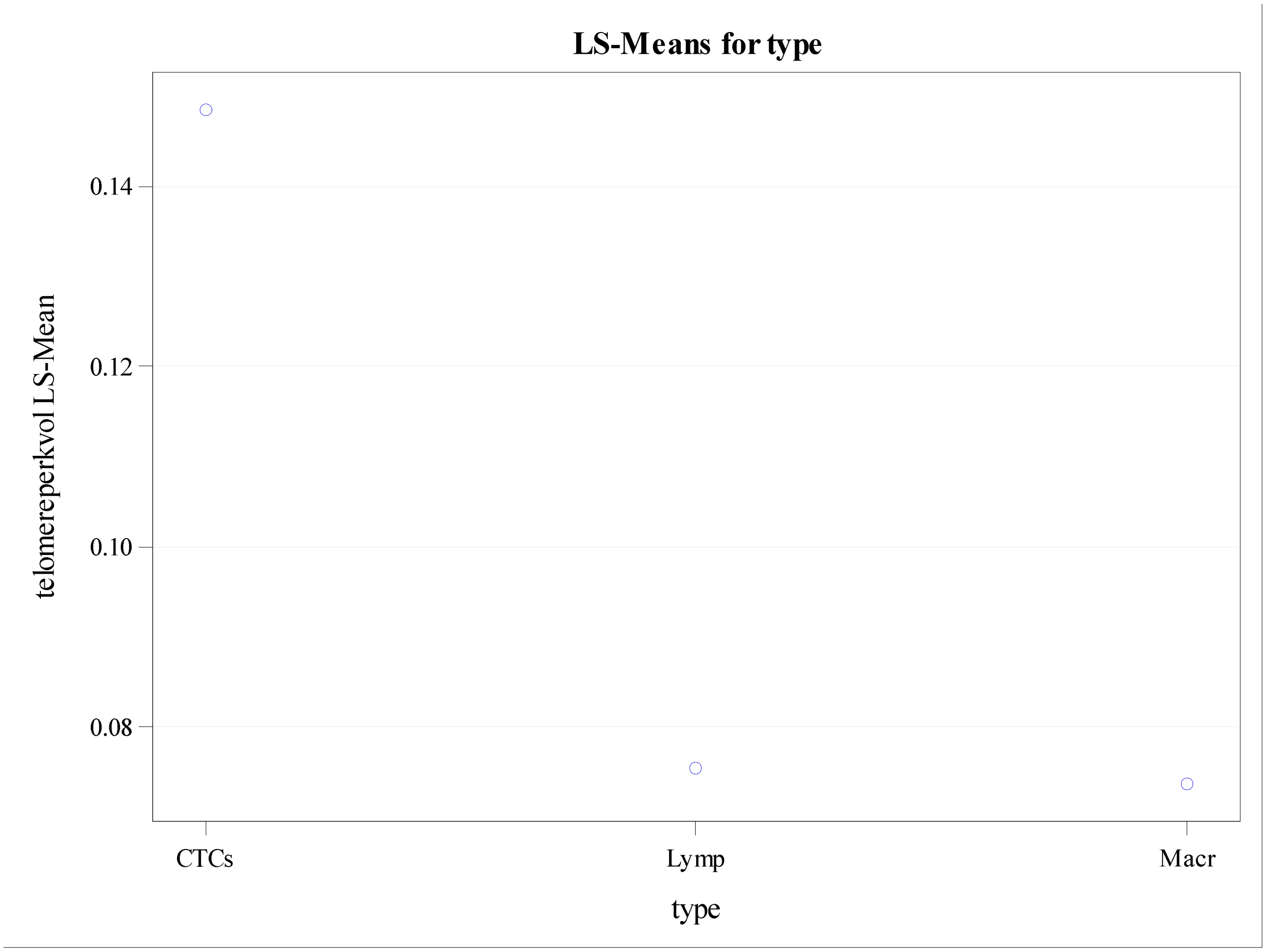

The GLM Procedure
Least Squares Means

pt=16AB0258

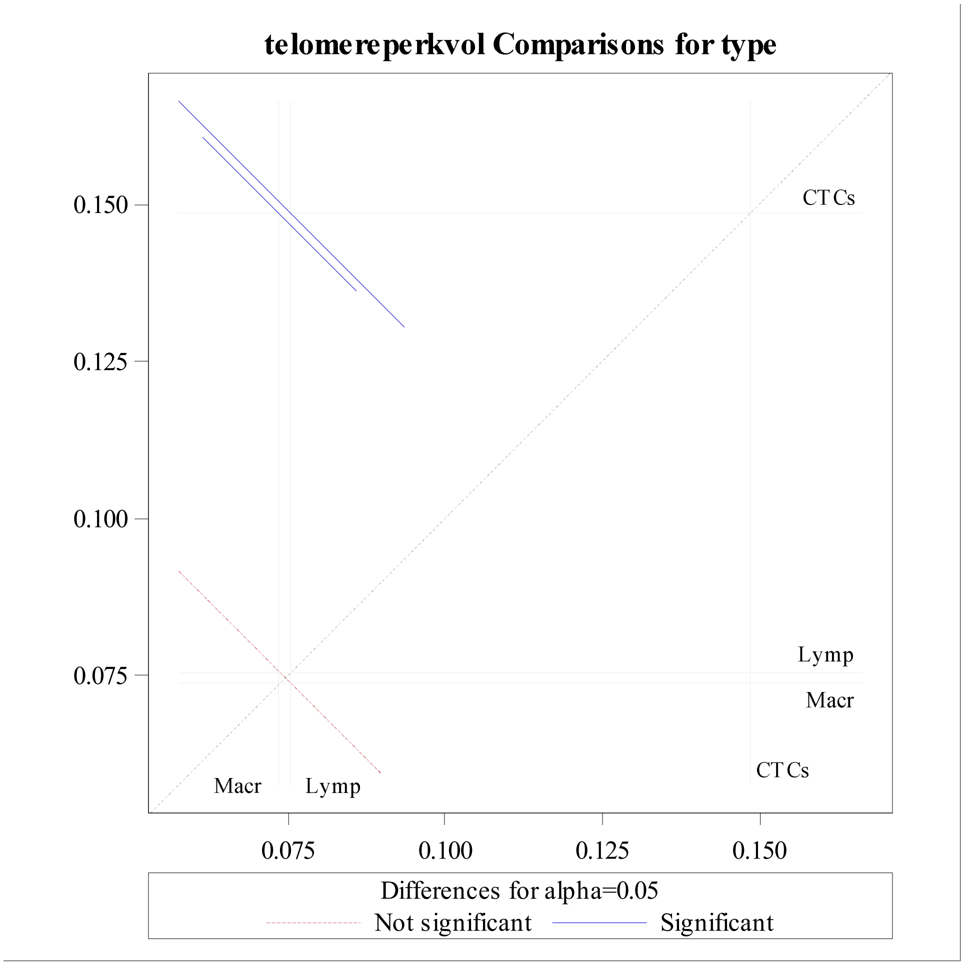

Note: To ensure overall protection level, only probabilities associated with pre-planned comparisons should be used.

The GLM Procedure

pt=16AB0304

Class Level Information		
**Class**	**Levels**	**Values**
**type**	3	CTCs Lymp Macr

**Number of Observations Read**	66
**Number of Observations Used**	66

The GLM Procedure

Dependent Variable: Totalnofsignals
Totalnofsignals

pt=16AB0304

Source	DF	Sum of Squares	Mean Square	F Value	Pr > F
**Model**	2	75334.7212	37667.3606	24.67	<.0001
**Error**	63	96186.8091	1526.7747		
**Corrected Total**	65	171521.5303			

R-Square	Coeff Var	Root MSE	Totalnofsignals Mean
0.439214	53.80517	39.07397	72.62121

Source	DF	Type III SS	Mean Square	F Value	Pr > F
**type**	2	75334.72125	37667.36062	24.67	<.0001

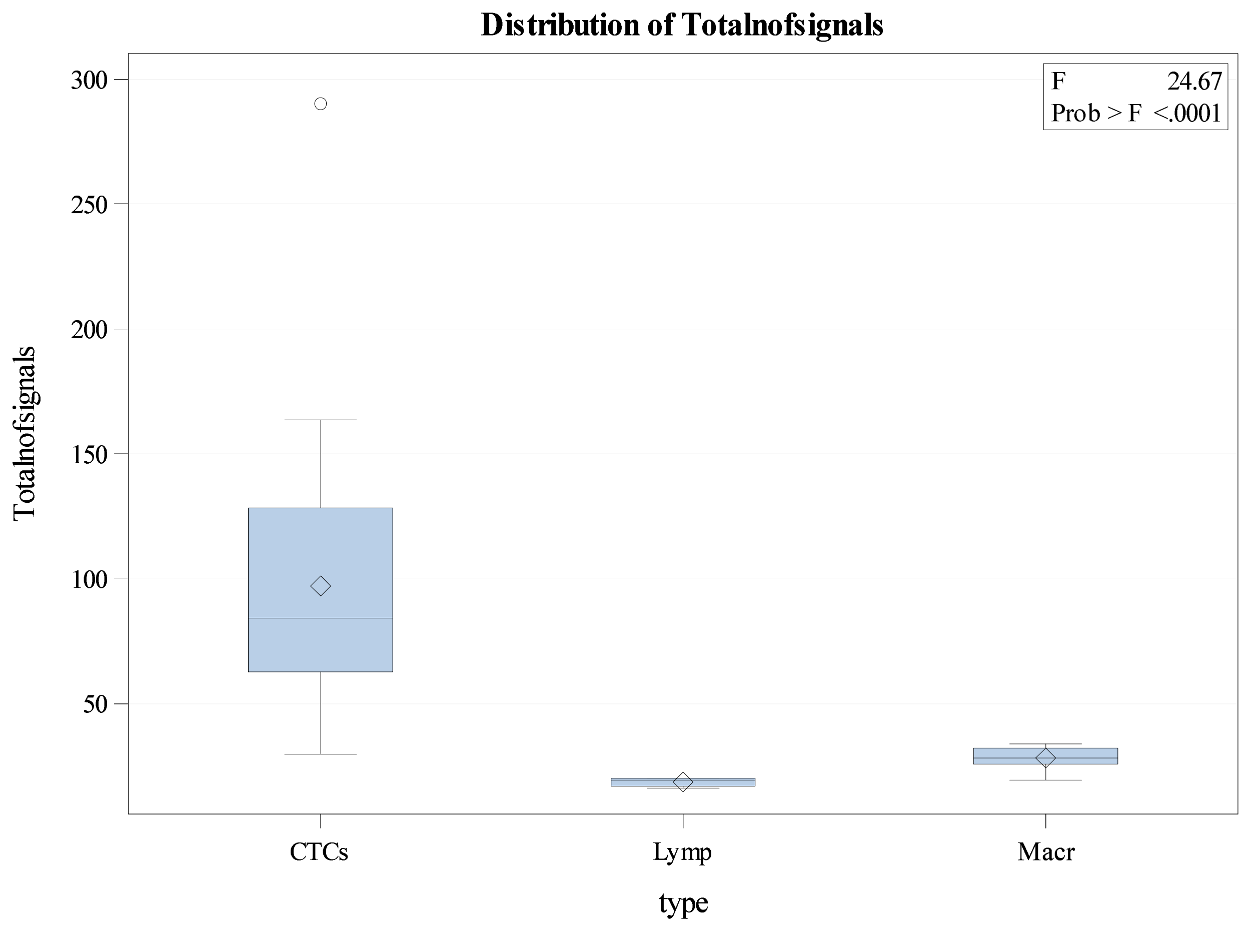

The GLM Procedure

Dependent Variable: Totalnofaggregates Totalnofaggregates

pt=16AB0304

Source	DF	Sum of Squares	Mean Square	F Value	Pr > F
Model	2	1218.768091	609.384045	12.04	<.0001
Error	63	3189.853121	50.632589		
Corrected Total	65	4408.621212			

R-Square	Coeff Var	Root MSE	Totalnofaggregates Mean
0.276451	88.11134	7.115658	8.075758

Source	DF	Type III SS	Mean Square	F Value	Pr > F
type	2	1218.768091	609.384045	12.04	<.0001

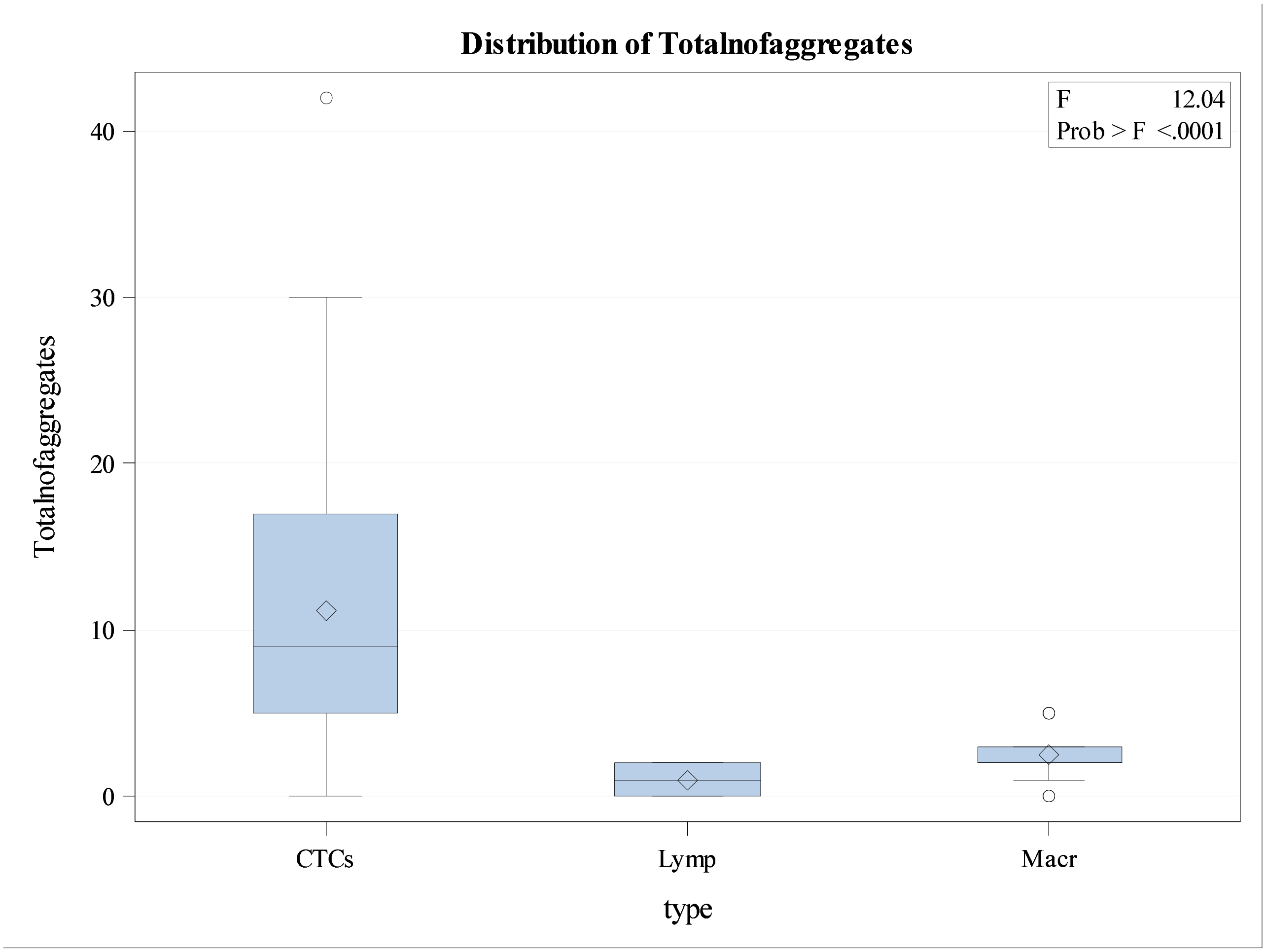

The GLM Procedure

Dependent Variable: acratio
acratio

pt=16AB0304

Source	DF	Sum of Squares	Mean Square	F Value	Pr > F
**Model**	2	119.0228977	59.5114488	18.31	<.0001
**Error**	63	204.7902579	3.2506390		
**Corrected Total**	65	323.8131556			

R-Square	Coeff Var	Root MSE	acratio Mean
0.367567	47.81362	1.802953	3.770793

Source	DF	Type III SS	Mean Square	F Value	Pr > F
**type**	2	119.0228977	59.5114488	18.31	<.0001

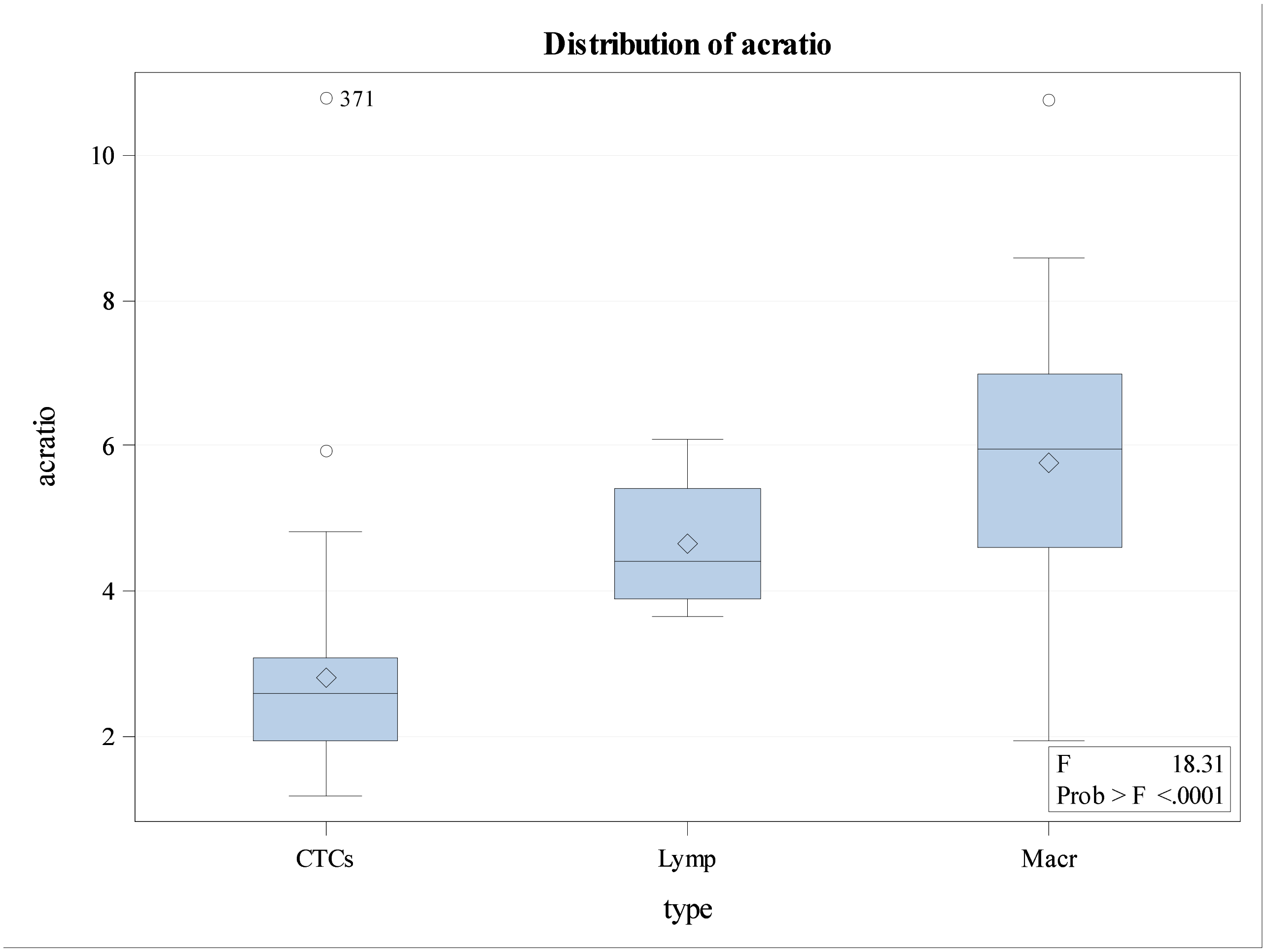

The GLM Procedure

Dependent Variable: AvIntallsignals
AvIntallsignals

pt=16AB0304

Source	DF	Sum of Squares	Mean Square	F Value	Pr > F
**Model**	2	53997655.4	26998827.7	9.43	0.0003
**Error**	63	180444107.7	2864192.2		
**Corrected Total**	65	234441763.1			

R-Square	Coeff Var	Root MSE	AvIntallsignals Mean
0.230324	18.19323	1692.392	9302.322

Source	DF	Type III SS	Mean Square	F Value	Pr > F
**type**	2	53997655.38	26998827.69	9.43	0.0003

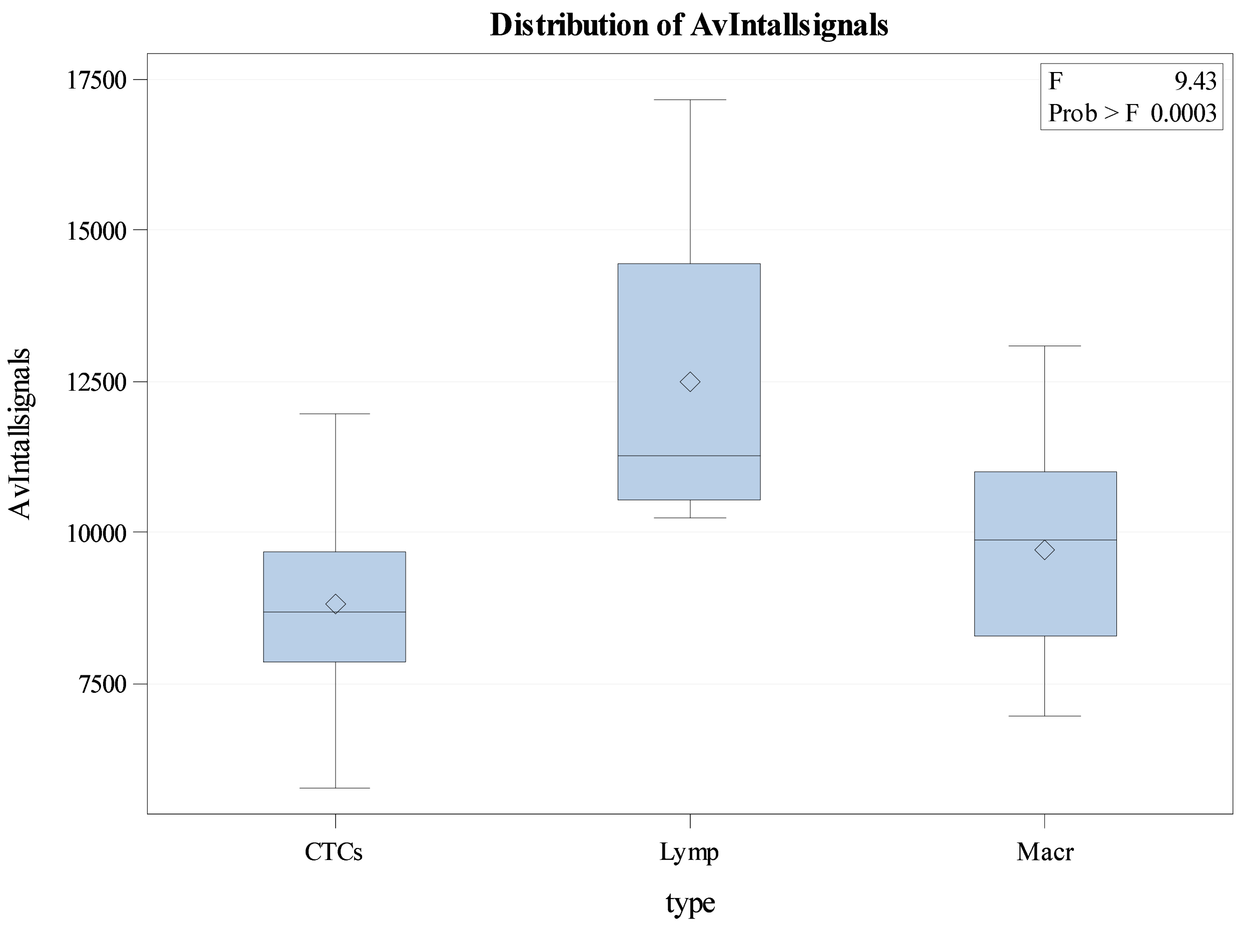

The GLM Procedure

Dependent Variable: Totalintensity Totalintensity

pt=16AB0304

Source	DF	Sum of Squares	Mean Square	F Value	Pr > F
**Model**	2	4.5664197E12	2.2832099E12	33.68	<.0001
**Error**	63	4.2708147E12	67790709622		
**Corrected Total**	65	8.8372344E12			

R-Square	Coeff Var	Root MSE	Totalintensity Mean
0.516725	41.57956	260366.5	626188.7

Source	DF	Type III SS	Mean Square	F Value	Pr > F
**type**	2	4.5664197E12	2.2832099E12	33.68	<.0001

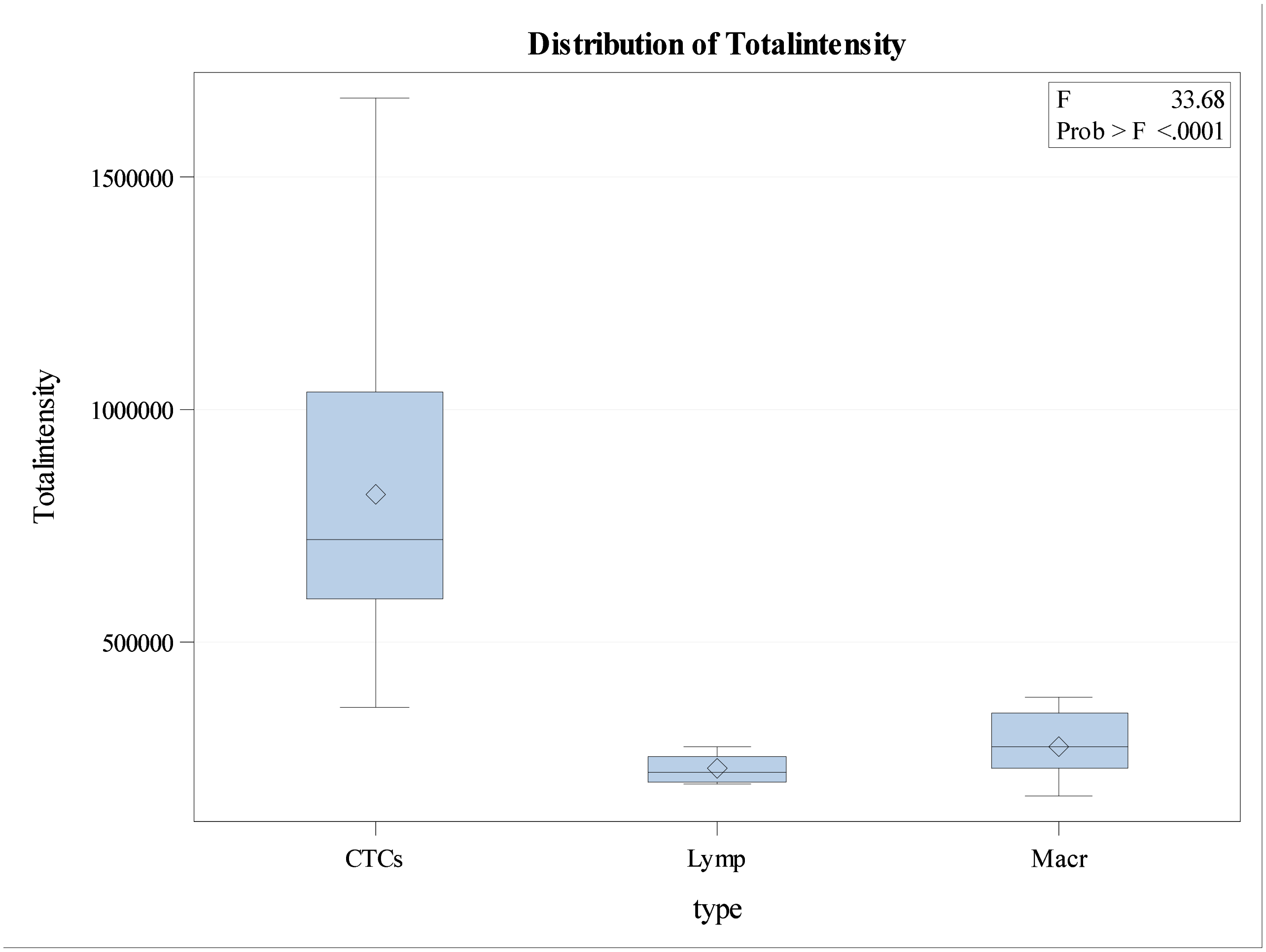

The GLM Procedure

Dependent Variable: Nuclearvolume Nuclearvolume

pt=16AB0304

Source	DF	Sum of Squares	Mean Square	F Value	Pr > F
**Model**	2	3.3752698E12	1.6876349E12	9.56	0.0002
**Error**	63	1.112016E13	176510482301		
**Corrected Total**	65	1.449543E13			

R-Square	Coeff Var	Root MSE	Nuclearvolume Mean
0.232851	54.39994	420131.5	772301.4

Source	DF	Type III SS	Mean Square	F Value	Pr > F
**type**	2	3.3752698E12	1.6876349E12	9.56	0.0002

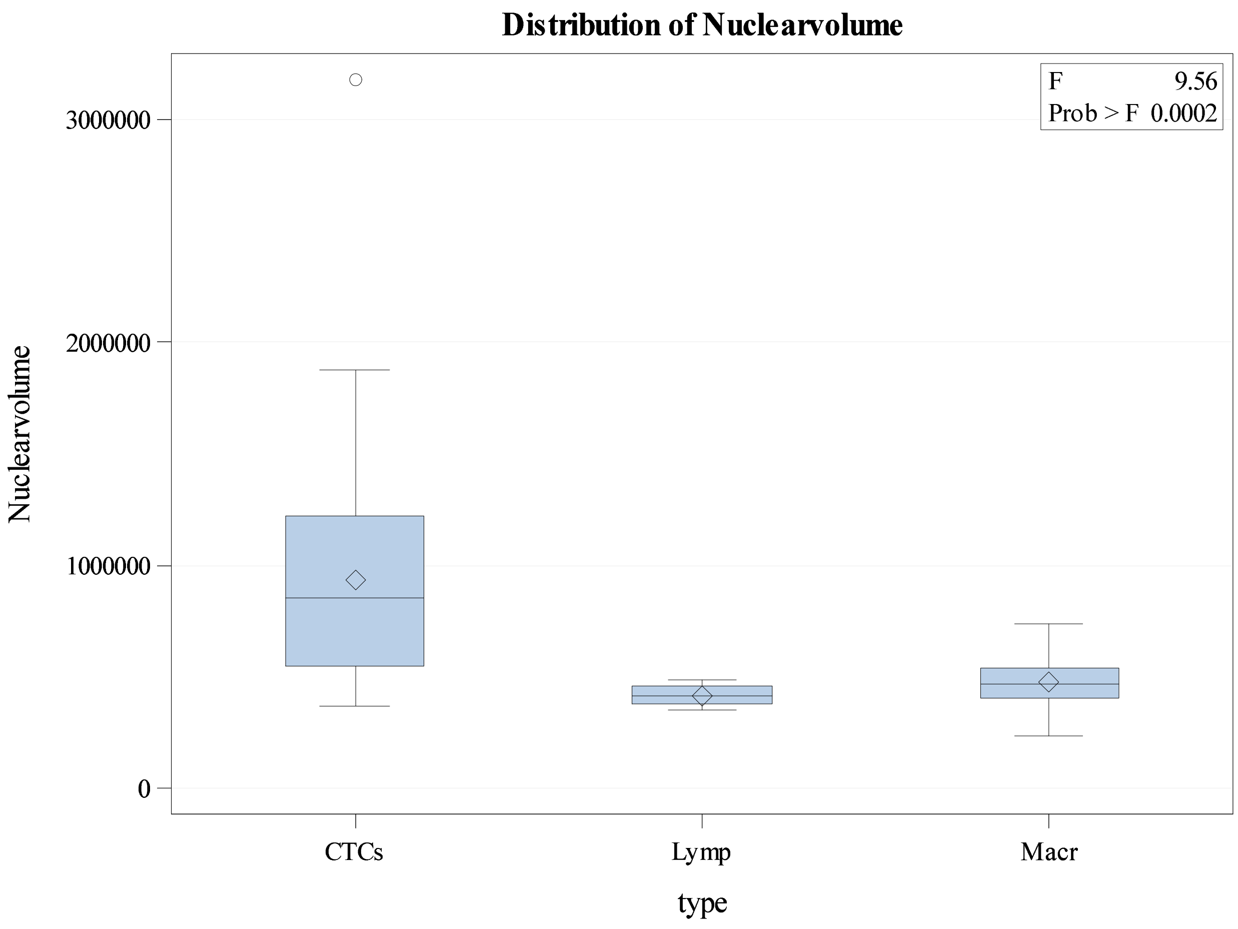

The GLM Procedure

Dependent Variable: telomereperkvol

pt=16AB0304

Source	DF	Sum of Squares	Mean Square	F Value	Pr > F
**Model**	2	0.03687050	0.01843525	38.34	<.0001
**Error**	63	0.03029535	0.00048088		
**Corrected Total**	65	0.06716585			

R-Square	Coeff Var	Root MSE	telomereperkvol Mean
0.548947	24.05241	0.021929	0.091171

Source	DF	Type III SS	Mean Square	F Value	Pr > F
**type**	2	0.03687050	0.01843525	38.34	<.0001

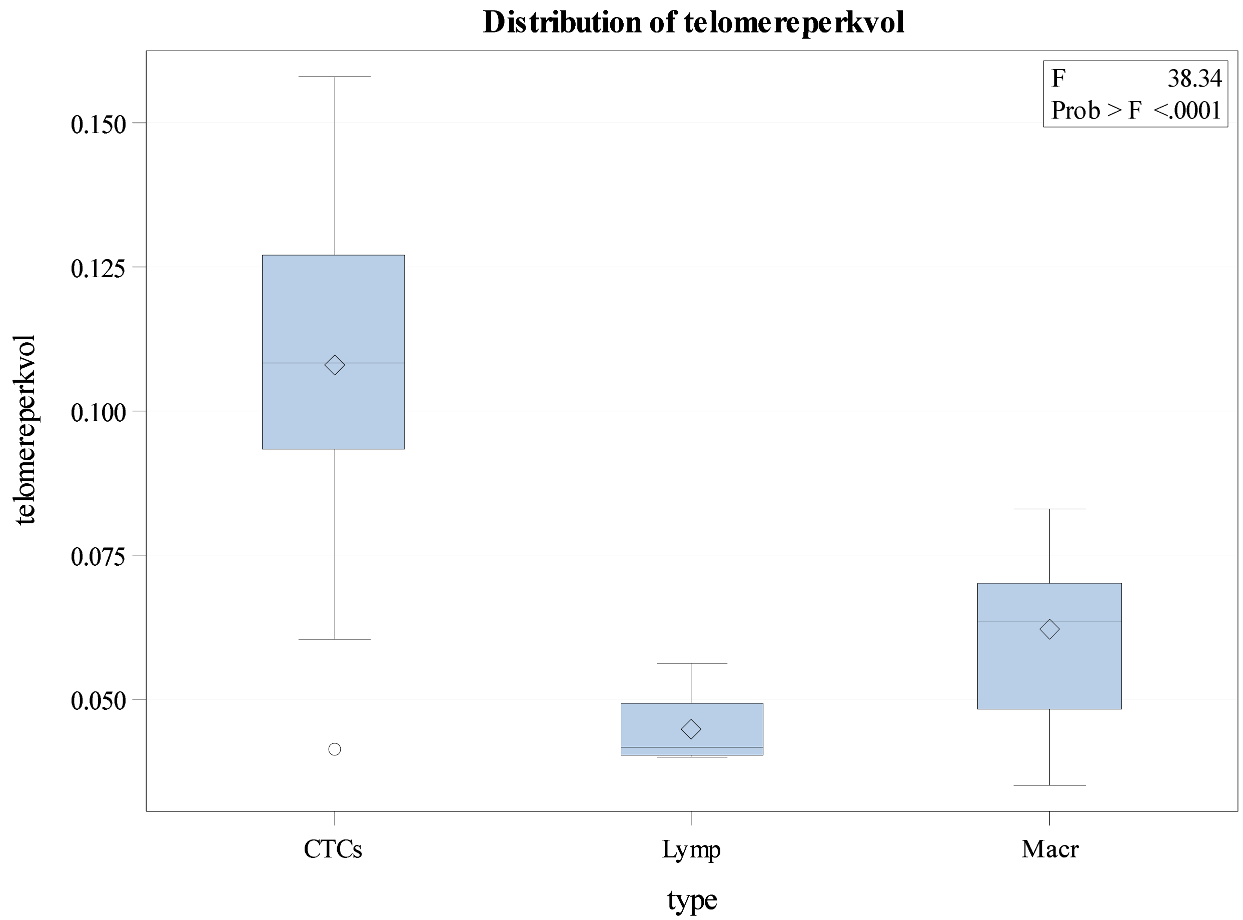

The GLM Procedure
Least Squares Means

pt=16AB0304

type	Totalnofsignals LSMEAN	Standard Error	Pr > |t|	LSMEAN Number
**CTCs**	97.2790698	5.9587238	<.0001	1
**Lymp**	18.5000000	19.5369825	0.3473	2
**Macr**	28.2105263	8.9641824	0.0025	3

Least Squares Means for effect type<br>Pr > |t| for H0: LSMean(i)=LSMean(j)<br><br>Dependent Variable: Totalnofsignals			
**i/j**	**1**	**2**	**3**
**1**		0.0003	<.0001
**2**	0.0003		0.6530
**3**	<.0001	0.6530	

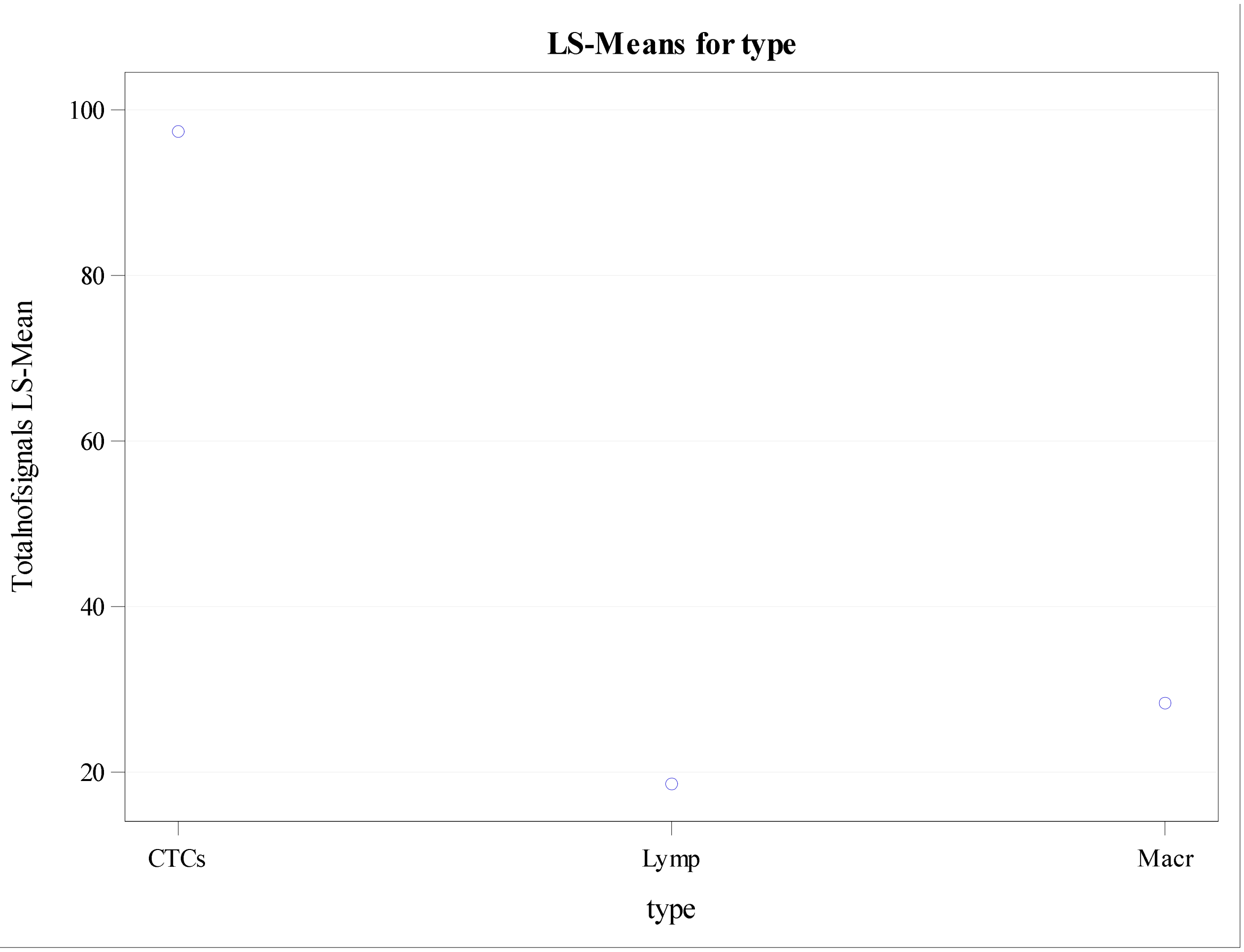

The GLM Procedure
Least Squares Means

pt=16AB0304

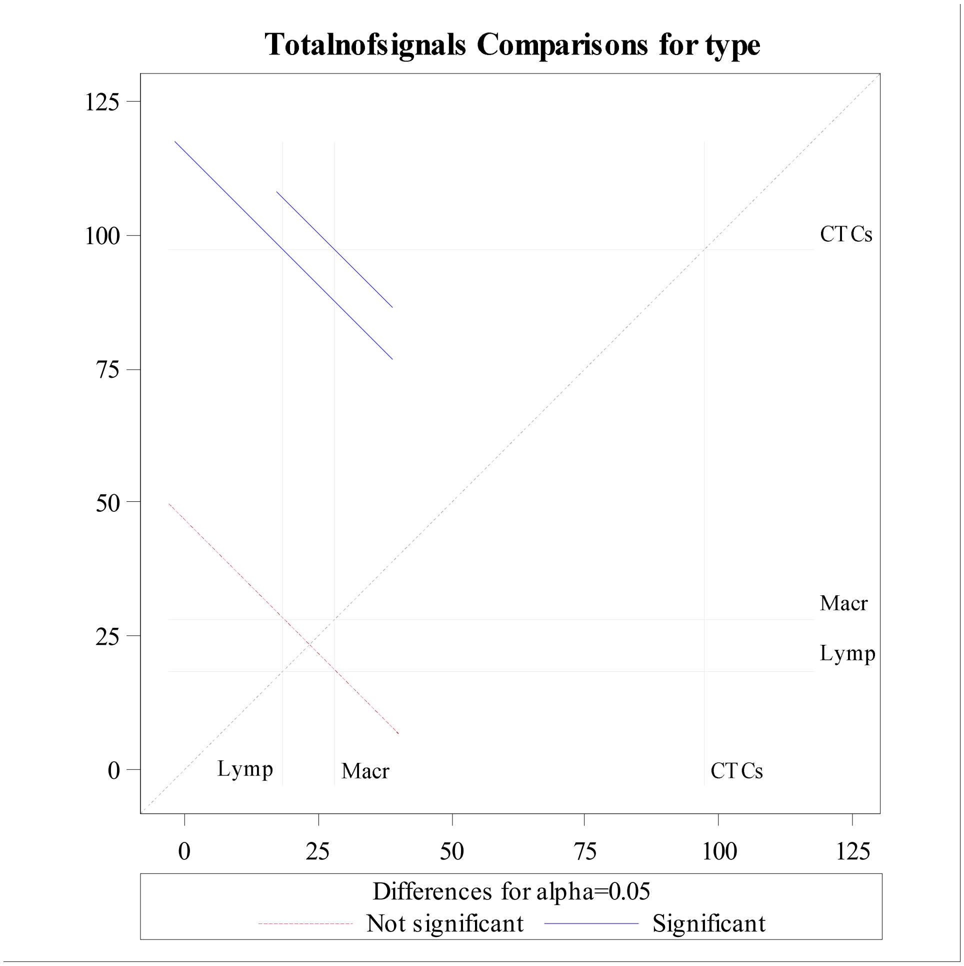

type	Totalnofaggregates LSMEAN	Standard Error	Pr > |t|	LSMEAN Number
**CTCs**	11.2093023	1.0851277	<.0001	1
**Lymp**	1.0000000	3.5578290	0.7796	2
**Macr**	2.4736842	1.6324439	0.1347	3

The GLM Procedure
Least Squares Means

pt=16AB0304

Least Squares Means for effect type<br>Pr > |t| for H0: LSMean(i)=LSMean(j)<br><br>Dependent Variable: Totalnofaggregates			
**i/j**	**1**	**2**	**3**
**1**		0.0079	<.0001
**2**	0.0079		0.7078
**3**	<.0001	0.7078	

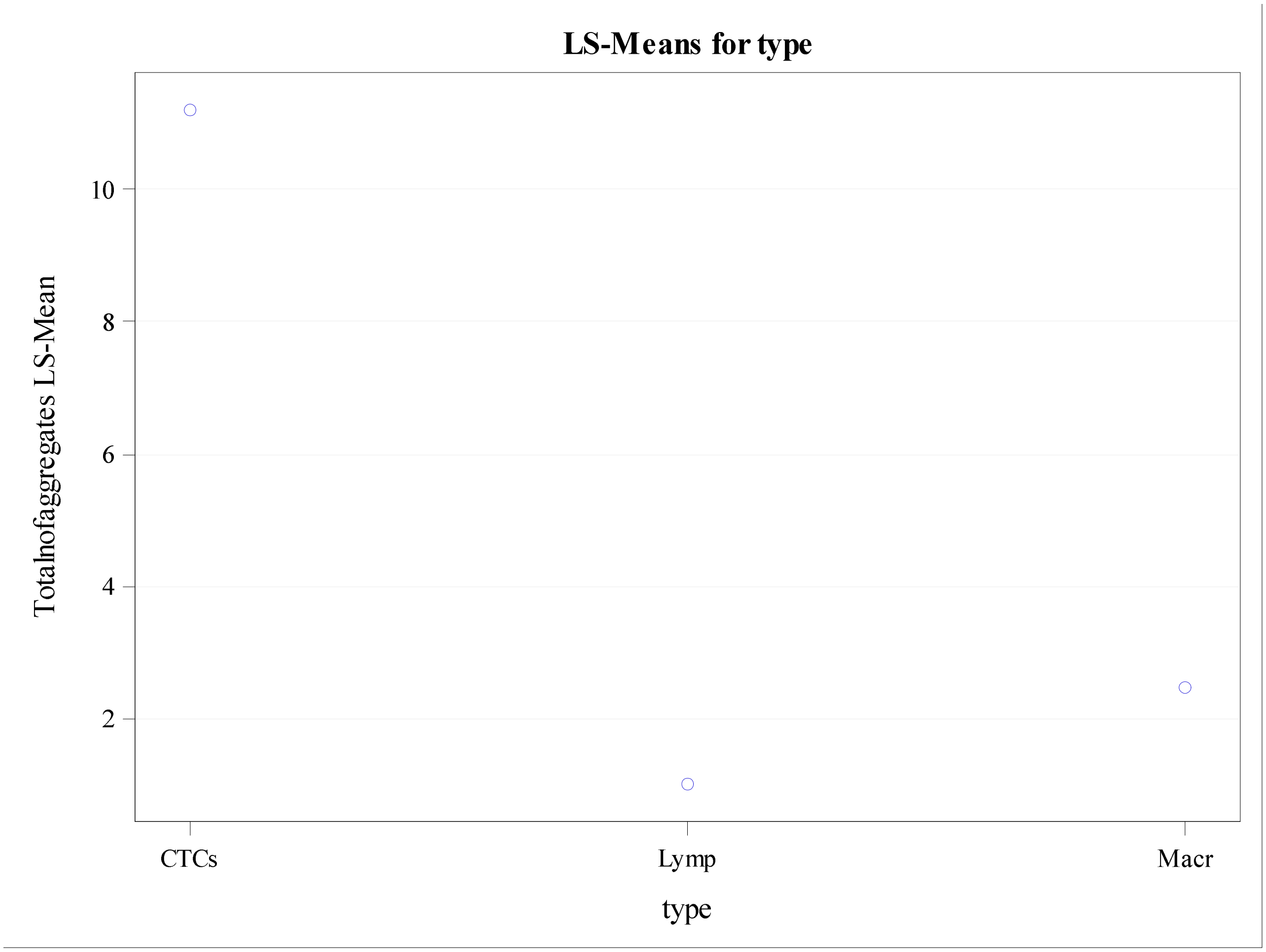

The GLM Procedure
Least Squares Means

pt=16AB0304

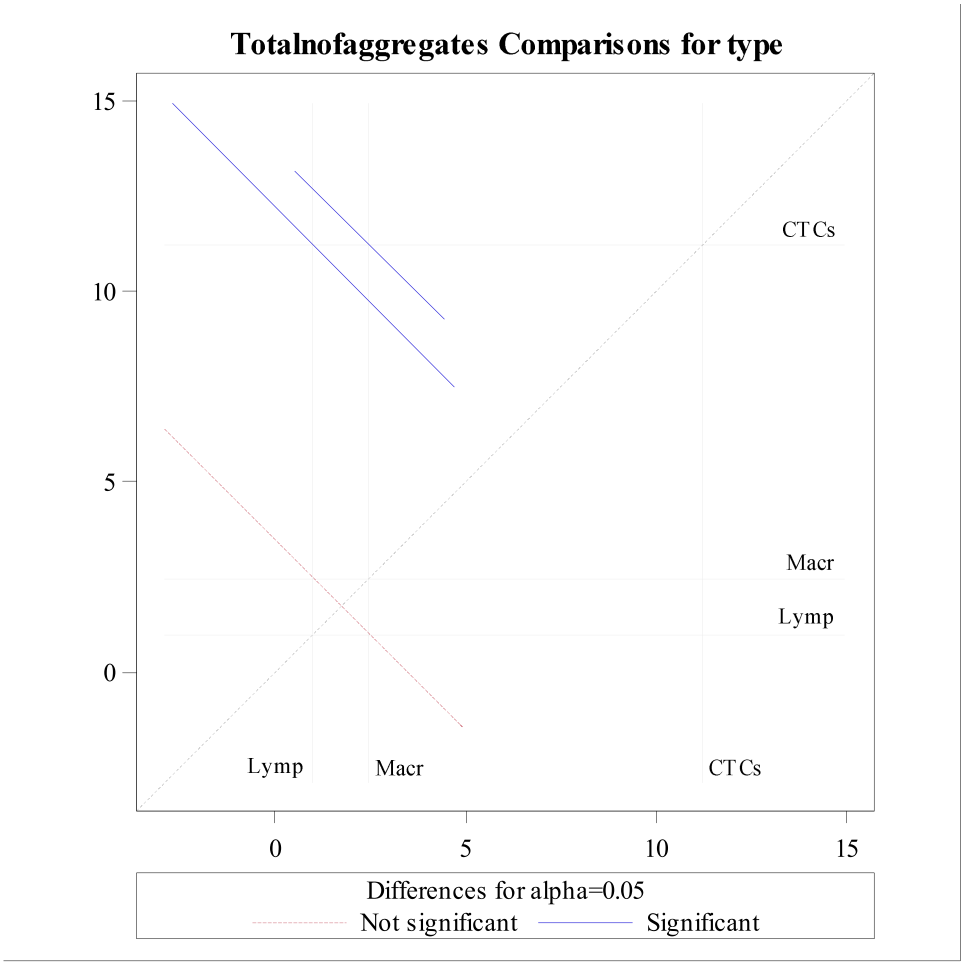

type	acratio LSMEAN	Standard Error	Pr > |t|	LSMEAN Number
**CTCs**	2.80619455	0.27494773	<.0001	1
**Lymp**	4.64135848	0.90147643	<.0001	2
**Macr**	5.77055619	0.41362575	<.0001	3

Least Squares Means for effect type<br>Pr > |t| for H0: LSMean(i)=LSMean(j)<br><br>Dependent Variable: acratio			
**i/j**	**1**	**2**	**3**
**1**		0.0560	<.0001
**2**	0.0560		0.2592
**3**	<.0001	0.2592	

The GLM Procedure
Least Squares Means

pt=16AB0304

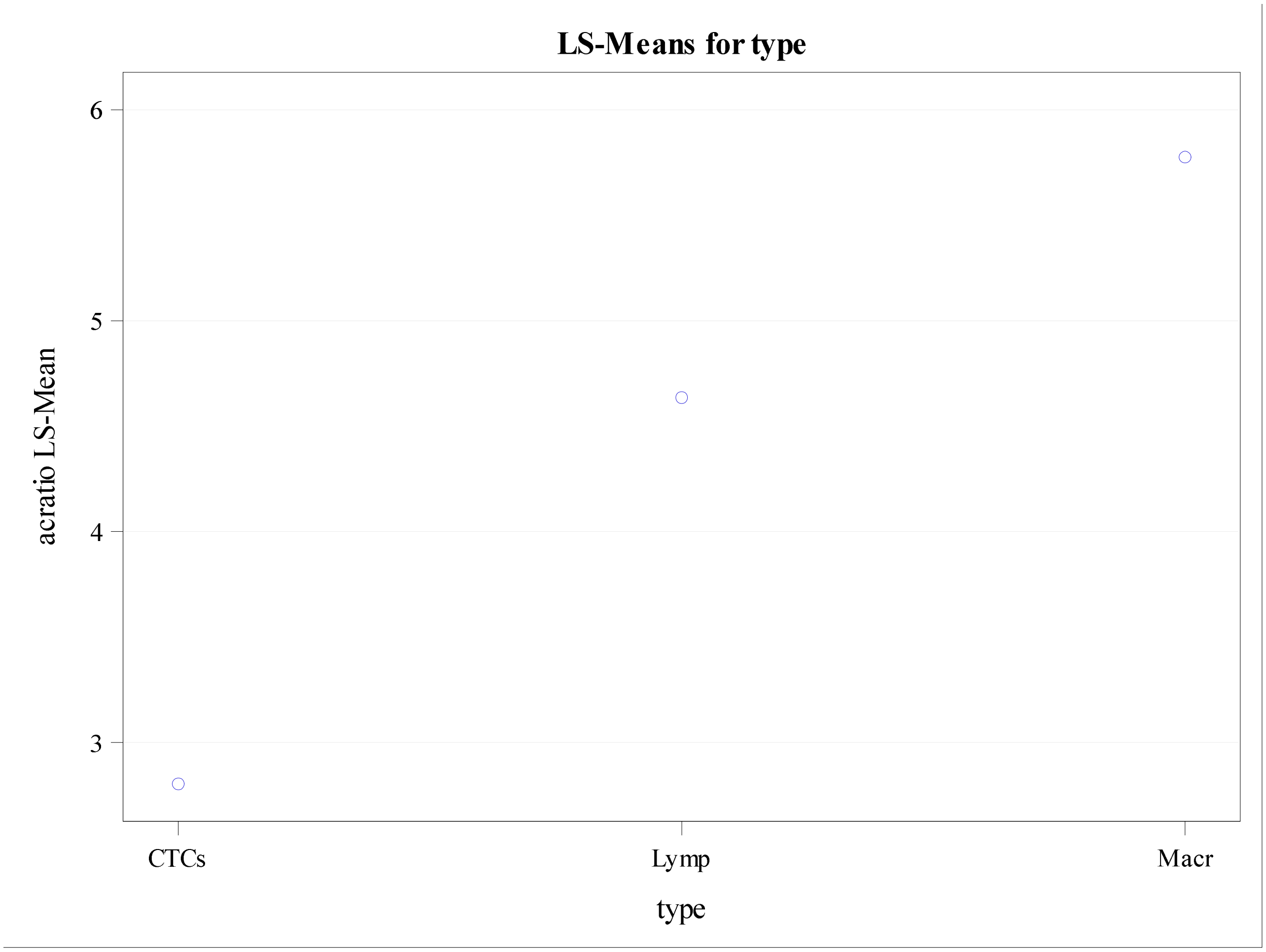

The GLM Procedure
Least Squares Means

pt=16AB0304

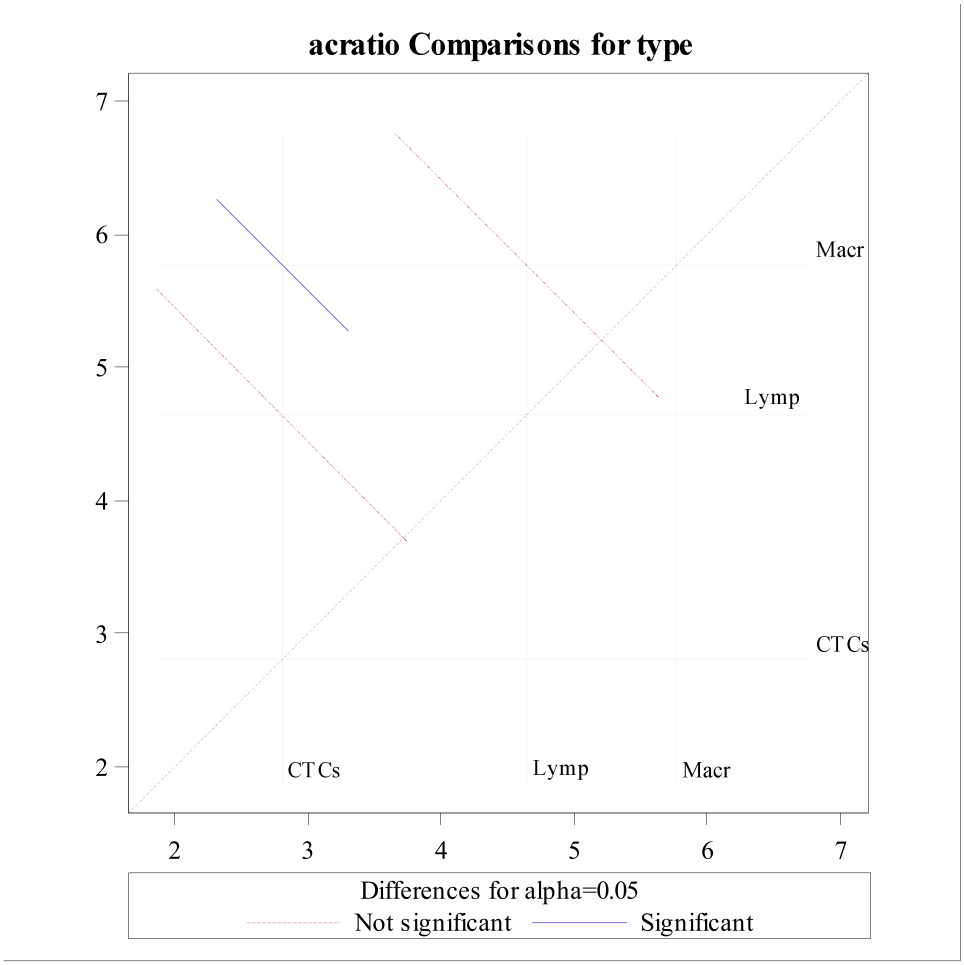

type	AvIntallsignals LSMEAN	Standard Error	Pr > |t|	LSMEAN Number
**CTCs**	8819.4850	258.0874	<.0001	1
**Lymp**	12487.7361	846.1962	<.0001	2
**Macr**	9724.4436	388.2615	<.0001	3

Least Squares Means for effect type
Pr > |t| for H0: LSMean(i)=LSMean(j)

Dependent Variable: AvIntallsignals

i/j	1	2	3
**1**		0.0001	0.0567
**2**	0.0001		0.0042
**3**	0.0567	0.0042	

The GLM Procedure
Least Squares Means

pt=16AB0304

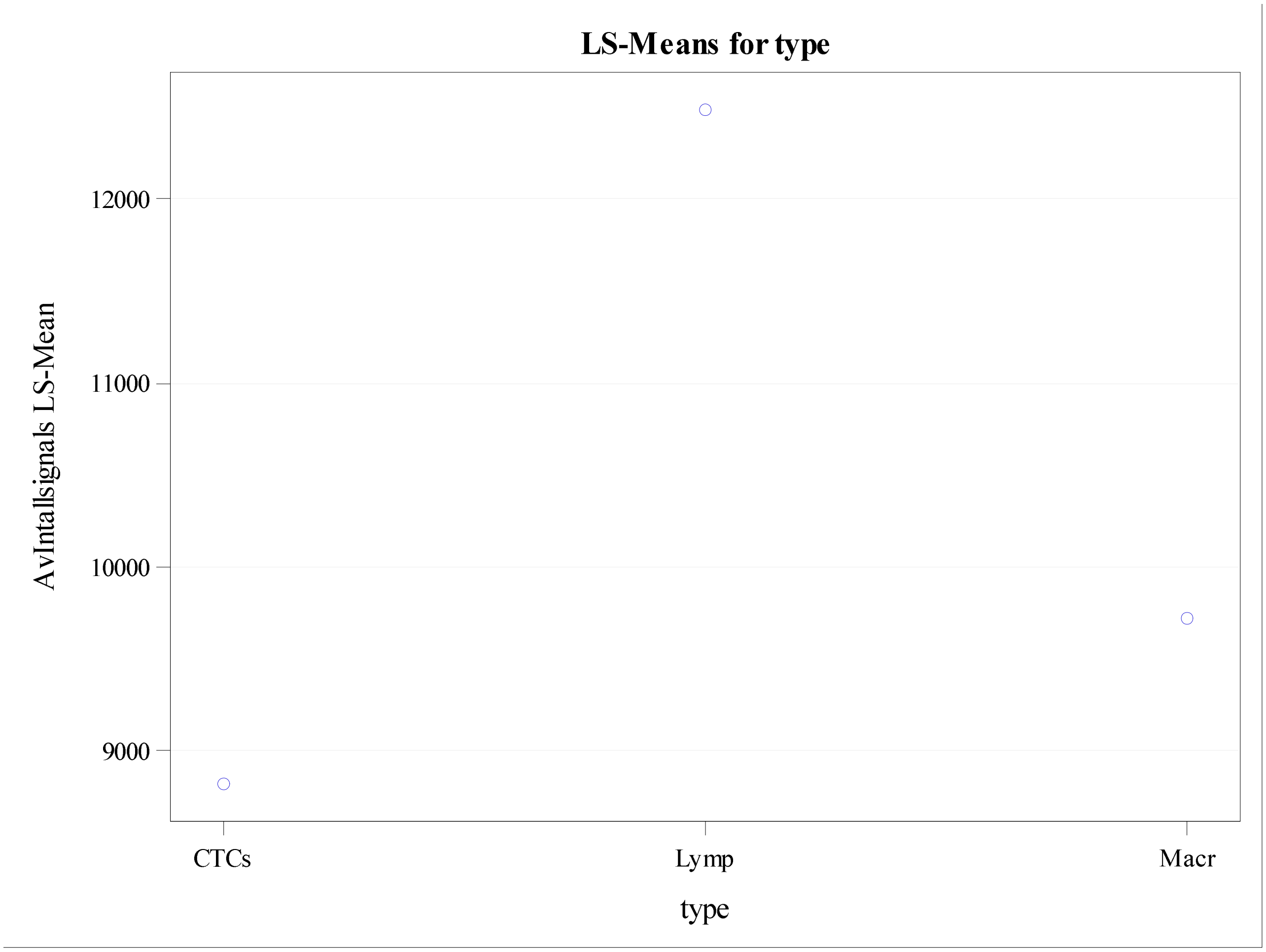

The GLM Procedure
Least Squares Means

pt=16AB0304

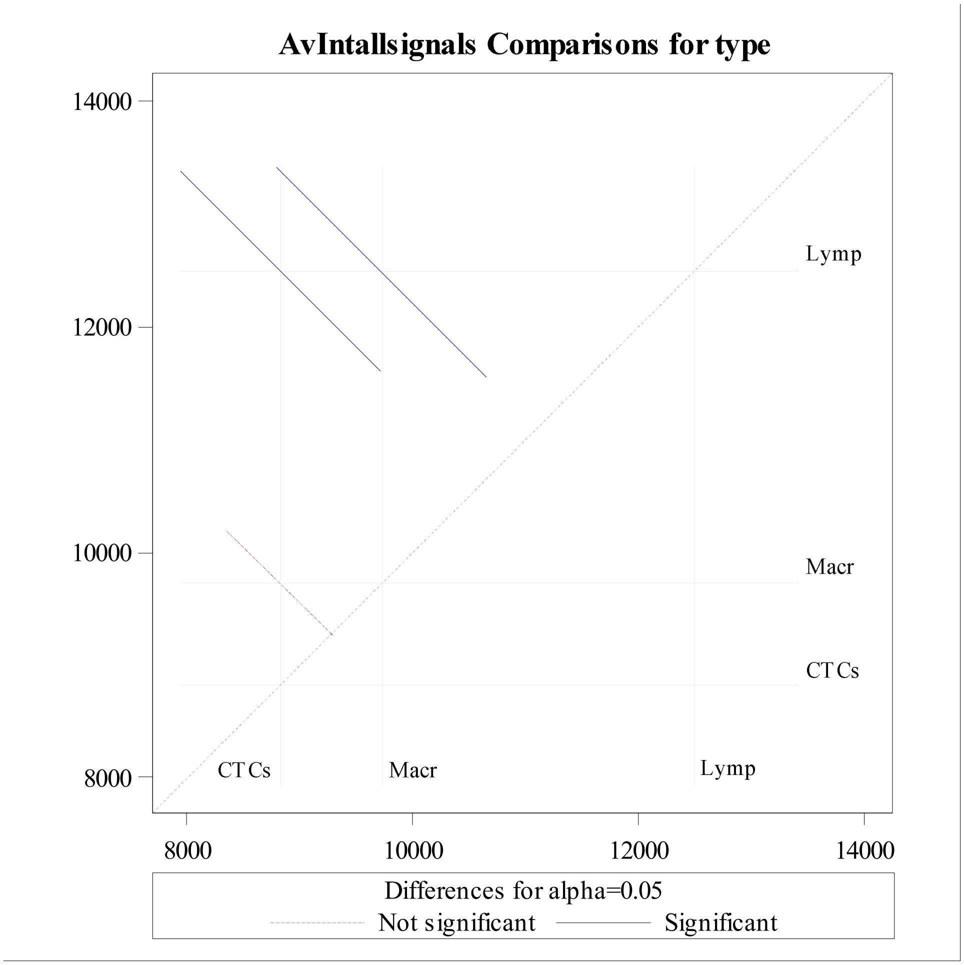

type	Totalintensity LSMEAN	Standard Error	Pr > |t|	LSMEAN Number
**CTCs**	818401.977	39705.518	<.0001	1
**Lymp**	227186.000	130183.245	0.0858	2
**Macr**	275180.263	59732.170	<.0001	3

Least Squares Means for effect type<br>Pr > |t| for H0: LSMean(i)=LSMean(j)<br><br>Dependent Variable: Totalintensity			
**i/j**	**1**	**2**	**3**
**1**		<.0001	<.0001
**2**	<.0001		0.7387
**3**	<.0001	0.7387	

The GLM Procedure
Least Squares Means

pt=16AB0304

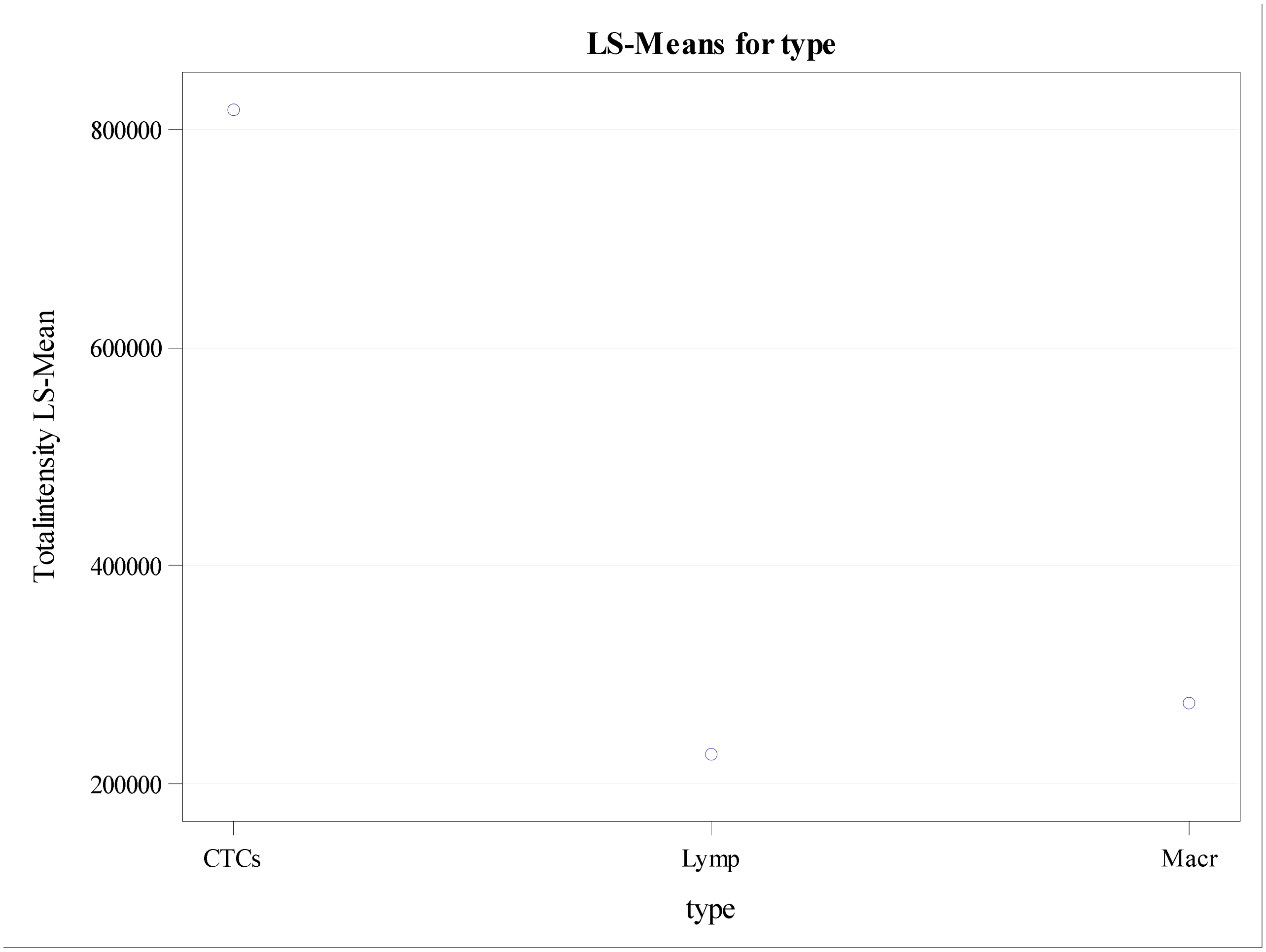

The GLM Procedure
Least Squares Means

pt=16AB0304

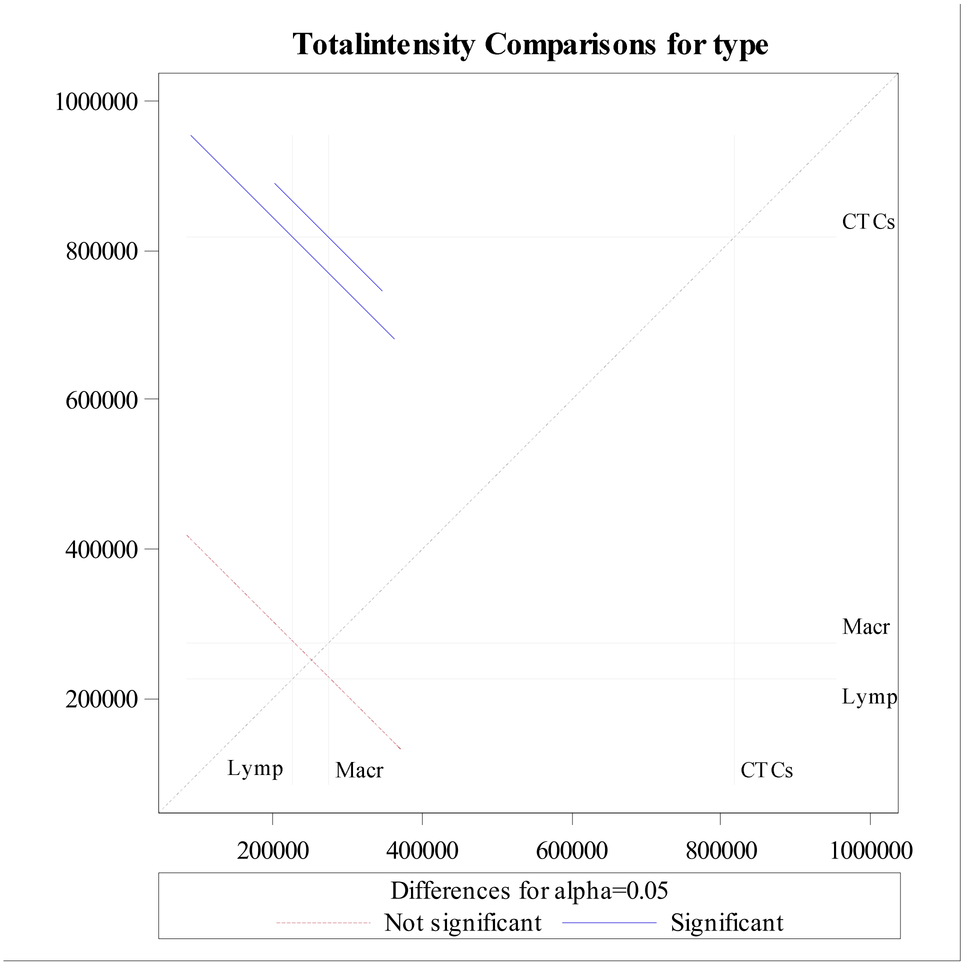

type	Nuclearvolume LSMEAN	Standard Error	Pr > |t|	LSMEAN Number
**CTCs**	937431.884	64069.454	<.0001	1
**Lymp**	416729.750	210065.753	0.0516	2
**Macr**	473442.368	96384.778	<.0001	3

Least Squares Means for effect type<br>Pr > |t| for H0: LSMean(i)=LSMean(j)<br><br>Dependent Variable: Nuclearvolume			
**i/j**	**1**	**2**	**3**
**1**		0.0208	0.0002
**2**	0.0208		0.8070
**3**	0.0002	0.8070	

The GLM Procedure
Least Squares Means

pt=16AB0304

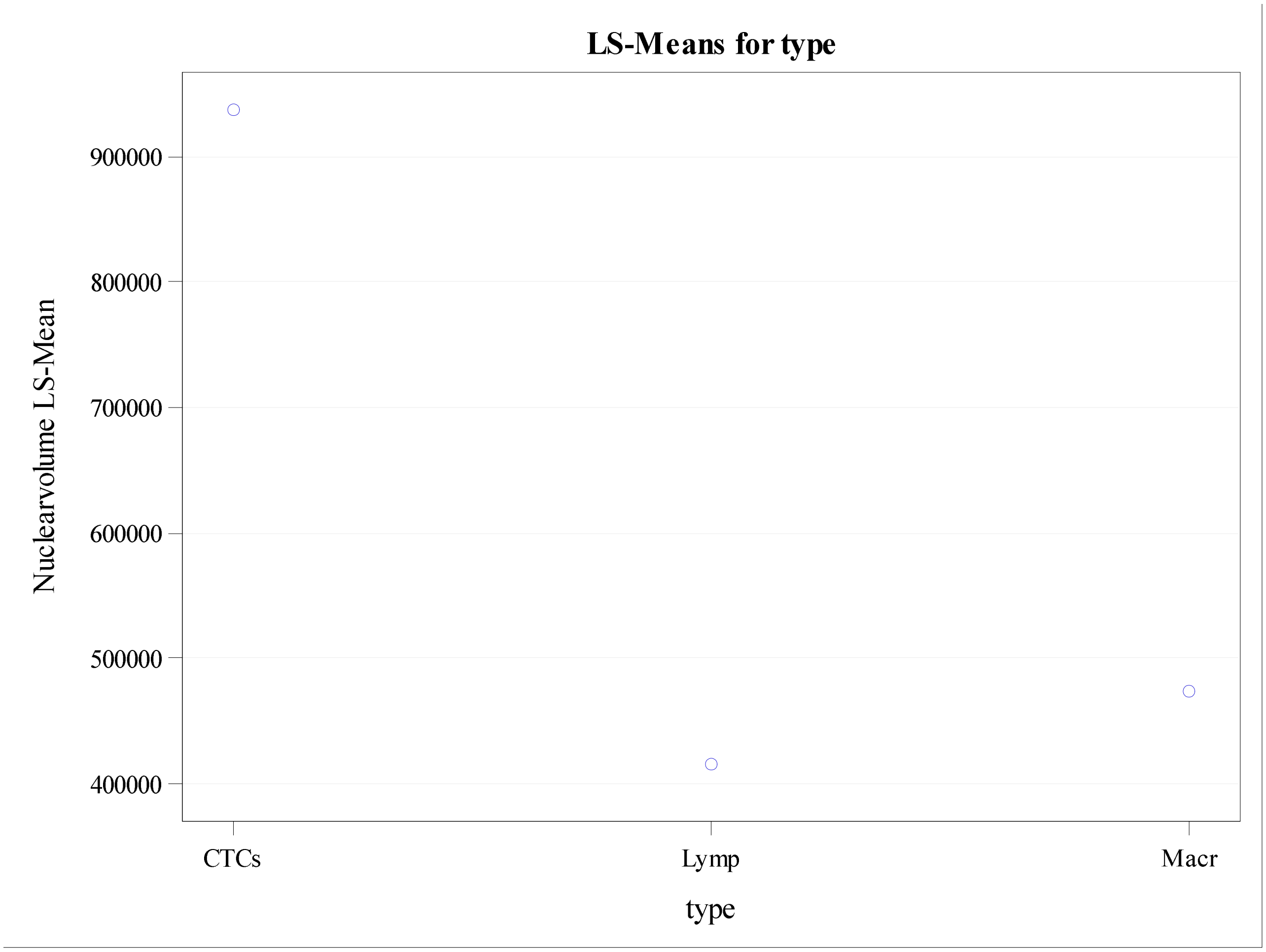

The GLM Procedure
Least Squares Means

pt=16AB0304

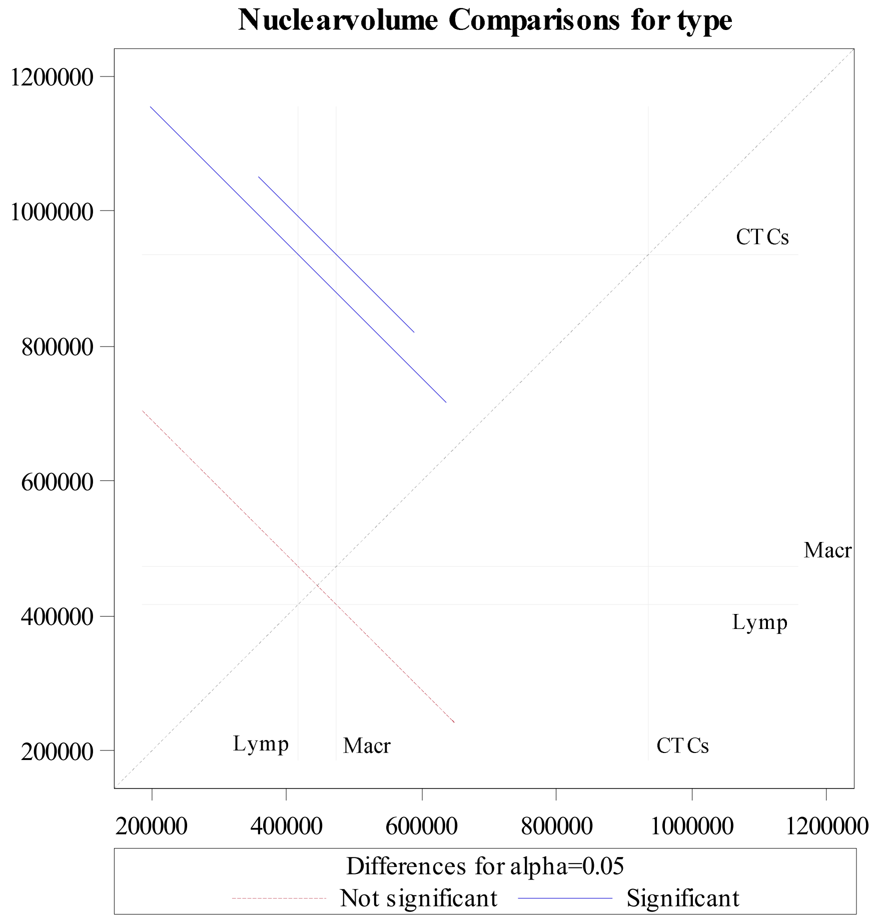

type	telomereperkvol LSMEAN	Standard Error	Pr > |t|	LSMEAN Number
**CTCs**	0.10822202	0.00334413	<.0001	1
**Lymp**	0.04493719	0.01096447	0.0001	2
**Macr**	0.06231698	0.00503084	<.0001	3

Least Squares Means for effect type<br>Pr > |t| for H0: LSMean(i)=LSMean(j)<br><br>Dependent Variable: telomereperkvol			
**i/j**	**1**	**2**	**3**
**1**		<.0001	<.0001
**2**	<.0001		0.1546
**3**	<.0001	0.1546	

The GLM Procedure
Least Squares Means

pt=16AB0304

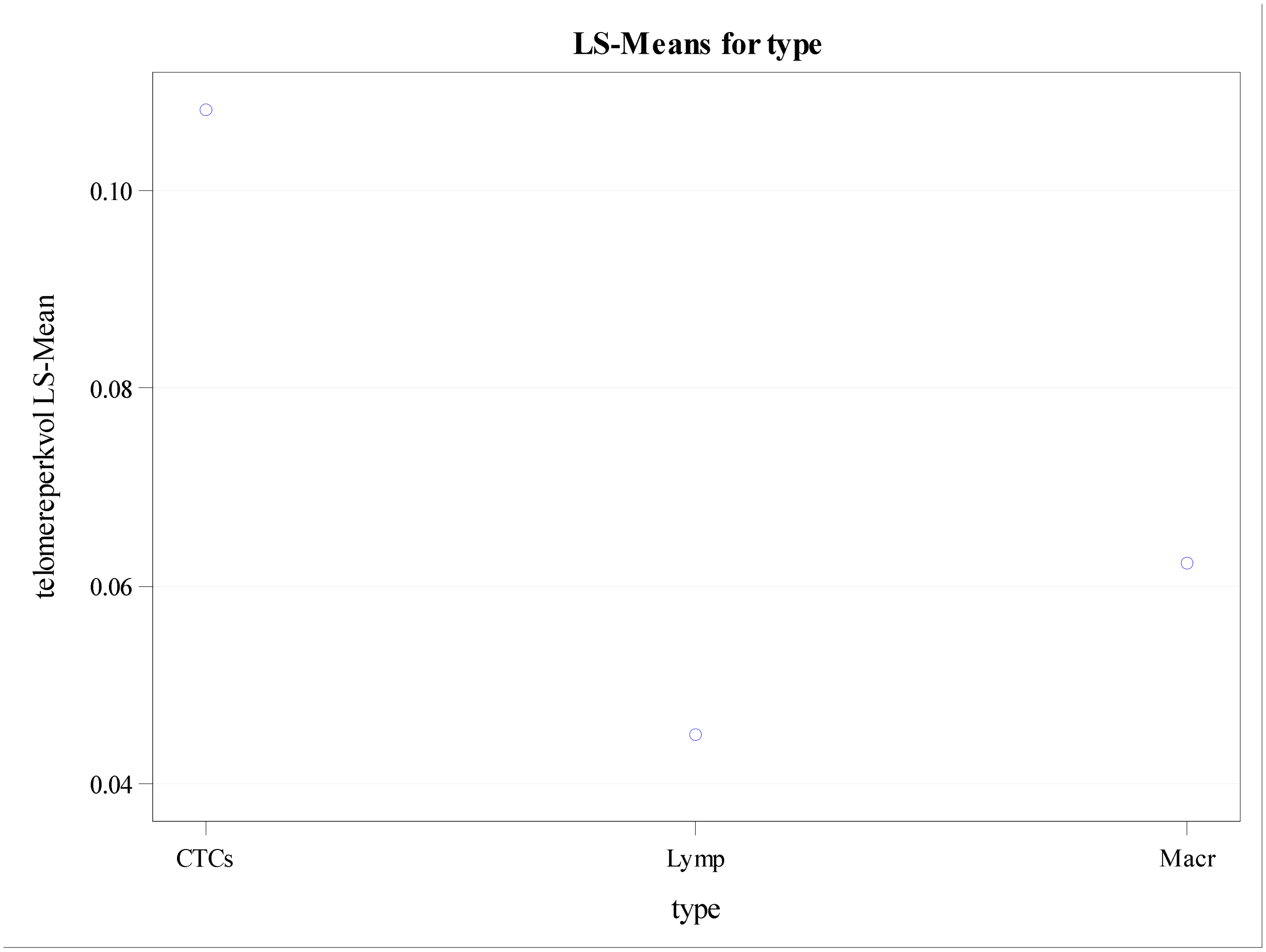

The GLM Procedure
Least Squares Means

pt=16AB0304

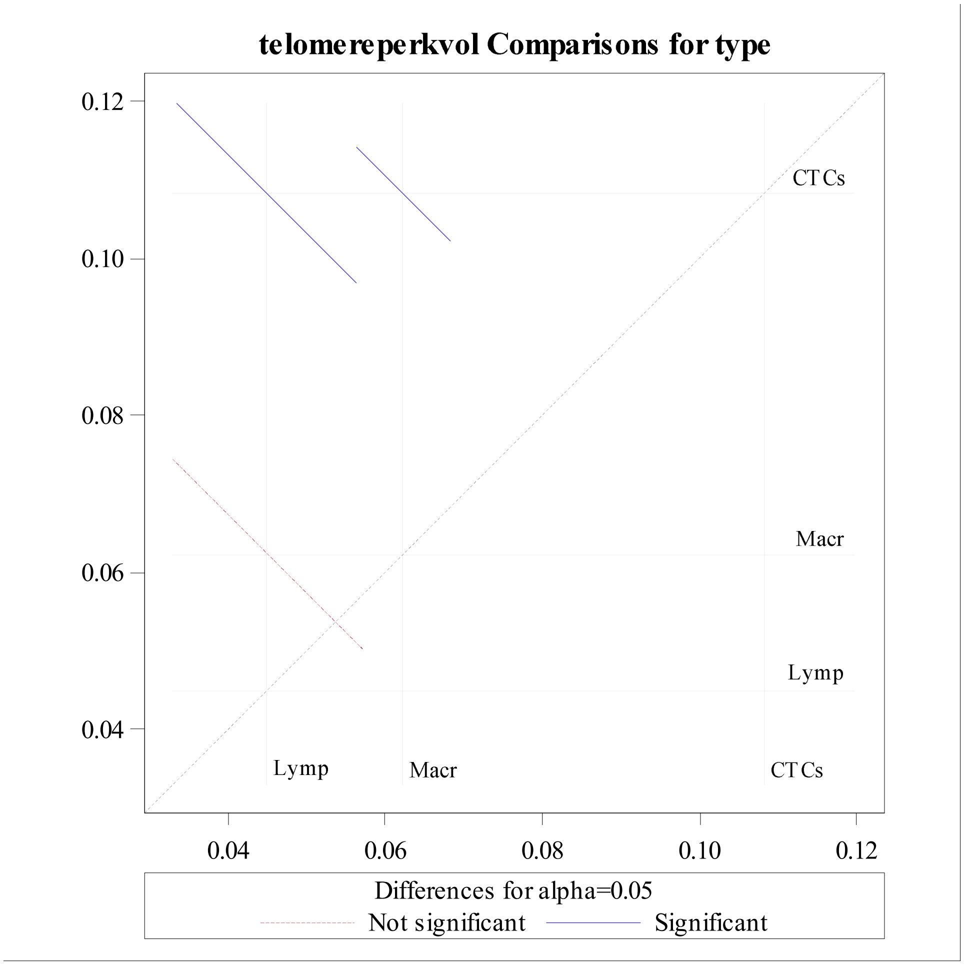

Note: To ensure overall protection level, only probabilities associated with pre-planned comparisons should be used.

The GLM Procedure

pt=17AA0828

Class Level Information		
**Class**	**Levels**	**Values**
**type**	3	CTCs Lymp Macr

**Number of Observations Read**	81
**Number of Observations Used**	81

The GLM Procedure

Dependent Variable: Totalnofsignals
Totalnofsignals

pt=17AA0828

Source	DF	Sum of Squares	Mean Square	F Value	Pr > F
**Model**	2	220600.3385	110300.1692	34.61	<.0001
**Error**	78	248557.6121	3186.6361		
**Corrected Total**	80	469157.9506			

R-Square	Coeff Var	Root MSE	Totalnofsignals Mean
0.470205	74.30085	56.45030	75.97531

Source	DF	Type III SS	Mean Square	F Value	Pr > F
**type**	2	220600.3385	110300.1692	34.61	<.0001

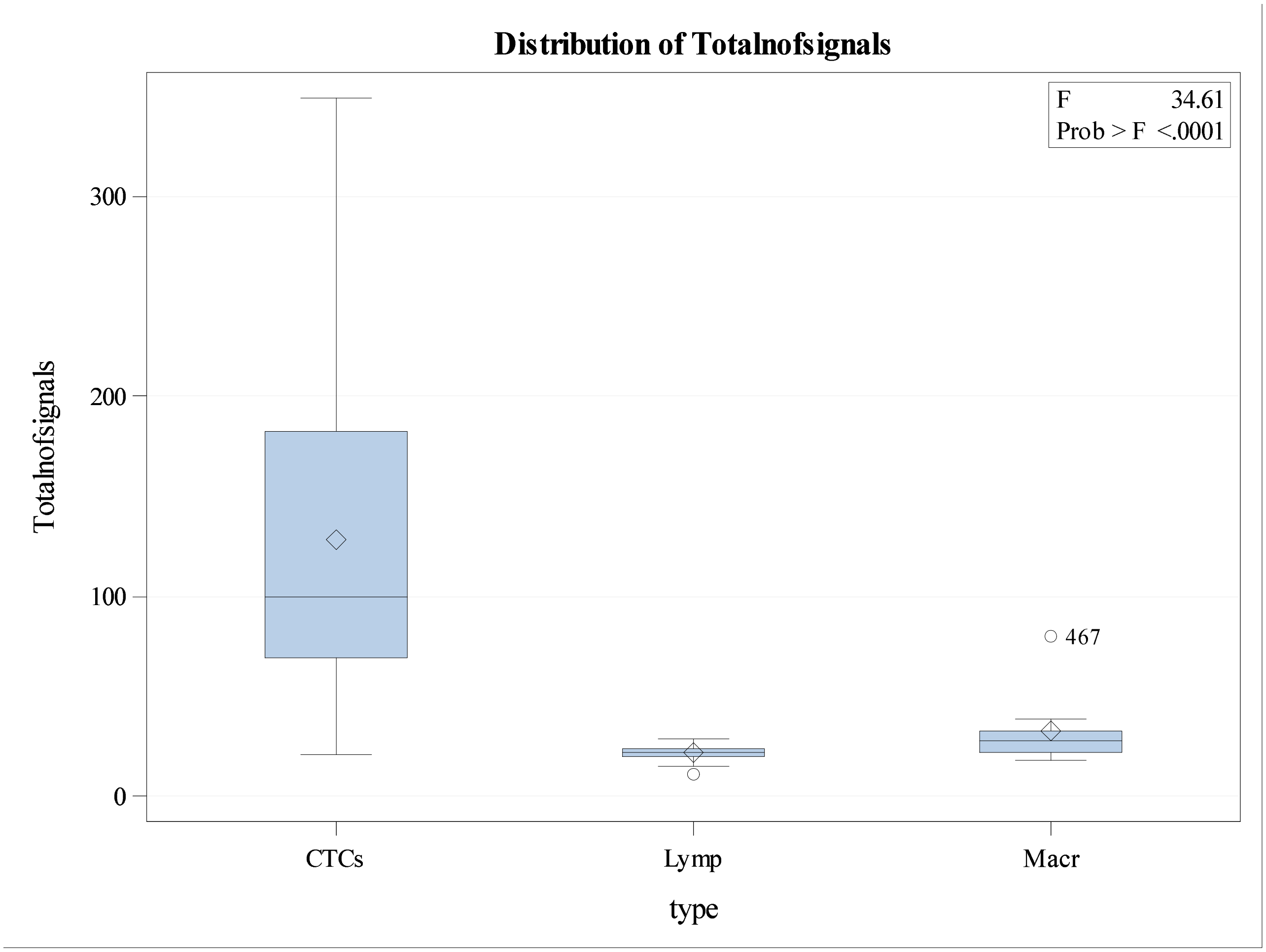

The GLM Procedure

Dependent Variable: Totalnofaggregates Totalnofaggregates

pt=17AA0828

Source	DF	Sum of Squares	Mean Square	F Value	Pr > F
**Model**	2	4123.21930	2061.60965	22.92	<.0001
**Error**	78	7017.17576	89.96379		
**Corrected Total**	80	11140.39506			

R-Square	Coeff Var	Root MSE	Totalnofaggregates Mean
0.370114	104.3857	9.484924	9.086420

Source	DF	Type III SS	Mean Square	F Value	Pr > F
**type**	2	4123.219304	2061.609652	22.92	<.0001

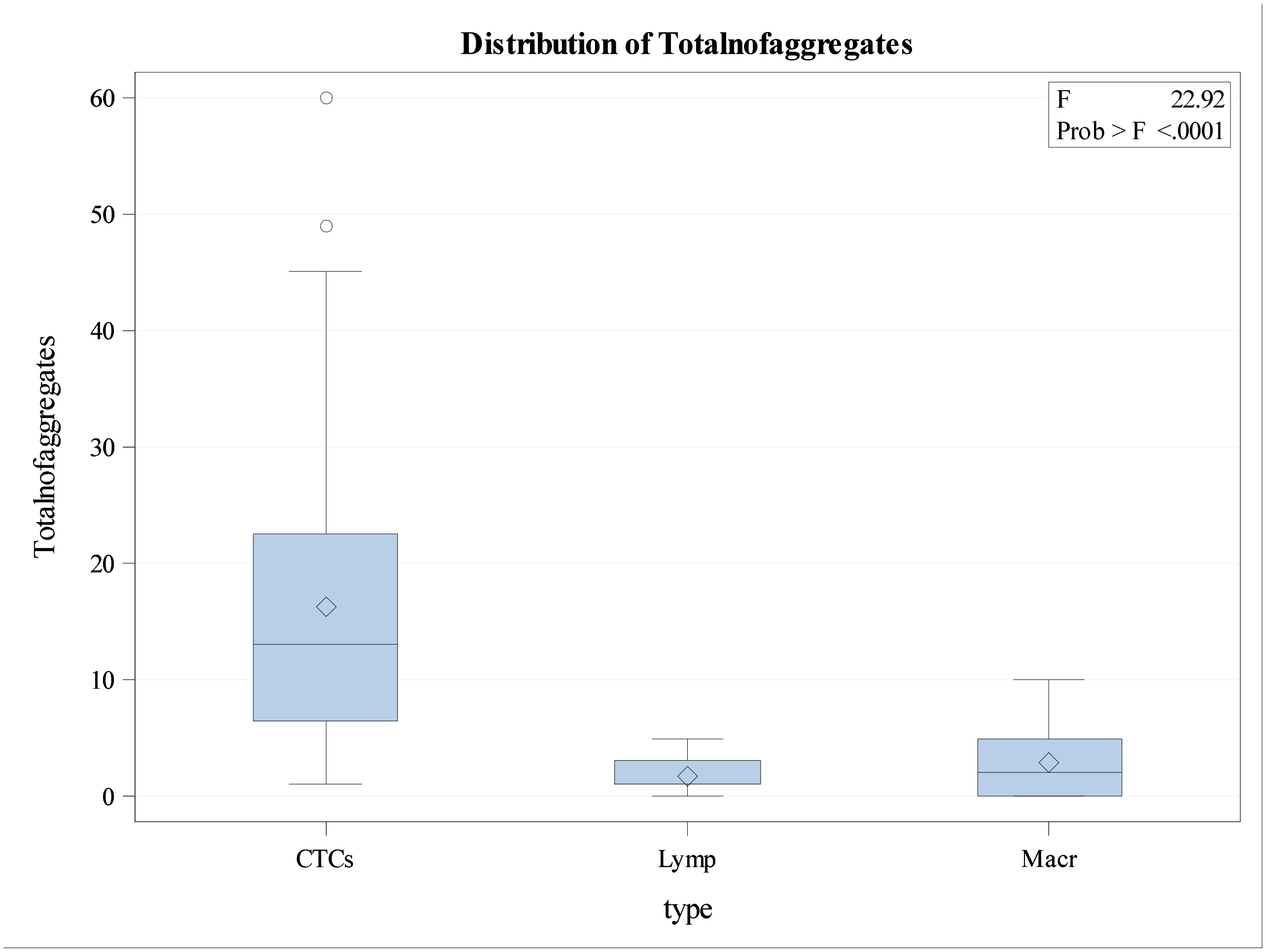

The GLM Procedure

Dependent Variable: acratio
acratio

pt=17AA0828

Source	DF	Sum of Squares	Mean Square	F Value	Pr > F
**Model**	2	67.9803130	33.9901565	31.22	<.0001
**Error**	78	84.9334397	1.0888903		
**Corrected Total**	80	152.9137527			

R-Square	Coeff Var	Root MSE	acratio Mean
0.444566	40.44454	1.043499	2.580074

Source	DF	Type III SS	Mean Square	F Value	Pr > F
**type**	2	67.98031303	33.99015652	31.22	<.0001

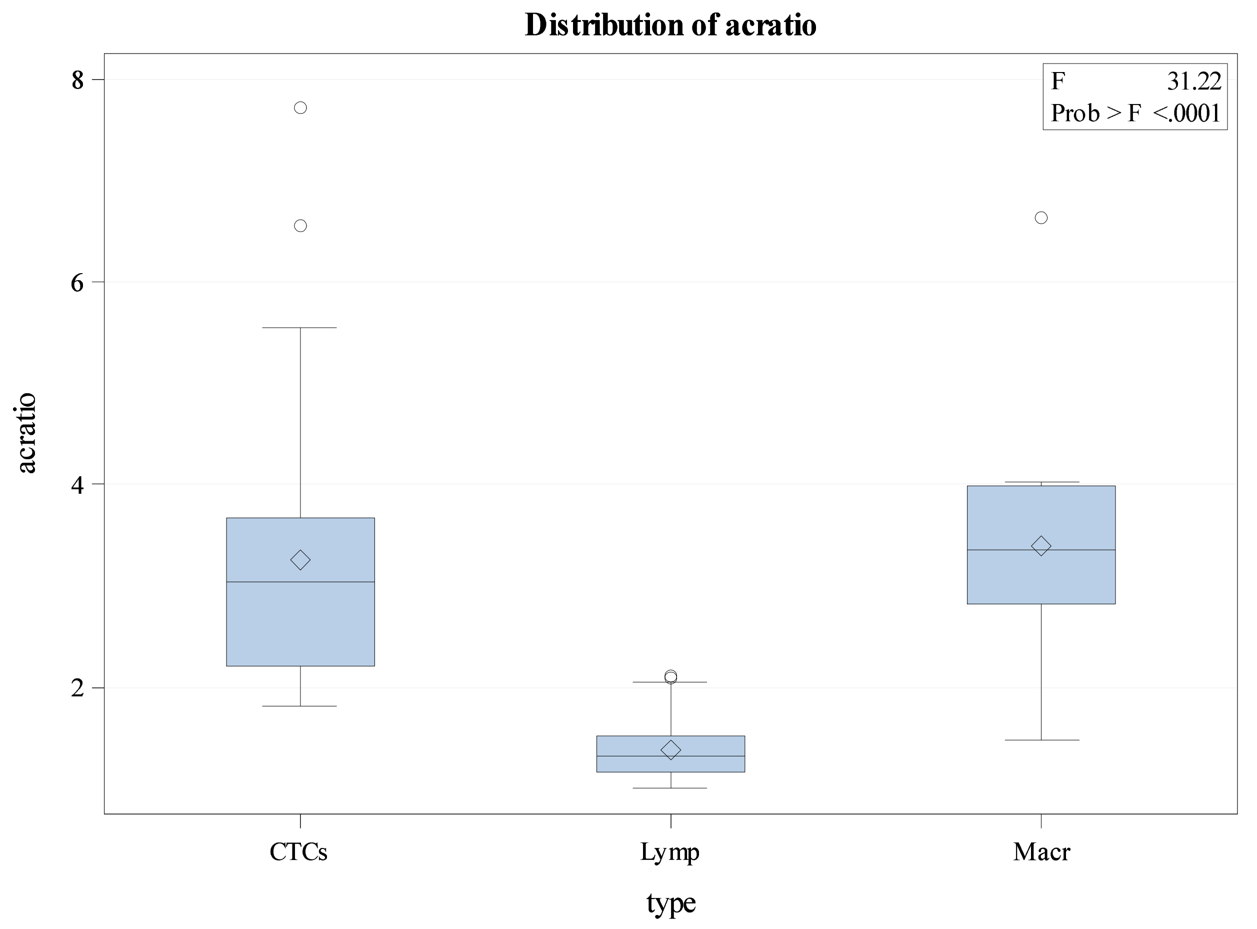

The GLM Procedure

Dependent Variable: AvIntallsignals
AvIntallsignals

pt=17AA0828

Source	DF	Sum of Squares	Mean Square	F Value	Pr > F
**Model**	2	6575299708	3287649854	166.76	<.0001
**Error**	78	1537750260	19714747		
**Corrected Total**	80	8113049968			

R-Square	Coeff Var	Root MSE	AvIntallsignals Mean
0.810460	26.17835	4440.129	16961.08

Source	DF	Type III SS	Mean Square	F Value	Pr > F
**type**	2	6575299708	3287649854	166.76	<.0001

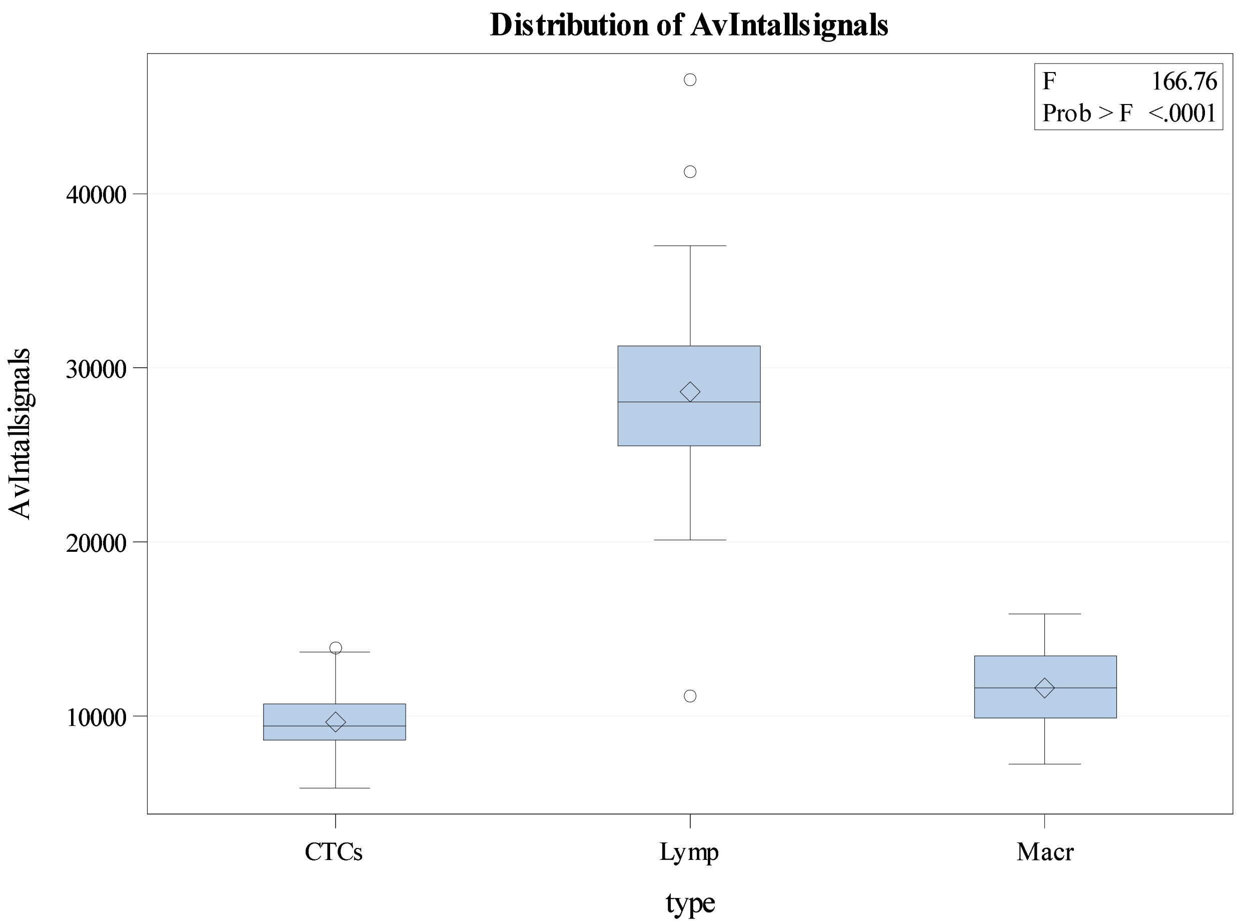

The GLM Procedure

Dependent Variable: Totalintensity
Totalintensity

pt=17AA0828

Source	DF	Sum of Squares	Mean Square	F Value	Pr > F
**Model**	2	9.6780785E12	4.8390392E12	17.70	<.0001
**Error**	78	2.1329685E13	273457503658		
**Corrected Total**	80	3.1007764E13			

R-Square	Coeff Var	Root MSE	Totalintensity Mean
0.312118	59.66239	522931.6	876484.6

Source	DF	Type III SS	Mean Square	F Value	Pr > F
**type**	2	9.6780785E12	4.8390392E12	17.70	<.0001

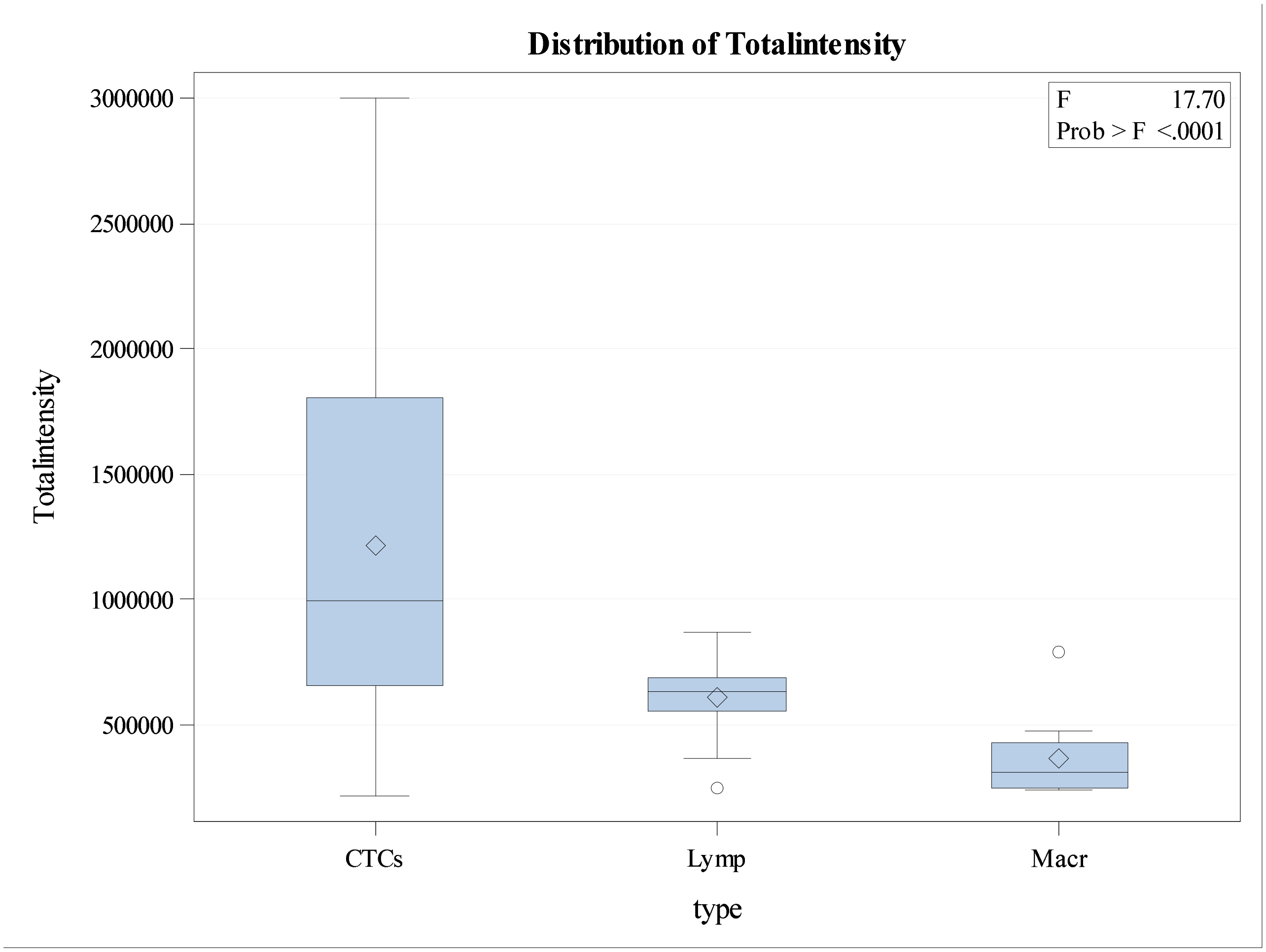

The GLM Procedure

Dependent Variable: Nuclearvolume Nuclearvolume

pt=17AA0828

Source	DF	Sum of Squares	Mean Square	F Value	Pr > F
**Model**	2	1.3242953E13	6.6214763E12	56.36	<.0001
**Error**	78	9.1640463E12	117487772980		
**Corrected Total**	80	2.2406999E13			

R-Square	Coeff Var	Root MSE	Nuclearvolume Mean
0.591019	56.52520	342764.9	606393.0

Source	DF	Type III SS	Mean Square	F Value	Pr > F
**type**	2	1.3242953E13	6.6214763E12	56.36	<.0001

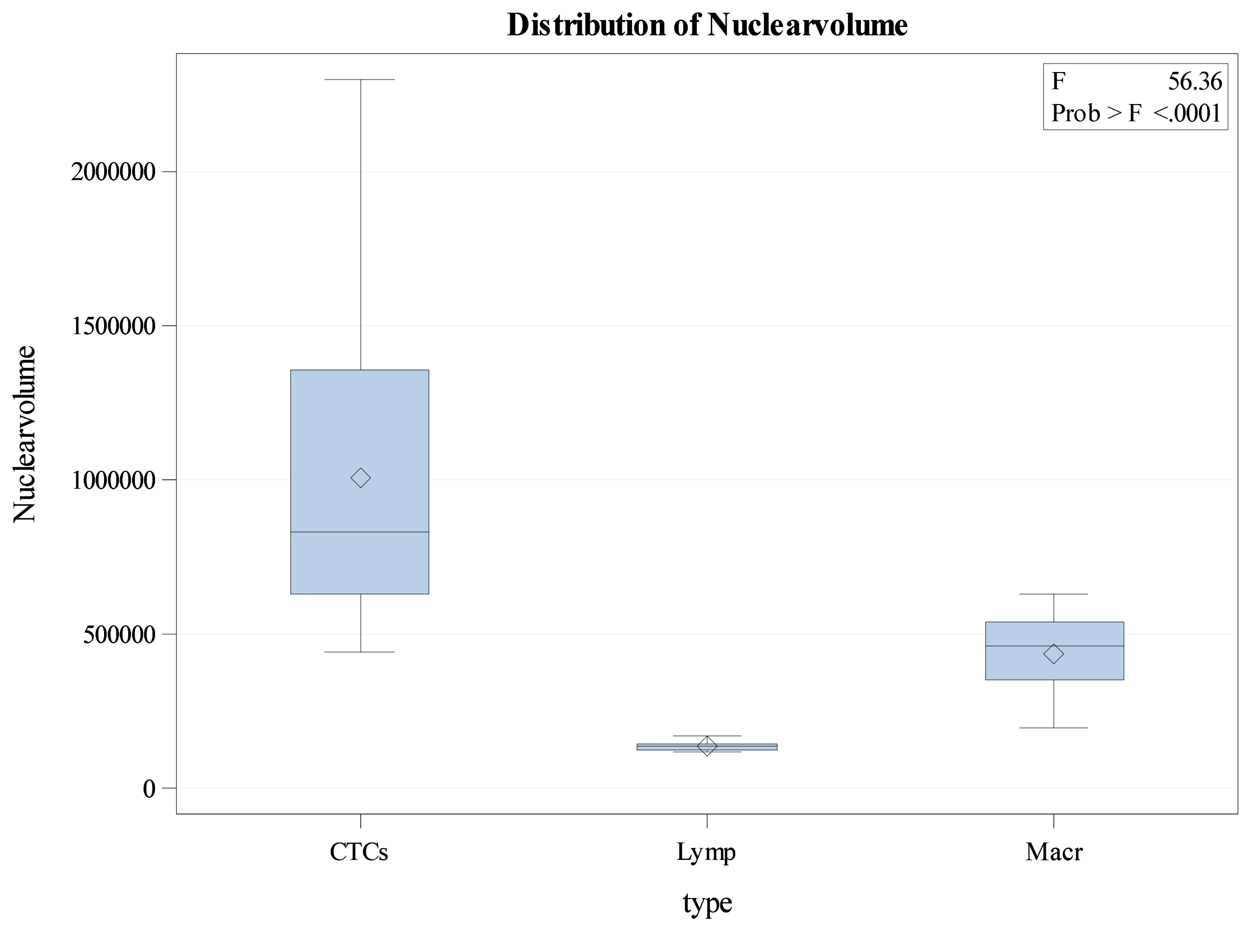

The GLM Procedure

Dependent Variable: telomereperkvol

pt=17AA0828

Source	DF	Sum of Squares	Mean Square	F Value	Pr > F
Model	2	0.04971177	0.02485588	17.92	<.0001
Error	78	0.10818698	0.00138701		
Corrected Total	80	0.15789875			

R-Square	Coeff Var	Root MSE	telomereperkvol Mean
0.314833	28.62725	0.037243	0.130095

Source	DF	Type III SS	Mean Square	F Value	Pr > F
type	2	0.04971177	0.02485588	17.92	<.0001

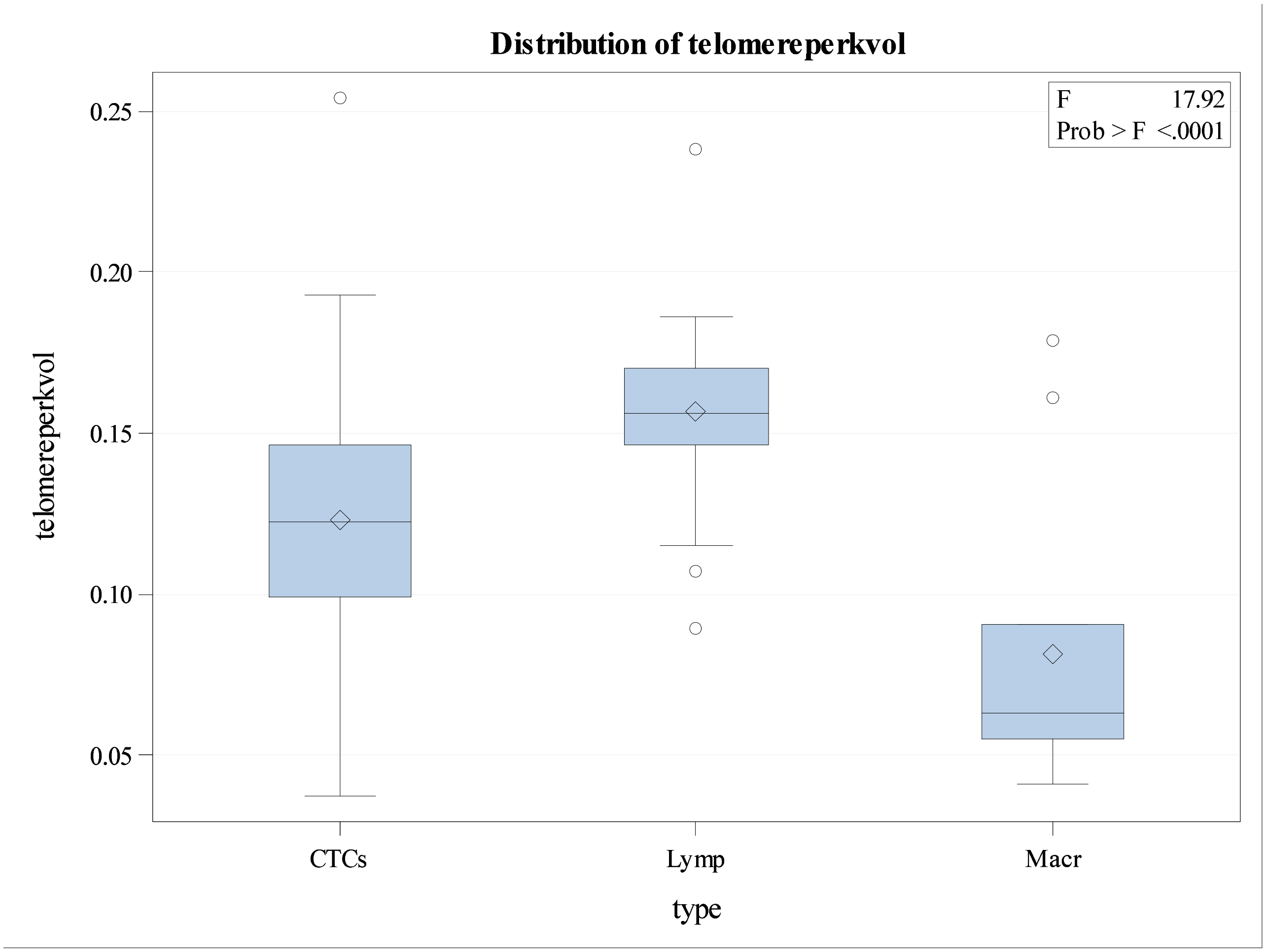

The GLM Procedure
Least Squares Means

pt=17AA0828

type	Totalnofsignals LSMEAN	Standard Error	Pr > |t|	LSMEAN Number
**CTCs**	128.700000	8.925576	<.0001	1
**Lymp**	21.666667	10.306367	0.0388	2
**Macr**	32.363636	17.020405	0.0609	3

Least Squares Means for effect type<br>Pr > |t| for H0: LSMean(i)=LSMean(j)<br><br>Dependent Variable: Totalnofsignals			
**i/j**	**1**	**2**	**3**
**1**		<.0001	<.0001
**2**	<.0001		0.5924
**3**	<.0001	0.5924	

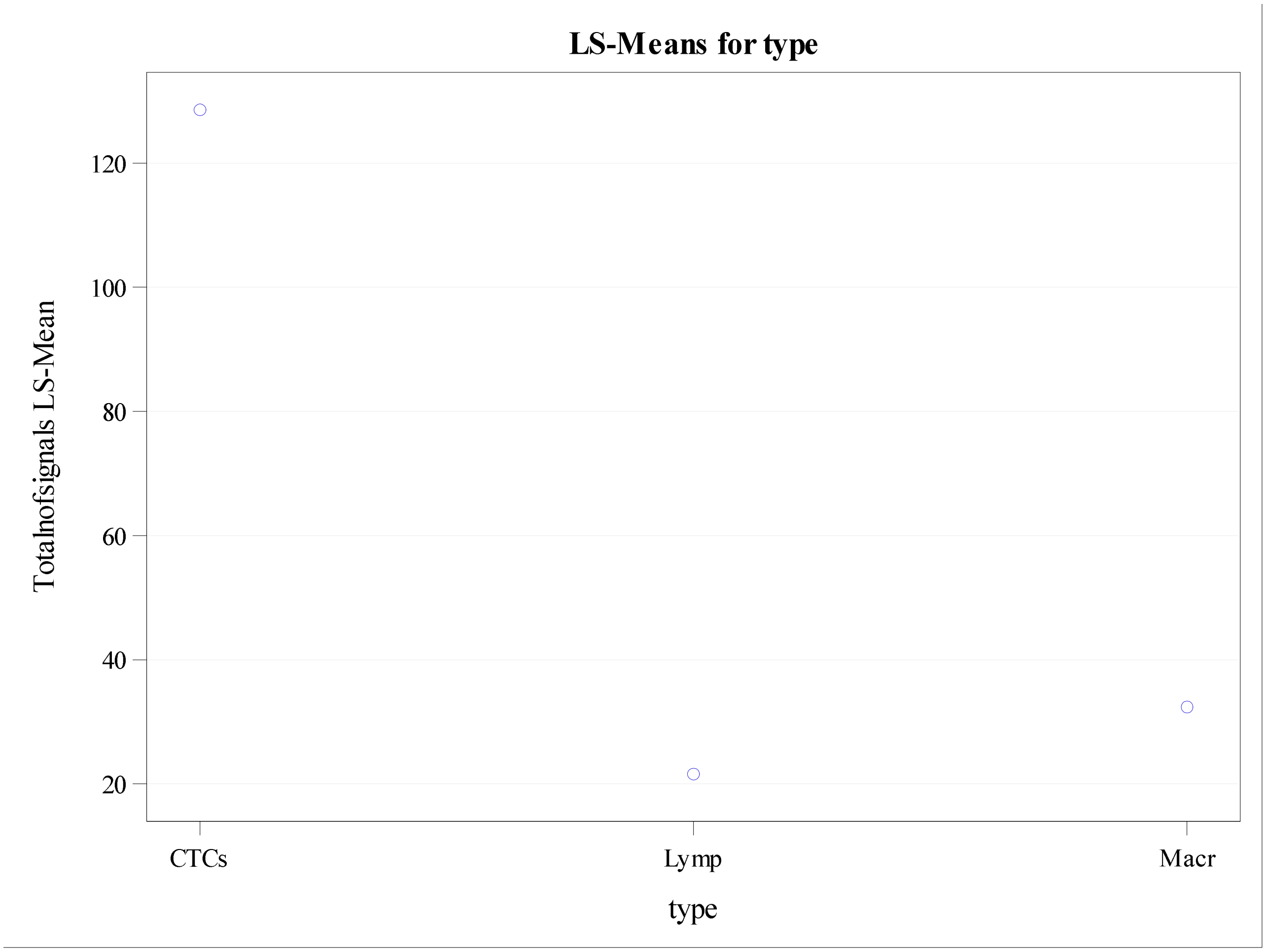

The GLM Procedure
Least Squares Means

pt=17AA0828

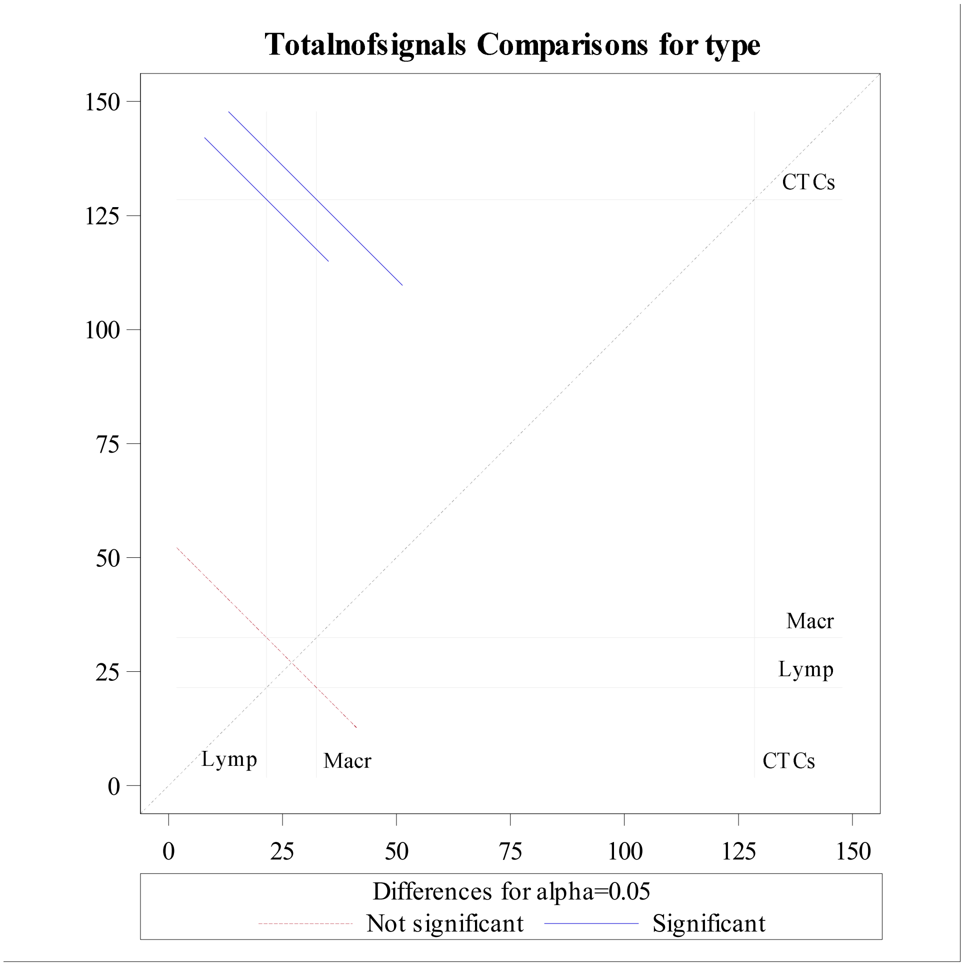

type	Totalnofaggregates LSMEAN	Standard Error	Pr > |t|	LSMEAN Number
**CTCs**	16.3000000	1.4996982	<.0001	1
**Lymp**	1.7333333	1.7317024	0.3200	2
**Macr**	2.9090909	2.8598123	0.3122	3

The GLM Procedure
Least Squares Means

pt=17AA0828

Least Squares Means for effect type<br>Pr > |t| for H0: LSMean(i)=LSMean(j)<br><br>Dependent Variable: Totalnofaggregates			
**i/j**	**1**	**2**	**3**
**1**		<.0001	<.0001
**2**	<.0001		0.7260
**3**	<.0001	0.7260	

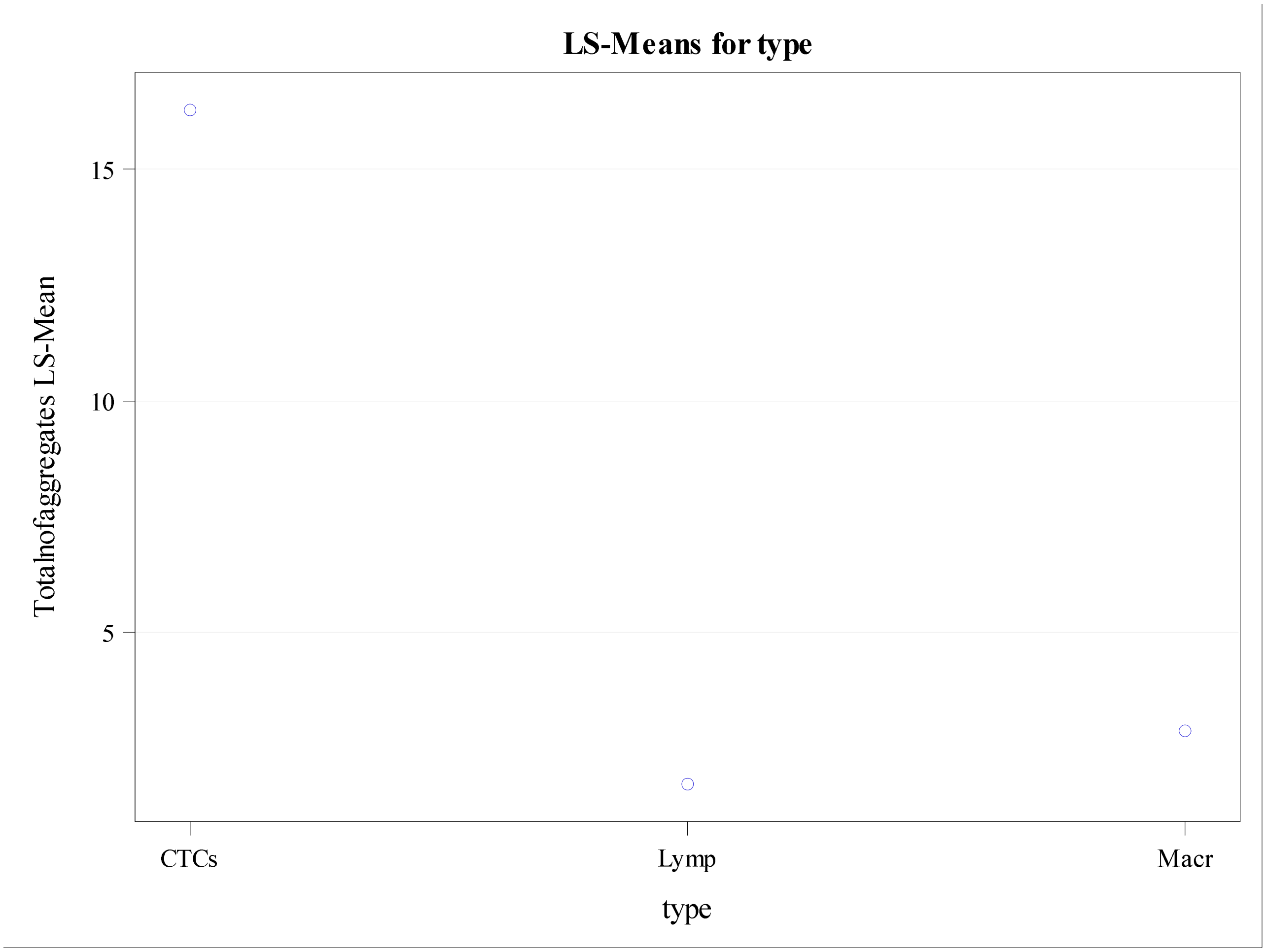

The GLM Procedure
Least Squares Means

pt=17AA0828

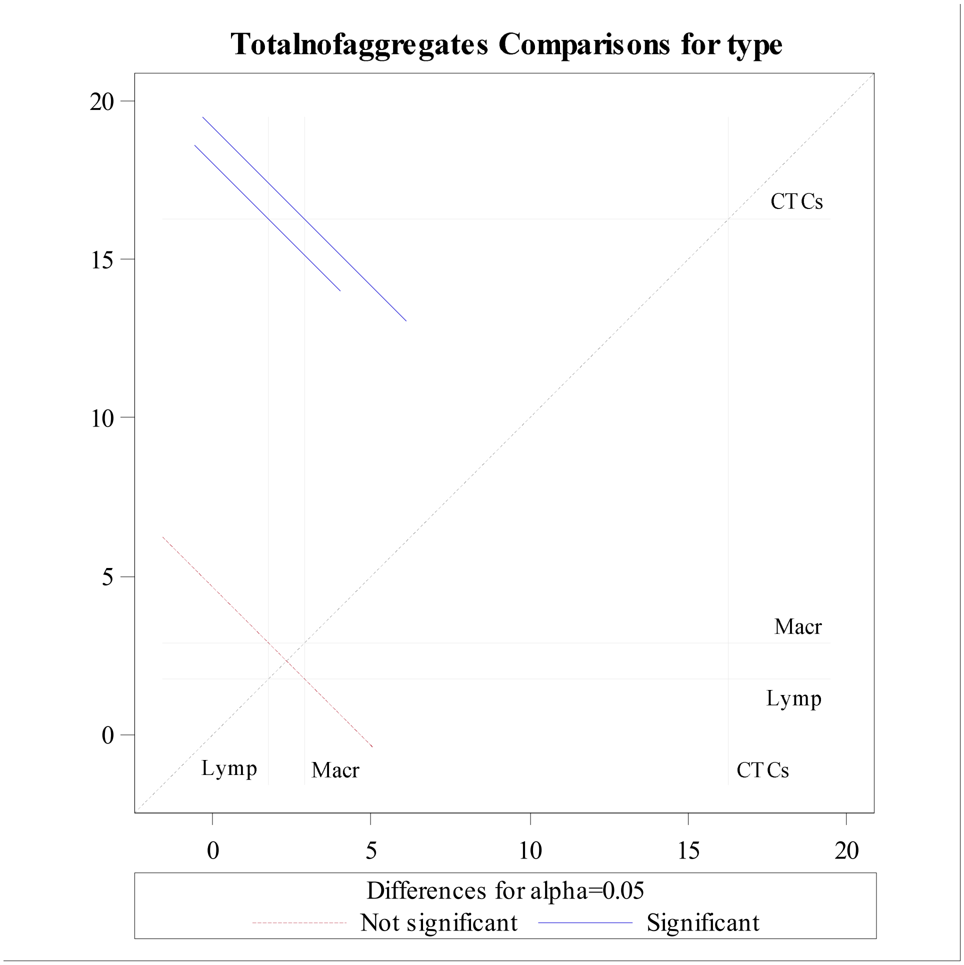

type	acratio LSMEAN	Standard Error	Pr > |t|	LSMEAN Number
**CTCs**	3.25167320	0.16499169	<.0001	1
**Lymp**	1.38709094	0.19051599	<.0001	2
**Macr**	3.39148334	0.31462680	<.0001	3

Least Squares Means for effect type<br>Pr > |t| for H0: LSMean(i)=LSMean(j)<br><br>Dependent Variable: acratio			
**i/j**	**1**	**2**	**3**
**1**		<.0001	0.6950
**2**	<.0001		<.0001
**3**	0.6950	<.0001	

The GLM Procedure
Least Squares Means

pt=17AA0828

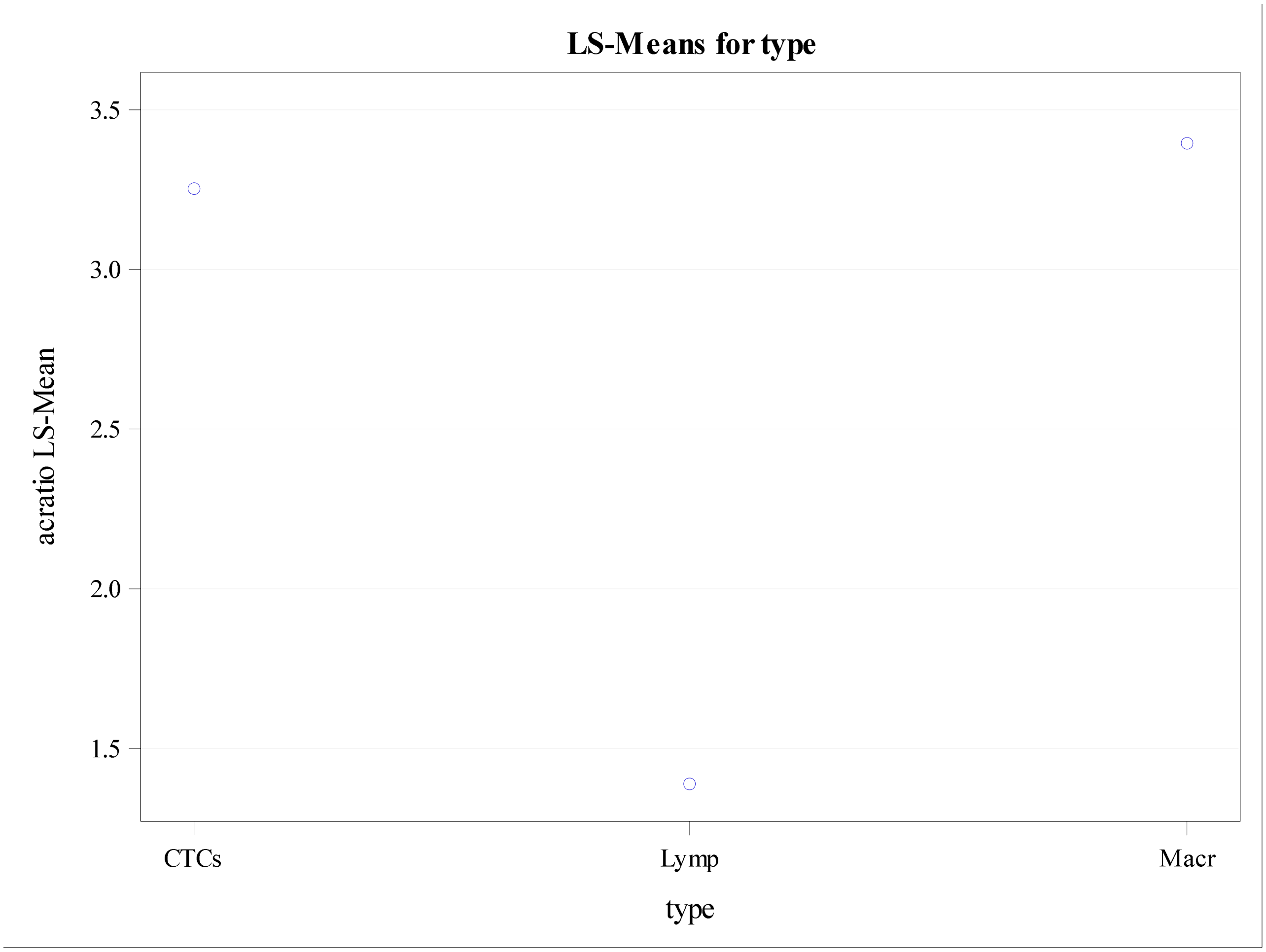

The GLM Procedure
Least Squares Means

pt=17AA0828

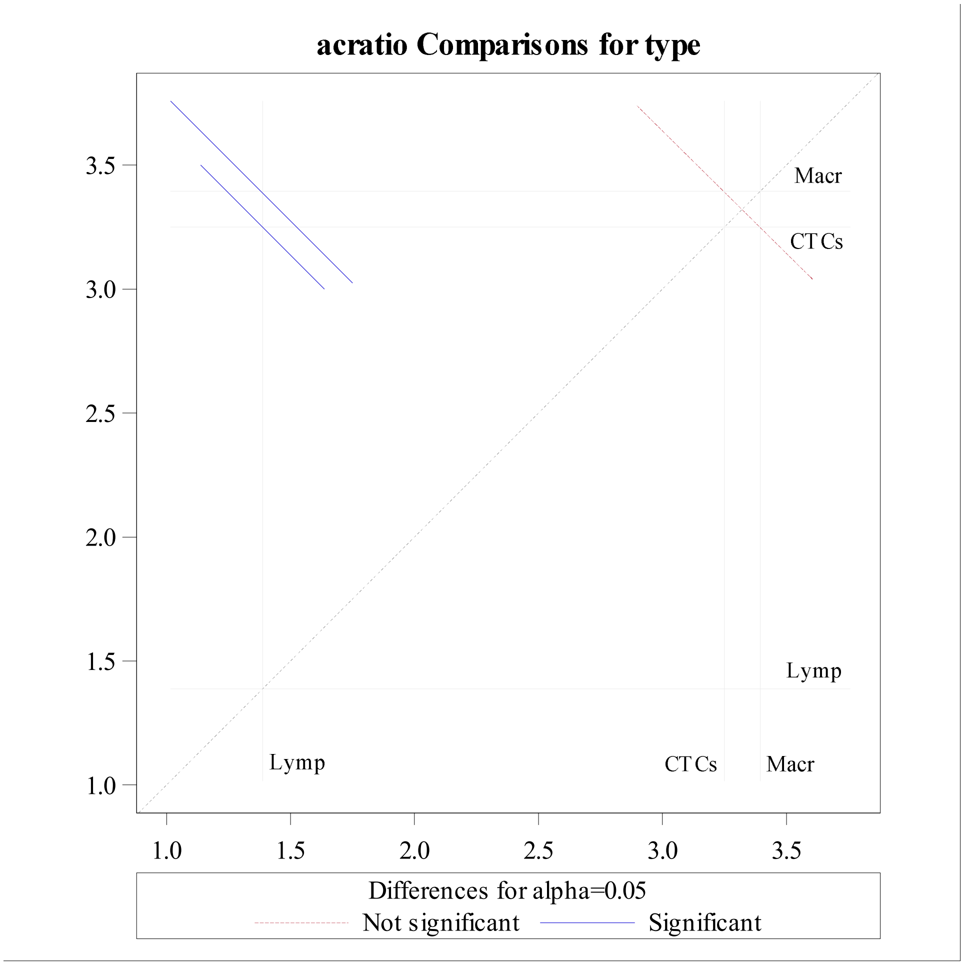

type	AvIntallsignals LSMEAN	Standard Error	Pr > |t|	LSMEAN Number
**CTCs**	9647.5311	702.0461	<.0001	1
**Lymp**	28679.0768	810.6530	<.0001	2
**Macr**	11597.5979	1338.7493	<.0001	3

Least Squares Means for effect type<br>Pr > |t| for H0: LSMean(i)=LSMean(j)<br><br>Dependent Variable: AvIntallsignals			
**i/j**	**1**	**2**	**3**
**1**		<.0001	0.2009
**2**	<.0001		<.0001
**3**	0.2009	<.0001	

The GLM Procedure
Least Squares Means

pt=17AA0828

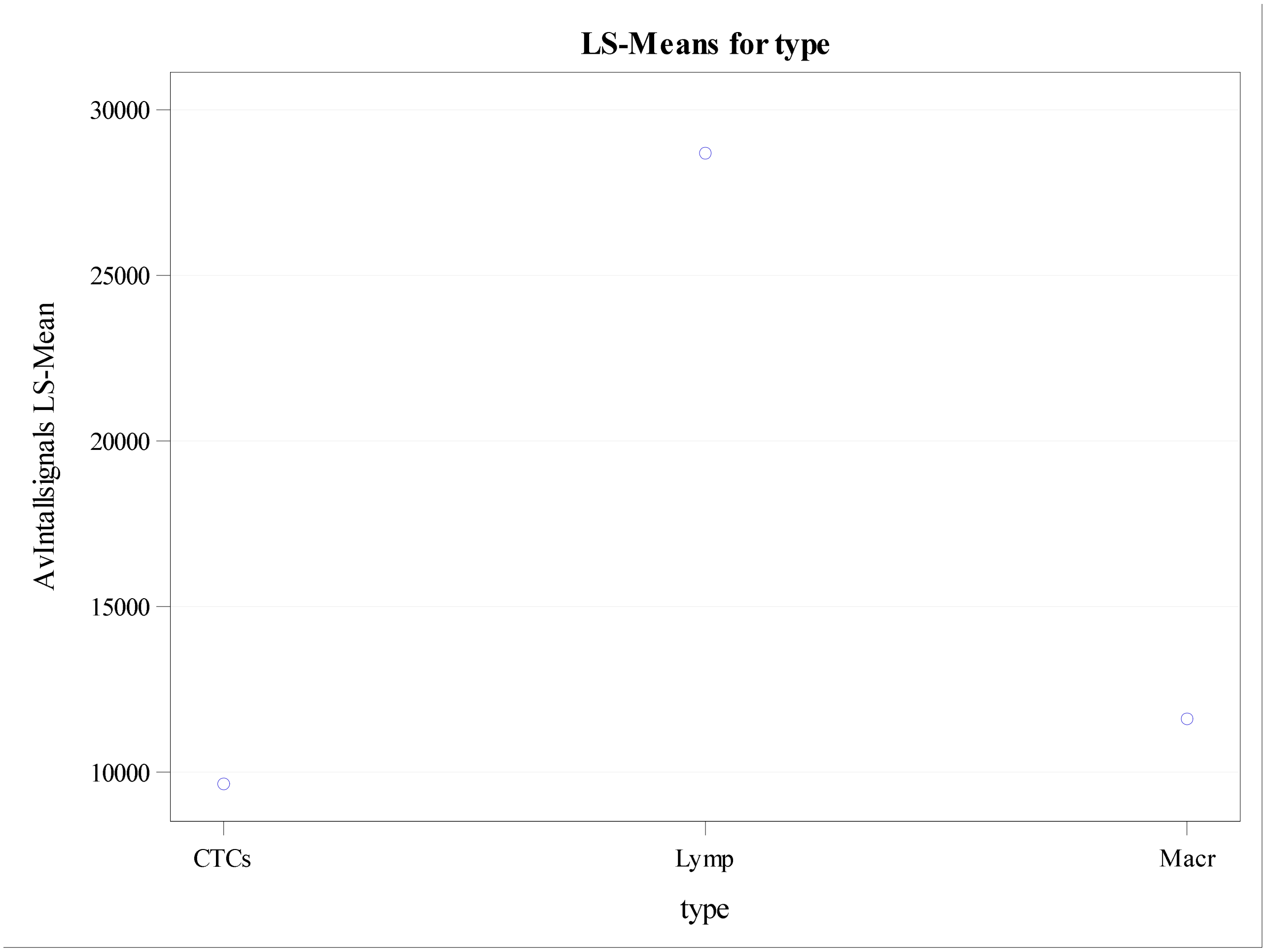

The GLM Procedure
Least Squares Means

pt=17AA0828

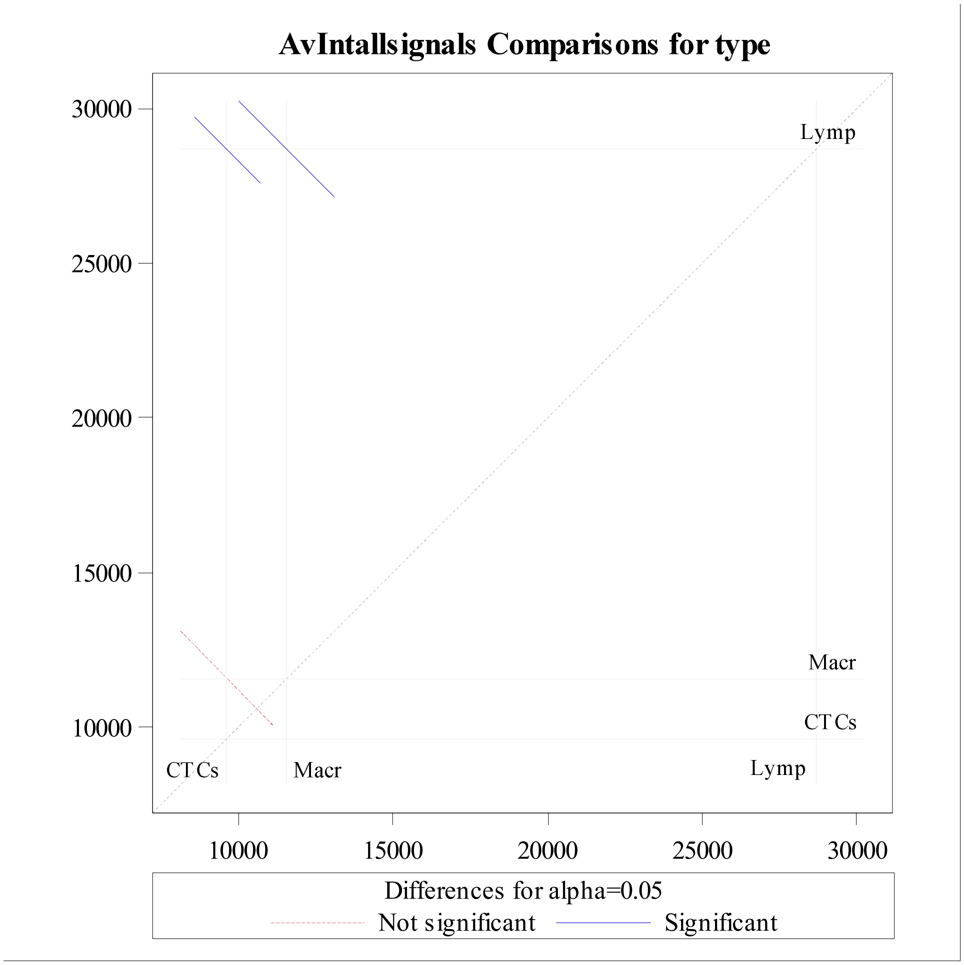

type	Totalintensity LSMEAN	Standard Error	Pr > |t|	LSMEAN Number
**CTCs**	1217496.18	82682.75	<.0001	1
**Lymp**	609881.10	95473.82	<.0001	2
**Macr**	363543.00	157669.82	0.0238	3

Least Squares Means for effect type<br>Pr > |t| for H0: LSMean(i)=LSMean(j)<br><br>Dependent Variable: Totalintensity			
**i/j**	**1**	**2**	**3**
**1**		<.0001	<.0001
**2**	<.0001		0.1853
**3**	<.0001	0.1853	

The GLM Procedure
Least Squares Means

pt=17AA0828

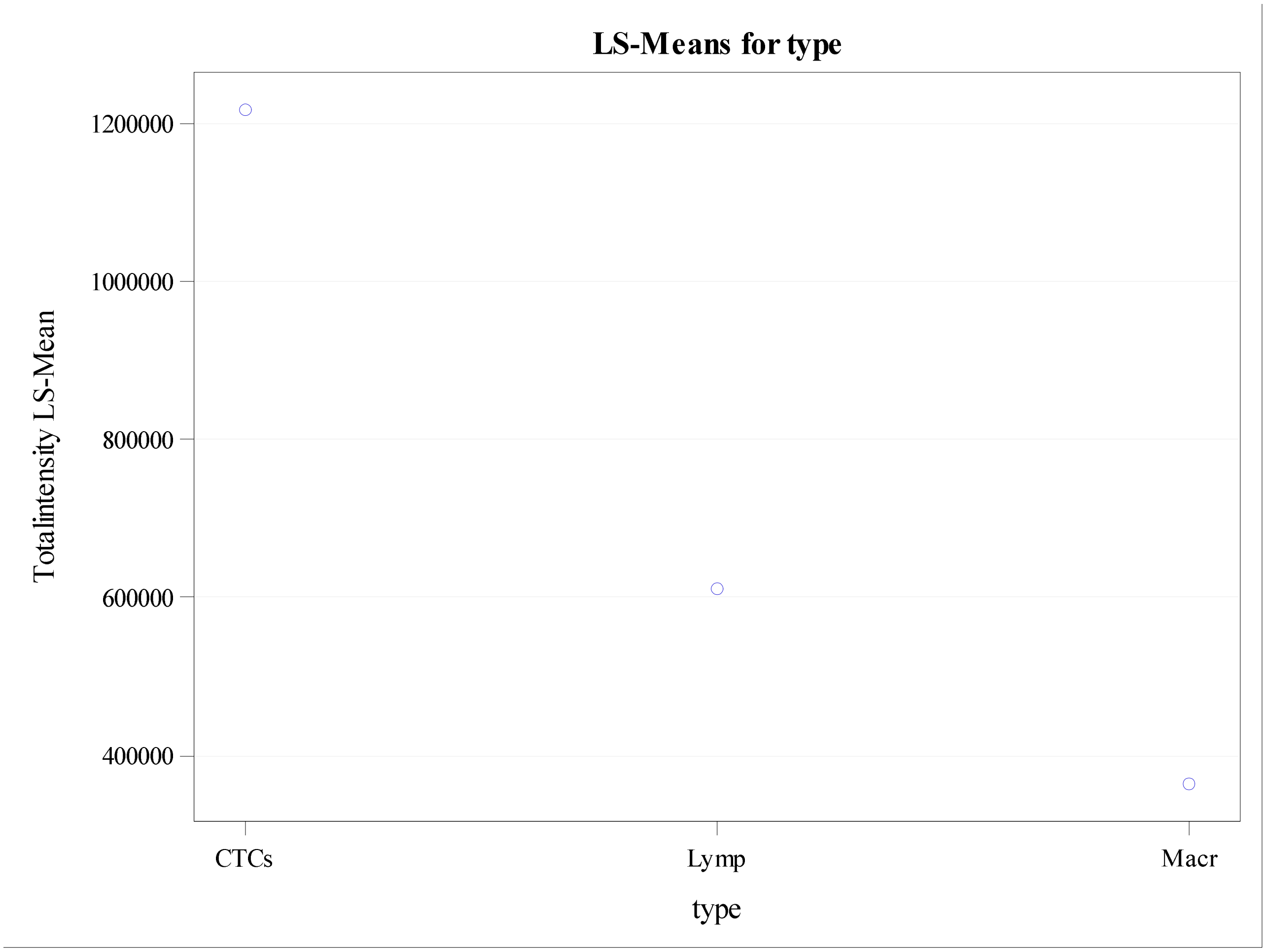

The GLM Procedure
Least Squares Means

pt=17AA0828

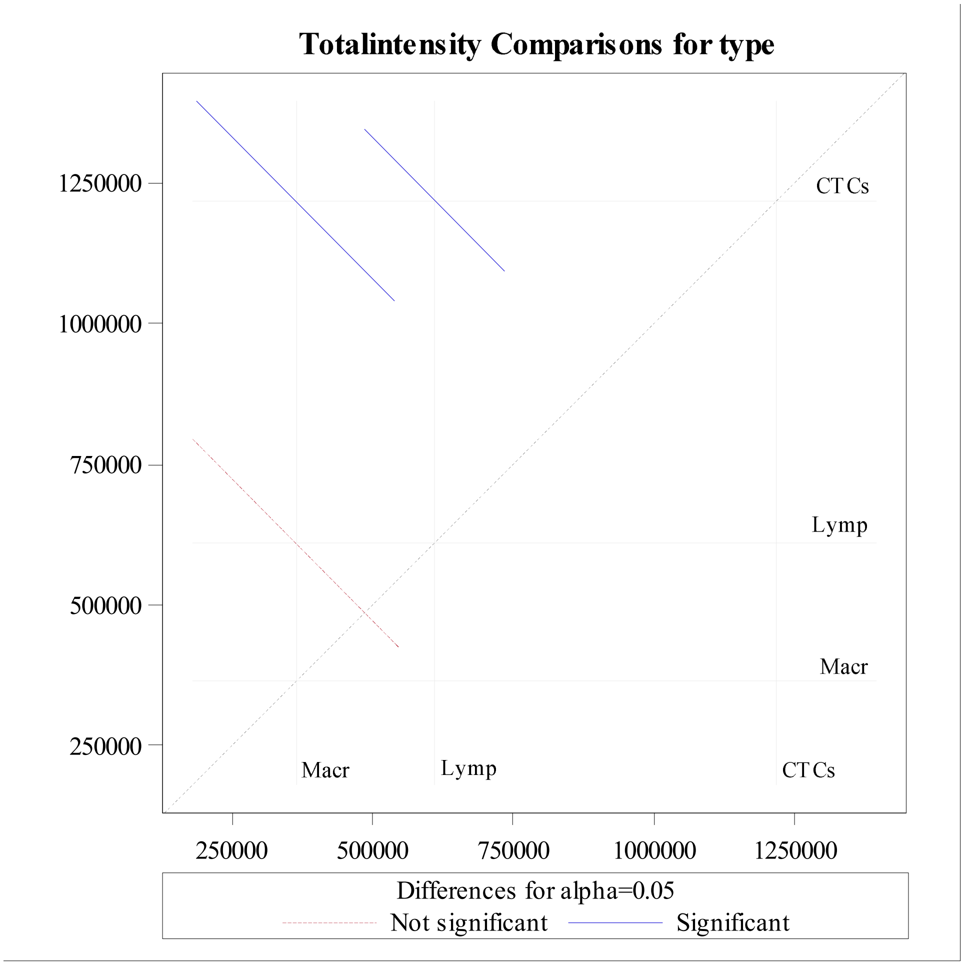

type	Nuclearvolume LSMEAN	Standard Error	Pr > |t|	LSMEAN Number
**CTCs**	1004742.90	54195.89	<.0001	1
**Lymp**	138459.67	62580.02	0.0299	2
**Macr**	434030.09	103347.50	<.0001	3

Least Squares Means for effect type<br>Pr > |t| for H0: LSMean(i)=LSMean(j)<br><br>Dependent Variable: Nuclearvolume			
**i/j**	**1**	**2**	**3**
**1**		<.0001	<.0001
**2**	<.0001		0.0167
**3**	<.0001	0.0167	

The GLM Procedure
Least Squares Means

pt=17AA0828

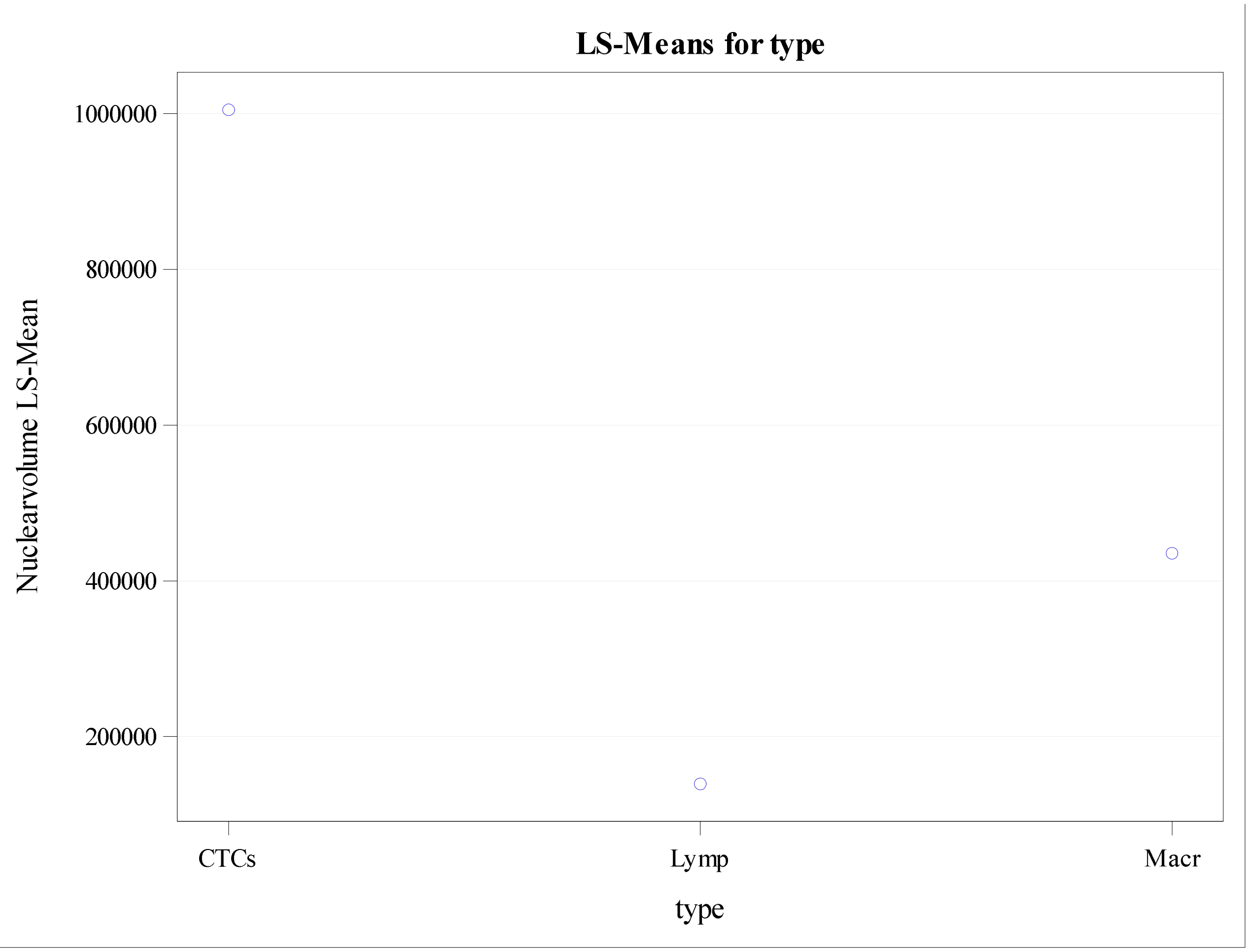

The GLM Procedure
Least Squares Means

pt=17AA0828

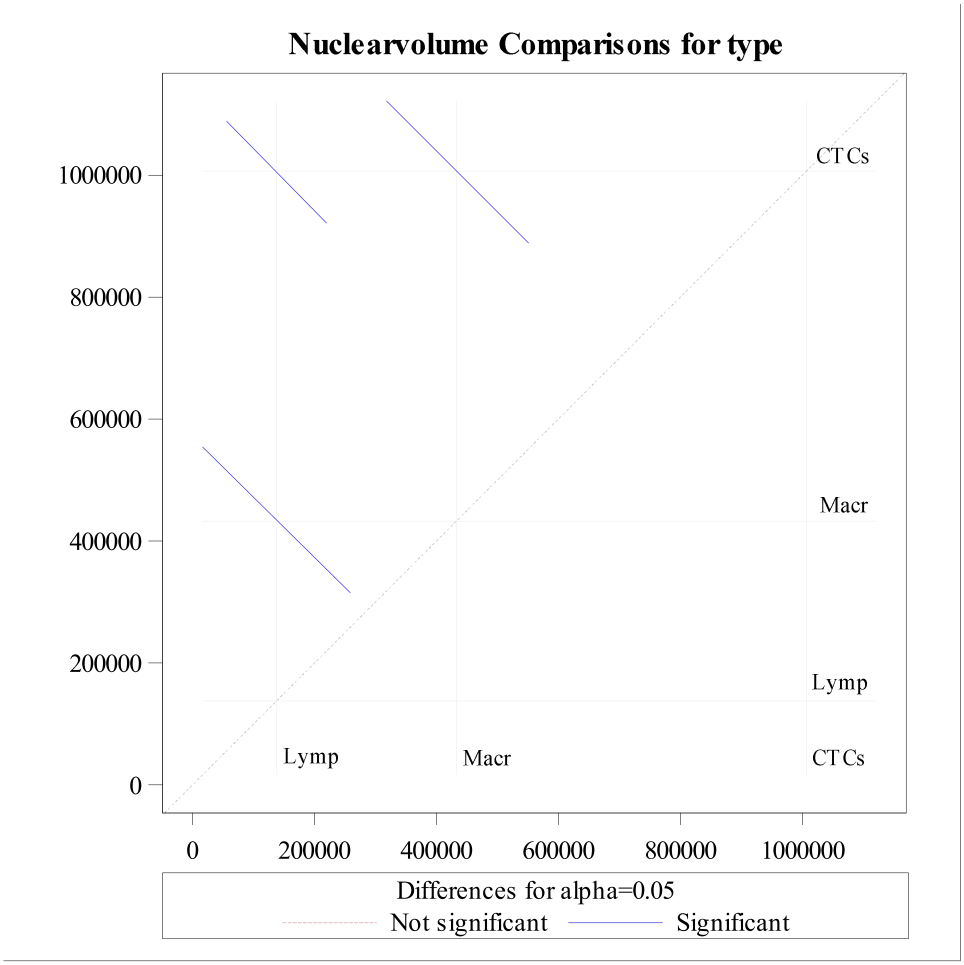

type	telomereperkvol LSMEAN	Standard Error	Pr > |t|	LSMEAN Number
**CTCs**	0.12329236	0.00588857	<.0001	1
**Lymp**	0.15702634	0.00679954	<.0001	2
**Macr**	0.08138265	0.01122907	<.0001	3

Least Squares Means for effect type
Pr > |t| for H0: LSMean(i)=LSMean(j)

Dependent Variable: telomereperkvol

i/j	1	2	3
**1**		0.0003	0.0014
**2**	0.0003		<.0001
**3**	0.0014	<.0001	

The GLM Procedure
Least Squares Means

pt=17AA0828

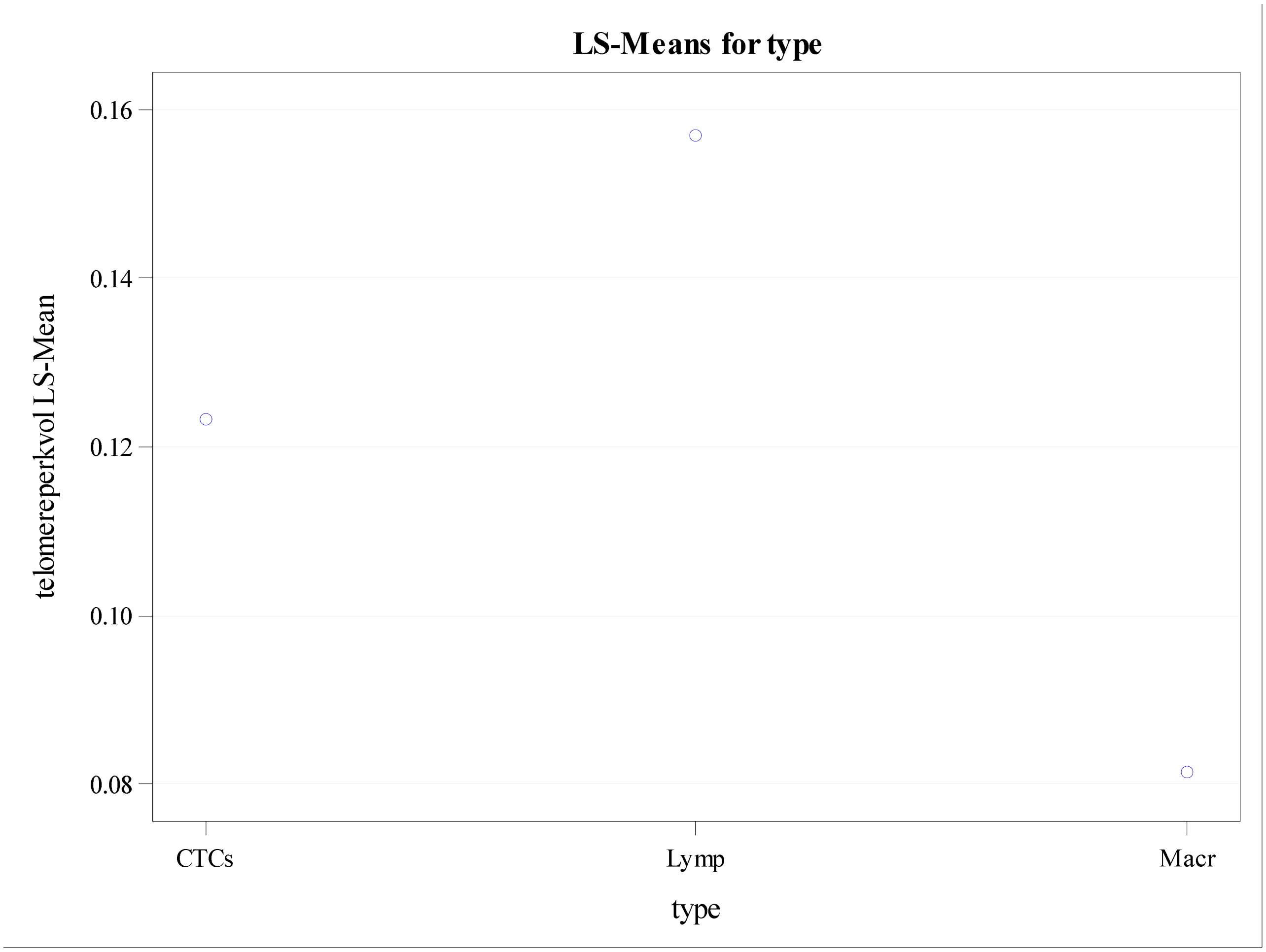

The GLM Procedure
Least Squares Means

pt=17AA0828

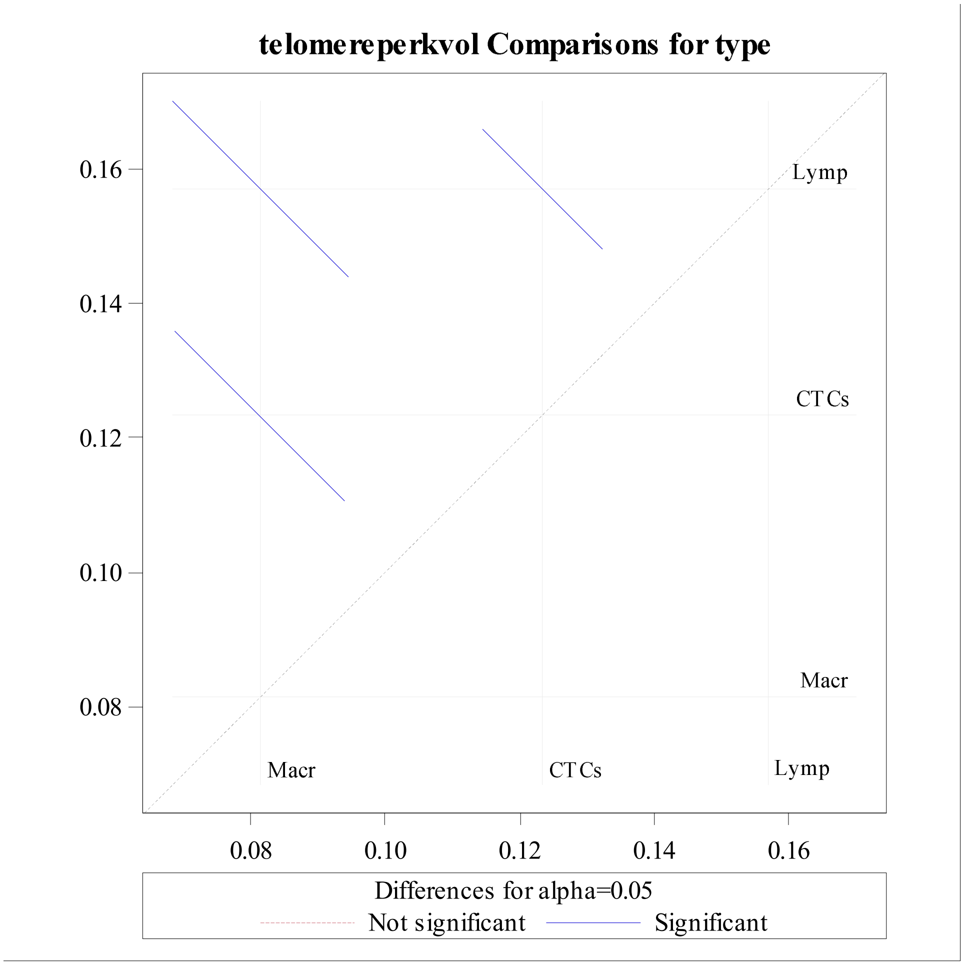

Note: To ensure overall protection level, only probabilities associated with pre-planned comparisons should be used.

The GLM Procedure

pt=17AA0845

Class Level Information		
**Class**	**Levels**	**Values**
**type**	3	CTCs Lymp Macr

**Number of Observations Read**	53
**Number of Observations Used**	53

The GLM Procedure

Dependent Variable: Totalnofsignals
Totalnofsignals

pt=17AA0845

Source	DF	Sum of Squares	Mean Square	F Value	Pr > F
**Model**	2	247113.0529	123556.5264	12.20	<.0001
**Error**	50	506578.6452	10131.5729		
**Corrected Total**	52	753691.6981			

R-Square	Coeff Var	Root MSE	Totalnofsignals Mean
0.327870	145.7185	100.6557	69.07547

Source	DF	Type III SS	Mean Square	F Value	Pr > F
**type**	2	247113.0529	123556.5264	12.20	<.0001

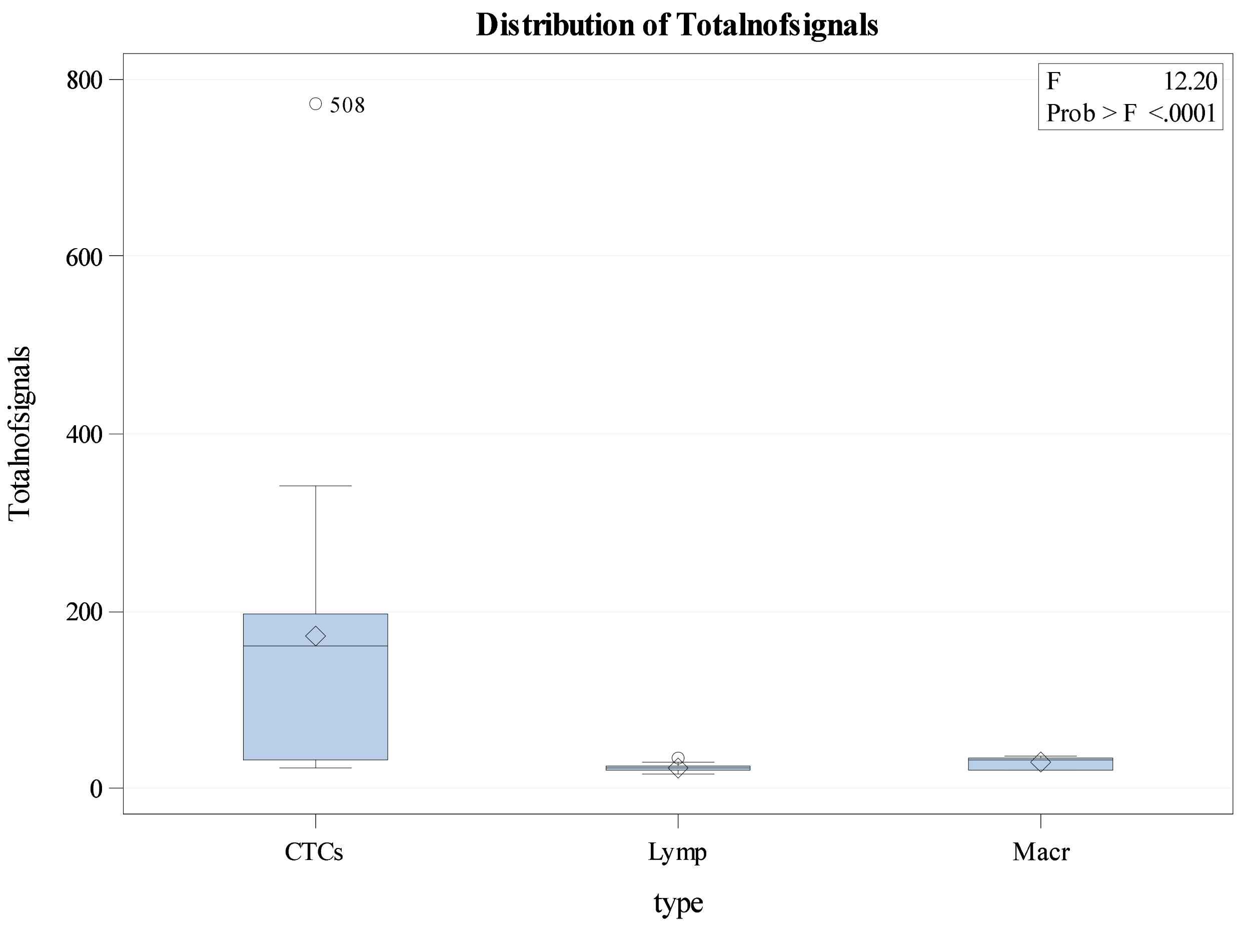

The GLM Procedure

Dependent Variable: Totalnofaggregates Totalnofaggregates

pt=17AA0845

Source	DF	Sum of Squares	Mean Square	F Value	Pr > F
Model	2	3612.23684	1806.11842	11.63	<.0001
Error	50	7762.89524	155.25790		
Corrected Total	52	11375.13208			

R-Square	Coeff Var	Root MSE	Totalnofaggregates Mean
0.317556	167.1882	12.46025	7.452830

Source	DF	Type III SS	Mean Square	F Value	Pr > F
type	2	3612.236837	1806.118419	11.63	<.0001

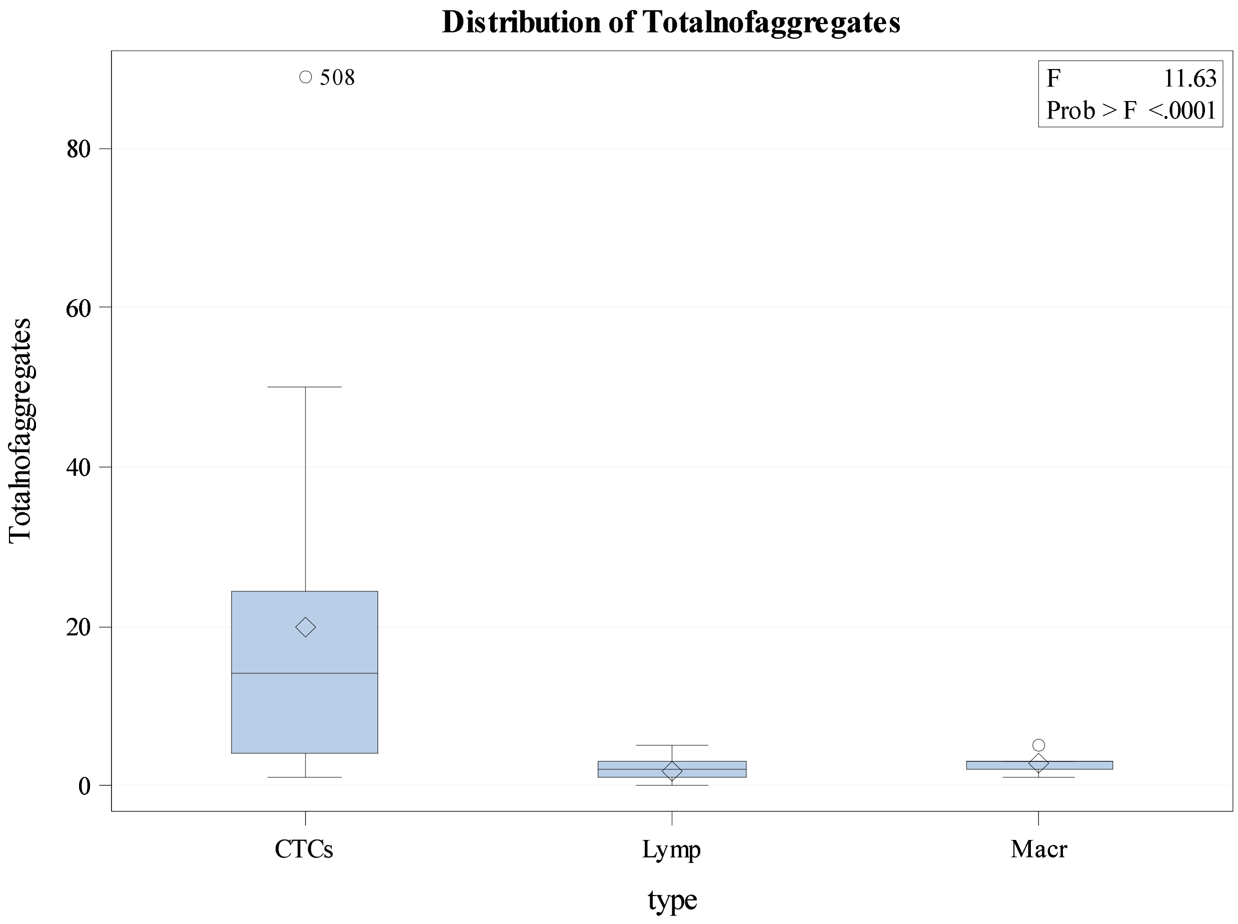

The GLM Procedure

Dependent Variable: acratio
acratio

pt=17AA0845

Source	DF	Sum of Squares	Mean Square	F Value	Pr > F
**Model**	2	133.6529888	66.8264944	44.05	<.0001
**Error**	50	75.8520106	1.5170402		
**Corrected Total**	52	209.5049993			

R-Square	Coeff Var	Root MSE	acratio Mean
0.637947	41.83030	1.231682	2.944473

Source	DF	Type III SS	Mean Square	F Value	Pr > F
**type**	2	133.6529888	66.8264944	44.05	<.0001

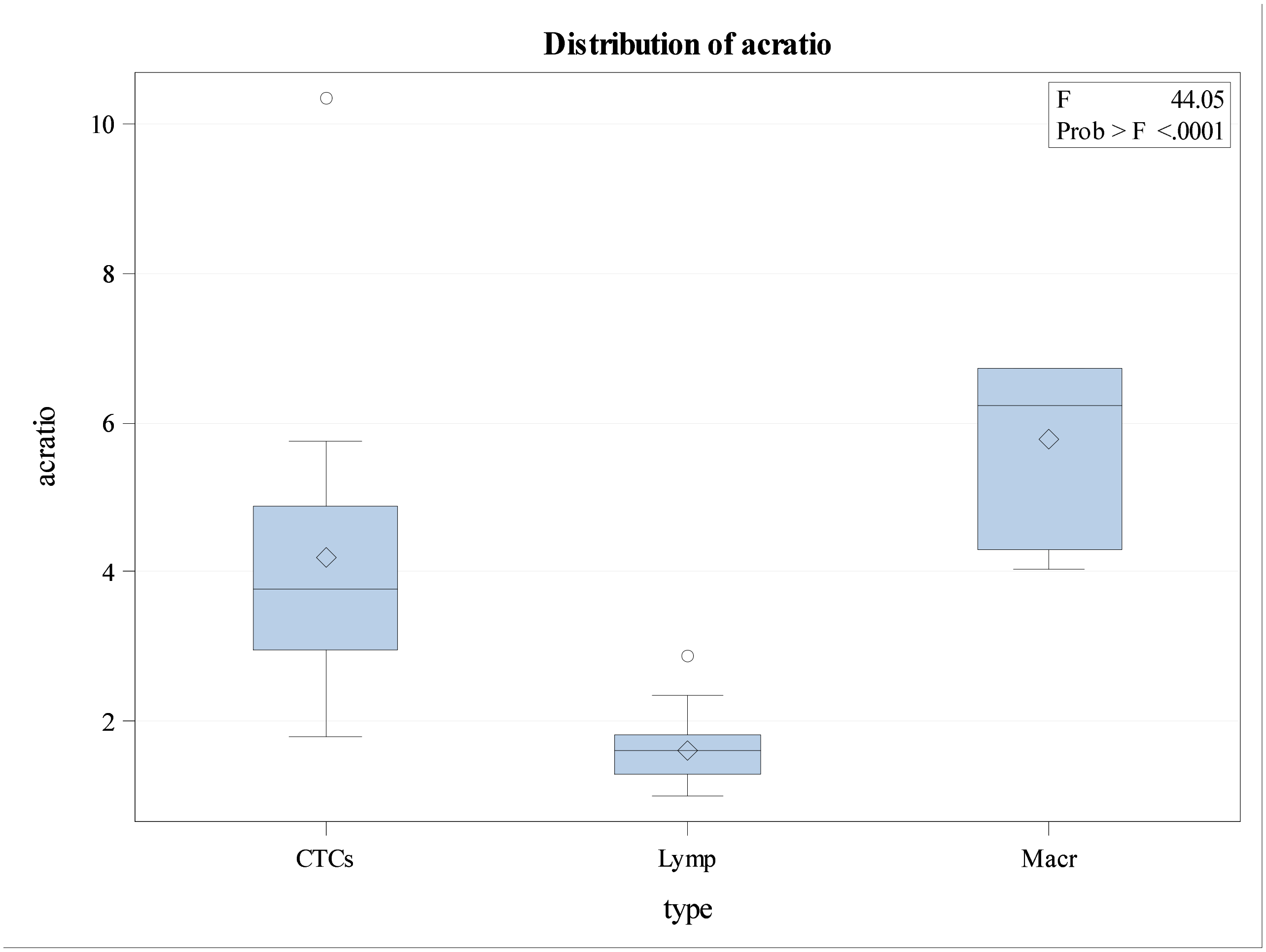

The GLM Procedure

Dependent Variable: AvIntallsignals
AvIntallsignals

pt=17AA0845

Source	DF	Sum of Squares	Mean Square	F Value	Pr > F
**Model**	2	2241078628	1120539314	45.59	<.0001
**Error**	50	1228851983	24577040		
**Corrected Total**	52	3469930611			

R-Square	Coeff Var	Root MSE	AvIntallsignals Mean
0.645857	24.45933	4957.524	20268.44

Source	DF	Type III SS	Mean Square	F Value	Pr > F
**type**	2	2241078628	1120539314	45.59	<.0001

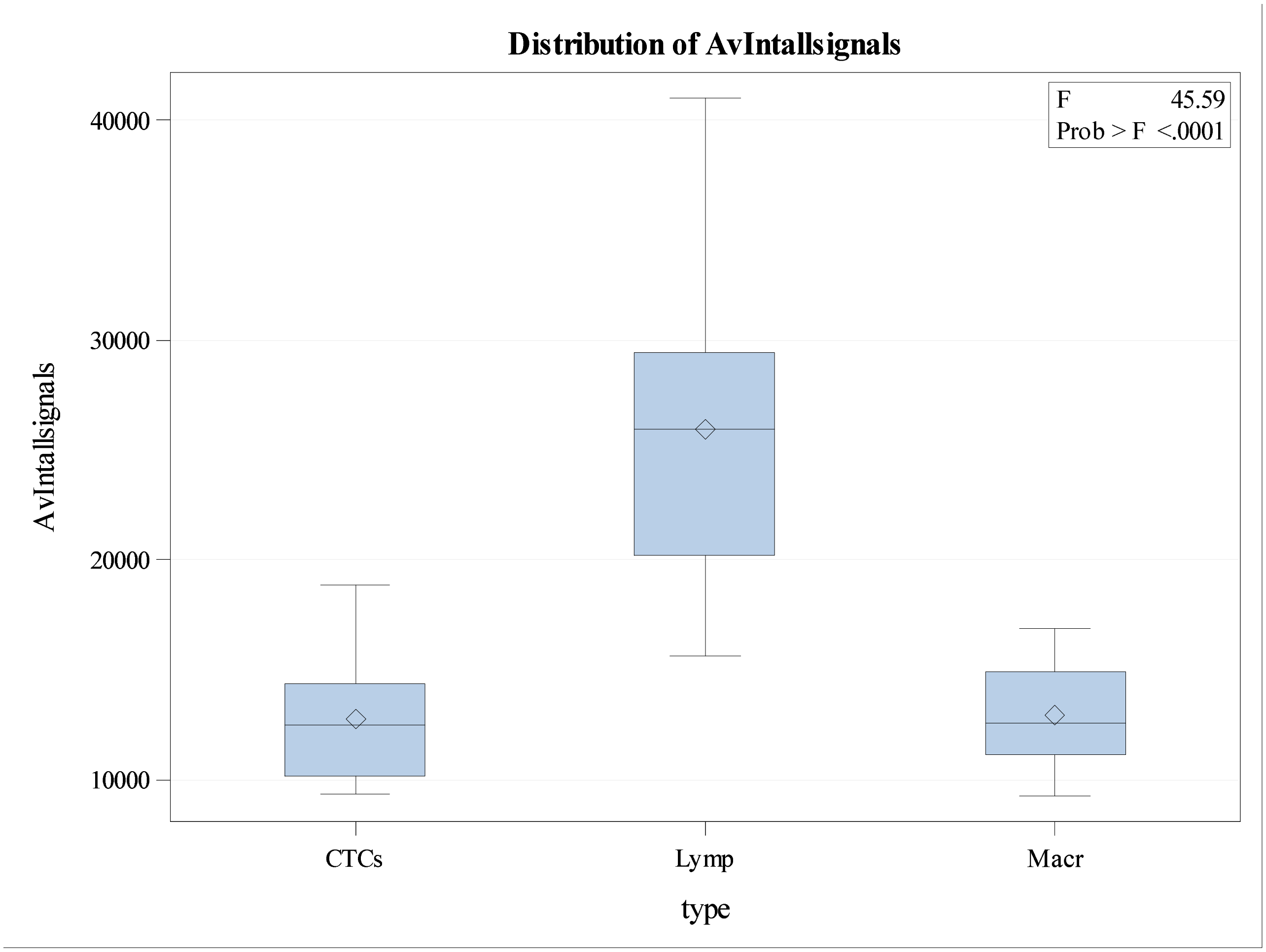

The GLM Procedure

Dependent Variable: Totalintensity
Totalintensity

pt=17AA0845

Source	DF	Sum of Squares	Mean Square	F Value	Pr > F
**Model**	2	2.2718622E13	1.1359311E13	9.24	0.0004
**Error**	50	6.1476912E13	1.2295382E12		
**Corrected Total**	52	8.4195534E13			

R-Square	Coeff Var	Root MSE	Totalintensity Mean
0.269832	112.9075	1108845	982083.4

Source	DF	Type III SS	Mean Square	F Value	Pr > F
**type**	2	2.2718622E13	1.1359311E13	9.24	0.0004

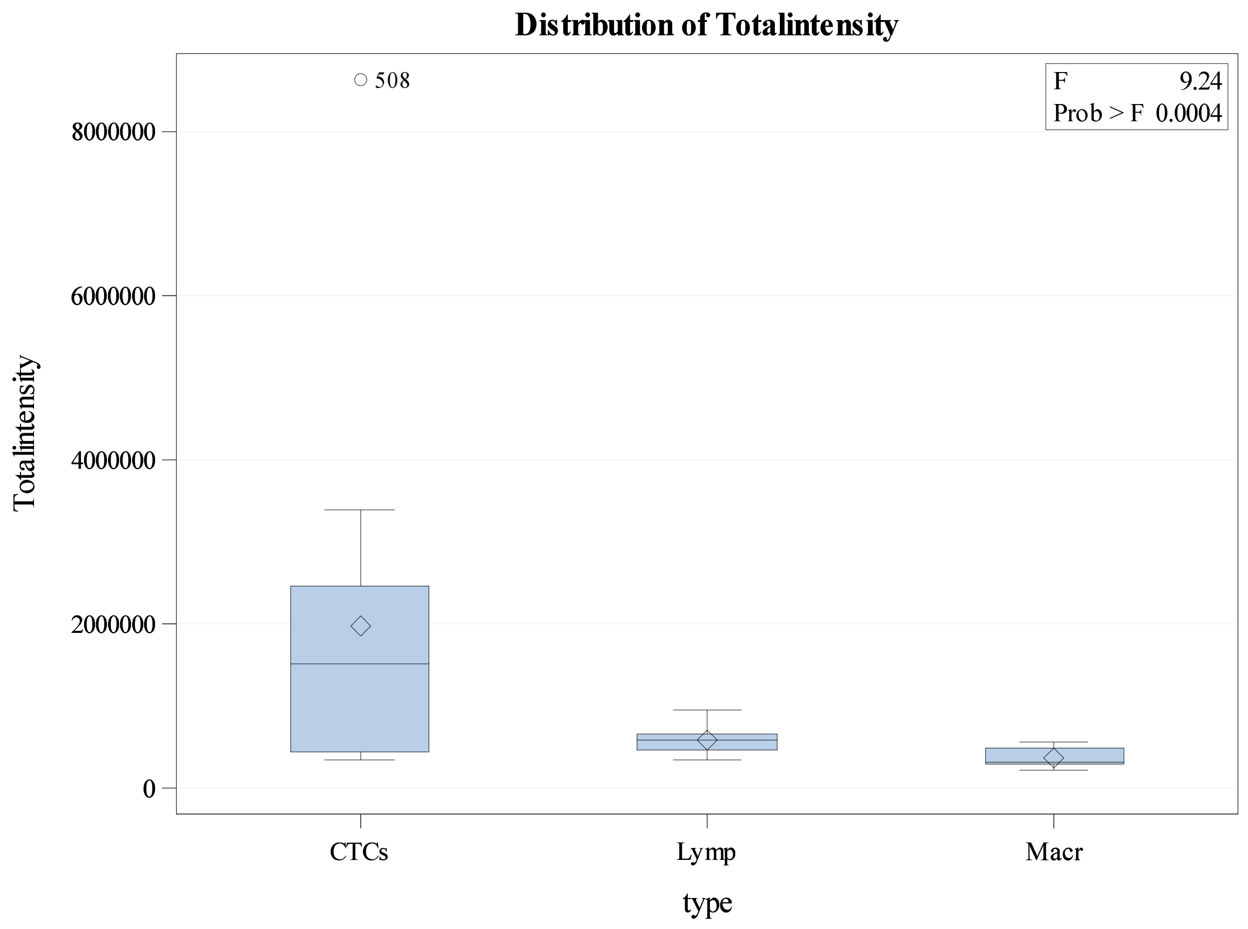

The GLM Procedure

Dependent Variable: Nuclearvolume Nuclearvolume

pt=17AA0845

Source	DF	Sum of Squares	Mean Square	F Value	Pr > F
**Model**	2	1.4815672E13	7.4078358E12	21.07	<.0001
**Error**	50	1.7579489E13	351589777355		
**Corrected Total**	52	3.2395161E13			

R-Square	Coeff Var	Root MSE	Nuclearvolume Mean
0.457342	108.0104	592950.1	548975.0

Source	DF	Type III SS	Mean Square	F Value	Pr > F
**type**	2	1.4815672E13	7.4078358E12	21.07	<.0001

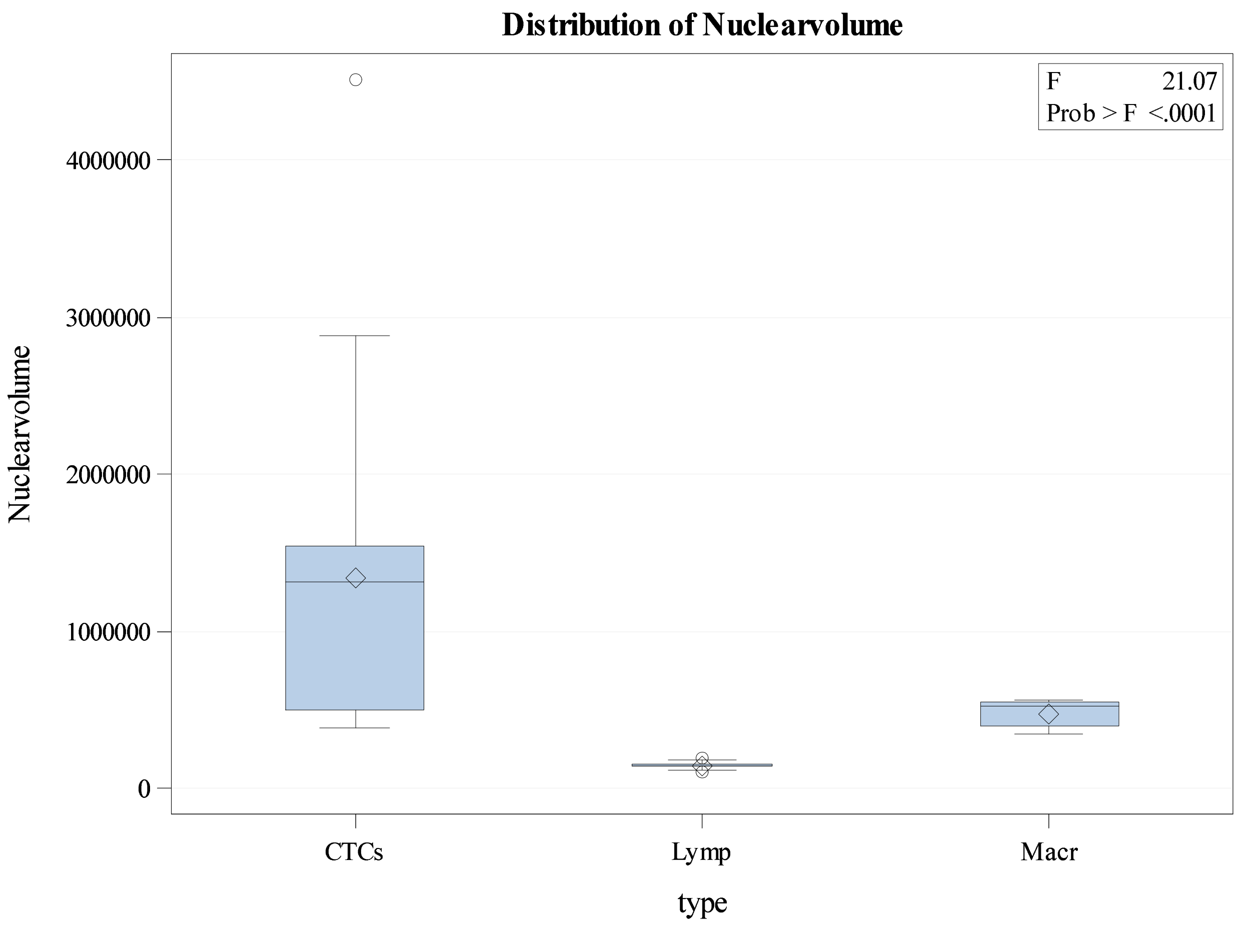

The GLM Procedure

Dependent Variable: telomereperkvol

pt=17AA0845

Source	DF	Sum of Squares	Mean Square	F Value	Pr > F
Model	2	0.06441565	0.03220782	29.25	<.0001
Error	50	0.05506254	0.00110125		
Corrected Total	52	0.11947819			

R-Square	Coeff Var	Root MSE	telomereperkvol Mean
0.539141	24.98575	0.033185	0.132816

Source	DF	Type III SS	Mean Square	F Value	Pr > F
type	2	0.06441565	0.03220782	29.25	<.0001

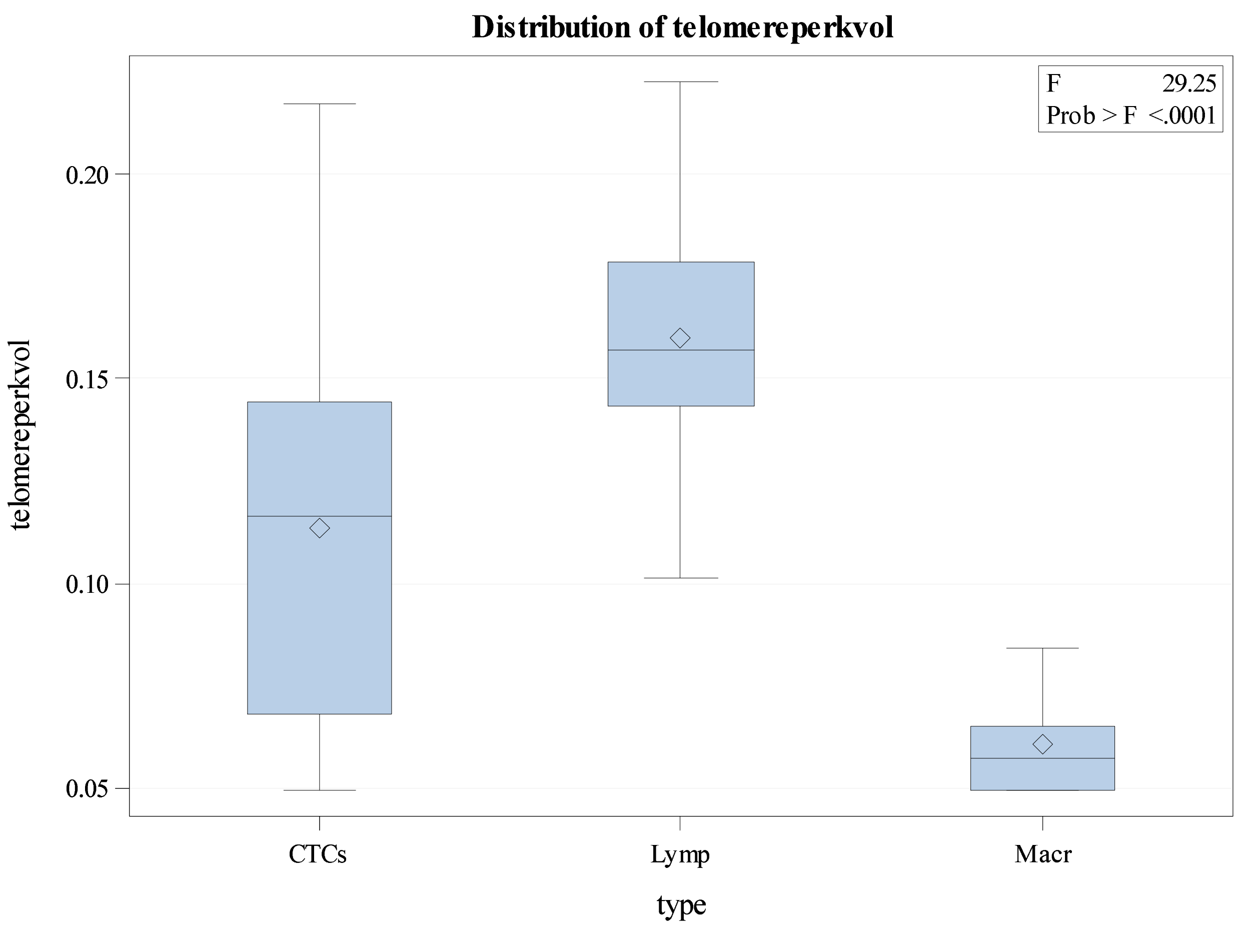

The GLM Procedure
Least Squares Means

pt=17AA0845

type	Totalnofsignals LSMEAN	Standard Error	Pr > |t|	LSMEAN Number
**CTCs**	172.875000	25.163929	<.0001	1
**Lymp**	23.133333	18.377135	0.2139	2
**Macr**	28.714286	38.044284	0.4539	3

Least Squares Means for effect type
Pr > |t| for H0: LSMean(i)=LSMean(j)

Dependent Variable: Totalnofsignals

i/j	1	2	3
**1**		<.0001	0.0027
**2**	<.0001		0.8954
**3**	0.0027	0.8954	

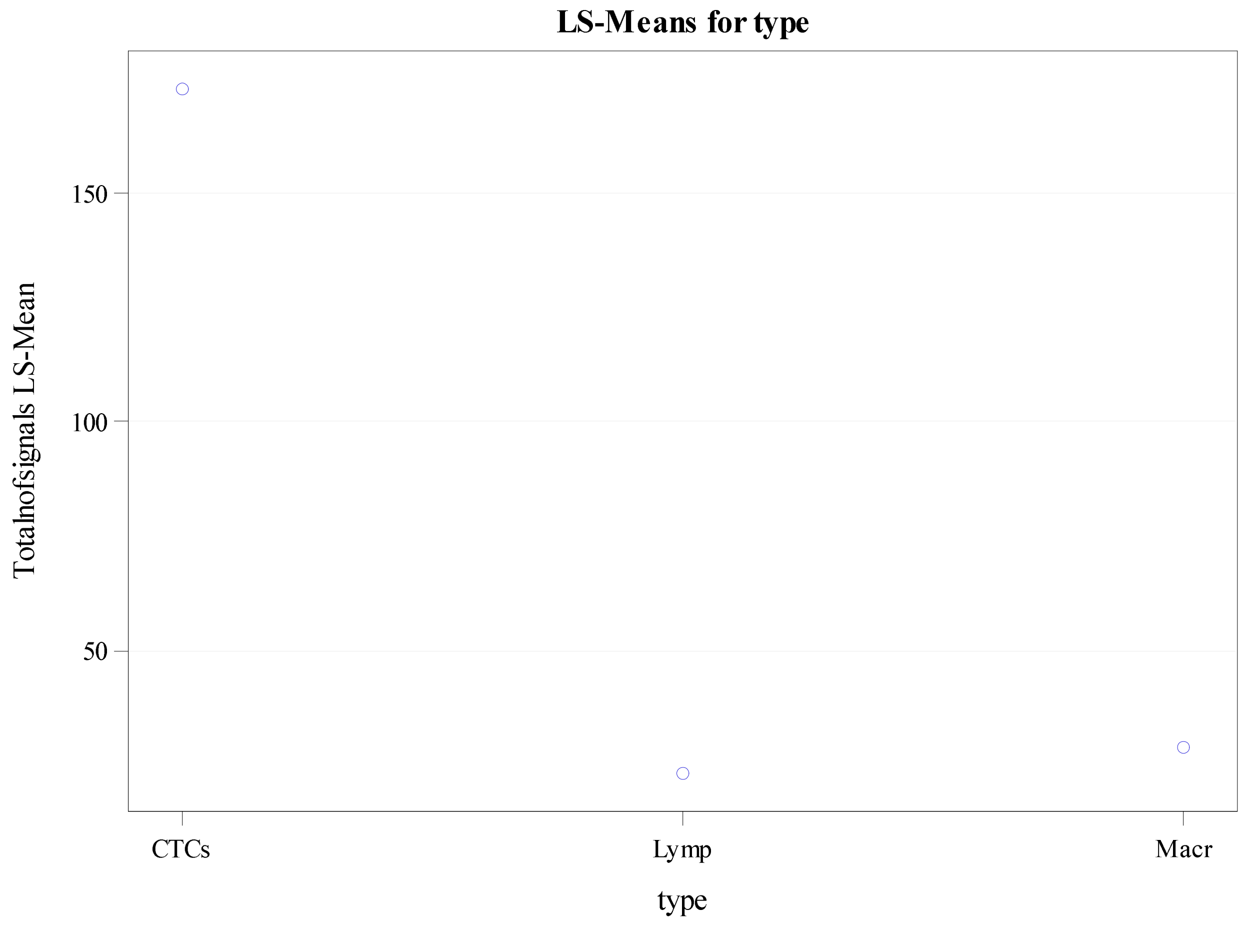

The GLM Procedure
Least Squares Means

pt=17AA0845

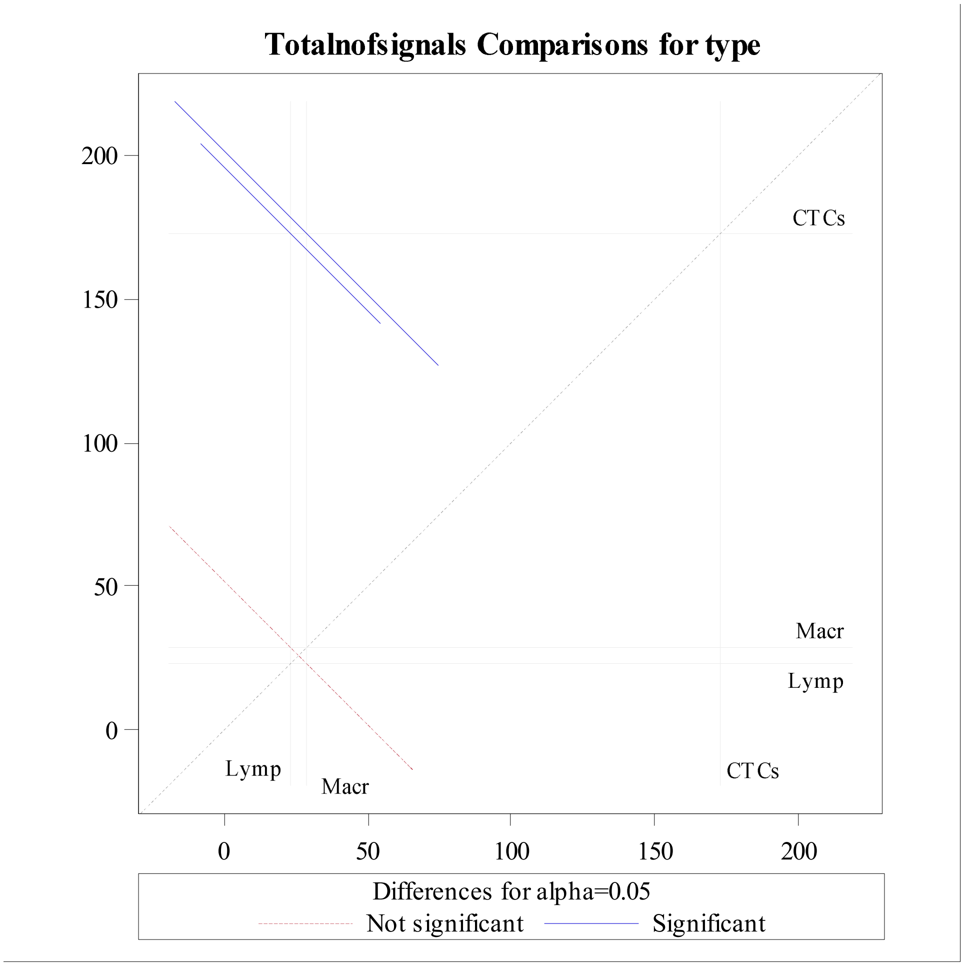

type	Totalnofaggregates LSMEAN	Standard Error	Pr > |t|	LSMEAN Number
**CTCs**	20.0000000	3.1150632	<.0001	1
**Lymp**	1.8666667	2.2749205	0.4158	2
**Macr**	2.7142857	4.7095330	0.5670	3

The GLM Procedure
Least Squares Means

pt=17AA0845

Least Squares Means for effect type Pr > |t| for H0: LSMean(i)=LSMean(j) Dependent Variable: Totalnofaggregates			
**i/j**	**1**	**2**	**3**
**1**		<.0001	0.0035
**2**	<.0001		0.8719
**3**	0.0035	0.8719	

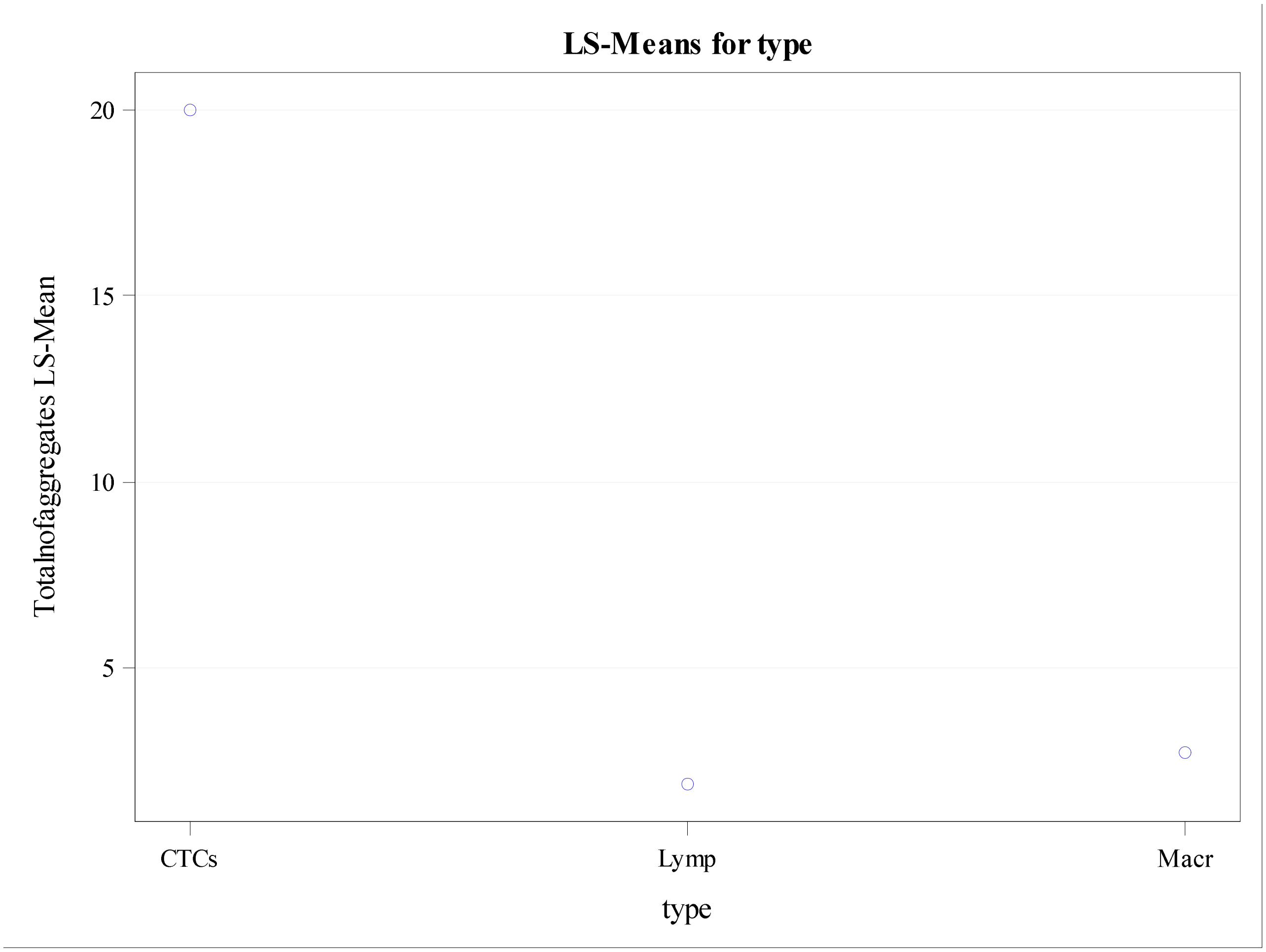

The GLM Procedure
Least Squares Means

pt=17AA0845

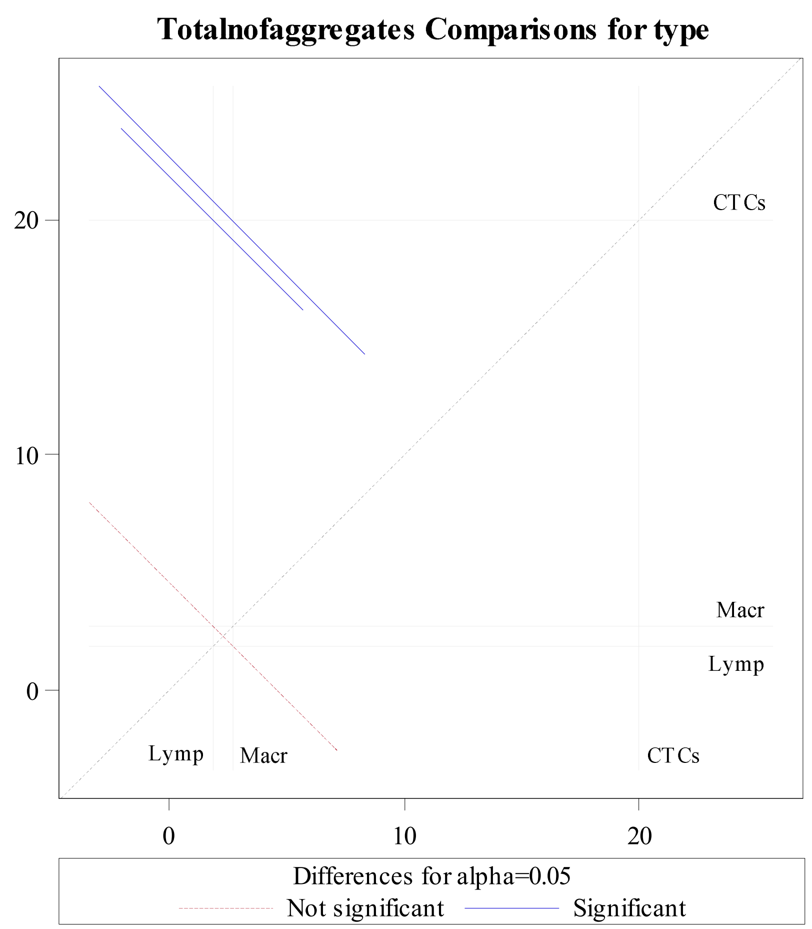

type	acratio LSMEAN	Standard Error	Pr > |t|	LSMEAN Number
**CTCs**	4.19030338	0.30792047	<.0001	1
**Lymp**	1.61927097	0.22487331	<.0001	2
**Macr**	5.77629933	0.46553199	<.0001	3

Least Squares Means for effect type
Pr > |t| for H0: LSMean(i)=LSMean(j)

Dependent Variable: acratio

i/j	1	2	3
**1**		<.0001	0.0065
**2**	<.0001		<.0001
**3**	0.0065	<.0001	

The GLM Procedure
Least Squares Means

pt=17AA0845

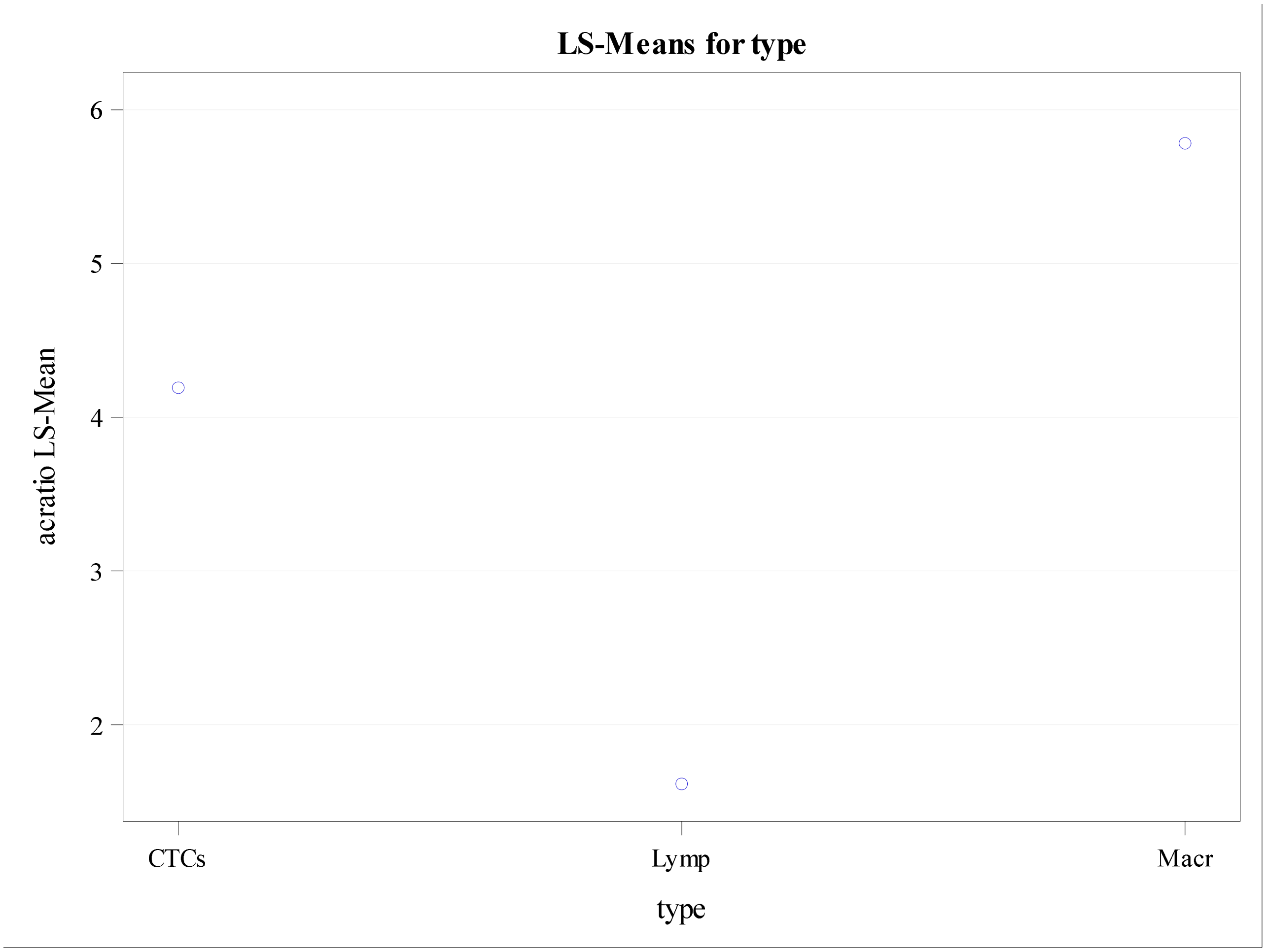

The GLM Procedure
Least Squares Means

pt=17AA0845

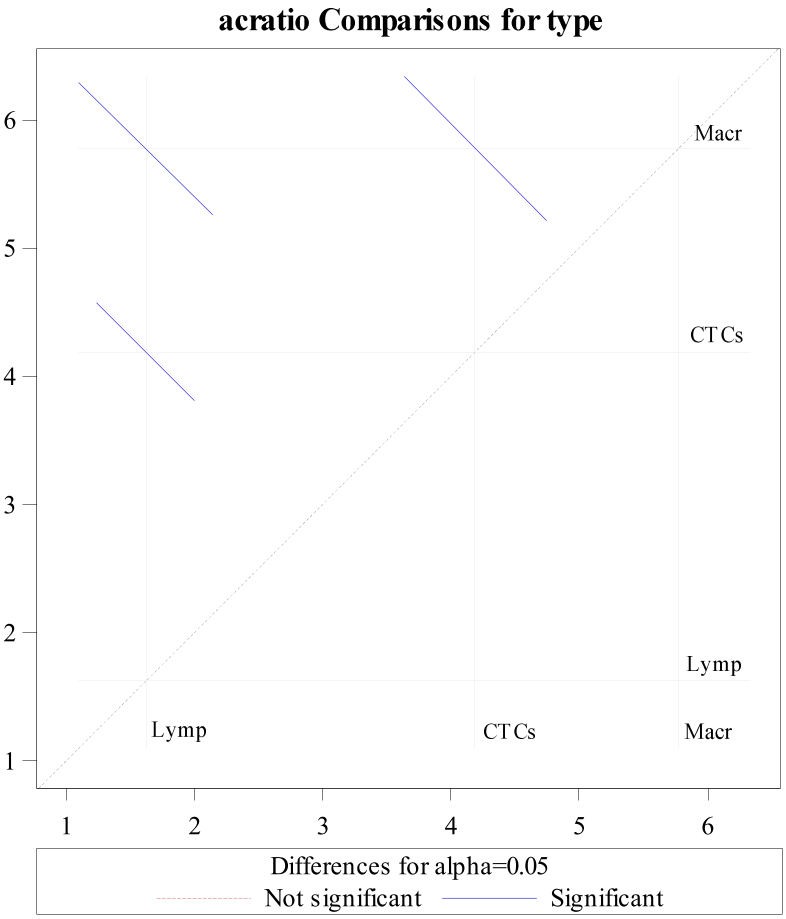

type	AvIntallsignals LSMEAN	Standard Error	Pr > |t|	LSMEAN Number
**CTCs**	12792.7757	1239.3809	<.0001	1
**Lymp**	25961.9650	905.1158	<.0001	2
**Macr**	12954.8233	1873.7678	<.0001	3

Least Squares Means for effect type
Pr > |t| for H0: LSMean(i)=LSMean(j)

Dependent Variable: AvIntallsignals

i/j	1	2	3
**1**		<.0001	0.9428
**2**	<.0001		<.0001
**3**	0.9428	<.0001	

The GLM Procedure
Least Squares Means

pt=17AA0845

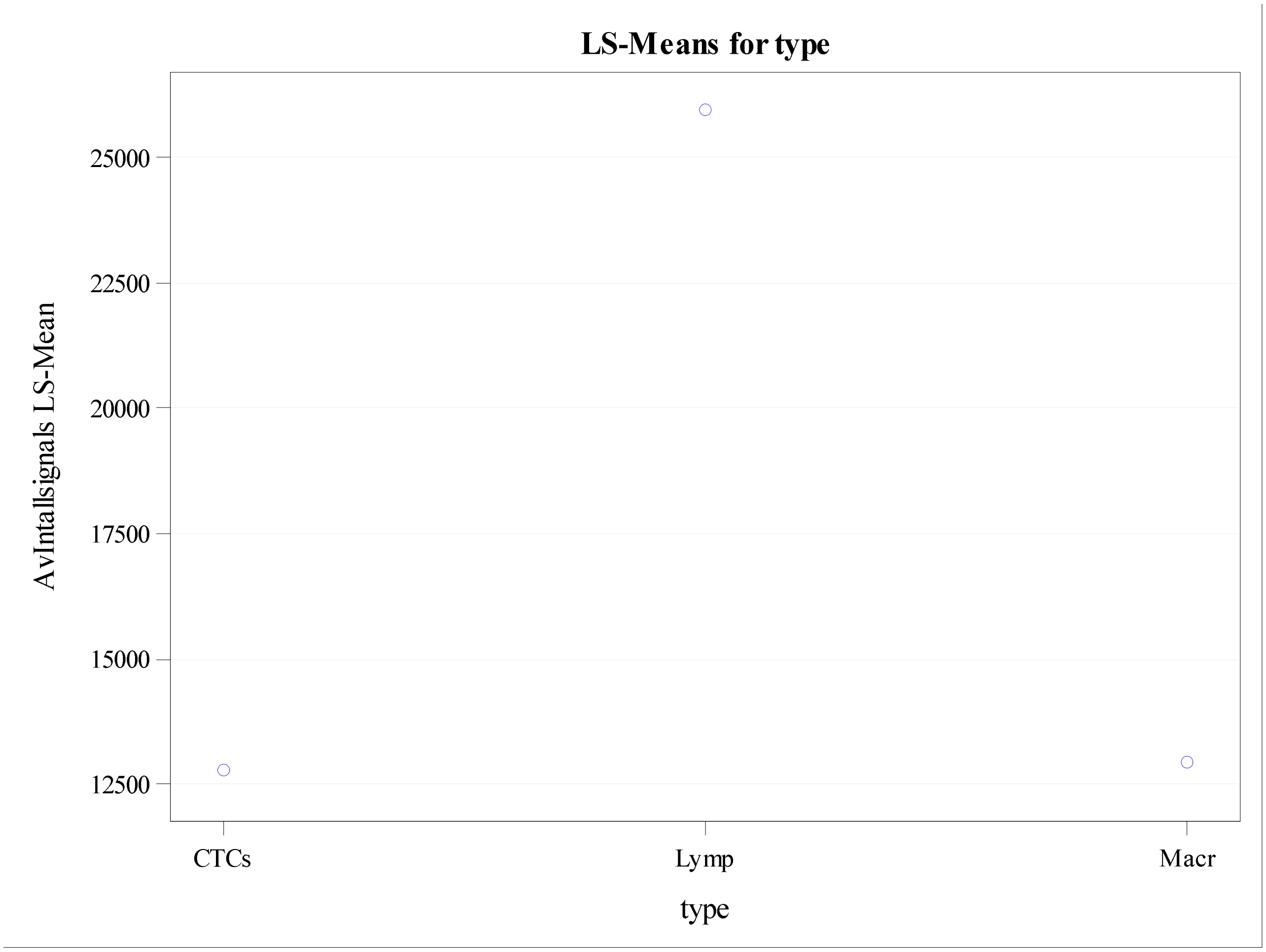

The GLM Procedure
Least Squares Means

pt=17AA0845

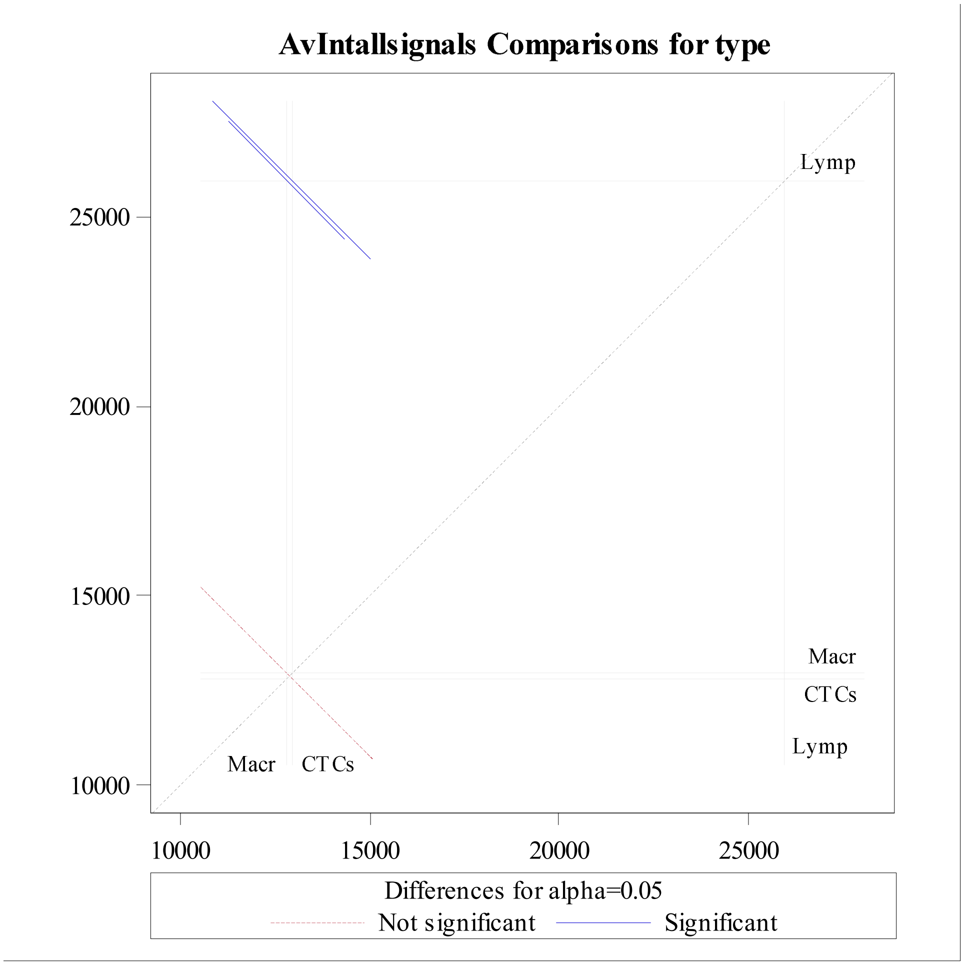

type	Totalintensity LSMEAN	Standard Error	Pr > |t|	LSMEAN Number
**CTCs**	1971419.69	277211.36	<.0001	1
**Lymp**	596723.40	202446.56	0.0049	2
**Macr**	372285.86	419104.19	0.3786	3

Least Squares Means for effect type
Pr > |t| for H0: LSMean(i)=LSMean(j)

Dependent Variable: Totalintensity

i/j	1	2	3
**1**		0.0002	0.0025
**2**	0.0002		0.6318
**3**	0.0025	0.6318	

The GLM Procedure
Least Squares Means

pt=17AA0845

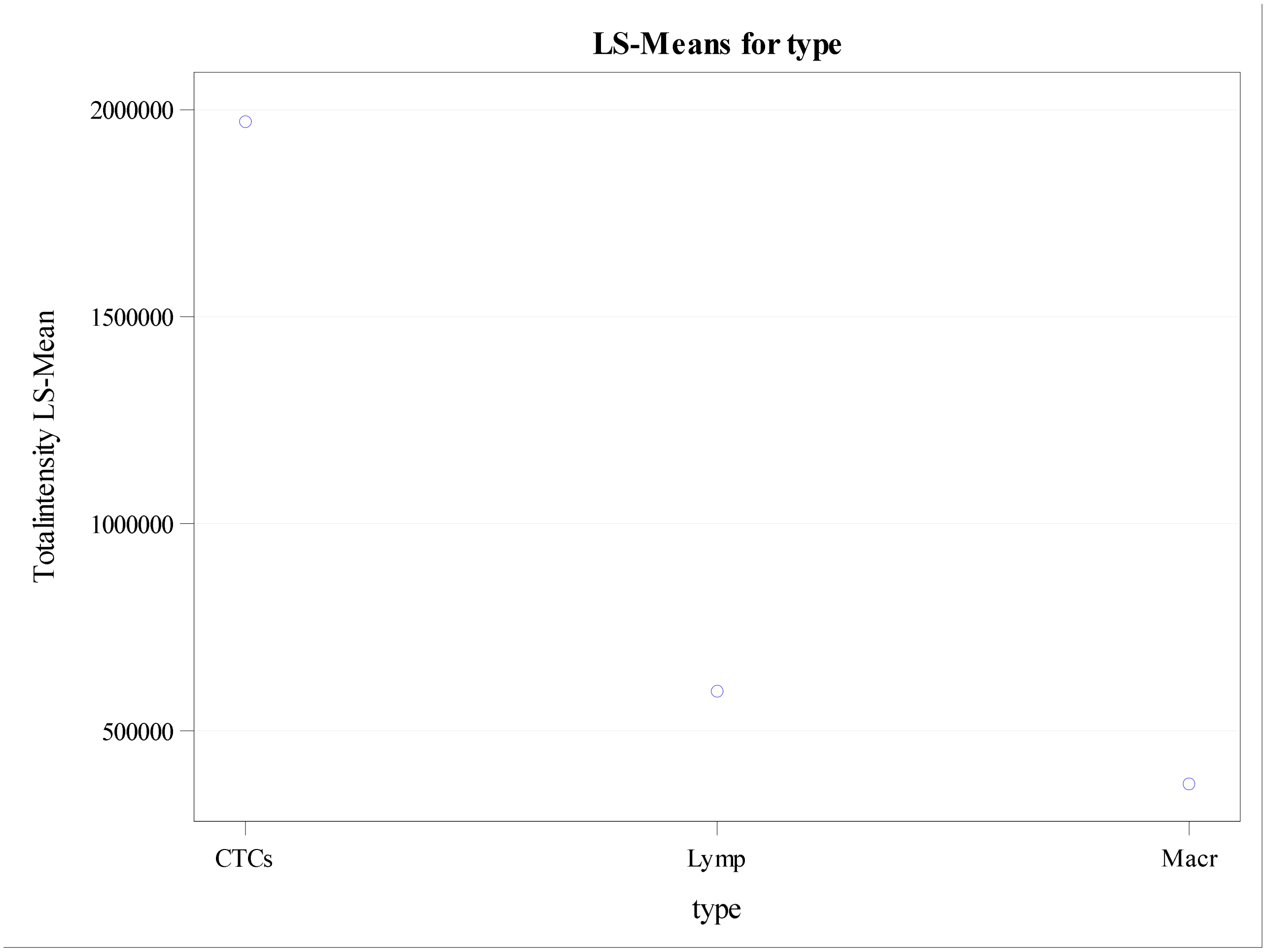

The GLM Procedure
Least Squares Means

pt=17AA0845

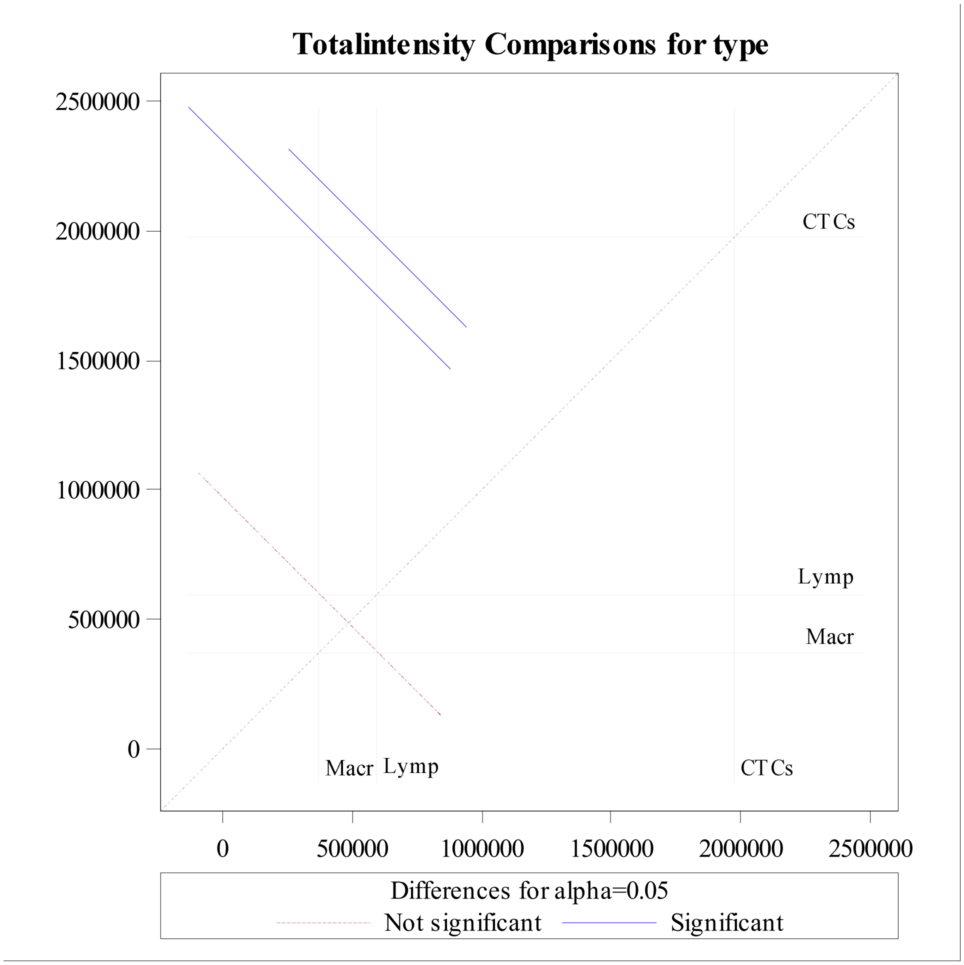

type	Nuclearvolume LSMEAN	Standard Error	Pr > |t|	LSMEAN Number
**CTCs**	1336007.38	148237.52	<.0001	1
**Lymp**	146145.93	108257.38	0.1831	2
**Macr**	476454.43	224114.06	0.0385	3

Least Squares Means for effect type<br>Pr > |t| for H0: LSMean(i)=LSMean(j)<br><br>Dependent Variable: Nuclearvolume			
**i/j**	**1**	**2**	**3**
**1**		<.0001	0.0024
**2**	<.0001		0.1905
**3**	0.0024	0.1905	

The GLM Procedure
Least Squares Means

pt=17AA0845

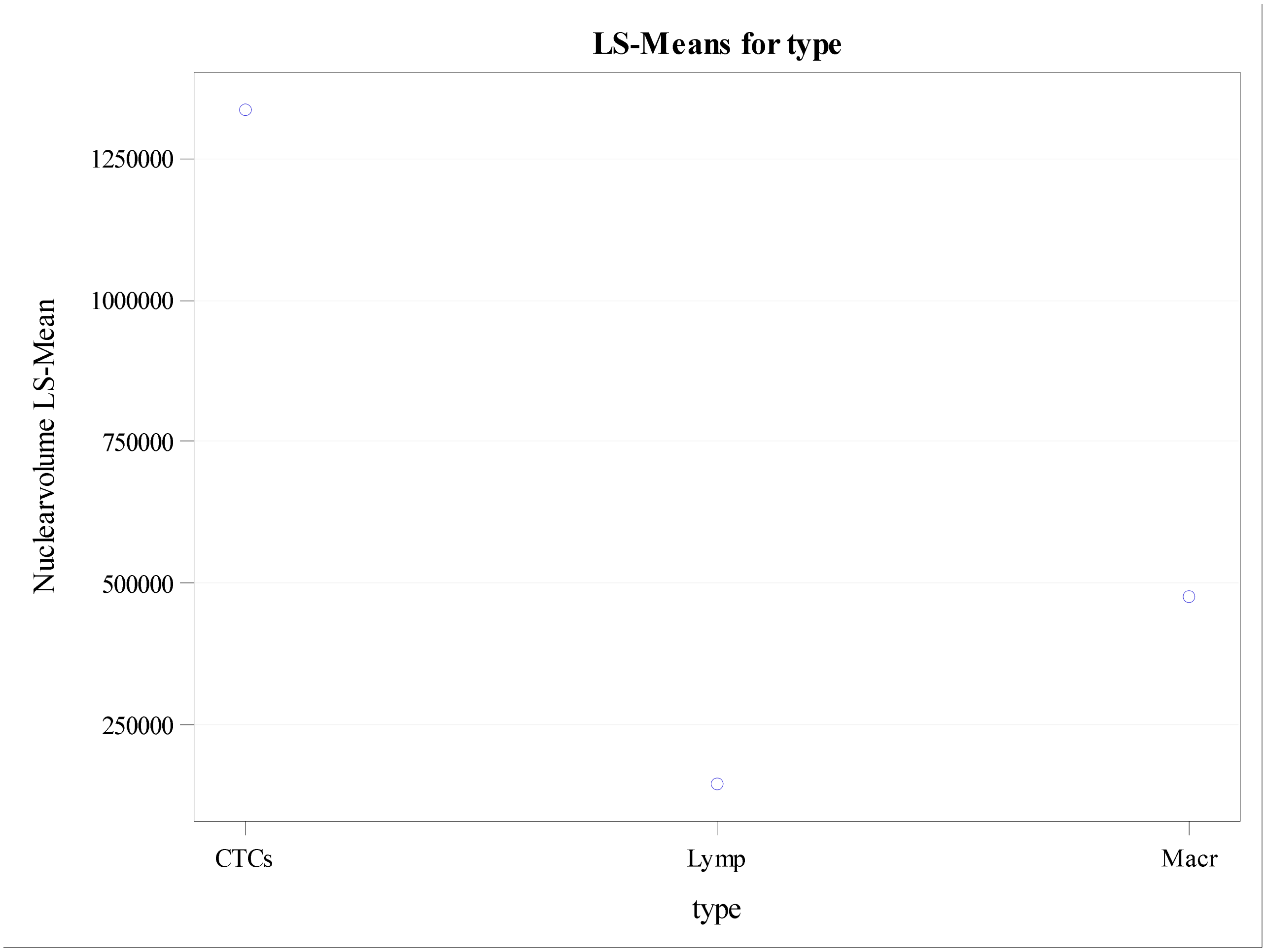

The GLM Procedure
Least Squares Means

pt=17AA0845

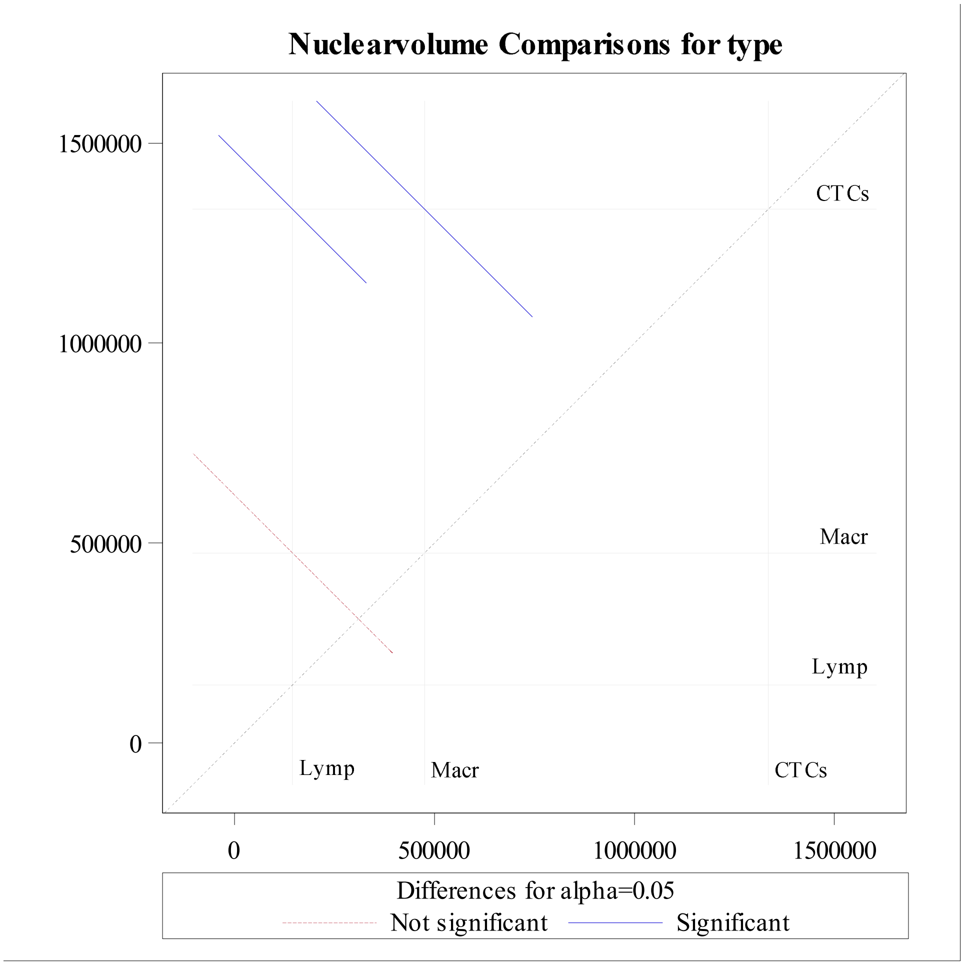

type	telomereperkvol LSMEAN	Standard Error	Pr > |t|	LSMEAN Number
**CTCs**	0.11361736	0.00829627	<.0001	1
**Lymp**	0.15990522	0.00605874	<.0001	2
**Macr**	0.06060256	0.01254279	<.0001	3

Least Squares Means for effect type<br>Pr > |t| for H0: LSMean(i)=LSMean(j)<br><br>Dependent Variable: telomereperkvol			
**i/j**	**1**	**2**	**3**
**1**		<.0001	0.0009
**2**	<.0001		<.0001
**3**	0.0009	<.0001	

The GLM Procedure
Least Squares Means

pt=17AA0845

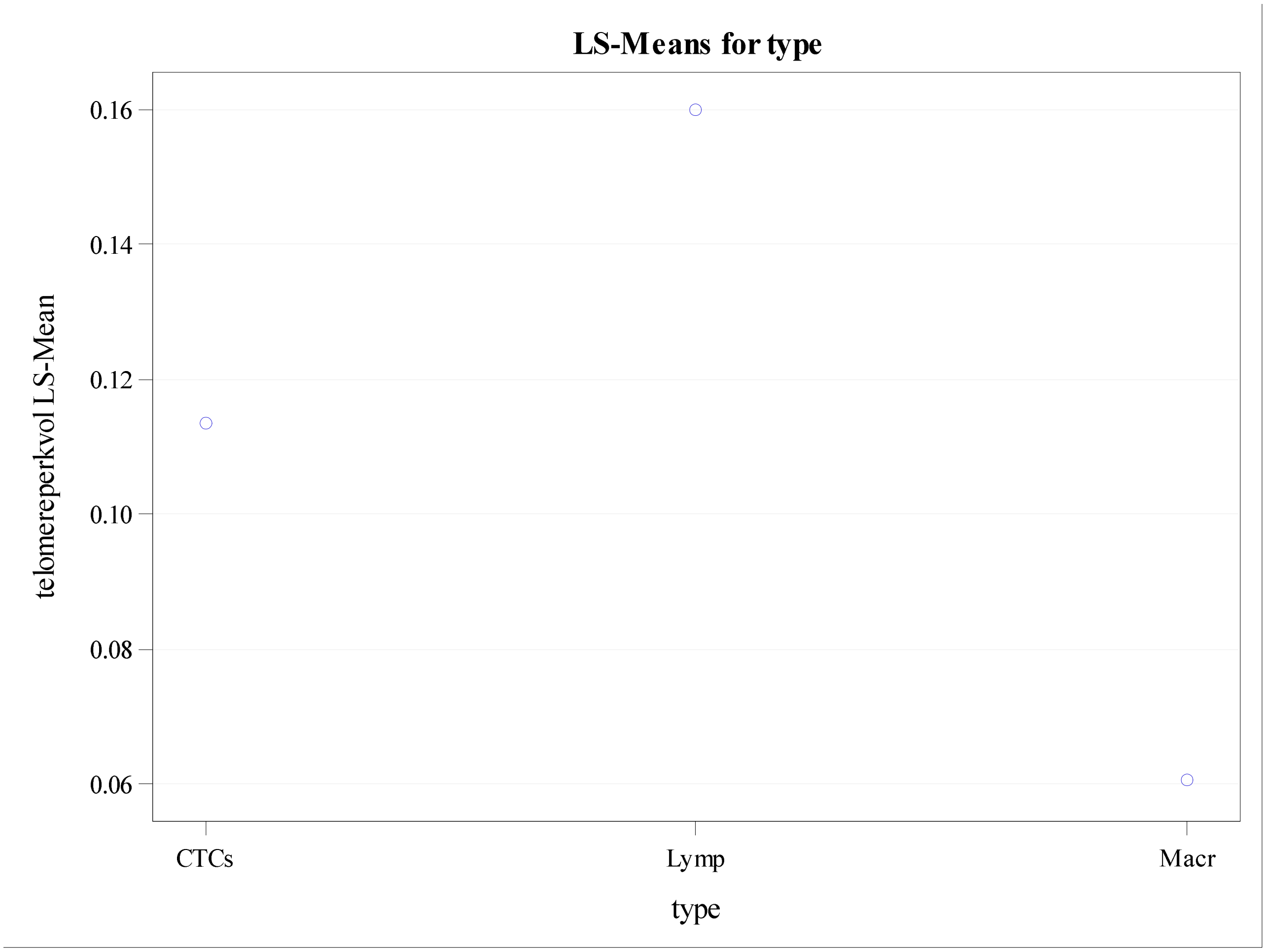

The GLM Procedure
Least Squares Means

pt=17AA0845

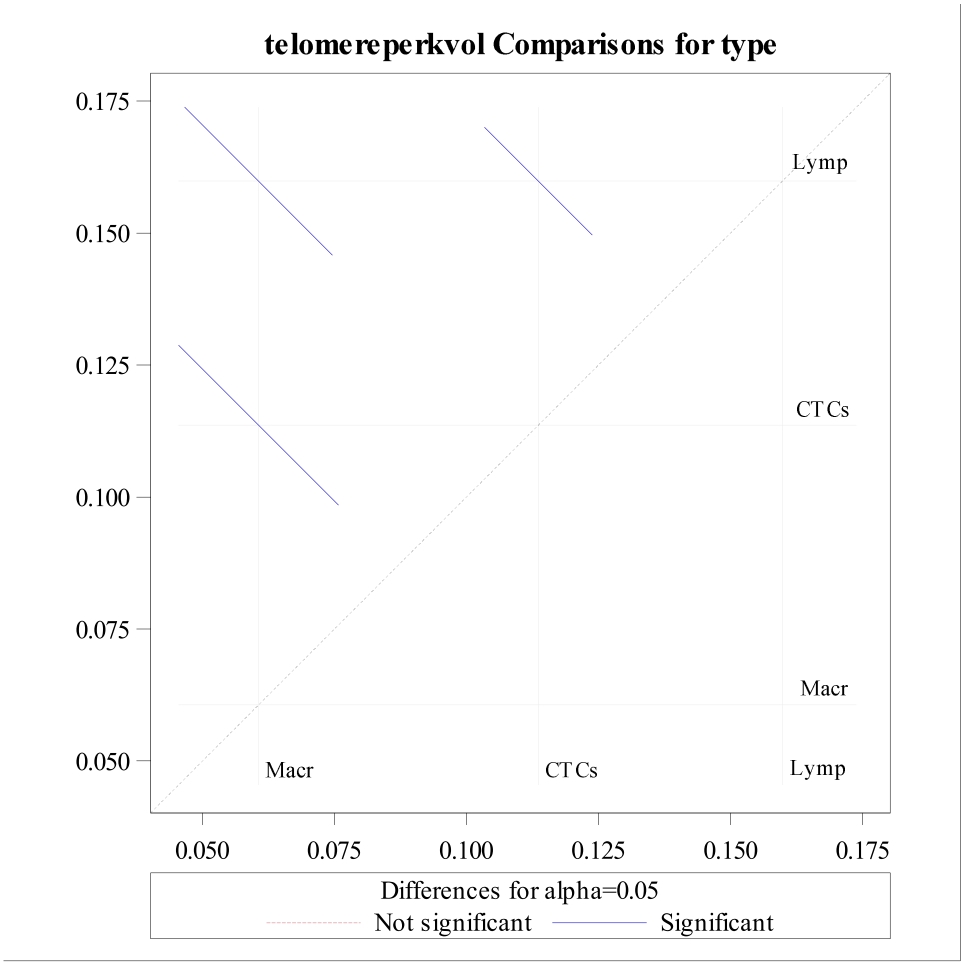

Note: To ensure overall protection level, only probabilities associated with pre-planned comparisons should be used.

The GLM Procedure

pt=17AA1313

Class Level Information		
**Class**	**Levels**	**Values**
**type**	3	CTCs Lymp Macr

**Number of Observations Read**	105
**Number of Observations Used**	105

The GLM Procedure

Dependent Variable: Totalnofsignals Totalnofsignals

pt=17AA1313

Source	DF	Sum of Squares	Mean Square	F Value	Pr > F
**Model**	2	76139.5482	38069.7741	44.17	<.0001
**Error**	102	87908.1090	861.8442		
**Corrected Total**	104	164047.6571			

R-Square	Coeff Var	Root MSE	Totalnofsignals Mean
0.464131	57.17871	29.35718	51.34286

Source	DF	Type III SS	Mean Square	F Value	Pr > F
**type**	2	76139.54817	38069.77408	44.17	<.0001

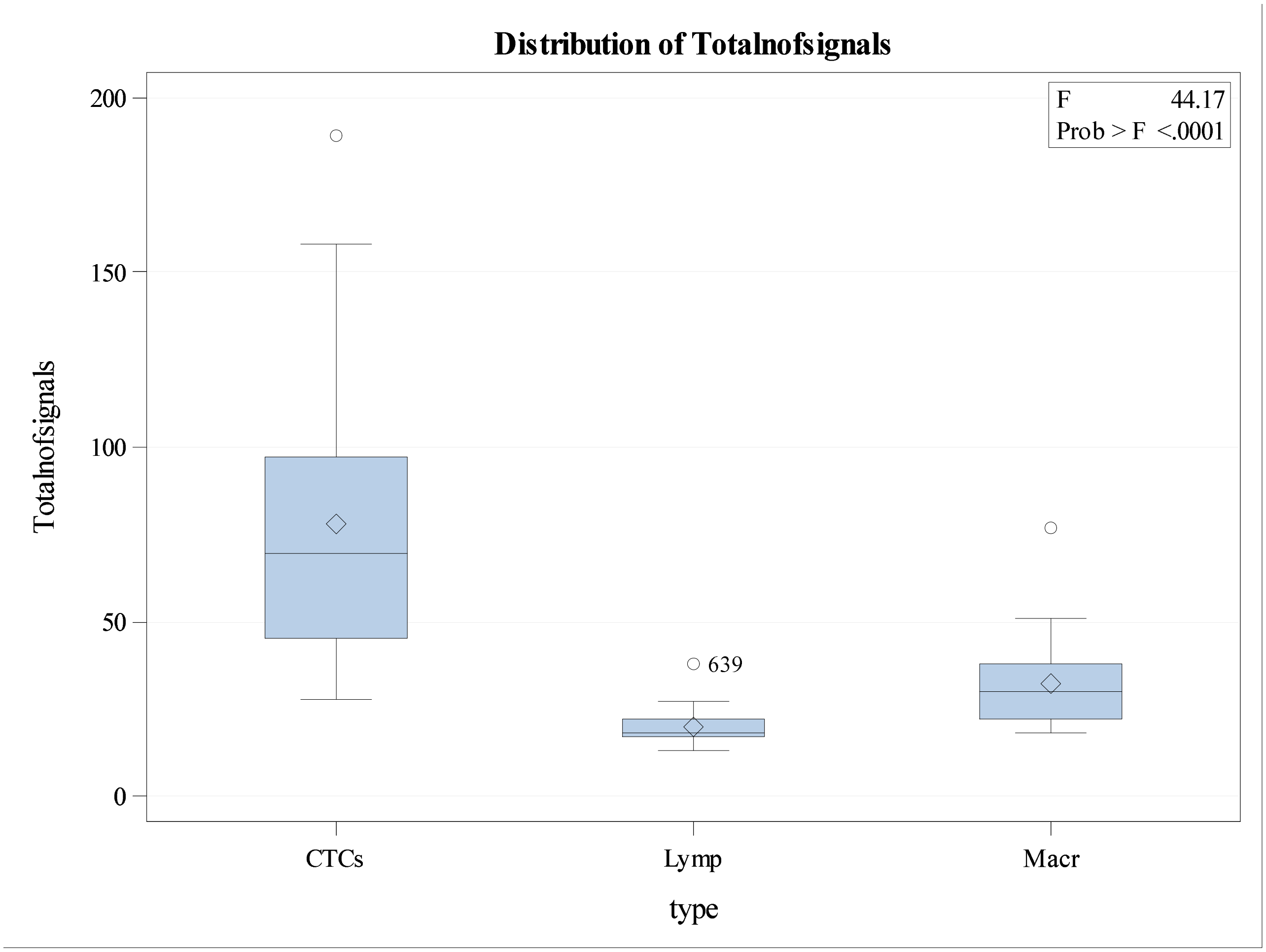

The GLM Procedure

Dependent Variable: Totalnofaggregates Totalnofaggregates

pt=17AA1313

Source	DF	Sum of Squares	Mean Square	F Value	Pr > F
**Model**	2	868.800964	434.400482	21.56	<.0001
**Error**	102	2055.446656	20.151438		
**Corrected Total**	104	2924.247619			

R-Square	Coeff Var	Root MSE	Totalnofaggregates Mean
0.297102	81.54822	4.489035	5.504762

Source	DF	Type III SS	Mean Square	F Value	Pr > F
**type**	2	868.8009635	434.4004818	21.56	<.0001

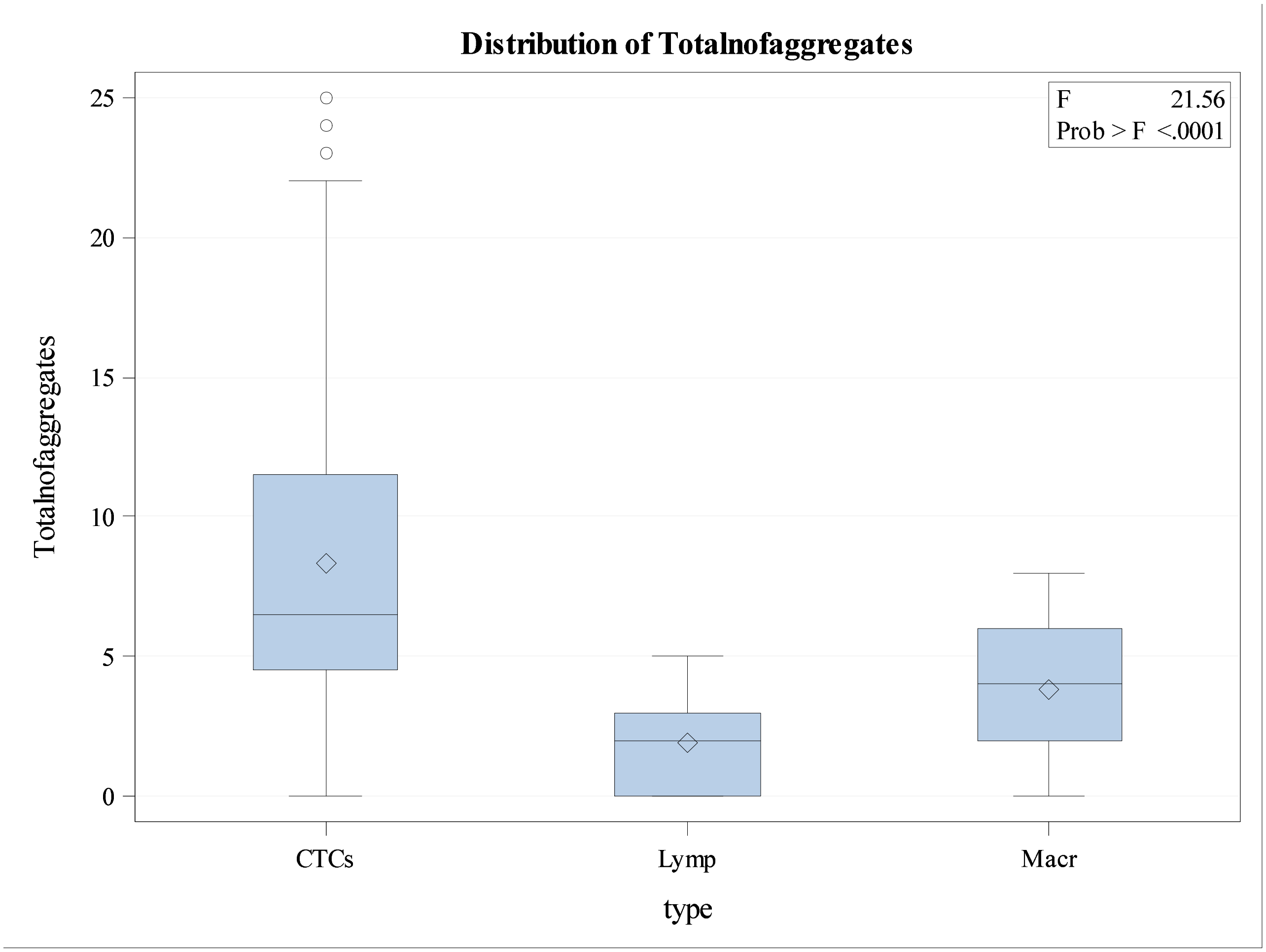

The GLM Procedure

Dependent Variable: acratio
acratio

pt=17AA1313

Source	DF	Sum of Squares	Mean Square	F Value	Pr > F
**Model**	2	123.7318413	61.8659206	23.16	<.0001
**Error**	102	272.5000089	2.6715687		
**Corrected Total**	104	396.2318501			

R-Square	Coeff Var	Root MSE	acratio Mean
0.312271	60.76809	1.634493	2.689723

Source	DF	Type III SS	Mean Square	F Value	Pr > F
**type**	2	123.7318413	61.8659206	23.16	<.0001

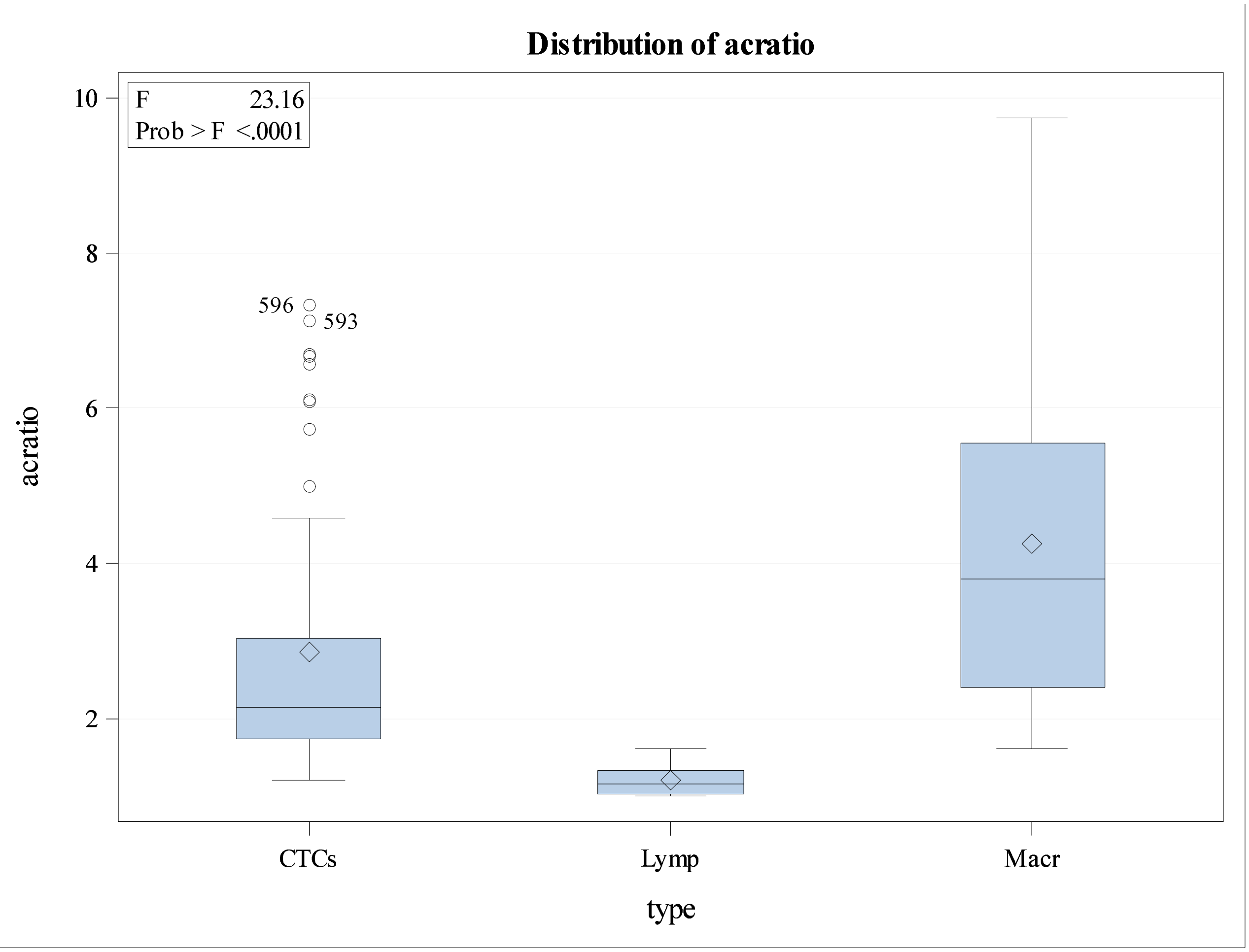

The GLM Procedure

Dependent Variable: AvIntallsignals
AvIntallsignals

pt=17AA1313

Source	DF	Sum of Squares	Mean Square	F Value	Pr > F
**Model**	2	2528963429	1264481715	81.75	<.0001
**Error**	102	1577662475	15467279		
**Corrected Total**	104	4106625904			

R-Square	Coeff Var	Root MSE	AvIntallsignals Mean
0.615825	29.99626	3932.846	13111.12

Source	DF	Type III SS	Mean Square	F Value	Pr > F
**type**	2	2528963429	1264481715	81.75	<.0001

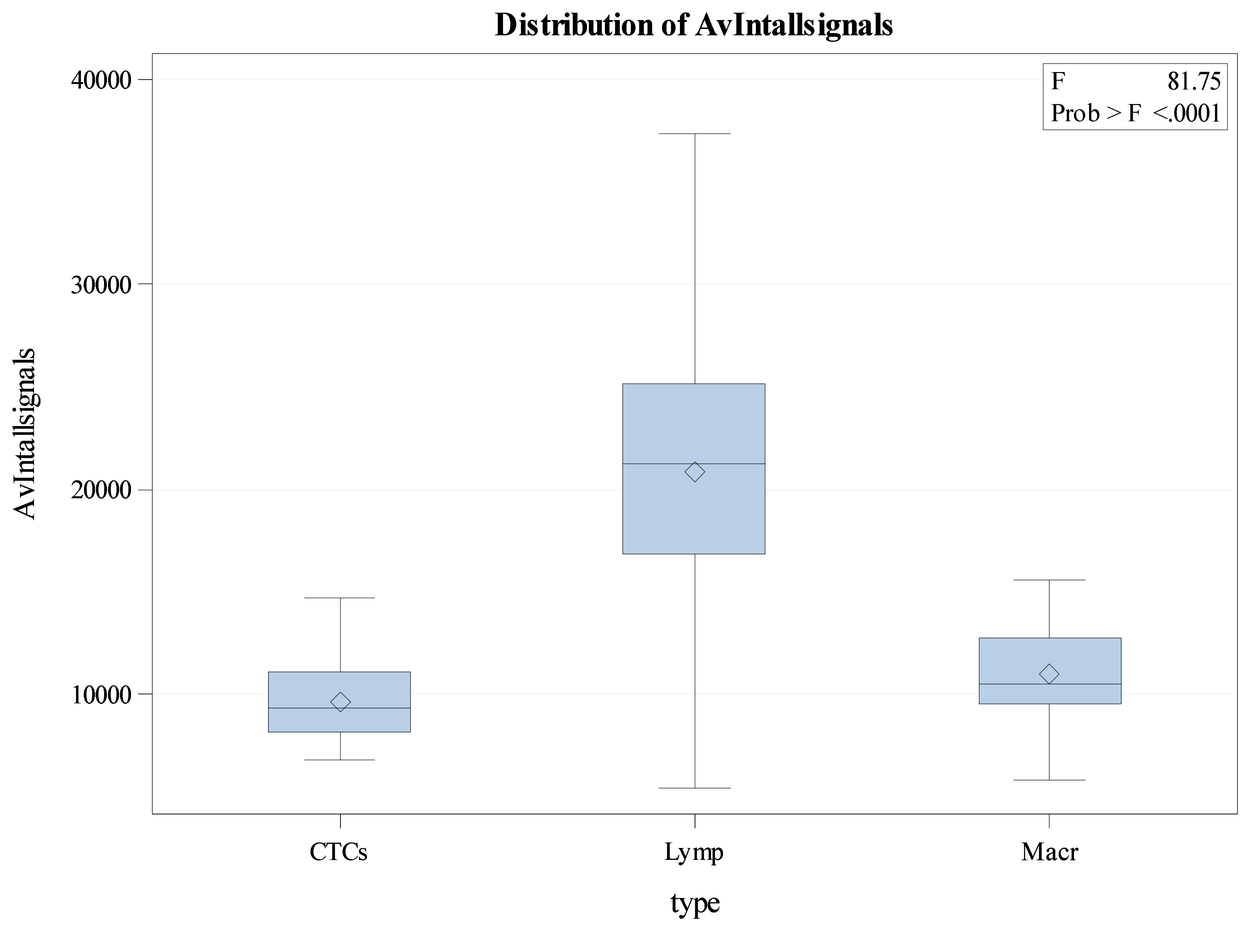

The GLM Procedure

Dependent Variable: Totalintensity Totalintensity

pt=17AA1313

Source	DF	Sum of Squares	Mean Square	F Value	Pr > F
**Model**	2	3.3490693E12	1.6745346E12	25.38	<.0001
**Error**	102	6.7310193E12	65990385003		
**Corrected Total**	104	1.0080089E13			

R-Square	Coeff Var	Root MSE	Totalintensity Mean
0.332246	46.58191	256885.9	551471.4

Source	DF	Type III SS	Mean Square	F Value	Pr > F
**type**	2	3.3490693E12	1.6745346E12	25.38	<.0001

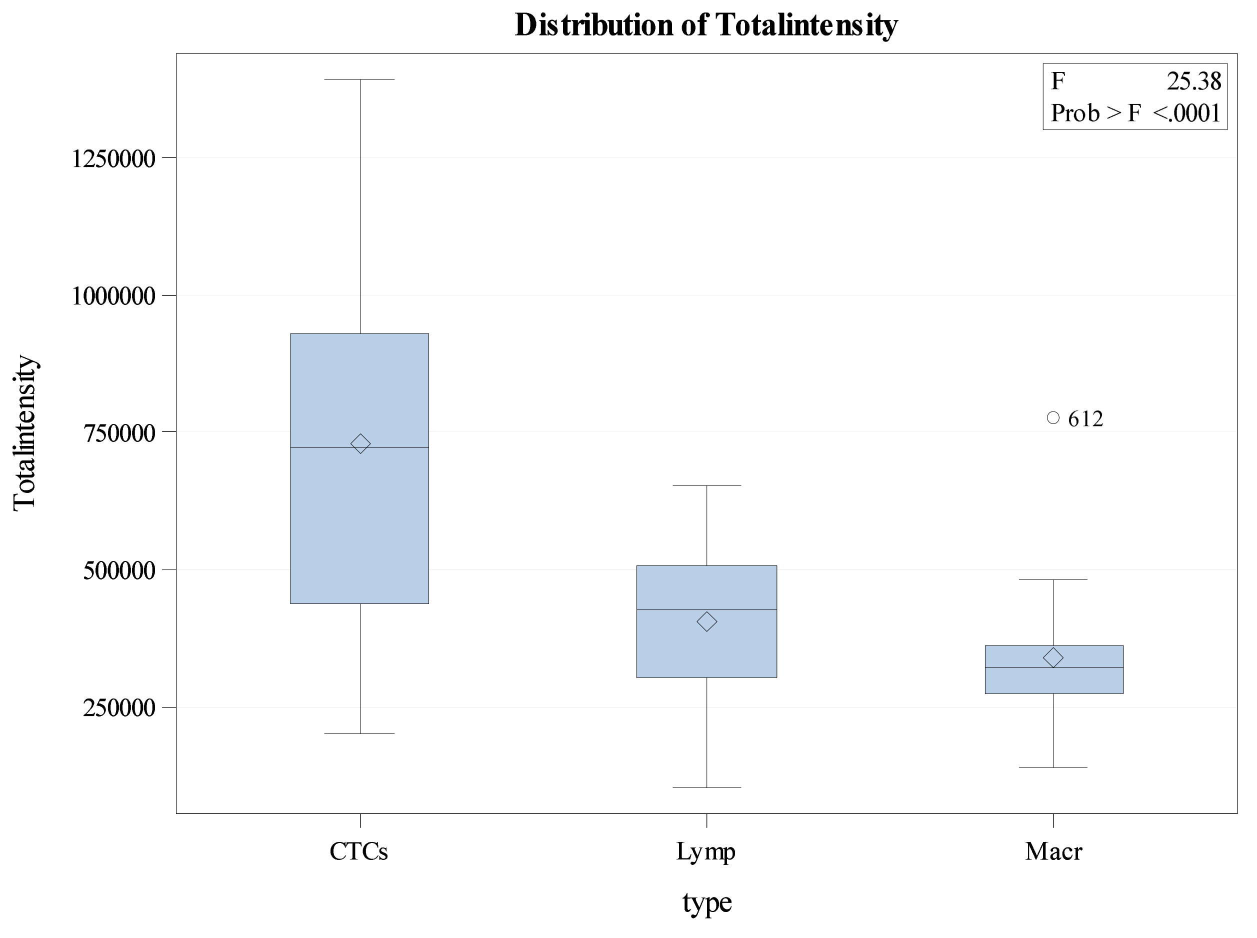

The GLM Procedure

Dependent Variable: Nuclearvolume Nuclearvolume

pt=17AA1313

Source	DF	Sum of Squares	Mean Square	F Value	Pr > F
**Model**	2	7.6470012E12	3.8235006E12	88.48	<.0001
**Error**	102	4.407574E12	43211509891		
**Corrected Total**	104	1.2054575E13			

R-Square	Coeff Var	Root MSE	Nuclearvolume Mean
0.634365	39.22162	207873.8	529998.0

Source	DF	Type III SS	Mean Square	F Value	Pr > F
**type**	2	7.6470012E12	3.8235006E12	88.48	<.0001

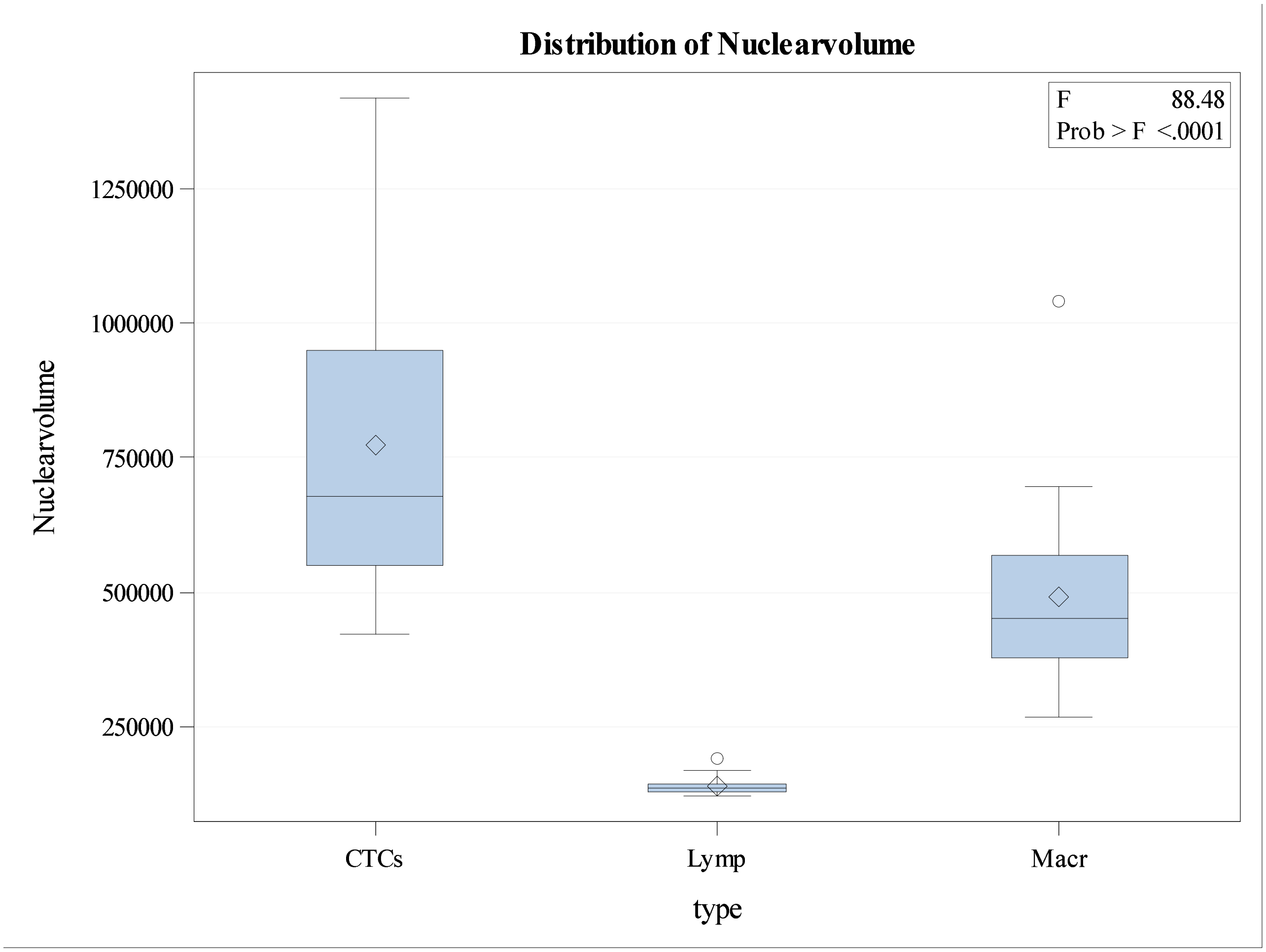

The GLM Procedure

Dependent Variable: telomereperkvol

pt=17AA1313

Source	DF	Sum of Squares	Mean Square	F Value	Pr > F
**Model**	2	0.07191542	0.03595771	31.38	<.0001
**Error**	102	0.11689247	0.00114600		
**Corrected Total**	104	0.18880789			

R-Square	Coeff Var	Root MSE	telomereperkvol Mean
0.380892	32.04627	0.033853	0.105637

Source	DF	Type III SS	Mean Square	F Value	Pr > F
**type**	2	0.07191542	0.03595771	31.38	<.0001

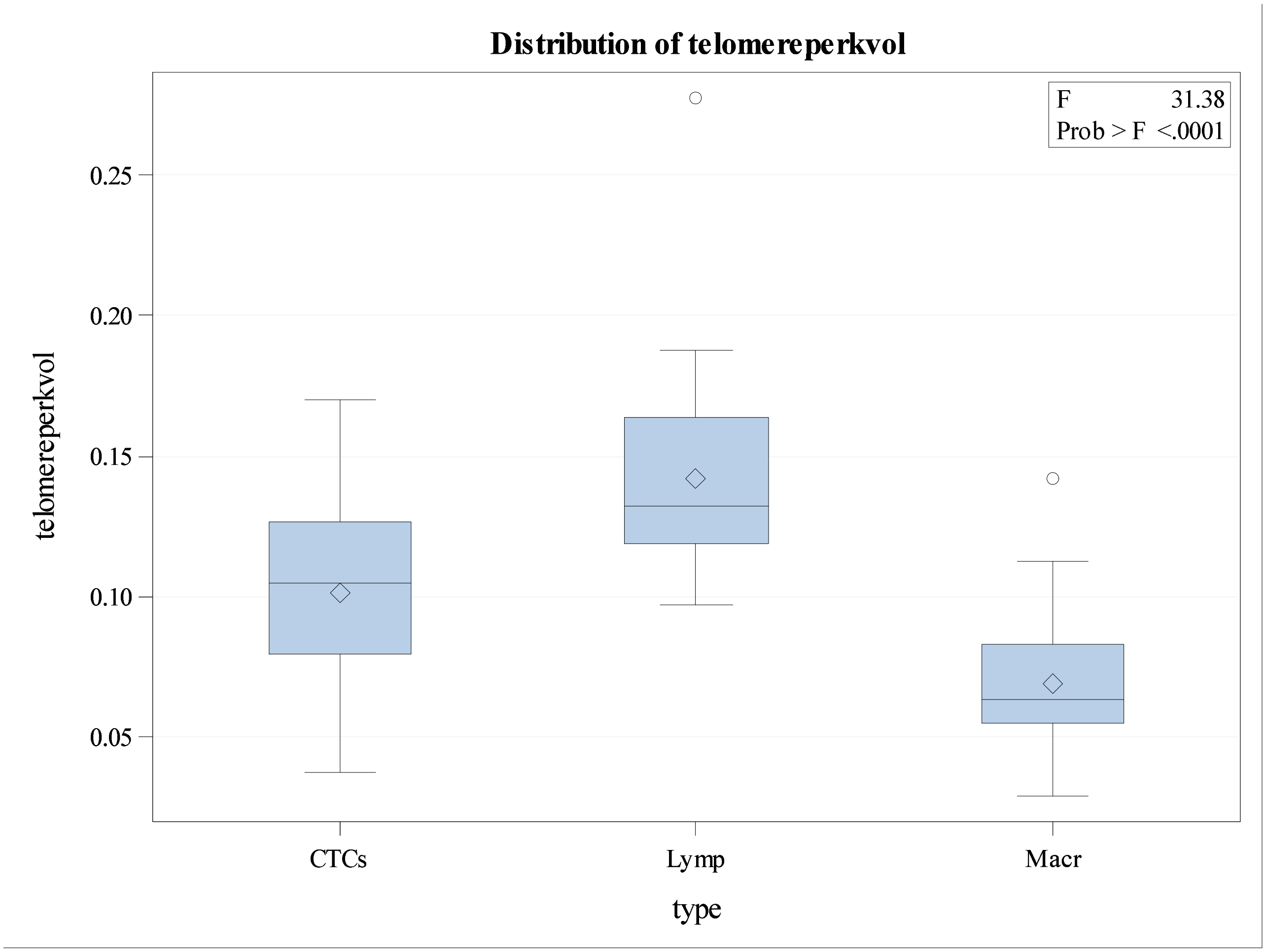

The GLM Procedure
Least Squares Means

pt=17AA1313

type	Totalnofsignals LSMEAN	Standard Error	Pr > |t|	LSMEAN Number
CTCs	78.1730769	4.0711088	<.0001	1
Lymp	19.6666667	5.3598638	0.0004	2
Macr	32.0000000	6.1213959	<.0001	3

Least Squares Means for effect type<br>Pr > |t| for H0: LSMean(i)=LSMean(j)<br><br>Dependent Variable: Totalnofsignals			
i/j	1	2	3
1		<.0001	<.0001
2	<.0001		0.1327
3	<.0001	0.1327	

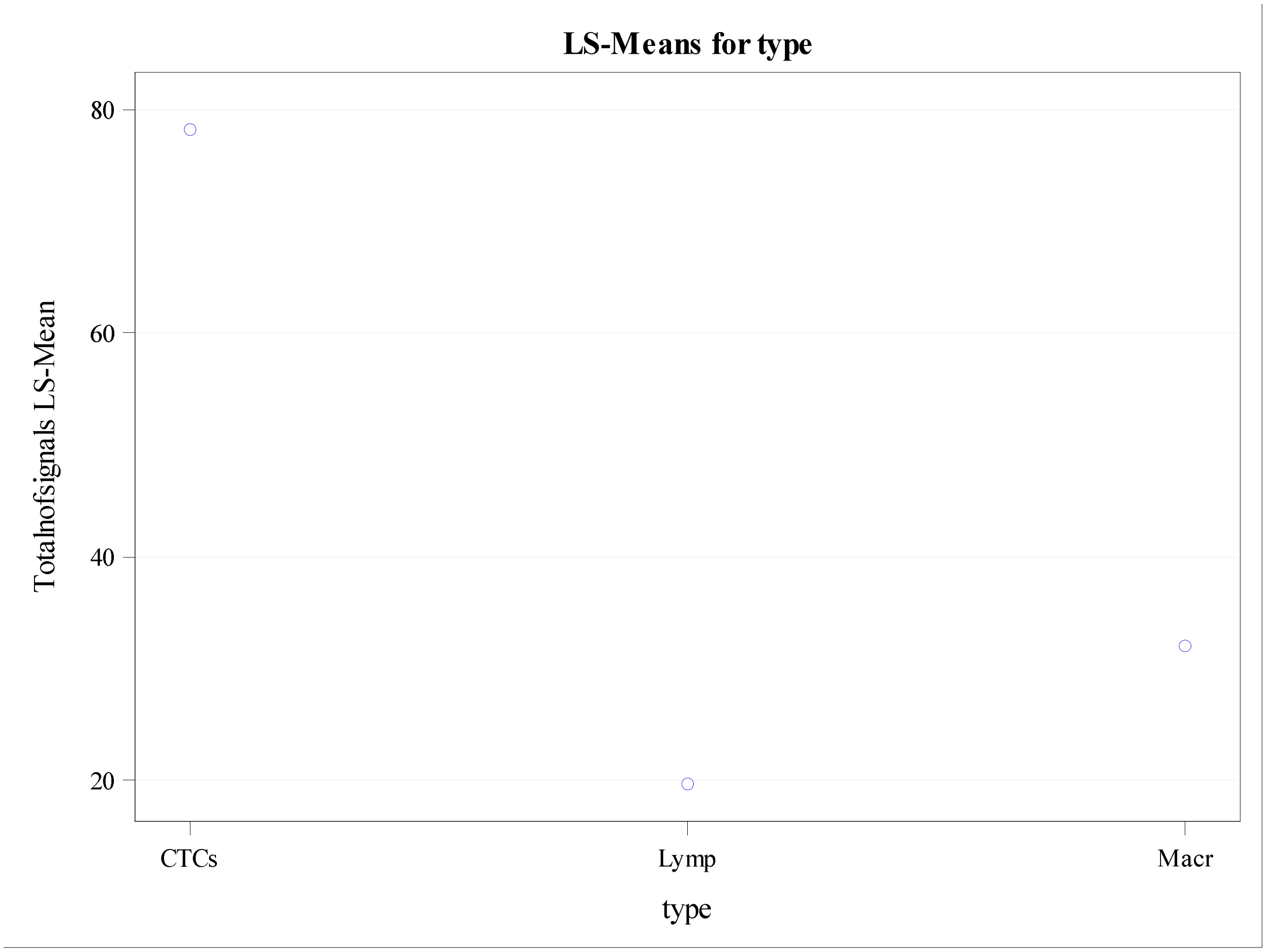

The GLM Procedure
Least Squares Means

pt=17AA1313

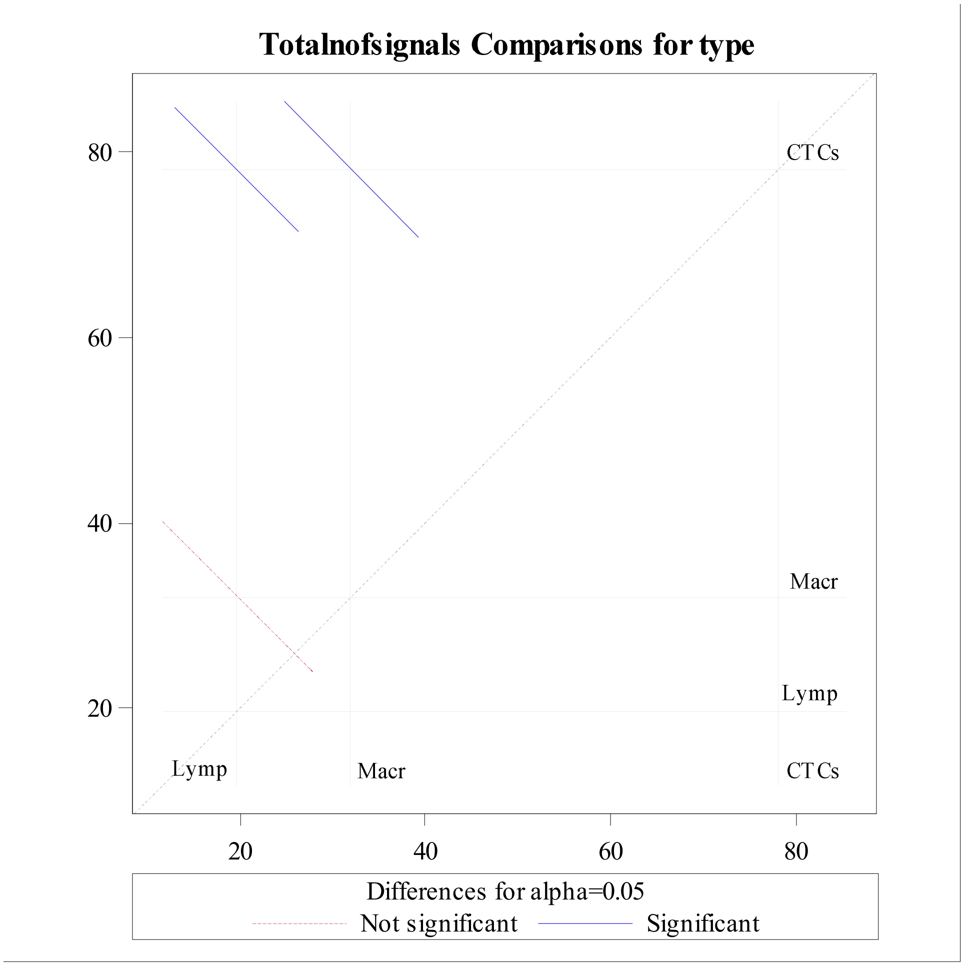

type	Totalnofaggregates LSMEAN	Standard Error	Pr > |t|	LSMEAN Number
**CTCs**	8.32692308	0.62251719	<.0001	1
**Lymp**	1.90000000	0.81958196	0.0224	2
**Macr**	3.82608696	0.93602856	<.0001	3

The GLM Procedure
Least Squares Means

pt=17AA1313

**Least Squares Means for effect type<br>Pr > |t| for H0: LSMean(i)=LSMean(j)<br><br>Dependent Variable: Totalnofaggregates**			
**i/j**	**1**	**2**	**3**
**1**		<.0001	0.0001
**2**	<.0001		0.1247
**3**	0.0001	0.1247	

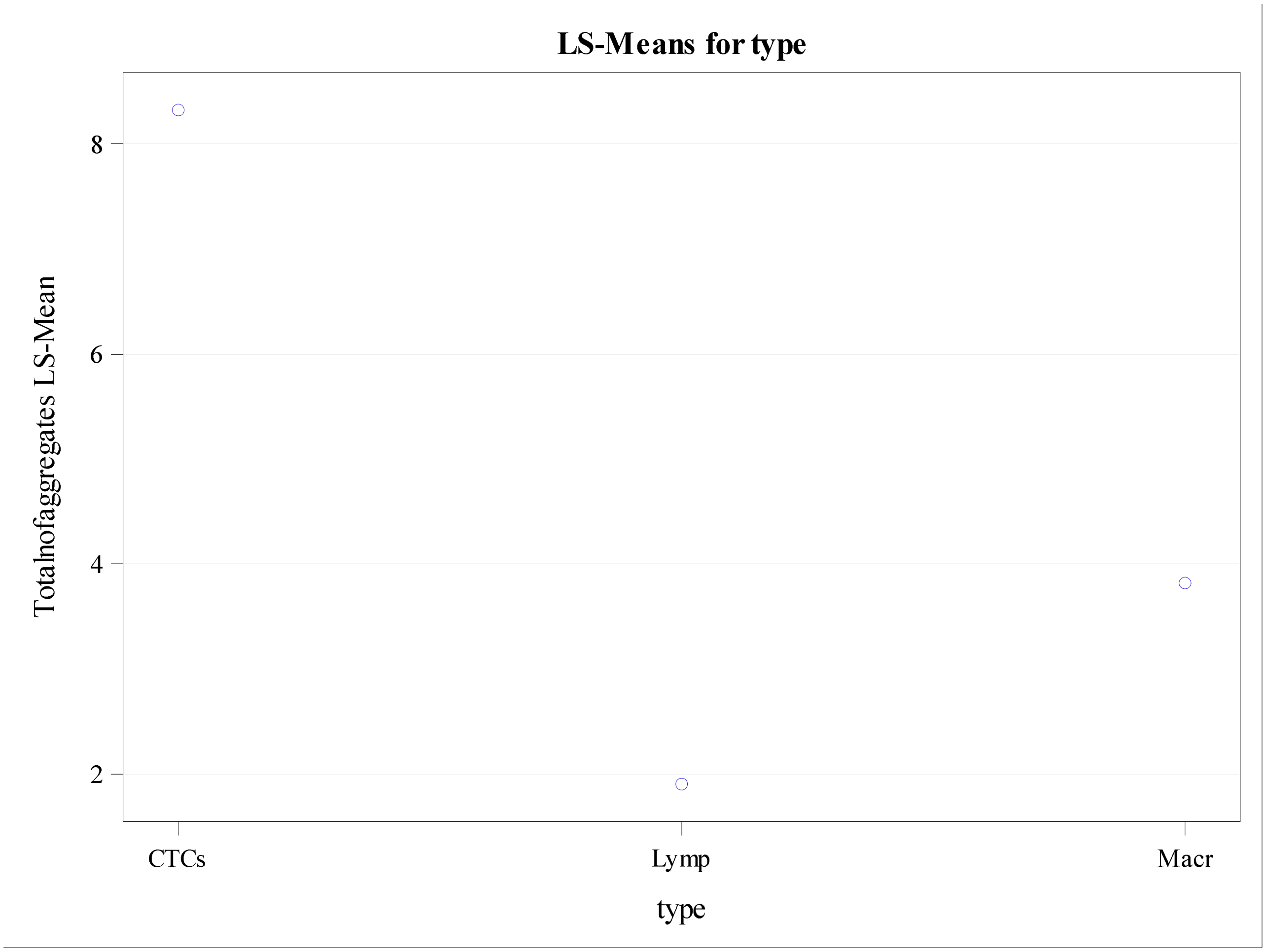

The GLM Procedure
Least Squares Means

pt=17AA1313

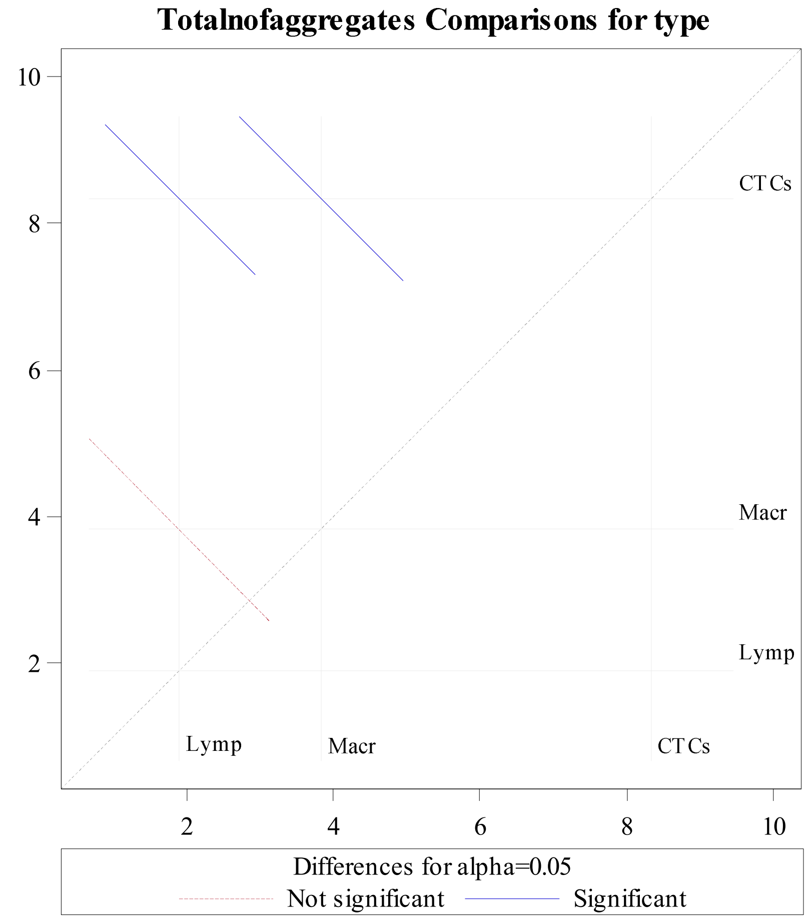

type	acratio LSMEAN	Standard Error	Pr > |t|	LSMEAN Number
**CTCs**	2.86059874	0.22666345	<.0001	1
**Lymp**	1.20058793	0.29841630	0.0001	2
**Macr**	4.24574665	0.34081544	<.0001	3

Least Squares Means for effect type
Pr > |t| for H0: LSMean(i)=LSMean(j)

Dependent Variable: acratio

i/j	1	2	3
**1**		<.0001	0.0010
**2**	<.0001		<.0001
**3**	0.0010	<.0001	

The GLM Procedure
Least Squares Means

pt=17AA1313

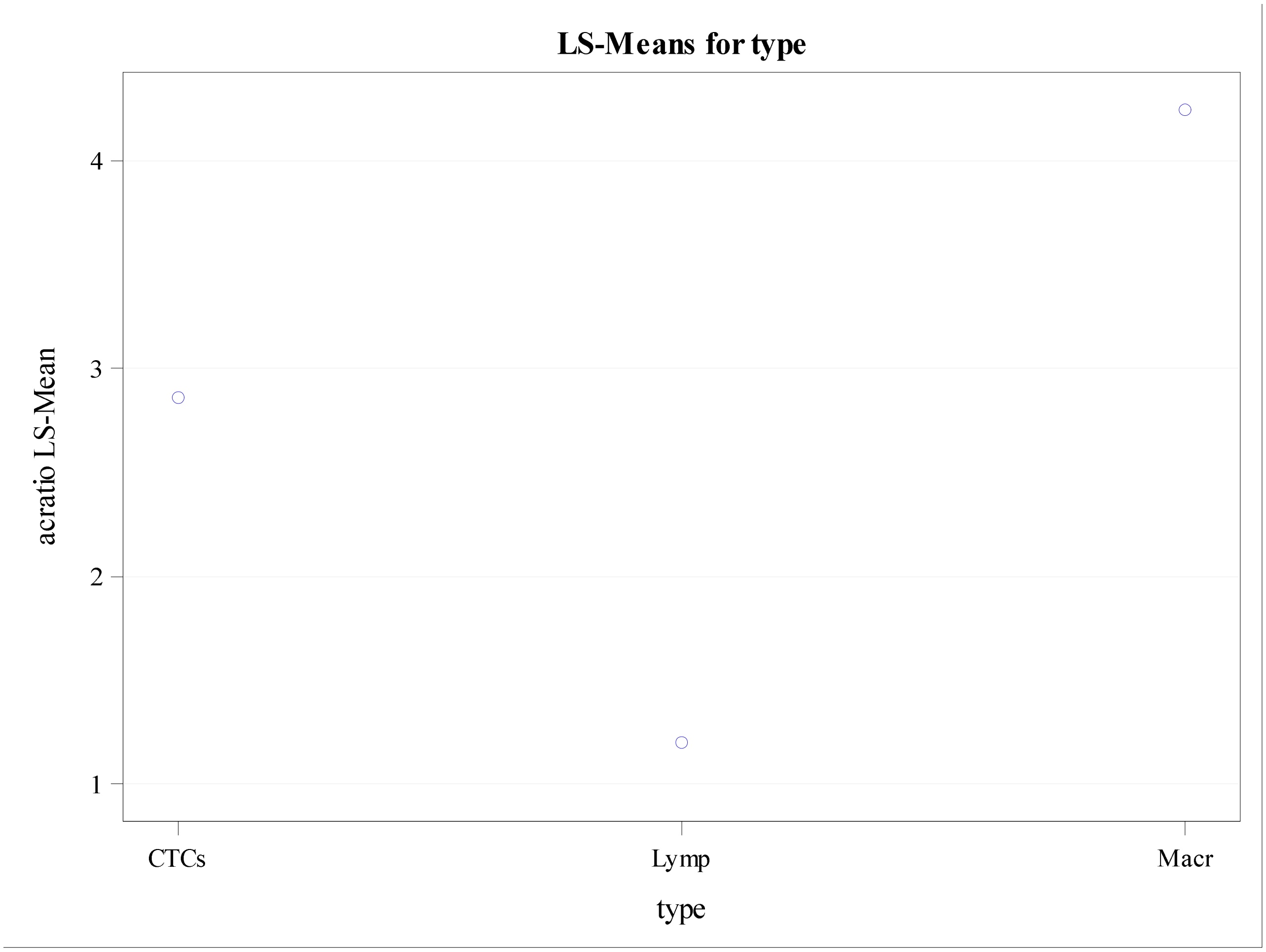

The GLM Procedure
Least Squares Means

pt=17AA1313

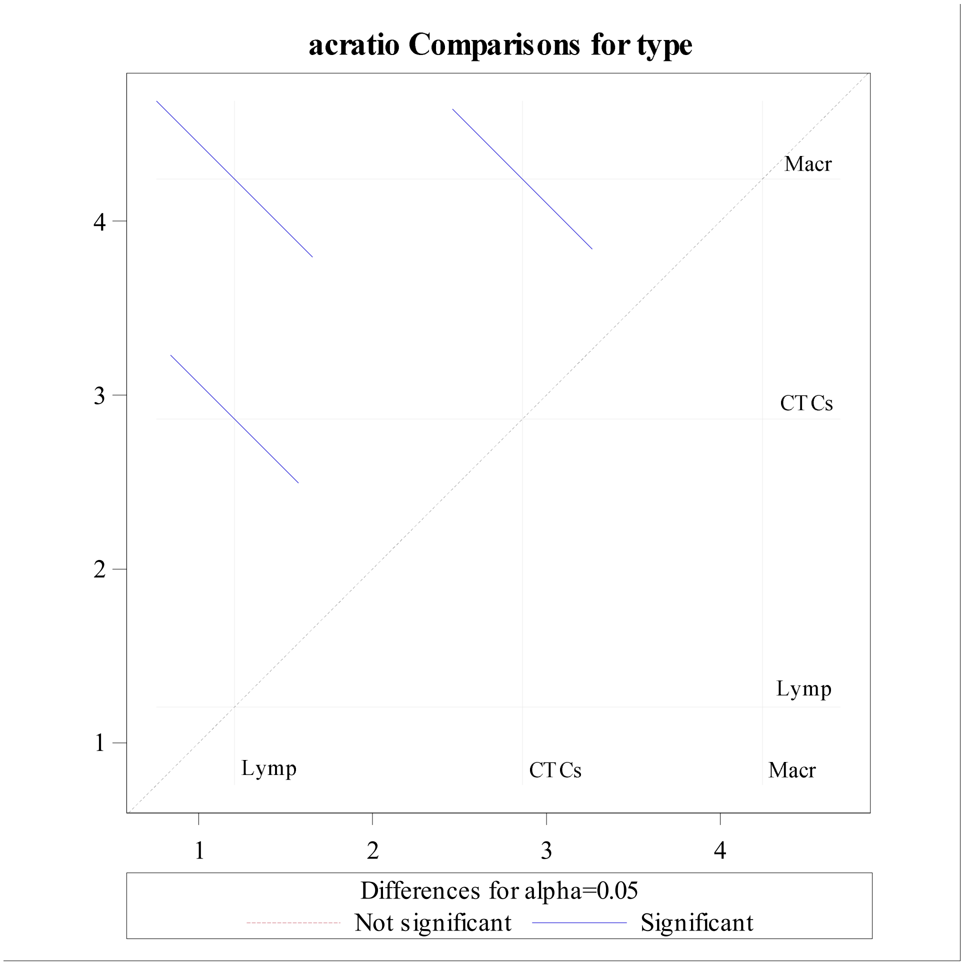

type	AvIntallsignals LSMEAN	Standard Error	Pr > |t|	LSMEAN Number
**CTCs**	9591.2456	545.3876	<.0001	1
**Lymp**	20821.2834	718.0362	<.0001	2
**Macr**	11012.3626	820.0551	<.0001	3

Least Squares Means for effect type
Pr > |t| for H0: LSMean(i)=LSMean(j)

Dependent Variable: AvIntallsignals

i/j	1	2	3
**1**		<.0001	0.1521
**2**	<.0001		<.0001
**3**	0.1521	<.0001	

The GLM Procedure
Least Squares Means

pt=17AA1313

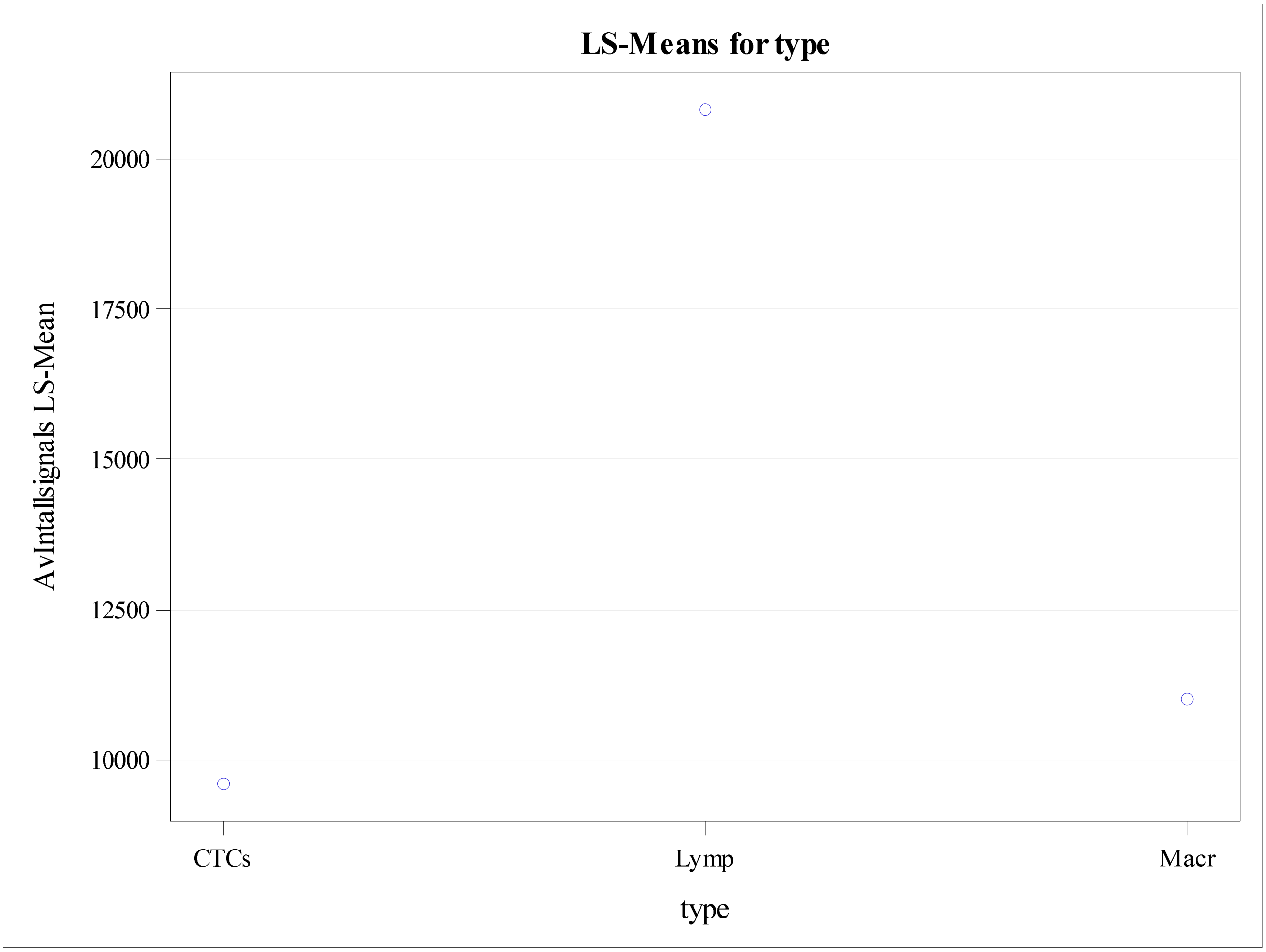

The GLM Procedure
Least Squares Means

pt=17AA1313

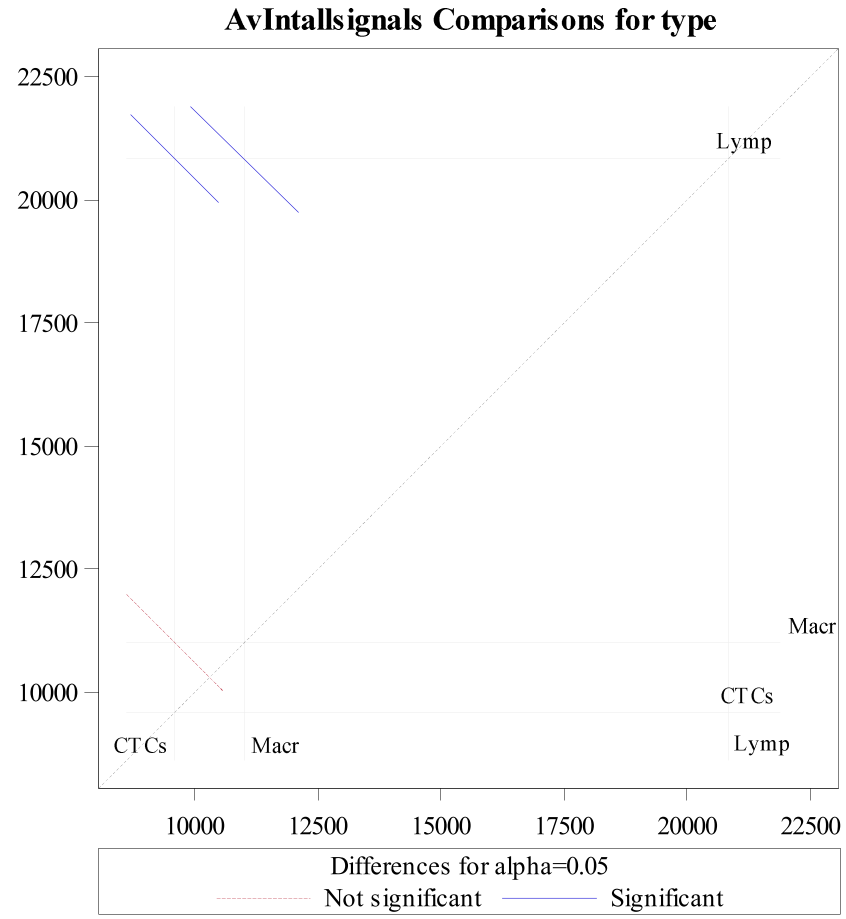

type	Totalintensity LSMEAN	Standard Error	Pr > |t|	LSMEAN Number
**CTCs**	730303.596	35623.670	<.0001	1
**Lymp**	404073.000	46900.741	<.0001	2
**Macr**	339414.087	53564.421	<.0001	3

Least Squares Means for effect type
Pr > |t| for H0: LSMean(i)=LSMean(j)

Dependent Variable: Totalintensity

i/j	1	2	3
**1**		<.0001	<.0001
**2**	<.0001		0.3659
**3**	<.0001	0.3659	

The GLM Procedure
Least Squares Means

pt=17AA1313

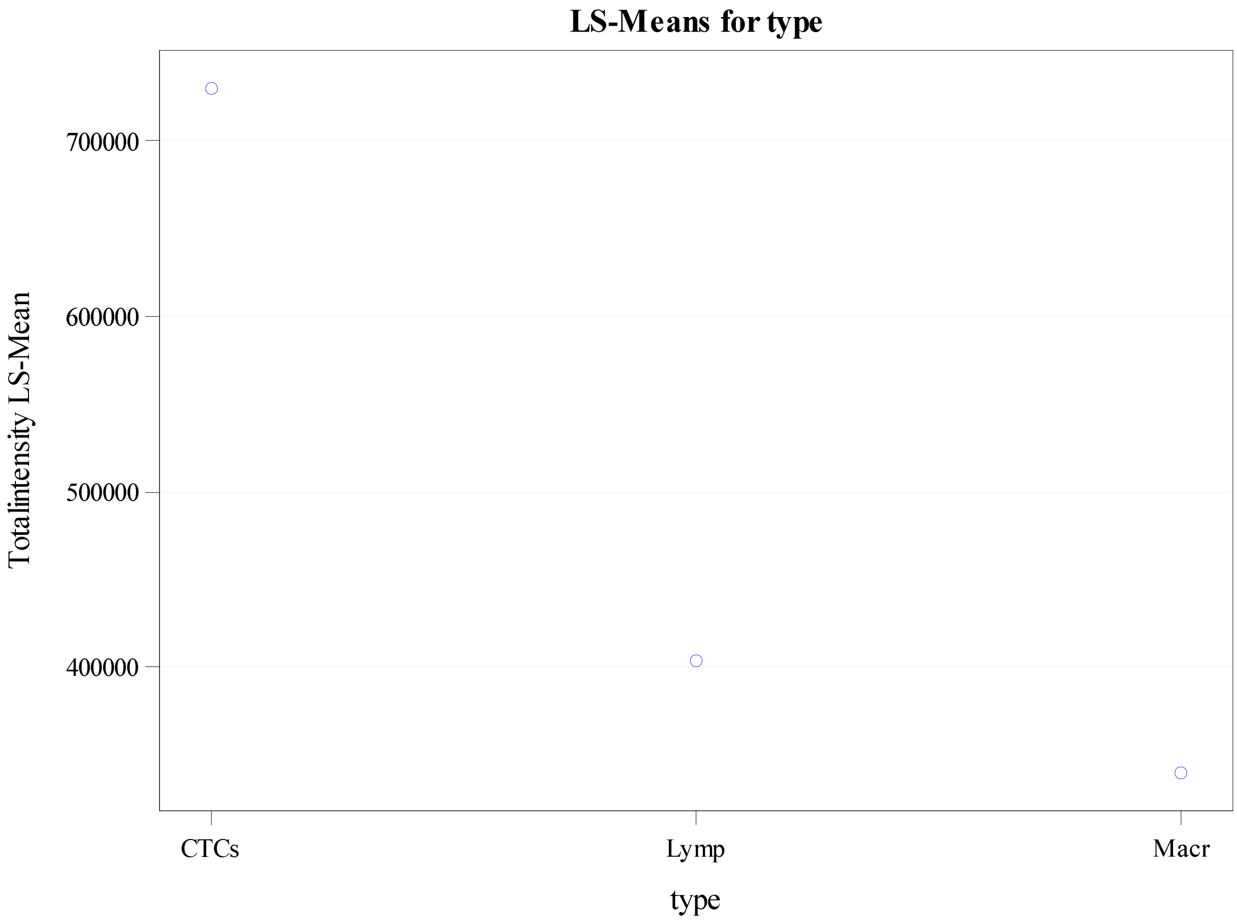

The GLM Procedure
Least Squares Means

pt=17AA1313

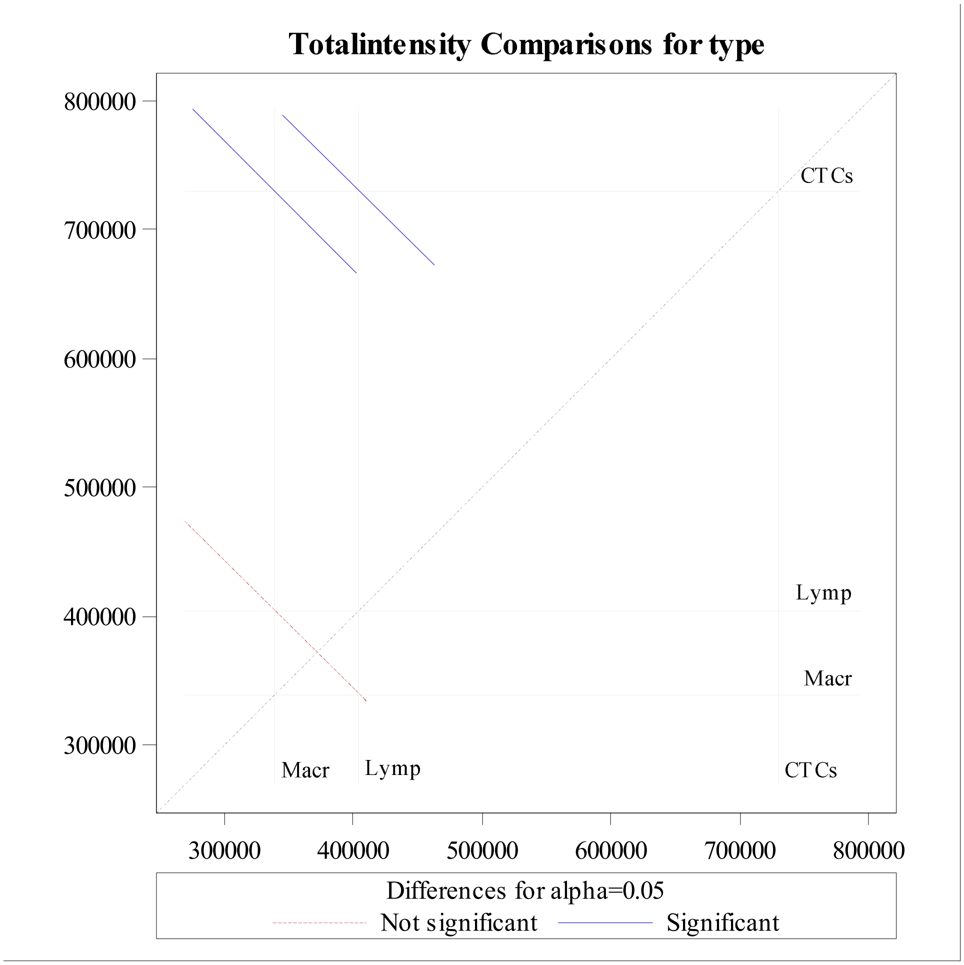

type	Nuclearvolume LSMEAN	Standard Error	Pr > |t|	LSMEAN Number
**CTCs**	772099.269	28826.907	<.0001	1
**Lymp**	139915.400	37952.387	0.0004	2
**Macr**	491442.043	43344.680	<.0001	3

Least Squares Means for effect type<br>Pr > |t| for H0: LSMean(i)=LSMean(j)<br><br>Dependent Variable: Nuclearvolume			
**i/j**	**1**	**2**	**3**
**1**		<.0001	<.0001
**2**	<.0001		<.0001
**3**	<.0001	<.0001	

The GLM Procedure
Least Squares Means

pt=17AA1313

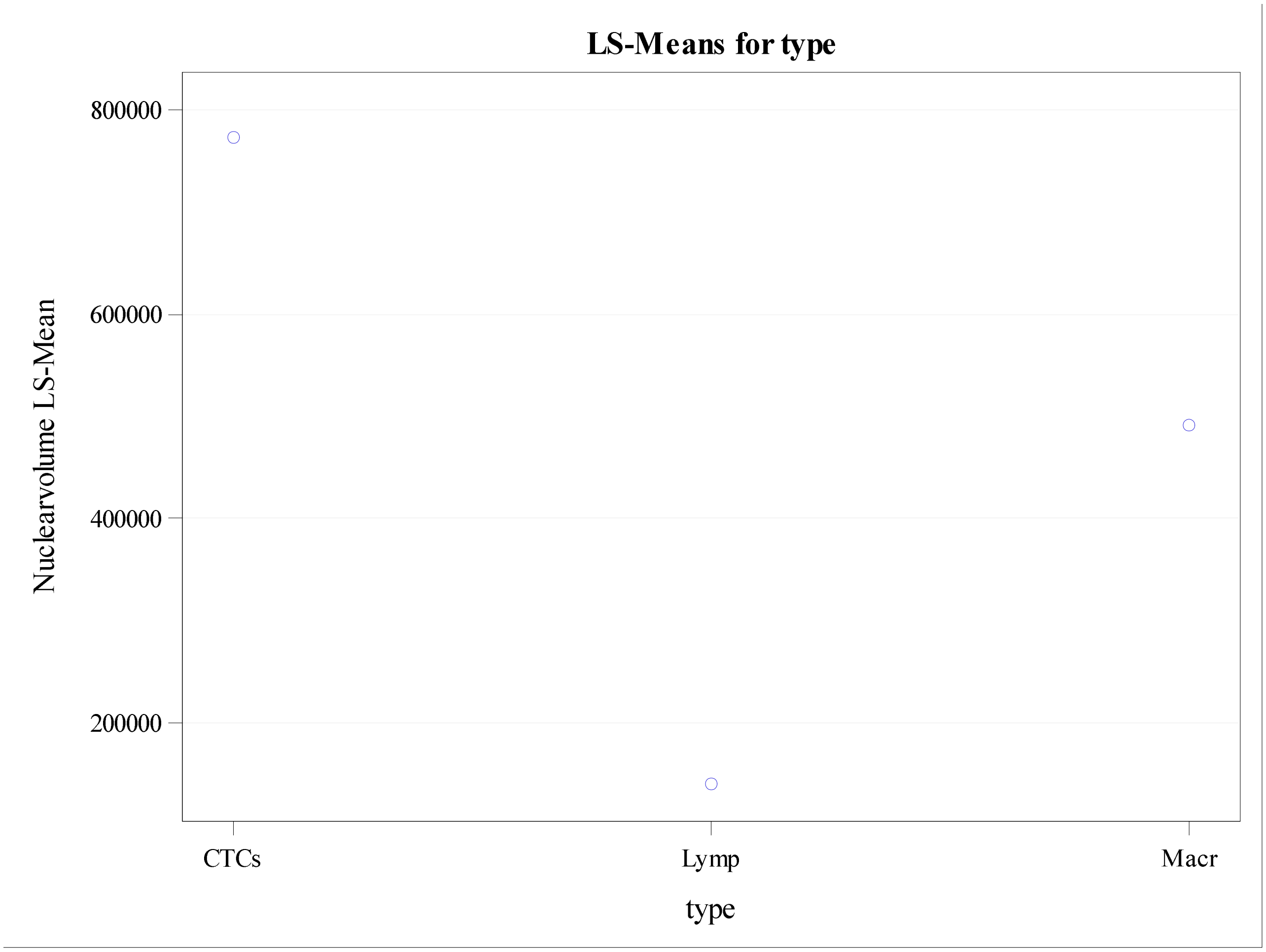

The GLM Procedure
Least Squares Means

pt=17AA1313

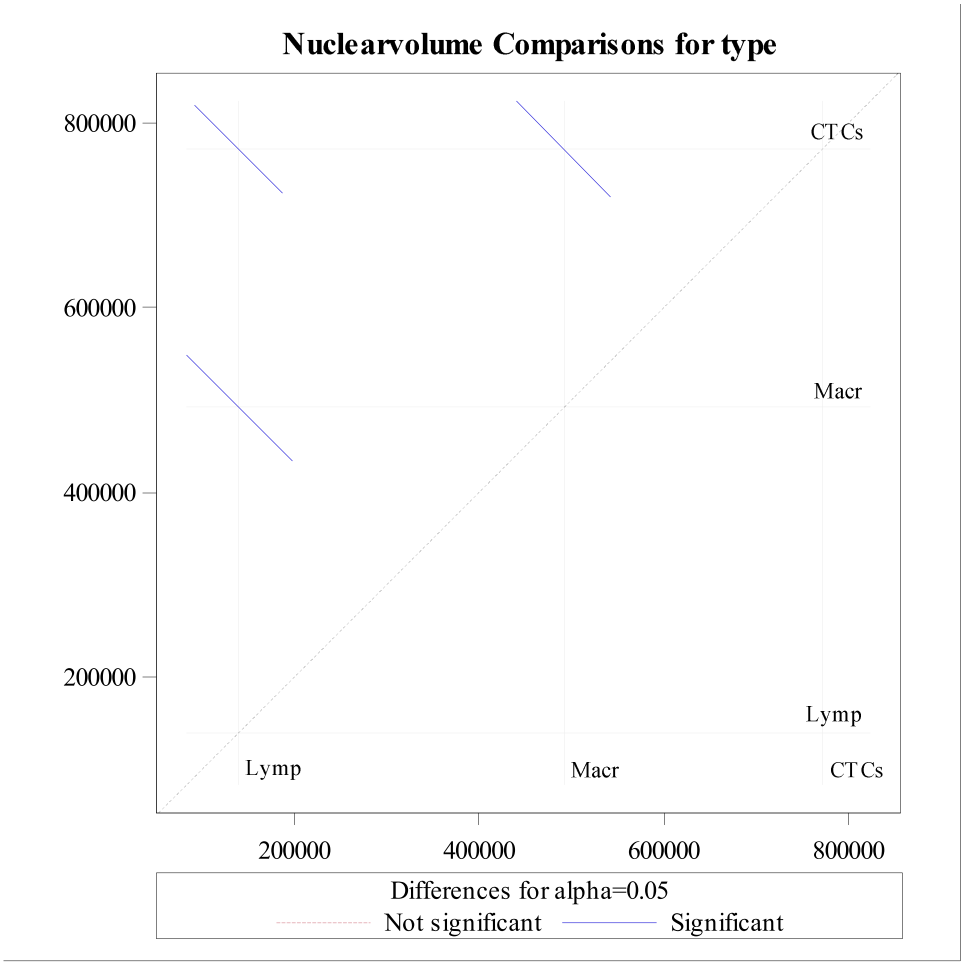

type	telomereperkvol LSMEAN	Standard Error	Pr > |t|	LSMEAN Number
**CTCs**	0.10110829	0.00469452	<.0001	1
**Lymp**	0.14185622	0.00618063	<.0001	2
**Macr**	0.06863290	0.00705877	<.0001	3

Least Squares Means for effect type<br>Pr > |t| for H0: LSMean(i)=LSMean(j)<br><br>Dependent Variable: telomereperkvol			
**i/j**	**1**	**2**	**3**
**1**		<.0001	0.0002
**2**	<.0001		<.0001
**3**	0.0002	<.0001	

The GLM Procedure
Least Squares Means

pt=17AA1313

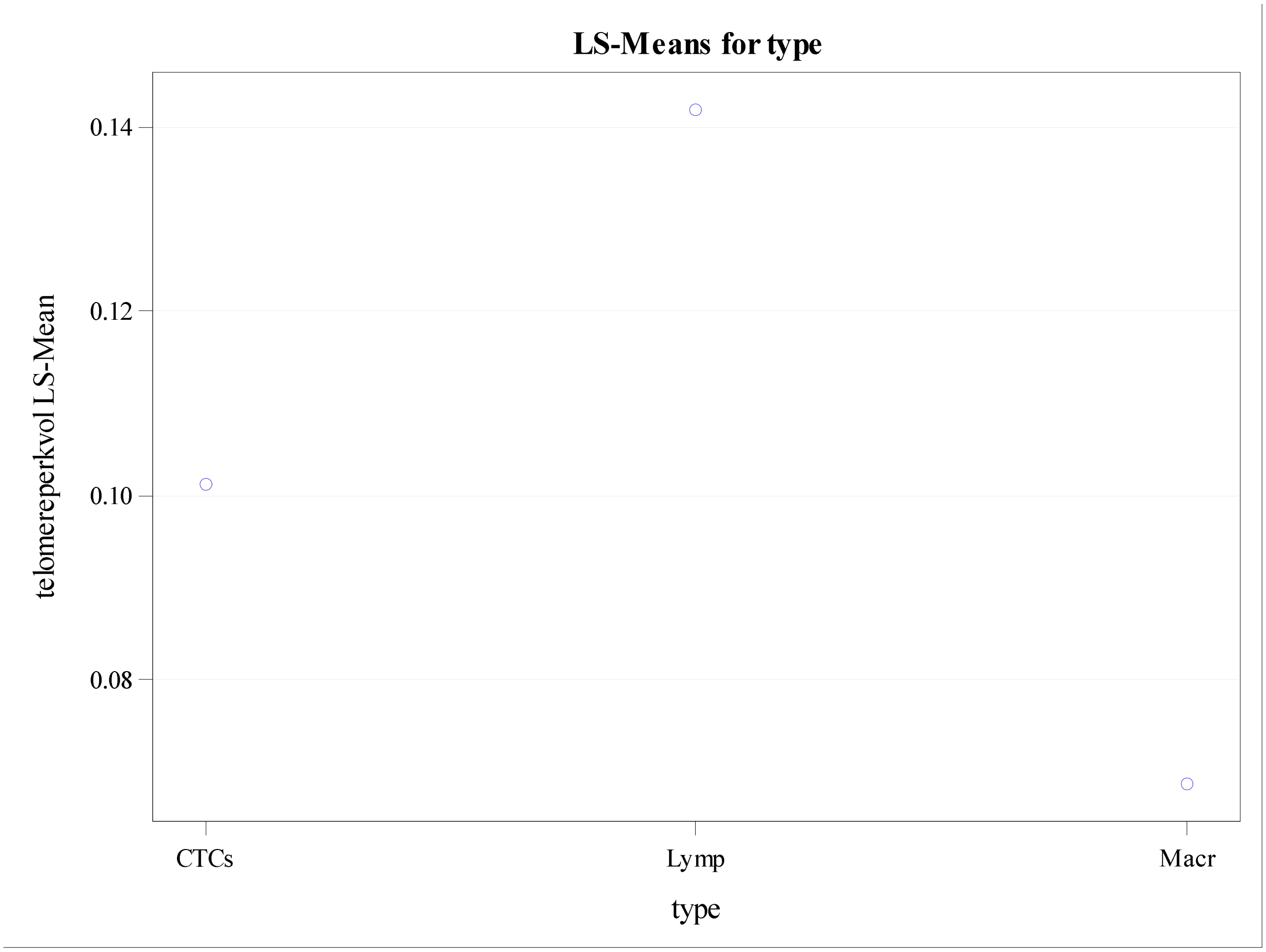

The GLM Procedure
Least Squares Means

pt=17AA1313

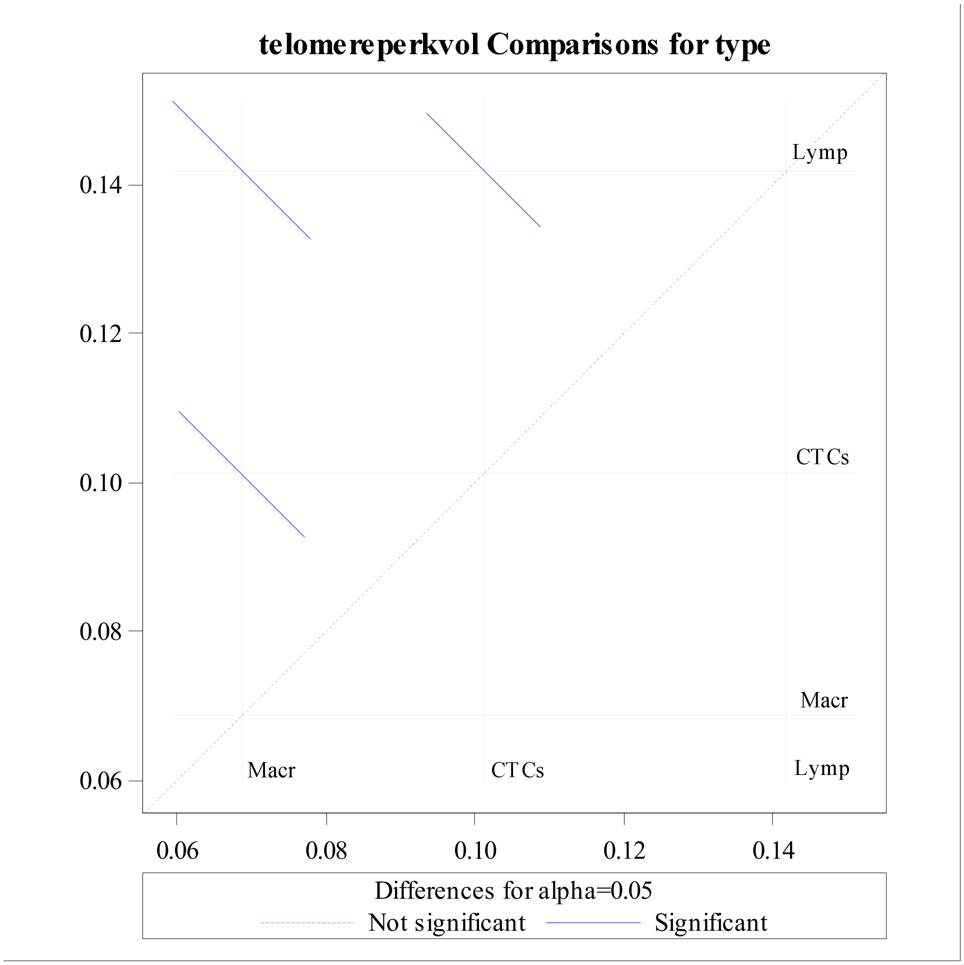

Note: To ensure overall protection level, only probabilities associated with pre-planned comparisons should be used.
