# Supplementary material for: Telomere-Associated Changes in Nuclear Architecture of Cancer-Associated Macrophage-like Cells in Liquid Biopsies from Melanoma Patients
Source: Biomedicines. 2022 Sep 25;10(10):2391. doi: 10.3390/biomedicines10102391 (PMC9598704; doi:10.3390/biomedicines10102391)
Supplement: Supplementary file 1 [file biomedicines-10-02391-s001.zip › Supplementary Figure S1.pdf]

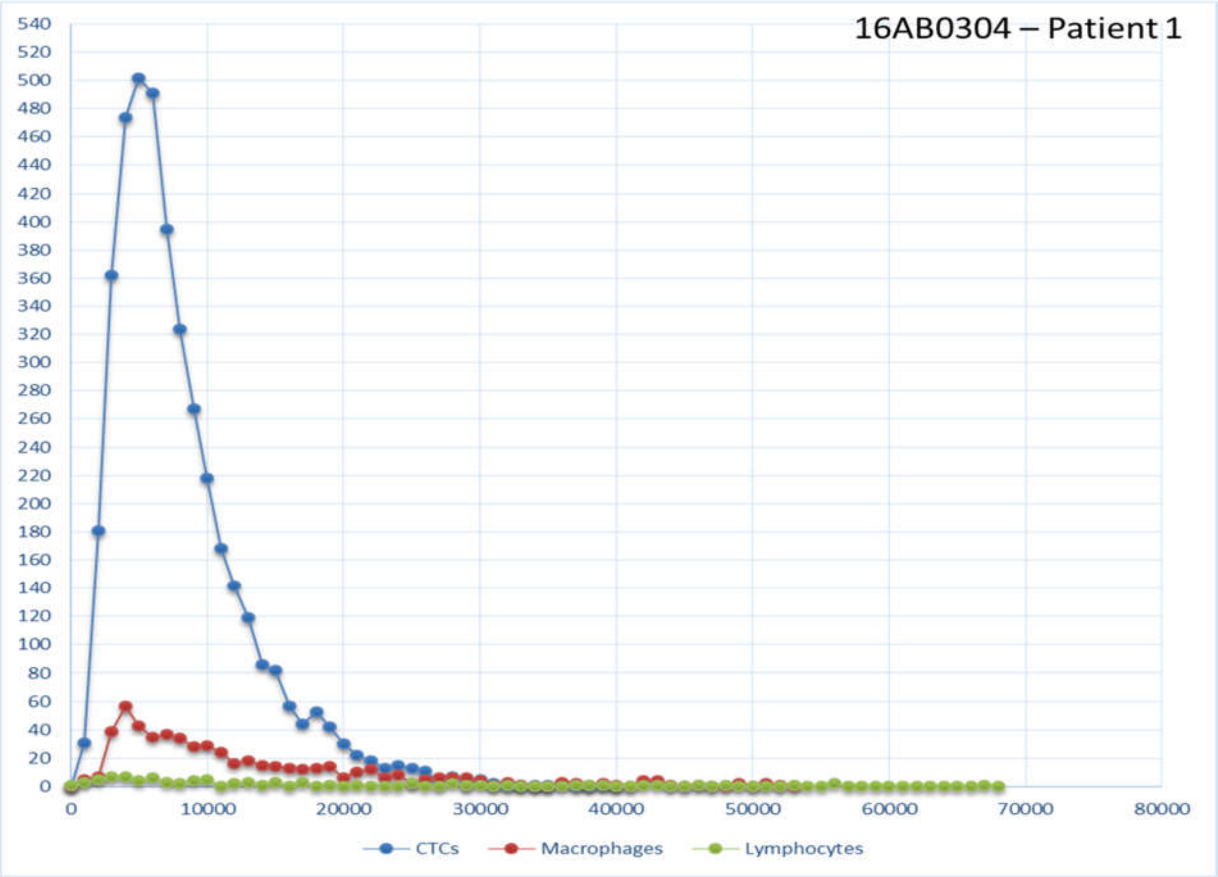
16AB0304 – Patient 1
540
520
500
480
460
440
420
400
380
360
340
320
300
280
260
240
220
200
180
160
140
120
100
80
60
40
20
0
0
10000
20000
30000
40000
50000
60000
70000
80000
CTCs
Macrophages
Lymphocytes

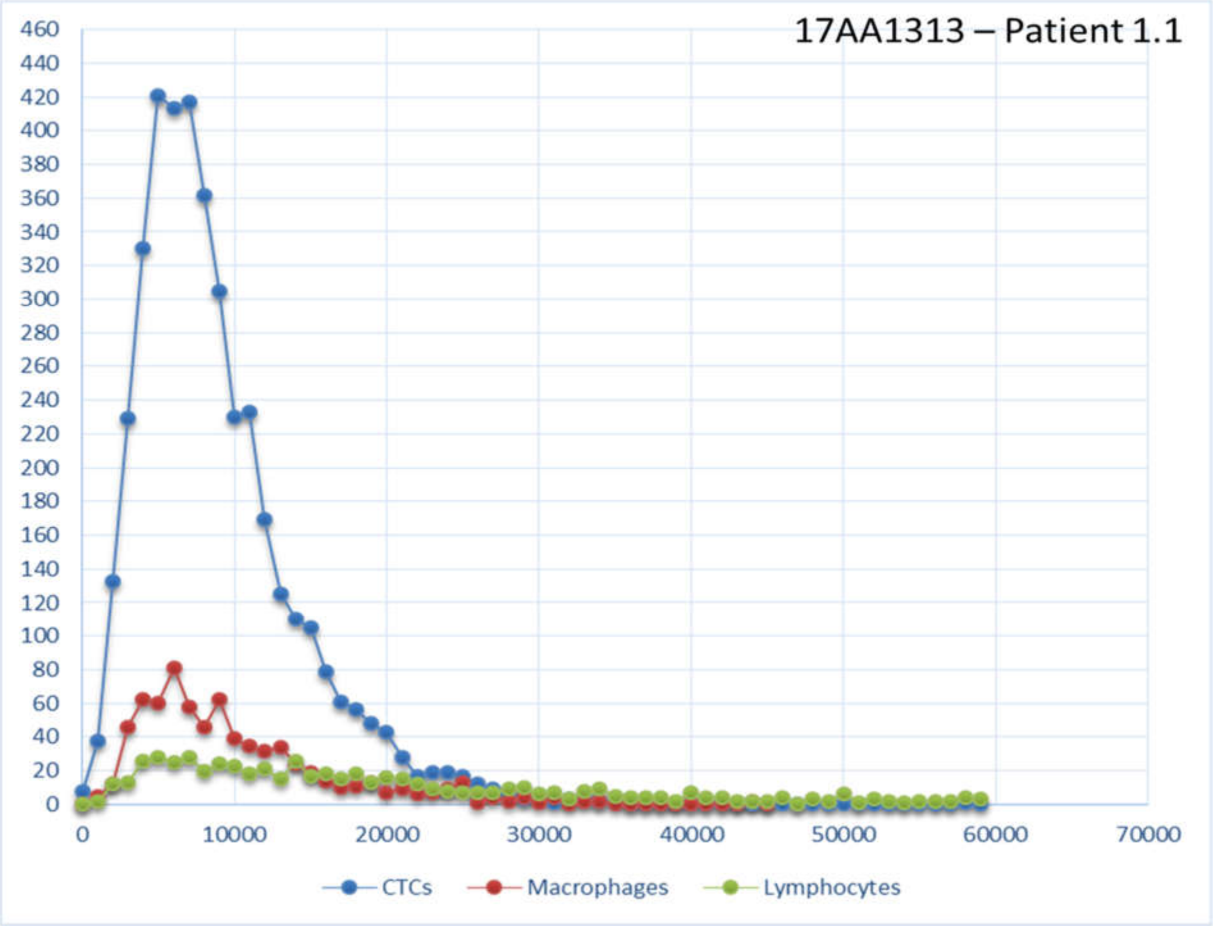
17AA1313 – Patient 1.1
460
440
420
400
380
360
340
320
300
280
260
240
220
200
180
160
140
120
100
80
60
40
20
0
0
10000
20000
30000
40000
50000
60000
70000
CTCs
Macrophages
Lymphocytes

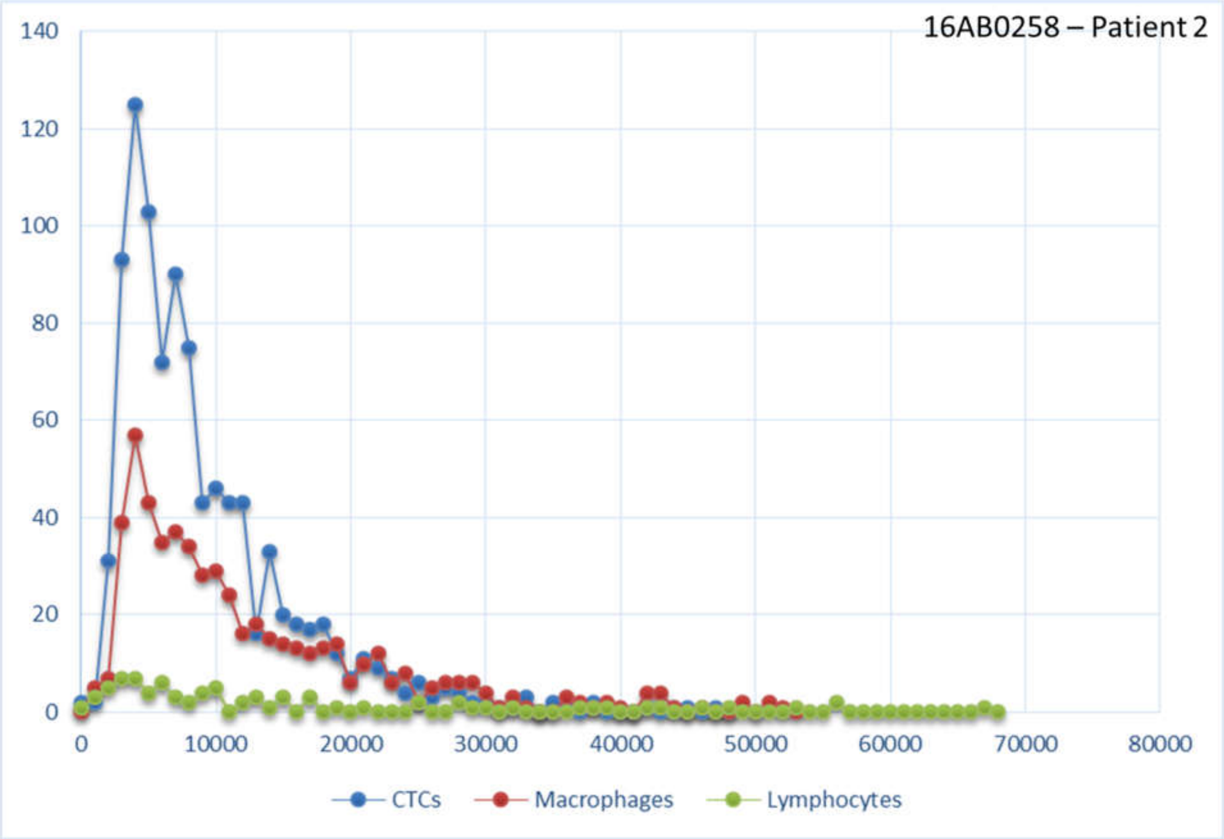

16AB0258 – Patient 2
140
120
100
80
60
40
20
0
0
10000
20000
30000
40000
50000
60000
70000
80000
CTCs
Macrophages
Lymphocytes

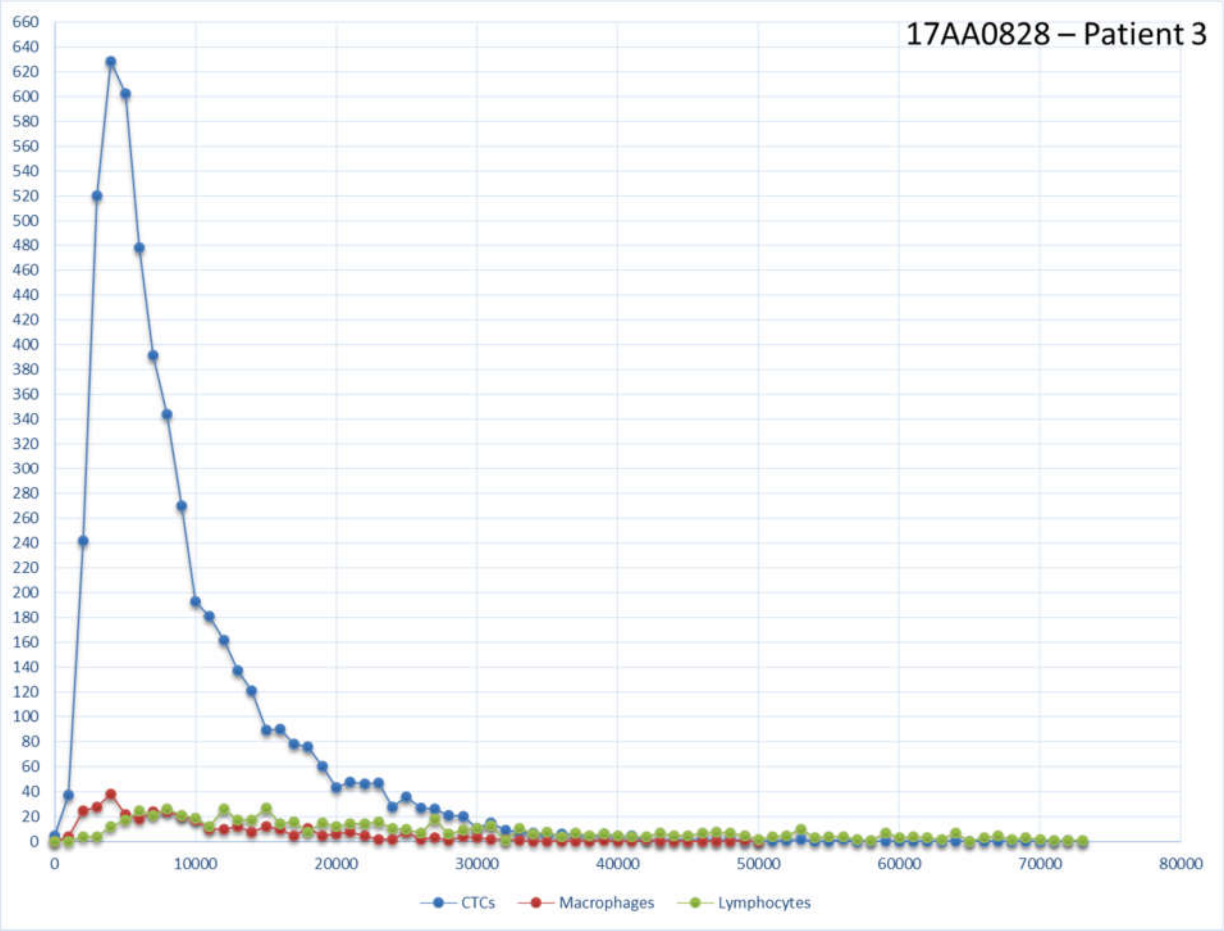

17AA0828 – Patient 3
660
640
620
600
580
560
540
520
500
480
460
440
420
400
380
360
340
320
300
280
260
240
220
200
180
160
140
120
100
80
60
40
20
0
0
10000
20000
30000
40000
50000
60000
70000
80000
CTCs
Macrophages
Lymphocytes

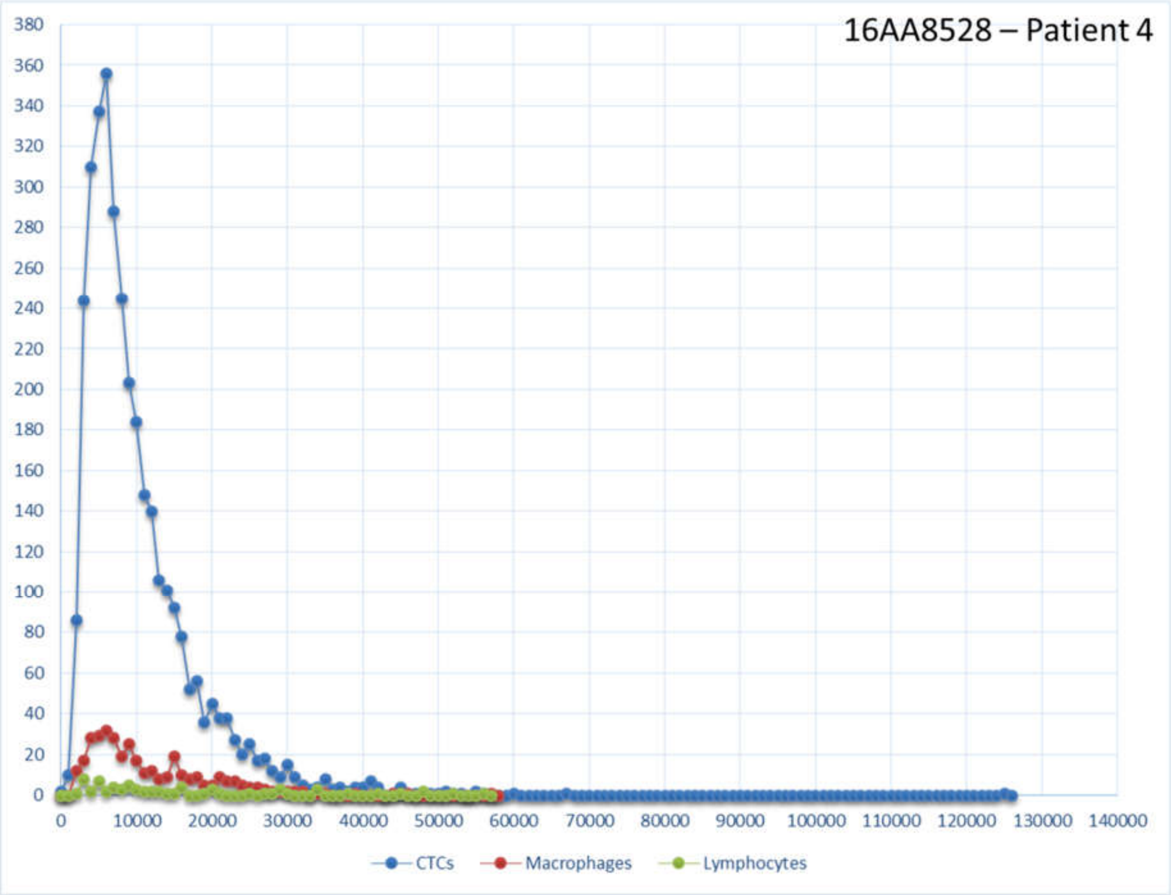
16AA8528 – Patient 4
CTCs
Macrophages
Lymphocytes

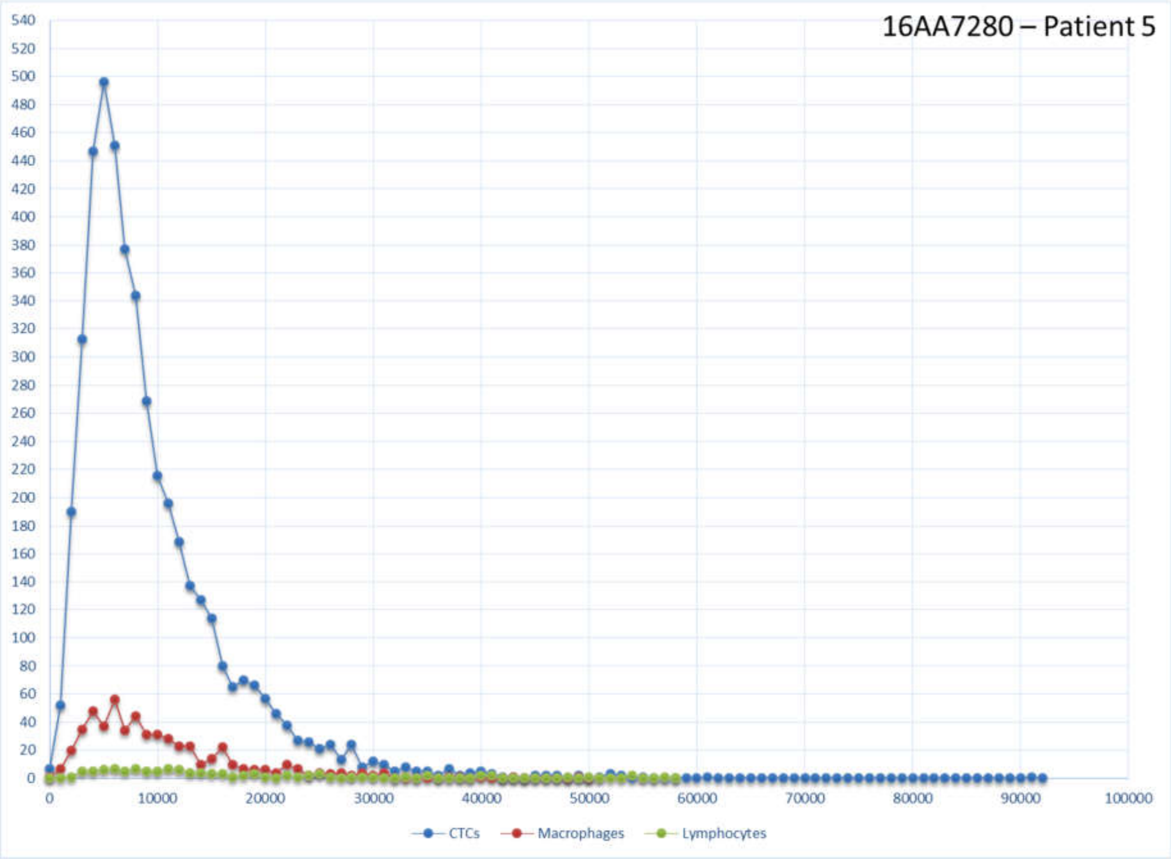
16AA7280 – Patient 5
CTCs
Macrophages
Lymphocytes

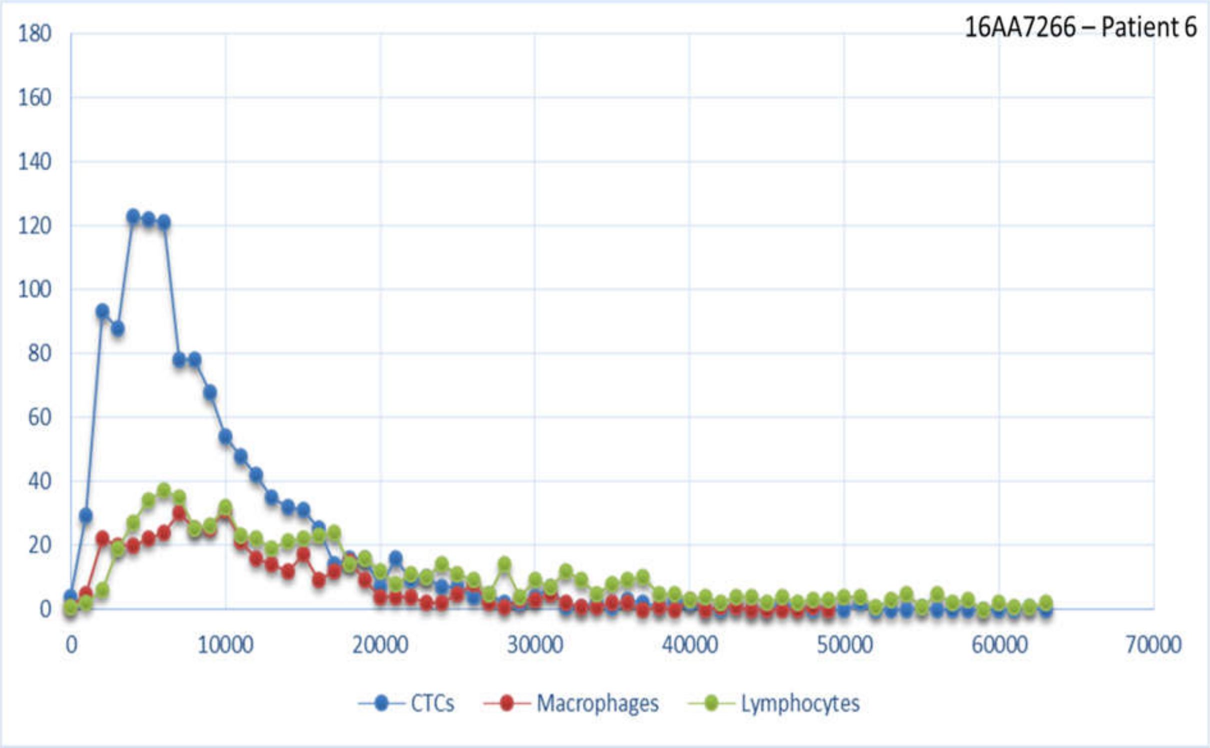
16AA7266 – Patient 6
180
160
140
120
100
80
60
40
20
0
0
10000
20000
30000
40000
50000
60000
70000
CTCs
Macrophages
Lymphocytes

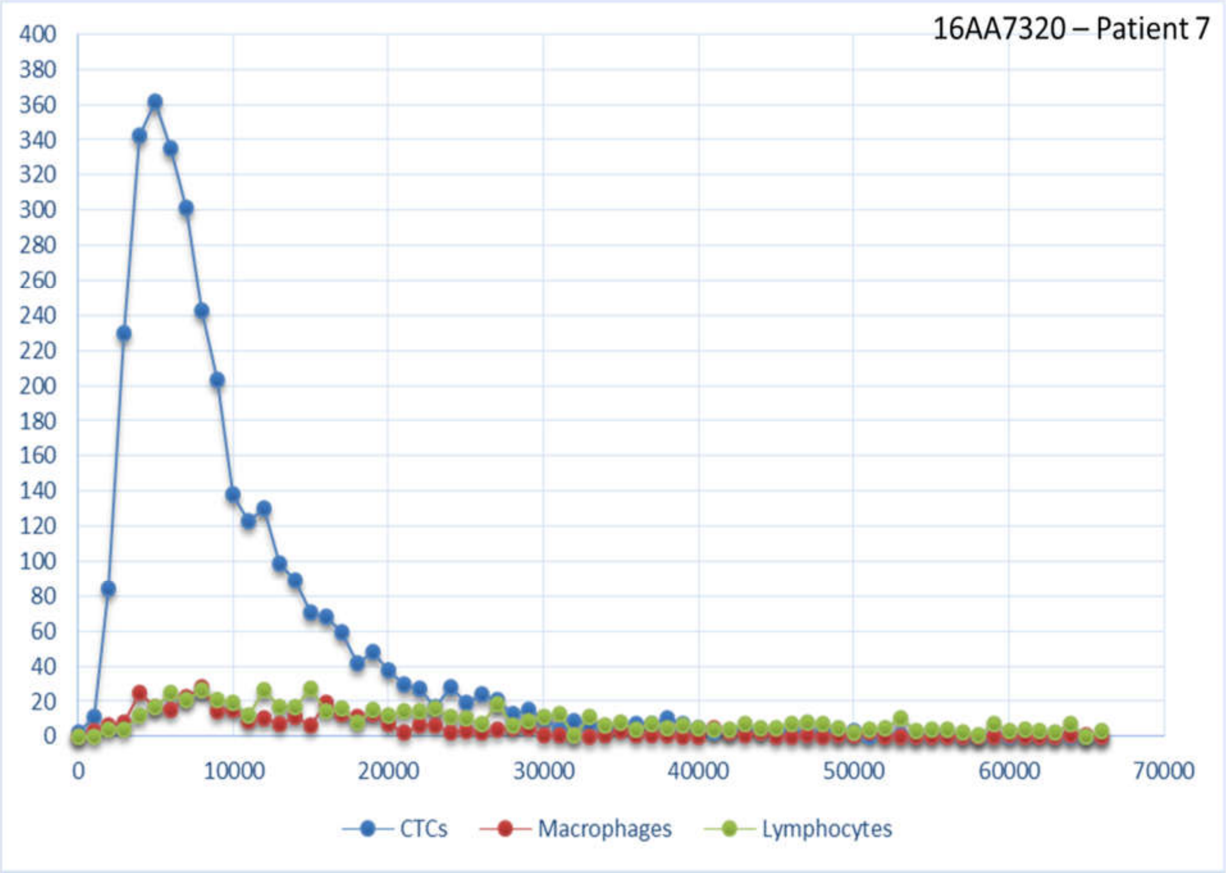
16AA7320 – Patient 7
400
380
360
340
320
300
280
260
240
220
200
180
160
140
120
100
80
60
40
20
0
0
10000
20000
30000
40000
50000
60000
70000
CTCs
Macrophages
Lymphocytes

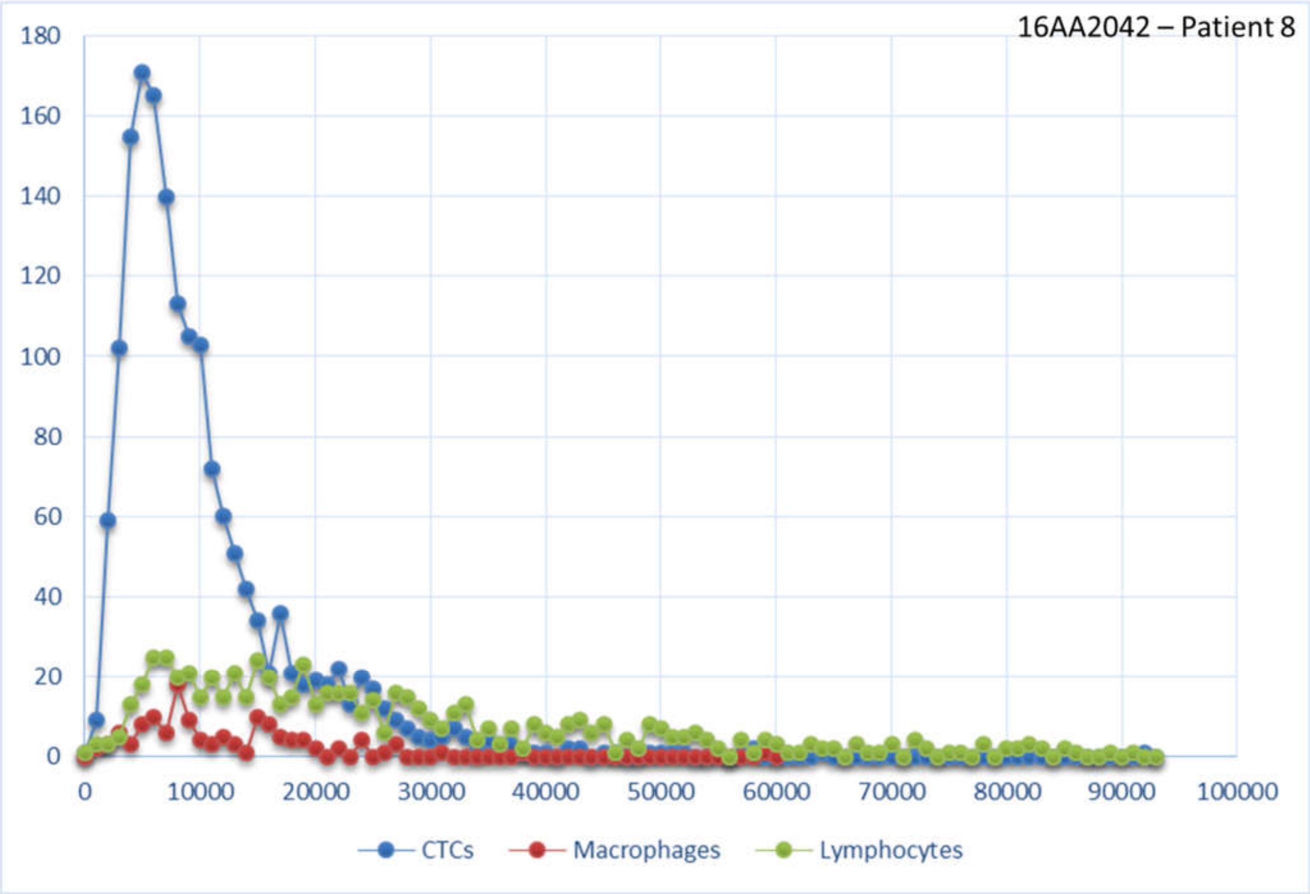
16AA2042 – Patient 8
180
160
140
120
100
80
60
40
20
0
0
10000
20000
30000
40000
50000
60000
70000
80000
90000
100000
CTCs
Macrophages
Lymphocytes

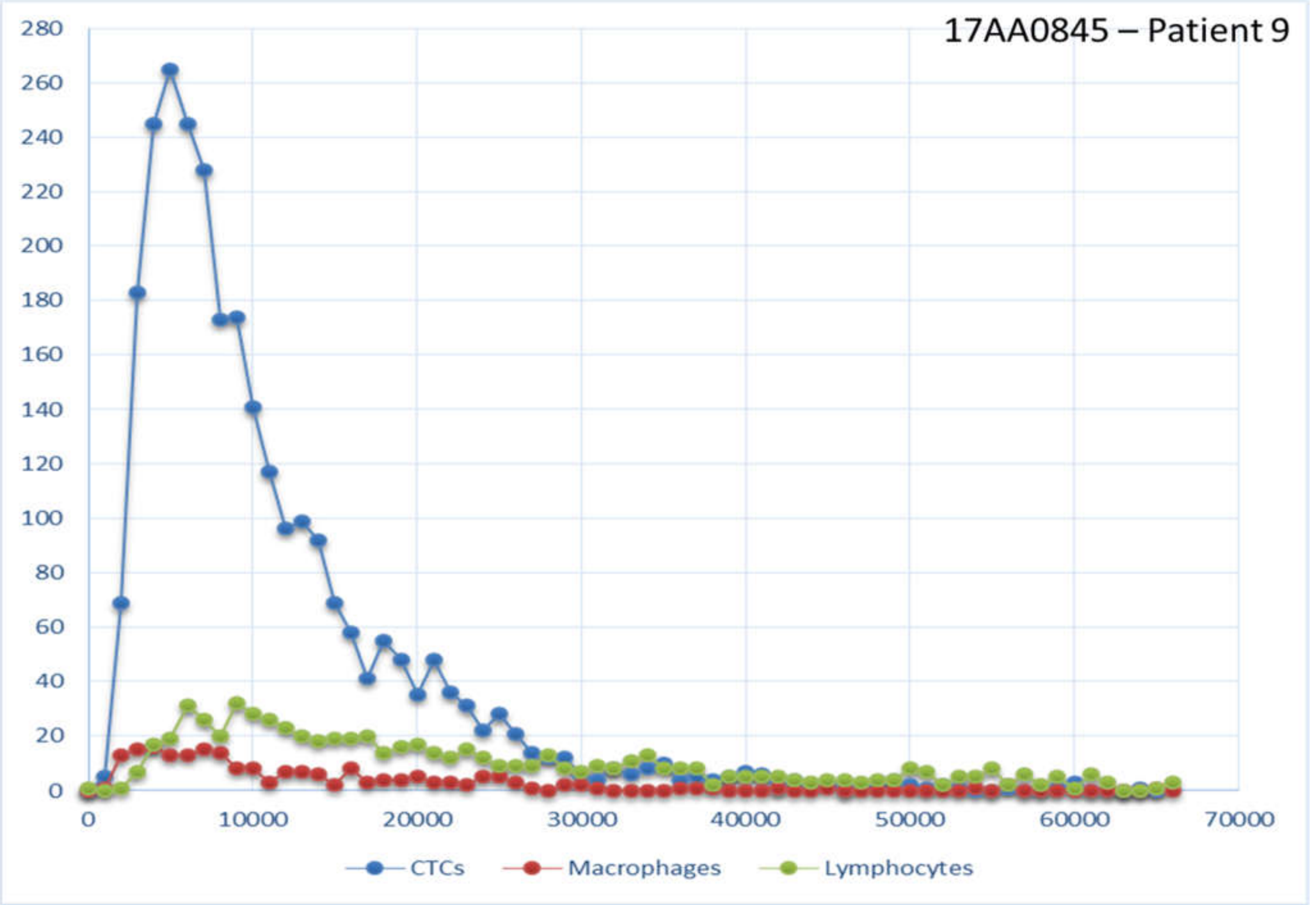
17AA0845 – Patient 9
280
260
240
220
200
180
160
140
120
100
80
60
40
20
0
0
10000
20000
30000
40000
50000
60000
70000
CTCs
Macrophages
Lymphocytes

Supplementary Figure S1: Representative figures of the CTCs, CAMLs and lymphocytes 3D telomere profile all patients assigned to profile 1 (Patients 3, 6, 7, 8 and 9) and profile 2 (Patients 1 and 1.1, 2, 4 and 5). In each graph, the telomere length is shown in arbitrary units of fluorescence (AU). The CTC, CAMLs (macrophages) and Lymphocytes are demarked with colors.
