# Supplementary material for: Telomere-Associated Changes in Nuclear Architecture of Cancer-Associated Macrophage-like Cells in Liquid Biopsies from Melanoma Patients
Source: Biomedicines. 2022 Sep 25;10(10):2391. doi: 10.3390/biomedicines10102391 (PMC9598704; doi:10.3390/biomedicines10102391)
Supplement: Supplementary file 1 [file biomedicines-10-02391-s001.zip › Supplemetary Table S1.pdf]

Patients	Sampling date	Melanoma	BRAF	Age	Stage	Number of metastatic sites	LDH (240-480)	Systemic treatment	Start Date Stop date	Best response	Date of best response	Date of progression	Death	Last news
**VS-1-018-ET3**	1 Feb 2017	Mucosal, nasal cavity	WT	41	IV M1c	skin, lymph node, bone, lung, liver, kidney, digestive	432	Temozolomid	30/Dec/2016 and 22/Feb/2017	Progression	1 Feb 2017	1 Feb 2017	26 July 2017	NA
**TM-1-019-M1**	8 Feb 2017	SSM, Calf	BRAF V600E	66	IV M1c	skin, bone, brain	2235	Dabrafenib + Trametinib	16/Jan/2017 and 14/Apr/2017	Progression	14 Abril 2017	14 April 2017	24 May 2017	NA
**LM-1-039-CL2**	17 May 2017	Unclassifiable, feet	WT	73	IV M1c	skin, lymph node, bone, liver	843	No treatment	17/May/2017 and 05/Jul/2017	NA	NA	NA	5 July 2017	NA
**BJ-1-071-ET4**	10 May 2017	Nodular, trunk	WT	88	IV M1c	skin, lymph node, bone	573	Pembrolizumab	02/Mar/2017 and 04/May/2017	Progression	10 March 2017	10 May 2017	3 Sep 2017	NA
**DA-1-075-ET2**	28 Feb 2017	SSM, trunk	BRAF V600E	73	IV M1c	lymph node, liver, brain	581	Clinical Trial Vemurafenib + Cobimetinib	05/Dec/2016 and 28/Mar/2017	Partial response	28 Feb 2017	28 May 2017	1 Sept 2017	NA
**VS-1-018-M1-3**	7 June 2017	Mucosal, nasal cavity	WT	41	IV M1c	skin, lymph node, bone, lung, liver, digestive, kidney, pancreas	481	Cobimetinib	22/Apr/2017 and 26/Jul/2017	Progression	10 March 2017	10 May 2017	26 July 2016	NA
**GF-1-080_ET1**	16 March 2017	SSM, abdominal wall	BRAF V600E	75	IV M1a	lymph node	378	Clinical Trial Pembrolizumab / placebo	21/Dec/2016 and 27/Mar/2017	Progression	15 March 2017	15 March2017	NA	20 June 2019
**LM-1-081_ET1**	7 March 2017	Unclassifiable Unknown origin	WT	73	IV M1c	skin, lymph node, bone, muscle, adrenal	545	No treatment	13/Feb/2017 and 05/May/2017	Progression	7 March 2017	7 March 2017	17 Oct 2018	NA
**AR-1-092_ET1**	15 March 2017	Unclassifiable Unknown origin	WT	65	IV M1c	lymph node, digestive, pancreas, muscle	772	Clinical Trial CheckMate 511 Ipilimumab + Nivolumab	22/Mar/2017 and 24/Jan/2018	Stable	15 June 2017	29 Nov 2017	2 Feb 2019	NA
**KJ-1-084_ET1**	19 March 2017	Unclassifiable, face	BRAF V600E	39	IV M1b	lung	431	Pembrolizumab	13/Feb/2017 and 27/Dec/2017	Stable	19 May2017	15 Sept 2017	13 Dec 2018	NA

Supplementary Table S1: Clinical information of all patients included in the study
